# London Dispersion Favors *Cis* Selectivity in the Johnson–Corey–Chaykovsky Epoxidation

**DOI:** 10.1021/acs.joc.6c00154

**Published:** 2026-03-26

**Authors:** Marvin H. J. Domanski, Lars Rummel, Saskia N. Krug, Heike Hausmann, Ephrath Solel, Peter R. Schreiner

**Affiliations:** † Institute of Organic Chemistry, 9175 Justus Liebig University, Heinrich-Buff-Ring 17, 35392 Giessen, Germany; ‡ EaStCHEM School of Chemistry, University of Edinburgh, Joseph Black Building, David Brewster Road, Edinburgh EH9 3FJ, United Kingdom

## Abstract

We elucidate the role of dispersion energy donors on the *cis*/*trans* selectivity of the Johnson–Corey–Chaykovsky epoxidation, focusing on bulky alkyl groups as well as halides as dispersion energy donors. Whereas the generally accepted explanation of the observed diastereoselectivity solely invokes steric repulsion, we determined that London dispersion interactions are an important source of stabilization of the preferred transition structures. This was brought forth experimentally by utilizing a series of NMR measurements, double mutant cycles, and crossover experiments. Additionally, density functional theory computations and symmetry-adapted perturbation theory computations were used to determine and quantify the role of noncovalent interactions.

## Graphical abstract

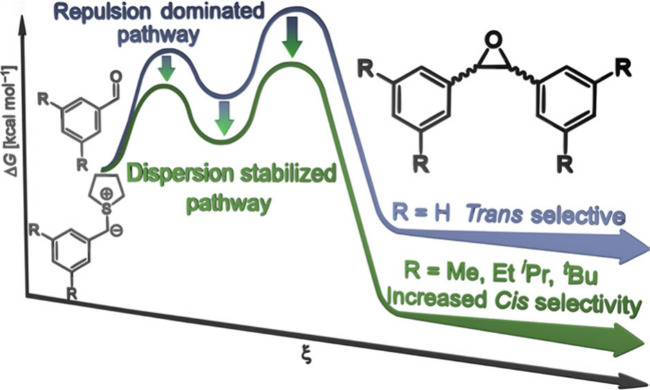

## Introduction

In chemistry, insights into reaction mechanisms are essential to maximize yields and stereoselectivities, especially the latter, which requires extensive knowledge of how molecules interact and connect via elementary reaction steps. The applicability of a detailed understanding of reaction pathways ranges from basic S_N_2 reactions^1^ proceeding via stereospecific backside attacks, to highly complex catalytic processes.^2^ In the past the origin of stereoselectivity was most often rationalized by repulsive steric interactions either between substrates or catalyst and substrate.^3^ While the concept of steric hindrance can typically explain the stereochemical outcome of reactions between *small* molecules and groups, bulky substituents counterintuitively can have the opposite effect through steric attraction.^4–6^ This has been recently demonstrated, for example, in the copper-catalyzed hydroamination of unactivated olefins,^7^ in paddlewheel catalysts,^8,9^ in the Brønsted acid-catalyzed transfer hydrogenation of imines,^10^ and for the Corey–Bakshi–Shibata reduction utilizing sterically highly encumbered oxazaborolidine catalysts.^11^ Here, the rate-determining step of the reduction proceeds through the more *crowded* transition state that profits from *attractive* noncovalent interactions. For hydroamination reactions it was demonstrated that increasing steric bulk promotes catalyst-substrate interactions, thereby accelerating the reaction.^7,12,13^ Both studies highlight the importance of London dispersion^14,15^ (LD) interactions as the key driving force for aggregation and as the essential interaction to rationalize the experimental results.^16^ To maximize LD, highly polarizable groups called dispersion energy donors (DEDs) were introduced as a concept and in practice.^4,17^

In comparison to ground-state stabilization via attractive σ–σ contacts, for example, in molecular balances,^18,19^ the preferential LD stabilization of transition states has not been thoroughly investigated. Here, we chose the Johnson–Corey–Chaykovsky (JCC) reaction^5,20–23^ to study the effects of DEDs on the stereodifferentiating transition states of this reaction. The JCC reaction takes place between an ylide and an aldehyde (Scheme 1); numerous procedures exist that give a variety of products, for example aziridines,^24^ cyclopropanes,^24,25^ and epoxides.^26–30^ Additionally, there are theoretical^31–34^ and experimental studies^35,36^ on the mechanism of the JCC reaction.

**Scheme 1:**
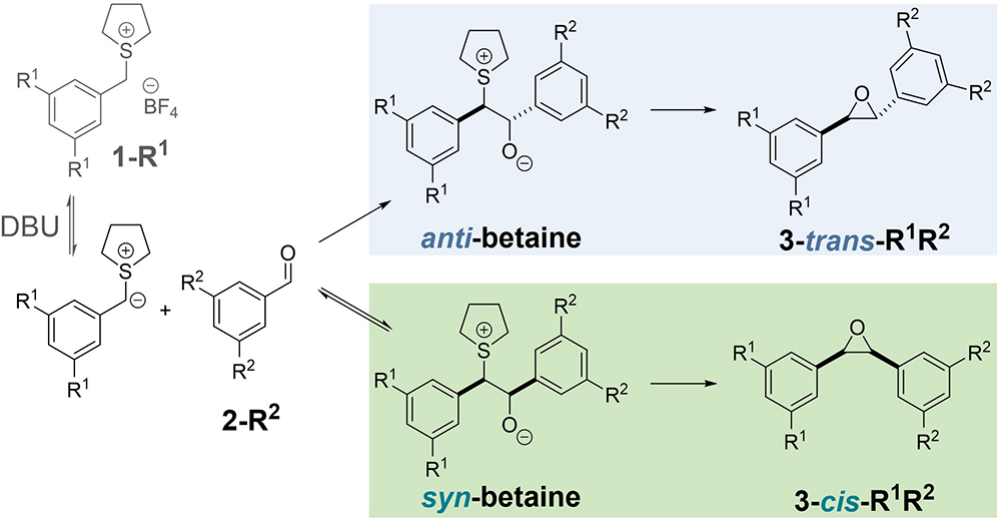
Currently Accepted Reaction Sequence of the Johnson–Corey–Chaykovsky Epoxidation

The current consensus is that after a pre-equilibrium deprotonation with base to generate the ylide from sulfonium salt **1-R** ^**1**^, the ylide adds nucleophilically to the aldehyde **2-R** ^**2**^ in the rate-determining step. Subsequent bond rotation around the central C–C bond gives the *syn*- or *anti*-betaine, followed by stereospecific elimination of sulfide to yield the *cis*- or *trans*-epoxide (**3-*cis*/*trans*-R** ^**1**^ **R** ^2^), respectively.

Experimentally, the Gibbs free energy of activation was determined as Δ*G* ^⧧^ = 22.2 kcal mol^–1^ (with R^1^ = H and R^2^ = H) at 298 K for the nucleophilic addition of the ylide to form the *trans*-epoxide.^35^ In contrast, previous computations suggested that the rotation around the central betaine C–C bond is rate-determining for the formation of the *cis*-epoxide.^34^ The origin of the diastereoselectivity lies in the reversibility of the *syn*-betaine formation on the one hand and the irreversibility of the *anti*-betaine formation (from crossover experiments) on the other. Accordingly, Aggarwal et al.^3^ outlined four main principles to rationalize stereoselectivity in the Johnson–Corey–Chaykovsky reaction: (1) steric repulsion between the phenyl moieties in the *syn*-betaine structure, (2) the inherent stability of the ylide, (3) the stability of the aldehyde, and (4) the role of solvation in promoting reversible *syn*-betaine formation. Herein, we investigate the role of DEDs in the JCC reaction, demonstrating the importance of LD in this reaction in particular and in stereoselective reactions in general.^16^

## Results

### Alkyl Substituents

To study the impact of DEDs on the JCC epoxidation, we systematically varied the substituent at **1-R** ^**1**^ and **2-R** ^**2**^ in the logical series from methyl (Me) through ethyl (Et) and isopropyl (^*i*^Pr) to *tert*-butyl (^*t*^Bu). We utilized 1-bromo-3,5-dialkyl-substituted benzene as starting material to generate all precursors. **2-R** ^**2**^ was prepared *via* a formylation reaction with DMF.^37^ The aldehyde was reduced utilizing LiAlH_4_, and the resulting alcohol was brominated *via* PBr_3_.^38,39^ A Finkelstein-type reaction with tetrahydrothiophene and NaBF_4_ yielded **1-R** ^**1**^.^40^ In addition, we utilized halides as DEDs in the all-*meta* position. Starting from 3,5-dihalo-substituted benzaldehyde, we generated the logical series from fluorine (F) to chlorine (Cl) and bromine (Br) at **1-R** ^**1**^ and **2-R** ^**2**^. The iodine­(I)-substituted starting material was synthesized from benzocaine according to Lindsey et al.^41^

To probe the effects of DEDs on the JCC reaction, the reaction conditions were chosen in analogy to Crudden et al.^35^ who experimentally investigated the mechanism of the JCC reaction. The epoxidation was performed in DCM under pseudo-first order kinetic conditions. Accordingly, the aldehyde was utilized in excess (0.5 M, 10 equiv) with limiting sulfonium salt (1.0 equiv) present. A buffer (1.0 equiv) of 1,8-diazabicyclo(5.4.0)­undec-7-ene (DBU) and *p*-toluenesulfonic acid (*p*-TSA) was utilized for a steady pH. Additionally, DBU (1.0 equiv) was added as a base to generate the ylide and to start the reaction. The reaction progress was monitored by ^1^H NMR spectroscopy at room temperature. The ratio of **3-*cis*-R** ^**1**^ **R** ^**2**^ and **3-*trans*-R** ^**1**^ **R** ^**2**^ was determined by integration of the *cis* and *trans* proton signals at the epoxide. Crudden et al.^35^ already demonstrated irreversibility of the parent system. To determine whether bulky substituents influence the reaction mechanism, we followed the reaction progress for **1-Me** and **2-Me** as well as **1-** ^***t***^ **Bu** and **2-** ^***t***^ **Bu** (see the Supporting Information (SI)). In both cases the product concentration steadily increased while the ratio between both epoxides remained constant. Additionally, we exposed a 1:1 mixture of **3-*trans*-HH** and **3-*cis*-HH** to the reaction conditions without observing a change in ratio (see the SI). Figure 1 shows the ^1^H NMR signals of the symmetric epoxides (R = R^1^ = R^2^) utilizing the alkyl substitution pattern with a chemical shift between 3.75 and 4.40 ppm.

**Figure 1:**
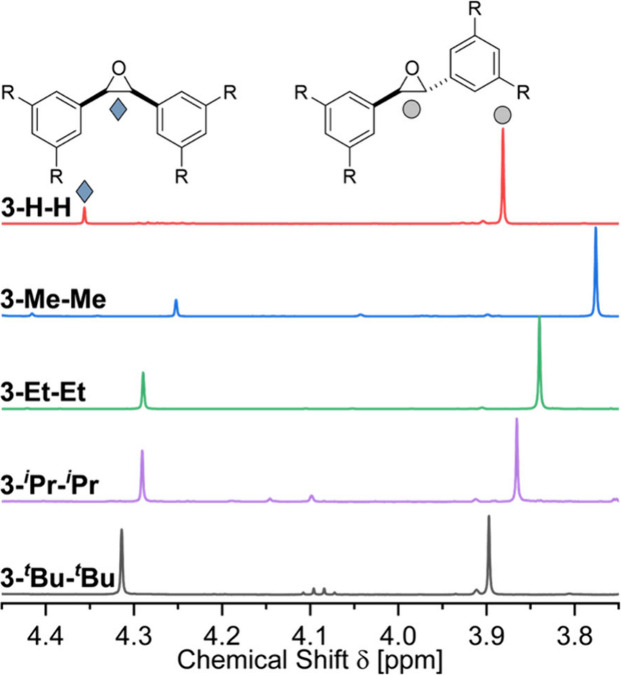
^1^H NMR measurements of symmetric **3-*cis*-R** ^**1**^ **R** ^**2**^ (blue marking) and **3-*trans*-R** ^**1**^ **R** ^**2**^ (gray marking) as a result of the reaction of **1-R** ^**1**^and **2-R** ^**2**^ with alkyl substituents in DCM at 298 K. For simplicity, only the CH signals of the symmetric epoxides are depicted. It is already apparent from the relative intensities that the amount of *cis*-epoxide increases with steric bulk.

The parent unsubstituted starting material resulted in a product ratio of 0.17:1.00 (*cis*:*trans*) (Figure 1, red NMR). In accordance with the literature,^35^ the larger signal (gray marking) was assigned to **3-*trans*-HH** and the smaller signal (blue marking) to **3-*cis*-HH**. The generation of **3-*trans*-HH** and **3-*cis*-HH** from stilbene with *m*-chloroperoxybenzoic acid (*m*CPBA) confirmed our assignment (see the SI). To gather as much information as possible on the influence of DEDs on the reaction we followed our procedure for all possible **1-R** ^**1**^ and **2-R** ^**2**^ combinations. The results for the symmetric alkyl-substituted products are depicted in Figure 1. Counterintuitively, the epoxide ratio (*cis*:*trans*) shifts to the more crowded **3-*cis*-R** ^**1**^ **R** ^**2**^ with increasing DED size. Accordingly, the ratio of **3-*trans*-** ^***t***^ **Bu** ^***t***^ **Bu** and **3-*cis*-** ^***t***^ **Bu** ^***t***^ **Bu** (Figure 1, black) is almost 1:1 (see the SI for all exact ratios).

Figure 2 displays the experimentally determined Δ*G*_R^1^R^2^‑HH_^⧧^ values for the reaction between differently alkyl-substituted tetrahydrothiophene salts **1-R** ^**1**^ and aldehydes **2-R** ^**2**^ to generate mixtures of **3-*cis*-R** ^**1**^ **R** ^**2**^ and **3-*trans*-R** ^**1**^ **R** ^**2**^. The energy values are derived from integration of the *cis* and *trans* proton signals of the epoxide. The unsubstituted system was utilized as reference reaction. Δ*G*_R^1^R^2^‑HH_^⧧^ refers to the free energy of the rate-determining transition state relative to that of the parent unsubstituted reaction of **1-H** and **2-H**. Therefore, Δ*G*_R^1^R^2^‑HH_^⧧^ for the reaction of **1-H** and **2-H** is denoted as Δ*G* ^⧧^_HH‑HH_ = 0.0 ± 0.1 kcal mol^–1^ (leftmost data point). With an absolute free energy value of Δ*G* ^⧧^_HH_ = 1.0 ± 0.0 kcal mol^–1^ (see the SI for absolute values) the reaction of **1-H** and **2-H** favors the less crowded **3-*trans*-HH** over **3-*cis*-HH** (*K*_HH_^⧧^ = 0.17). In line with the argument that large groups such as phenyl moieties interact repulsively, the transition state leading to **3-*trans*-HH** is lower in energy by around 1.0 kcal mol^–1^ than its more crowded counterpart. With this result and classic “steric repulsion thinking” in mind, bulkier groups, *e.g.,* with an all-*meta* substitution pattern, should shift the ratio even further to **3-*trans*-R** ^**1**^ **R** ^**2**^ (Δ*G* ^⧧^ > 0).^3^ In stark contrast, Figure 2 shows the *opposite* trend. The attached substituents shift the ratio to the more crowded **3-*cis*** **-RR** (Δ*G* ^⧧^ < 0) contradicting the well-established rationale that increasing steric bulk predominantly favors **3-*trans*-R** ^**1**^ **R** ^**2**^. However, our results are well in line with investigations focusing on attractive σ–σ and σ–π interactions.^4,42–47^ Similar effects were observed in numerous catalytic processes highlighted earlier.^7–11^ In those cases, bulky substituents were utilized to lower the energy of the more crowded transition state by enforcing close σ–σ and σ–π contacts between catalyst and substrate. A similar effect appears to govern the JCC reaction as well.

**Figure 2:**
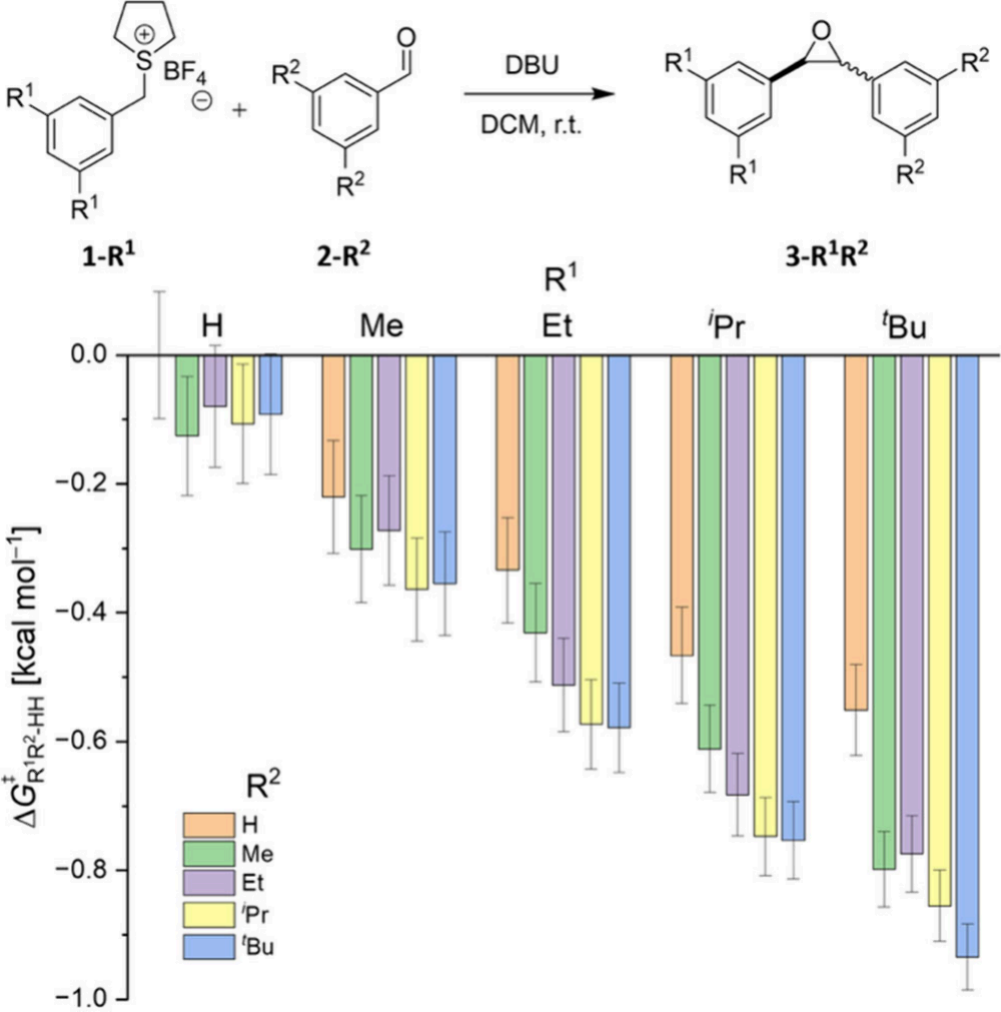
Experimentally determined relative Gibbs free energy values Δ*G*_R^1^R^2^‑HH_^⧧^ for the reaction of alkyl-substituted **1-R** ^**1**^ and **2-R** ^**2**^ at room temperature relative to the parent case with R^1^ = R^2^ = H (leftmost data point, *cis*:*trans* ratio of 0.17:1.00). The depicted energy values correspond to the energy differences of the transition states in the rate-determining step. Δ*G* ^⧧^ < 0 indicates favoring of **3-*cis*-R** ^**1**^ **R** ^**2**^ formation relative to the parent system. R^1^ = R^2^ = ^*t*^Bu results almost in a *cis*:*trans* ratio of 1:1. The gray lines indicate error bars.

To study the effect of LD on the transition state of the reaction, all combinations of **1-R** ^**1**^ and **2-R** ^**2**^ were measured. The unsubstituted **1-H** always favors the reaction forming **3-*trans*** **-HR** ^**2**^ (leftmost block of columns). The energy difference of the transition states does not change with increasing substituent size at **2-R** ^**2**^ (Δ*G*_HR^2^‑HH_^⧧^ ≈ −0.1 ± 0.1 kcal mol^–1^). On the other hand, in a reaction with unsubstituted benzaldehyde **2-H** (orange bars) increasing substituent size at **1-R** ^**1**^ decreases the energy gap between the two transition states. Stabilizing σ–π interactions appear to decrease the energy of the *more crowded* transition state. While the transition state to **3-*cis*** **-MeH** benefits by around Δ*G*_MeH‑HH_^⧧^ ≈ −0.2 ± 0.1 kcal mol^–1^ from stabilizing σ–π contacts, the incorporation of additional CH_3_ substituents increases this effect by around −0.1 kcal mol^–1^. In the series of singly substituted starting materials (orange bars and leftmost block of columns), the largest effect can be observed for the reaction of **1-** ^***t***^ **Bu** and **2-H** (Δ*G*_^*t*^BuH‑HH_^⧧^ ≈ −0.6 ± 0.1 kcal mol^–1^). In stark contrast to the well-established rule-of-thumb that steric hindrance governs the transition state of the JCC reaction, an increase in substituent size at **1-R** ^**1**^ and **2-R** ^**2**^ shifts the *dr* toward the more crowded **3-*cis*-R** ^**1**^ **R** ^**2**^. The systematic increase of **2-R** ^**2**^ within each series of **1-R** ^**1**^ identifies the *tert*-butyl substituent (blue bars) as the best DED. The strongest interaction can be observed for the reaction of **1-** ^***t***^ **Bu** and **2-** ^**t**^ **Bu** almost resulting in a 1:1 mixture (*K*_^*t*^Bu^*t*^Bu_^⧧^ = 0.93). Since we are under kinetic conditions, the transition state along the reaction to the more crowded **3-*cis*** **-** ^***t***^ **Bu** ^***t***^ **Bu** is favored by around Δ*G*_^*t*^Bu^*t*^Bu‑HH_^⧧^ ≈ −0.9 ± 0.1 kcal mol^–1^ relative to its parent counterpart.

### Halogen Substituents

Having studied the alkyl substitution pattern, we shifted our focus to halogen substitution. While recent studies^46,48–56^ demonstrated that halogens can act as DEDs, polar effects could potentially override LD interactions. The same procedure as for the alkyl-substituted starting material was applied for reactions between differently halo-substituted tetrahydrothiophene salts **1-R** ^**1**^ and aldehydes **2-R** ^**2**^. In addition, we expanded our scope and included all possible alkyl-halogen combinations by varying **1-R** ^**1**^ and **2-R** ^**2**^ systematically. Since **1-F** was insoluble in DCM, it had to be excluded.

Moreover, all possible combinations of aldehydes with all-*meta* halogen-substituted tetrahydrothiophene salts **1-R** ^**1**^ resulted only in **3-*trans*-R** ^**1**^ **R** ^**2**^. This implies that secondary effects, such as electronegativity, significantly influence the behavior of **1-R** ^**1**^. Interestingly, this is not the case for the halo-substituted **2-R** ^**2**^. Figure 3 displays the experimentally determined Δ*G*_R^1^R^2^‑HH_^⧧^ values for the reaction of alkyl-substituted **1-R** ^**1**^ and halo-substituted **2-R** ^**2**^. For the reaction of **1-H** and **2-H**, Δ*G*_HH‑HH_^⧧^ = 0.0 ± 0.1 kcal mol^–1^ (leftmost data point). In contrast to alkyl–alkyl interactions (Figure 2), the reactions of **1-H** or **1-Me** (leftmost and second leftmost block of columns, Figure 3) with halo-substituted **2-R** ^**2**^ result in the formation of more **3-*trans*** **-HR** ^**2**^ in comparison to the parent system. Consequently, the electronic effects due to halogen incorporation increase the energy difference of the transition states. This effect is most obvious for the reaction of **1-H** and **2-F**. Here, the energy difference of the transition states increases to Δ*G*_HF‑HH_^⧧^ ≈ 0.5 ± 0.1 kcal mol^–1^. Nevertheless, bulkier halogens, such as iodine, shift the *dr* again toward the more crowded **3-*cis*-R** ^**1**^ **R** ^**2**^. While the energy difference of the transition states for the reaction of **1-** ^**i**^ **Pr** and **2-F** is about the same as of **1-H** and **2-H** (and essentially thermoneutral), combinations of bulky alkyl groups and halogens clearly favor the more crowded transition states. By utilizing **1-** ^***t***^ **Bu** and **2-I** (the biggest and bulkiest substituents incorporated), the transition state to the more crowded **3-*cis*** **-** ^***t***^ **BuI** is favored by around Δ*G*_^*t*^BuI‑HH_^⧧^ ≈ −0.6 ± 0.1 kcal mol^–1^.

**Figure 3:**
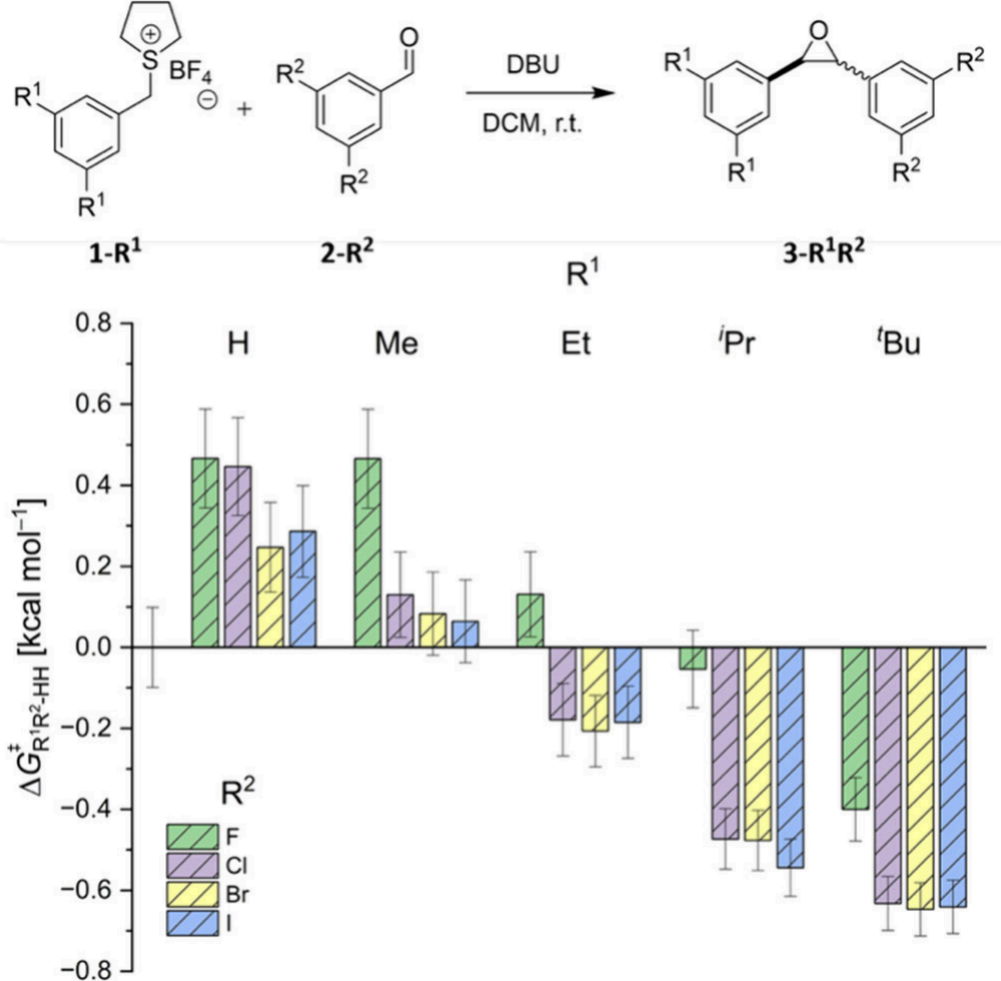
Experimentally determined relative Gibbs free energy values Δ*G*_R^1^R^2^‑HH_^⧧^ for the reaction of alkyl-substituted **1-R** ^**1**^ and halo-substituted **2-R** ^**2**^ relative to the parent case with R^1^ = R^2^ = H (leftmost data point). The depicted energy values correspond to the energy differences of the transition states in the rate-determining step. Δ*G* ^⧧^ < 0 indicates favoring of **3-*cis*-R** ^**1**^ **R** ^**2**^ formation with regards to the parent system. The gray lines indicate error bars.

## Discussion

As described in the introduction, four main factors were made responsible for diastereoselectivity of the reaction between sulfur ylide and aldehyde. On the basis of our experimental data, three of these can be re-evaluated and refined (differential solvation is not part of our study).

### Stability of the Carbonyl Group

Aggarwal et al.^3^ observed a significant increase in *trans* diastereoselectivity when utilizing aromatic instead of aliphatic aldehydes. While the formation of the *syn*-betaine structure is reversible (Scheme 1), stabilization of the starting material allows for an increase in *trans*-betaine formation. Aryl moieties stabilize the carbonyl form over the betaine structure. Accordingly, high *dr* is expected for benzaldehyde derivatives. Additionally, this effect should be amplified by electron-donor substituents due to an increase in electron density (for alkyl substituents) at the carbonyl carbon resulting in lower electrophilicity.^3^ However, our experimental data show the opposite selectivity. While unsubstituted phenyl moieties favor **3-*trans*-R** ^**1**^ **R**,^2^ the attachment of DEDs clearly shifts the ratio toward **3-*cis*-R** ^**1**^ **R** ^**2**^ (for example, *K*_^*t*^Bu^*t*^Bu_^⧧^ = 0.93). The same is true for halogen substituents at **2-R** ^**2**^, although the relative Gibbs free energy values Δ*G*_R^1^R^2^‑HH_^⧧^ are shifted toward **3-*trans*-R** ^**1**^ **R** ^**2**^. Accordingly, we deduce that the stability of the carbonyl group is less important and conclude that the action of increasingly larger alkyl groups at the aryl ring mainly act through stabilizing LD interactions.

### Stability of the Ylide

In line with the argument above, Aggarwal et al.^3^ found that the more stable the ylide, the higher the *trans* selectivity. Accordingly, electron-deficient functional groups increase diastereocontrol, whereas electron-rich groups yield lower *dr*. This statement is in line with our experimental findings. The attached alkyl groups decrease stereocontrol. The energy gap between both rate determining transition states is equal in height to result in 1:1 mixture of *cis* and *trans* epoxide with bulky *tert*-butyl groups attached. At the same time, the *dr* increases to high *trans* selectivity with electron-deficient functional groups, such as halogens. The halogen-substituted **1-R** ^**1**^ resulted in **3-*trans*-R** ^**1**^ **R** ^**2**^. Nevertheless, our experimental findings let us conclude that the stability of the ylide is not the only factor for stereocontrol but that this also depends on the fine balance between attractive and repulsive interactions. A combination of an increasing number of close LD contacts and a higher electron density maximize LD interactions in the more crowded transition state.

Since the first two principles are based on reversible betaine formation, we performed ring closure and crossover experiments to investigate the reversibility for the unsubstituted and heavily substituted alkyl systems. Hence, we synthesized the protonated diastereomers of betaine intermediates **4-*anti*-R** and **4-*syn*-R** (Scheme 2) to probe the epoxide formation starting with a single diastereomer. The starting materials were generated from *cis*- and *trans*-stilbene after epoxidation and stereoselective ring opening with thiomethoxide, methylation with methyliodide yields **4-*syn*-H** and **4-*anti*-H** selectively.^57^ The *tert*-butyl derivative was synthesized accordingly. Hereby, the *cis*- and *trans*-stilbene derivatives were prepared from hydrogenation of bis­(3,5-di-*tert*-butylphenyl)­acetylene, which was the product of a coupling reaction with 2-butynedioic acid.^58^

**Scheme 2:**
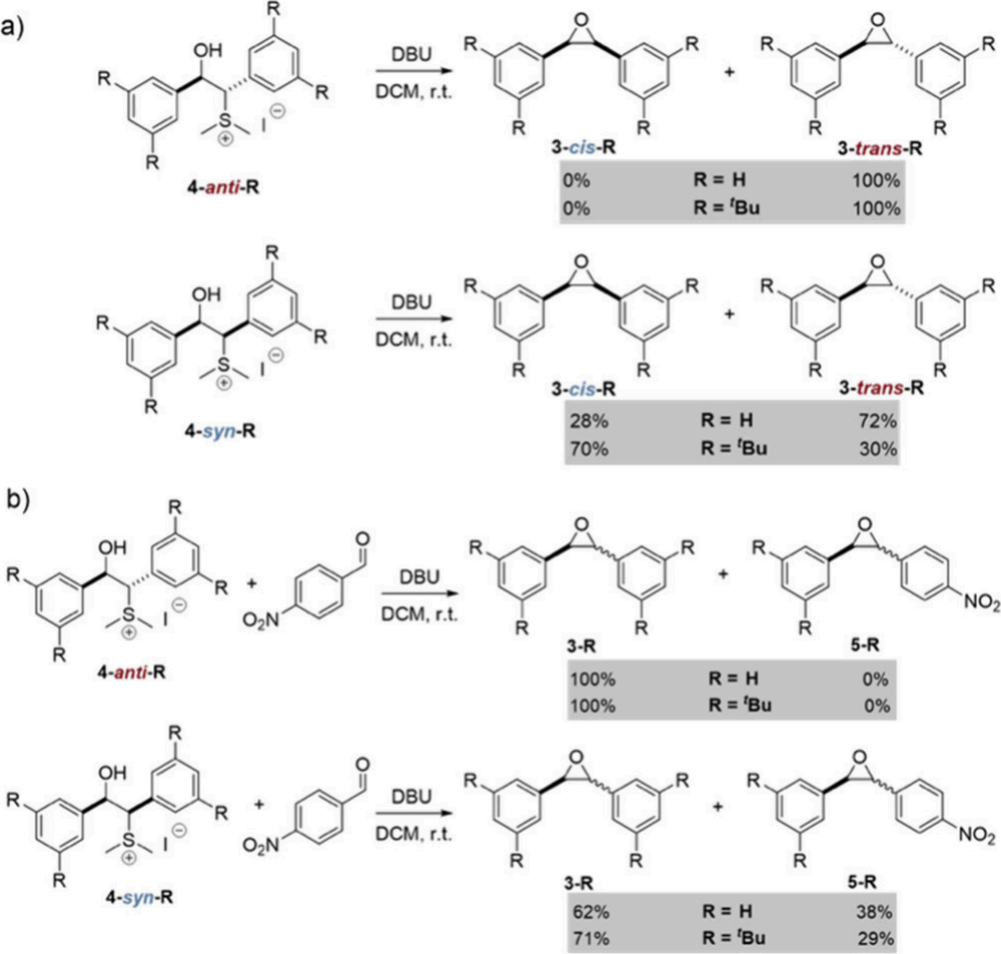
Ring Closure Experiments (top) and Crossover Experiments (bottom) of **4-*anti*-R** and **4-*syn*-R** in DCM at Room Temperature with DBU as the Base

The ring closure experiments (Scheme 2, top) of **4-*anti*-R** and **4-*syn*-R** were performed at room temperature in DCM by adding 1.0 equiv of base (DBU). With **4-*anti*-R** with R = H or ^*t*^Bu, the less crowded **3-*trans*-R** ^**1**^ **R** ^**2**^ forms exclusively. This implies that the rate-determining step for the JCC reaction has to occur *prior* to the final epoxidation step (thus betaine formation). In line with earlier reports,^29,30,36,57,59,60^ the epoxidation of **4-*syn*-H** results in a 28:72 *cis*:*trans* product ratio. Thus, the rate-determining step of **3-*cis*-HH** formation lies *after* betaine formation. Upon deprotonating **4-*syn*-H** the *syn*-betaine can either be directly converted to **3-*cis*-HH** or equilibrate to form the starting material (Figure 4). Recombination of 1*-R (the asterisk indicates minor changes of the molecule to **1-R**) and **2-R** also results in a *cis* and *trans* product mixture.

**Figure 4:**
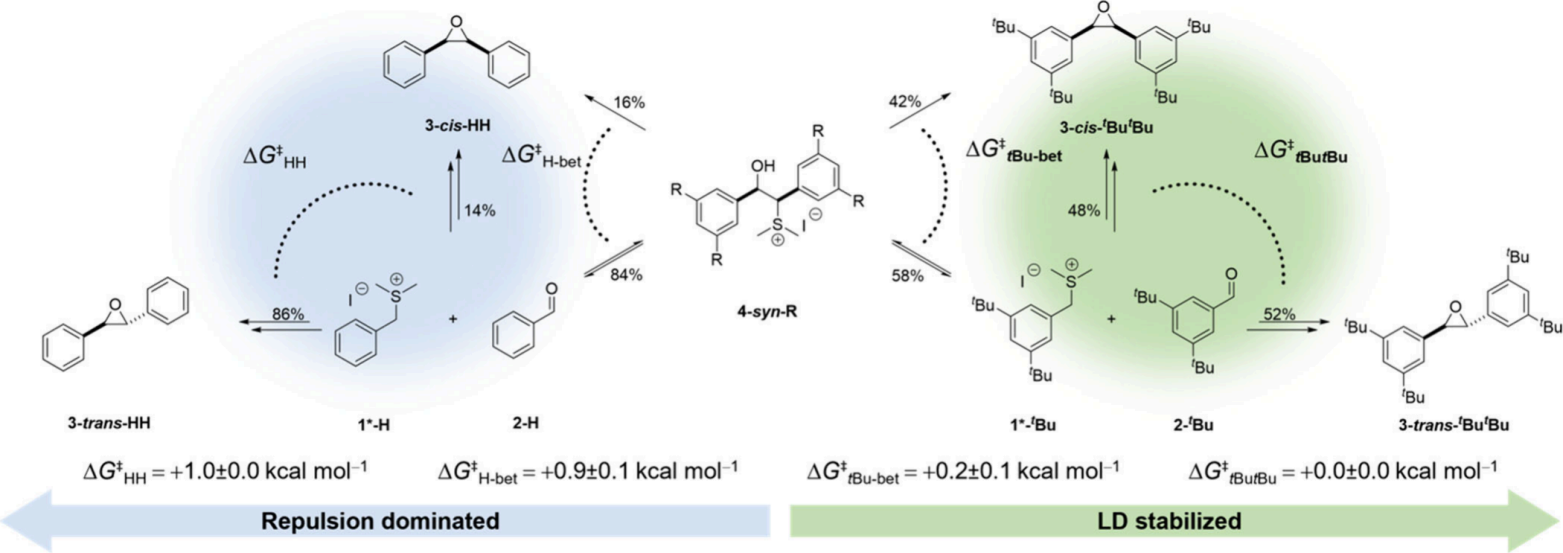
Reaction path shown in its protonated form for ring closure experiments of **4-*syn*-R** with R = H (left, blue) and R = ^*t*^Bu (right, green). The percentage give the probability of conversion of **4-** *syn* **-R** and recombination of 1*-R and **2-R**. The Gibbs free energy values correspond to the difference in transition state of the conversion of **4-*syn*-R** (Δ*G*_R‑bet_^⧧^) and for rate-determining transition states of recombination of 1*-R and **2-R** (Δ*G*_R^1^R^2^_^⧧^).

Under the assumption that molecular changes in 1*-R are minor and that, in a second approximation, the ratio of the pseudo-first order kinetic experiments is transferable to a reaction of **1-R** and **2-R** under thermodynamic conditions (control experiments confirm this assumption; see the SI), Δ*G*_R^1^R^2^‑HH_^⧧^ (Figure 2) can be utilized to calculate the probability for the conversion of **4-*syn*-R**. The ratio for the reaction of **4-*syn*-H** in combination with results of the JCC epoxidation to form **3-*cis*/*trans*-HH** (Figure 2) corresponds to a theoretical difference of Δ*G*_H‑bet_^⧧^ ≈ +0.9 ± 0.1 kcal mol^–1^ for the transition states of the conversion (ring closure reaction or equilibration) according to probability calculations. This suggests that the majority of **4-*syn*-H** (∼84%) equilibrates while the remaining 16% are directly converted to **3-*cis*-HH** (Figure 4). The same effect can be observed for **4-*syn*-** ^***t***^ **Bu**. Remarkably, now the final ratio for the consumption of **4-*syn*-** ^***t***^ **Bu** is 70:30 (*cis*:*trans*) in favor of the more crowded product **3-*cis*** **-** ^***t***^ **Bu** ^***t***^ **Bu**, suggesting a significant change in transition structures for the conversion of **4-*syn*-** ^***t***^ **Bu**. The ratio corresponds to a theoretical energy difference of Δ*G*_^*t*^Bu‑bet_^⧧^ ≈ +0.2 ± 0.1 kcal mol^–1^, thereby, slightly favoring conversion to **1-** ^***t***^ **Bu** and **2-** ^***t***^ **Bu**. Accordingly, 42% of **4-*syn*-** ^***t***^ **Bu** are directly converted to **3-*cis*** **-** ^***t***^ **Bu** ^***t***^ **Bu** while the remaining 58% equilibrate to **1-R** and **2-R** (Figure 4). The direct formation of **3-*cis*** **-** ^***t***^ **Bu** ^***t***^ **Bu** from **4-*syn*-** ^***t***^ **Bu** significantly increases in comparison to parent **4-*syn*-H**. This can be related to additional LD interactions favoring the folded conformation **4-*syn*-** ^***t***^ **Bu** with the hydroxyl function in the *anti* position to the thioether functional group.

A similar DED-effect was already observed for hexaphenylethane.^61–66^ While the unsubstituted hexaphenylethane is not thermodynamically stable due to steric hindrance,^65^ the *tert*-butyl-substituted derivative offers multiple LD contacts to stabilize the molecule.^63^ Likewise, the unsubstituted reaction pathway of **4-*syn*-H** is governed by repulsive interactions. On the other hand, LD interactions stabilize the folded *syn*-betaine structure, thereby increasing the probability of direct conversion to **3-*cis*-** ^***t***^ **Bu** ^***t***^ **Bu**.

To demonstrate that an equilibration must occur by reopening the betaine structure, we performed crossover experiments (Scheme 2, bottom).^29,30,36,57,59,60^ Activated *p*-nitrobenzaldehyde was utilized as trapping agent for the ylide due to its higher reactivity (reactions proceed around 62 times faster than with benzaldehyde).^36^ Whereas **4-*anti*-R** with R = H or ^*t*^Bu solely results in the formation of **3-*trans*-R** ^**1**^ **R** ^**2**^ without incorporating nitrobenzaldehyde, **4-*syn*-R** gives a mixture of **3-*trans*-R** ^**1**^ **R** ^**2**^ and the corresponding nitro-product **5-R** (Scheme 2b). This demonstrates that the equilibration of the *syn*-betaine has to occur by breaking the central carbon bond to give ylide and aldehyde.

### Steric Hindrance of the Ylide/Aldehyde

Finally, steric hindrance is widely believed to affect the stereochemical outcome of the JCC reaction.^3^ While this may be true for special cases, it is unlikely the sole contributor to selectivity because all bulky substituents used here (and elsewhere) show the opposite (Figure 2): The parent reaction of **1-H** with **2-H** yields the highest *trans* selectivity with a transition state energy difference of Δ*G*_HH_^⧧^ = 1.0 ± 0.0 kcal mol^–1^). On the contrary, the reaction of **1-** ^***t***^ **Bu** with **2-** ^***t***^ **Bu** results in an approximately 1:1 mixture of **3-*cis*** **-** ^***t***^ **Bu** ^***t***^ **Bu** and **3-*trans*-** ^***t***^ **Bu** ^***t***^ **Bu** (i.e., Δ*G*_^*t*^Bu^*t*^Bu_^⧧^ ≈ 0.0 kcal mol^–1^). Consequently, the energy gap of around 1.0 ± 0.0 kcal mol^–1^ between the two diastereomeric transition states of the parent case vanishes due to the attachment of bulky *tert*-butyl groups.

To shed more light on the origin of the transition state stabilization, we dissected the ΔΔ*G*_R^1^R^2^_^⧧^ energy values by mutating all substituents separately. By comparing and applying this against energies of singly substituted reactions the interaction energy ΔΔ*G*_R^1^R^2^_^⧧^ can be dissected (Figure 5, top). ΔΔ*G*_R^1^R^2^_^⧧^ solely resembles the interaction energy between both alkyl groups R^1^ and R^2^, thereby disregarding the energy gain due to close σ–π contacts. For this procedure^67,68^ (termed a double mutant cycle, a practical application of Hess’ law) we applied the following equation:

1 $$\Delta \Delta G_{R^{1}R^{2}}^{⧧}=\Delta G_{R^{1}R^{2}}^{⧧}-\Delta G_{R^{1}H}^{⧧}-\Delta G_{HR^{2}}^{⧧}+\Delta G_{HH}^{⧧}$$

**Figure 5:**
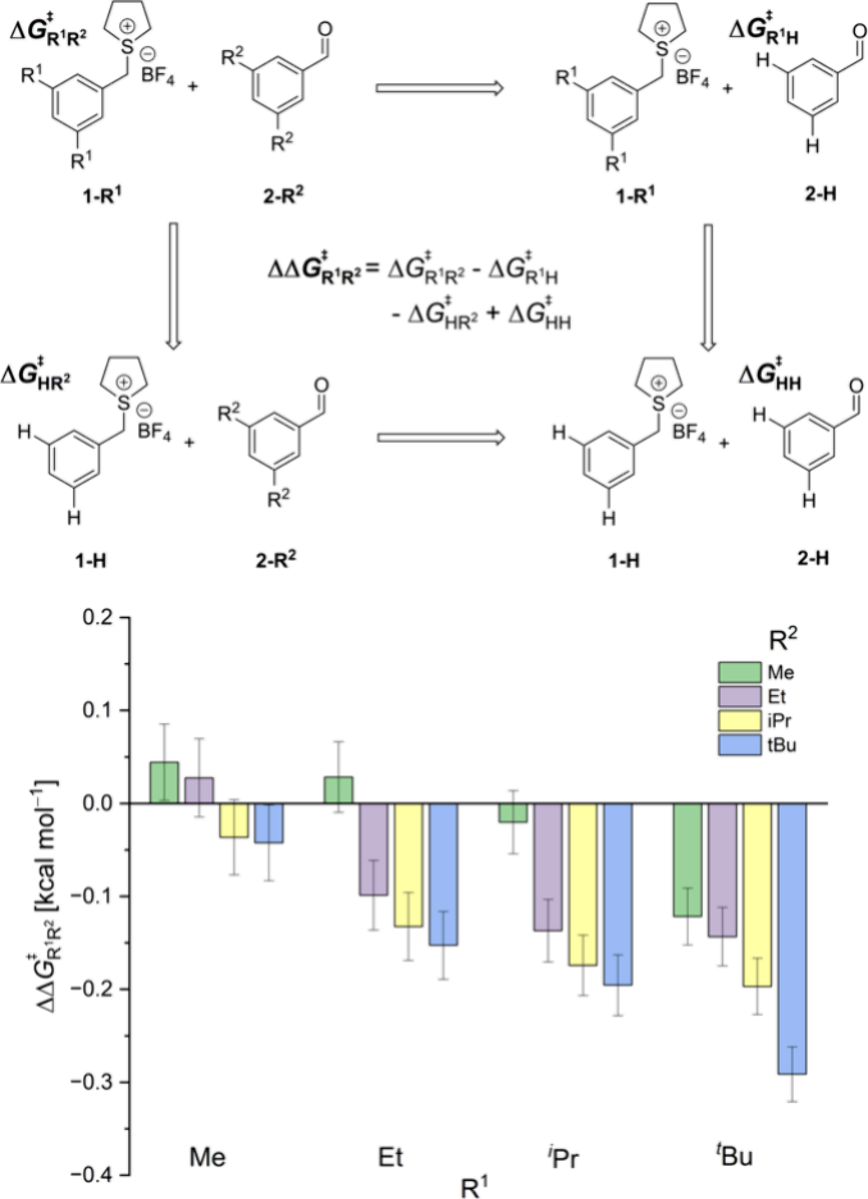
Double mutant cycle (top) and experimental results of the analysis (bottom). ΔΔ*G*_R^1^R^2^_^⧧^ illustrates the interaction energy between R^1^ and R^2^ in the reaction of **1-R** ^**1**^ with **2-R** ^**2**^. Negative energy values correspond to stabilizing interactions between R^1^ and R^2^. Gray lines indicate error bars.

The result provides an experimental estimate of the role DEDs play in the reaction of ylide and aldehyde. While all combinations were measured for alkyl–alkyl interactions, the double mutant cycle could not be determined for halogen substituents since all-*meta* halogen-substituted tetrahydrothiophene salt **1-R** ^**1**^ resulted only in **3-*trans*-R** ^**1**^ **R** ^**2**^. In contrast to the concept of steric repulsion, Figure 5 (bottom) qualitatively highlights the stabilizing effect (negative energy values) of each DED in the transition state of the reaction.

Apart from Me-Et combinations, all energy values are negative corresponding to stabilizing interactions between the alkyl substituents. While this effect is small for **1-Me** (leftmost block of columns), the stabilization increases with DED size, i.e., polarizability. Consequently, the largest effect can be observed for ^*t*^Bu–^*t*^Bu contacts (ΔΔ*G*_^*t*^Bu^*t*^Bu_^⧧^ = −0.3 ± 0.1 kcal mol^–1^). By comparing the relative Gibbs free energy values Δ*G*_R^1^R^2^‑HH_^⧧^ (Figure 2) to the results of the double mutant cycle (Figure 5), the interaction energy between each DED can be dissected into σ–σ interactions (between DEDs) and σ–π interactions (between DED and phenyl system). Accordingly, the energy difference Δ*G*_^*t*^Bu^*t*^Bu‑HH_^⧧^ ≈ −0.9 ± 0.1 kcal mol^–1^ for the reaction of **1-** ^***t***^ **Bu** with **2-** ^***t***^ **Bu** (Figure 2) contains stabilizing σ–σ interactions of ΔΔ*G*_^*t*^Bu^*t*^Bu_^⧧^ = −0.3 ± 0.1 kcal mol^–1^ (Figure 5). Around 30% of the experimentally determined relative Gibbs free energies can be accounted for with LD attractive alkyl–alkyl contacts. The remaining 70% correspond to LD attractive σ–π interactions. These results qualitatively fit to measurements performed for molecular balances in the ground state.^47^

Most recently, *N*,*N*-diphenylthiourea was shown to experience the same distribution of σ–σ and σ–π interactions to stabilize the most crowded *syn*,*syn* conformer.^47^ Accordingly, the observations and values concerning the role of LD in molecular balances can be directly transferred to transition state stabilizations, but might we weaker due to the loosely bound nature of transition states.^18^

## Computations

There have been previous computational studies on the Corey–Chaykovsky mechanism,^31–34^ but to the best of our knowledge, none considered the effect of LD interactions on the rates and selectivity. The mechanism of the Corey–Chaykovsky reaction can, generally, be divided into three stages, according to Figure 6:

1. **C–C bond formation:** This stage is shown in red in Figure 6. Formation of noncovalently bound complexes of the reactants, which then react to form the bond between the two carbon atoms (the SC–CO bond) leading to the betaines. The relative orientation of the oxygen and sulfur groups around the new C–C bond can be either *gauche* or *anti.* If irreversible, this step determines the stereochemistry of the epoxide product.
2. **Rotation around the C–C bond:** This stage is shown in black in Figure 6. Once one of the above-mentioned betaine rotamers is formed, rotation around the SC–CO bond can interconvert them. The *gauche* and *gauche*′ rotamers are lower in energy than the *anti* rotamers, and interchange between the two possible *gauche* rotamers (via **TS3**) has a low barrier, so that they are in equilibrium with one another. The barriers to form the *anti* betaine (**TS1** and **TS2**) are higher in energy.
3. **Ring closing to form the epoxide:** This stage is shown in blue in Figure 6. Once the *anti*-betaine has formed via either of the two former stages, it can react further in an S_N_2-type reaction to form the epoxide product.

**Figure 6:**
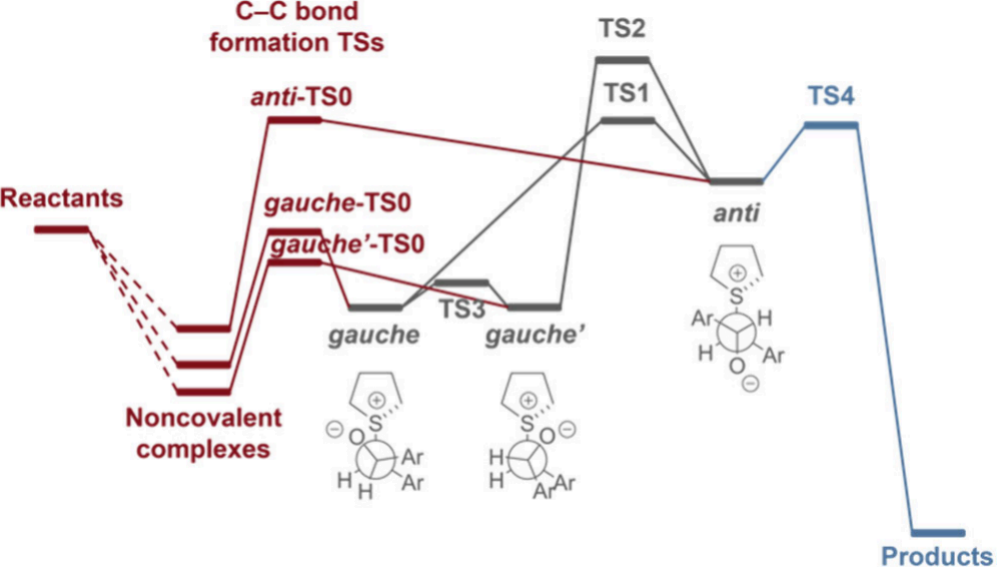
Schematic representation of the complete reaction profile for one of the possible anti configurations (leading to a *trans*-epoxide product).

A suggested route through a [2 + 2] cycloaddition to form a four-membered oxathietane, resembling the Wittig oxaphosphetane intermediate, that then directly forms the epoxide, was previously shown to be significantly higher in energy.^33^ In our computations the oxathietanes were identified as nonstationary structures.

From the above, there are two possible routes for the formation of the *anti* betaine: (1) direct formation from the reactants (through *anti*-**TS0**) or (2) formation of either of the *gauche*/*gauche*′ rotamers (through *gauche*-**TS0**/*gauche*′-**TS0**) and then conversion to the *anti* rotamer through **TS1** or **TS2**. Practically, this means that formation of the *anti* rotamer can happen through at least one of five possible pathways, represented by the TSs along the path: (a) *anti*-**TS0**; (b) *gauche*-**TS0**, **TS1**; (c) *gauche*′-**TS0**, **TS2**; (d) *gauche*-**TS0**, **TS3**, **TS2**; (e) *gauche*′-**TS0**, **TS3**, **TS1**.

In order to determine which of these five routes is preferred, we computed all intermediates and TSs using the B3LYP^69,70^ /def2-TZVPP^71^ level of theory, with Grimme’s D3­(BJ) dispersion correction^72,73^ for unsubstituted Ph (R^1^ = R^2^ = H, Figure 7) and for the ^*t*^Bu-substituted reactants (R^1^ = R^2^ = ^*t*^Bu, Figure 9). To match the experimental conditions, our computations included dichloromethane (DCM) as an implicit solvent, using the solvation model based on density (SMD).^74^ All reported energies are ZPVE-corrected, unless stated otherwise. The highest TS for each of the five routes was found, and this was defined as the barrier value for this route. The pathway with the lowest barrier value will be the preferred one. This process was done for four possible conformers of each of the *anti*-betaines leading to the *cis*- and *trans*-epoxides (**A**–**H**, Figure 8)

**Figure 7:**
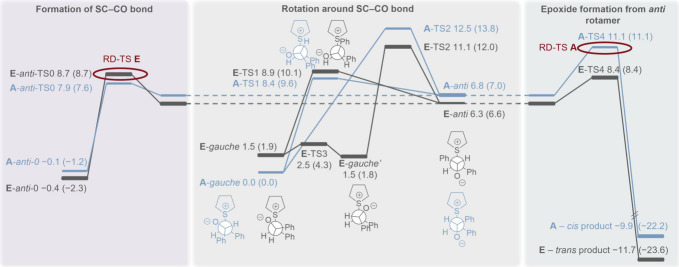
Energy diagram at the B3LYP-D3­(BJ)/def2-TZVPP level of theory for the most stable pathways leading to each of the two products (**A** and **E**) for R^1^ = R^2^ = H, showing the ZPVE-corrected energies in kcal mol^−1^ (and free energies in parentheses).

**Figure 8:**
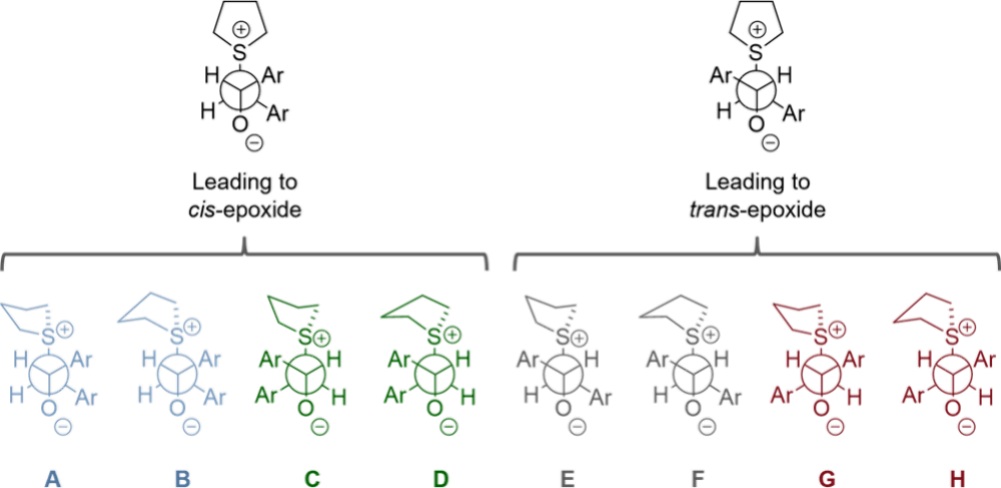
Newman projections of the computed *anti* rotamers at the B3LYP-D3­(BJ)/def2-TZVPP level of theory.

After formation of the *anti* rotamer, the products can form via **TS4** (Figures 6 and 7). If **TS4** is higher in energy than the barrier value for formation of the *anti*-betaine, then the TS for epoxide formation will be the rate-determining TS (RD-TS), and all steps leading to it could be considered in quasi-equilibrium. If the barrier value for the *anti* rotamer formation is higher than **TS4**, then the TS associated with this barrier would be considered the RD-TS, and the formation of the *anti*-betaine could be considered irreversible.

After computing the pathways for **A**-**H** (for the full pathways see the SI), we found that for both derivatives (R^1^ = R^2^ = H and R^1^ = R^2^ = ^*t*^Bu) the path with the lowest RD-TS leading to the *trans* product is **E**, while for the *cis* product the lowest RD-TS is in path **A**. The energy diagrams of pathways **A** and **E** are shown in Figures 7 and 9. Interestingly, for both derivatives, the RD-TS of path **E** is one of the TSs leading to the *anti*-betaine **(E-*anti*-TS0 for** R^1^ = R^2^ = H). However, for path **A** the epoxide formation (**TS4**) involves the RD-TS.

**Figure 9:**
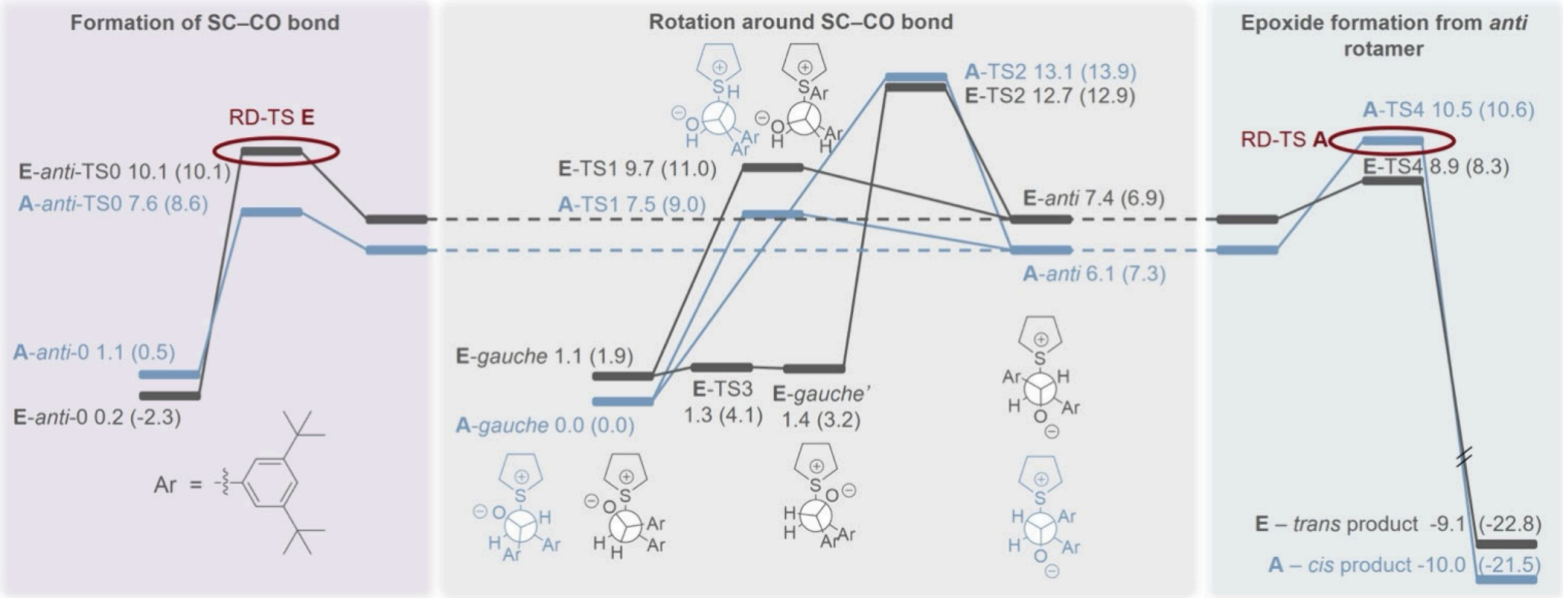
Energy diagram at the B3LYP-D3­(BJ)/def2-TZVPP level of theory for the most stable pathways leading to each of the two products (**A** and **E**) for R^1^ = R^2^ = ^*t*^Bu, showing the ZPVE-corrected energies in kcal mol^−1^ (and free energies in parentheses). Two RD-TSs are shown here for pathway **E**: **E-*anti*-TS0** (RD-TS according to free energies) and **E-TS1** (according to free energies).

For R^1^ = R^2^ = H, the difference in ZPVE-corrected energy between the RD-TSs for paths **A** and **E** is 2.4 kcal mol^–1^ (and the difference in free energy is 2.4 kcal mol^–1^), predicting a preference for the *trans* product, in agreement with the experiment.

For the ^***t***^Bu-substituted reactants (R^1^ = R^2^ = ^*t*^Bu), for **A** the RD-TS is **TS4**, while **TS1** and ***anti*-TS0** are lower in energy and close to each other (Figure 9). For **E** the RD-TS is **TS1** (according to ZPVE-corrected energies, 0.8 kcal mol^–1^ below the **A** RD-TS) or ***anti*-TS0** (according to free energies, 0.5 kcal mol^–1^ below the **A** RD-TS), while **TS4** is lower in energy. These computations identify the ^***t***^Bu-substituted reaction to show preference for the formation of the *trans* product, but this preference is much smaller than for the unsubstituted reaction, matching the experimental results.

Regarding the reversibility of the formation of the two epoxide products, for the *trans*-epoxide (pathway **E)**, **E-*anti*-TS0** and **E-TS1** are somewhat higher in energy than **E-TS4**, meaning that the formation of the *anti*-betaine should be irreversible. For the *cis*-epoxide (pathway **A**), **TS4** is the highest TS (for R^1^ = R^2^ = H and for R^1^ = R^2^ = ^*t*^Bu), and so only the epoxidation step is expected to be irreversible, while the steps leading to the *anti* rotamer are expected to be reversible. This matches the experimental results from previous studies and from the ring closure and crossover experiments shown above. A closer inspection of the RD-TSs can provide insight into the effects that influence their energies. To explore the effects of noncovalent interactions, we performed intramolecular symmetry-adapted perturbation theory (I-SAPT) analysis^75,76^ using the SIAO1 partitioning algorithm.^77^ I-SAPT is a method for decomposition of the intramolecular interaction energies between two parts of the same molecule that are connected by a linker. The interaction can be decomposed into basic contributions: electrostatic, exchange (repulsion), induction, and dispersion. We chose the SIAO1 partitioning method, as it can mitigate the effects of artificial dipole moments (such as an unphysically large electrostatic repulsion), produced by previous partitioning algorithms. The analysis was performed on the interaction between the two aromatic rings (including R groups), while the two sp^3^ carbons, the oxygen and sulfur groups, where defined as the linker. The changes in the orientation of the aromatic rings are shown in Figure 10, and the energies are presented in Figure 11.

**Figure 10:**
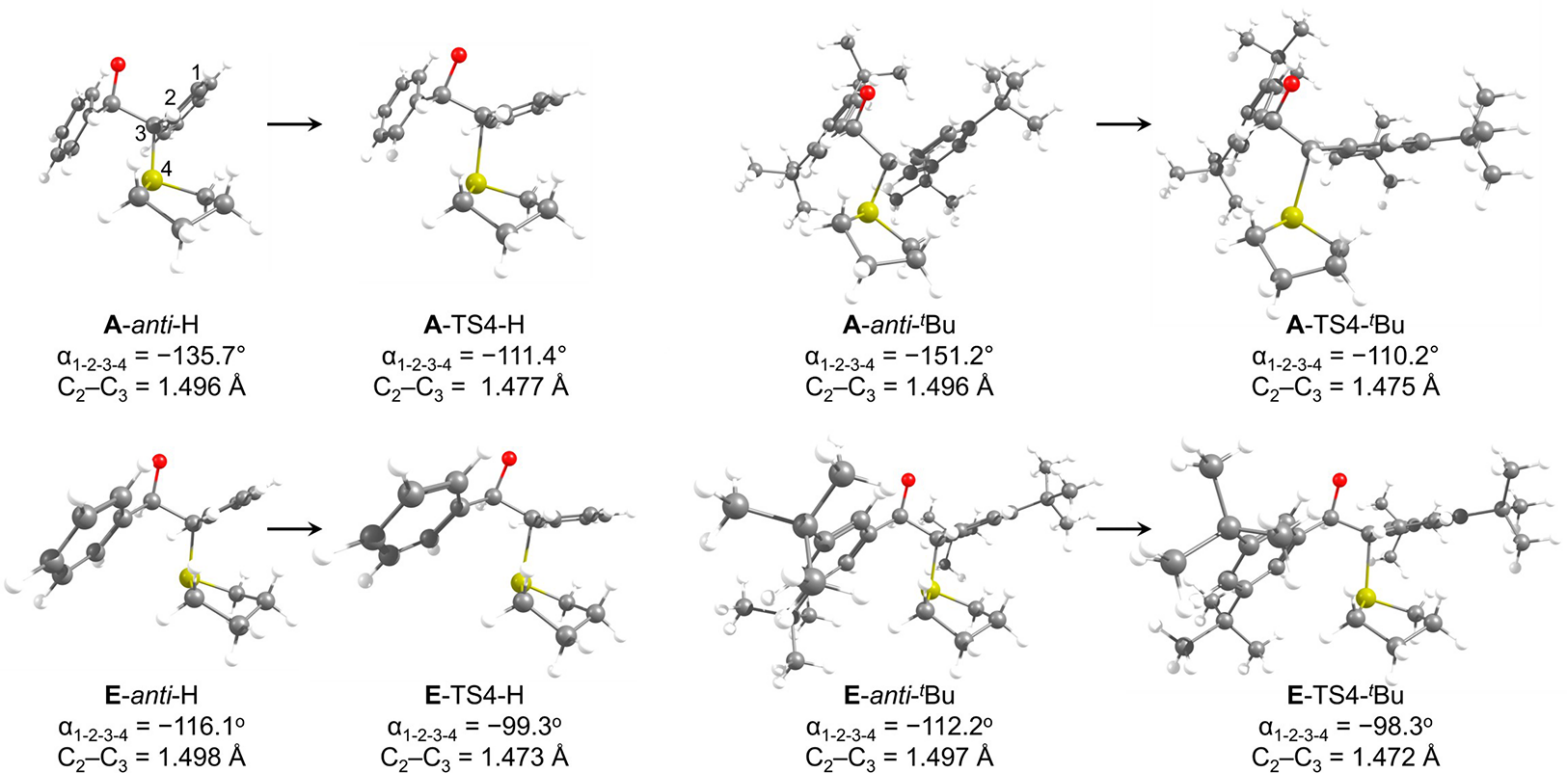
Changes in orientation of the aromatic rings going from the *anti* rotamer to **TS4**. See the diagram at the top left for atom numbering.

**Figure 11:**
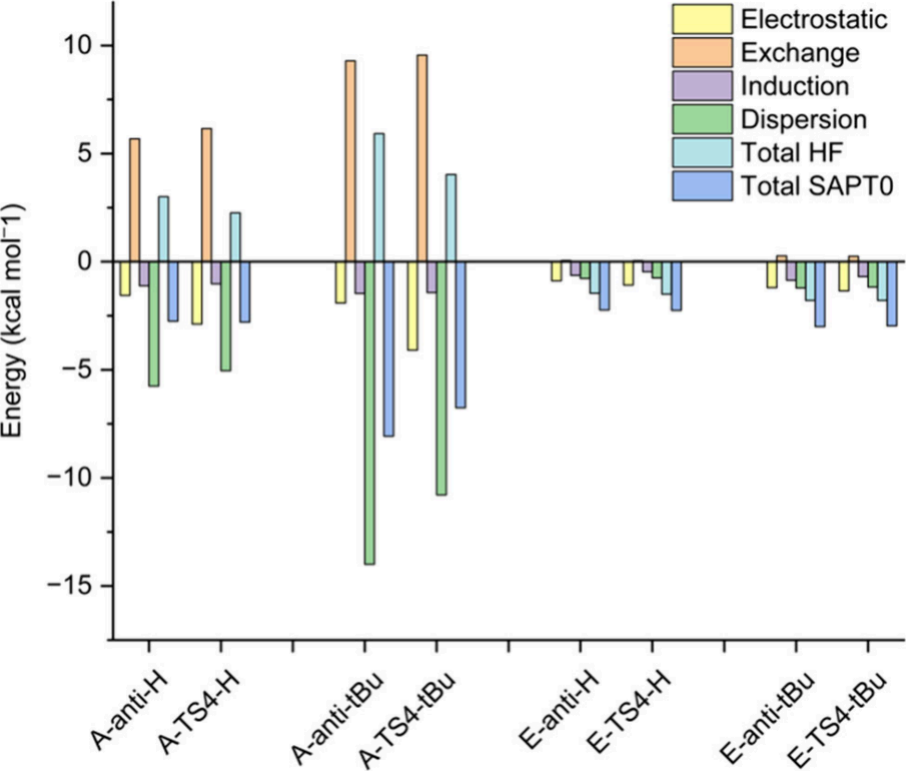
Results of the I-SAPT analysis at the DF-SAPT0 aug-cc-pVTZ level of theory between the two aromatic rings in the *anti*-betaine and in **TS4** of pathways **A** and **E**.

First, looking at the epoxidation TS (Figures 7 and 9), we observe that for both R^1^ = R^2^ = *t*Bu and for R^1^ = R^2^ = H, **TS4** is lower for pathway **E** than for pathway **A**. The structures of **TS4** in Figure 10 show that the aromatic ring on the sulfur-bound carbon rotates during the reaction (the ring becomes more perpendicular to the C–S bond, such that α_1–2–3–4_ becomes closer to −90° upon formation of **TS4**; for more structural data, see the SI). This rotation allows for a better conjugation between the π system and the orbitals that participate in the S_N_2-type reaction (as evidenced by the shortening of the C_2_–C_3_ bond in **TS4**, and from the LUMO and HOMO–4 orbitals in Figure S8). However, this geometric change is expected to weaken LD interactions, as the two aromatic rings are no longer oriented parallel to one another. Comparing all the **E** species in Figure 10 to the analogous **A** species, we can see that α_1–2–3–4_ is closer to −90 ° in all **E** species, which is expected to result in better orbital overlap (and a shorter C_2_–C_3_ bond) and explains the increased stabilization of **TS4** in the **E** pathway. In addition, the change in angle going from **E-*anti*** to **E**-**TS4** is smaller than the change from **A-*anti*** to **A-TS4**, contributing also to the smaller barrier along the **E** pathway. Another interesting point we can learn from the structures shown in Figure 10 is that in **A-TS4-** ^***t***^ **Bu** the R^1^ groups are directed toward the aldehyde aromatic ring in a geometry that facilitates σ–π interactions, while the R^2^ groups point outward relative to the sulfide aromatic ring and are expected to have mainly σ–σ interactions with the R^1^ groups (for the larger R^1^ groups) and no σ–π interactions with the sulfide aromatic ring. As a result, we would expect the R^1^ groups to have a more significant LD-based effect (relative to R^2^) of stabilizing **A-TS4** and increasing the amounts of the *cis*-epoxide product. Further, when R^1^ = H we would expect relatively weak LD interactions between the aromatic groups, even with large R^2^ substituents, close to the interactions for R^1^ = R^2^ = H.

This matches and explains the experimental results in Figure 2, which show indeed that the effect of R^2^ on increasing the amount of *cis* product is smaller than the effect of R^1^, and is negligible when R^1^ = H. In addition, the geometry of **A-TS4** suggests that if there were halogens at the R^2^ position, they would not interact significantly with the smaller R^1^ groups (H and Me) in this TS, and the main interactions in this case would be C–H­(R^1^)···π interactions. Such interactions would be less stabilizing for π-systems with more electron-withdrawing halogens, explaining their higher preference for the *trans* product in Figure 3.

Figure 11 shows the I-SAPT analyses for the *anti*-betaine and **TS4** for pathways **A** and **E**. The total interaction energies between the two aromatic rings in **E-*anti*** and **E**-**TS4** are very small (up to only 3 kcal mol^–1^) both for R^1^ = R^2^ = ^*t*^Bu and for R^1^ = R^2^ = H, and there is no significant difference between the interaction energies in **E-*anti*** and **E**-**TS4**. This is to be expected, as the aromatic groups are far away from one another in these **E** species.

However, going from **E** to **A**, the individual parts of the interaction become much larger, and the most significant contributions are from exchange and dispersion. Again, this is to be expected, as the aromatic groups are close to one another and have no significant electron donating/withdrawing groups. For all **A** species shown on the graph, except for **A**-**TS4**-H, the dispersion stabilization is larger than the exchange destabilization, and this difference is especially large for **A-*anti*-** ^***t***^ **Bu** (more than 4 kcal mol^–1^). Comparing **A-*anti*** with **A**-**TS4**, for both R groups the dispersion contribution is more stabilizing in **A-*anti*** than in **TS4**. The exchange repulsion increases somewhat when going from **A-*anti*** to **A**-**TS4**.

All of these observations regarding **A**, support the suggestion presented above, that the rotation of one aromatic ring in **A**-**TS4** disrupts some of the dispersion attraction between the rings. This results in a decrease in total interaction energy between the rings when going from **A-*anti*-** ^***t***^ **Bu** to **A**-**TS4-** ^***t***^ **Bu**. For **A-*anti*-H** and **A-TS4-H** the total interaction energy is almost the same, because of the increase in electrostatic interaction in **TS4**, possibly from Ar–H···π interactions. As a result of the decrease in total interaction energy for **A-TS4-** ^***t***^ **Bu** but not for **A-TS4-H**, we would expect the energy barrier for the epoxidation step to be larger for R^1^ = R^2^ = ^*t*^Bu than for R^1^ = R^2^ = H. This is indeed the case, but the difference is very small (4.40 for ^***t***^Bu, 4.29 for H).

In general, the total interaction energies in **A-*anti*-H**, **A-TS4-H**, **E-*anti*-H**, **E-TS4-H**, **E-*anti*-** ^***t***^ **Bu**, and **E-TS4-** ^***t***^ **Bu** are very similar and small (all between 2 and 3 kcal mol^–1^). For **A-*anti*-** ^***t***^ **Bu** and **A-TS4-** ^***t***^ **Bu**, the total interaction energies become larger (more stabilizing), because of LD from the bulky groups. So, when comparing the epoxidation-step PESs of pathways **A** and **E**, even though **E-TS4** is lower in energy than **A-TS4**, we expect the energy difference between **A-TS4-** ^***t***^ **Bu** and **E-TS4-** ^***t***^ **Bu** to be smaller than that between **A-TS4-H** and **E-TS4-H**. This can be observed in Figures 7 and 9, along with the fact that **A-*anti*-H** is higher in energy than **E-*anti*-H** while **A-*anti*-** ^***t***^ **Bu** is more stable than **E-*anti*-** ^***t***^ **Bu** due to these interactions. Note, however, that there are additional interactions that can affect the energies, such as LD interactions between the aromatic rings (especially the aldehyde aromatic ring) and the sulfonium five-membered ring (see Figure S9).

Regarding the two mechanisms for formation of the *anti* betaine, either directly from the complex via **TS0** or from the gauche rotamer via **TS1**, both of these TSs, **TS0** and **TS1**, are lower in energy for pathway **A** relative to **E** (for both R^1^ = R^2^ = H and R^1^ = R^2^ = ^*t*^Bu), even though the aromatic rings are closer to one another for both TSs on **A**. But the difference in energy between the pathways is significantly larger for R^1^ = R^2^ = ^*t*^Bu, indicating that also here the **A** PES is lowered in energy relative to the **E** PES when going to the bulkier R groups. This is again a result of LD interactions, as evidenced by I-SAPT analysis (Figures 12 and 13).

**Figure 12:**
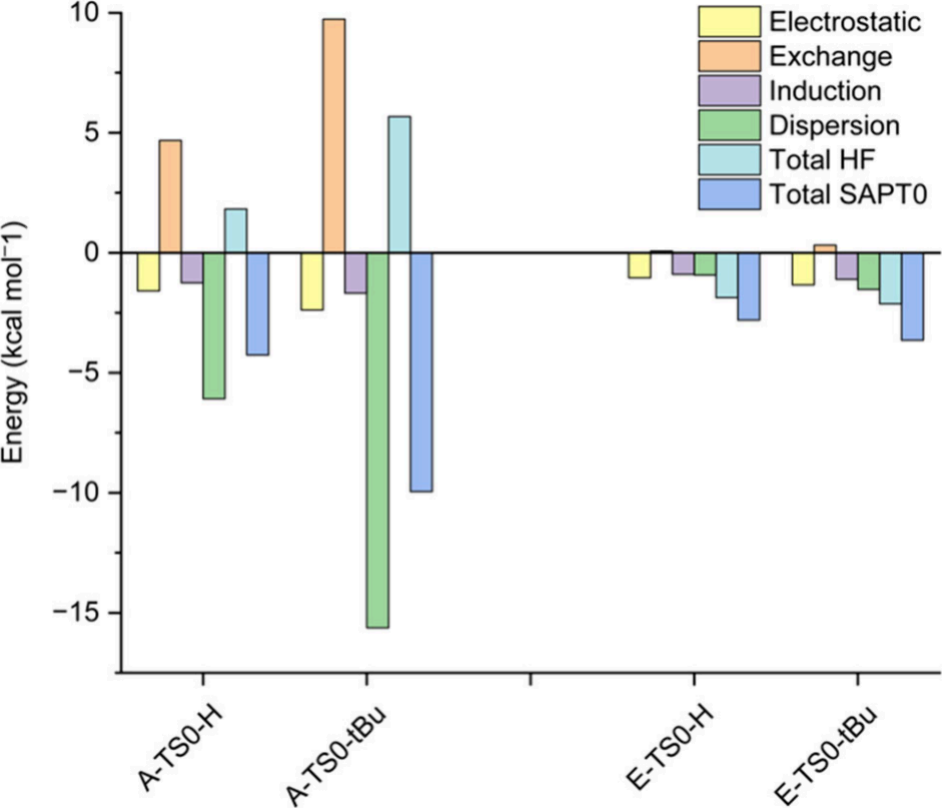
Results of the I-SAPT analysis at the DF-SAPT0 aug-cc-pVTZ level of theory between the two aromatic rings in **TS0** of pathways **A** and **E**.

**Figure 13:**
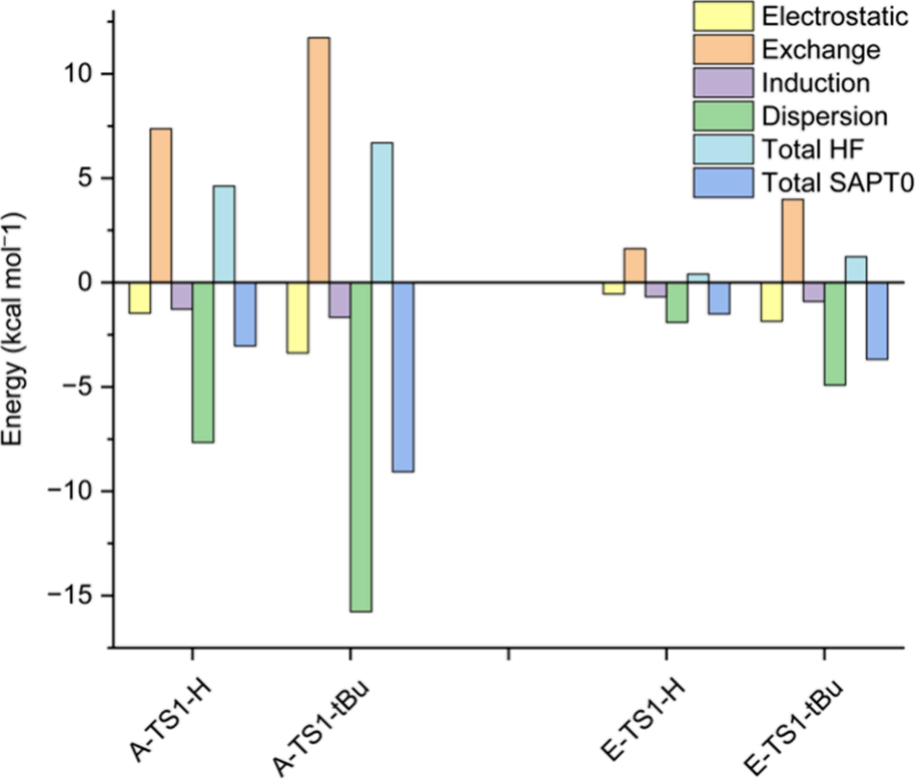
Results of the I-SAPT analysis at the DF-SAPT0 aug-cc-pVTZ level of theory between the two aromatic rings in **TS1** of pathways **A** and **E**.

It can be seen that for **E**-**TS0** (Figure 12) and **E**-**TS1** (Figure 13), the interactions between the two rings are weak for both R groups. The total interactions in **A**-**TS0-H** and **A**-**TS1-H** are also weak and close in magnitude to those in the **E** species. The total interaction in **A**-**TS0-H** and **A**-**TS1-H** is small even though the LD stabilization is larger than in **E**, because the repulsion is also larger. However, in **A-TS0-** ^***t***^ **Bu** and **A-TS1-** ^***t***^ **Bu** LD is more significant, and the exchange part is smaller than the LD part, causing the total interaction energy to be more stabilizing, and thus lowering the energies of **A-TS0-** ^***t***^ **Bu** and **A-TS1-** ^***t***^ **Bu** relative to the energies of **E-TS0-** ^***t***^ **Bu** and **E-TS1-** ^***t***^ **Bu**, more than for R^1^ = R^2^ = H. This matches the energy profiles in Figures 7 and 9. Note that in all the species shown in Figure 12 and 13, the LD stabilization outweighs the exchange destabilization, indicating that we are at the attractive region of the van der Waals potential. In this region, larger groups are expected to be mainly a source of stabilization via LD and not primarily a source of steric repulsion.

## Conclusions

This study underscores the pivotal role of London dispersion (LD) interactions in influencing the *cis*/*trans* diastereoselectivity of the Johnson–Corey–Chaykovsky (JCC) epoxidation. While traditional views attribute diastereoselectivity to steric repulsion, our findings reveal that LD interactions preferentially stabilize transition states, favoring the *cis* product in the presence of bulky (highy polarizable) dispersion energy donors (DEDs).

Through NMR spectroscopy, we demonstrate that larger DEDs stabilize crowded transition states with little steric penalties, with *tert*-butyl substituents showing the strongest shift toward the *cis*-products with Δ*G*_^*t*^Bu^*t*^Bu‑HH_^⧧^ ≈ −0.9 ± 0.1 kcal mol^–1^ for the reaction of **1-** ^***t***^ **Bu** with **2-** ^***t***^ **Bu**. In addition, we confirmed the potential of halogens to act as DEDs when interacting with alkyl groups. Crossover and ring-closure experiments confirm the reversible formation of *syn*-betaine intermediates, further highlighting the contribution of noncovalent interactions to the reaction pathway.

Computational studies using dispersion-corrected DFT provided deeper insights into the molecular interactions driving these trends. The reaction pathway was mapped for various substituents, revealing that LD interactions stabilize crowded betaine and epoxide-forming transition states. I-SAPT analyses demonstrate that with bulky DEDs like *tert*-butyl groups, dispersion outweighs repulsion, resulting in preferential net stabilization.

By demonstrating that LD interactions, rather than steric repulsion, govern transition state stabilizations in the JCC reaction, this work challenges conventional steric models and provides a deeper understanding of the role of noncovalent interactions in stereoselective organic synthesis. Additionally, our study demonstrates, for the first time, that halogen substituents can serve as dispersion energy donors in this reaction context. In contrast to alkyl substituents, halogenation provides a synthetically practical means to modulate stereoselectivity while retaining the potential for further functionalization. This represents a new and potentially useful strategy for reaction design.

## Supplementary Material



## Data Availability

The data underlying this study are available in the published article and its Supporting Information.

## Notes

Experimental details, compound characterization, analytical data, and computational details (PDF)
