# Supplementary material for: London Dispersion Favors Cis Selectivity in the Johnson–Corey–Chaykovsky Epoxidation
Source: J Org Chem. 2026 Mar 26;91(14):4829–41. doi: 10.1021/acs.joc.6c00154 (PMC13077699; doi:10.1021/acs.joc.6c00154)
Supplement: Supplementary file 1 [file jo6c00154_si_001.pdf]

Electronic Supporting Information for

London Dispersion Favors *cis*-Selectivity in the Johnson-Corey-Chaykovsky Epoxidation

Marvin H. J. Domanski,[a] Lars Rummel,[a] Saskia N. Krug,[a] Heike Hausmann,[a] Ephrath Solel*[b] and Peter R. Schreiner*[a]

* Corresponding authors

[a] Institute of Organic Chemistry, Justus Liebig University, Heinrich-Buff-Ring 17, 35392 Giessen, Germany, E-mail: prs@uni-giessen.de

[b] EaStCHEM School of Chemistry, University of Edinburgh, Joseph Black Building, David Brewster Road, Edinburgh EH9 3FJ, United Kingdom, Email: Ephrath.Solel@ed.ac.uk

Contents

Experimental Investigation S5

General Procedure: Corey-Chaykovsky-Epoxidation S5

Computational Evaluation S11

Reaction diagrams for all A-H pathways: S11

TS4 – Noncovalent interactions S18

TS4 – NBO S19

I-SAPT data S20

Synthetic Details S21

General Information S21

Synthetic Procedures S22

3,5-Dimethylbenzaldehyde (2-Me) S22

3,5-Diethylbenzaldehyde (2-Et) S23

3,5-Di-iso-propyl-benzaldehyde (2-iPr) S24

3,5-Di-tert-butyl-benzaldehyde (2-tBu) S25

3,5-Dimethylbenzyl alcohol S26

3,5-Diethylbenzyl alcohol S27

3,5-Di-iso-propylbenzyl alcohol S28

3,5-Di-tert-butylbenzyl alcohol S29

3,5-Dimethylbenzyl bromide...S30

3,5-Diethylbenzyl bromide..S31

3,5-Di-iso-propylbenzyl bromide...S32

3,5-Di-tert-butylbenzyl bromide S33

1-[(Phenyl)methyl] tetrahydrothiophenium tetrafluoroborate (1-H)..........................S34

1-[(3,5-Dimethylphenyl)methyl] tetrahydrothiophenium tetrafluoroborate (1-Me)..S35

1-[(3,5-Diethylphenyl)methyl] tetrahydrothiophenium tetrafluoroborate (1-Et).......S36

1-[(3,5-Di-iso-propyl-phenyl)methyl] tetrahydrothiophenium tetrafluoroborate (1-iPr)S37

1-[(3,5-Di-tert-butyl-phenyl)methyl] tetrahydrothiophenium tetrafluoroborate (1-tBu)S38

Bis-(3,5-di-tert-butyl-phenyl)acetylene...S39

1,1′-(1Z)-1,2-Ethenediylbis[3,5-di-tert-butylbenzene]..S40

1,1′-(1E)-1,2-Ethenediylbis[3,5-di-tert-butylbenzene]..S41

Cis-diphenyloxiran (3-cis-H) S42

Trans-diphenyloxiran (3-trans-H) S43

Cis-2,3-Bis[3,5-di-tert-butylphenyl]oxirane (3-cis-tBu)..S44

Trans-2,3-Bis[3,5-di-tert-butylphenyl]oxirane (3-trans-tBu).....................................S45

Syn-1,2-diphenyl-2-hydroxyethyl(dimethyl) sulfonium iodide (4-syn-H) S46

Anti-1,2-diphenyl-2-hydroxyethyl(dimethyl) sulfonium iodide (4-anti-H)...............S47

Syn-1,2-Bis[3,5-di-tert-butyl]phenyl-2-hydroxyethyl(dimethyl) sulfonium iodide (4-syn-tBu)..S48

Anti-1,2-Bis[3,5-di-tert-butyl]phenyl-2-hydroxyethyl(dimethyl) sulfonium iodide (4-anti-tBu)..S49

2-(3,5-Dimethylphenyl)-3-phenyl oxirane (3-H-Me)..S50

2-(3,5-Di-iso-propylphenyl)-3-phenyl oxirane (3-H-iPr)..S51

2-(3,5-Di-tert-butylphenyl)-3-phenyl oxirane (3-H-tBu) S52
2,3-Di-(3,5-dimethyl)-phenyl oxirane (3-Me-Me) S53
NMR Spectroscopy S54
General Information S54
3,5-Dimethylbenzaldehyde (2-Me) S55
3,5-Diethylbenzaldehyde (2-Et) S56
3,5-Di-iso-propyl-benzaldehyde (2-iPr) S57
3,5-Di-tert-butyl-benzaldehyde (2-tBu) S58
3,5-Dimethylbenzyl alcohol S59
3,5-Diethylbenzyl alcohol S60
3,5-Di-iso-propylbenzyl alcohol S61
3,5-Di-tert-butylbenzyl alcohol S62
3,5-Dimethylbenzyl bromide S63
3,5-Diethylbenzyl bromide S64
3,5-Di-iso-propylbenzyl bromide S65
3,5-Di-tert-butylbenzyl bromide S66
1-[(Phenyl)methyl] tetrahydrothiophenium tetrafluoroborate (1-H) S67
1-[(3,5-Dimethylphenyl)methyl] tetrahydrothiophenium tetrafluoroborate (1-Me) .. S68
1-[(3,5-Diethylphenyl)methyl] tetrahydrothiophenium tetrafluoroborate (1-Et) S69
1-[(3,5-Di-iso-propyl-phenyl)methyl] tetrahydrothiophenium tetrafluoroborate (1-iPr)S70
1-[(3,5-Di-tert-butyl-phenyl)methyl] tetrahydrothiophenium tetrafluoroborate (1-tBu)S71
Bis-(3,5-di-tert-butyl-phenyl)acetylene S72
1,1′-(1Z)-1,2-Ethenediylbis[3,5-di-tert-butylbenzene] S73
1,1′-(1E)-1,2-Ethenediylbis[3,5-di-tert-butylbenzene] S74
Cis-diphenyloxirane (3-cis-H) S75
Trans-diphenyloxirane (3-trans-H) S76
Cis-2,3-Bis[3,5-di-tert-butylphenyl]oxirane (3-cis-tBu) S77
Trans-2,3-Bis[3,5-di-tert-butylphenyl]oxirane (3-trans-tBu) S78

Syn-1,2-diphenyl-2-hydroxyethyl(dimethyl) sulfonium iodide (4-syn-H) S79

Anti-1,2-diphenyl-2-hydroxyethyl(dimethyl) sulfonium iodide (4-anti-H)...............S80

Syn-1,2-Bis[3,5-di-tert-butyl]phenyl-2-hydroxyethyl(dimethyl) sulfonium iodide (4-syn-tBu)...S81

Anti-1,2-Bis[3,5-di-tert-butyl]phenyl-2-hydroxyethyl(dimethyl) sulfonium iodide (4-anti-tBu)...S82

2-(3,5-Dimethylphenyl)-3-phenyl oxirane (3-H-Me)..S83

2-(3,5-Di-iso-propylphenyl)-3-phenyl oxirane (3-H-iPr)...S84

2-(3,5-Di-tert-butylphenyl)-3-phenyl oxirane (3-H-tBu)...S85

2,3-Di-(3,5-dimethyl)-phenyl oxirane (3-Me-Me)..S86

Computational Details...S87

References...S194

Experimental Investigation

General Procedure: Corey-Chaykovsky-Epoxidation

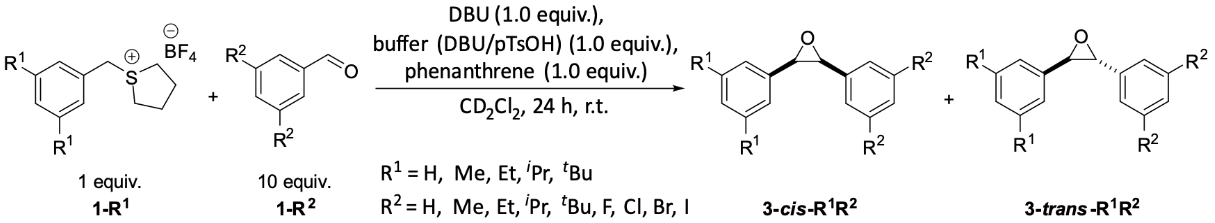

All reactions were carried out in an NMR tube and directly analyzed without further purification. For this purpose, stock solutions for each sulfone (0.05 M in CH_2Cl_2), for each aldehyde (0.5 M in CH_2Cl_2), for the buffer solution (0.05 M DBU/pTsOH*$6H_2O$ in CH_2Cl_2), for DBU (0.05 M in CH_2Cl_2) and phenanthrene (0.05 M in CH_2Cl_2) as internal standard were prepared.

A NMR tube was filled with 0.1 mL of the corresponding sulfone stock solution, followed by the addition of 0.1 mL stock solution phenanthrene, 0.1 mL buffer, 0.1 mL aldehyde and finally 0.1 mL DBU. The Tube was vigorously shook and analyzed after 24 h at room temperature. The product ratios were determined by integration of the proton shifts using a 600 MHz NMR instrument.

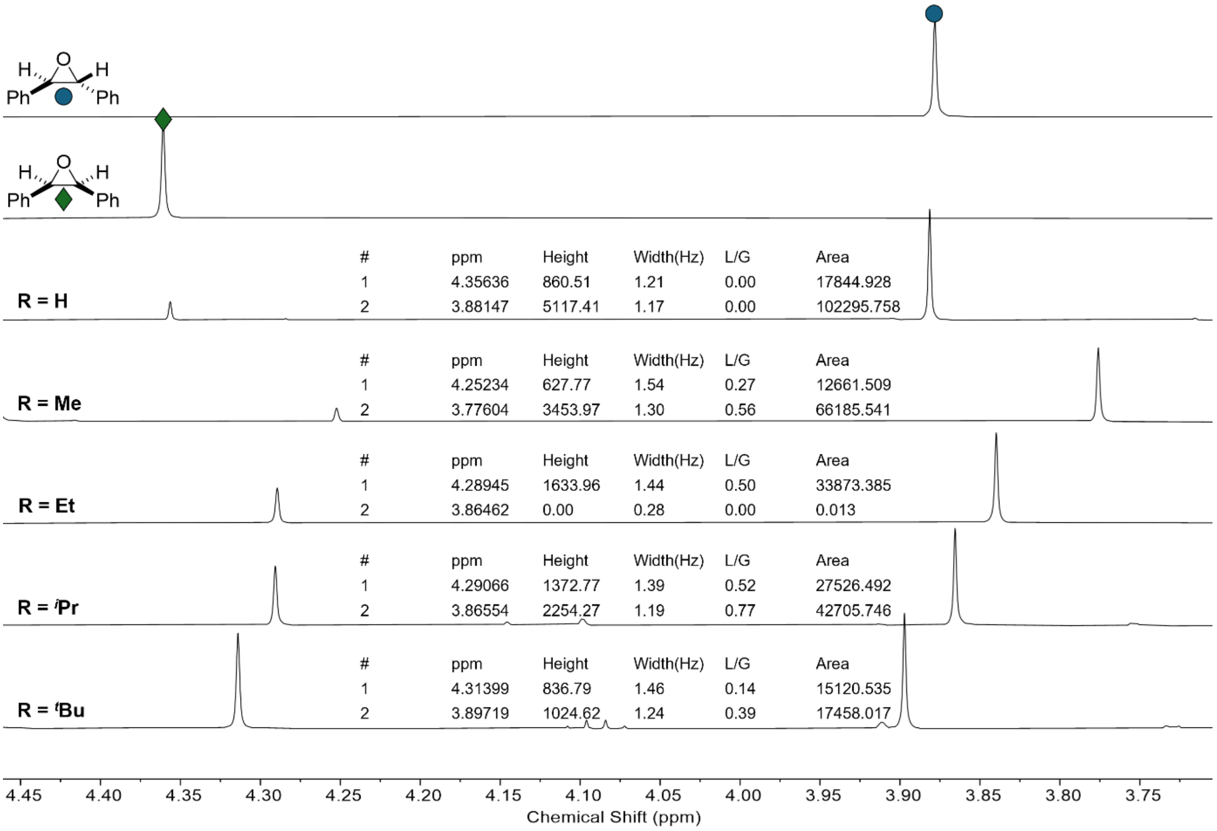

Figure S1: ^{1}H-NMR shifts of the relevant regions depicted for the symmetric alkyl-series and the corresponding peak areas used for further calculations. The top two 1-H NMR's show the recorded reference spectrum used for the assignment.

The product ratios were determined *via* integration of the aliphatic C−H Signals of the spectra. The error of the quantitative NMR analysis was estimated to be 5% with $\Delta T = \pm 0.15$ K.

$\Delta_r G$ was calculated for each temperature according to the following equation:

$$\Delta_r G = -\ln(K)\,RT$$

The total differential was utilized for the error calculation for $\Delta_r G$:

$$\Delta_r G = \left|-\frac{RT}{K_{eq}}\right| \Delta K_{eq} + \left|-\ln K_{eq}\, R\right| \Delta T$$

Table S1: Measured product ratios 3-*cis*-R^1-R^2 and 3-*trans*-R^1-R^2 with the corresponding absolute Gibbs free energy values $\Delta G_{R^1R^2}$ and their errors for the Corey-Chaykovsky-Epoxidation.

Sulfone 1-R^1	Aldehyde 2-R^2	Cis / trans ratio	$\Delta G_{R^1R^2}$ kcal / mol	$\Delta G_{R^1R^2}$-HH kcal / mol	$\Delta\Delta G_{R^1R^2}$ kcal / mol	$\Delta\Delta G_{R^1R^2}$-HH kcal / mol
H	H	0.166	0.975	0.000	0.049	0.099
H	Me	0.209	0.849	-0.125	0.043	0.092
H	Et	0.192	0.895	-0.080	0.045	0.095
H	^{i}Pr	0.202	0.868	-0.107	0.044	0.093
H	^{t}Bu	0.196	0.883	-0.092	0.045	0.094
Me	H	0.249	0.754	-0.220	0.038	0.087
Me	Me	0.289	0.673	-0.301	0.034	0.083
Me	Et	0.274	0.702	-0.272	0.036	0.085
Me	^{i}Pr	0.324	0.611	-0.364	0.031	0.080
Me	^{t}Bu	0.319	0.620	-0.354	0.031	0.081
Et	H	0.307	0.641	-0.334	0.032	0.082
Et	Me	0.367	0.544	-0.431	0.027	0.077
Et	Et	0.427	0.462	-0.512	0.023	0.073
Et	^{i}Pr	0.477	0.401	-0.573	0.020	0.070
Et	^{t}Bu	0.482	0.396	-0.578	0.020	0.069

^{i}Pr	H	0.392	0.509	-0.466	0.026	0.075
^{i}Pr	Me	0.512	0.363	-0.612	0.018	0.068
^{i}Pr	Et	0.584	0.292	-0.683	0.015	0.064
^{i}Pr	^{i}Pr	0.658	0.227	-0.747	0.011	0.061
^{i}Pr	^{t}Bu	0.665	0.221	-0.753	0.011	0.060
^{t}Bu	H	0.458	0.424	-0.551	0.021	0.071
^{t}Bu	Me	0.722	0.177	-0.798	0.009	0.058
^{t}Bu	Et	0.691	0.201	-0.774	0.010	0.059
^{t}Bu	^{i}Pr	0.802	0.120	-0.855	0.006	0.055
^{t}Bu	^{t}Bu	0.928	0.040	-0.934	0.002	0.051
H	F	0.070	1.441	0.466	0.073	0.122
H	Cl	0.073	1.421	0.446	0.072	0.121
H	Br	0.105	1.222	0.247	0.062	0.111
H	I	0.098	1.261	0.286	0.064	0.113
Me	F	0.070	1.440	0.466	0.073	0.122
Me	Cl	0.130	1.105	0.130	0.056	0.105
Me	Br	0.142	1.058	0.083	0.053	0.103
Me	I	0.147	1.039	0.064	0.053	0.102
Et	F	0.130	1.106	0.131	0.056	0.105
Et	Cl	0.231	0.796	-0.179	0.040	0.090
Et	Br	0.243	0.768	-0.207	0.039	0.088
^{i}Pr	I	0.233	0.789	-0.185	0.040	0.089
^{i}Pr	F	0.183	0.921	-0.053	0.047	0.096
^{i}Pr	Cl	0.397	0.501	-0.473	0.025	0.075
^{i}Pr	Br	0.399	0.498	-0.477	0.025	0.074

^{i}Pr	I	0.452	0.431	-0.544	0.022	0.071
^{t}Bu	F	0.347	0.575	-0.400	0.029	0.078
^{t}Bu	Cl	0.532	0.342	-0.632	0.017	0.067
^{t}Bu	Br	0.547	0.328	-0.647	0.017	0.066
^{t}Bu	I	0.541	0.334	-0.641	0.017	0.066

Additionally, we monitored the reaction progress over time for the symmetric Methyl and ^{i}Pr-substituted System. To lower the reaction rate, we carried out the reaction at 0 °C with THB as a base and observed the reactions progress over four h. Only one equivalent of aldehyde has been used to better track the decay of the aldehyde. Over the period of the reaction, regardless of the substituent, no change in the product distribution was observed.

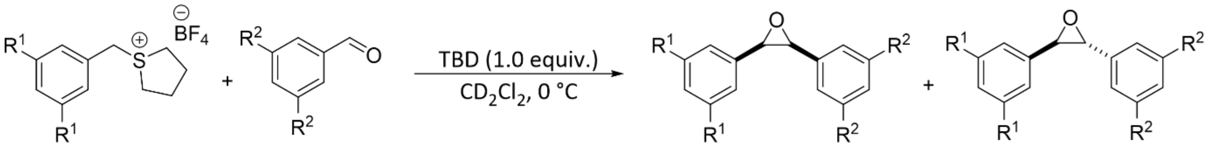

1 equiv. 1 equiv.

1-R¹ **2-R²** **3-*cis*-R¹-R²** **3-*trans*-R¹-R²**

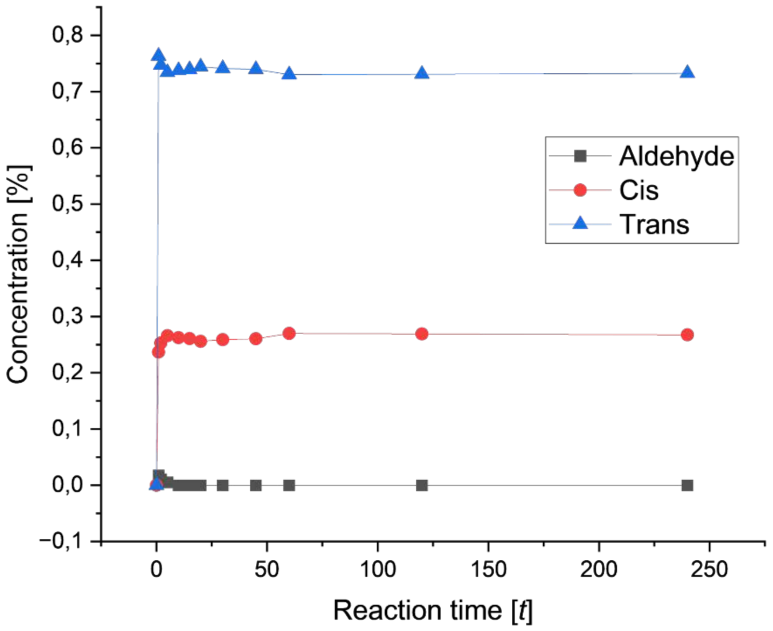

Figure S2: Measured reaction progress in minutes for the symmetric R1=R^2=H system over a period of 4 h.

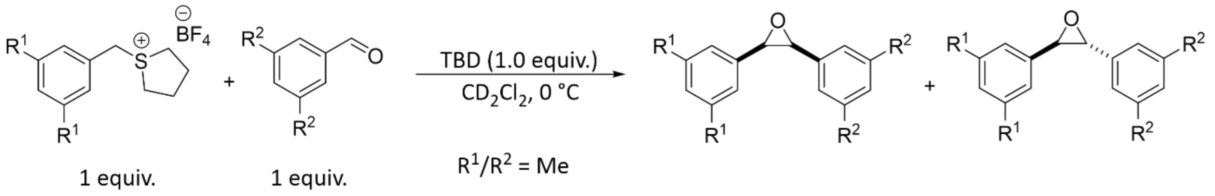

1-R[1] **2-R**[2] **3-*cis*-R**[1]**-R**[2] **3-*trans*-R**[1]**-R**[2]

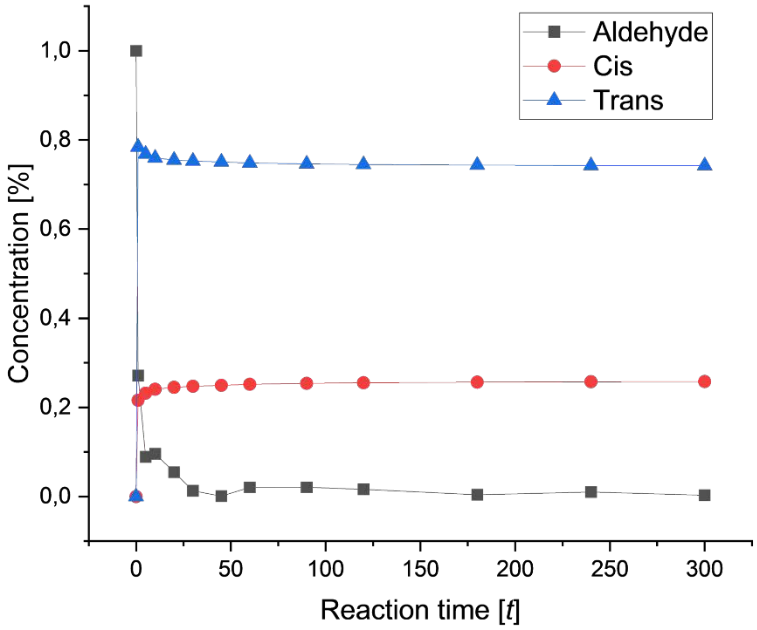

Figure S3: Measured reaction progress in minutes for the symmetric R^1=R^2=Me system over a period of 5 h.

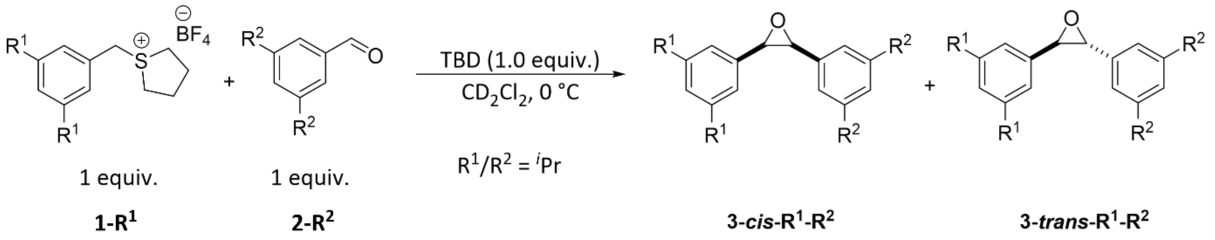

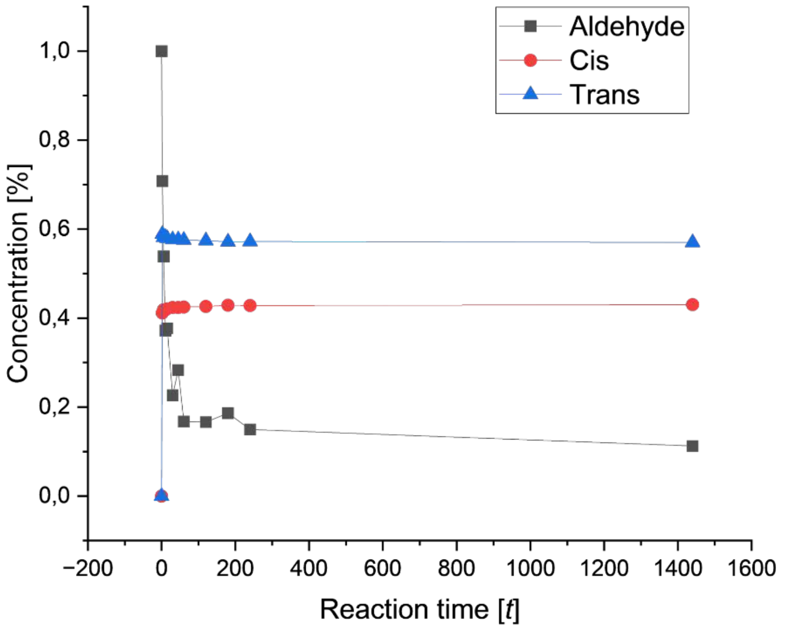

Figure S4: Measured reaction progress in minutes for the symmetric R^1=R^2=^{i}Pr system over a period of 24 h.

Computational Evaluation

CREST[1,2] version 2.1 was used in order to find various conformers for the betaine intermediates, for unsubstituted Ph (R^1=R^2=H). The conformers for R^1=R^2=^{t}Bu were generated based on the (DFT optimized) unsubstituted Ph conformers. All molecular geometries were optimized at the B3LYP[3,4]/def2-TZVPP[5] level of theory, with Grimme's D3(BJ) dispersion correction.[6,7] To match the experimental conditions we used a temperature of 298.15 K in our computations, with dichloromethane (DCM) as the implicit solvent with the Solvation Model based on Density (SMD[8]). All reported energies are ZPVE-corrected, unless stated otherwise. all structures were characterized with analytical Hessian calculations to ensure real minima (Nimag=0) or transition structures (Nimag=1) and for obtaining thermochemical data. For all DFT computations we used the Gaussian16 revision C.01 program package.[9] NCIPlots[10,11] were generated with Multiwfn 3.7,[12] and visualized using VMD.[13] NBO analyses were performed with NBO 3.1[14] as implemented in Gaussian16. I-SAPT[15,16] analyses with the SIAO1 partitioning algorithm[17] was performed with the PSI4[18] software version 1.3.2, using DF-SAPT0 with the aug-cc-pVTZ basis set for all atoms.

Reaction diagrams for all A-H pathways:

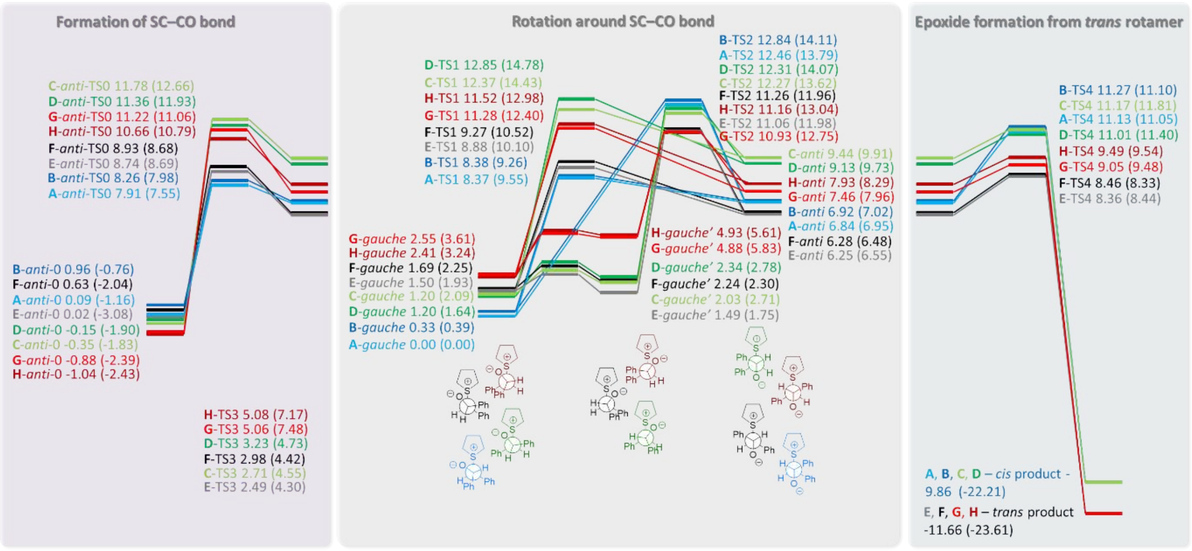

Figure S5: An energy diagram for pathways **A-H** for R^1=R^2=H, showing the ZPVE-corrected energies in kcal mol^{-1} computed at the at the B3LYP-D3(BJ)/def2-TZVPP level of theory (free energies in parenthesis).

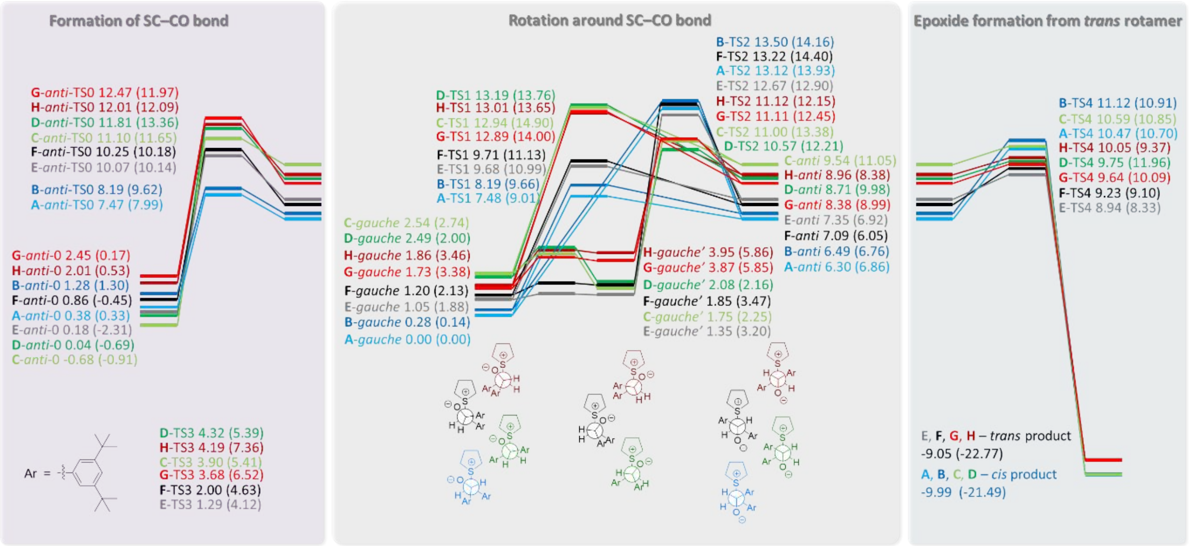

Figure S6: An energy diagram for pathways **A**-**H** for $R^1=R^2={}^tBu$, showing the ZPVE-corrected energies in kcal mol^{-1} computed at the at the B3LYP-D3(BJ)/def2-TZVPP level of theory (free energies in parenthesis).

Table S2: Relative ZPVE-corrected energies in kcal mol^{-1} (and free energies in parenthesis) for the various species of pathways **A**-**H** for $R^1=R^2=H$. The reference zero energy is taken as the most stable *gauche* betaine (**A-*gauche***).

	A	B	C	D	E	F	G	H
***gauche*-0-H**	0.36 (-2.07)	0.71 (-3.44)	0.65 (-1.84)	0.23 (-1.88)	-0.36 (-2.30)	0.00 (-2.11)	-0.26 (-2.33)	-0.29 (-1.95)
***gauche*-TS0-H**	6.57 (6.43)	6.26 (5.77)	8.77 (8.94)	7.67 (8.25)	6.36 (6.34)	5.94 (6.14)	8.20 (8.41)	7.36 (7.88)
***gauche*-H**	0.00 (0.00)	0.33 (0.39)	1.20 (2.09)	1.20 (1.64)	1.50 (1.93)	1.69 (2.25)	2.55 (3.61)	2.41 (3.24)
***gauche'*-0-H**	-	-	0.04 (-2.32)	0.25 (-1.97)	0.09 (-0.99)	0.97 (-0.91)	-0.87 (-2.41)	-0.76 (-2.17)
***gauche'*-TS0-H**	-	-	8.81 (9.08)	8.31 (8.76)	6.50 (6.52)	7.06 (6.71)	10.25 (10.27)	9.76 (10.22)
***gauche'*-H**	-	-	2.03 (2.71)	2.34 (2.78)	1.49 (1.75)	2.24 (2.30)	4.88 (5.83)	4.93 (5.61)
***anti*-0-H**	0.09 (-1.16)	0.96 (-0.76)	-0.35 (-1.83)	-0.15 (-1.90)	0.02 (-3.08)	0.63 (-2.04)	-0.88 (-2.39)	-1.04 (-2.43)
***anti*-TS0-H**	7.91 (7.55)	8.26 (7.98)	11.78 (12.66)	11.36 (11.93)	8.74 (8.69)	8.93 (8.68)	11.22 (11.06)	10.66 (10.79)
***anti*-H**	6.84 (6.95)	6.92 (7.02)	9.44 (9.91)	9.13 (9.73)	6.25 (6.55)	6.28 (6.48)	7.46 (7.96)	7.93 (8.29)
**TS1-H**	8.37 (9.55)	8.38 (9.26)	12.37 (14.43)	12.85 (14.78)	8.88 (10.10)	9.27 (10.52)	11.28 (12.40)	11.52 (12.98)
**TS3-H**	-	-	2.71 (4.55)	3.23 (4.73)	2.49 (4.30)	2.98 (4.42)	5.06 (7.48)	5.08 (7.17)
**TS2-H**	12.46 (13.79)	12.84 (14.11)	12.27 (13.62)	12.31 (14.07)	11.06 (11.98)	11.26 (11.96)	10.93 (12.75)	11.16 (13.04)
**TS4-H**	11.13 (11.05)	11.27 (11.10)	11.17 (11.81)	11.01 (11.40)	8.36 (8.44)	8.46 (8.33)	9.05 (9.48)	9.49 (9.54)

**TS between A-anti and D-anti**	10.91 (12.21)	-	-	-	-	-	-	-

Table S3: Relative ZPVE-corrected energies in kcal mol^{-1} (and free energies in parenthesis) for the various species of pathways **A**-**H** for $R^1=R^2={}^tBu$. The reference zero energy is taken as the most stable *gauche* betaine (**A-*gauche***).

	A	B	C	D	E	F	G	H
***gauche*-0-^{t}Bu**	-0.25 (-2.62)	0.12 (-1.74)	-0.42 (-1.01)	1.38 (-0.97)	0.47 (-2.10)	1.54 (0.95)	-1.00 (-0.98)	-1.27 (-1.58)
***gauche*-TS0-^{t}Bu**	7.54 (8.12)	7.26 (7.55)	10.11 (10.18)	9.44 (9.87)	7.19 (8.11)	6.42 (6.62)	8.97 (10.96)	8.53 (10.53)
***gauche*-^{t}Bu**	0.00 (0.00)	0.28 (0.14)	2.54 (2.74)	2.49 (2.00)	1.05 (1.88)	1.20 (2.13)	1.73 (3.38)	1.86 (3.46)
***gauche*'-0-^{t}Bu**	-	-	-0.38 (-1.01)	-0.22 (-0.55)	1.49 (0.47)	1.62 (0.64)	-0.96 (-1.03)	-1.55 (-1.89)
***gauche*'-TS0-^{t}Bu**	-	-	8.63 (10.28)	9.04 (9.00)	6.41 (7.75)	6.86 (8.02)	8.96 (10.98)	8.70 (10.87)
***gauche*'-^{t}Bu**	-	-	1.75 (2.25)	2.08 (2.16)	1.35 (3.20)	1.85 (3.47)	3.87 (5.85)	3.95 (5.86)
***anti*-0-^{t}Bu**	0.38 (0.33)	1.28 (1.30)	-0.68 (-0.91)	0.04 (-0.69)	0.18 (-2.31)	0.86 (-0.45)	2.45 (0.17)	2.01 (0.53)
***anti*-TS0-^{t}Bu**	7.47 (7.99)	8.19 (9.62)	11.10 (11.65)	11.81 (13.36)	10.07 (10.14)	10.25 (10.18)	12.47 (11.97)	12.01 (12.09)
***anti*-^{t}Bu**	6.30 (6.86)	6.49 (6.76)	9.54 (11.05)	8.71 (9.98)	7.35 (6.92)	7.09 (6.05)	8.38 (8.99)	8.96 (8.38)
**TS1-^{t}Bu**	7.48 (9.01)	8.19 (9.66)	12.94 (14.90)	13.19 (13.76)	9.68 (10.99)	9.71 (11.13)	12.89 (14.00)	13.01 (13.65)
**TS3-^{t}Bu**	-	-	3.90 (5.41)	4.32 (5.39)	1.29 (4.12)	2.00 (4.63)	3.68 (6.52)	4.19 (7.36)
**TS2-^{t}Bu**	13.12 (13.93)	13.50 (14.16)	11.00 (13.38)	10.57 (12.21)	12.67 (12.90)	13.22 (14.40)	11.11 (12.45)	11.12 (12.15)
**TS4-^{t}Bu**	10.47 (10.70)	11.12 (10.91)	10.59 (10.85)	9.75 (11.96)	8.94 (8.33)	9.23 (9.10)	9.64 (10.09)	10.05 (9.37)
**TS between A-anti and D-anti**	10.64 (12.23)	-	-	-	-	-	-	-

It should be noted that **TS0**, **TS1** and **TS4** of pathway **E** (for both $R^1=R^2=H$ and $R^1=R^2={}^tBu$) are lower in energy than the TSs of the other *trans*-epoxide forming pathways (**F**, **G**, **H)**, such that conformational changes that can switch between **E** and these other pathways is not energetically advantageous (*i.e* will not form a path with a lower RD-TS). However, for the *cis*-epoxide forming pathways the situation is more complicated. While for pathway **A**, **TS0** and **TS1** are lower than in pathways **B**-**D**, **TS4** is lower for pathway **D**. As **TS4** is the RD-TS of pathway **A**, conversion from **A**-*anti* to **D**-*anti* by movement of the five-membered ring could possibly lead to a lower total RD-TS. To examine this, we computed the barrier for this conversion, and found the TS between **A**-*anti* and **D**-*anti* to have an energy of 10.91 (12.21) kcal mol^{-1} for $R^1=R^2=H$, and 10.64 (12.23) kcal mol^{-1} for $R^1=R^2={}^tBu$. Thus, for $R^1=R^2=H$ this TS is lower than **A-TS4**-H (considering the ZPVE-corrected energy, but not the free energy), and could only slightly lower the total ZPVE-corrected total barrier for the *cis* product of $R^1=R^2=H$ from 11.13 (**A-TS4**-H) in pathway **A** to 11.01 (**D-TS4**-H) in pathway **D**. However, for $R^1=R^2={}^tBu$ the TS for conversion between **A**-*anti* to **D**-*anti* is higher in energy than **A-TS4**-${}^tBu$, so this conversion will not lower the RD-TS.

As written in the main text, the structure of **TS4** shows that the aromatic ring on the sulfur-bound carbon rotates during the epoxide-forming step (see Fig. S7), such that the ring becomes more perpendicular to the C–S bond. In addition to $\alpha_{1\text{-}2\text{-}3\text{-}4}$ becoming closer to -90° upon formation of TS4, $\alpha_{1\text{-}2\text{-}3\text{-}5}$ becomes closer to zero and $\alpha_{1\text{-}2\text{-}3\text{-}6}$ becomes closer to 180°). This rotation allows for a better conjugation between the π system and the orbitals that participate in the S_N2-type reaction.

In **A**-*anti* (for both R groups) the two aromatic rings are on the same side of the O-C-C-S plane, and they can form noncovalent interaction (π-π stacking, LD interactions) which will favor an almost-parallel relative orientation between them. As a result, rotating one of the aromatic rings towards **A-TS4** results in loss of the interactions between the rings and can also cause a clash between them. This hinders the rotation and results in worse orbital overlap in **A-TS4** relative to **E-TS4**, in which there is no such hindering, as the aromatic rings are further apart.

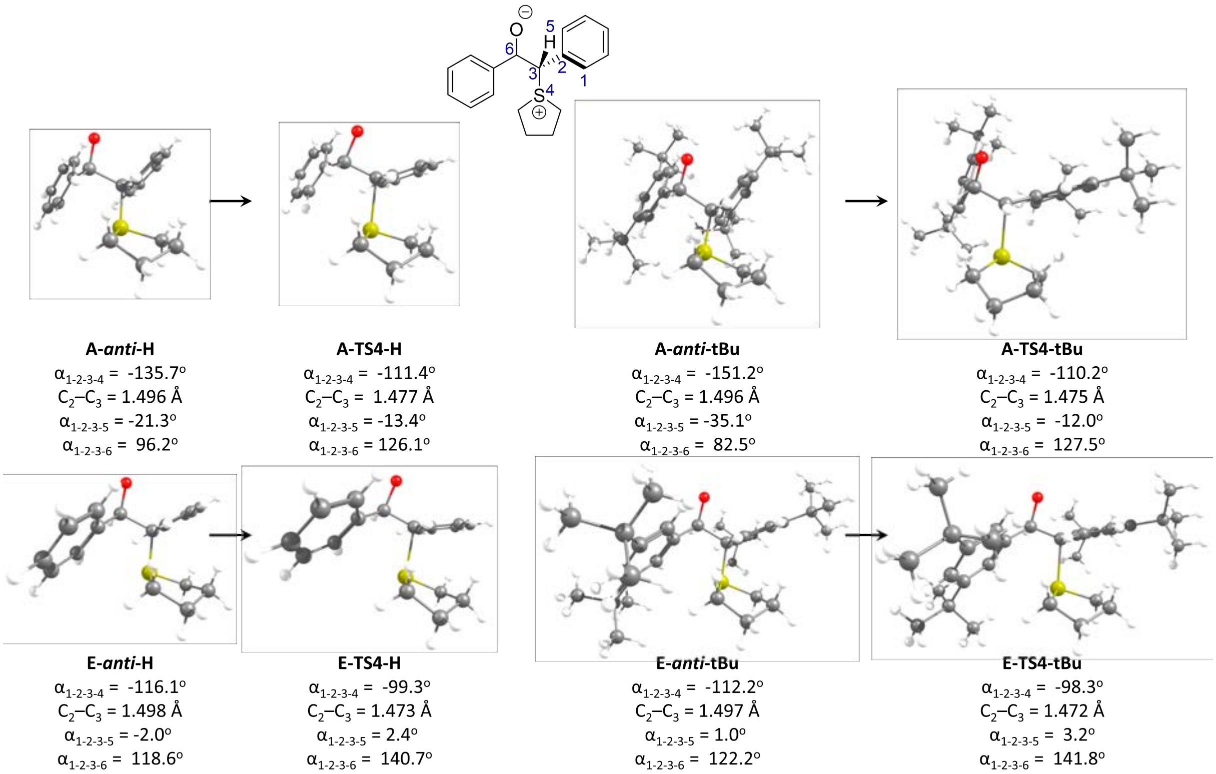

Figure S7: Changes in orientation of the aromatic rings going from the *anti*-rotamer to **TS4**.

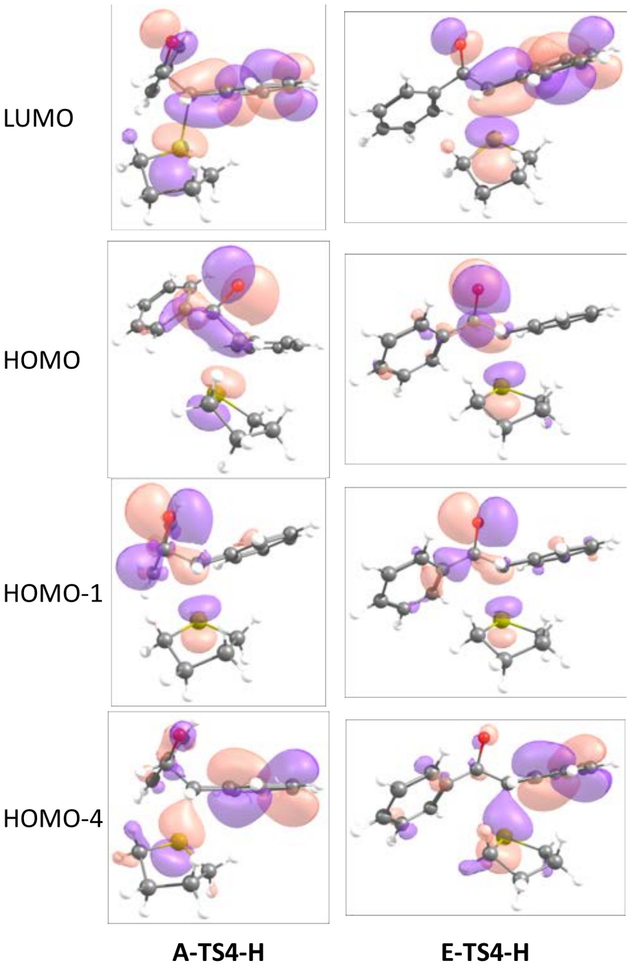

Figure S8: Selected molecular orbitals for **A-TS4-H** and **E-TS4-H**.

TS4 – Noncovalent interactions

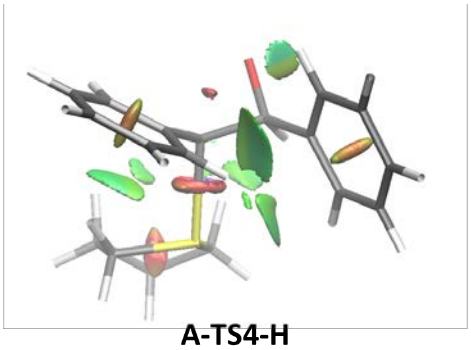

A-TS4-H

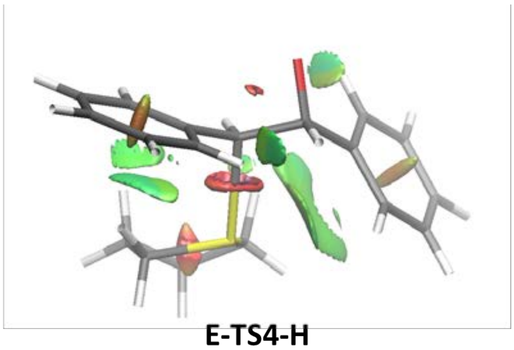

E-TS4-H

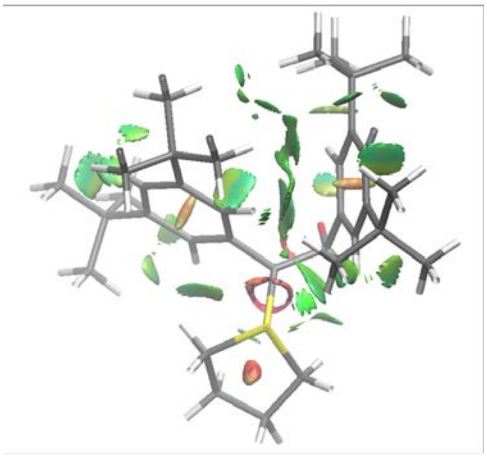

A-TS4-tBu

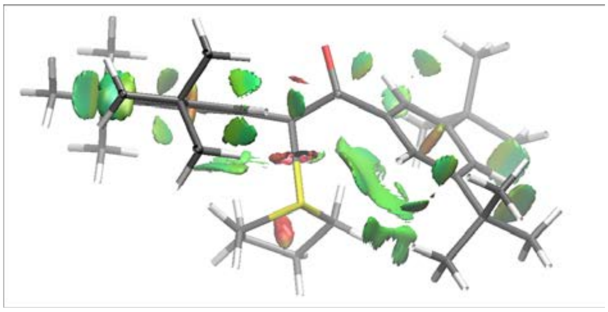

E-TS4-tBu

Figure S9: Noncovalent interaction plots (NCIplots) of the different **TS4**s.

Comparing the structures of **A-TS4** for R^1=R^2=*t*Bu and for R^1=R^2=H (fig. S10), we can see that there is a good agreement between the two structures (dihedral angles α for R^1=R^2=*t*Bu and for R^1=R^2=H). This suggests that the tBu groups, although they form stronger interactions between the rings, do not cause enough repulsion/attraction to greatly increase/reduce the distances between the aromatic rings.

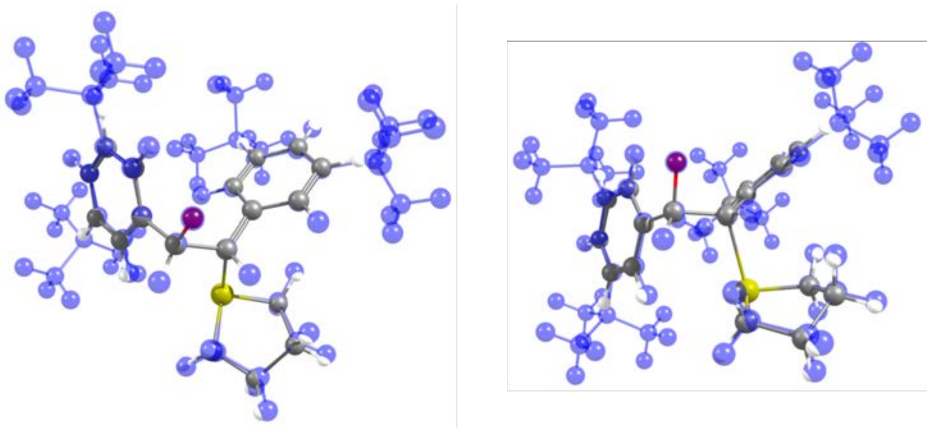

Figure S10: An overlay of **A-TS4-H** (in colors: gray – Carbon, white – Hydrogen, red – Oxygen, yellow – Sulfur) and **A-TS4-tBu** (in blue), at two different orientations.

TS4 – NBO

Checking the NBO charges of the aromatic rings and the C-S carbon (Table S4), the easier rotation and expected better orbital overlap (as seen from the dihedral angles in fig. S7) in **E-TS4** as compared to **A-TS4**, can be the reason for the slightly less positive charge on the C-S carbon for **E-TS4**. This has a small effect on the charges of the neighboring aromatic ring. However, this could also be connected to the fact that **E-TS4** is an earlier TS (closer to **E-*anti*** relative to **A-TS4**, **A-*anti***) and so the charges are more concentrated on the sulfonium ring and on the oxygen.

Table S4: Table of the NBO charges on each aromatic ring (including substituents), on the C-S carbon, on the sulfonium ring and on the H-C-O group. All charges are with hydrogen charges summed into the carbons' charges.

	**Ar(-C-O)**	**Ar(-C-S)**	**C(-S)**	**S ring**	**H-C-O**
**A-TS4-H**	-0.06	-0.01	0.20	0.47	-0.60
**A-TS4-tBu**	-0.06	-0.01	0.20	0.48	-0.61
**E-TS4-H**	-0.06	0.00	0.18	0.51	-0.62
**E-TS4-tBu**	-0.06	0.00	0.18	0.51	-0.63

I-SAPT data

Table S5: Results of the **I**-SAPT analysis between the two aromatic rings in the *anti* betaine and in **TS4, TS0** and **TS1** of pathways **A** and **E**.

	Electrostatic	Echange	Induction	Dispersion	E_{Int}
**A-*anti*-H**	-1.56	5.68	-1.12	-5.75	-2.75
**A-TS4-H**	-2.88	6.16	-1.03	-5.04	-2.79
**A-*anti*-tBu**	-1.90	9.29	-1.46	-13.99	-8.07
**A-TS4-tBu**	-4.10	9.55	-1.42	-10.79	-6.75
**E-*anti*-H**	-0.88	0.06	-0.63	-0.78	-2.23
**E-TS4-H**	-1.08	0.05	-0.47	-0.75	-2.25
**E-*anti*-tBu**	-1.20	0.27	-0.86	-1.21	-3.00
**E-TS4-tBu**	-1.35	0.25	-0.69	-1.18	-2.97
**A-TS0-H**	-1.59	4.68	-1.26	-6.08	-4.25
**A-TS0-tBu**	-2.38	9.74	-1.68	-15.63	-9.95
**E-TS0-H**	-1.05	0.07	-0.89	-0.93	-2.80
**E-TS0-tBu**	-1.34	0.32	-1.10	-1.52	-3.64
**A-TS1-H**	-1.46	7.36	-1.28	-7.66	-3.03
**A-TS1-tBu**	-3.37	11.73	-1.67	-15.76	-9.07
**E-TS1-H**	-0.54	1.62	-0.68	-1.90	-1.50
**E-TS1-tBu**	-1.85	3.99	-0.90	-4.91	-3.68

Synthetic Details

General Information

Chemicals

Chemicals of high purity from Merck, Sigma Aldrich, and Tokyo Chemical Industries were used for this work. The solvents used were distilled before use. If necessary, work was carried out under inert gas, in which case nitrogen was used. Water−free solvents were purchased from Acros Organics.

1-Bromo-3,5-disubstitutedphenyl precursors were synthesized according to literature procedures, if not commercially available.

Thin layer chromatography

For this work, DC cards of the company Macherey−Nagel of the type SUL G/UV254, layer thickness 0.20 mm silica gel with fluorescent indicator were used. These were developed with potassium permanganate solution (5% in methanol) or molybdate phosphoric acid solution (1.5% in H_2O).

Column chromatography

Silica gel M60 (particle size 0.040 mm − 0.063 mm) from Macherey−Nagel was used for column chromatographic purification.

Gas chromatography coupled with mass spectrometry

A Hewlett−Packard type 5890 II gas chromatograph combined with an MS 5971 was used for the GC−MS analyses prepared in this work. The capillary column has a length of 30 m, an inner diameter of 0.25 mm, and a stationary phase of 95% methyl− and 5% phenylpolysiloxane.

NMR spectroscopy

^{1}H and ^{13}C nuclear magnetic resonance spectra were recorded using Bruker Avance II 400 MHz and Avance III HD 400 MHz. All ^{1}H data was measured at 400 MHz and ^{13}C in decoupled mode at 101 MHz respectively. The respective deuterated solvent was used as a shift reference. All data was recorded at 298 K. The following abbreviations were used for the multiplicities: s = singlet, d = doublet, t = triplet, q = quartet, p = pentet, td = triplet of doublet, m = multiplet, J = coupling constant.

Mass spectrometry

Electrospray ionization mass spectrometry (ESI-MS) was performed on a Bruker Daltonics Impact II ultrahigh resolution quadrupole time-of-flight (UHR qTOF) mass spectrometer.

Synthetic Procedures

3,5-Dimethylbenzaldehyde (**2-Me**)

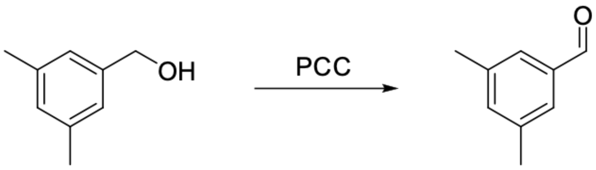

A flame-dried 100 mL flask was charged with 1.80 g (8.4 mmol, 1.25 equiv.) PCC and a small amount of SiO_2 under Schlenk conditions. The mixture was suspended in 30 mL dry DCM and cooled to 0 °C. The resulting orange suspension was treated with 0.91 g (6.7 mmol, 1.00 equiv.) of 3,5-dimethylbenzyl alcohol. The mixture turned black immediately. The solution was allowed to heat to room temperature and stirred for 4 h. The reaction mixture was diluted with 20 mL EtOAc and filtered through a silica pad on fritted glass. The precipitate was washed with 60 mL (3 x 20 mL) EtOAc. The resulting yellow liquid was concentrated under reduced pressure and purified by column chromatography (Hex:EtOAc / 95:5). The organic phase was evaporated to afford 0.5979 g 3,5dimethylbenzaldehyde (**2-Me**) (4.5 mmol, 67%) as a slightly yellow oil.

^{1}H-NMR (400 MHz, $CDCl_3$): δ (in ppm) = 9.95 (s, 1H), 7.49 (s, 2H), 7.26 (s, 1H), 2.39 (d, 6H)
^{13}C{1H}-NMR (101 MHz, $CDCl_3$): δ (in ppm) = 192.9, 138.9, 136.3, 127.7, 21.2
IR (ATR): Wavenumber (in cm^{-1}) = 681, 705, 852, 1143, 1294, 1598, 1609, 1695, 2726, 2808, 2921
HRMS (ESI-TOF) m/z: [M + Na]$^+$ calculated for $C_9H_{10}ONa^+$: 157.0624
Found: 157.0626

3,5-Diethylbenzaldehyde (**2-Et**)

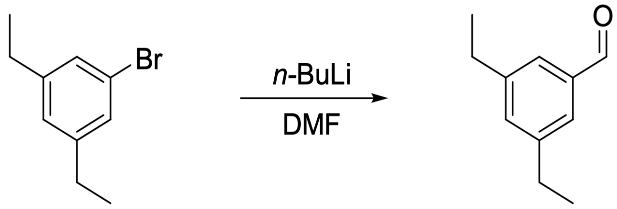

A flame-dried 100 mL flask was charged with 5.10 g (24.0 mmol, 1.0 equiv.) 1-bromo-3,5-di-ethyl-benzene under Schlenk conditions and dissolved in 20 mL dry THF. The mixture was cooled to −80 °C, after which 16.85 mL (29.0 mmol, 1.2 equiv.) *n*-butyllithium-solution (1.7 M in THF) were added over a period of 20 min. The reaction was stirred for 20 min at −80 °C. Then, 2.61 mL (34.0 mmol, 1.4 equiv.) DMF were added dropwise. The reaction was stirred for 12 h at room temperature, cooled to 0 °C and diluted with 20 mL H_2O. The organic phase was separated and the aqueous phase extracted with 100 mL (2 x 50 mL) diethylether. The organic phases were combined dried over $MgSO_4$, filtered and concentrated under reduced pressure to give a dark orange liquid. The crude product was purified by column chromatography (Hex:EtOAc / 95:5). The organic phase was evaporated to afford 2.26 g 3,5di-ethyl-benzaldehyde (**2-Et**) (14.0 mmol, 58%) as an orange liquid.

^{1}H-NMR (400 MHz, $CDCl_3$): δ (in ppm) = 9.98 (s, 1H), 7.54 (d, 2H), 7.30 (s, 1H), 2.71 (q, 4H), 1.27 (t, 6H)

^{13}C{1H}-NMR (101 MHz, $CDCl_3$): δ (in ppm) = 193.0, 145.3, 136.9, 134.2, 126.8, 28.7, 15.5

IR (ATR): Wavenumber (in cm^{-1}) = 873, 1387, 1460, 1697, 2965

HRMS (ESI-TOF) m/z: [M + Na]$^+$ calculated for $C_{11}H_{14}ONa^+$: 185.0937

Found: 185.0938

3,5-Di-*iso*-propyl-benzaldehyde (**2-^{i}Pr**)

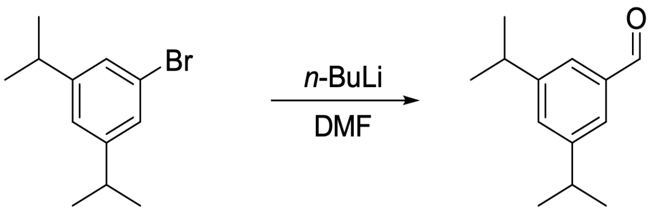

A flame-dried 100 mL flask was charged with 1.02 g (3.7 mmol, 1.0 equiv.) 3,5-di-*iso*-propyl-benzyl bromide under Schlenk conditions and dissolved in 20 mL dry THF. The mixture was cooled to −80 °C, after which 1.82 mL (4.6 mmol, 1.2 equiv.) *n*-butyllithium-solution (2.5 M in THF) were added over a period of 20 min. The reaction was stirred for 20 min at −80 °C. Then, 0.41 mL (5.3 mmol, 1.4 equiv.) DMF were added dropwise. The reaction was stirred for 12 h at room temperature, cooled to 0 °C and diluted with 20 mL H_2O. The organic phase was separated and the aqueous phase extracted with 100 mL (2 x 50 mL) diethylether. The organic phases were combined dried over $MgSO_4$, filtered and concentrated under reduced pressure to give a dark orange liquid. The crude product was purified by column chromatography (Hex:EtOAc / 95:5). The organic phase was evaporated to afford 0.40 g 3,5di-*iso*-propyl-benzaldehyde (**2-^{i}Pr**) (2.1 mmol, 56%) as an orange liquid.

^{1}H-NMR (400 MHz, $CDCl_3$): δ (in ppm) = 9.99 (s, 1H), 7.58 (d, 2H), 7.35 (t, 1H), 2.98 (sept, 2H), 1.29 (d, 12H)
^{13}C{1H}-NMR (101 MHz, $CDCl_3$): δ (in ppm) = 193.1, 150.0, 136.9, 131.7, 125.4, 34.2, 24.0
IR (ATR): Wavenumber (in cm^{-1}) = 701, 876, 1166, 1465, 1598, 1697, 2871, 2960
HRMS (ESI-TOF) m/z: [M + Na]$^+$ calculated for $C_{13}H_{18}ONa^+$: 213.1250
Found: 213.1248

3,5-Di-*tert*-butyl-benzaldehyde (**2-*ᵗ*Bu**)

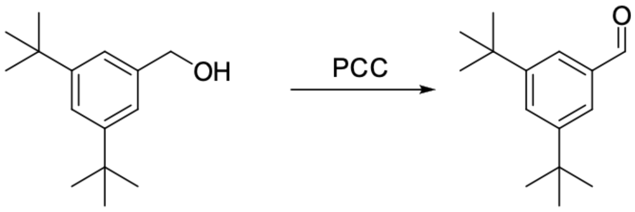

A flame-dried 100 mL flask was charged with 1.80 g (8.4 mmol, 1.25 equiv.) PCC and a small amount of SiO_2 under Schlenk conditions. The mixture was suspended in 30 mL dry DCM and cooled to 0 °C. The resulting orange suspension was treated with 1.47 g (6.7 mmol, 1.00 equiv.) of 3,5-di-*tert*-butyl-benzyl alcohol. The mixture turned black immediately. The solution was allowed to heat to room temperature and stirred for 4 h. The reaction mixture was diluted with 20 mL EtOAc and filtered through a silica pad on fritted glass. The precipitate was washed with 60 mL (3 x 20 mL) EtOAc. The resulting yellow liquid was concentrated under reduced pressure and purified by column chromatography (Hex:EtOAc / 95:5). The organic phase was evaporated to afford 1.18 g 3,5di-*tert*-butyl-benzaldehyde (**2-*ᵗ*Bu**) (5.4 mmol, 80%) as a white solid.

^{1}H-NMR (400 MHz, $CDCl_3$): δ (in ppm) = 10.01 (s, 1H), 7.72 (m, 3H), 1.37 (s, 18H)
^{13}C{1H}-NMR (101 MHz, $CDCl_3$): δ (in ppm) = 193.4, 152.0, 136.4, 129.0, 124.3, 35.1, 31.5
Melting Point: 85.7 °C
IR (ATR): Wavenumber (in cm^{-1}) = 698, 706, 882, 1188, 1363, 1463, 1591, 1686, 2868, 2959
HRMS (ESI-TOF) m/z: $[M + Na]^+$ calculated for $C_{15}H_{22}ONa^+$: 241.1563
Found: 241.1561

3,5-Dimethylbenzyl alcohol

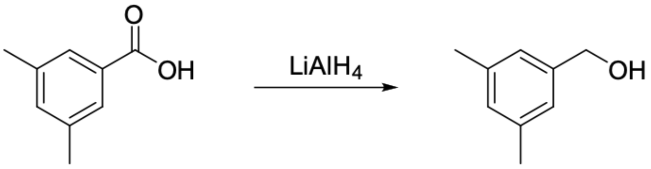

A flame-dried 250 mL flask was charged with 60 mL dry THF and cooled to 0 °C. 3.16 g (83.2 mmol, 2.5 equiv.) $LiAlH_4$ was added in portions and the mixture stirred for 10 min. The resulting suspension was treated with 5.00 g (33.3 mmol, 1.0 equiv.) 3,5-dimethyl-benzoic acid. The reaction mixture was allowed to warm to room temperature and stirred for 4 h. The solution was again cooled to 0 °C and quenched gradually with 50 mL saturated ammonium chloride solution. To ensure all $LiAlH_4$ has reacted, the mixture was stirred for 15 min. The resulting suspension was filtered in a Büchner funnel and the solids washed with EtOAc (2 x 30 mL). The aqueous layer was extracted with EtOAc (3 x 20 mL). The combined organic phase was dried over $MgSO_4$, filtered and concentrated under reduced pressure to afford 3,5-dimethylbenzyl alcohol (4.25 g, 31.2 mmol, 94%) as a clear, colorless liquid.

^{1}H-NMR (400 MHz, $CDCl_3$): δ (in ppm) = 6.99 (s, 2H), 6.94 (s, 1H), 4.62 (s, 2H), 2.33 (s, 6H), 1.64 (s, 1H)
^{13}C{1H}-NMR (101 MHz, $CDCl_3$): δ (in ppm) = 141.0, 138.3, 129.4, 125.0, 65.6, 21.4
IR (ATR): Wavenumber (in cm^{-1}) = 511, 644, 690, 840, 992, 1034, 1460, 1608, 2917, 3319
HRMS (ESI-TOF) m/z: $[M + Na]^+$ calculated for $C_9H_{12}ONa^+$: 159.0780
Found: 159.0782

3,5-Diethylbenzyl alcohol

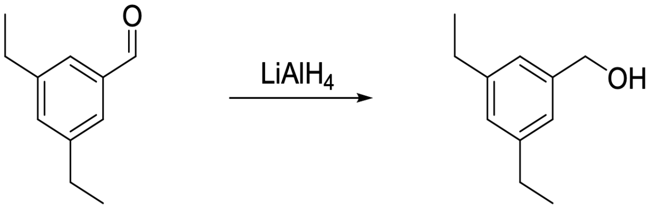

A flame-dried 250 mL flask was charged with 50 mL dry THF and cooled to 0 °C. 0.98 g (26.0 mmol, 2.5 equiv.) $LiAlH_4$ was added in portions and the mixture stirred for 10 min. The resulting suspension was treated with 1.70 g (10.0 mol, 1.0 equiv.) 3,5-di-ethyl-benzyl aldehyde (**2-Et**) over a period of 30 min. The reaction mixture was allowed to warm to room temperature and stirred for 2 h. The solution was again cooled to 0 °C and quenched gradually with 50 mL saturated ammonium chloride solution. To ensure all $LiAlH_4$ has reacted, the mixture was stirred for 15 min. The resulting suspension was filtered in a Büchner funnel and the solids washed with EtOAc (2 x 50 mL). The aqueous layer was extracted with EtOAc (3 x 30 mL). The combined organic phase was dried over Na_2SO_4, filtered and concentrated under reduced pressure to afford 3,5-di-ethyl benzyl alcohol (1.63 g, 10.0 mmol, 95%) as a clear, colorless liquid.

^{1}H-NMR (400 MHz, $CDCl_3$): δ (in ppm) = 7.04-7.03 (m, 2H), 6.98 (s, 1H), 4.66 (s, 2H), 2.64 (q, 4H), 1.67 (s, 1H), 1.25 (t, 6H)

^{13}C{1H}-NMR (101 MHz, $CDCl_3$): δ (in ppm) = 144.8, 141.1, 127.1, 124.1, 65.7, 28.9, 15.7

IR (ATR): Wavenumber (in cm^{-1}) = 3312, 2962, 1603, 1458, 858

HRMS (ESI-TOF) m/z: $[M + Na]^+$ calculated for $C_{11}H_{15}ONa^+$: 187.1093

Found: 187.1093

3,5-Di-*iso*-propylbenzyl alcohol

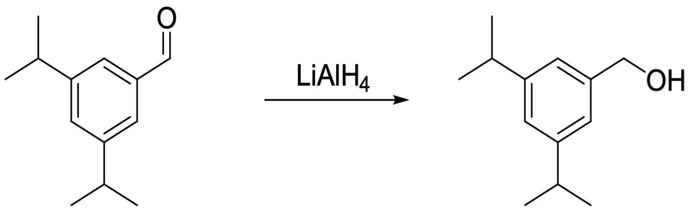

A flame-dried 250 mL flask was charged with 50 mL dry THF and cooled to 0 °C. 0.99 g (26.1 mmol, 2.5 equiv.) $LiAlH_4$ was added in portions and the mixture stirred for 10 min. The resulting suspension was treated with 1.98 g (10.4 mmol, 1.0 equiv.) 3,5-di-*iso*-propyl benzyl aldehyde (**2-^{i}Pr**) dissolved in 30 mL dry THF over a period of 30 min. The reaction mixture was allowed to warm to room temperature and stirred for 2 h. The solution was again cooled to 0 °C and quenched gradually with 50 mL saturated ammonium chloride solution. To ensure all $LiAlH_4$ has reacted, the mixture was stirred for 15 min. The resulting suspension was filtered in a Büchner funnel and the solids washed with EtOAc (2 x 50 mL). The aqueous layer was extracted with EtOAc (3 x 30 mL). The combined organic phase was dried over Na_2SO_4, filtered and concentrated under reduced pressure to afford 3,5-di-*iso*-propylbenzyl alcohol (1.05 g, 5.5 mmol, 52%) as a clear, colorless liquid.

^{1}H-NMR (400 MHz, $CDCl_3$): δ (in ppm) = 7.07 (d, 2H), 7.02 (t, 1H), 4.68 (d, 2H), 2.90 (sept, 2H), 1.59 (t, 1H), 1.26 (d, 12H)
^{13}C{1H}-NMR (101 MHz, $CDCl_3$): δ (in ppm) = 149.5, 140.9, 124.4, 122.7, 66.0, 34.3, 24.2
IR (ATR): Wavenumber (in cm^{-1}) = 712, 858, 1016, 1465, 1465, 1602, 2868, 2957, 3309
HRMS (ESI-TOF) m/z: [M + Na]$^+$ calculated for $C_{13}H_{20}ONa^+$: 215.1406
Found: 215.1408

3,5-Di-*tert*-butylbenzyl alcohol

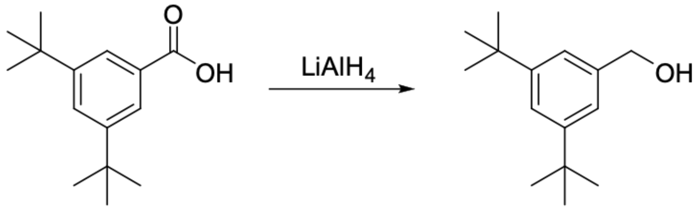

A flame-dried 100 mL flask was charged with 40 mL dry THF and cooled to 0 °C. 1.832 g (48.3 mmol, 2.2 equiv.) $LiAlH_4$ was added in portions and the mixture stirred for 10 min. The resulting suspension was treated with 5.00 g (21.3 mmol, 1.0 equiv.) 3,5-di-*tert*-butyl benzyl aldehyde dissolved in 30 mL dry THF over a period of 30 min. The reaction mixture was allowed to warm to room temperature and stirred for 2 h. The solution was again cooled to 0 °C and quenched gradually with 50 mL saturated ammonium chloride solution. To ensure all $LiAlH_4$ has reacted, the mixture was stirred for 15 min. The resulting suspension was filtered in a Büchner funnel and the solids washed with EtOAc (2 x 50 mL). The aqueous layer was extracted with EtOAc (3 x 30 mL). The combined organic phase was dried over Na_2SO_4, filtered and concentrated under reduced pressure to afford 3,5-di-*tert*-butylbenzyl alcohol (4.38 g, 19.9 mmol, 93%) as a colorless solid.

^{1}H-NMR (400 MHz, $CDCl_3$): δ (in ppm) = 7.38 (t, 1H), 7.23 (d, 2H), 4.69 (s, 2H), 1.65 (s, 1H), 1.34 (s, 18H).
^{13}C{1H}-NMR (101 MHz, $CDCl_3$): δ (in ppm) = 151.3, 140.2, 122.0, 121.5, 66.3, 35.0, 31.6 ppm.
Melting Point: 51.9 °C
IR (ATR): Wavenumber (in cm^{-1}) = 712, 872, 894, 1013, 1200, 1460, 2867, 3312
HRMS (ESI-TOF) m/z: [M + Na]$^+$ calculated for $C_{15}H_{24}ONa^+$: 243.1724
Found: 243.1725

3,5-Dimethylbenzyl bromide

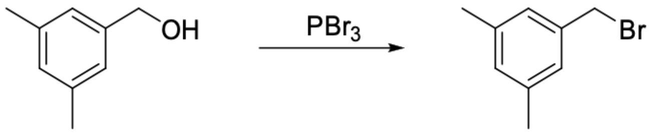

A 100 mL flask was charged with 3.07 g (22.6 mmol, 1.0 equiv.) 3,5-dimethylbenzyl alcohol and 50 mL DCM. The mixture was cooled to 0 °C and 0.86 mL (9.0 mmol 0.4 equiv.) PBr_3 were added cautiously. The reaction was allowed to warm to room temperature and stirred for 2 h. 50 mL of water was used to quench the reaction. The aqueous phase was separated and extracted with 3 x 20 mL DCM. The combined organic phase was washed with 40 mL (2 x 20 mL) $NaHCO_3$ and 20 mL brine. The organic layer was dried over $MgSO_4$, filtered and concentrated under reduced pressure. The resulting solid was further purified by column chromatography (Hex:EtOAC / 98:2) to afford 3,5-dimethylbenzyl bromide (2.73 g, 13.7 mmol, 61%) as a white solid.

^{1}H-NMR (400 MHz, $CDCl_3$): δ (in ppm) = 7.03 (m, 2H), 6.94 (m, 1H), 4.45 (s, 2H), 2.32 (d, 6H)
^{13}C{1H}-NMR (101 MHz, $CDCl_3$): δ (in ppm) = 138.6, 137.7, 130.3, 127.0, 34.0, 21.3
Melting Point: 34.6 °C
IR (ATR): Wavenumber (in cm^{-1}) = 476, 528, 698, 850, 1207, 1302, 1607, 3015
HRMS (APCI-TOF) m/z: $[M + H]^+$ calculated for $C_9H_{12}Br^+$: 199.0117
Found: 199.0117

3,5-Diethylbenzyl bromide

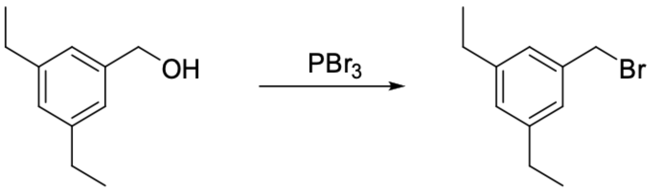

A 100 mL flask was charged with 1,63 g (10 mmol, 1.0 equiv.) 3,5-diethylbenzyl alcohol and 50 mL DCM. The mixture was cooled to 0 °C and 0.37 mL (3.9 mmol, 0.4 equiv.) PBr_3 were added cautiously. The reaction was allowed to warm to room temperature and stirred for 2 h. 50 mL of water was used to quench the reaction. The aqueous phase was separated and extracted with 3 x 20 mL DCM. The combined organic phase was washed with 40 mL (2 x 20 mL) $NaHCO_3$ and 20 mL brine. The organic layer was dried over $MgSO_4$, filtered and concentrated under reduced pressure. The resulting solid was further purified by column chromatography (Hex:EtOAC / 98:2) to afford 3,5-diethylbenzyl bromide (1.63 g, 7.2 mmol, 72%) as colorless liquid.

^{1}H-NMR (400 MHz, $CDCl_3$): δ (in ppm) = 7.06 - 7.05 (m, 2H), 6.98 (s, 1H), 4.48 (s, 2H), 2.63 (q, 4H), 1.24 (t, 6H).
^{13}C{1H}-NMR (101 MHz, $CDCl_3$): δ (in ppm) = 145.0, 137.8, 127.9, 126.1, 34.2, 28.8, 15.6.
IR (ATR): Wavenumber (in cm^{-1}) = 703, 1458, 1602, 2963, 3018
HRMS (APCI-TOF) m/z: [M + H]$^+$ calculated for $C_{11}H_{17}Br^{+}$: 227.0430
Found: 227.0430

3,5-Di-*iso*-propylbenzyl bromide

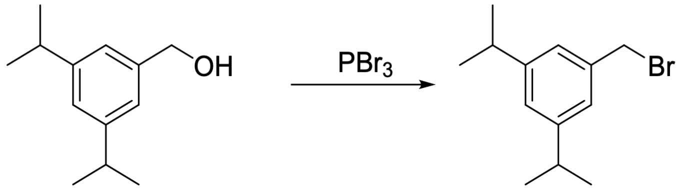

A 100 mL flask was charged with 1.05 g (5.5 mmol, 1.0 equiv.) 3,5-di-*iso*-propyl benzyl alcohol and 50 mL DCM. The mixture was cooled to 0 °C and 0.20 mL (2.1 mmol 0.4 equiv.) PBr_3 were added cautiously. The reaction was allowed to warm to room temperature and stirred for 2 h. 50 mL of water was used to quench the reaction. The aqueous phase was separated and extracted with 3 x 20 mL DCM. The combined organic phase was washed with 40 mL (2 x 20 mL) $NaHCO_3$ and 20 mL brine. The organic layer was dried over $MgSO_4$, filtered and concentrated under reduced pressure to afford 3,5-di-*iso*-propylbenzyl bromide (1.01 g, 3.9 mmol, 72%) as colorless liquid.

^{1}H-NMR (400 MHz, $CDCl_3$): δ (in ppm) = 7.09 (d, 2H), 7.03 (t, 1H), 4.50 (s, 2H), 2.90 (sept, 2H), 1.26 (d, 12H)

^{13}C{1H}-NMR (101 MHz, $CDCl_3$): δ (in ppm) = 149.6, 137.7, 125.2, 124.8, 34.4, 34.2, 24.1

IR (ATR): Wavenumber (in cm^{-1}) = 708, 877, 1465, 1601, 2958.

HRMS (APCI-TOF) m/z: $[M + H]^+$ calculated for $C_{13}H_{20}Br^+$: 255.0743

Found: 255.0741

3,5-Di-tert-butylbenzyl bromide

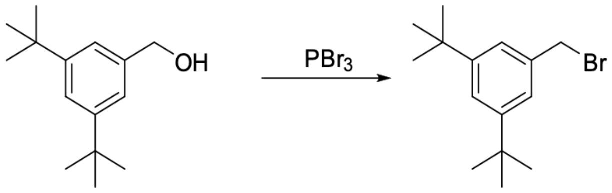

A dry 100 mL flask was charged with 5.00 g (22.7 mmol, 1.0 equiv.) 3,5-di-*tert*-butylbenzyl alcohol and 50 mL dry DCM. The mixture was cooled to 0 °C and 0.86 mL (9.1 mmol 0.4 equiv.) PBr_3 were added cautiously. The reaction was allowed to warm to room temperature and stirred for 4 h. 50 mL of water was used to quench the reaction. The aqueous phase was separated and extracted with 3 x 20 mL DCM. The combined organic phase was washed with 40 mL (2 x 20 mL) $NaHCO_3$ and 20 mL brine. The organic layer was dried over $MgSO_4$, filtered and concentrated under reduced pressure to give 3,5-di-*tert*-butylbenzyl bromide (5.15 g, 18.2 mmol, 80%) as a white solid.

^{1}H-NMR (400 MHz, $CDCl_3$): δ (in ppm) = 7.37 (t, J = 1.8 Hz, 1H), 7.24 (d, J = 1.8 Hz, 2H), 4.53 (s, 2H), 1.34 (s, 18H)
^{13}C{1H}-NMR (101 MHz, $CDCl_3$): δ (in ppm) = 151.4, 137.0, 123.4, 122.8, 35.0, 34.9, 31.5
Melting Point: 33.9 °C
IR (ATR): Wavenumber (in cm^{-1}) = 511, 543, 584, 658, 708, 878, 1215, 1248, 1363, 1477, 1599, 2867, 2904, 2962
HRMS (ESI-TOF) m/z: $[M - Br]^+$ calculated for $C_{15}H_{24}^+$: 203.1794
Found: 203.1801

1-[(Phenyl)methyl] tetrahydrothiophenium tetrafluoroborate (**1-H**)

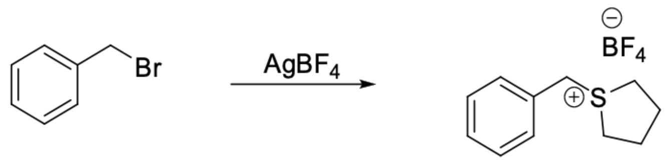

A 50 mL round-bottom flask was charged with 0.70 mL (5.8 mmol, 1.1 equiv.) benzyl bromide and 0.94 mL (10.6 mmol, 2.0 equiv.) tetrahydrothiophene. The mixture was dissolved in 25 mL acetone and the flask covered with aluminum foil. 1.03 g (5.3 mmol, 1.0 equiv.) $AgBF_4$ were added and the reaction mixture stirred for 4 h at room temperature in the dark during which a slightly green precipitate was formed. The solution was filtered *via* a syringe and the organic solvent removed under reduced pressure. The resulting solid was purified by column chromatography (first 50 mL DCM, following 50 mL acetone). The organic layer was dried over $MgSO_4$, filtered and concentrated under reduced pressure to afford 1-[(phenyl)methyl] tetrahydrothiophenium tetrafluoroborate (**1-H**) (0.78 g, 2.9 mmol, 55%) as a white solid.

^{1}H-NMR (400 MHz, $CDCl_3$): δ (in ppm) = 7.43 (m, 5H), 4.55 (m, 2H), 3.50 (m, 4H), 2.29 (m, 4H)

^{13}C{1H}-NMR (101 MHz, $CDCl_3$): δ (in ppm) = 130.7, 130.4, 130.0, 128.1, 46.2, 42.4, 28.6

Melting Point: 82.5 °C

IR (ATR): Wavenumber (in cm^{-1}) = 520, 701, 775, 1022, 1423, 1458, 2960, 3011

HRMS (ESI-TOF) m/z: $[M - BF_4^-]^+$ calculated for $C_{11}H_{15}S^+$: 179.0889

Found: 179.0889

1-[(3,5-Dimethylphenyl)methyl] tetrahydrothiophenium tetrafluoroborate (**1-Me**)

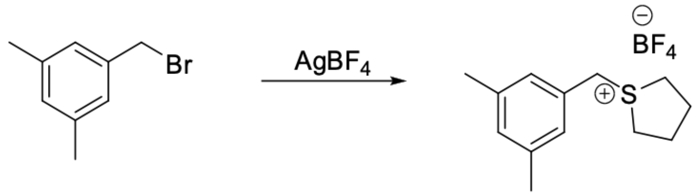

A 100 mL flask was charged with 1.00 g (5.0 mmol, 1.1 equiv.) 3,5-dimethylbenzyl bromide, 0.81 mL (9.1 mmol, 2.0 equiv.) THT and 50 mL acetone. The reaction flask was covered with aluminum foil and 0.89 g (4.6 mmol, 1.0 equiv.) $AgBF_4$ was added. The mixture was stirred for 4 h at room temperature in the dark. Afterwards, the organic phase was filtered *via* a syringe and the solvents removed under reduced pressure. The resulting grey solid was purified by column chromatography (first 50 mL DCM, following 50 mL acetone). The organic layer was dried over $MgSO_4$, filtered and concentrated under reduced pressure to afford 1-[(3,5-dimethylphenyl)methyl] tetrahydrothiophenium tetrafluoroborate (**1-Me**) (1.13 g, 3.8 mmol, 84%) as a white solid.

^{1}H-NMR (400 MHz, CDCl$_3$): δ (in ppm) = 7.04 (s, 3H), 4.45 (s, 2H), 3.58-3.39 (m, 4H), 2.35-2.27 (m, 10H)
^{13}C{1H}-NMR (101 MHz, CDCl$_3$): δ (in ppm) = 139.8, 132.0, 128.3, 127.8, 46.2, 42.4, 28.6, 21.2
Melting Point: 105.3 °C
IR (ATR): Wavenumber (in cm^{-1}) = 520, 718, 856, 1025, 1049, 1609
HRMS (ESI-TOF) m/z: [M – BF_4^-]$^+$ calculated for $C_{13}H_{19}S^+$: 207.1202
Found: 207.1205

1-[(3,5-Diethylphenyl)methyl] tetrahydrothiophenium tetrafluoroborate (**1-Et**)

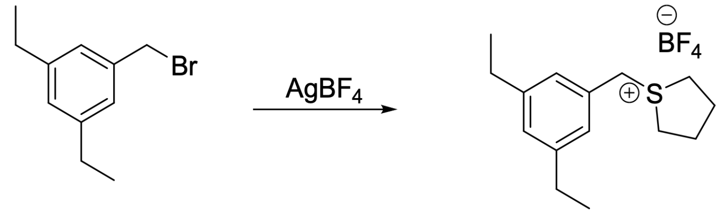

A 100 mL flask was charged with 1.44 g (6.4 mmol, 1.1 equiv.) 3,5-diethylbenzyl bromide, 1.02 mL (12 mmol, 2.0 equiv.) THT and 50 mL acetone. The reaction flask was covered with aluminum foil and 1.13 g (5.8 mmol, 1.0 equiv.) $AgBF_4$ was added. The mixture was stirred for 72 h at room temperature in the dark. Afterwards, the organic phase was filtered *via* a syringe and the solvents removed under reduced pressure. The resulting grey solid was purified by column chromatography (first 50 mL DCM, following 50 mL acetone). The organic layer was dried over $MgSO_4$, filtered and concentrated under reduced pressure to afford 1-[(3,5-diethylphenyl)methyl] tetrahydrothiophenium tetrafluoroborate (**1-Et**) (0.85 g, 2.6 mmol, 46%) as a white solid.

^{1}H-NMR (400 MHz, CDCl3): δ (in ppm) = 7.08 (s, 3H), 4.49 (s, 2H), 3.60-3.41 (m, 4H), 2.26 (q, 4H), 2.36-2.25 (m, 4H), 1.21 (t, 6H)

^{13}C{1H}-NMR (101 MHz, CDCl3): δ (in ppm) = 146.4, 129.7, 127.8, 127.5, 46.5, 42.3, 28.7, 28.6, 15.6

Melting Point: 84.9 °C

IR (ATR): Wavenumber (in cm^{-1}) = 873, 1458, 1602, 2966

HRMS (ESI-TOF) m/z: $[M – BF_4^-]^+$ calculated for $C_{15}H_{23}S^+$: 235.1515

Found: 235.1518

1-[(3,5-Di-*iso*-propyl-phenyl)methyl] tetrahydrothiophenium tetrafluoroborate (**1-^{i}Pr**)

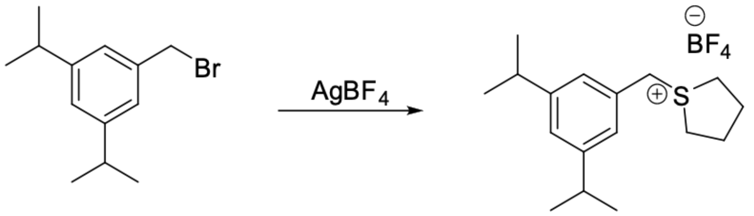

A 100 mL flask was charged with 0.51 g (2.0 mmol, 1.1 equiv.) 3,5-di-*iso*-propyl benzyl bromide, 0.32 mL (3.6 mmol, 2.0 equiv.) THT and 50 mL acetone. The reaction flask was covered with aluminum foil and 0.35 g (1.8 mmol, 1.0 equiv.) $AgBF_4$ was added. The mixture was stirred for 72 h at room temperature in the dark. Afterwards, the organic phase was filtered *via* a syringe and the solvents removed under reduced pressure. The resulting grey solid was purified by column chromatography (first 50 mL DCM, following 50 mL acetone). The organic layer was dried over $MgSO_4$, filtered and concentrated under reduced pressure to afford 1-[(3,5-di-*iso*-propylphenyl)methyl] tetrahydrothiophenium tetrafluoroborate (**1-^{i}Pr**) (0.35 g, 1.0 mmol, 51%) as a white solid.

^{1}H-NMR (400 MHz, CDCl3): δ (in ppm) = 7.11 (s, 3H), 4.49 (s, 2H), 3.60-3.39 (m, 4H), 2.87 (sept, 2H), 2.29-2.25 (m, 4H), 1.22 (d, 12H)

^{13}C{1H}-NMR (101 MHz, CDCl3): δ (in ppm) = 151.0, 127.8, 126.9, 126.2, 46.7, 42.3, 34.2, 28.6, 24.0

Melting Point: 87.1 °C

IR (ATR): Wavenumber (in cm-1) = 881, 1466, 1601, 2959

HRMS (ESI-TOF) m/z: [M – BF_4^-]$^+$ calculated for $C_{17}H_{27}S^+$: 263.1828

Found: 263.1830

1-[(3,5-Di-*tert*-butyl-phenyl)methyl] tetrahydrothiophenium tetrafluoroborate (**1-*t*Bu**)

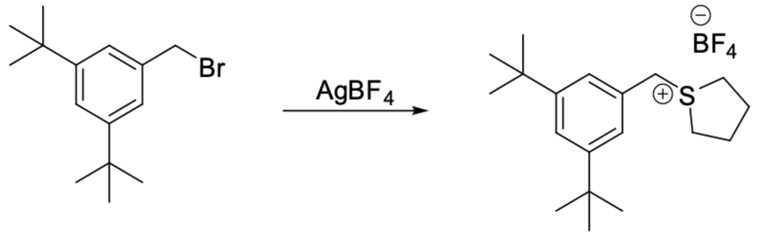

A 100 mL flask was charged with 2.144 g (7.6 mmol, 1.0 equiv.) 3,5-di-*iso*-propyl benzyl bromide, 1.33 mL (15 mmol, 2.0 equiv.) THT and 50 mL acetone. The reaction flask was covered with aluminum foil and 0.831 g (7.6 mmol, 1.0 equiv.) $NaBF_4$ was added. The mixture was stirred for 72 h at room temperature in the dark. Afterwards, the organic phase was filtered *via* a syringe and the solvents removed under reduced pressure. The resulting grey solid was purified by column chromatography (first 50 mL DCM, following 50 mL acetone). The organic layer was dried over $MgSO_4$, filtered and concentrated under reduced pressure to afford 1-[(3,5-di-*tert*-butylphenyl)methyl] tetrahydrothiophenium tetrafluoroborate (**1-*t*Bu**) (0.489 g, 1.3 mmol, 17%) as a white solid.

^{1}H-NMR (400 MHz, CDCl3): δ (in ppm) = 7.47 (t, 1H), 7.28 (d, 2H), 4.53 (s, 2H), 3.61 – 3.40 (m, 4H), 2.26 (m, 4H), 1.31 (s, 18H)

^{13}C{1H}-NMR (101 MHz, CDCl3): δ (in ppm) = 152.9, 127.2, 124.9, 124.3, 47.1, 42.3, 35.1, 31.4, 28.6

Melting Point: 163.4 °C

IR (ATR): Wavenumber (in cm-1) = 724, 1011, 1600, 2959

HRMS (ESI-TOF) m/z: $[M – BF_4^-]^+$ calculated for $C_{19}H_{31}S^+$: 291.2141

Found: 291.2143

Bis-(3,5-di-*tert*-butyl-phenyl)acetylene

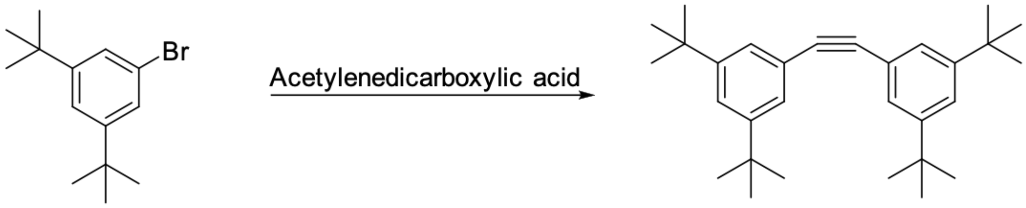

A 100 mL flame dried Schlenk flask was charged with 5.21 g (18.6 mmol, 2.0 equiv.) 3,5-di-*tert*-butyl-bromobenze, 0.65 g (0.93 mmol, 0.05 equiv.) [$(PPh_3)PdCl_2$], 0.749 g (1.86 mmol, 0.1 equiv.), 5.66 g (37.2 mmol, 4.0 equiv.) DBU, 1.06 g (9.29 mmol, 1.0 equiv.) acetylenedicarboxylic acid and 50 mL dry DMSO under N_2 atmosphere. The mixture was heated to 110 °C for 3 h. After completion of the reaction, monitored by TLC, the reaction mixture was cooled to room temperature and poured into 100 mL saturated NH_4Cl solution. The mixture was extracted with 4x 50 mL Et_2O and concentrated under reduced pressure. The product was purified by column chromatography (Hex:EtOAc / 95:5). The organic phase was evaporated to afford Bis-(3,5-di-*tert*-butyl-phenyl)acetylene (3.16 g, 7.84 mmol, 84%) as light yellow powder.

^{1}H-NMR (400 MHz, $CDCl_3$): δ (in ppm) = 7.43 – 7.35 (m, 6H), 1.35 (d, 36H)
^{13}C{1H}-NMR (101 MHz, $CDCl_3$): δ (in ppm) = 150.9, 126.0, 122.7, 122.6, 89.4, 35.0, 31.5
Melting Point: 184.5 °C
IR (ATR): Wavenumber (in cm^{-1}) = 535, 702, 872, 1244, 1362, 1590, 2952
HRMS (EI-TOF) m/z: $[M + H]^+$ calculated for $C_{30}H_{43}^+$: 403.3359
Found: 403.3364

1,1′-(1*Z*)-1,2-Ethenediylbis[3,5-di-*tert*-butylbenzene]

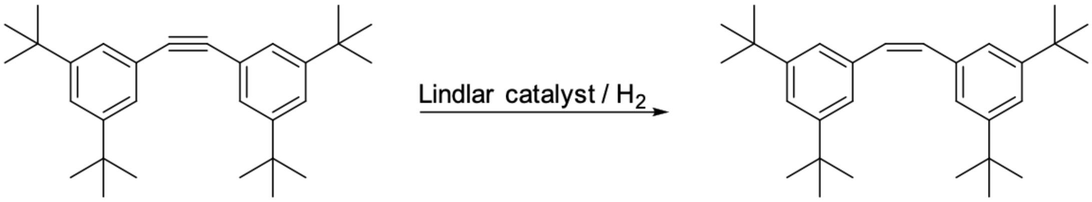

A flame dried 250 mL Schlenk flask was charged with 2.00 g (4.95 mmol, 1.00 equiv.) Bis-(3,5-di-tert-butyl-phenyl)acetylene, 1.02 g (5% Pd content, 0.48 mmol, 0.10 equiv.) Lindlar catalyst and 1 mL (8.5 mmol, 1.7 equiv.) quinoline, and 100 mL dry hexane. The flask was stirred at r.t. under H_2 atmosphere for 7 days. After completion of the reaction, monitored by TLC, the reaction was filtered and 10 mL 1M HCl solution and 50 mL H_2O was added. The phases were separated and the organic was additionally washed once with brine. The organic phase was dried over Na_2SO_4 and concentrated under reduced pressure to yield 1,1′-(1*Z*)-1,2-Ethenediylbis[3,5-di-*tert*-butylbenzene] (1.69 g, 4.18 mmol, 84%) as colourless oil.

^{1}H-NMR (400 MHz, $CDCl_3$): δ (in ppm) = 7.23 (t, 2H), 7.01 (d, 4H), 6.66 (s, 2H), 1.21 (s, 36H)

^{13}C{1H}-NMR (101 MHz, $CDCl_3$): δ (in ppm) = 150.7, 137.3, 131.3, 123.1, 34.8, 31.5

IR (ATR): Wavenumber (in cm^{-1}) = 708, 742, 828, 1362, 1476, 2953.

HRMS (ESI-TOF) m/z: [M + H]$^+$ calculated for $C_{30}H_{45}^+$: 405.3516

Found: 405.3513

1,1′-(1E)-1,2-Ethenediylbis[3,5-di-*tert*-butylbenzene]

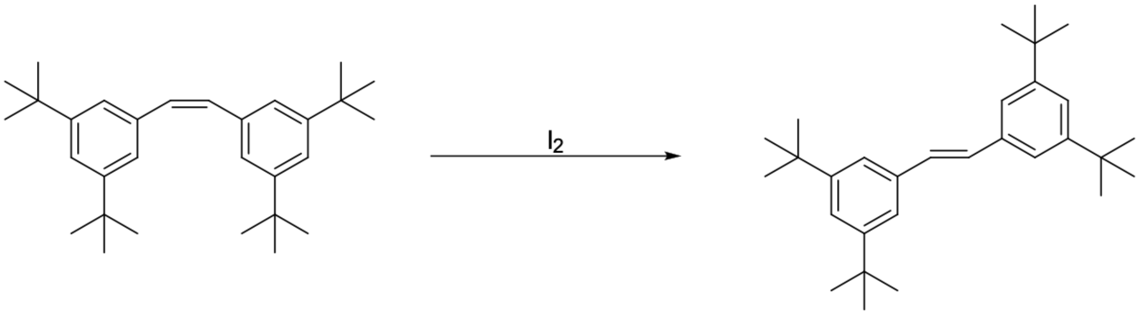

A 100 mL flask was charged 0.75 g (1.85 mmol, 1.00 equiv.) 1,1′-(1Z)-1,2-Ethenediylbis[3,5-di-*tert*-butylbenzene] and 0.05 g (0.2 mmol, 0.10 equiv.) iodine and 50 ml distilled *n*-hexane and refluxed for 72 h. After cooling to r.t. the solvent was removed under reduced pressure. The resulting oil was further purified by column chromatography (*n*-hexane, R_f = 0.27) to yield 1,1′-(1E)-1,2-Ethenediylbis[3,5-di-*tert*-butylbenzene] (0.61 g, 1.50 mmol, 82%) as a colourless oil.

^{1}H-NMR (400 MHz, $CDCl_3$): δ (in ppm) = 7.40 (2, 1H), 7.36 (d, 4H), 7.14 (s, 2H), 1.39 (s, 36H)
^{13}C{1H}-NMR (101 MHz, $CDCl_3$): δ (in ppm) = 151.1, 136.9, 129.2, 35.0, 31.7
IR (ATR): Wavenumber (in cm-1) = 707, 878, 901, 1264, 1393, 1592, 2866, 2952.
HRMS (ESI-TOF) m/z: [M + H]$^+$ calculated for $C_{30}H_{45}^+$: 405.3516
Found: 405.3515

Cis-diphenyloxiran (**3-*cis*-H**)

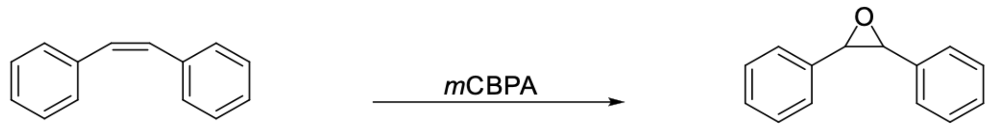

A flame dried 100 mL Schlenk flask was charged under nitrogen with 1.087 g (6.30 mmol, 3.8 equiv.) 3-Chlorobenzene-1-carboperoxoic acid and 50 mL dry CH_2Cl_2. A solution containing 0.296 g (1.64 mmol, 1.00 equiv.) in 5 mL dry CH_2Cl_2 was added dropwise over 15 minutes at 0 °C. After complete addition the mixture was stirred at r.t. for 16 h. The solvent was removed under reduced pressure. To the resulting solid 50 mL concentrated $NaHCO_3$ solution was added and extracted 3 times with 50 mL diethylether. The combined organic phases were dried over Na_2SO_4 and concentrated under reduced pressure, afterwards purified by column chromatography (Hex:EtOAc / 95:5). The organic phase was evaporated to afford cis-diphenyloxiran (**3-*cis*-H**) (0.243 g, 1.24 mmol, 76%) as a white solid

^{1}H-NMR (400 MHz, CD_2Cl_2): δ (in ppm) = 7.22 – 7.15 (m, 10H), 4.36 (s, 2H)
^{13}C{1H}-NMR (101 MHz, CD_2Cl_2): δ (in ppm) = 1135.0, 128.1, 127.8, 127.2, 60.1
Melting Point: 39.1 °C
IR (ATR): Wavenumber (in cm-1) = 527, 586, 694, 730, 747, 896, 1368, 1407, 1451, 1496.
HRMS (ESI-TOF) m/z: [M + H]$^+$ calculated for $C_{14}H_{13}O^+$: 197.0961
Found: 197.0961

Trans-diphenyloxiran (**3-*trans*-H**)

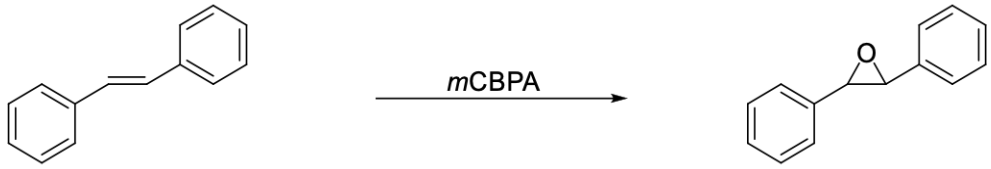

A flame dried 100 mL Schlenk flask was charged under nitrogen with 1.6305 g (9.45 mmol, 3.8 equiv.) 3-Chlorobenzene-1-carboperoxoic acid and 75 mL dry CH_2Cl_2. A solution containing 0.445 g (2.47 mmol, 1.00 equiv.) in 10 mL dry CH_2Cl_2 was added dropwise over 15 minutes at 0 °C. After complete addition the mixture was stirred at r.t. for 16 h. The solvent was removed under reduced pressure. To the resulting solid 50 mL concentrated $NaHCO_3$ solution was added and extracted 3 times with 50 mL diethylether. The combined organic phases were dried over Na_2SO_4 and concentrated under reduced pressure, afterwards purified by column chromatography (Hex:EtOAc / 95:5). The organic phase was evaporated to afford trans-diphenyloxiran (**3-*trans*-H**) (0.451 g, 2.30 mmol, 93%) as a white solid

^{1}H-NMR (400 MHz, CD_2Cl_2): δ (in ppm) = 7.43 – 7.32 (m, 10H), 3.88 (s, 2H)
^{13}C{1H}-NMR (101 MHz, CD_2Cl_2): δ (in ppm) = 137.7, 128.9, 128.7, 125.9, 63.1
Melting Point: 64.3 °C
IR (ATR): Wavenumber (in cm-1) = 507, 694, 741, 796, 835, 852, 1021, 1070, 1451, 1487.
HRMS (ESI-TOF) m/z: [M + H]$^+$ calculated for $C_{14}H_{13}O^+$: 197.0961
Found: 197.0962

Cis-2,3-Bis[3,5-di-*tert*-butylphenyl]oxirane (**3-*cis*-^{t}Bu**)

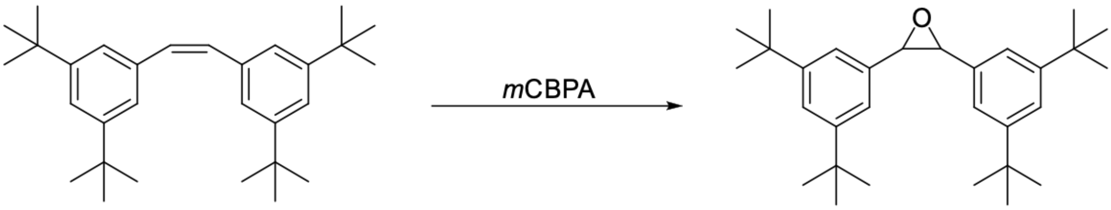

A flame dried 100 mL Schlenk flask was charged under nitrogen with 0.9602 g (5.56 mmol, 2.3 equiv.) 3-Chlorobenzene-1-carboperoxoic acid and 50 mL dry CH_2Cl_2. A solution containing 0.7636 g 1,1′-(1*Z*)-1,2-Ethenediylbis[3,5-di-*tert*-butylbenzene] (1.89 mmol, 1.00 equiv.) in 5 mL dry CH_2Cl_2 was added dropwise over 10 minutes at 0 °C. After complete addition the mixture was stirred at r.t. for 16 h. The solvent was removed under reduced pressure. To the resulting solid 50 mL concentrated $NaHCO_3$ solution was added and extracted 3 times with 50 mL diethylether. The combined organic phases were dried over Na_2SO_4 and concentrated under reduced pressure. The crude product was purified purified by column chromatography (Hex:EtOAc / 50:1) to yield *cis*-2,3-Bis[3,5-di-*tert*-butylphenyl]oxirane (**3-*cis*-^{t}Bu**) (0.675 g, 1.60 mmol, 85 %) as colourless oil, which was directly used in the next step.

^{1}H-NMR (400 MHz, $CDCl_3$): δ (in ppm) = 7.16 (t, 2H), 6.89 (d, 4H), 4.34 (s, 2H), 1.15 (s, 36H)
^{13}C{1H}-NMR (101 MHz, $CDCl_3$): δ (in ppm) = 150.2, 134.1, 121.6, 121.3, 59.8, 31.5
IR (ATR): Wavenumber (in cm^{-1}) = 702, 855, 920, 1208, 1250, 1603, 2854, 2953.
HRMS (ESI-TOF) m/z: [M + H]$^+$ calculated for $C_{30}H_{45}O^+$: 443.3284
Found: 443.3281

Trans-2,3-Bis[3,5-di-*tert*-butylphenyl]oxirane (**3-*trans*-^{t}Bu**)

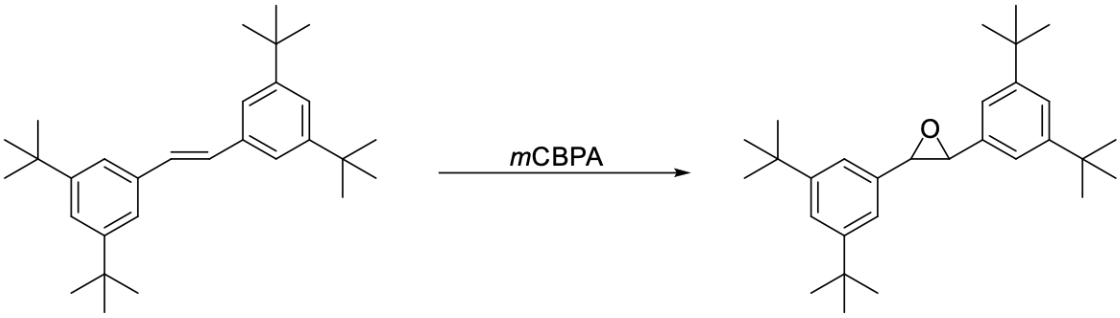

A flame dried 100 mL Schlenk flask was charged under nitrogen with 0.733 g (3.27 mmol, 2.2 equiv.) 3-Chlorobenzene-1-carboperoxoic acid and 50 mL dry CH_2Cl_2. A solution containing 0.6012 g 1,1′-(1E)-1,2-Ethenediylbis[3,5-di-*tert*-butylbenzene] (1.49 mmol, 1.00 equiv.) in 5 mL dry CH_2Cl_2 was added dropwise over 10 minutes at 0 °C. After complete addition the mixture was stirred at r.t. for 16 h. The solvent was removed under reduced pressure. To the resulting solid 50 mL concentrated $NaHCO_3$ solution was added and extracted 3 times with 50 mL diethylether. The combined organic phases were dried over Na_2SO_4 and concentrated under reduced pressure. The crude product was purified purified by column chromatography (Hex:EtOAc / 50:1) to yield *trans*-2,3-Bis[3,5-di-*tert*-butylphenyl]oxirane (**3-*trans*-^{t}Bu**) (0.519 g, 1.23 mmol, 81 %) as white solid, which was directly used in the next step.

^{1}H-NMR (400 MHz, $CDCl_3$): δ (in ppm) = 7.42 (t, 2H), 7.23 (d, 4H), 3.90 (s, 2H), 1.36 (s, 36H)
^{13}C{1H}-NMR (101 MHz, $CDCl_3$): δ (in ppm) = 151.3, 136.7, 122.5, 119.8, 63.7, 35.1, 31.6
Melting Point: 131.2 °C
IR (ATR): Wavenumber (in cm-1) = 710, 852, 895, 924, 1247, 1362, 1476, 1600, 2855, 2953,
HRMS (ESI-TOF) m/z: [M + H]$^+$ calculated for $C_{30}H_{45}O^+$: 443.3284
Found: 443.3281

Syn-1,2-diphenyl-2-hydroxyethyl(dimethyl) sulfonium iodide (**4-*syn*-H**)

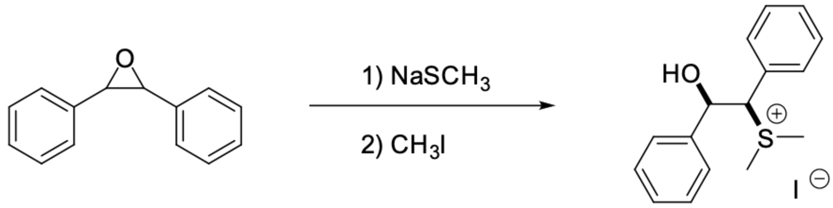

To a solution of 0.501 g, cis-diphenyloxiran (**3-*cis*-H**) (2.55 mmol, 1.0 equiv.) in 50 mL destillted ethanol 2.26 mL of a solution containing 15% wt. Methylmercaptansodiumsalt in H_2O (4.88 mmol, 1.9 equiv.) was added and refluxed for 1.5 h. Afterwards 5 mL of H_2O were added, and the Ethanol was removed under reduced pressure. The watery phase was extracted three times 20 mL diethylether. The organic phase was washed with brine, dried over Na_2SO_4 and concentrated under reduced pressure to give the syn-hydroxy sulfide. Without further analysis, the crude reaction product was stirred in 5 mL methyl iodide (80 mmol, 32 equiv.) for 16 h. The methyl iodide was removed under reduced pressure and the solid residue washed several times with diethylether to yield 0.846 g (2.20 mmol, 86%) syn-1,2-diphenyl-2-hydroxyethyl(dimethyl) sulfonium iodide (**4-*syn*-H**) as off white solid.

^{1}H-NMR (400 MHz, DMSO-d6): δ (in ppm) = 7.37 – 7.30 (m, 5H), 7.22 – 7.11 (m, 5H), 6.72 (dd, 1H), 5.53 (t, 1H), 5.20 (d, 1H), 3.10 (s, 3H), 2.59 (s, 3H).

^{13}C{1H}-NMR (101 MHz, DMSO-d6): δ (in ppm) = 140.8, 130.3, 130.0, 129.4, 129.1, 128.2, 128.2, 127.1, 73.0, 66.1, 26.3, 22.1

Melting Point: Decomposition observed at 146 °C

IR (ATR): Wavenumber (in cm-1) = 698, 786, 1059, 1090, 1430, 1451, 1490, 3163.

HRMS (ESI-TOF) m/z: [M – I]$^{+}$ calculated for $C_{16}H_{19}OS^{+}$: ([M]$^{+}$): 259.1151

Found: 259.1153

Anti-1,2-diphenyl-2-hydroxyethyl(dimethyl) sulfonium iodide (**4-*anti*-H**)

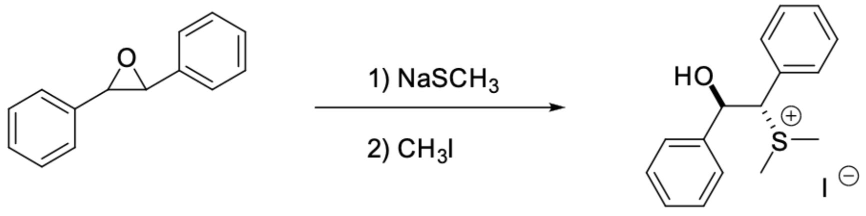

To a solution of 0.099 g, *trans*-diphenyloxiran (**3-*trans*-H**) (0.501, mmol, 1.0 equiv.) in 10 mL destillted ethanol 0.46 mL of a solution containing 15% wt. Methylmercaptansodiumsalt in H_2O (0.98 mmol, 1.9 equiv.) was added and refluxed for 1.5 h. Afterwards 5 mL of H_2O were added, and the Ethanol was removed under reduced pressure. The watery phase was extracted three times 10 mL diethylether. The organic phase was washed with brine, dried over Na_2SO_4 and concentrated under reduced pressure to give the syn-hydroxy sulfide. Without further analysis, the crude reaction product was stirred in 2 mL methyl iodide (32 mmol, 64 equiv.) for 16 h. The methyl iodide was removed under reduced pressure and the solid residue washed several times with diethylether to yield 0.182 g (0.47 mmol, 94%) trans-1,2-diphenyl-2-hydroxyethyl(dimethyl) sulfonium iodide (**4-*anti*-H**) as off white solid.

^{1}H-NMR (400 MHz, DMSO-d6): δ (in ppm) = 7.37 – 7.30 (m, 5H), 7.23 – 7.12 (m, 5H), 6.72 (d, 1H), 5.54 (t, 1H), 5.20 (d, 1H), 3.10 (s, 3H), 2.59 (s, 3H)

^{13}C{1H}-NMR (101 MHz, DMSO-d6): δ (in ppm) = 139.9, 130.6, 129.6, 129.2, 128.9, 127.9, 127.5, 126.0, 66.9, 65.1, 23.8, 23.3

Melting Point: Decomposition observed at 150 °C

IR (ATR): Wavenumber (in cm-1) = 529, 693, 708, 1076, 1326, 1489, 1539, 2981, 3288

HRMS (ESI-TOF) m/z: [M – I]$^+$ calculated for $C_{16}H_{19}OS^+$: 259.1151

Found: 259.1151

Syn-1,2-Bis[3,5-di-tert-butyl]phenyl-2-hydroxyethyl(dimethyl) sulfonium iodide (**4-*syn*-^{t}Bu**)

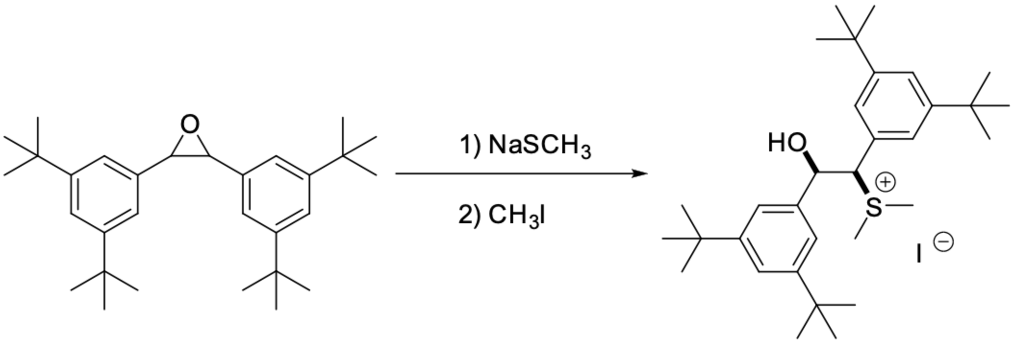

To a solution of 0.350 g, *Cis*-2,3-Bis[3,5-di-tert-butylphenyl]oxirane (0.83 mmol, 1.0 equiv.) (**3-*cis*-^{t}Bu**) in 10 mL destillted ethanol 0.75 mL of a solution containing 15% wt. Methylmercaptansodiumsalt in H_2O (1.58 mmol, 1.9 equiv.) was added and refluxed for 16 h. Afterwards 5 mL of H_2O were added, and the Ethanol was removed under reduced pressure. The watery phase was extracted three times 10 mL diethylether. The organic phase was washed with brine, dried over Na_2SO_4 and concentrated under reduced pressure to give the syn-hydroxy sulfide. Without further analysis, the crude reaction product was stirred in 5 mL methyl iodide (80 mmol, 96 equiv.) for 16 h. The methyl iodide was removed under reduced pressure and the solid residue washed several times with diethylether to yield 0.385 g (0.63 mmol, 76%) of *syn*-1,2-Bis[3,5-di-tert-butyl]phenyl-2-hydroxyethyl(dimethyl) sulfonium (**4-*syn*-^{t}Bu**) white solid.

^{1}H-NMR (400 MHz, DMSO-d6): δ (in ppm) = 7.28 (t, 1H), 7.17 – 7.05 (m, 3H), 6.99 (d, 2H), 6.68 (d, 1H), 5.46 (dd, 1H), 5.19 (d, 1H), 2.96 (s, 3H), 2.50 (s, 3H), 1.19 (s, 18H), 1.15 (s, 18H)
^{13}C{1H}-NMR (101 MHz, DMSO-d6): δ (in ppm) = 151.0, 149.9, 140.0, 129.0, 124.4, 122.5, 121.2, 121.1, 74.1, 67.2, 34.5, 34.3, 31.1, 31.0, 26.6, 22.6
Melting Point: Decomposition observed at 167 °C
IR (ATR): Wavenumber (in cm-1) = 717, 755, 882, 1045, 1188, 1203, 1363, 1476, 1599, 2953
HRMS (ESI-TOF) m/z: [M – I]$^+$ calculated for $C_{32}H_{51}OS^+$: 483.3655
Found: 483.3654

Anti-1,2-Bis[3,5-di-tert-butyl]phenyl-2-hydroxyethyl(dimethyl) sulfonium iodide (**4-*anti*-^{t}Bu**)

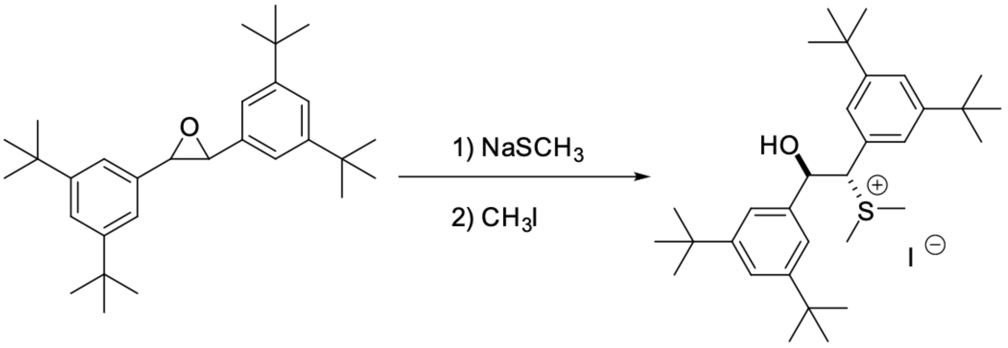

To a solution of 0.650 g, trans-2,3-Bis[3,5-di-tert-butylphenyl]oxirane (1.55 mmol, 1.0 equiv.) (**3-*anti*-^{t}Bu**) in 30 mL destillted ethanol 1.37 mL of a solution containing 15% wt. Methylmercaptansodiumsalt in H_2O (2.93 mmol, 1.9 equiv.) was added and refluxed for 16 h. Afterwards 5 mL of H_2O were added, and the Ethanol was removed under reduced pressure. The watery phase was extracted three times 20 mL diethylether. The organic phase was washed with brine, dried over Na_2SO_4 and concentrated under reduced pressure to give the syn-hydroxy sulfide. Without further analysis, the crude reaction product was stirred in 5 mL methyl iodide (80 mmol, 52 equiv.) for 16 h. The methyl iodide was removed under reduced pressure and the solid residue washed several times with diethylether to yield 0.729 g (1.19 mmol, 77%) of anti-1,2-Bis[3,5-di-tert-butyl]phenyl-2-hydroxyethyl(dimethyl) sulfonium (**4-*anti*-^{t}Bu**) as white solid.

^{1}H-NMR (400 MHz, DMSO-d6): δ (in ppm) = 7.30 (t, 1H), 7,11 (t, 1H), 7.03 (s, 2H), 6.96 (d, 2H), 6.52 (d, 1H), 5.48 (t, 1H), 5.13 (d, 1H), 3.12 (s, 3H), 2.59 (s, 3H), 1.17 (s, 18H), 1.14 (s, 18H)

^{13}C{1H}-NMR (101 MHz, DMSO-d6): δ (in ppm) = 150.39, 149.6, 139.01, 128.9, 124.2, 122.6, 120.5, 120.1, 70.1, 66.1, 34.4, 31.2, 31.1, 25.6, 23.4

Melting Point: Decomposition observed at 168 °C

IR (ATR): Wavenumber (in cm-1) = 542, 715, 819, 924, 1143, 1428, 1592, 2950,

HRMS (ESI-TOF) m/z: [M – I]$^+$ calculated for $C_{32}H_{51}OS^+$: 483.3655

Found: 483.3656

2-(3,5-Dimethylphenyl)-3-phenyl oxirane (**3-H-Me**)

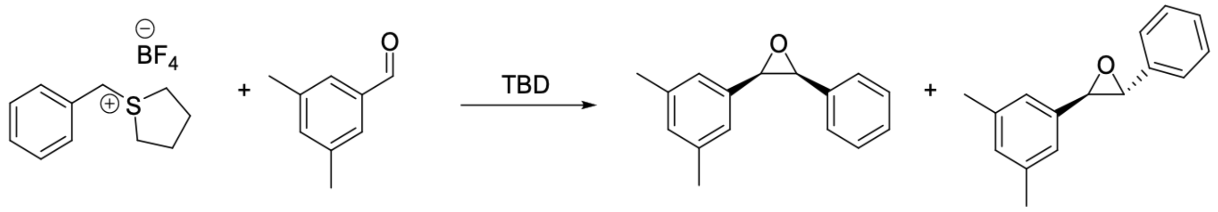

A 50 mL round-bottomed flask was charged with 0.33 g (1.24 mmol, 2.0 equiv.) thiophenium salt (**1-H**) and 0.08 g (0.62 mmol, 1.0 equiv.) aldehyde (**2-Me**). The mixture was dissolved in 20 mL DCM. Afterwards, 0.19 g (1.37 mmol, 2.2 equiv) TBD was added at room temperature. The reaction was stirred for 8 h, after which it was quenched by adding 20 mL of water. The organic layer was washed with 40 mL (2 x 20 mL) of water. The combined aqueous layer was extracted with 60 mL (3 x 20 mL) DCM. The organic phase was separated and dried over $MgSO_4$, filtered and concentrated under reduced pressure to give slightly yellow oil. The resulting product was further purified by column chromatography (Hex:EtOAC / 2:1) to afford 2-(3,5-dimethylphenyl)-3-phenyl oxirane (**3-H-Me**) as a cis/trans mixture (0.08 g, 0.41 mmol, 30%, cis/trans ratio: 0.22:1) as a colorless oil.

^{1}H-NMR (400 MHz, $CDCl_3$): δ (in ppm) = 7.36 (m, 5H, *trans*), 7.20 (m, 5H, *cis*), 6.98 (m, 3H, *trans*), 6.79 (m, 3H, *cis*), 4.33 (d, J = 4.4 Hz, 1H, *cis*), 4.30 (d, J = 4.3 Hz, 1H, *cis*), 3.86 (d, J = 2.0 Hz, 1H, *trans*), 3.81 (d, J = 2.0 Hz, 1H, *trans*), 2.34 (s, 6H, *trans*), 2.19 (s, 6H, *cis*)
^{13}C{1H}-NMR (101 MHz, $CDCl_3$): δ (in ppm) = 138.4, 137.4, 137.4, 137.2, 134.7, 134.3, 130.1, 129.3, 128.7, 128.4, 127.8, 127.6, 127.1, 125.6, 124.8, 123.4, 63.1, 62.8, 60.0, 60.0, 21.4, 21.3
HRMS (ESI-TOF) m/z: [M + Na]$^+$ calculated for $C_{16}H_{16}ONa^+$: 247.1093
Found: 247.1094

2-(3,5-Di-iso-propylphenyl)-3-phenyl oxirane (**3-H-^{i}Pr**)

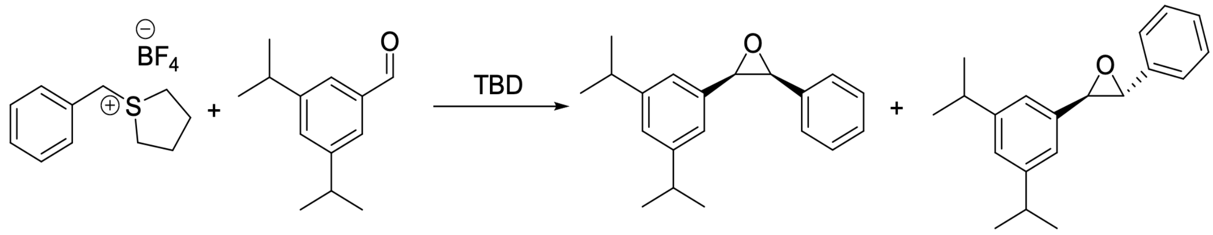

A 50 mL round-bottomed flask was charged with 0.11 g (0.42 mmol, 2.0 equiv.) thiophenium salt (**1-H**) and 0.04 g (0.21 mmol, 1.0 equiv.) aldehyde (**2-^{i}Pr**). The mixture was dissolved in 20 mL DCM. Afterwards, 0.06 g (0.46 mmol, 2.2 equiv) TBD was added at room temperature. The reaction was stirred for 8 h, after which it was quenched by adding 20 mL of water. The organic layer was washed with 40 mL (2 x 20 mL) of water. The combined aqueous layer was extracted with 60 mL (3 x 20 mL) DCM. The organic phase was separated and dried over $MgSO_4$, filtered and concentrated under reduced pressure to give slightly orange oil. The resulting product was further purified by column chromatography (Hex:EtOAC / 2:1) to afford 2-(3,5-di-*iso*-propylphenyl)-3-phenyl oxirane (**3-H-^{i}Pr**) as a cis/trans mixture (0.4 g, 0.15 mmol, 70%, cis/trans ratio: 0.26:1) as a colorless oil.

^{1}H-NMR (400 MHz, $CDCl_3$): δ (in ppm) = 7.41-7.34 (m, 5H, *trans*), 7.20-7.15 (m, 5H, *cis*), 7.07-7.05 (m, 3H, *trans*), 6.84-6.82 (m, 3H, *cis*), 4.37 (d, J = 4.3 Hz, 1H, *cis*), 4.33 (d, J = 4.3 Hz, 1H, *cis*), 3.90 (d, J = 1.9 Hz, 1H, *trans*), 3.86 (d, J = 1.9 Hz, 1H, *trans*), 1.28 (d, 12H, *trans*), 1.13-1.10 (m, 12H, *cis*)

^{13}C{1H}-NMR (101 MHz, $CDCl_3$): δ (in ppm) = 149.5, 148.3, 137.5, 137.1, 134.8, 134.2, 128.7, 128.3, 127.8, 127.5, 127.1, 125.6, 125.1, 124.5, 122.6, 121.1, 63.4, 62.9, 59.9, 59.7, 34.3, 34.1, 24.2, 23.9

HRMS (ESI-TOF) m/z: [M + Na]$^{+}$ calculated for $C_{20}H_{24}ONa^{+}$: 303.1719

Found: 303.1715

2-(3,5-Di-tert-butylphenyl)-3-phenyl oxirane (**3-H-^{t}Bu**)

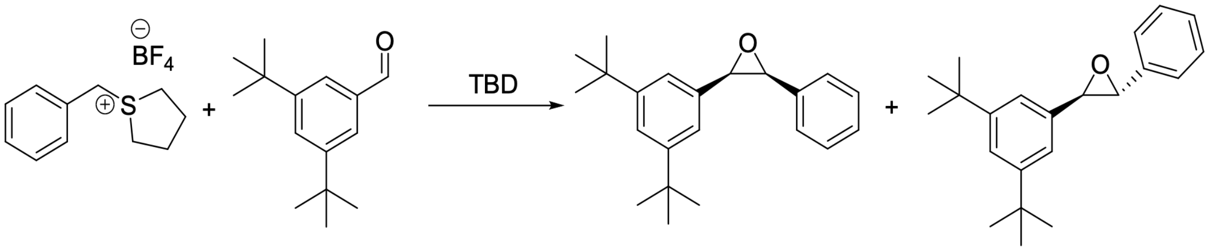

A 50 mL round-bottomed flask was charged with 0.10 g (0.38 mmol, 2.0 equiv.) thiophenium salt (**1-H**) and 0.04 g (0.19 mmol, 1.0 equiv.) aldehyde (**2-^{t}Bu**). The mixture was dissolved in 20 mL DCM. Afterwards, 0.06 g (0.41 mmol, 2.2 equiv) TBD was added at room temperature. The reaction was stirred for 8 h, after which it was quenched by adding 20 mL of water. The organic layer was washed with 40 mL (2 x 20 mL) of water. The combined aqueous layer was extracted with 60 mL (3 x 20 mL) DCM. The organic phase was separated and dried over $MgSO_4$, filtered and concentrated under reduced pressure to give slightly yellow oil. The resulting product was further purified by column chromatography (Hex:EtOAC / 2:1) to afford 2-(3,5-dimethylphenyl)-3-phenyl oxirane (**3-H-^{t}Bu**) as a cis/trans mixture (0.04 g, 0.11 mmol, 58%, cis/trans ratio: 0.23:1) as a colorless oil.

^{1}H-NMR (400 MHz, $CDCl_3$): δ (in ppm) = 7.44-7.33 (m, 10H, *cis*/*trans*), 7.22-7.17 (m, 4H, *cis/trans*), 6.97-6.96 (m, 2H, *cis*), 4.39 (d, J = 4.3 Hz, 1H, *cis*), 4.35 (d, J = 4.3 Hz, 1H, *cis*), 3.91 (d, J = 1.9 Hz, 1H, *trans*), 3.89 (d, J = 1.9 Hz, 1H, *trans*), 1.36 (s, 18H, *trans*), 1.20 (s, 18H, *cis*)

^{13}C{1H}-NMR (101 MHz, $CDCl_3$): δ (in ppm) = 151.3, 150.1, 137.5, 136.4, 134.9, 133.4, 128.7, 128.3, 127.8, 127.4, 127.2, 125.6, 122.7, 121.6, 121.4, 119.8, 63.8, 62.9, 60.3, 60.0, 35.1, 34.8, 31.6, 31.4

HRMS (ESI-TOF) m/z: [M + Na]$^+$ calculated for $C_{22}H_{28}ONa^+$: 331.2032

Found: 331.2029

2,3-Di-(3,5-dimethyl)-phenyl oxirane (**3-Me-Me**)

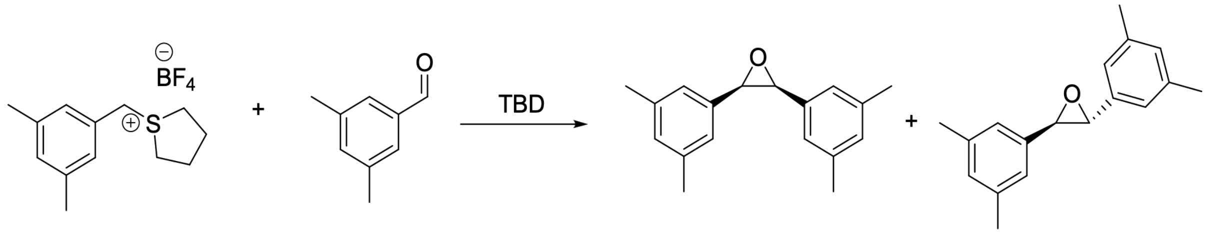

A 50 mL round-bottomed flask was charged with 0.31 g (1.05 mmol, 2.0 equiv.) thiophenium salt (**1-Me**) and 0.07 g (0.53 mmol, 1.0 equiv.) aldehyde (**2-Me**). The mixture was dissolved in 20 mL DCM. Afterwards, 0.16 g (1.16 mmol, 2.2 equiv) TBD was added at room temperature. The reaction was stirred for 8 h, after which it was quenched by adding 20 mL of water. The organic layer was washed with 40 mL (2 x 20 mL) of water. The combined aqueous layer was extracted with 60 mL (3 x 20 mL) DCM. The organic phase was separated and dried over $MgSO_4$, filtered and concentrated under reduced pressure to give slightly yellow oil. The resulting product was further purified by column chromatography (Hex:EtOAC / 2:1) to afford 2,3di-(3,5-dimethyl)-phenyl oxirane (**3-Me-Me**) as a cis/trans mixture (0.09 g, 0.36 mmol, 31%, cis/trans ratio: 0.29:1) as a colorless oil.

^{1}H-NMR (400 MHz, $CDCl_3$): δ (in ppm) = 6.96–6.95 (m, 6H, *trans*), 6.82 (m, 4H, *cis*), 6.78 (m, 2H, *cis*), 4.26 (s, 2H, *cis*), 3.79 (s, 2H, *trans*), 2.33 (s, 12H, *trans*), 2.20 (s, 12H, *cis*)

^{13}C{1H}-NMR (101 MHz, $CDCl_3$): δ (in ppm) = 138.4, 137.3, 137.3, 134.4, 130.1, 129.3, 124.8, 123.4, 62.9, 60.2, 21.4, 21.3

HRMS (ESI-TOF) m/z: [M + Na]$^+$ calculated for $C_{18}H_{20}ONa^+$: 275.1406

Found: 275.1407

NMR Spectroscopy

General Information

^{1}H and ^{13}C nuclear magnetic resonance spectra were recorded using Bruker Avance II 400 MHz and Avance III HD 400 MHz. All ^{1}H data was measured at 400 MHz and ^{13}C in decoupled mode at 101 MHz respectively. The respective deuterated solvent was used as a shift reference. All data was recorded at 298K. The following abbreviations were used for the multiplicities: s = singlet, d = doublet, t = triplet, q = quartet, p = pentet, td = triplet of doublet, m = multiplet, J = coupling constant.

3,5-Dimethylbenzaldehyde (**2-Me**)

^{1}H-NMR (400 MHz, CDCl$_3$):

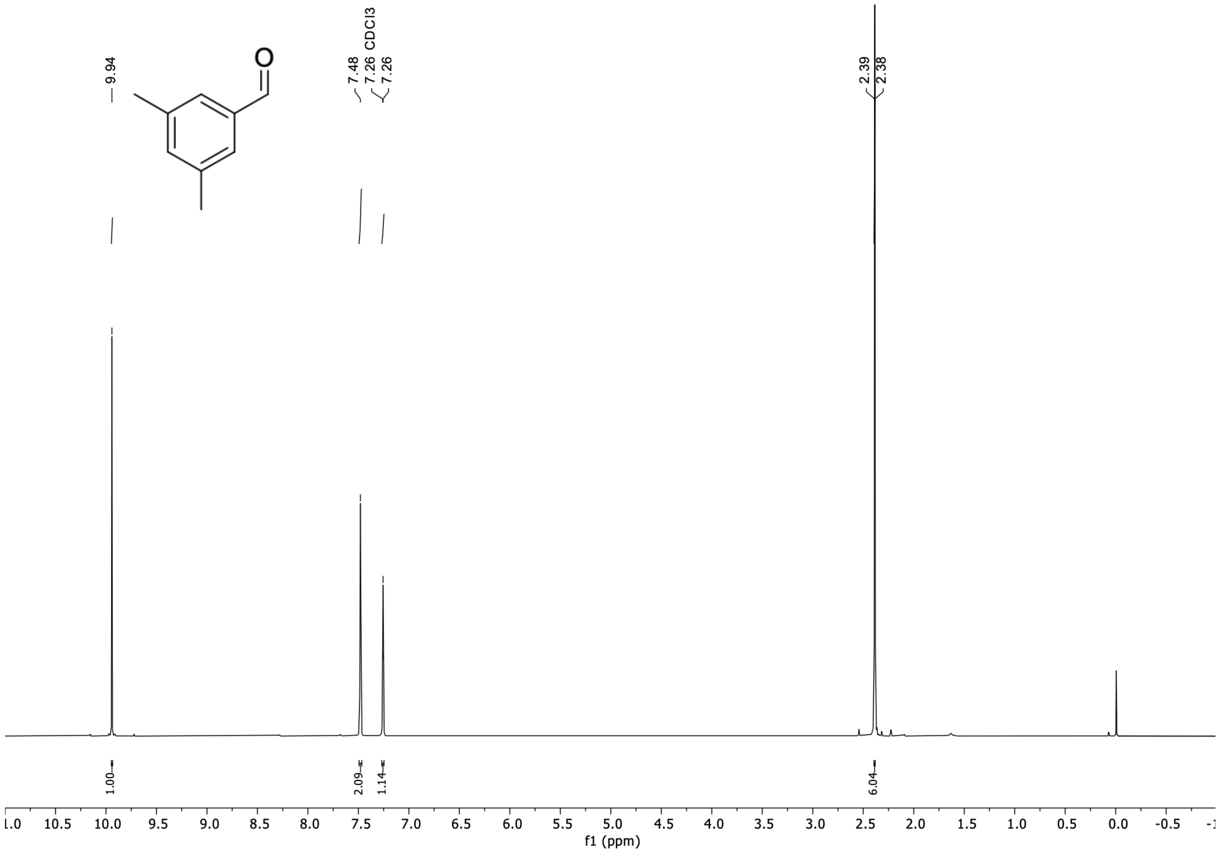

^{13}C{1H}-NMR (101 MHz, CDCl$_3$):

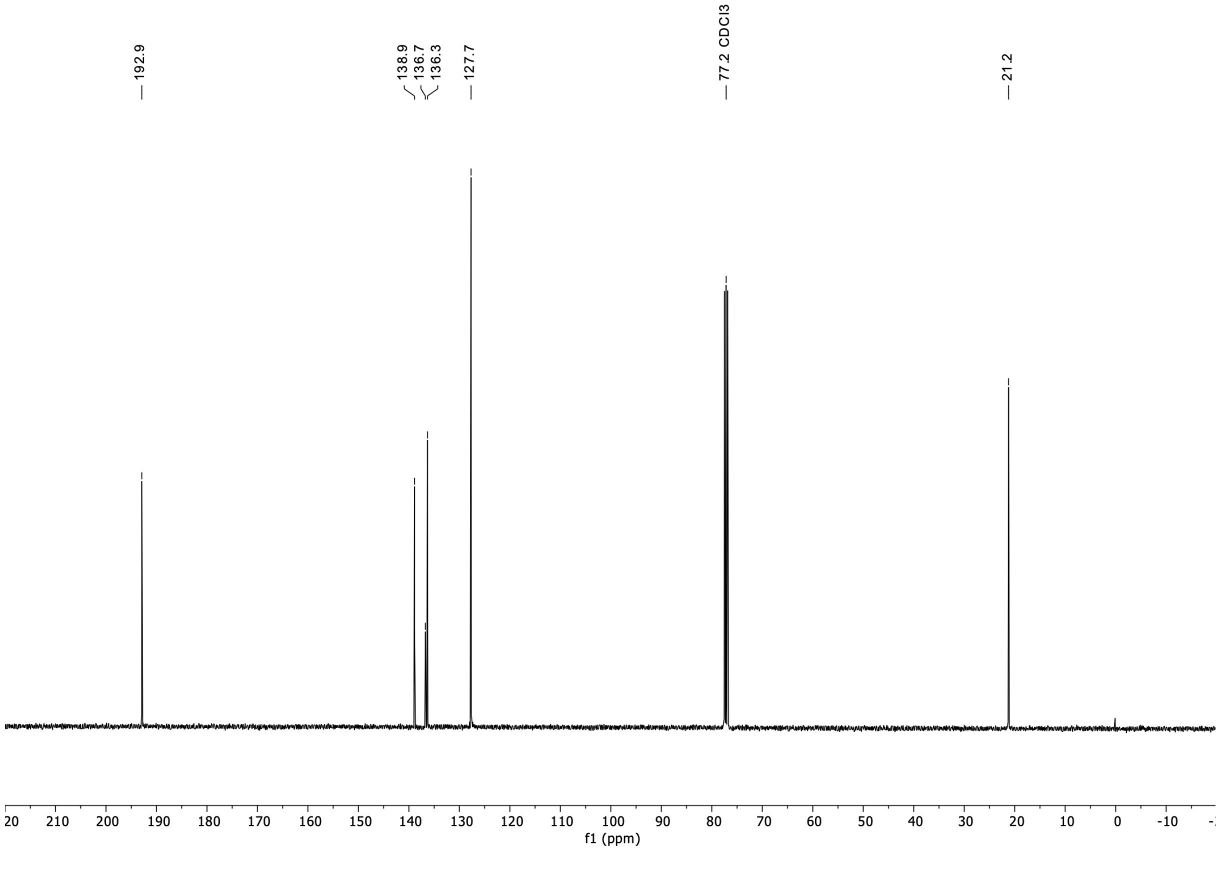

3,5-Diethylbenzaldehyde (**2-Et**)

^{1}H-NMR (400 MHz, CDCl$_3$):

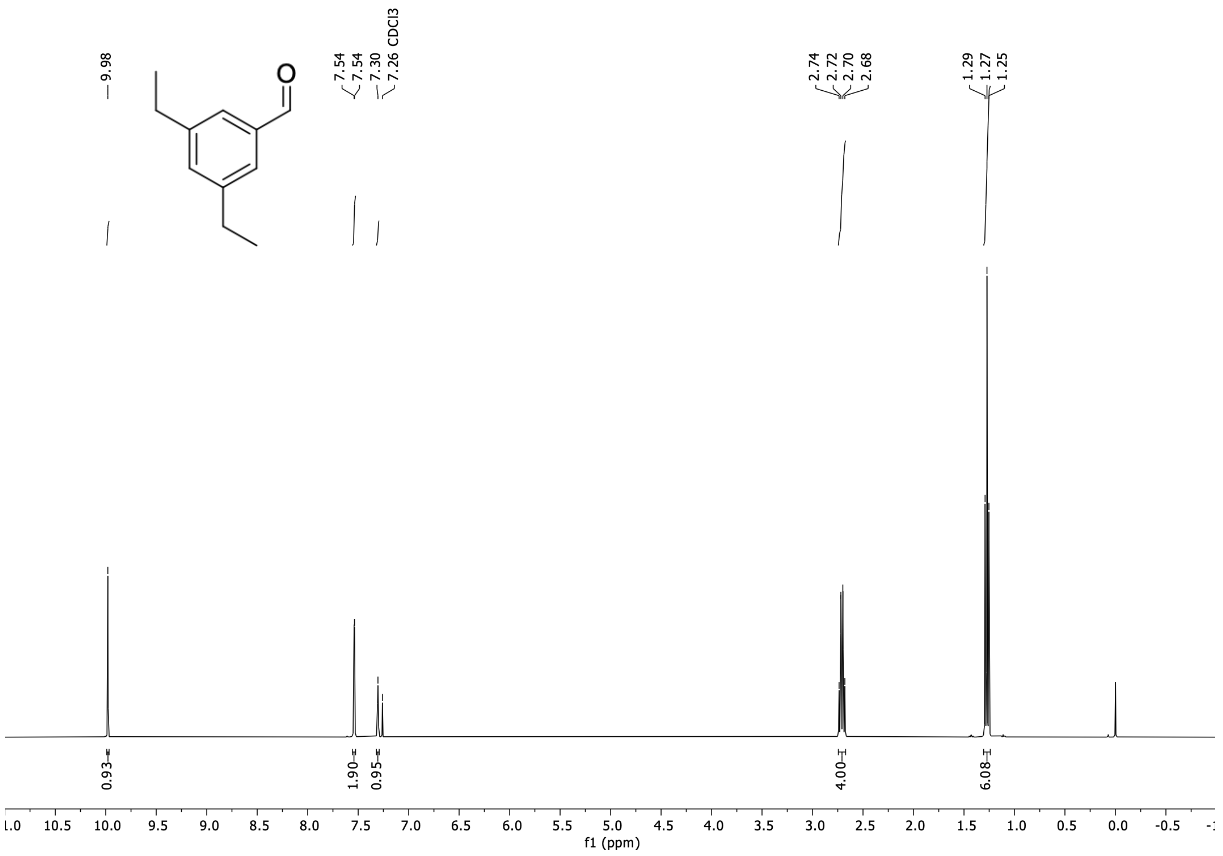

^{13}C{1H}-NMR (101 MHz, CDCl$_3$):

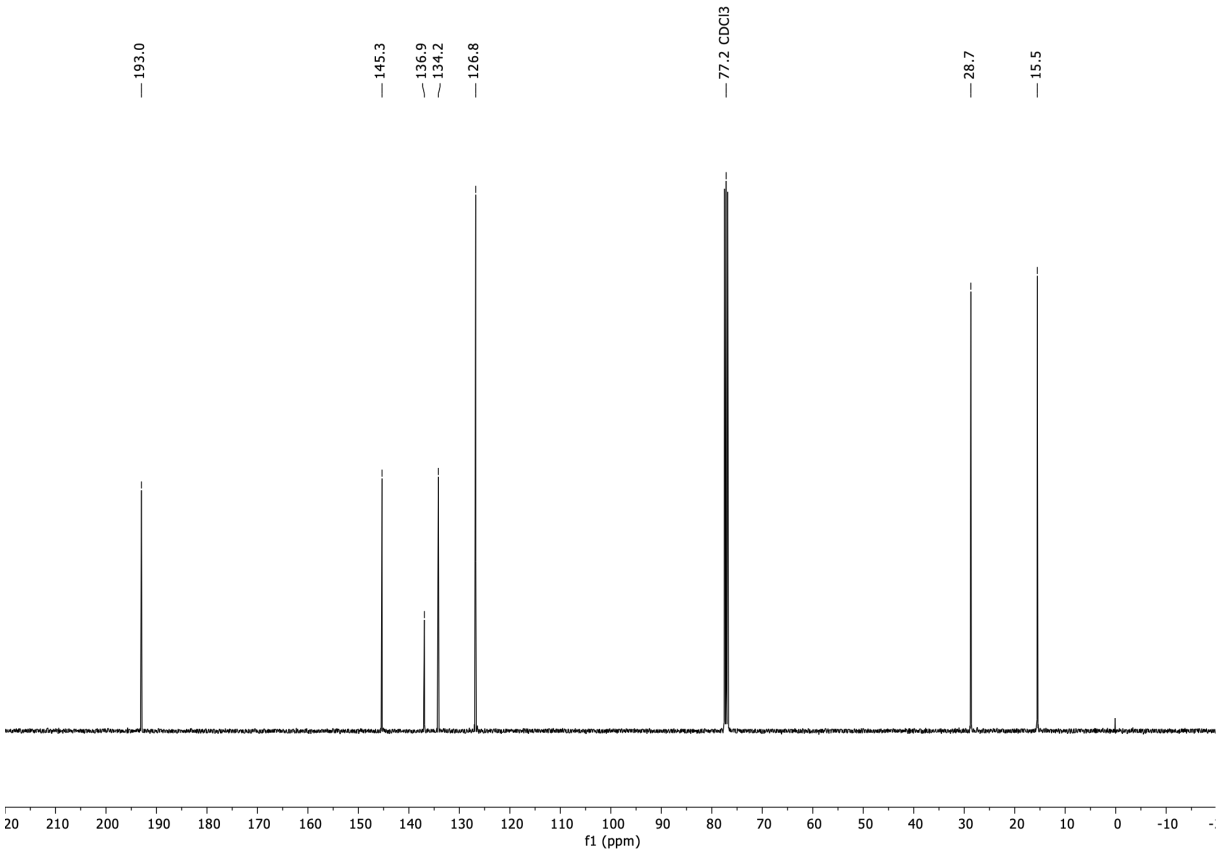

3,5-Di-*iso*-propyl-benzaldehyde (**2-^{i}Pr**)

^{1}H-NMR (400 MHz, $CDCl_3$):

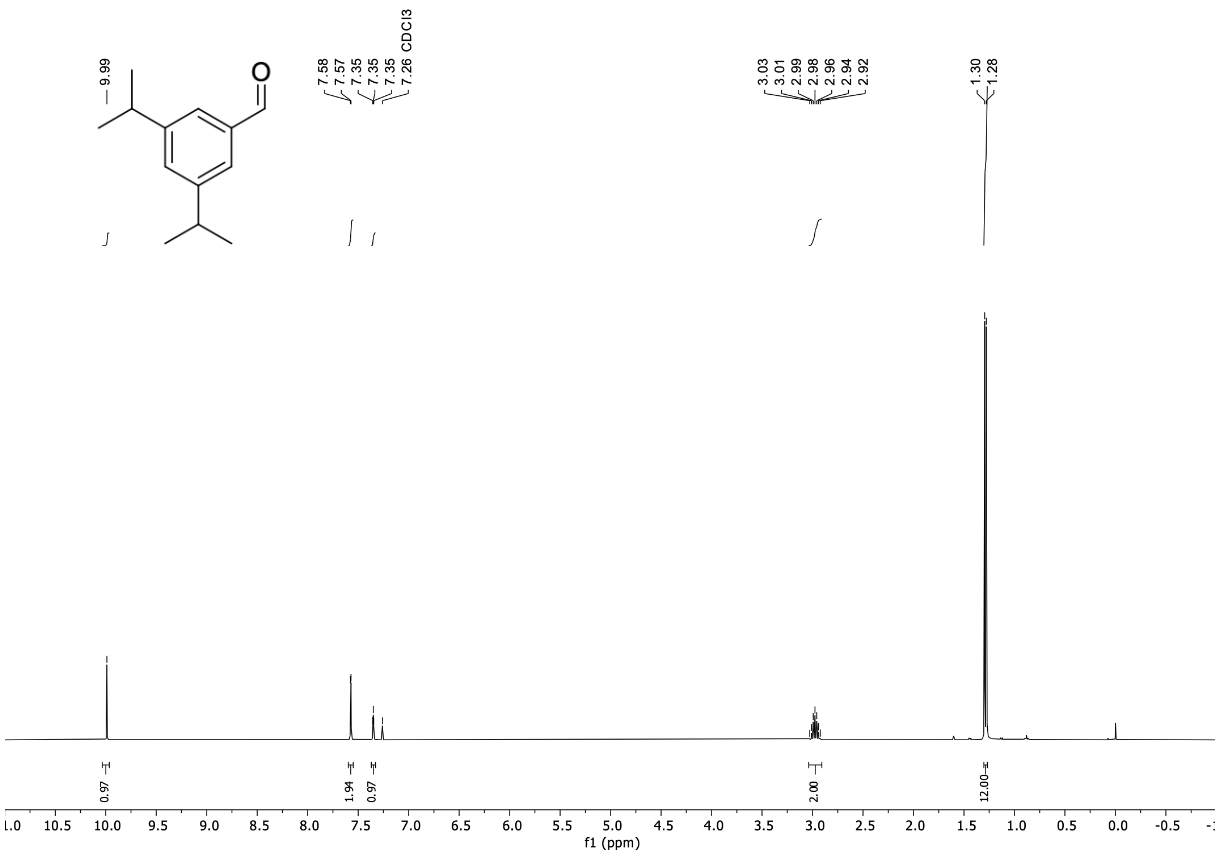

^{13}C{1H}-NMR (101 MHz, $CDCl_3$):

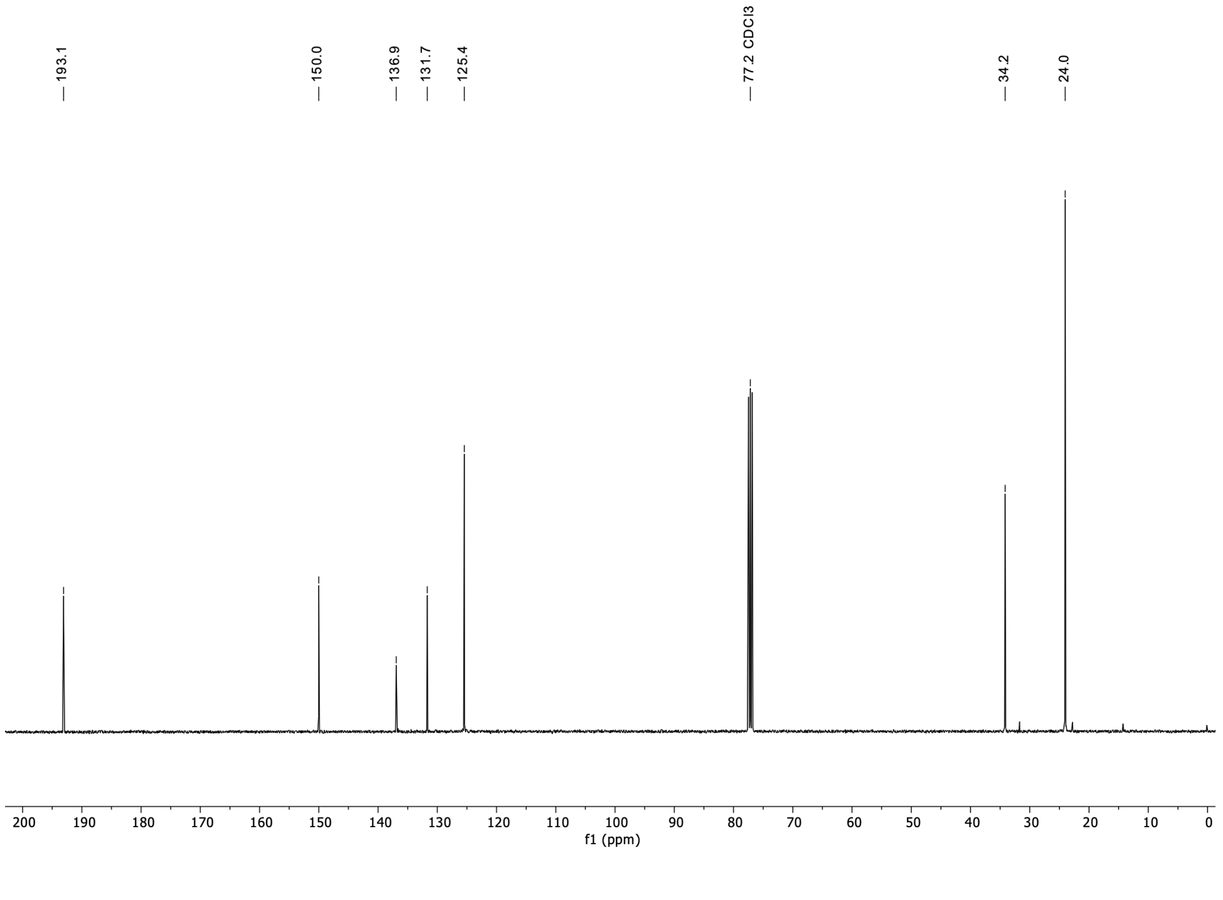

3,5-Di-*tert*-butyl-benzaldehyde (**2-*t*Bu**)

^{1}H-NMR (400 MHz, CDCl$_3$):

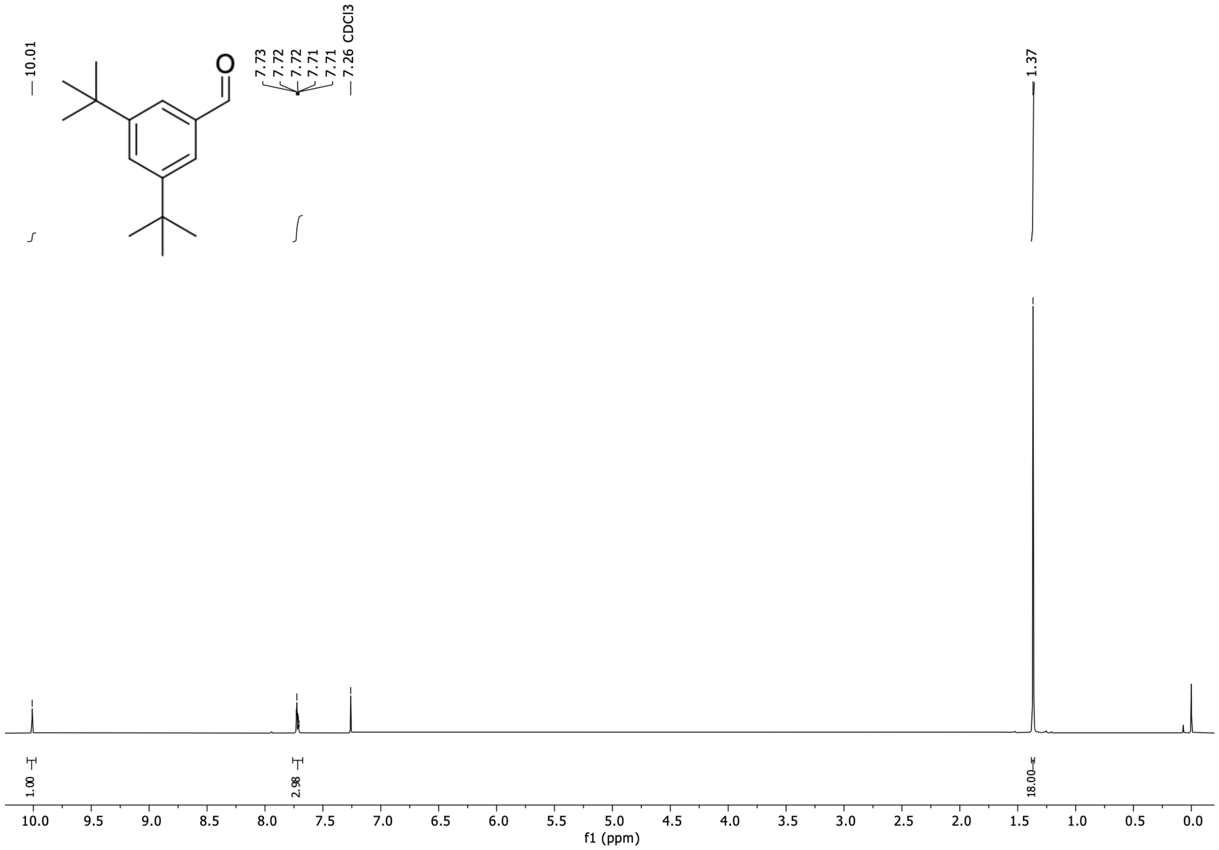

^{13}C{1H}-NMR (101 MHz, CDCl$_3$):

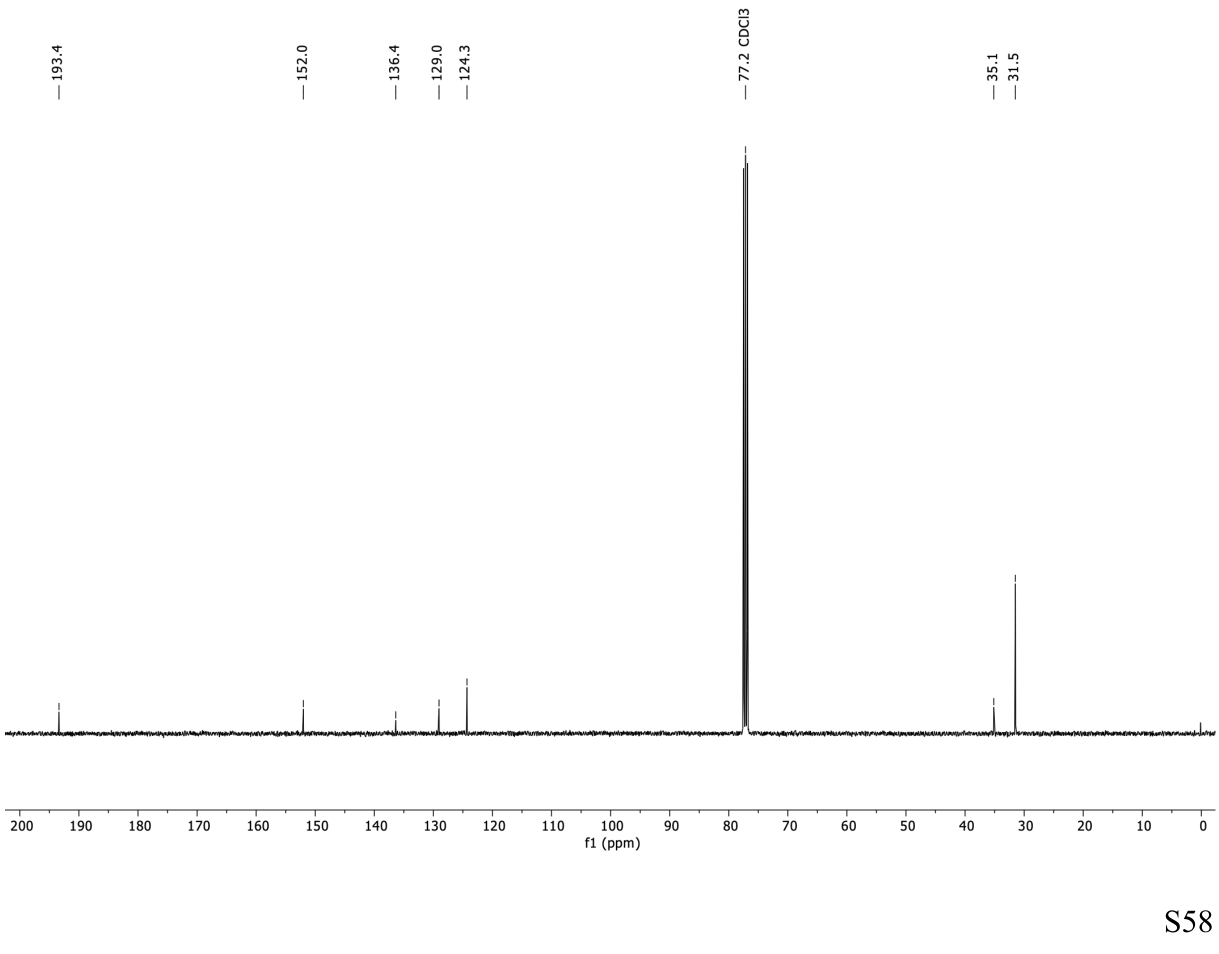

3,5-Dimethylbenzyl alcohol

^{1}H-NMR (400 MHz, $CDCl_3$):

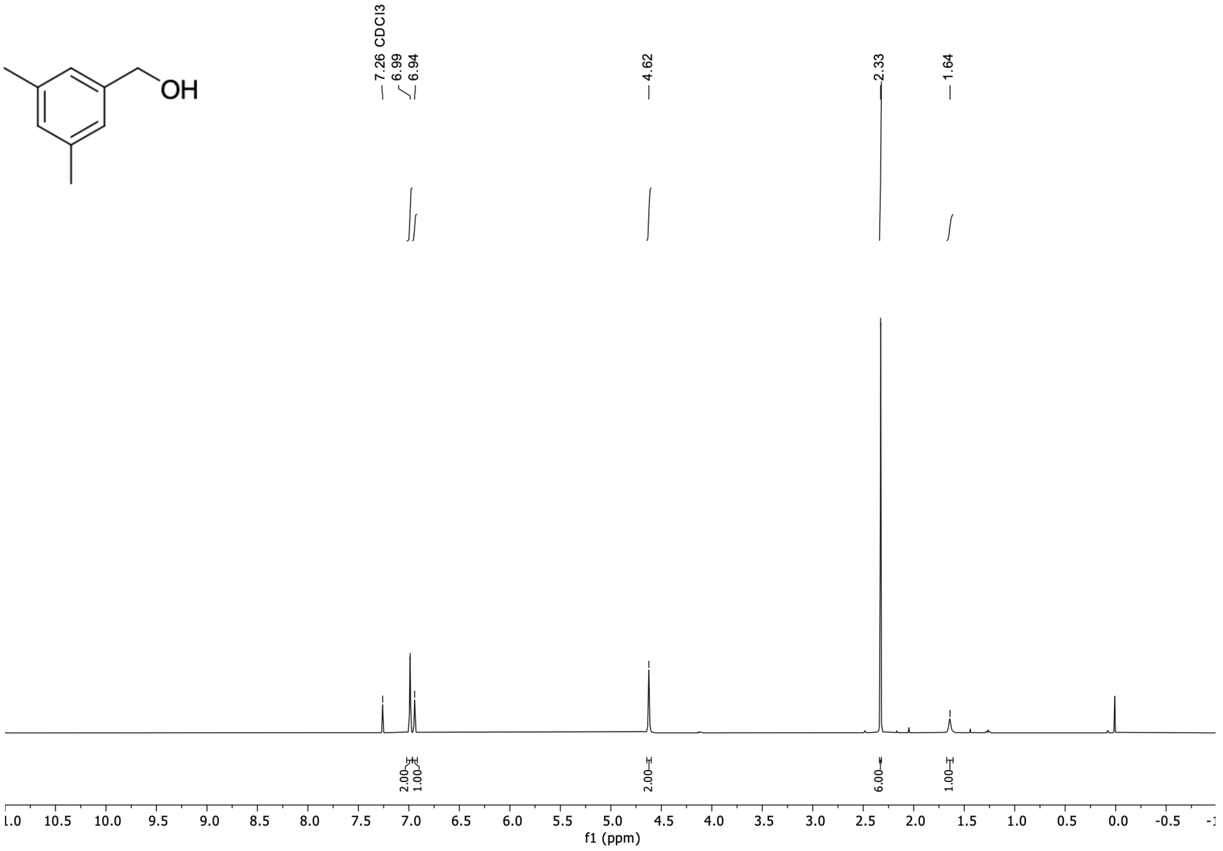

^{13}C{1H}-NMR (101 MHz, $CDCl_3$):

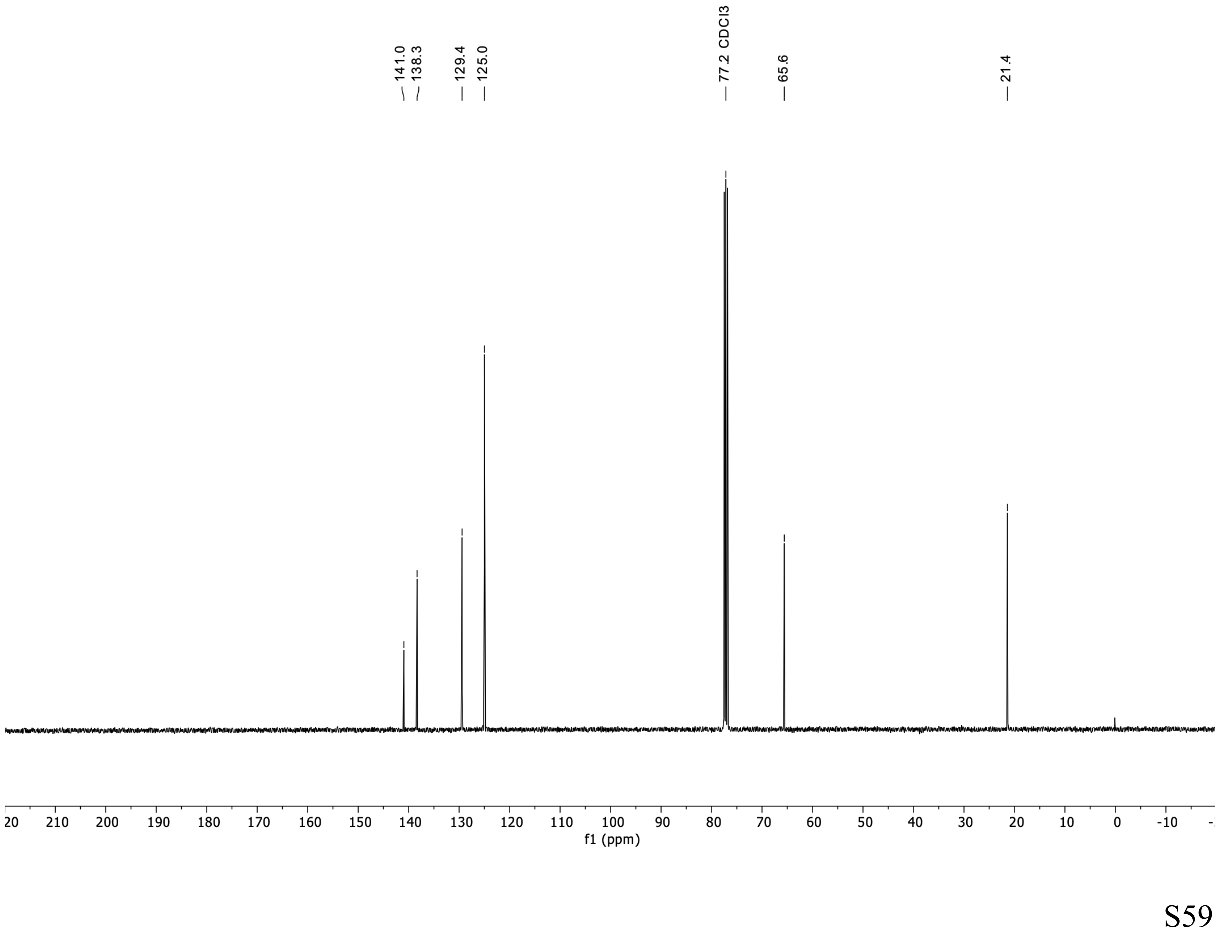

3,5-Diethylbenzyl alcohol

^{1}H-NMR (400 MHz, $CDCl_3$):

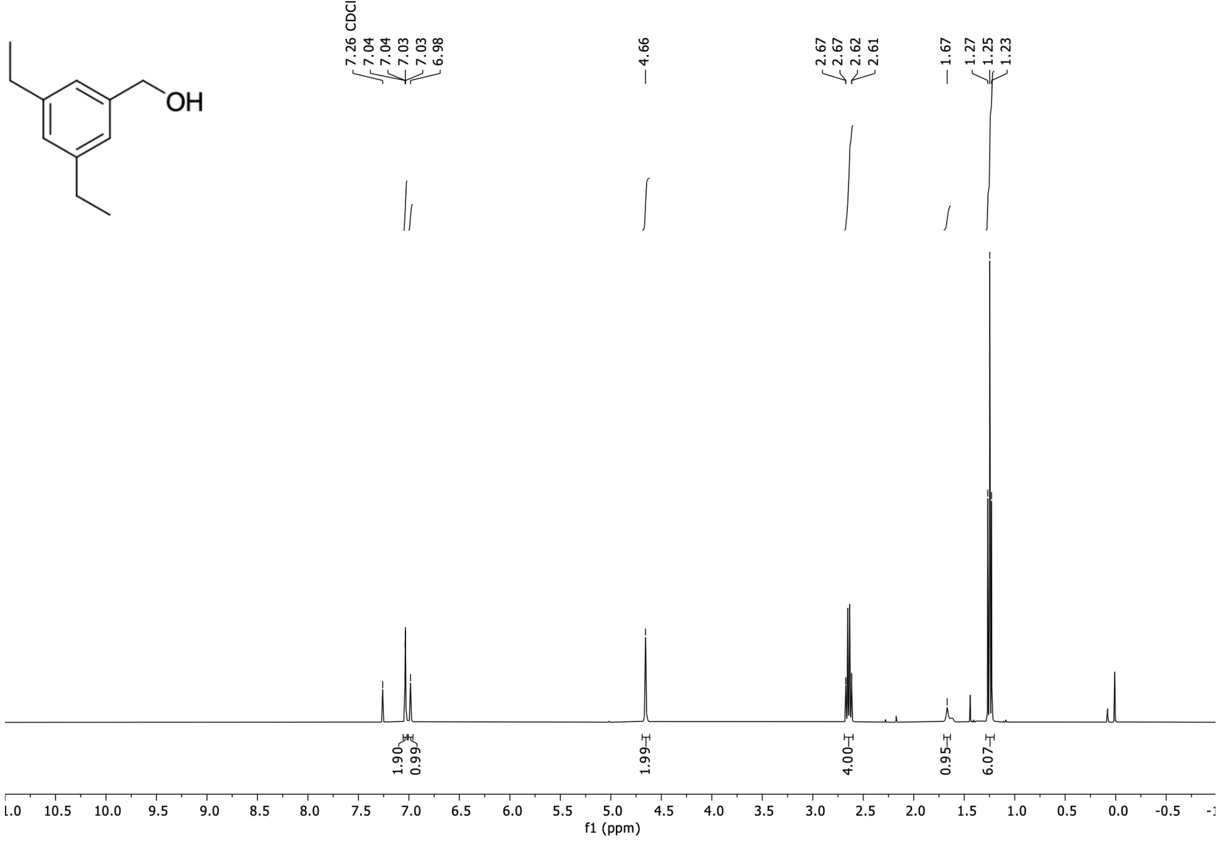

^{13}C{1H}-NMR (101 MHz, $CDCl_3$):

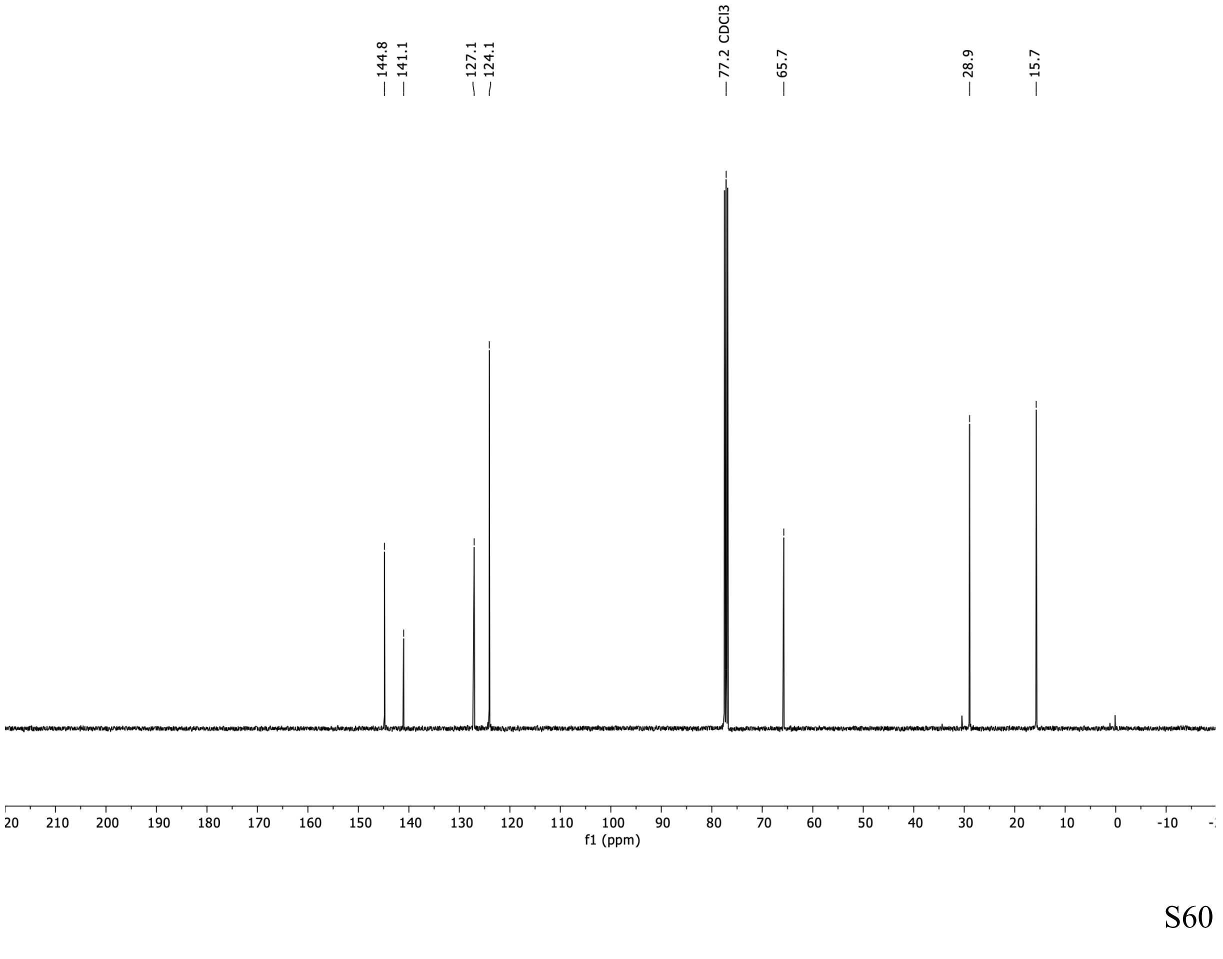

3,5-Di-*iso*-propylbenzyl alcohol

^{1}H-NMR (400 MHz, $CDCl_3$):

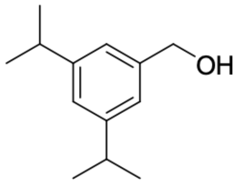

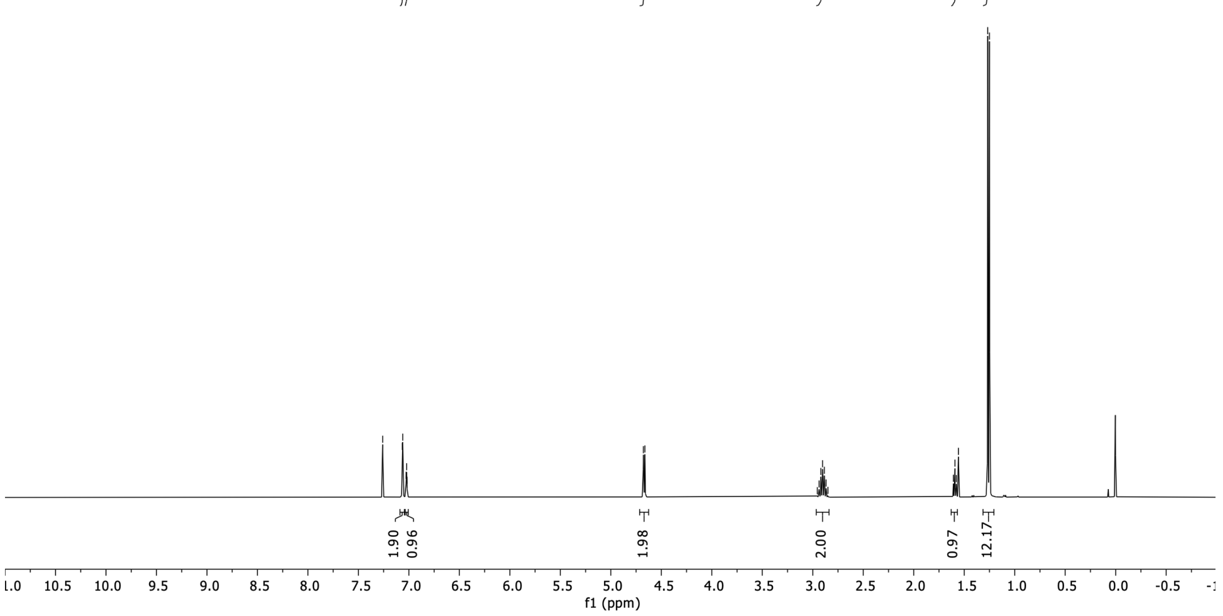

^{13}C{1H}-NMR (101 MHz, $CDCl_3$):

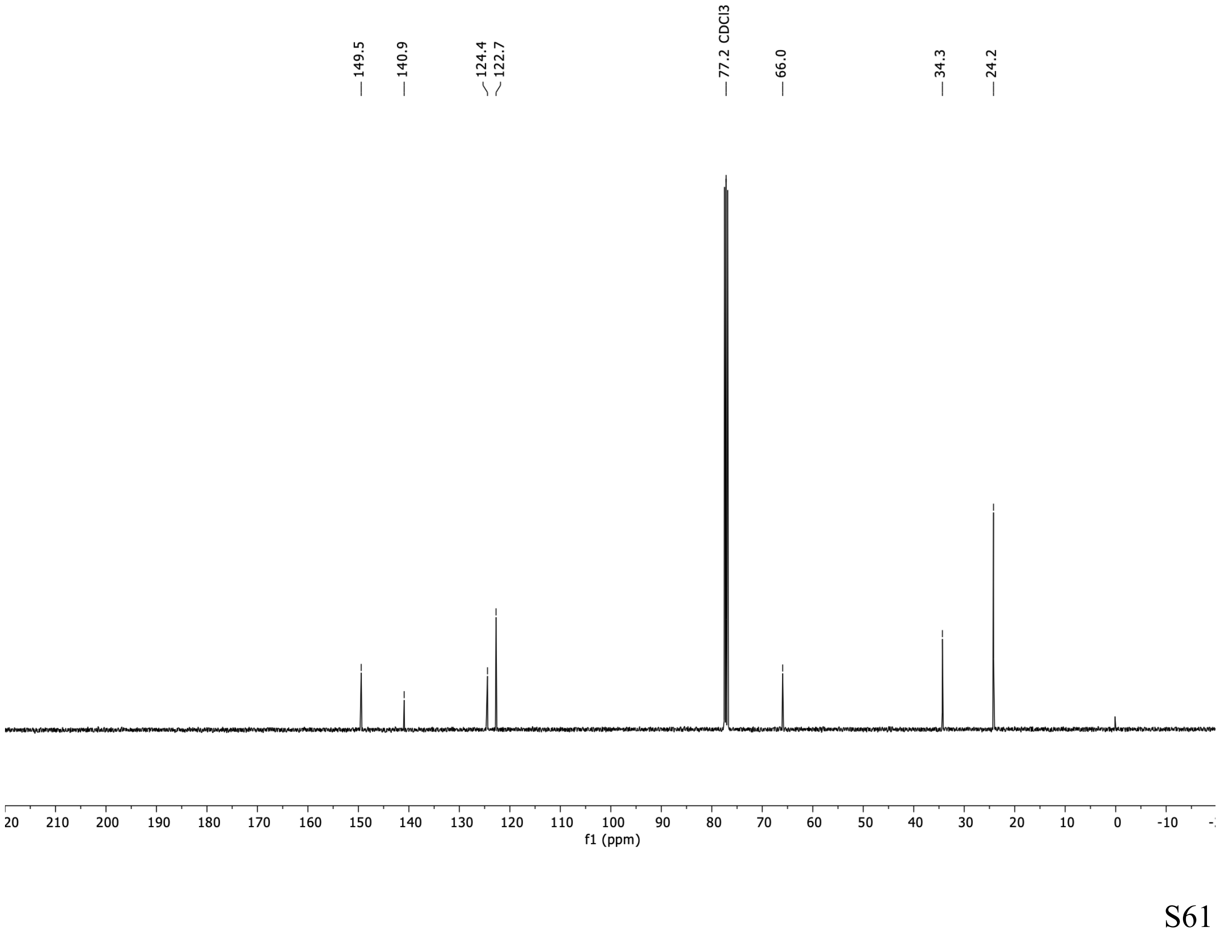

3,5-Di-*tert*-butylbenzyl alcohol

^{1}H-NMR (400 MHz, $CDCl_3$):

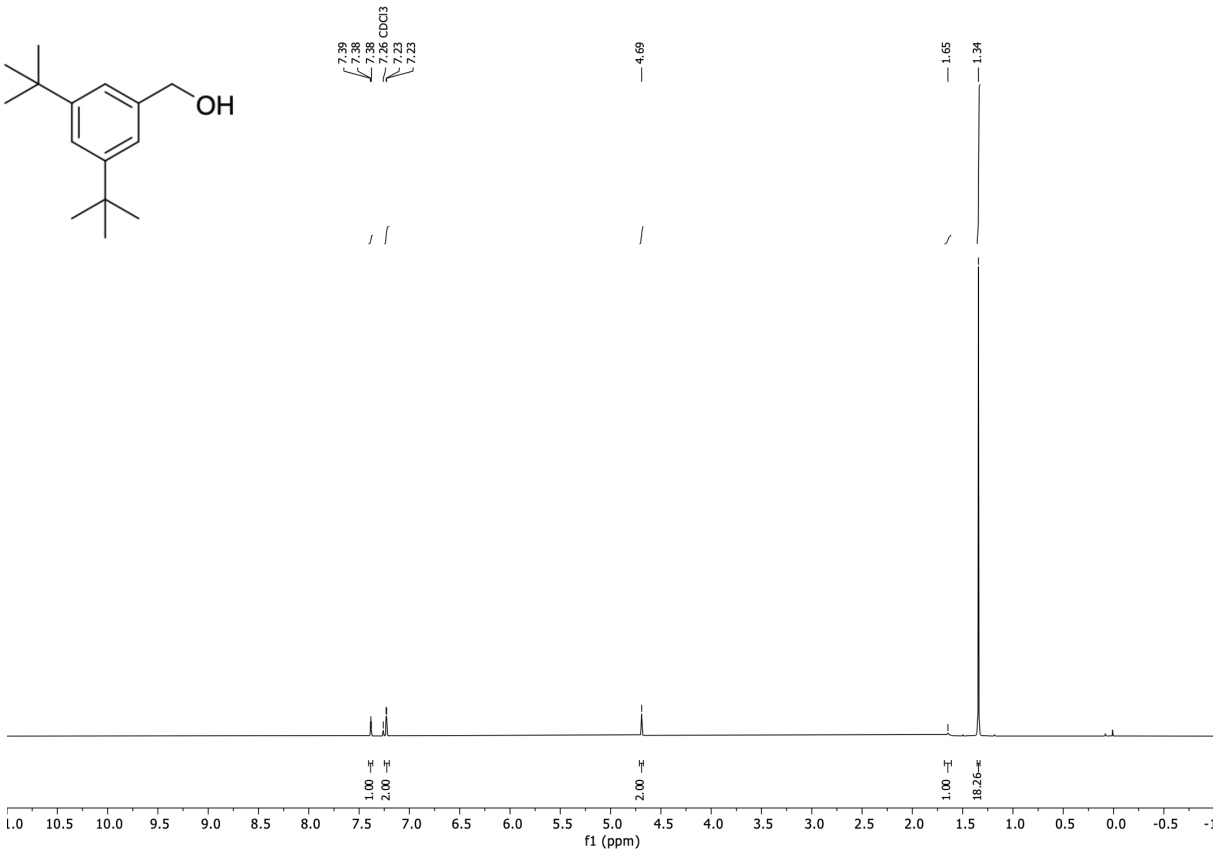

^{13}C{1H}-NMR (101 MHz, $CDCl_3$):

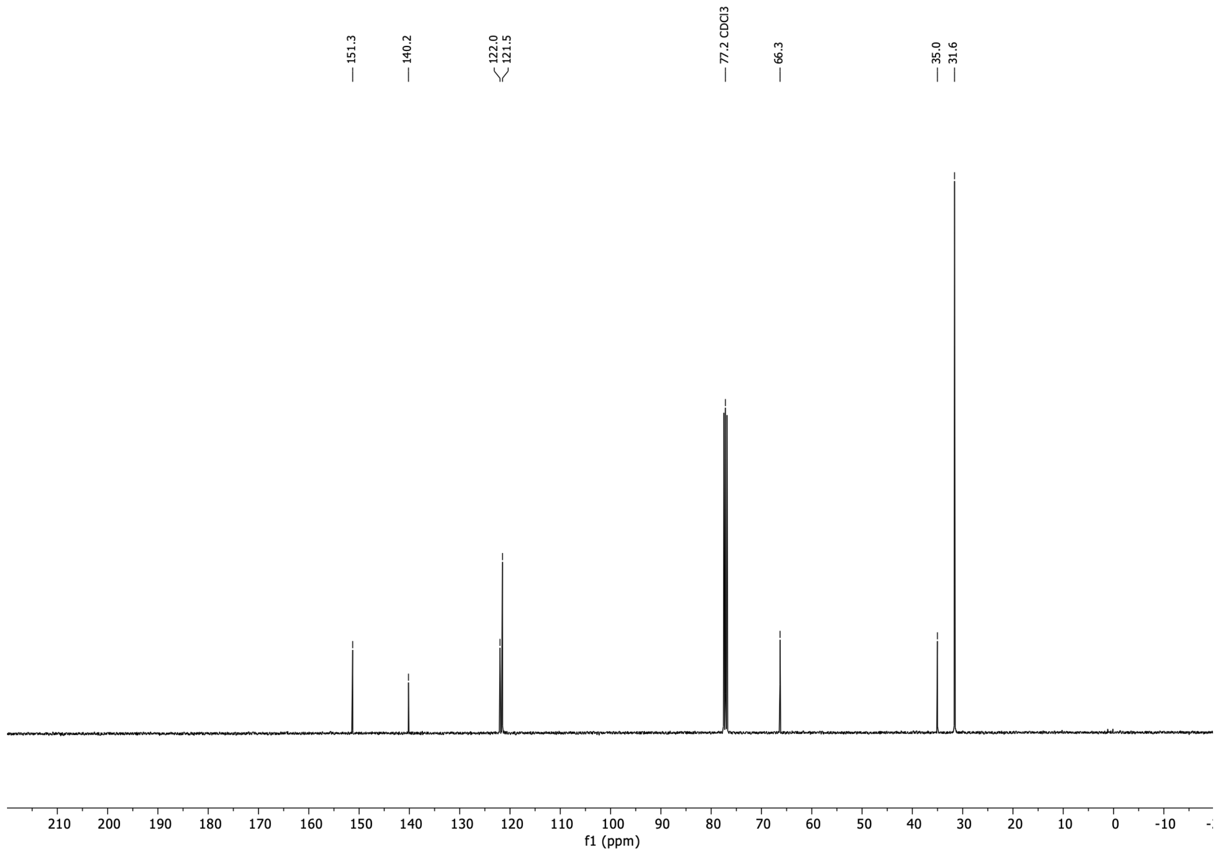

3,5-Dimethylbenzyl bromide

¹H-NMR (400 MHz, $CDCl_3$):

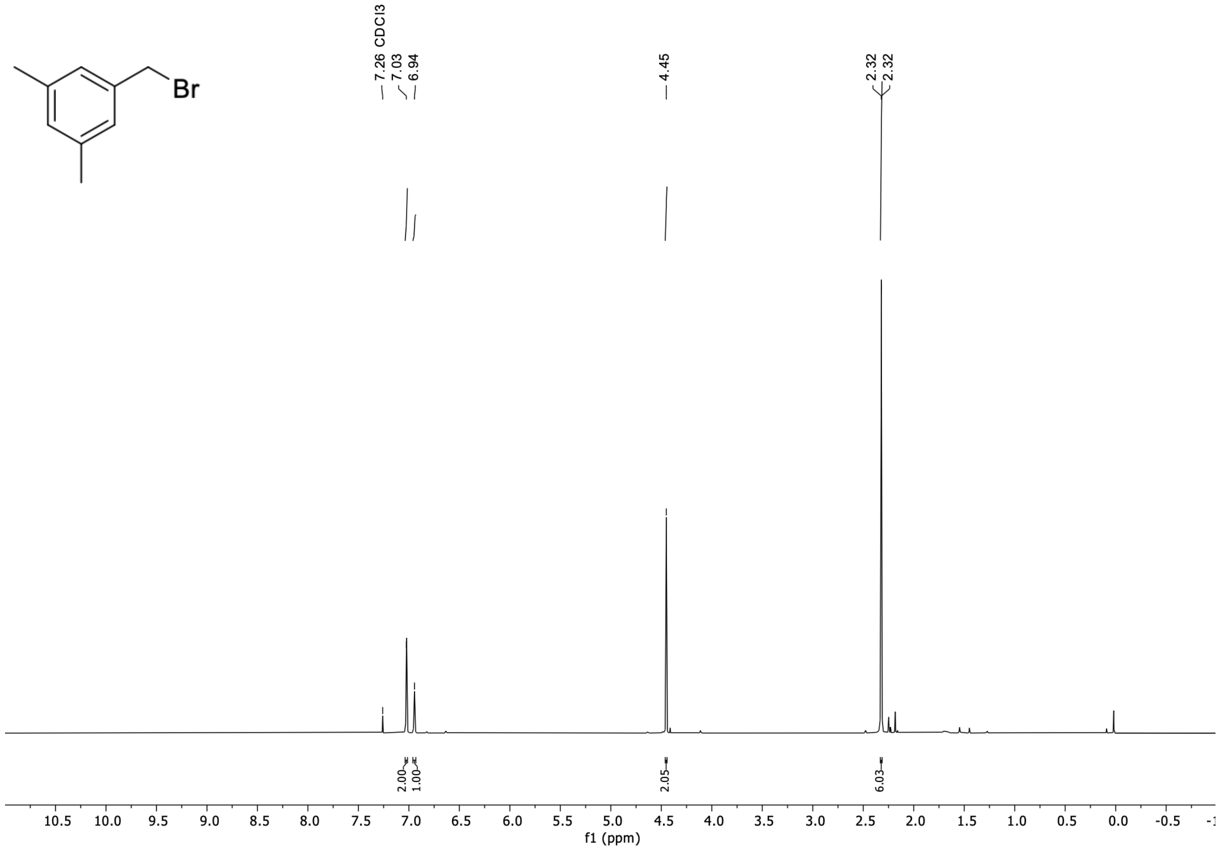

¹³C{1H}-NMR (101 MHz, $CDCl_3$):

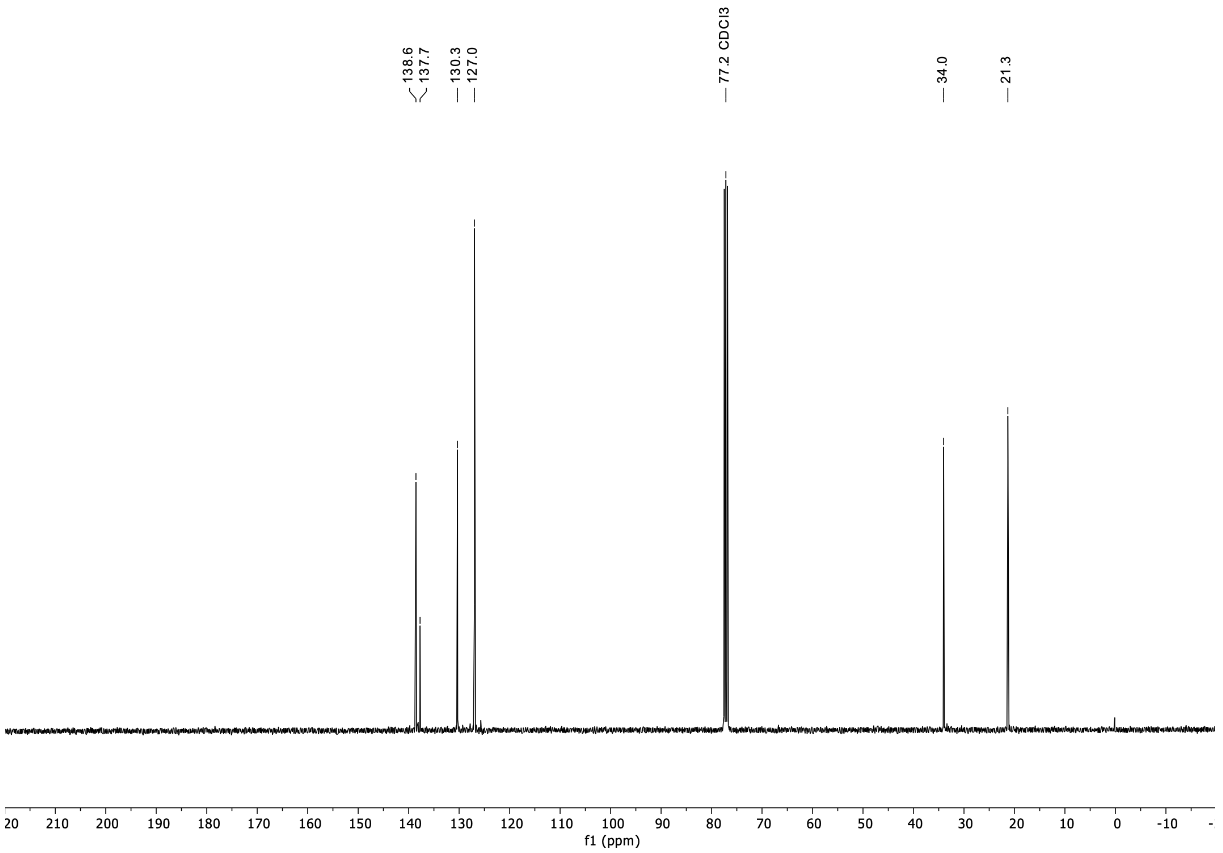

3,5-Diethylbenzyl bromide

^{1}H-NMR (400 MHz, CDCl$_3$):

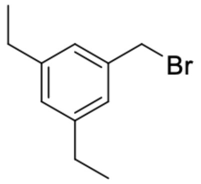

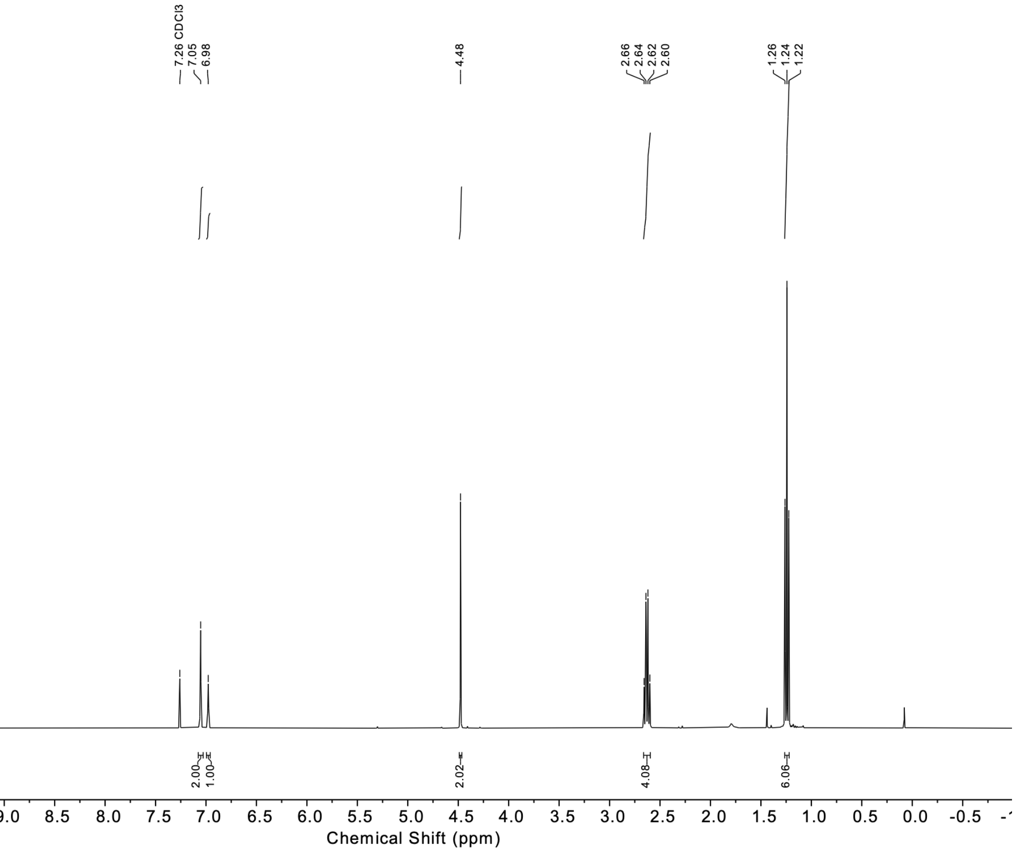

^{13}C{1H}-NMR (101 MHz, CDCl$_3$):

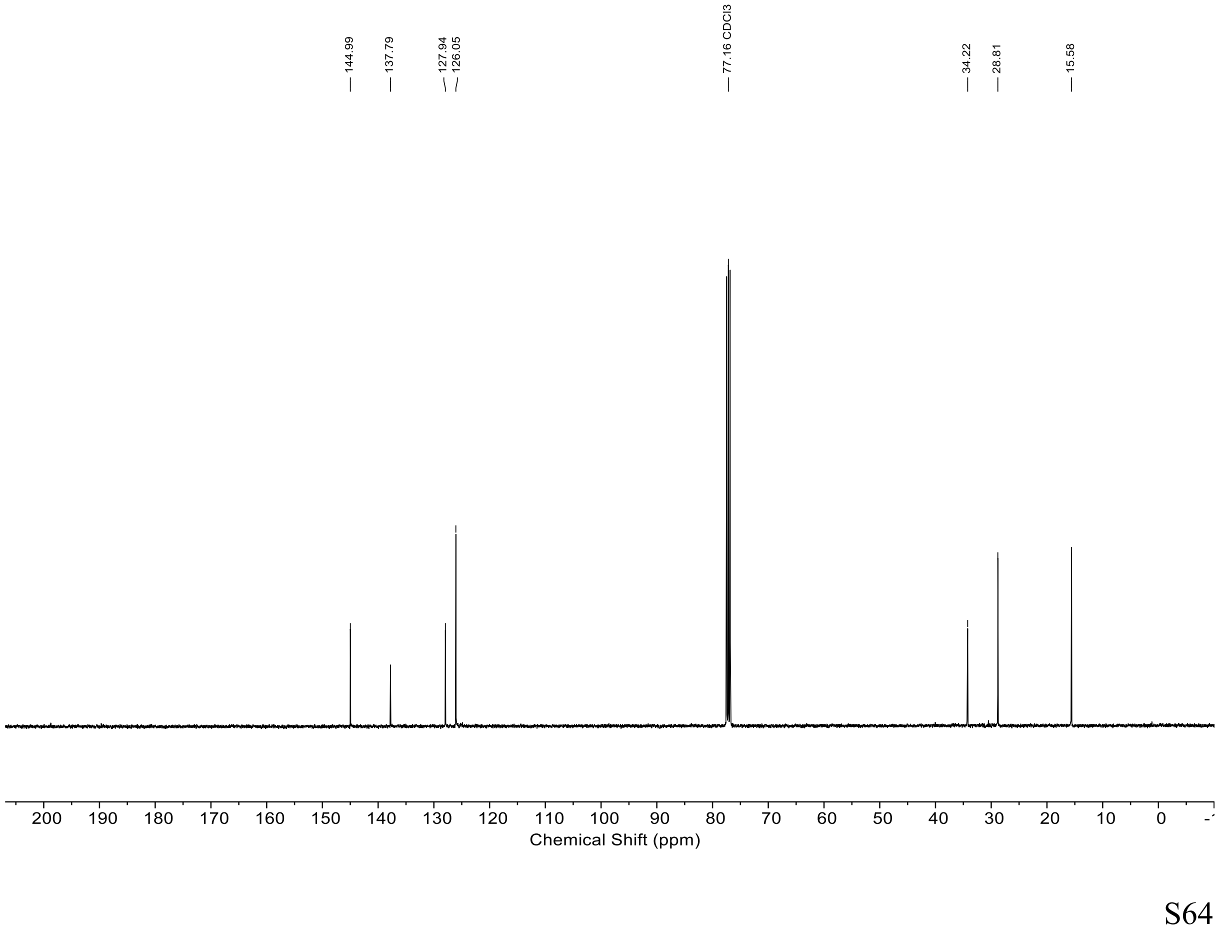

3,5-Di-*iso*-propylbenzyl bromide

^{1}H-NMR (400 MHz, $CDCl_3$):

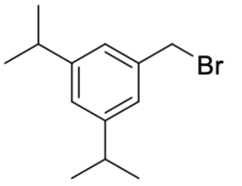

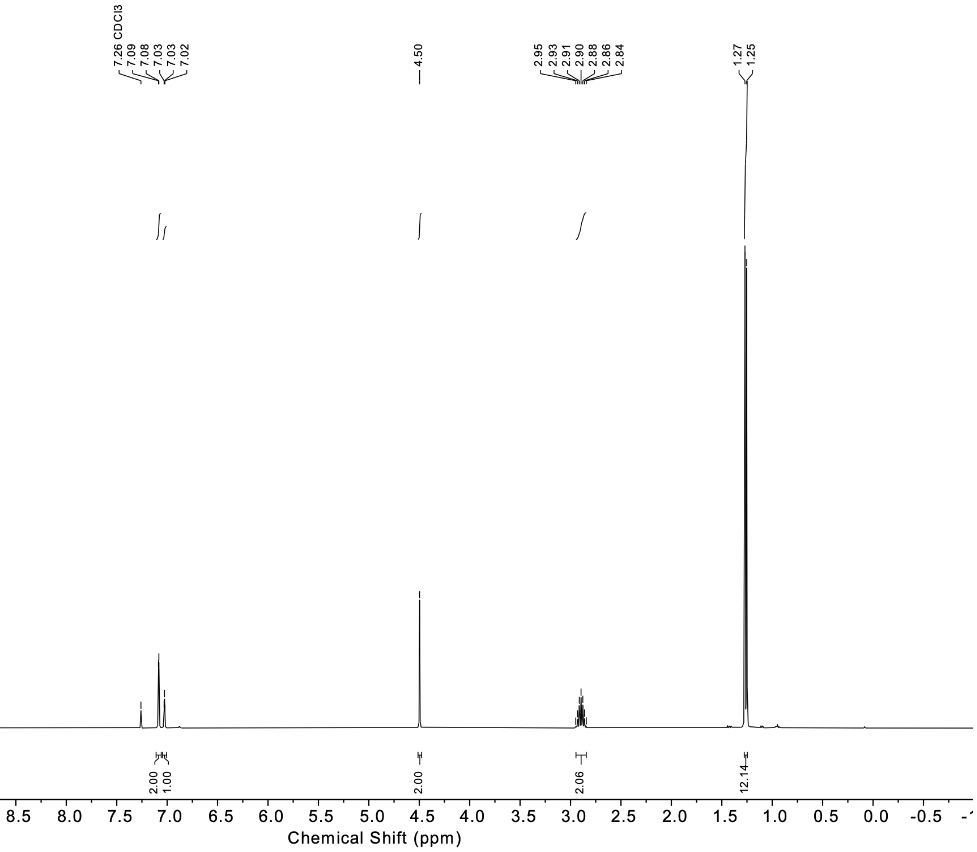

^{13}C{1H}-NMR (101 MHz, $CDCl_3$):

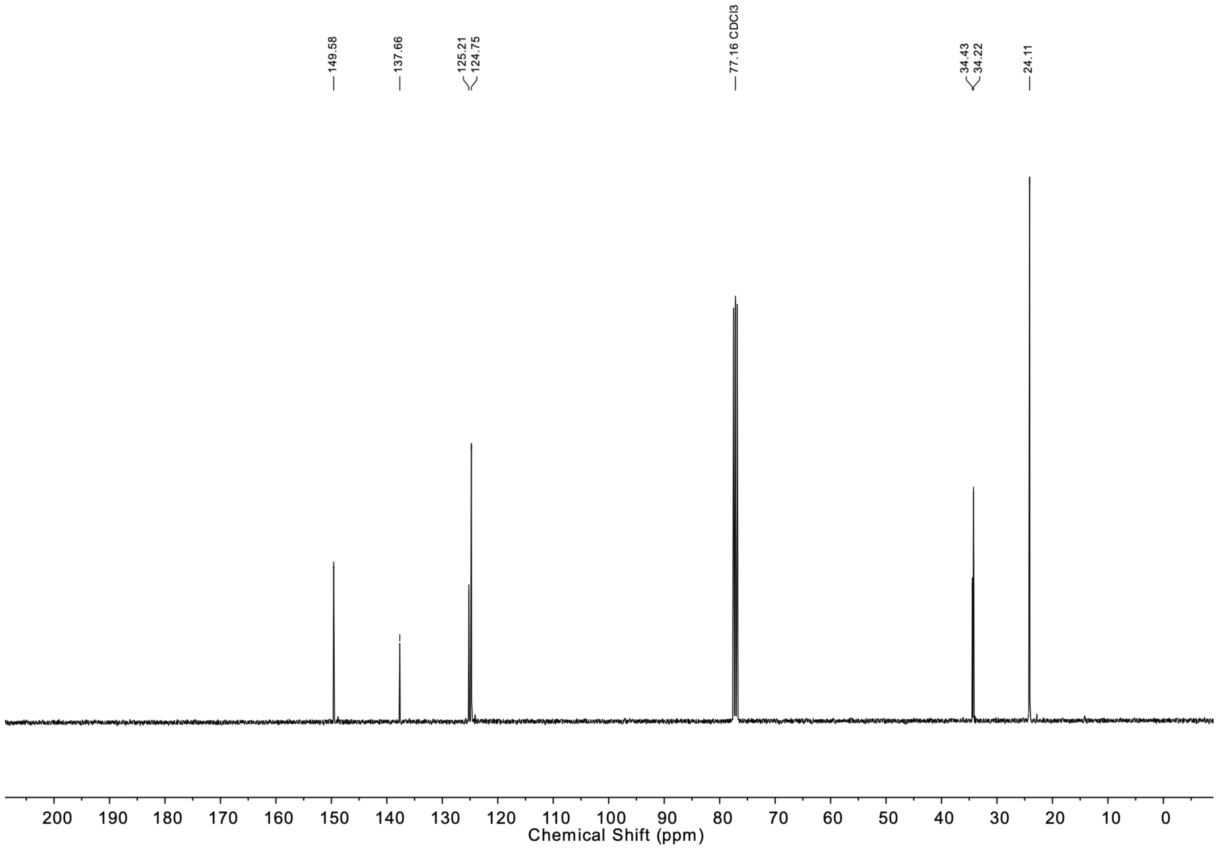

3,5-Di-*tert*-butylbenzyl bromide

^{1}H-NMR (400 MHz, $CDCl_3$):

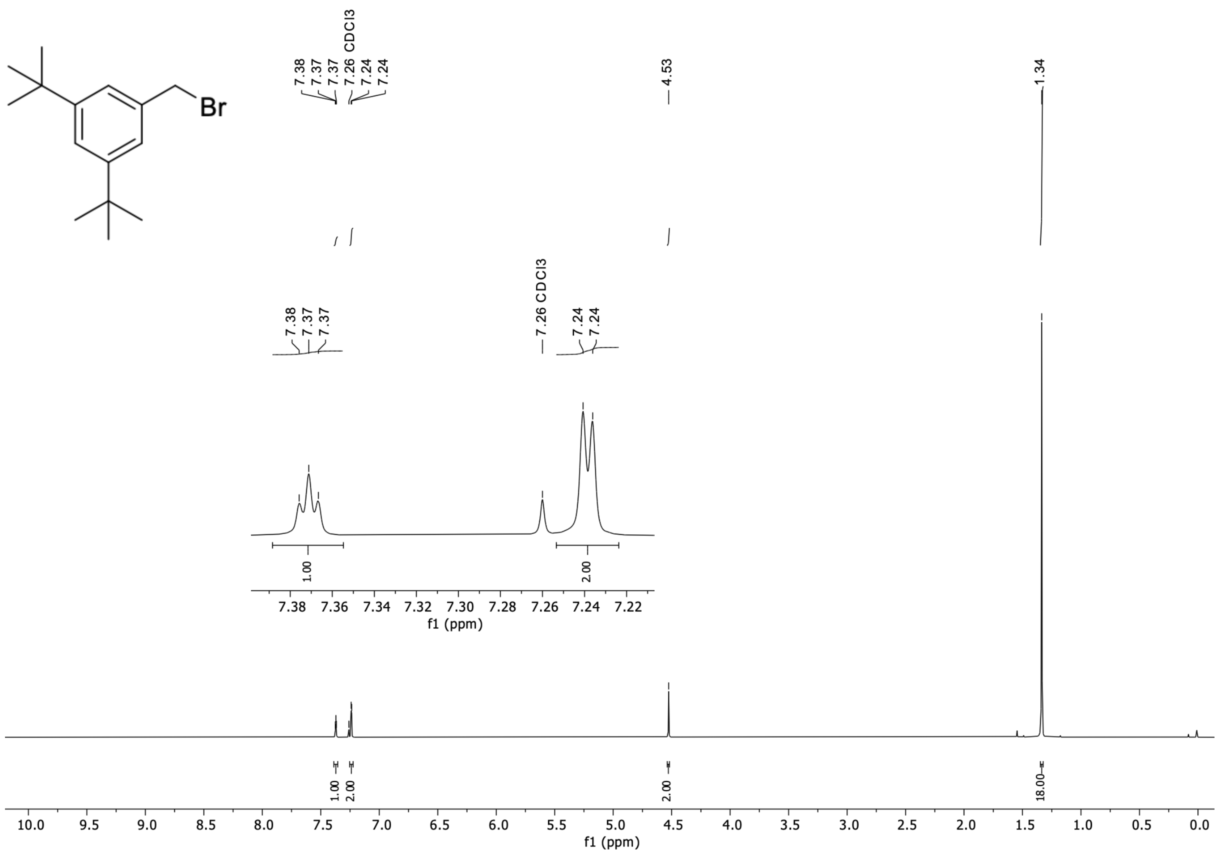

$^{13}C\{1H\}$-NMR (101 MHz, $CDCl_3$):

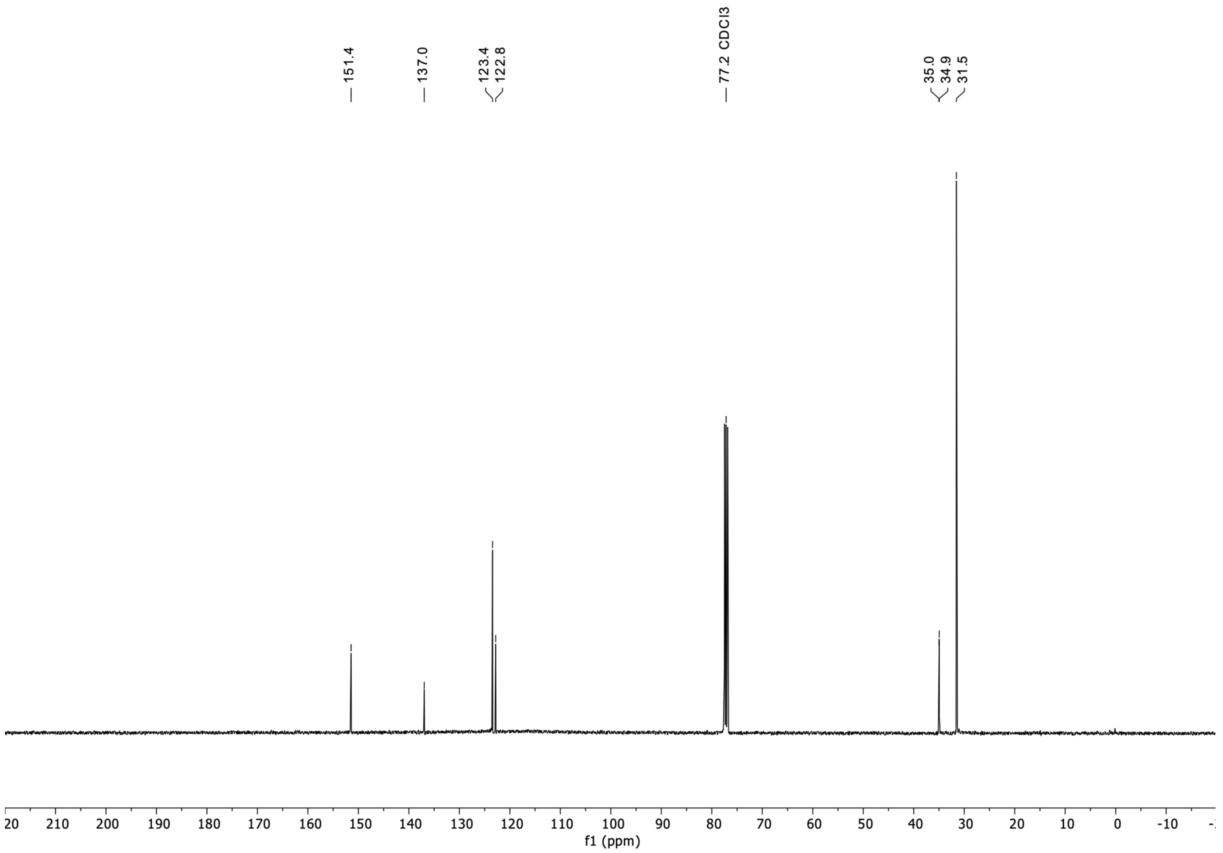

1-[(Phenyl)methyl] tetrahydrothiophenium tetrafluoroborate (**1-H**)

^{1}H-NMR (400 MHz, $CDCl_3$):

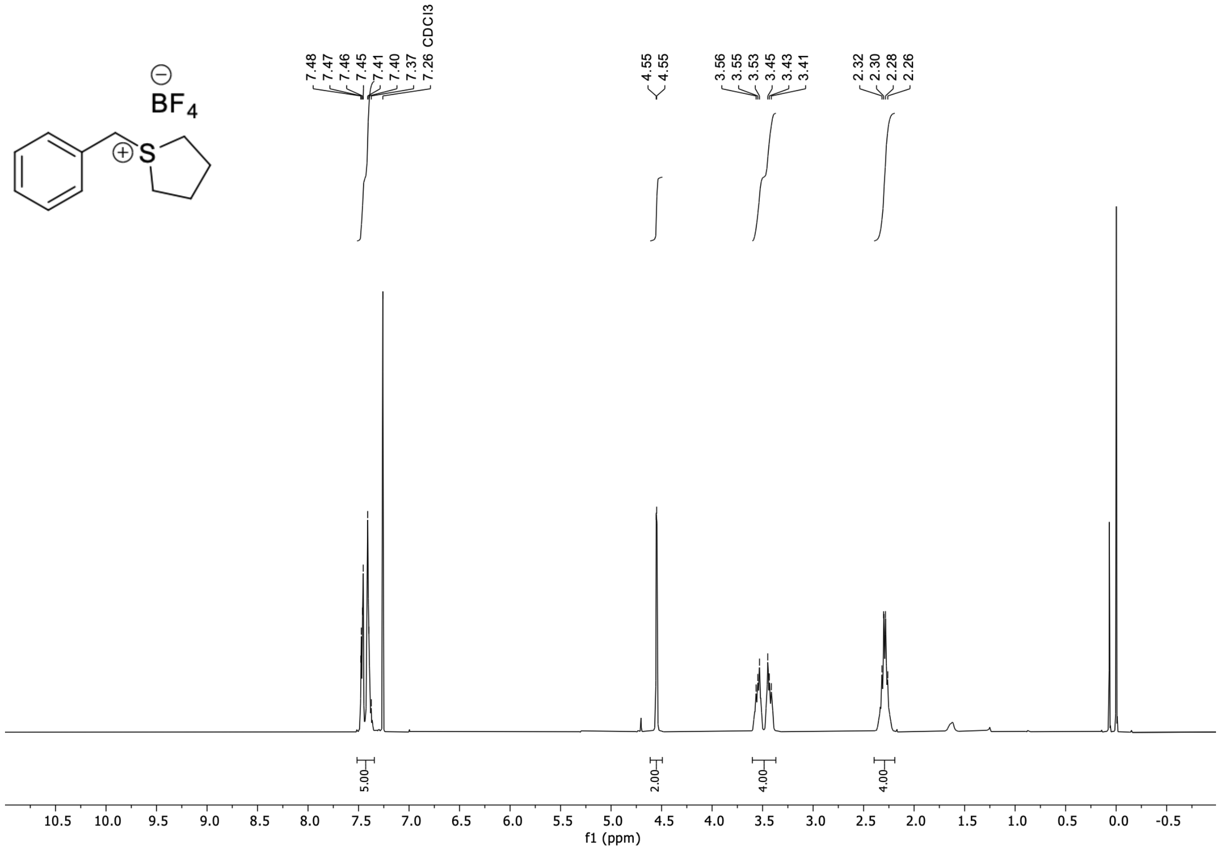

^{13}C{1H}-NMR (101 MHz, $CDCl_3$):

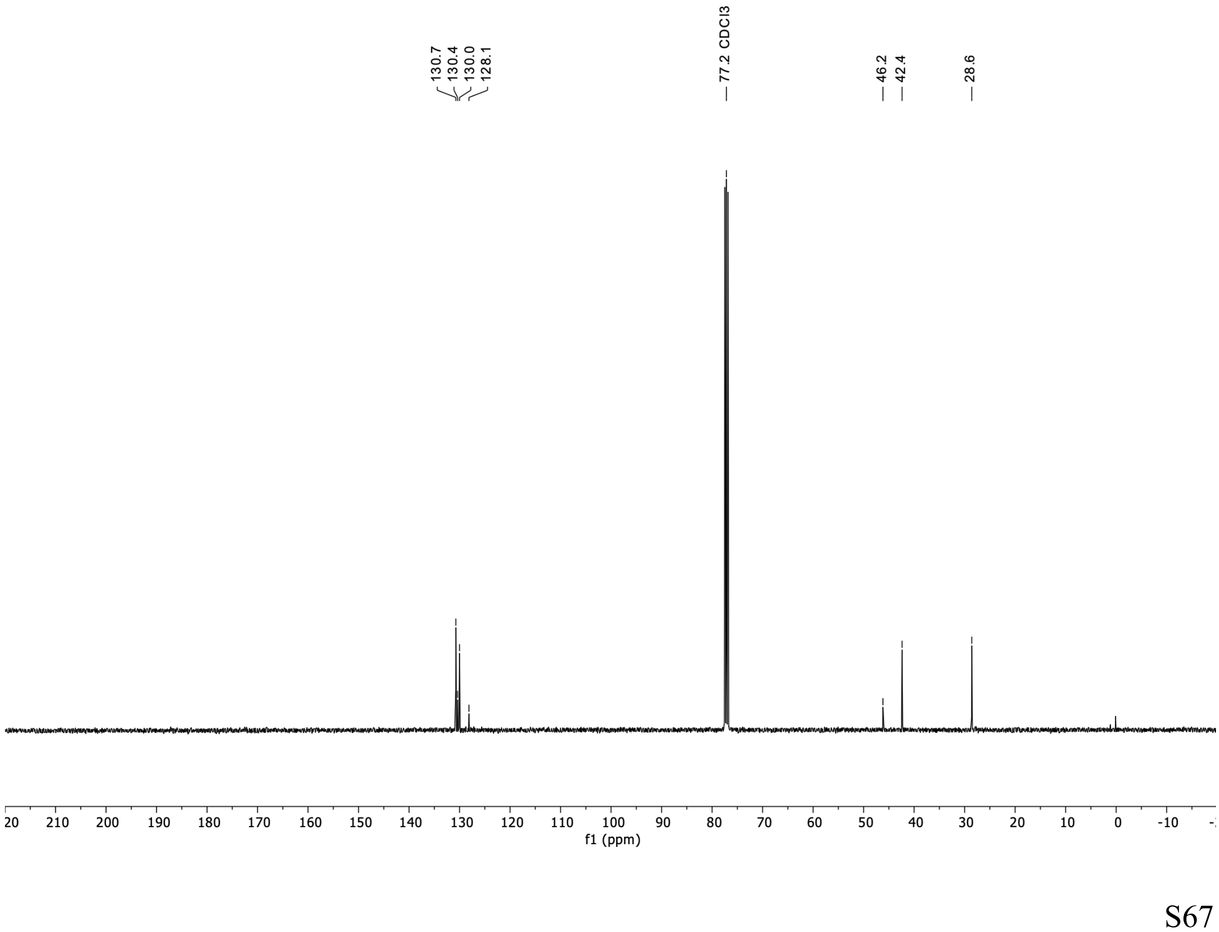

1-[(3,5-Dimethylphenyl)methyl] tetrahydrothiophenium tetrafluoroborate (**1-Me**)

^{1}H-NMR (400 MHz, $CDCl_3$):

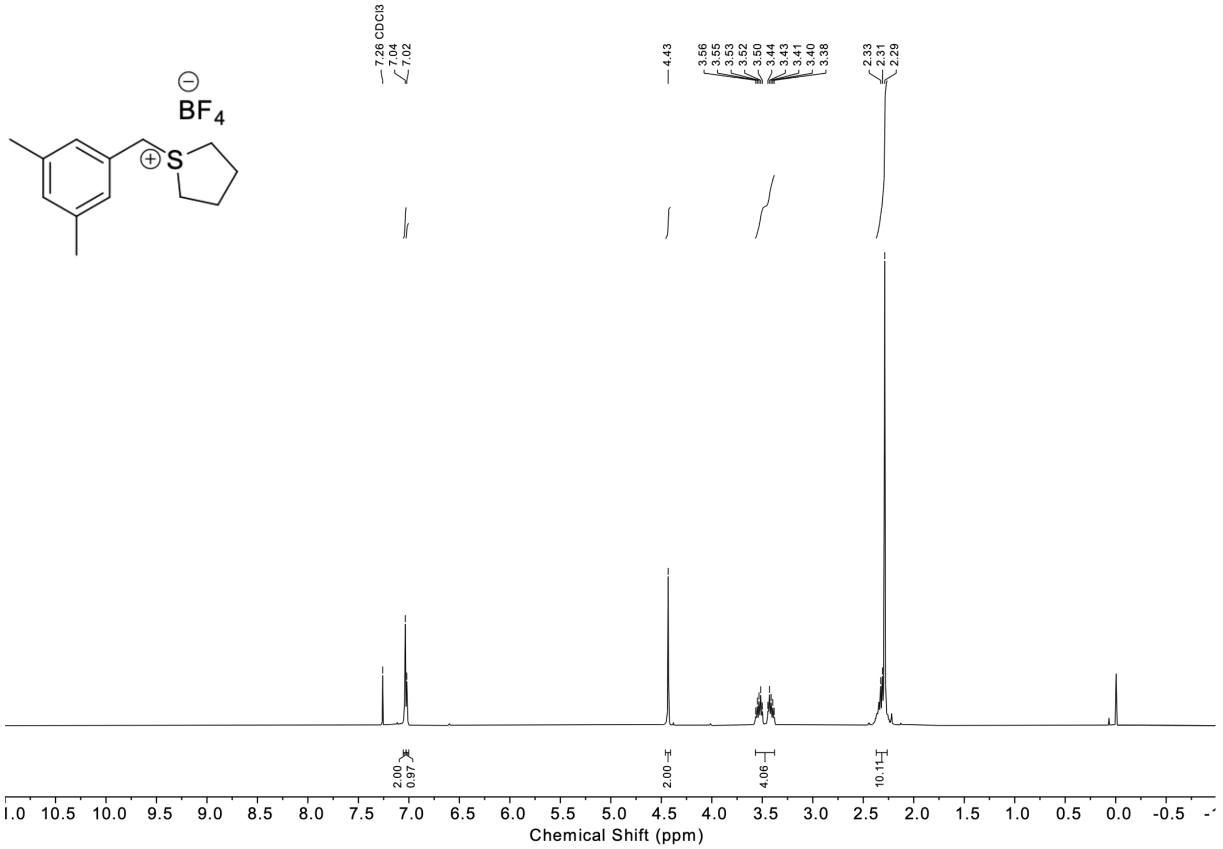

^{13}C{1H}-NMR (101 MHz, $CDCl_3$):

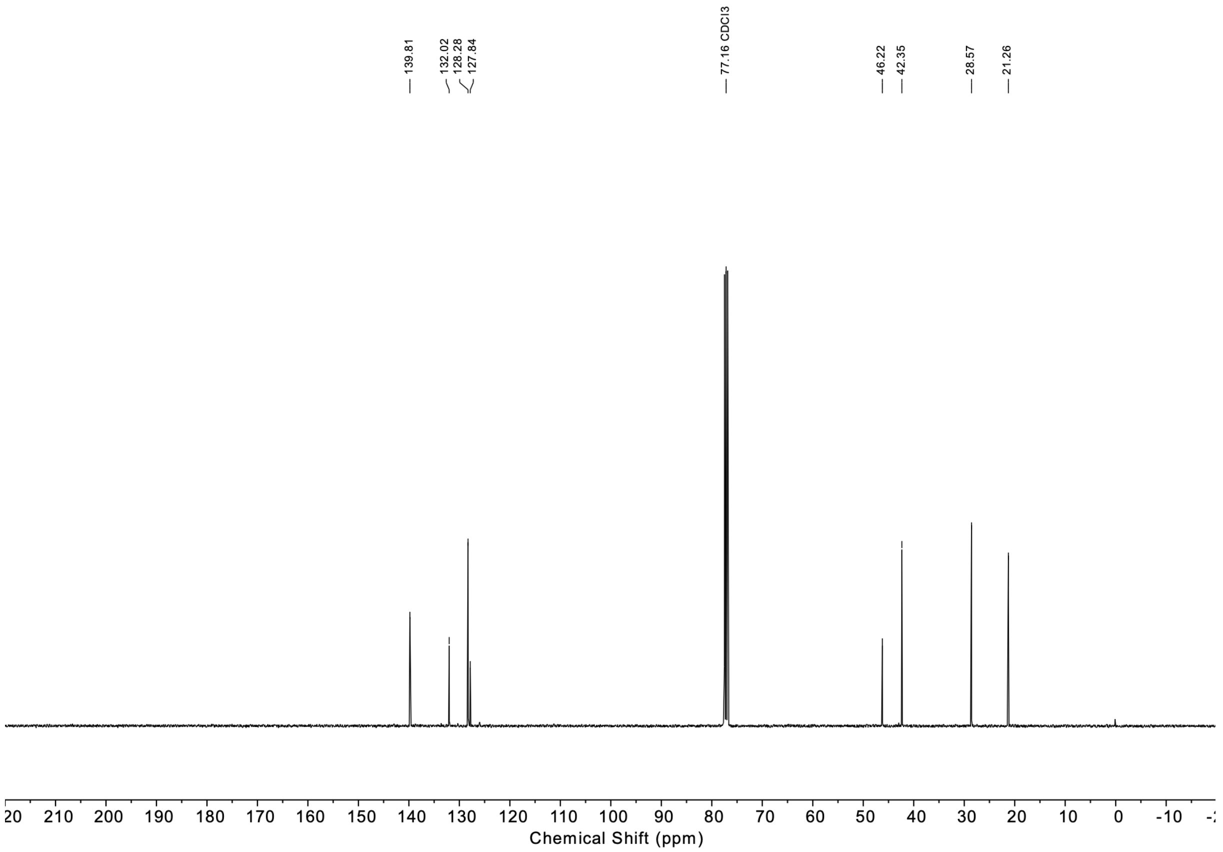

1-[(3,5-Diethylphenyl)methyl] tetrahydrothiophenium tetrafluoroborate (**1-Et**)

^{1}H-NMR (400 MHz, $CDCl_3$):

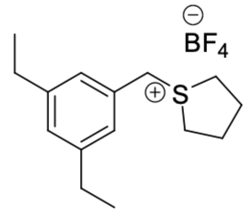

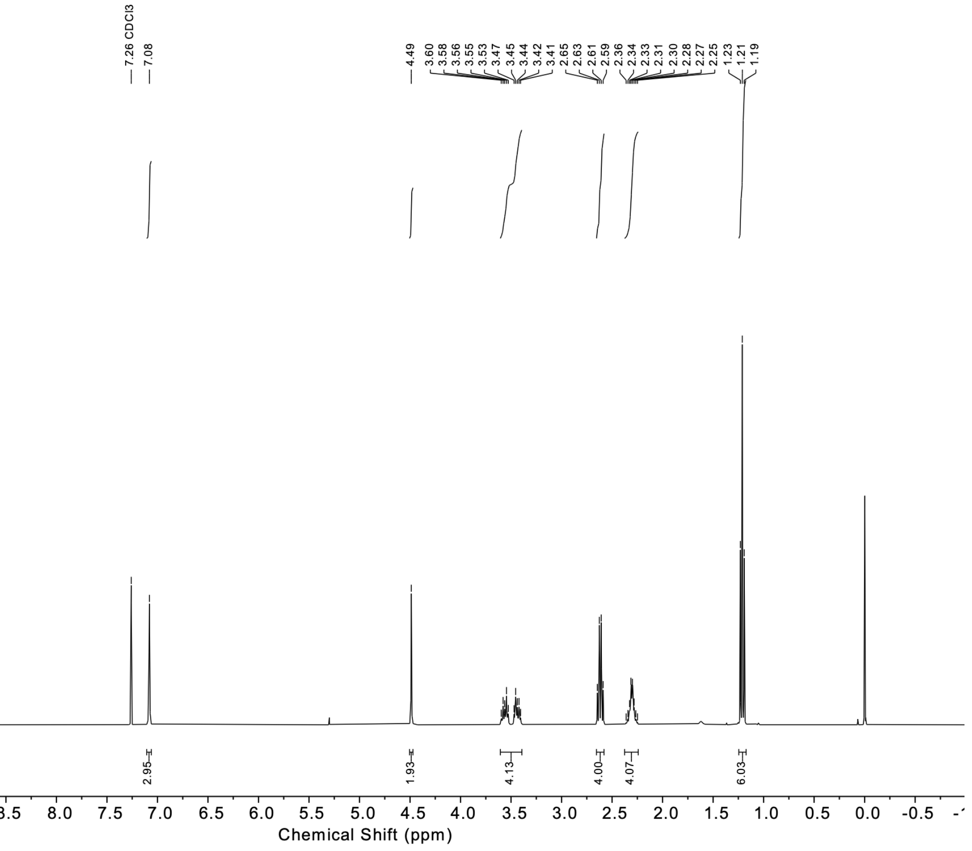

^{13}C{1H}-NMR (101 MHz, $CDCl_3$):

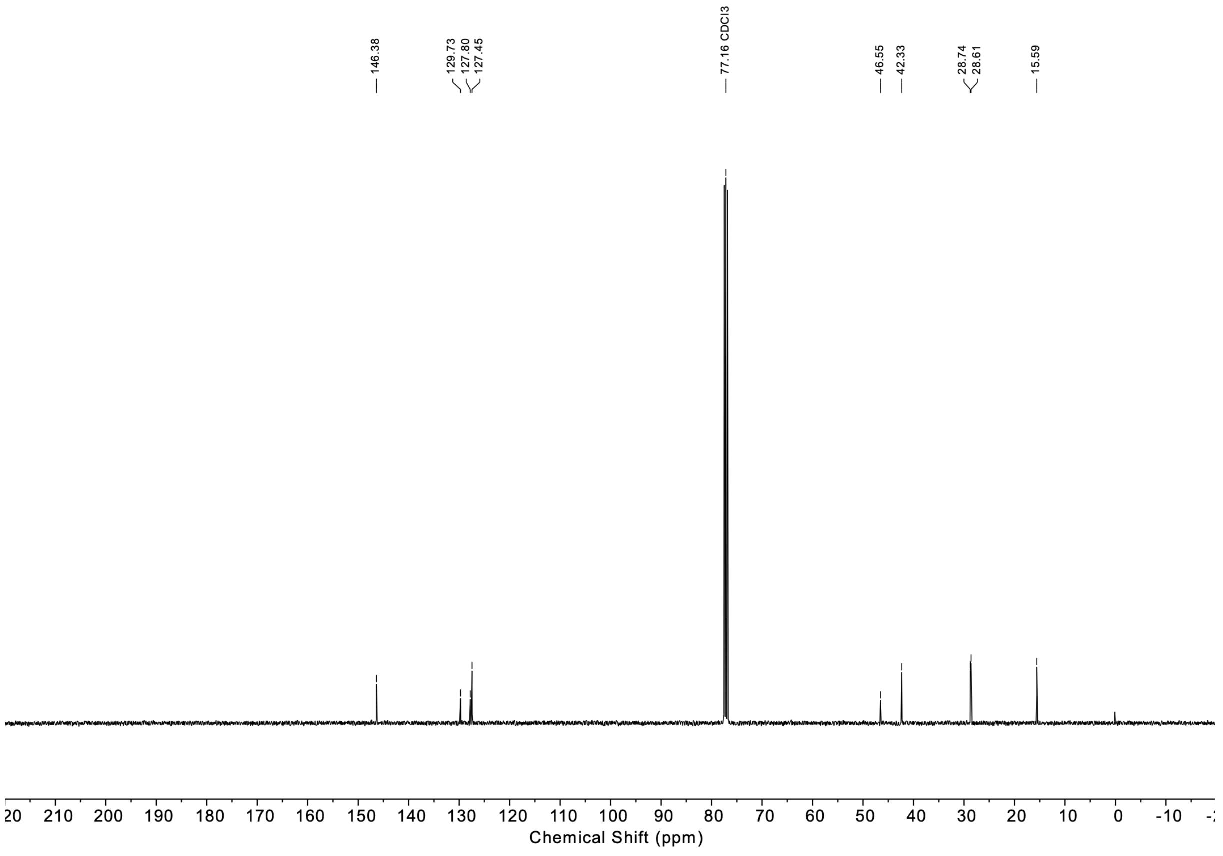

1-[(3,5-Di-*iso*-propyl-phenyl)methyl] tetrahydrothiophenium tetrafluoroborate (**1-^{i}Pr**)

^{1}H-NMR (400 MHz, CDCl$_3$):

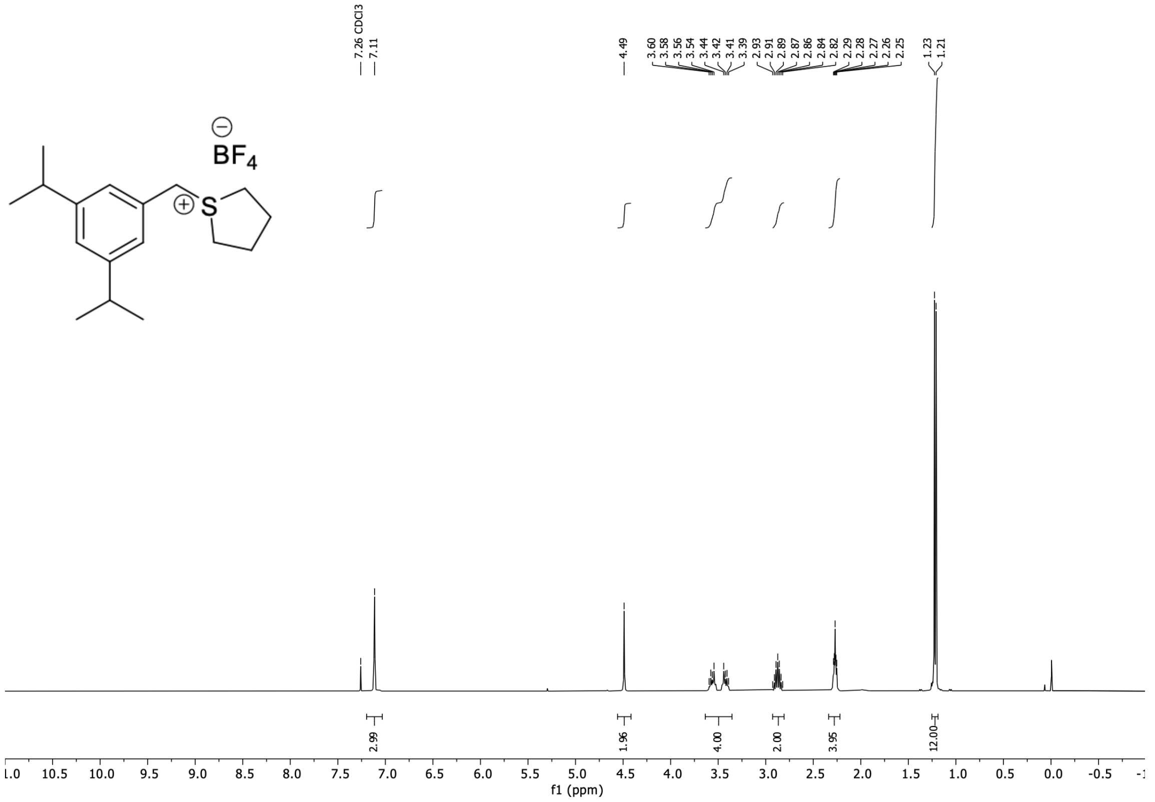

^{13}C{1H}-NMR (101 MHz, CDCl$_3$):

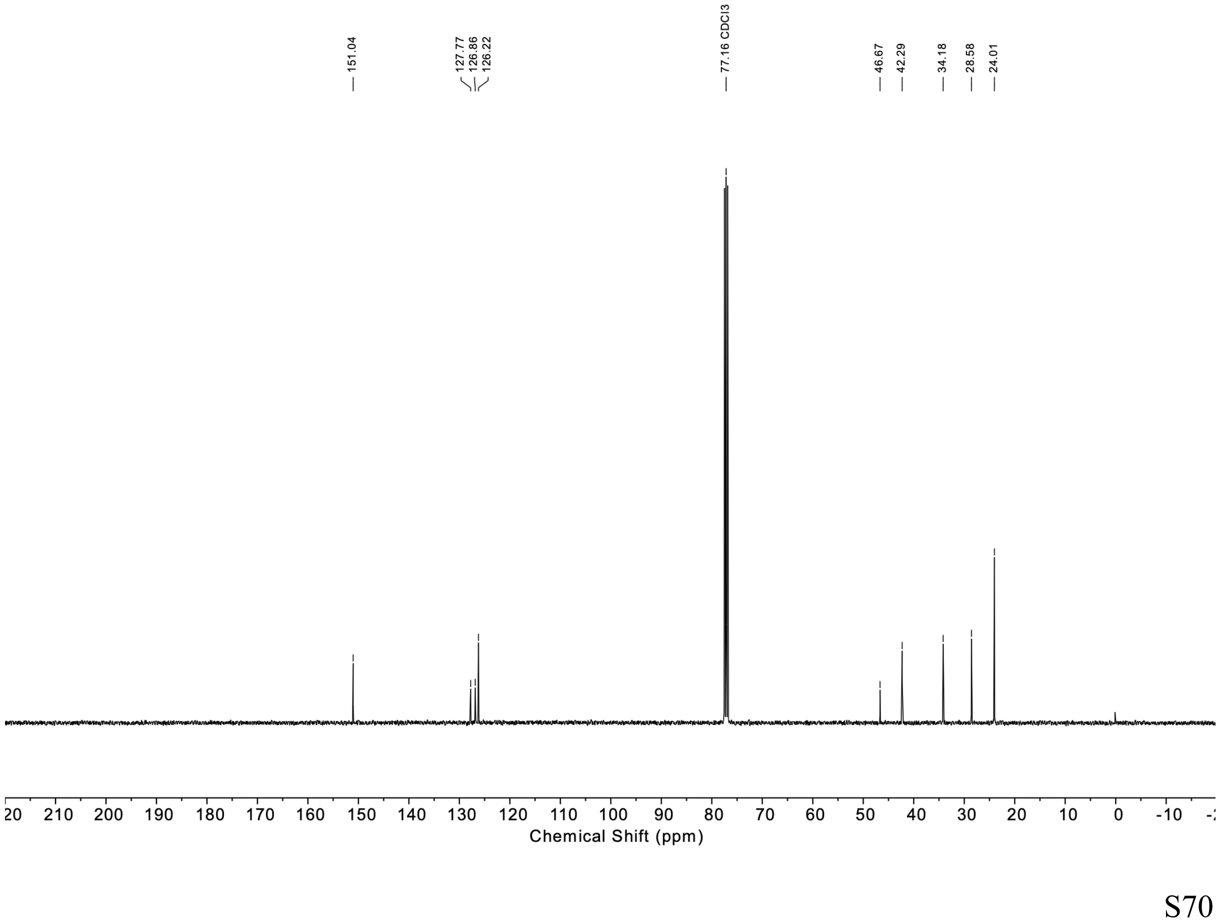

1-[(3,5-Di-*tert*-butyl-phenyl)methyl] tetrahydrothiophenium tetrafluoroborate (**1-^{t}Bu**)

^{1}H-NMR (400 MHz, $CDCl_3$):

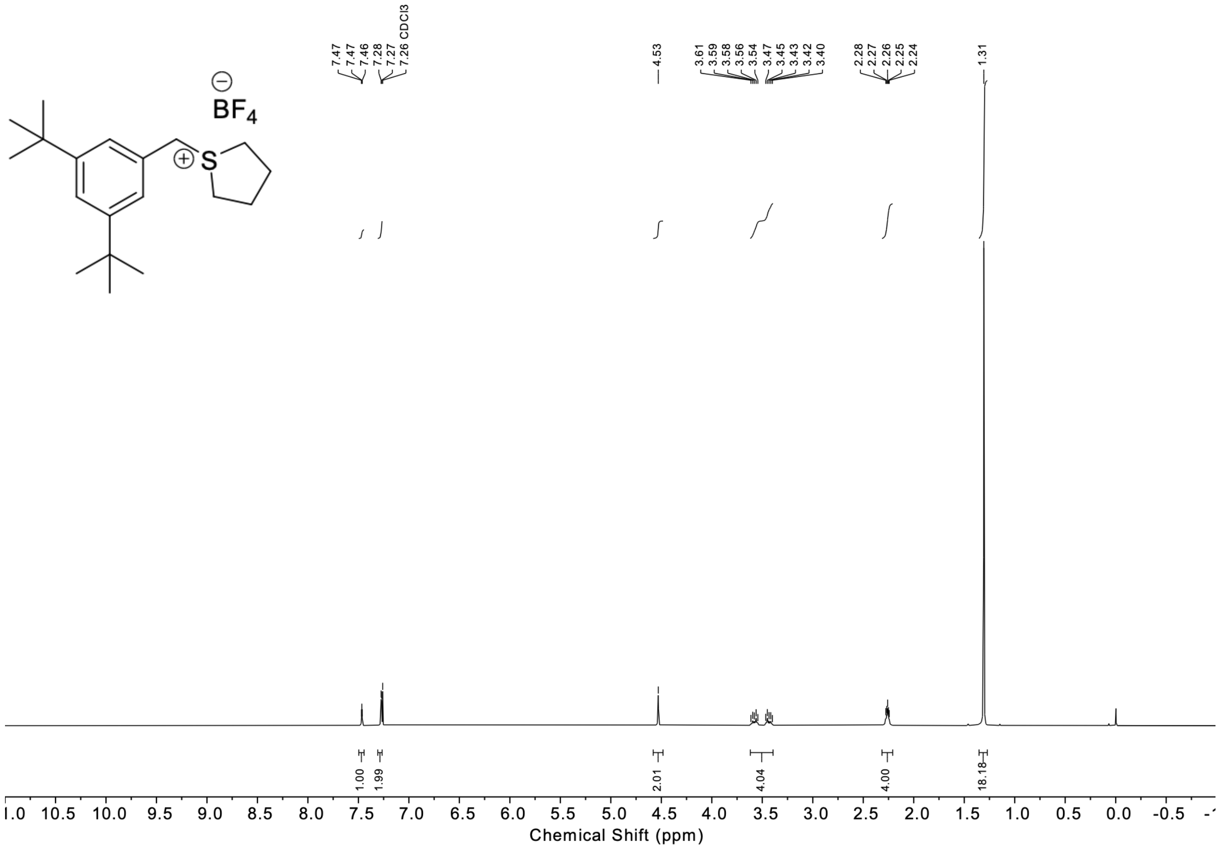

^{13}C{1H}-NMR (101 MHz, $CDCl_3$):

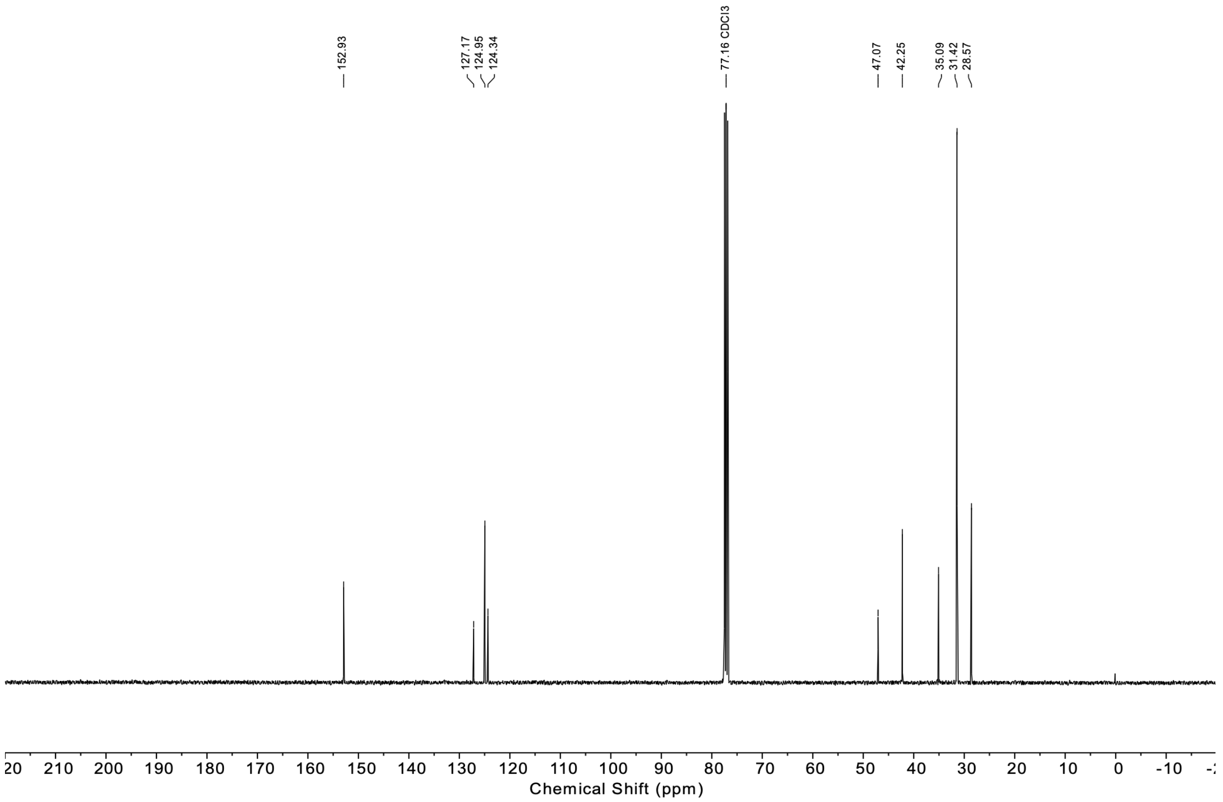

Bis-(3,5-di-*tert*-butyl-phenyl)acetylene

^{1}H-NMR (400 MHz, $CDCl_3$):

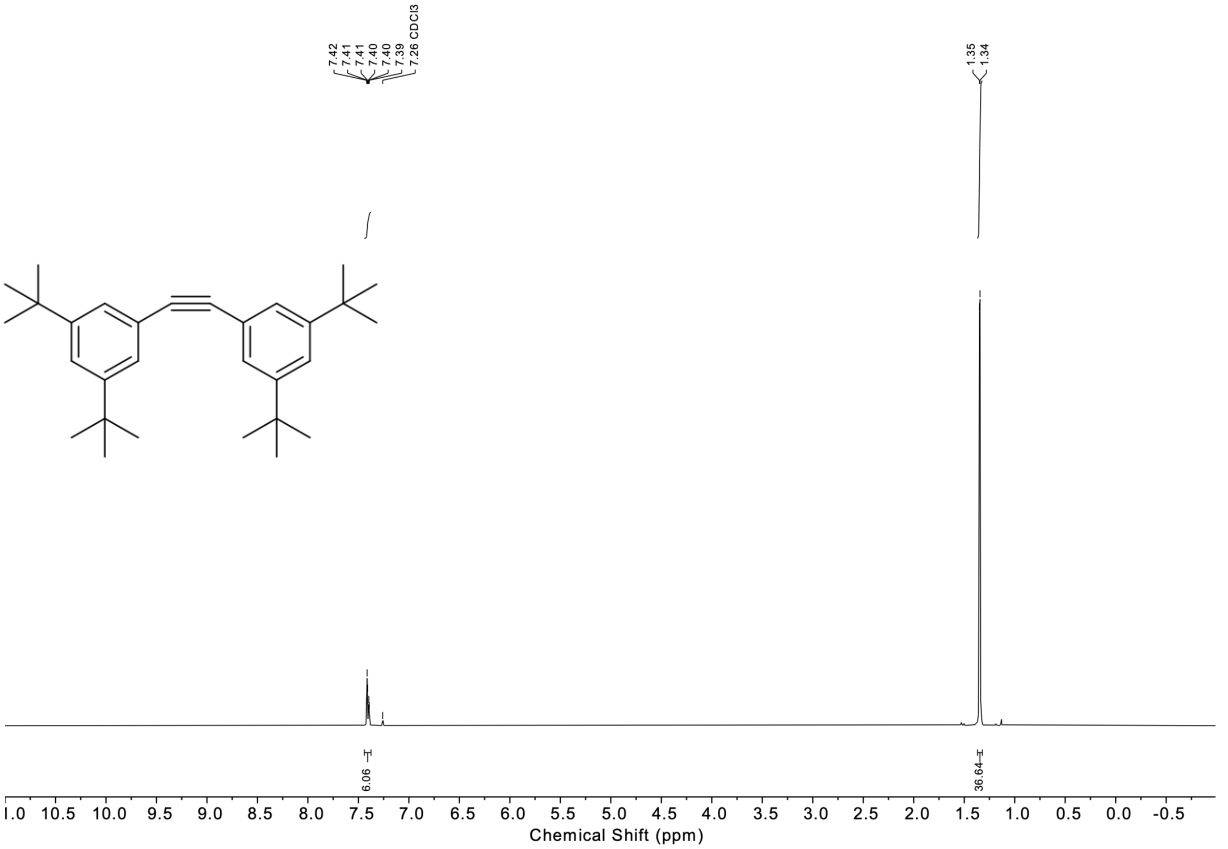

^{13}C{1H}-NMR (101 MHz, $CDCl_3$):

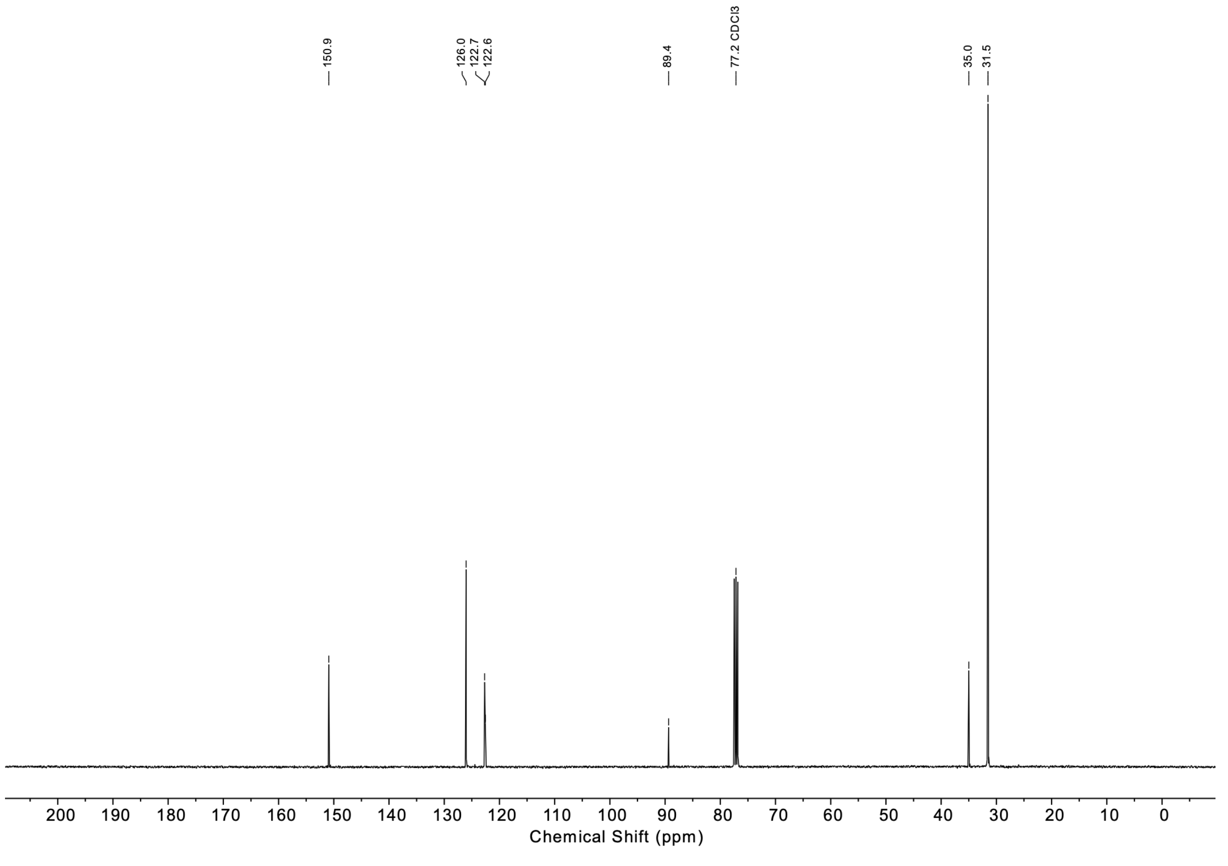

1,1′-(1*Z*)-1,2-Ethenediylbis[3,5-di-*tert*-butylbenzene]

^{1}H-NMR (400 MHz, CDCl$_3$):

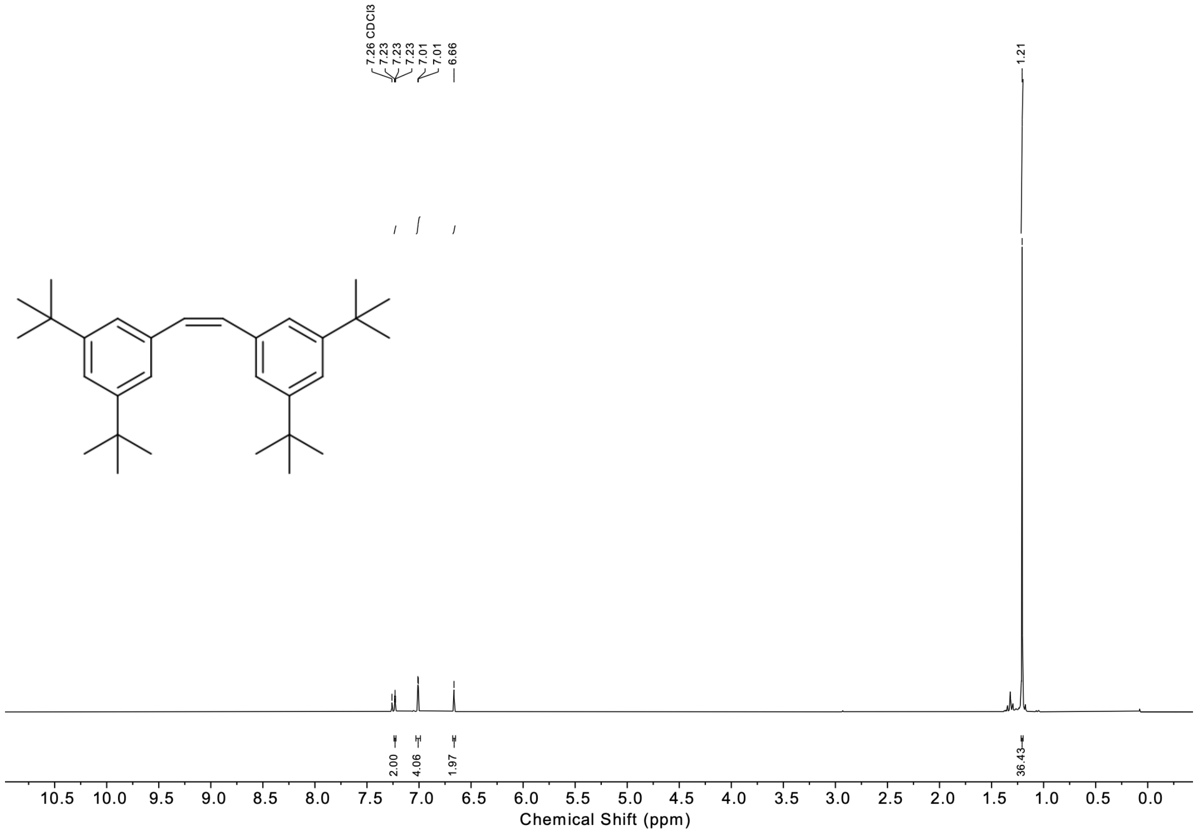

^{13}C{1H}-NMR (101 MHz, CDCl$_3$):

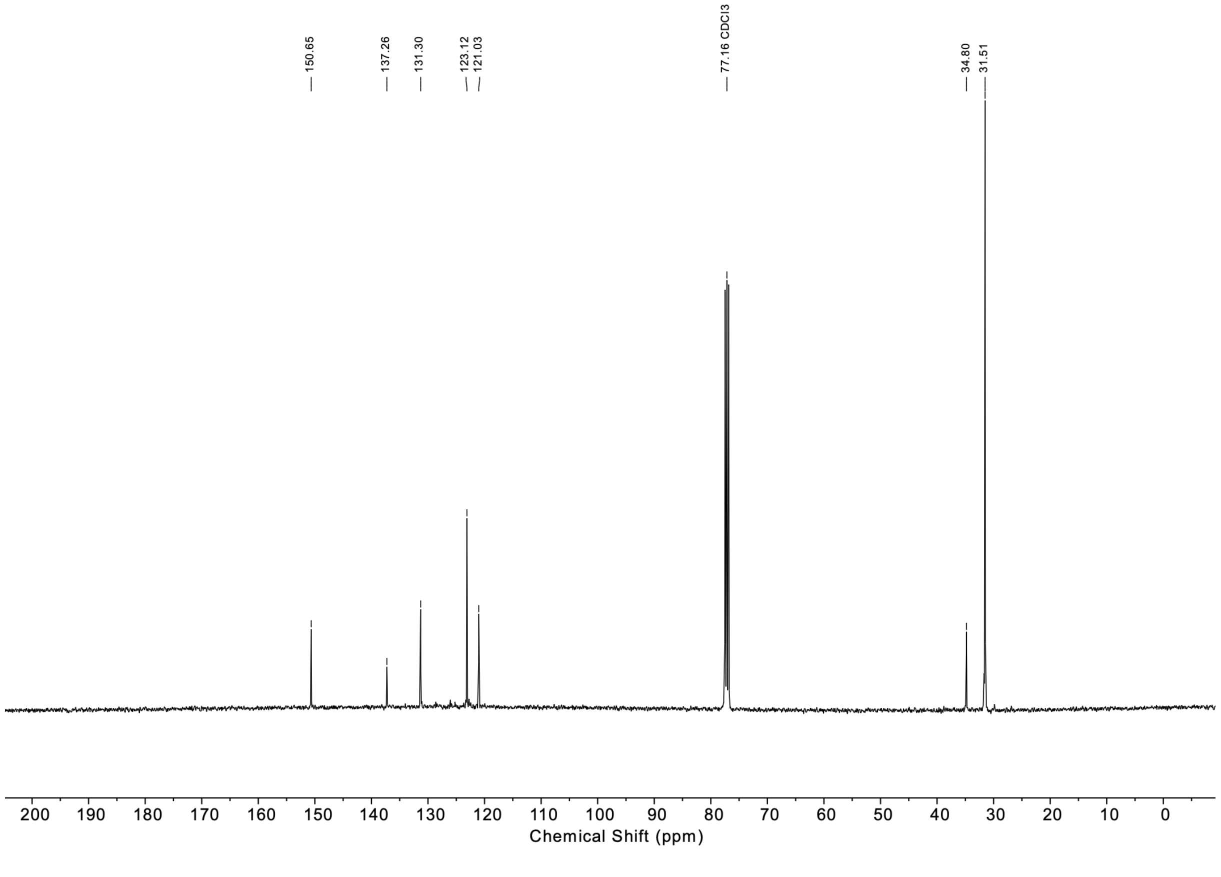

1,1′-(1E)-1,2-Ethenediylbis[3,5-di-*tert*-butylbenzene]

^{1}H-NMR (400 MHz, CDCl$_3$):

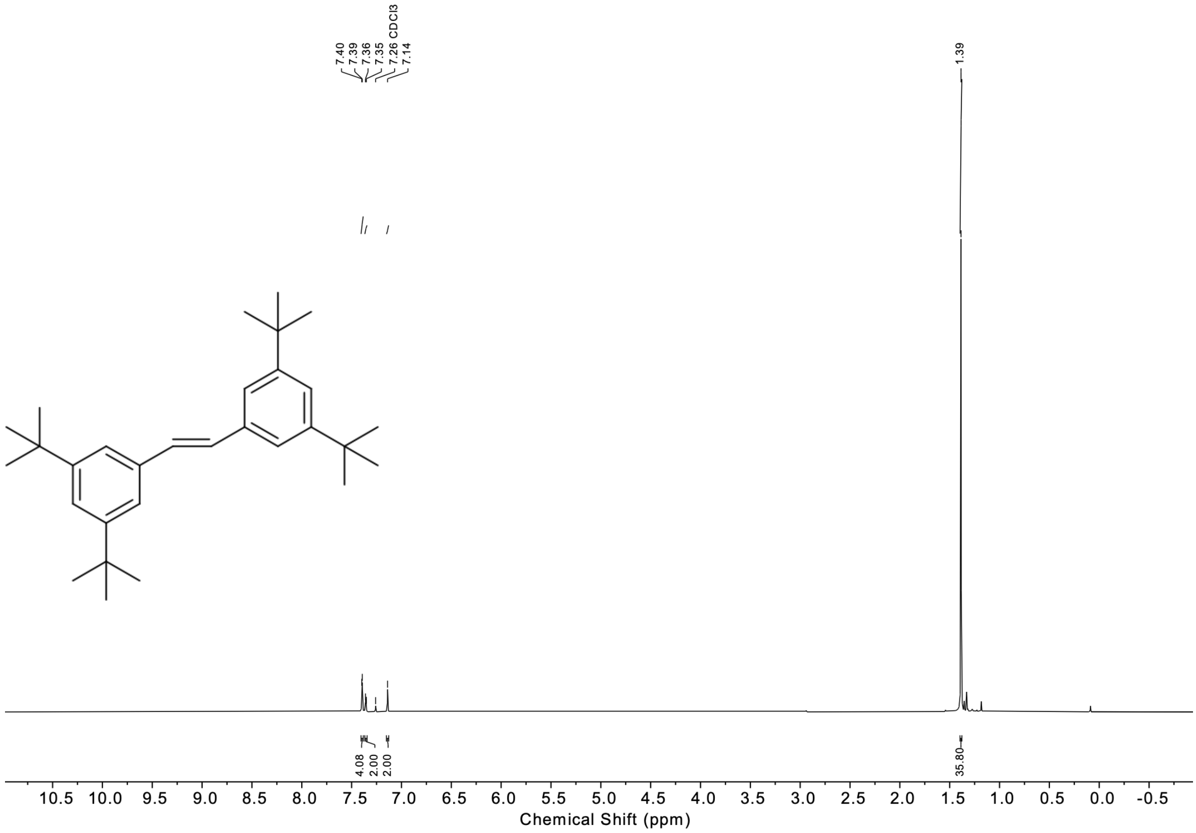

^{13}C{1H}-NMR (101 MHz, CDCl$_3$):

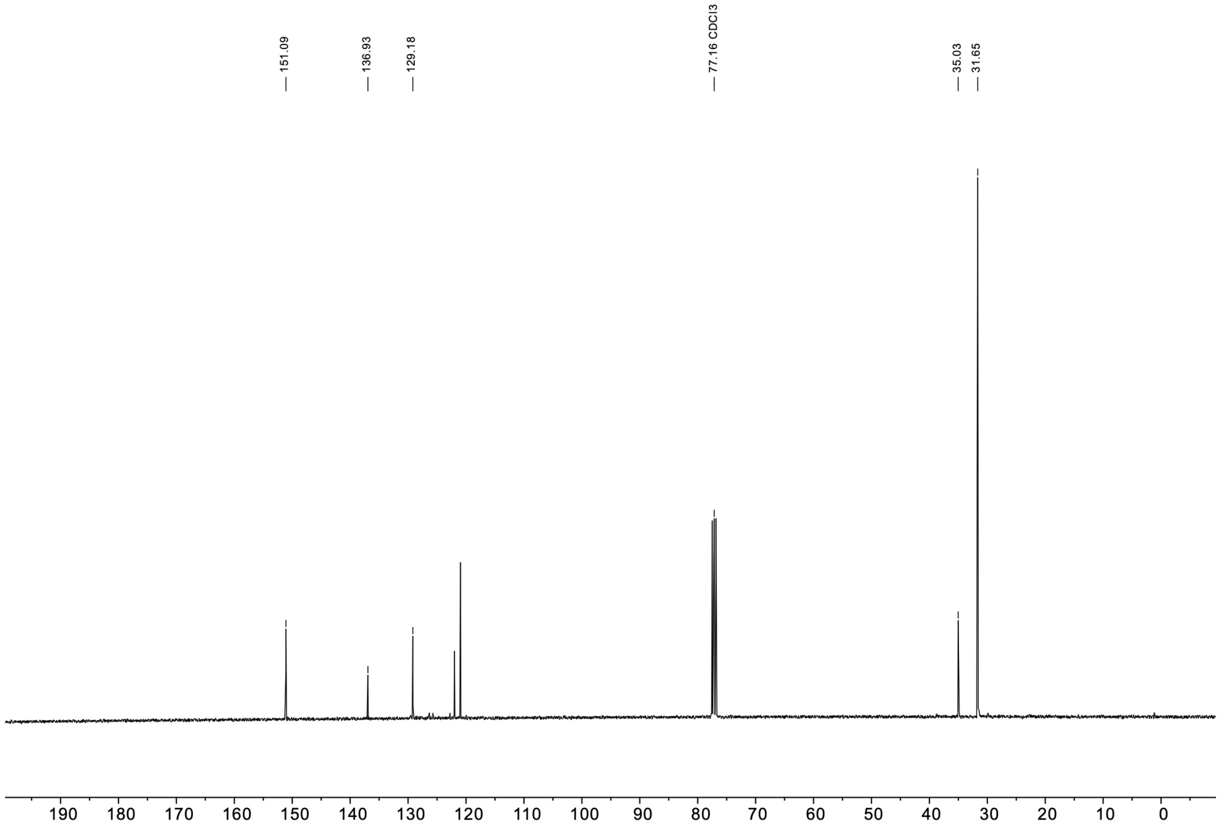

Cis-diphenyloxirane (**3-*cis*-H**)

^{1}H-NMR (400 MHz, CD_2Cl_2):

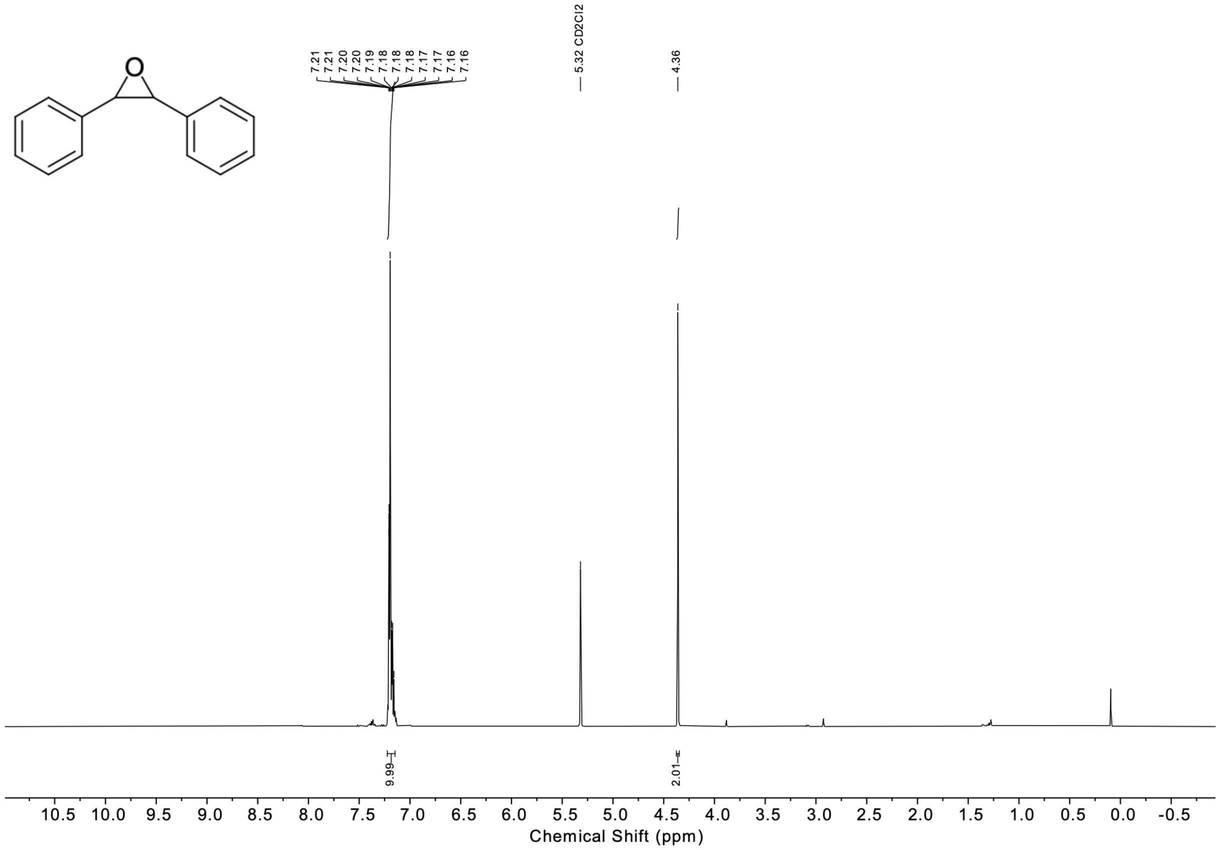

^{13}C{1H}-NMR (101 MHz, CD_2Cl_2):

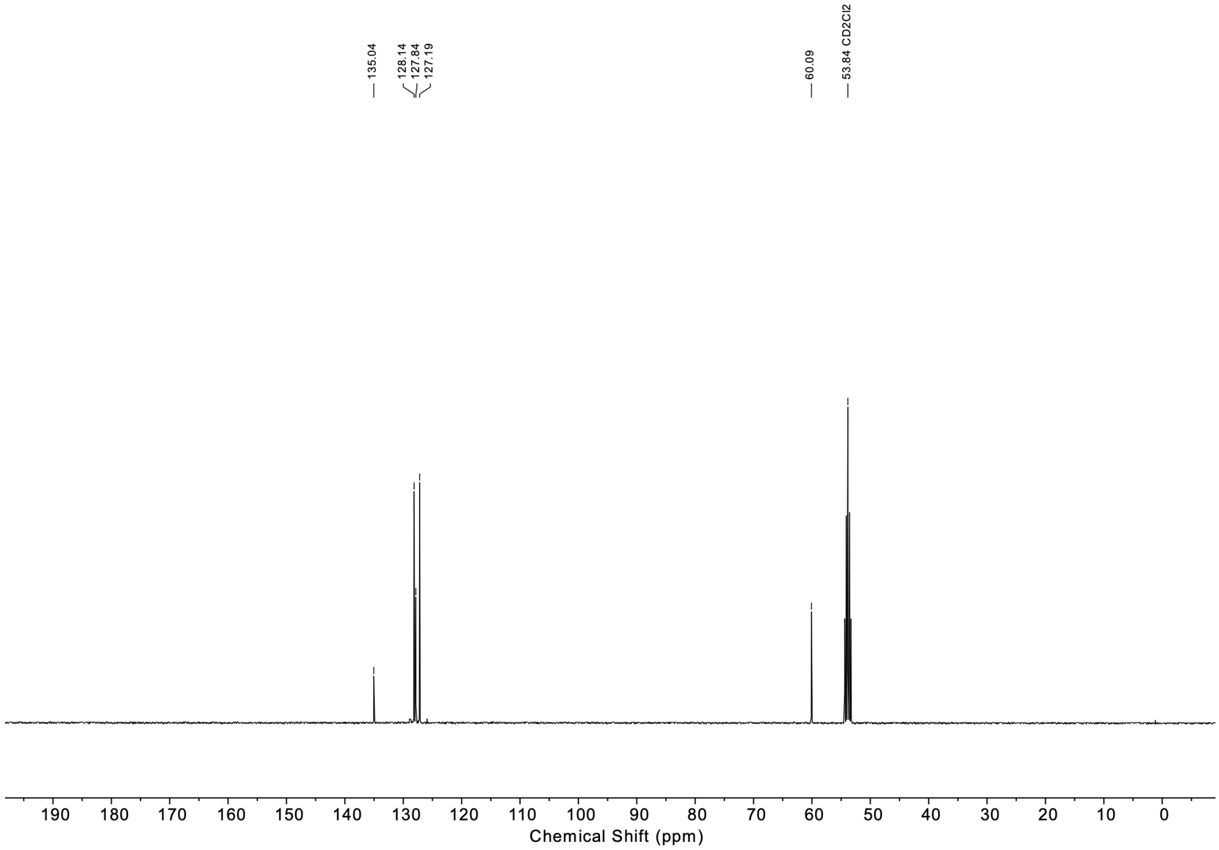

Trans-diphenyloxirane (**3-*trans*-H**)

^{1}H-NMR (400 MHz, CD_2Cl_2):

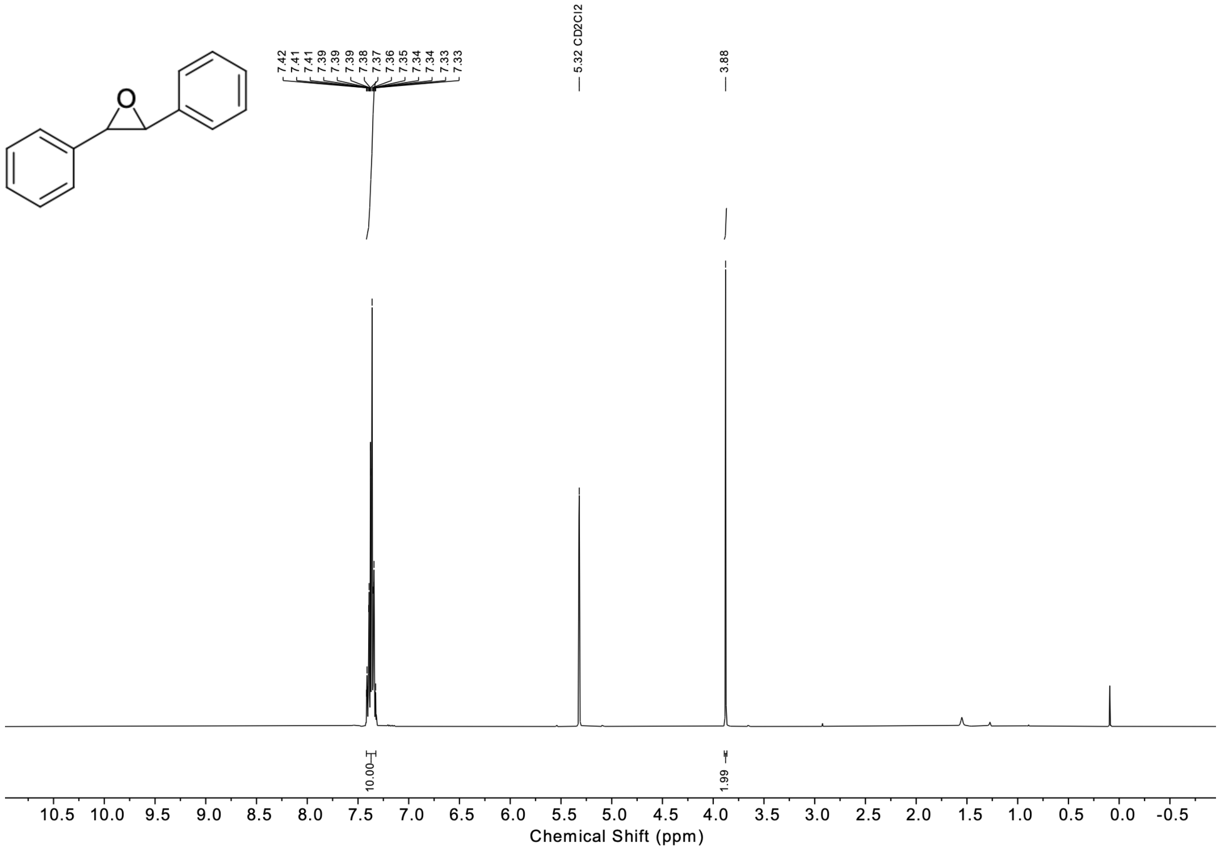

^{13}C{1H}-NMR (101 MHz, CD_2Cl_2):

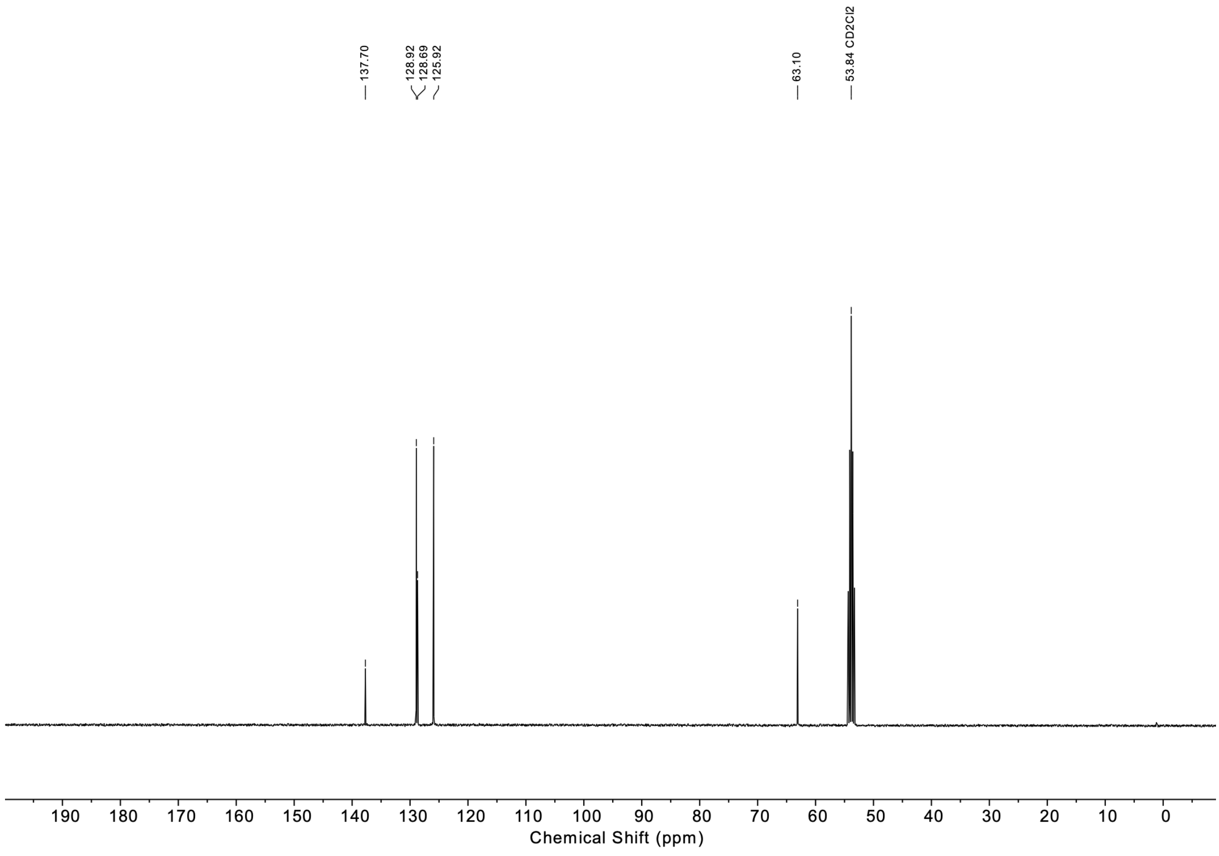

Cis-2,3-Bis[3,5-di-tert-butylphenyl]oxirane (**3-*cis*-^{t}Bu**)

^{1}H-NMR (400 MHz, $CDCl_3$):

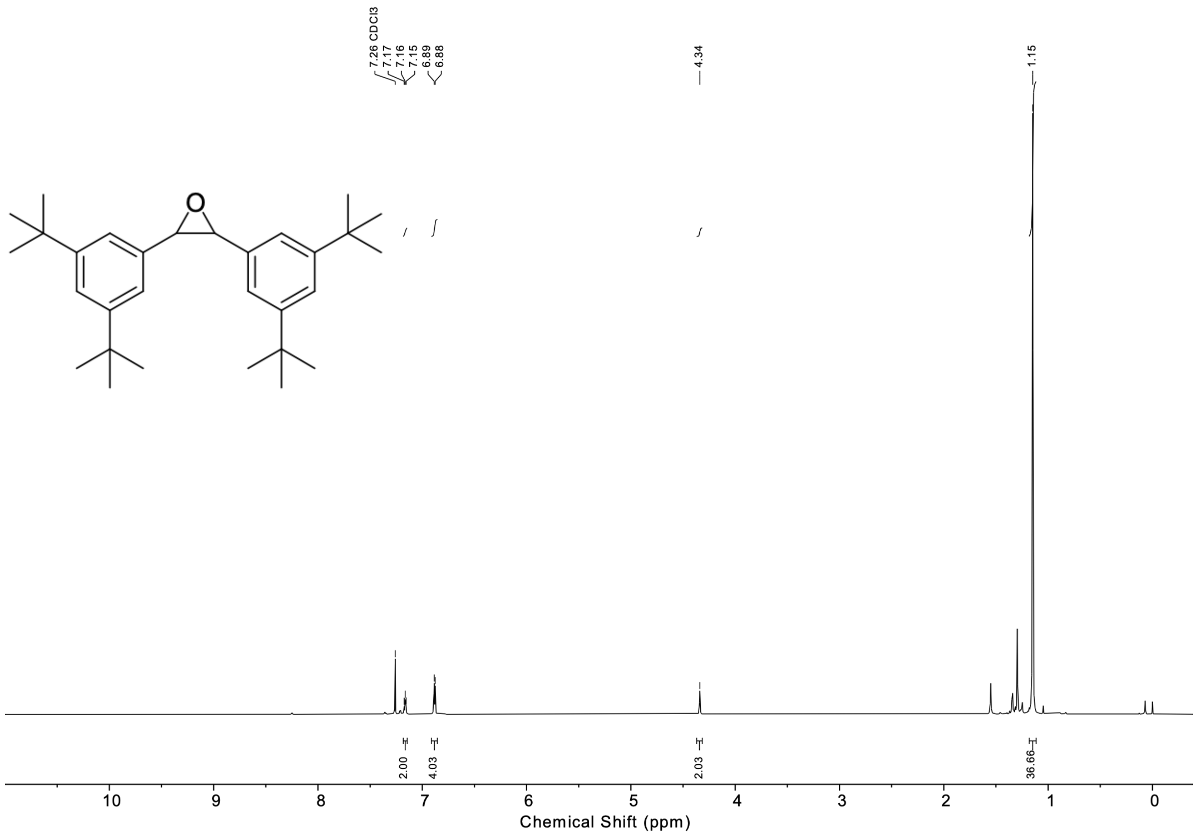

^{13}C{1H}-NMR (101 MHz, $CDCl_3$):

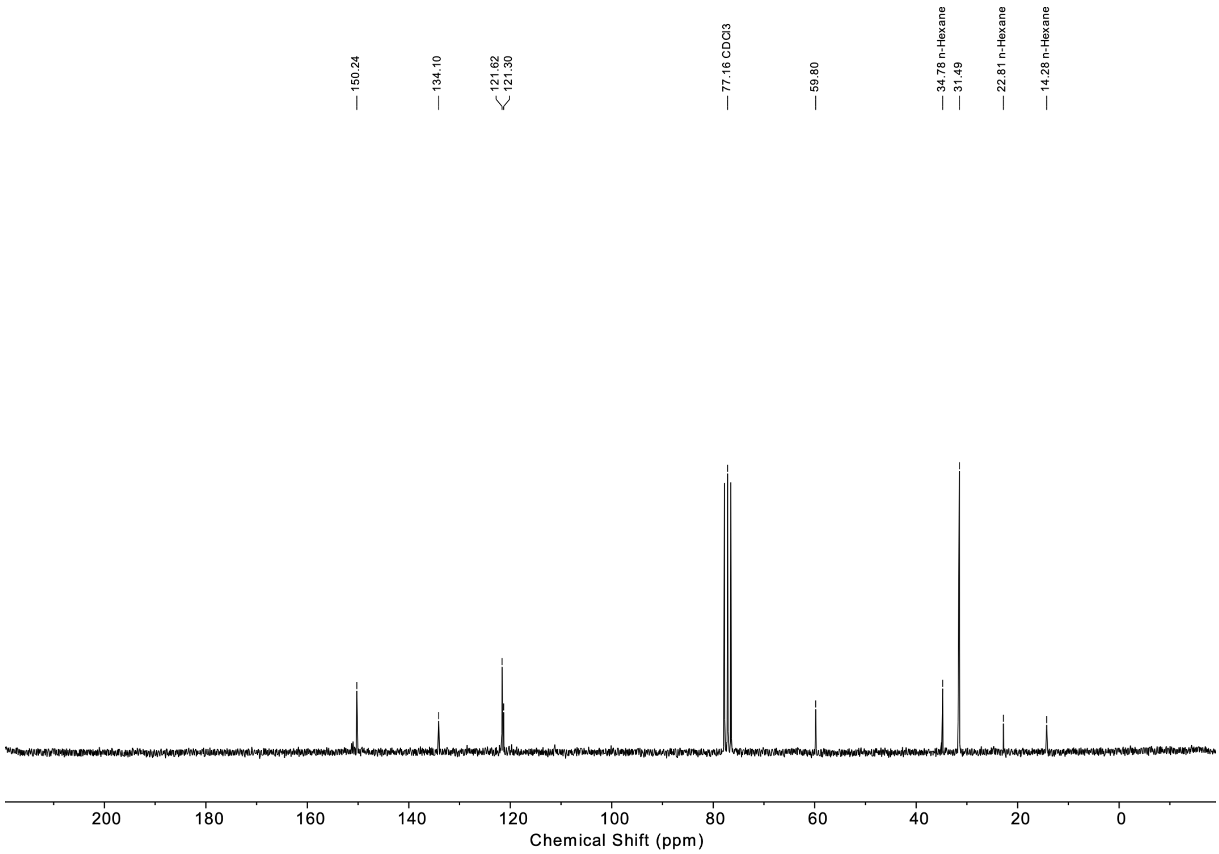

Trans-2,3-Bis[3,5-di-tert-butylphenyl]oxirane (**3-*trans*-^{t}Bu**)

^{1}H-NMR (400 MHz, $CDCl_3$):

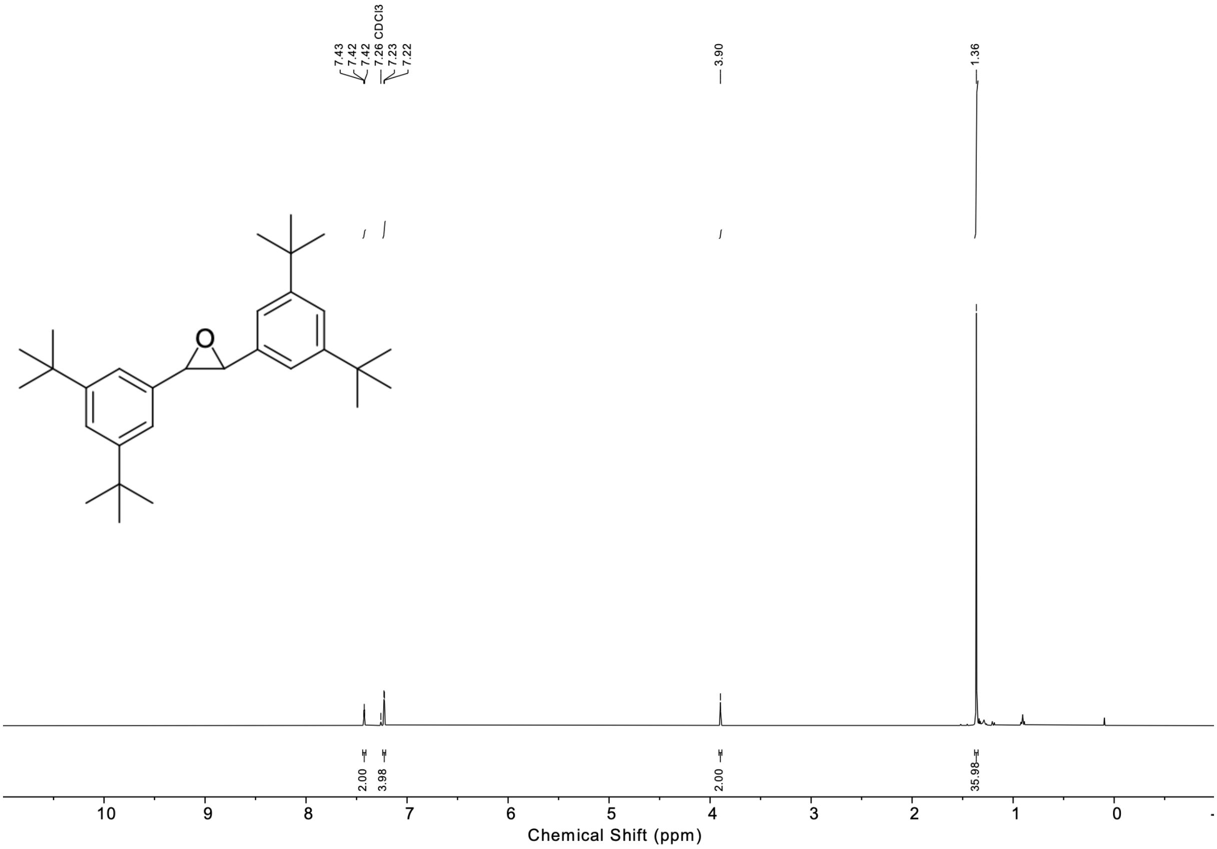

^{13}C{1H}-NMR (101 MHz, $CDCl_3$):

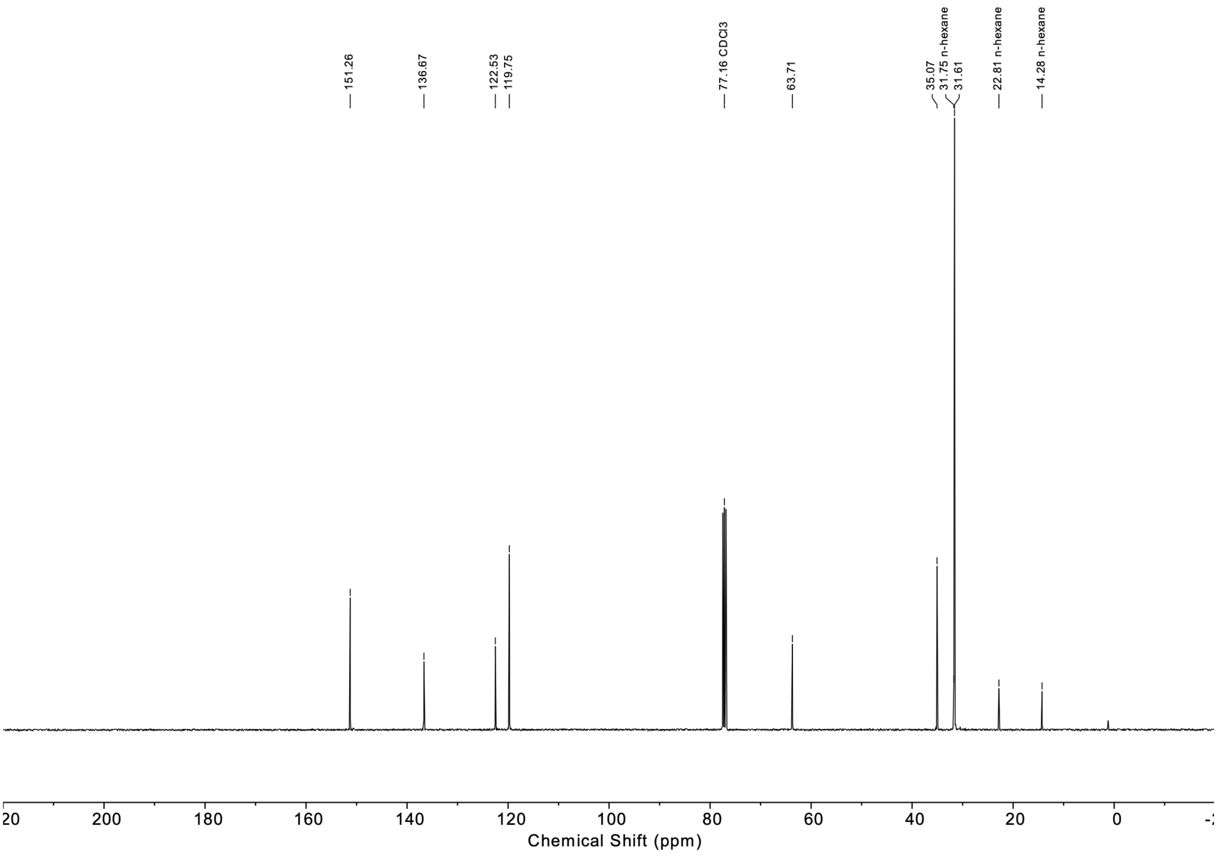

Syn-1,2-diphenyl-2-hydroxyethyl(dimethyl) sulfonium iodide (**4-*syn*-H**)

^{1}H-NMR (400 MHz, DMSO-d_6):

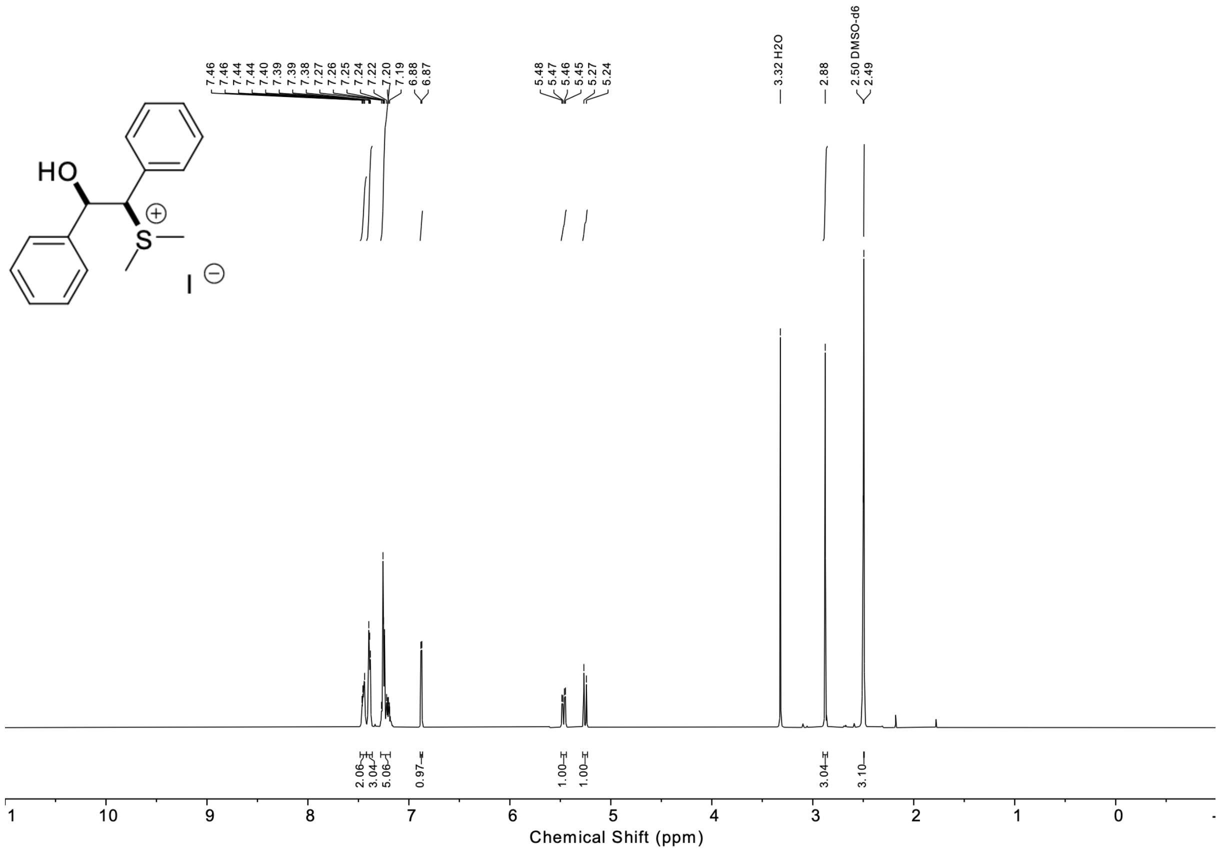

^{13}C{1H}-NMR (101 MHz, DMSO-d_6):

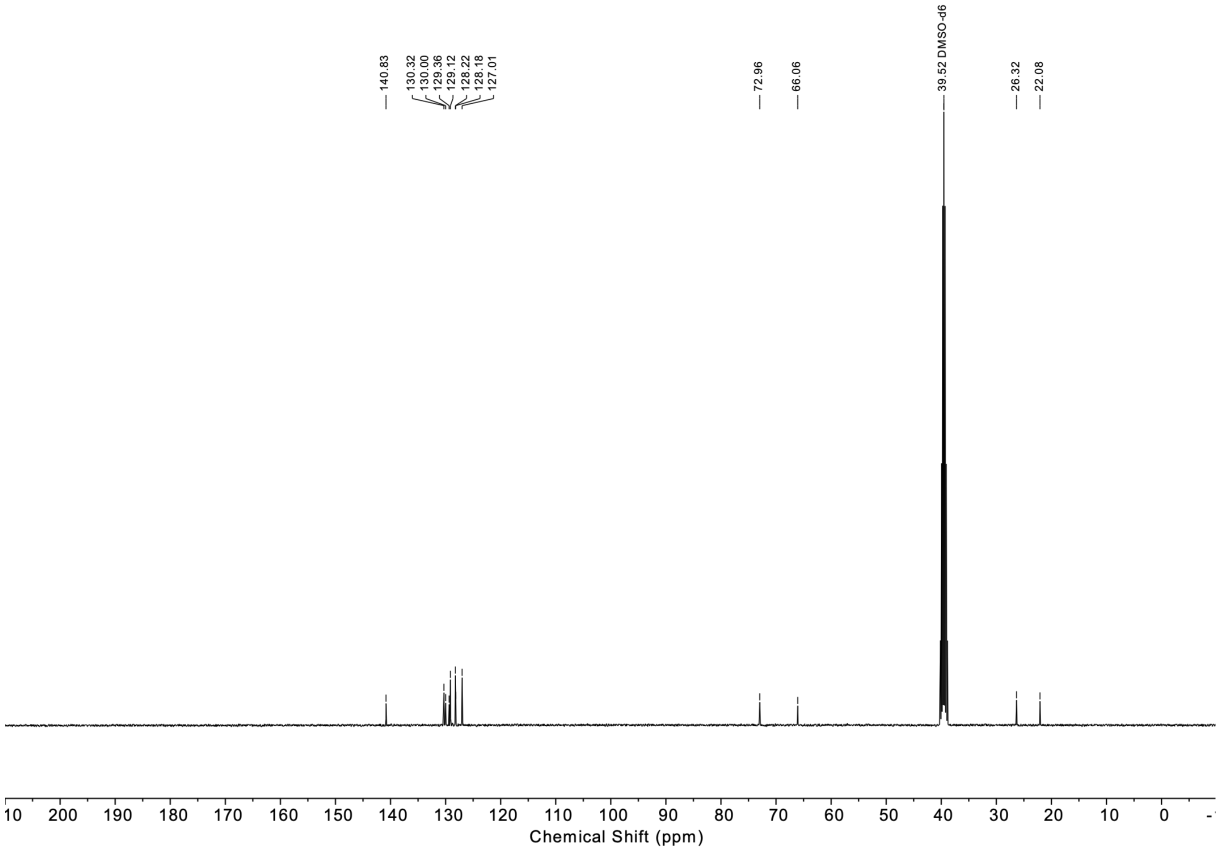

Anti-1,2-diphenyl-2-hydroxyethyl(dimethyl) sulfonium iodide (**4-*anti*-H**)

^{1}H-NMR (400 MHz, DMSO-*d*6):

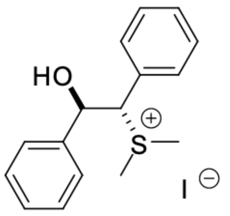

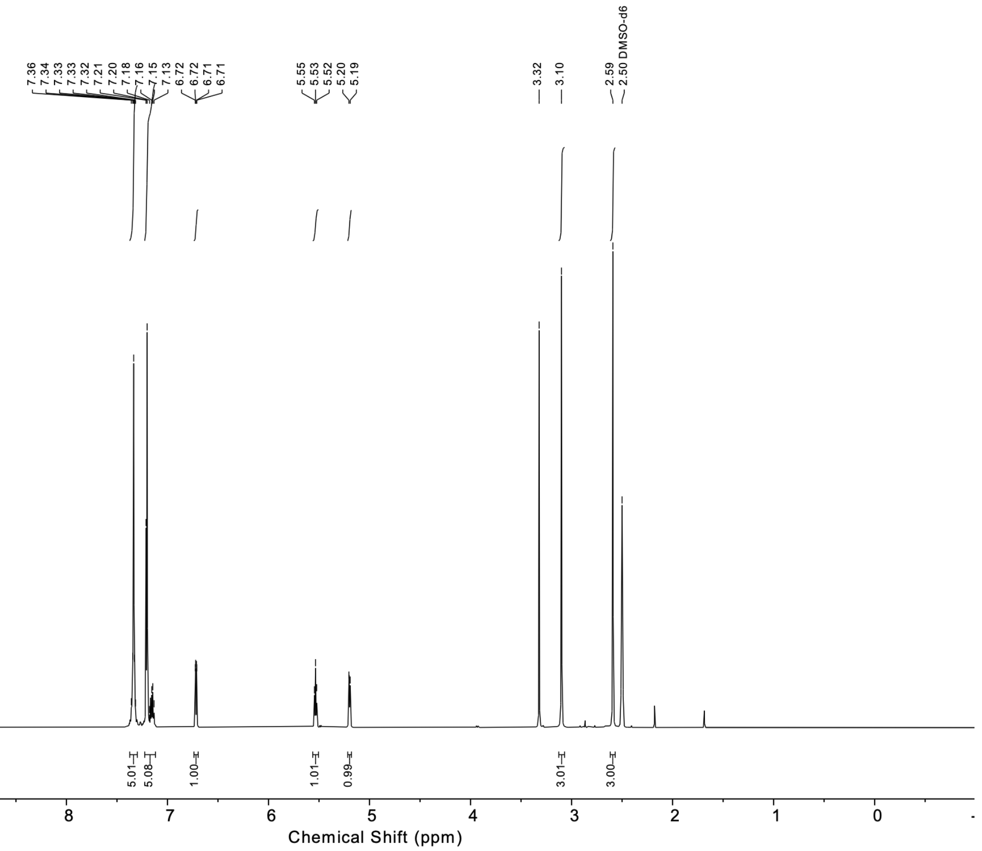

^{13}C{1H}-NMR (101 MHz, DMSO-*d*6):

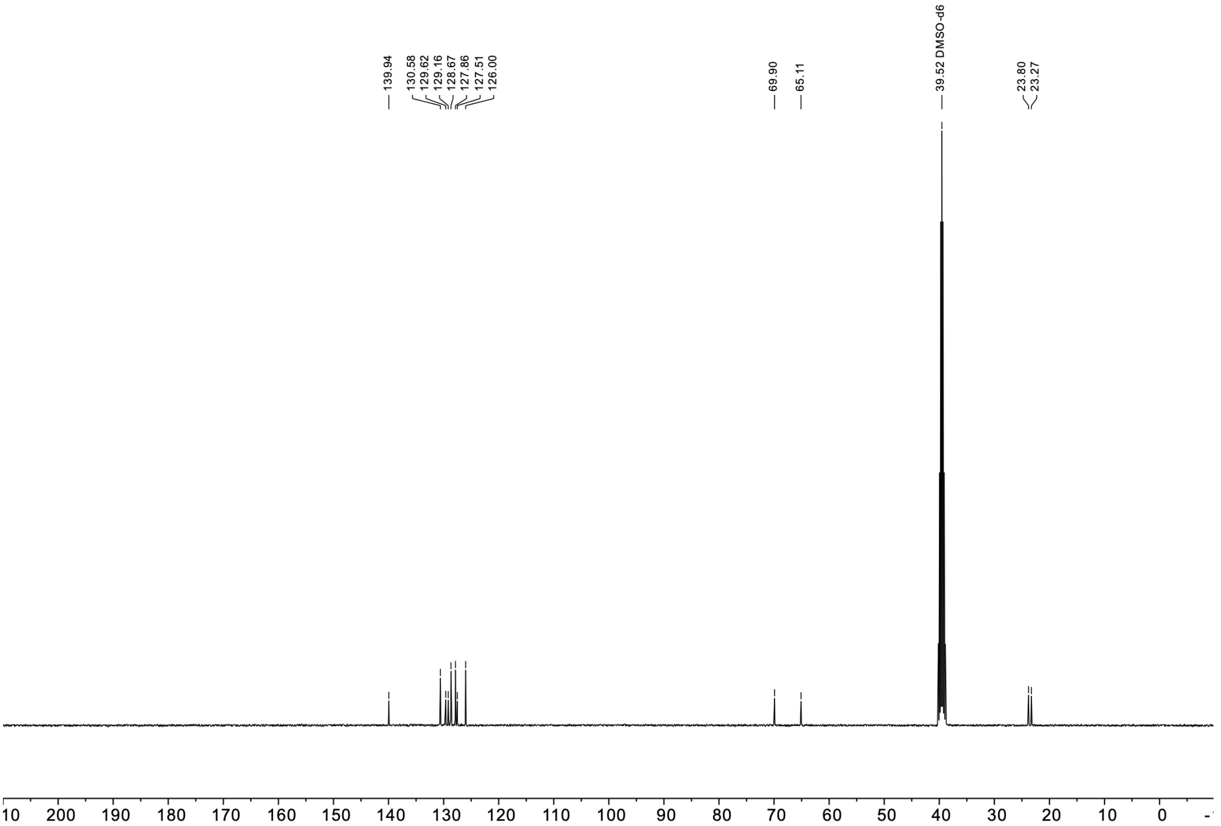

Syn-1,2-Bis[3,5-di-tert-butyl]phenyl-2-hydroxyethyl(dimethyl) sulfonium iodide (**4-*syn*-^{t}Bu**)

^{1}H-NMR (400 MHz, DMSO-d_6):

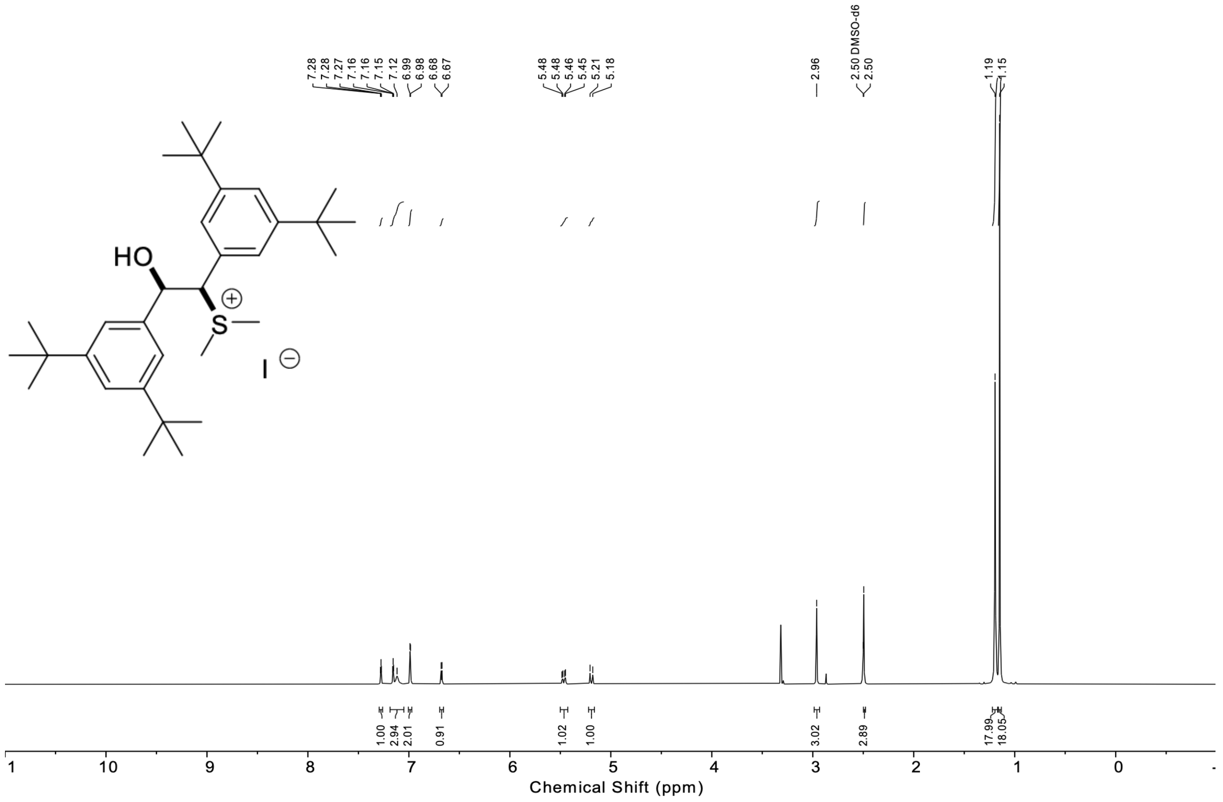

^{13}C{1H}-NMR (101 MHz, DMSO-d_6):

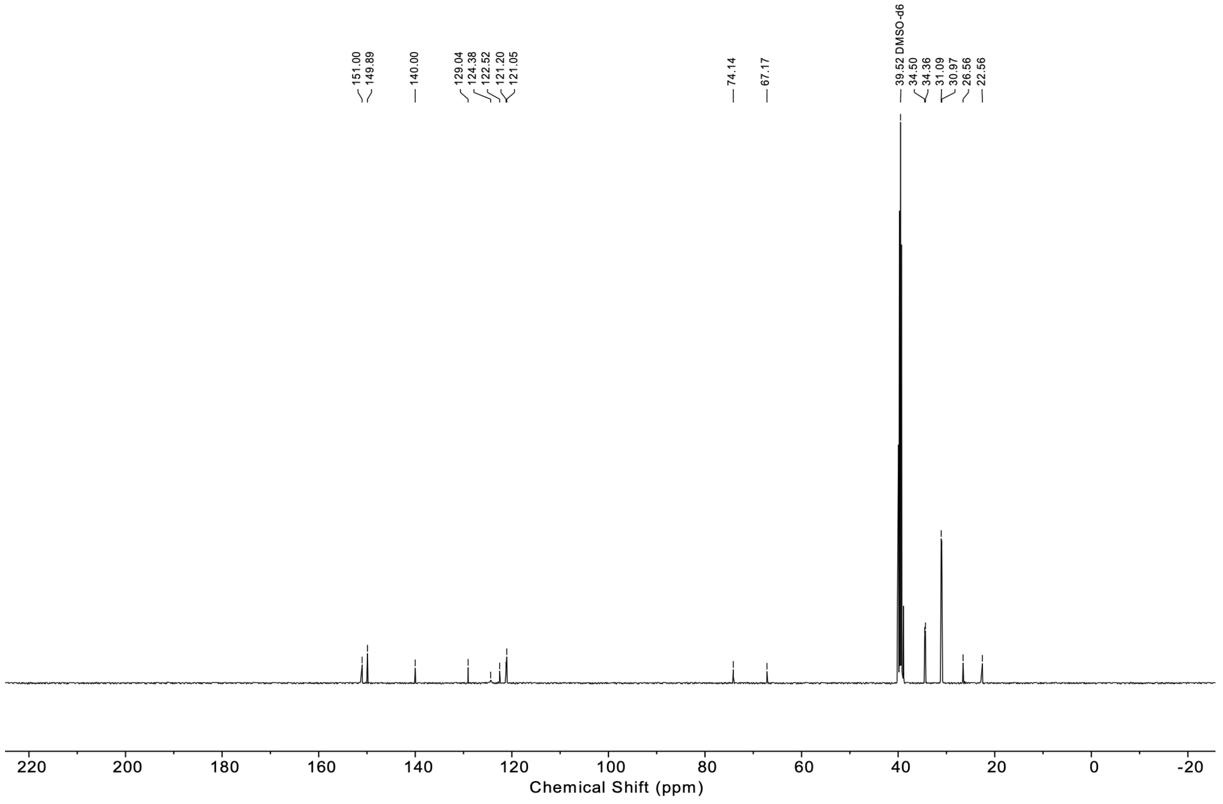

Anti-1,2-Bis[3,5-di-tert-butyl]phenyl-2-hydroxyethyl(dimethyl) sulfonium iodide (**4-*anti*-^{t}Bu**)

^{1}H-NMR (400 MHz, DMSO-d_6):

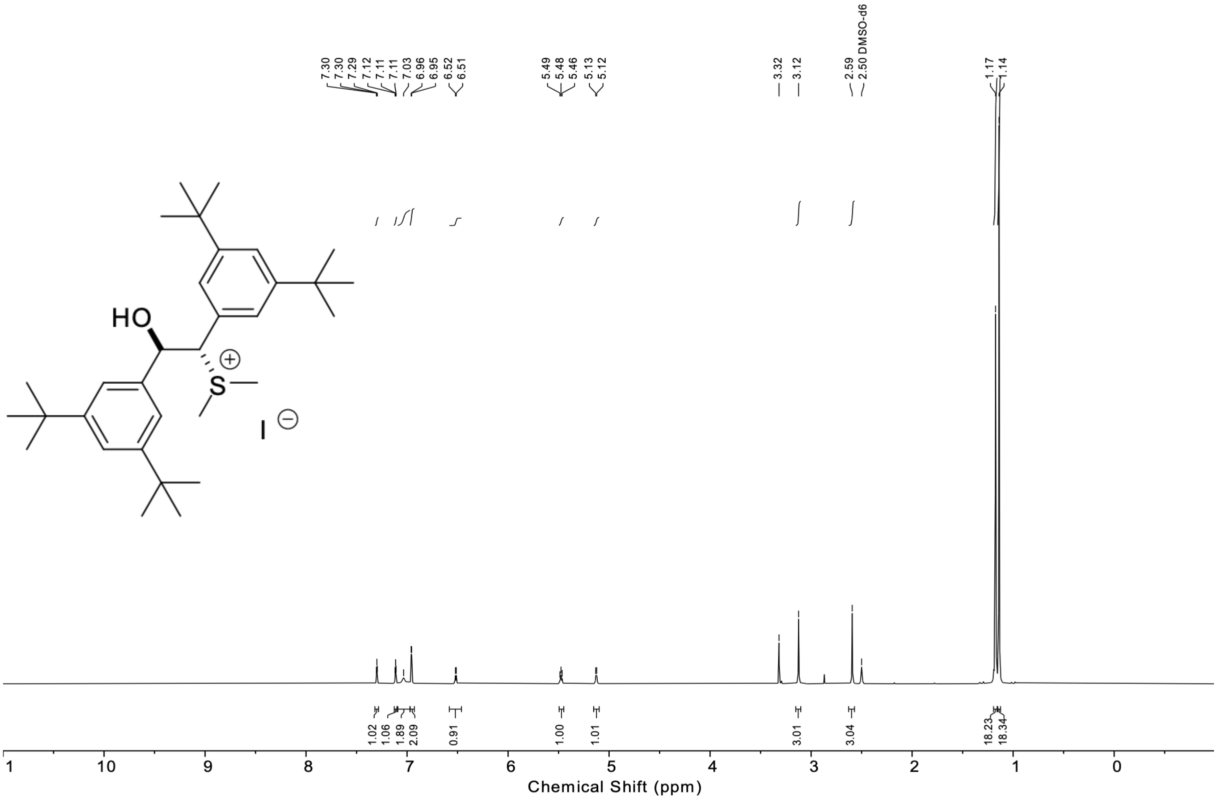

^{13}C{1H}-NMR (101 MHz, DMSO-d_6):

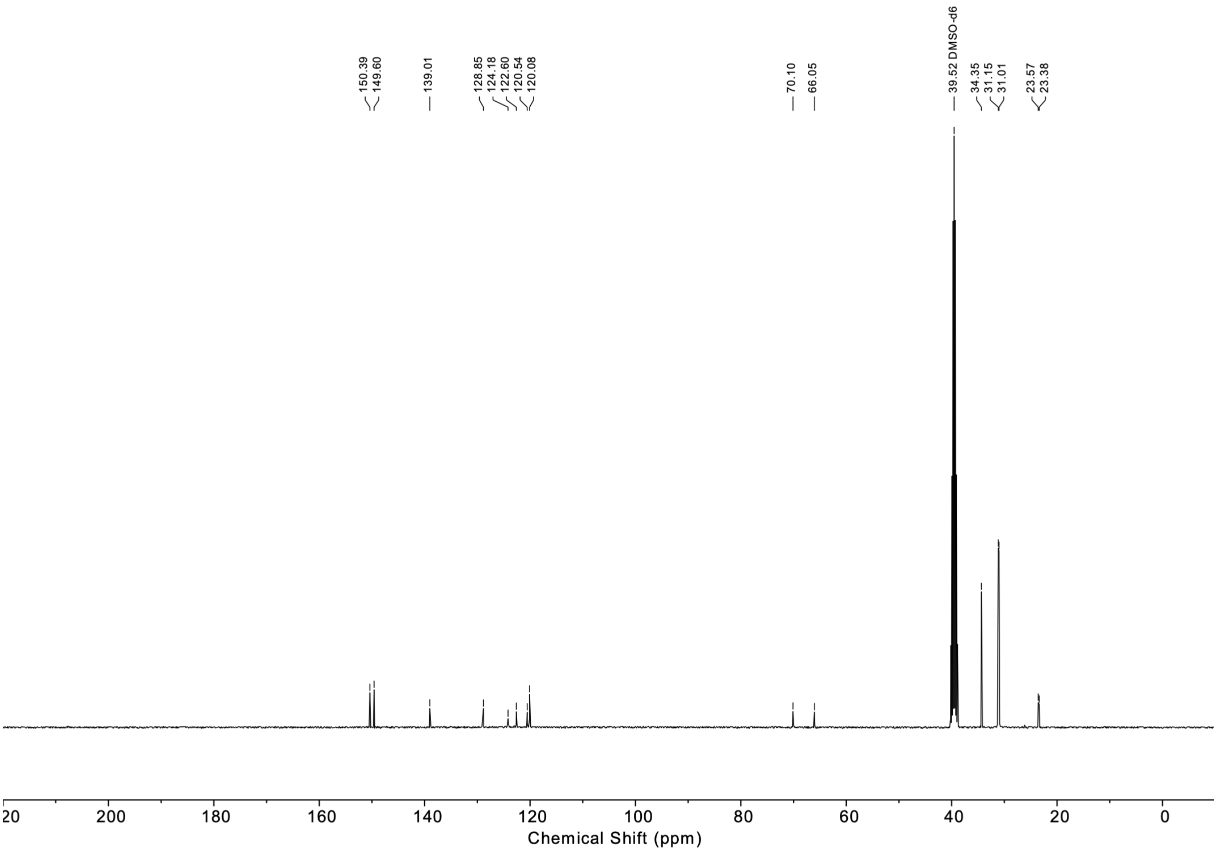

2-(3,5-Dimethylphenyl)-3-phenyl oxirane (**3-H-Me**)

^{1}H-NMR (400 MHz, $CDCl_3$):

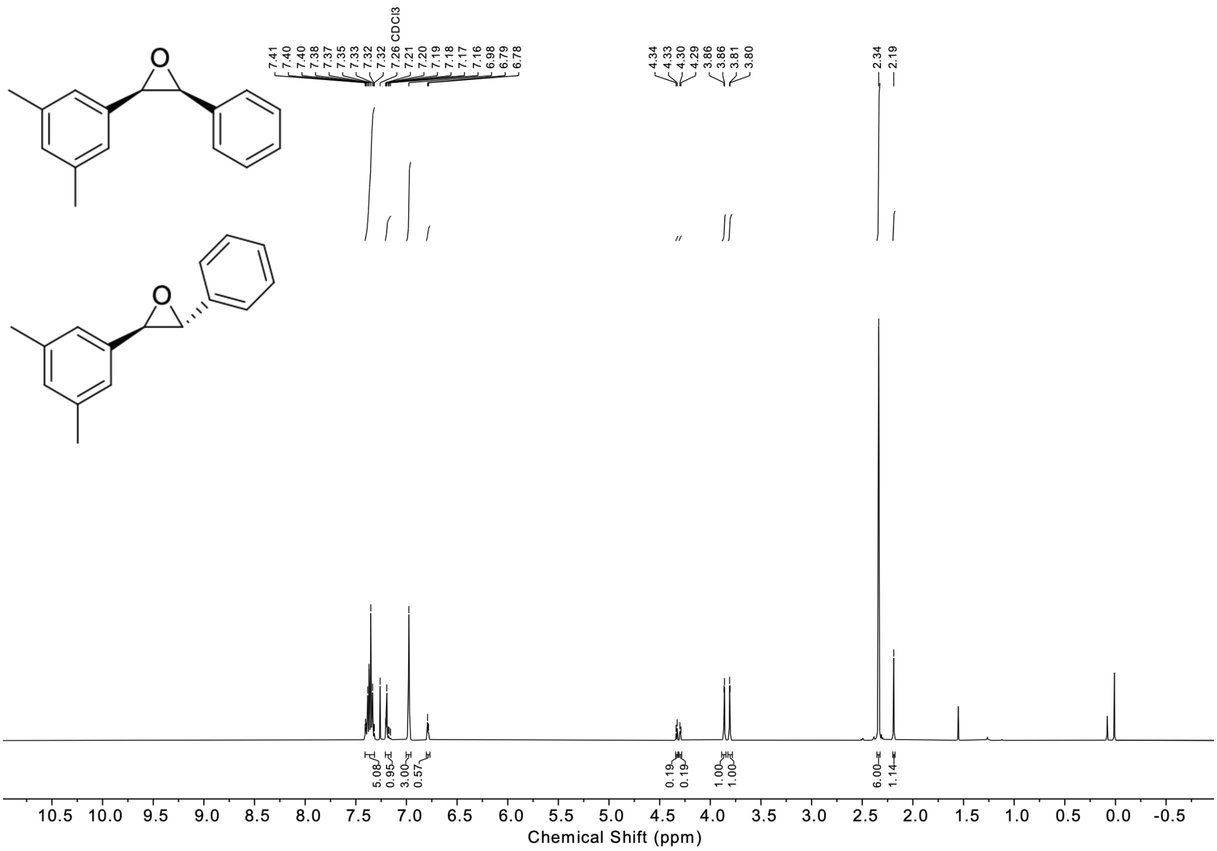

^{13}C{1H}-NMR (101 MHz, $CDCl_3$):

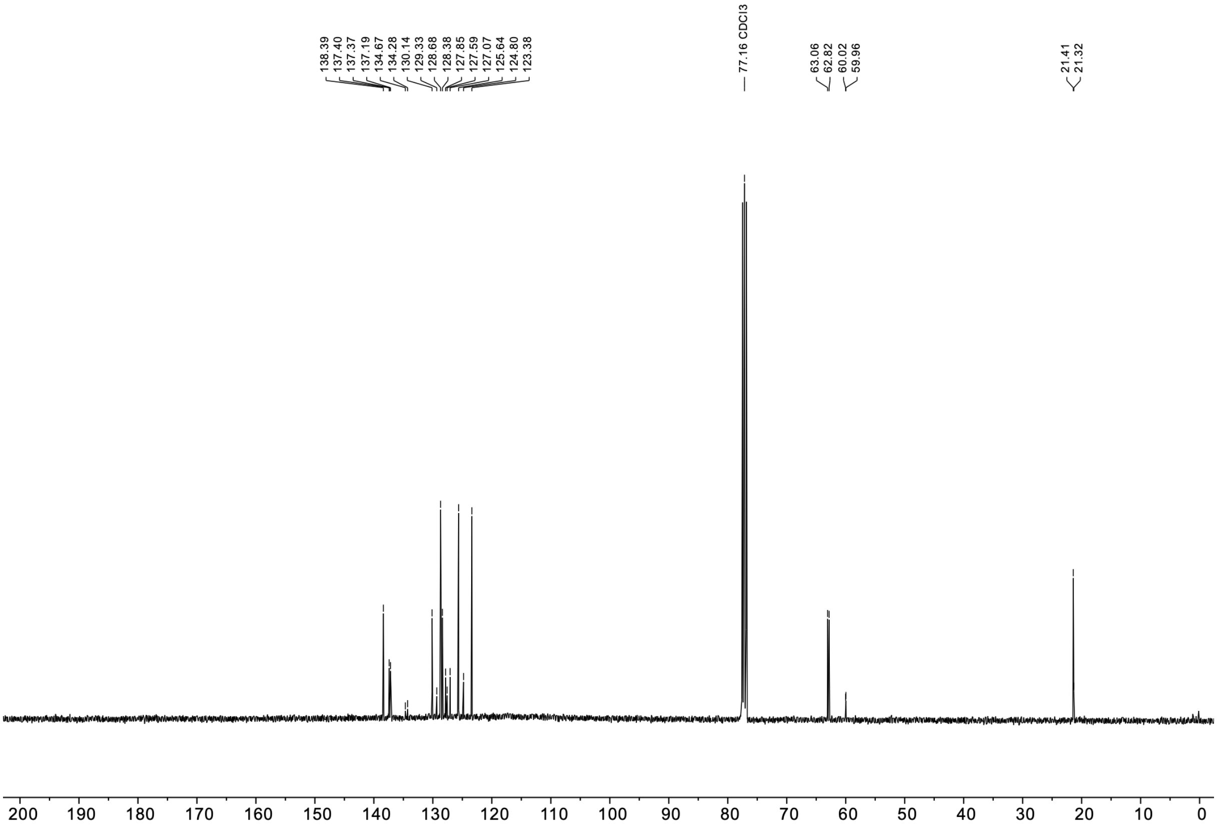

2-(3,5-Di-iso-propylphenyl)-3-phenyl oxirane (**3-H-^{i}Pr**)

^{1}H-NMR (400 MHz, $CDCl_3$):

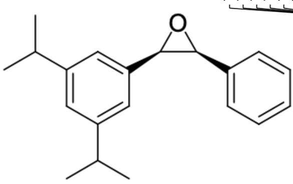

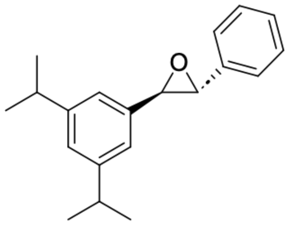

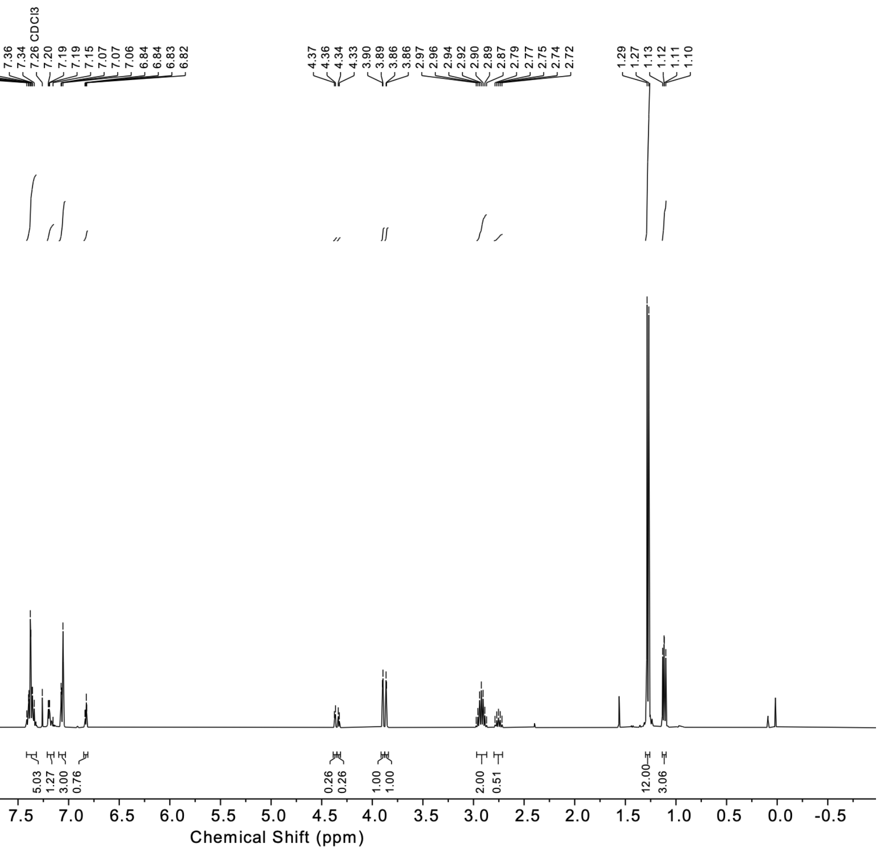

^{13}C{1H}-NMR (101 MHz, $CDCl_3$):

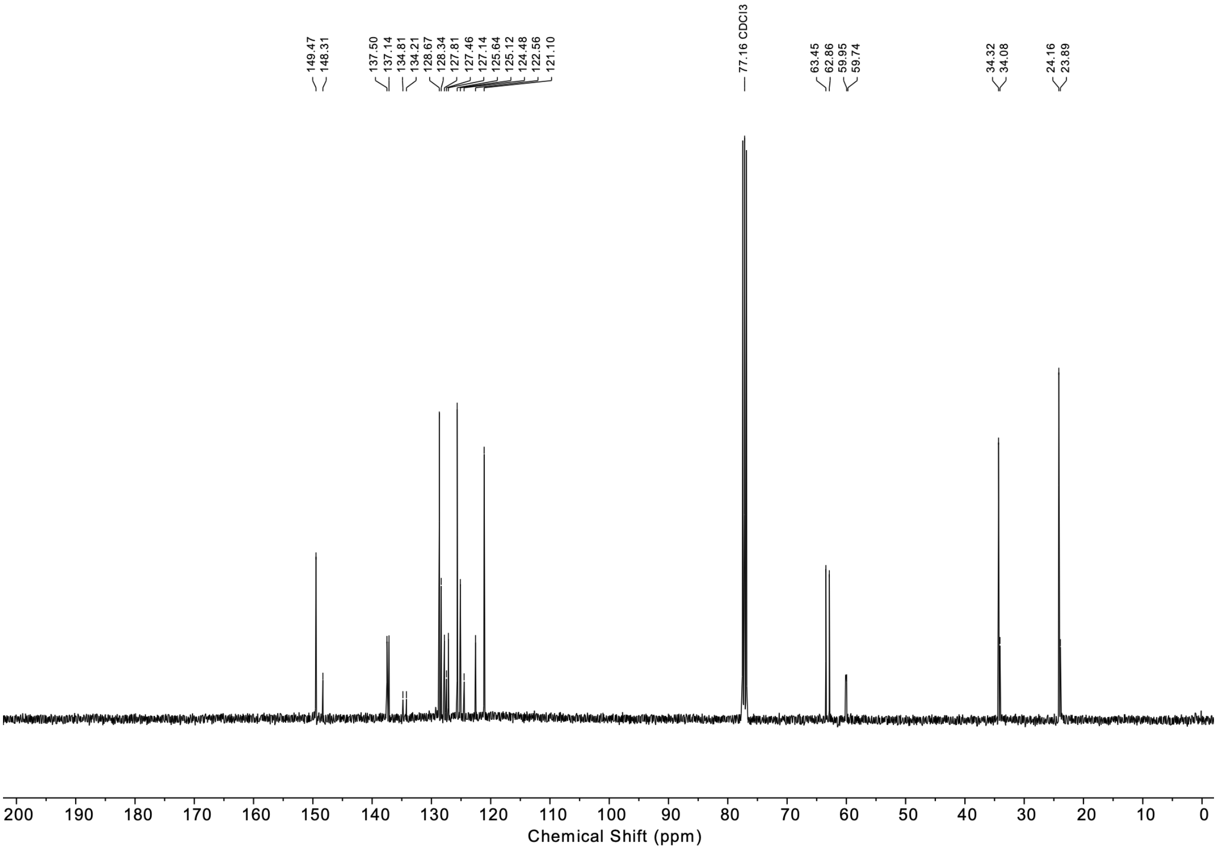

2-(3,5-Di-tert-butylphenyl)-3-phenyl oxirane (**3-H-^{t}Bu**)

^{1}H-NMR (400 MHz, CDCl$_3$):

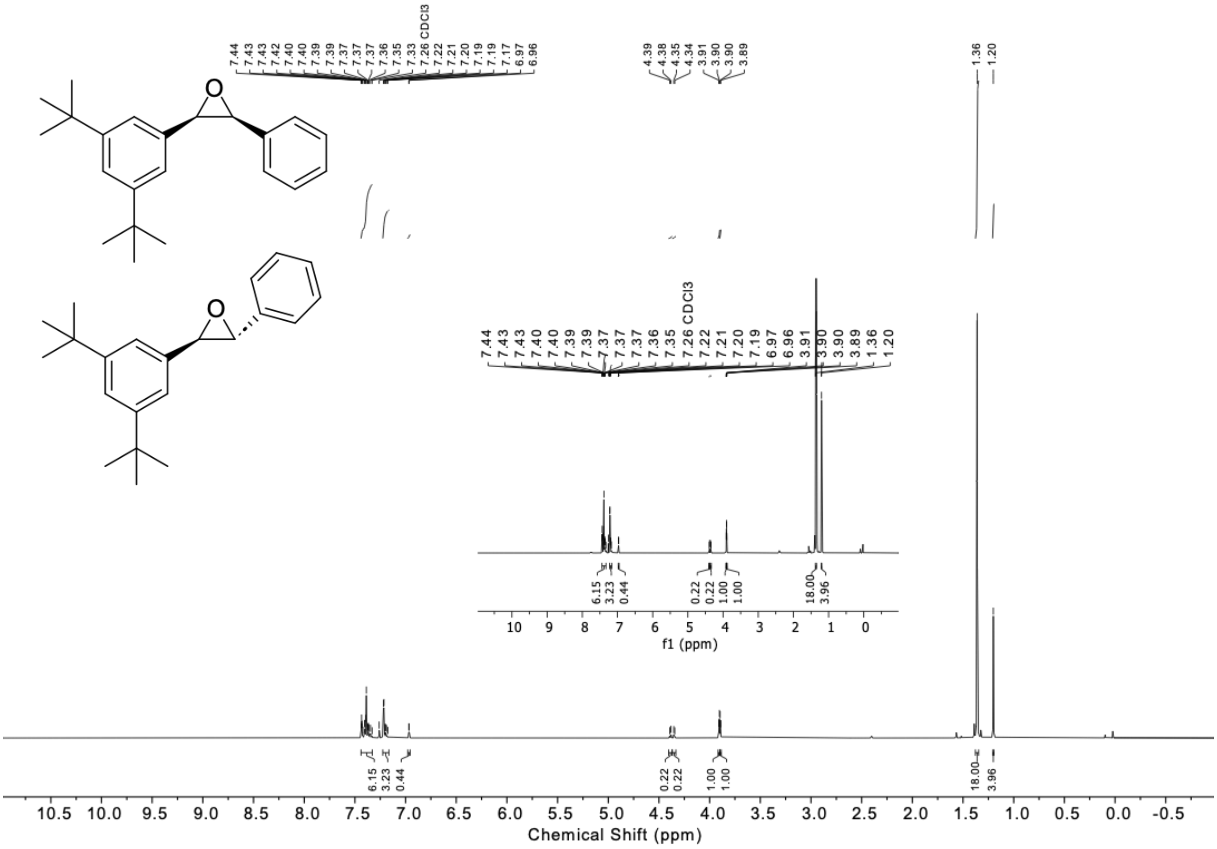

^{13}C{1H}-NMR (101 MHz, CDCl$_3$):

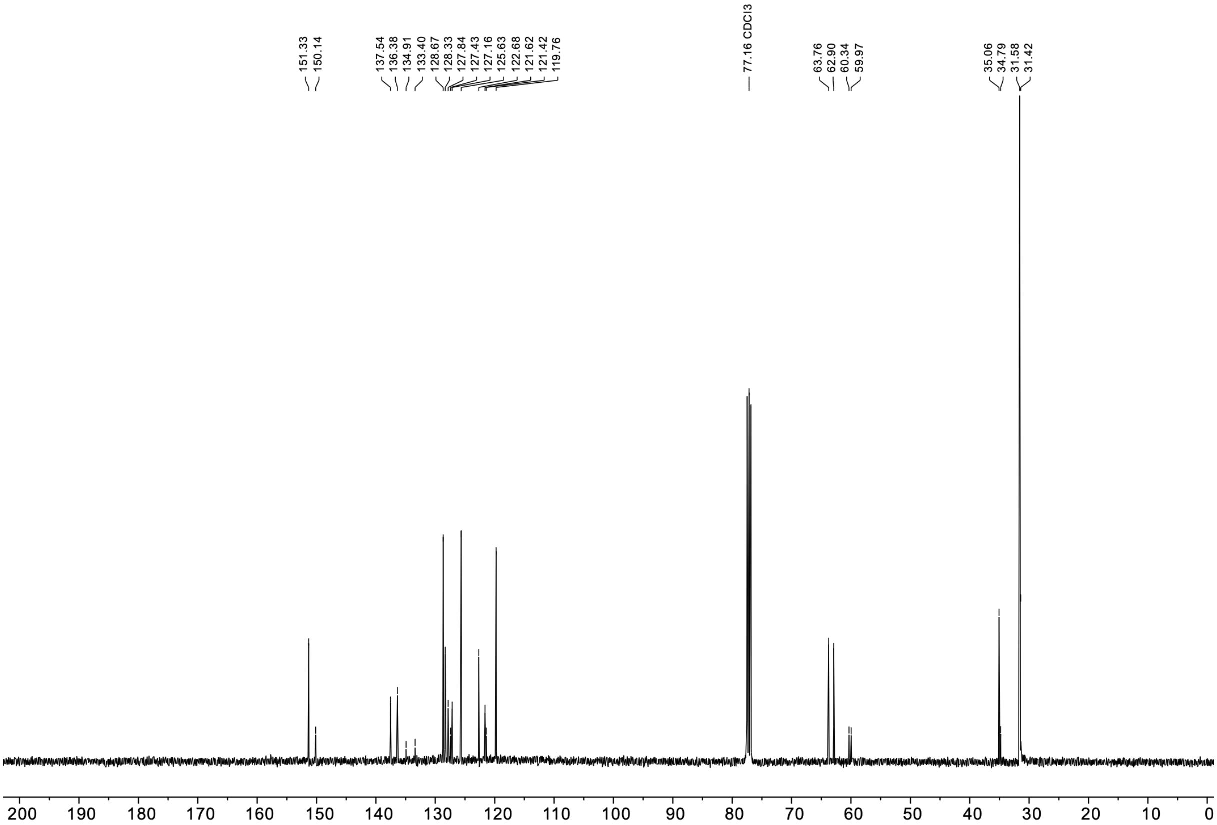

2,3-Di-(3,5-dimethyl)-phenyl oxirane (**3-Me-Me**)

^{1}H-NMR (400 MHz, CDCl$_3$):

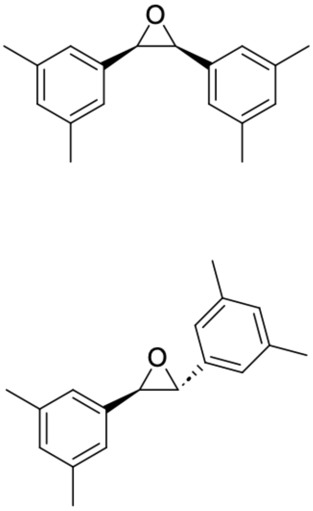

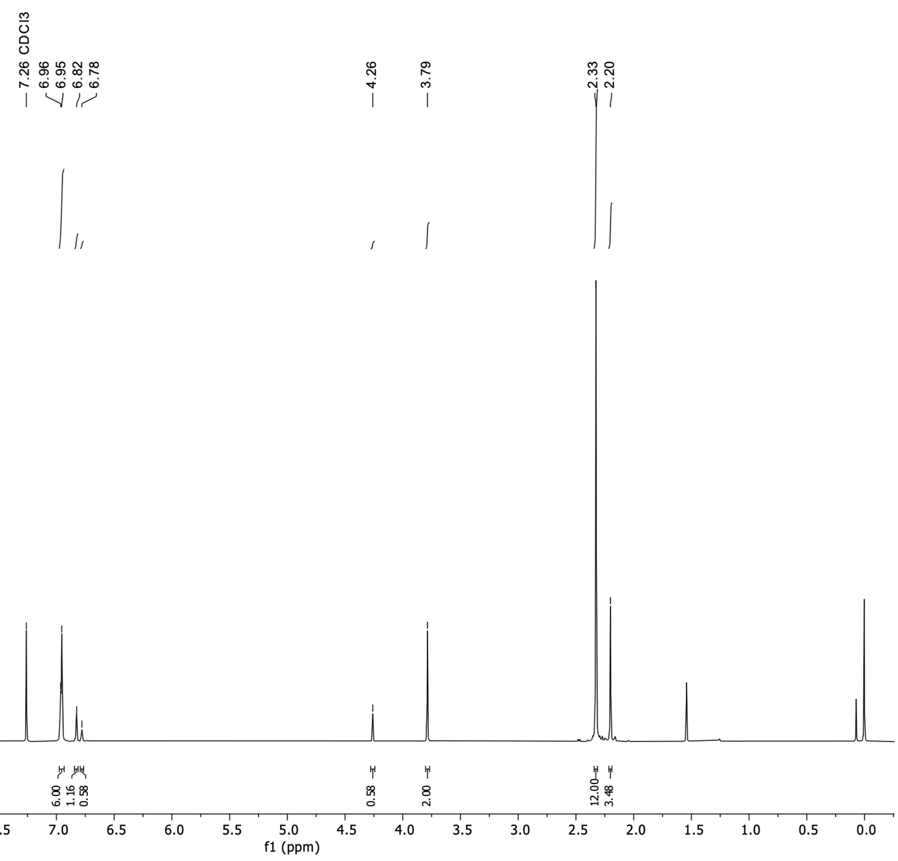

^{13}C{1H}-NMR (101 MHz, CDCl$_3$):

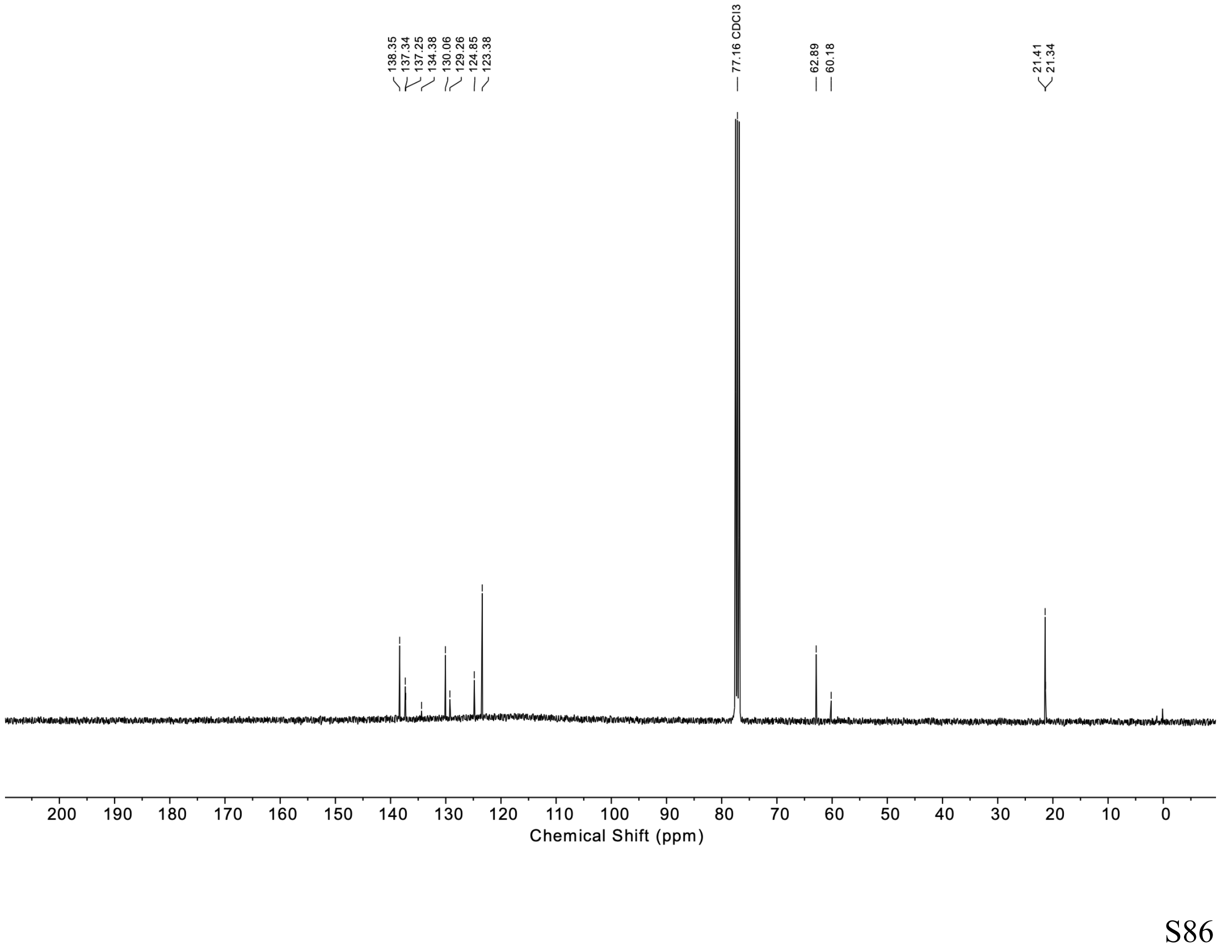

Computational Details

Computed coordinates and energies

2-H
Number of imaginary frequencies: 0
Electronic Energy (SCF) = -345.747923994 a.u
ZPV corrected Energy = -345.638034 a.u.
Enthalpy = -345.630796 a.u.
Free Energy = -345.668594 a.u.

C	-0.262112	2.017399	0.000000
H	0.646344	2.647899	0.000000
C	-0.000000	0.569711	0.000000
C	1.323172	0.123825	0.000000
C	-1.048151	-0.356729	-0.000000
C	1.600699	-1.236551	-0.000000
H	2.128382	0.848053	0.000000
C	-0.769502	-1.712883	-0.000000
H	-2.068656	0.001019	-0.000000
C	0.554442	-2.152981	-0.000000
H	2.625516	-1.582154	-0.000000
H	-1.577292	-2.432235	-0.000000
H	0.767770	-3.213730	-0.000000
O	-1.364169	2.527551	0.000000

ylide-H
Number of imaginary frequencies: 0
Electronic Energy (SCF) = -825.996245273 a.u
ZPV corrected Energy = -825.775548 a.u.
Enthalpy = -825.763500 a.u.
Free Energy = -825.812792 a.u.

S	-1.498492	-1.162531	-0.080142
C	-2.046454	-0.074791	1.320597
H	-1.173530	0.455155	1.693670
H	-2.426726	-0.736719	2.094492
C	-1.983290	0.118305	-1.359905
H	-2.730138	-0.373261	-1.979855
H	-1.099396	0.318938	-1.958480
C	-2.542620	1.331755	-0.628391
H	-3.320314	1.815257	-1.218992
H	-1.752517	2.064274	-0.462796
C	-3.089081	0.852841	0.724421
H	-3.290646	1.689887	1.392835
H	-4.027824	0.314241	0.577731
C	0.133703	-1.507607	-0.055296
H	0.313751	-2.569614	-0.128868
C	1.246619	-0.605221	-0.008918
C	2.560152	-1.134524	-0.009817
C	1.139252	0.802196	0.031087
C	3.675796	-0.316574	0.027104
H	2.686838	-2.209938	-0.041355
C	2.263818	1.615712	0.068541
H	0.166823	1.269502	0.034168
C	3.544861	1.072456	0.067325
H	4.661596	-0.765702	0.024424
H	2.133719	2.690800	0.098418
H	4.417700	1.710391	0.096402

tetrahydrothiophene
Number of imaginary frequencies: 0
Electronic Energy (SCF) = -555.558895271 a.u
ZPV corrected Energy = -555.445455 a.u.
Enthalpy = -555.439364 a.u.
Free Energy = -555.473416 a.u.

S	0.000000	0.000000	1.308816
C	-0.000000	-1.347395	0.050947
H	-0.989101	-1.802770	0.020940
H	0.723592	-2.105703	0.342988
C	0.000000	1.347395	0.050947
H	0.989101	1.802770	0.020940
H	-0.723592	2.105703	0.342988
C	-0.344292	0.681586	-1.278322
H	-0.024540	1.302760	-2.116487
H	-1.425641	0.547308	-1.353717
C	0.344292	-0.681586	-1.278322
H	0.024540	-1.302760	-2.116487
H	1.425641	-0.547308	-1.353717

3-cis-HH
Number of imaginary frequencies: 0
Electronic Energy (SCF) = -616.211845378 a.u
ZPV corrected Energy = -615.991957 a.u.
Enthalpy = -615.979382 a.u.
Free Energy = -616.031621 a.u.

C	0.751109	1.887193	0.148625
H	1.236030	2.707177	0.670826
C	-0.731448	1.893943	0.171785
H	-1.192616	2.717943	0.709557
O	-0.007338	2.296833	-0.994109
C	-1.552393	0.658157	0.105047
C	-2.136418	0.176685	1.278249
C	-1.756197	-0.029965	-1.088297
C	-2.896874	-0.984783	1.260198
H	-1.988180	0.711930	2.208015
C	-2.523857	-1.189813	-1.107111
H	-1.316043	0.347659	-1.999428
C	-3.091973	-1.672996	0.065706
H	-3.338484	-1.353015	2.176828
H	-2.676945	-1.715727	-2.040393
H	-3.685963	-2.577162	0.050563
C	1.559798	0.645671	0.061113
C	2.281563	0.240066	1.185575
C	1.616630	-0.126004	-1.097281
C	3.032281	-0.927548	1.157570
H	2.250700	0.841559	2.085681
C	2.374031	-1.292136	-1.126702
H	1.073029	0.192239	-1.974098
C	3.079623	-1.698980	-0.000285
H	3.582841	-1.234185	2.037184
H	2.412343	-1.882598	-2.032722
H	3.666739	-2.607419	-0.024285

3-trans-HH
Number of imaginary frequencies: 0
Electronic Energy (SCF) = -616.214496228 a.u
ZPV corrected Energy = -615.994825 a.u.
Enthalpy = -615.982126 a.u.
Free Energy = -616.033864 a.u.

C	-0.556366	-0.487894	0.458700
C	0.556366	0.487894	0.458700
O	0.000000	0.000000	1.687044
H	1.542261	0.092383	0.234401

C	0.359572	1.919531	0.127263
C	1.220187	2.533069	-0.783052
C	-0.678627	2.663040	0.690530
C	1.038967	3.864998	-1.137039
H	2.034243	1.963626	-1.213829
C	-0.855424	3.995416	0.340811
H	-1.337875	2.197509	1.410474
C	0.000000	4.599839	-0.576223
H	1.712417	4.329669	-1.845082
H	-1.660629	4.564699	0.786392
H	-0.139386	5.638133	-0.846473
H	-1.542261	-0.092383	0.234401
C	-0.359572	-1.919531	0.127263
C	-1.220187	-2.533069	-0.783052
C	0.678627	-2.663040	0.690530
C	-1.038967	-3.864998	-1.137039
H	-2.034243	-1.963626	-1.213829
C	0.855424	-3.995416	0.340811
H	1.337875	-2.197509	1.410474
C	-0.000000	-4.599839	-0.576223
H	-1.712417	-4.329669	-1.845082
H	1.660629	-4.564699	0.786392
H	0.139386	-5.638133	-0.846473

A-gauche-0-H
Number of imaginary frequencies: 0
Electronic Energy (SCF) = -1171.75206244 a.u
ZPV corrected Energy = -1171.421125 a.u.
Enthalpy = -1171.400576 a.u.
Free Energy = -1171.472950 a.u.

S	-2.248955	0.276685	0.370614
C	-3.847442	0.886059	-0.393383
H	-3.597486	1.786701	-0.948178
H	-4.504759	1.138917	0.439144
C	-2.684403	-1.488370	0.049025
H	-2.465875	-2.048909	0.951684
H	-2.008697	-1.807556	-0.741594
C	-4.142506	-1.524872	-0.384504
H	-4.796748	-1.523419	0.489340
H	-4.340699	-2.436803	-0.947614
C	-4.390892	-0.265621	-1.218498
H	-3.853762	-0.333971	-2.166803
H	-5.448678	-0.127813	-1.443097
C	-0.915786	0.584684	-0.605146
C	0.624967	-1.282411	1.611868
O	-0.160764	-2.130993	1.992339
H	0.578366	-0.255448	2.010463
C	1.718179	-1.518113	0.656269
C	2.645726	-0.500612	0.428031
C	1.846889	-2.741288	-0.010535
C	3.697523	-0.702621	-0.456330
H	2.535023	0.445082	0.940595
C	2.891303	-2.937622	-0.897491
H	1.120757	-3.521065	0.174370
C	3.818801	-1.917974	-1.119676
H	4.413807	0.088010	-0.633663
H	2.990957	-3.880357	-1.418786
H	4.634366	-2.075003	-1.813336
H	-0.929060	0.153796	-1.597562
C	0.035989	1.611745	-0.301319
C	1.045661	1.908984	-1.247406
C	0.085030	2.336059	0.913245
C	2.038815	2.838947	-0.988749
H	1.046048	1.376750	-2.190675
C	1.087732	3.258873	1.166433
H	-0.671045	2.166006	1.668648
C	2.081571	3.523709	0.224699
H	2.794842	3.026263	-1.741755
H	1.090914	3.783788	2.114216
H	2.858180	4.248670	0.426856

A-gauche-TS0-H
Number of imaginary frequencies: 1
Electronic Energy (SCF) = -1171.74333432 a.u
ZPV corrected Energy = -1171.411241 a.u.
Enthalpy = -1171.392313 a.u.
Free Energy = -1171.459406 a.u.

S	2.190589	-0.127487	-0.698845
C	3.317712	0.743493	0.462329
H	2.816864	1.651193	0.788070
H	4.201634	1.008097	-0.117647
C	2.725598	-1.799646	-0.149516
H	3.004655	-2.357157	-1.036673
H	1.844553	-2.259561	0.284444
C	3.866792	-1.592860	0.847609
H	4.819445	-1.561420	0.317152
H	3.898930	-2.431037	1.542931
C	3.631855	-0.261665	1.559336
H	2.789181	-0.337999	2.247782
H	4.504425	0.056591	2.129170
C	0.565847	0.012492	-0.006430
C	-0.444899	-1.112743	-1.355936
O	0.246550	-2.120324	-1.694913
H	-0.532118	-0.263646	-2.060870
C	-1.716781	-1.292202	-0.571822
C	-2.722956	-0.326484	-0.610522
C	-1.910990	-2.435147	0.205005
C	-3.893914	-0.491511	0.121360
H	-2.586557	0.557857	-1.219477
C	-3.077898	-2.602258	0.939336
H	-1.137548	-3.191596	0.214558
C	-4.074309	-1.628734	0.901918
H	-4.665971	0.266220	0.080610
H	-3.216623	-3.493647	1.538192
H	-4.985707	-1.760195	1.470534
H	0.518506	-0.527217	0.933632
C	0.030358	1.370235	0.046744
C	-0.778348	1.746364	1.131980
C	0.191028	2.296008	-0.998611
C	-1.404189	2.984035	1.166611
H	-0.921048	1.048076	1.946394
C	-0.428782	3.537343	-0.955692
H	0.798912	2.041228	-1.857230
C	-1.233182	3.890760	0.124140
H	-2.026761	3.242925	2.013498
H	-0.287056	4.231129	-1.774462
H	-1.717176	4.857678	0.153179

A-gauche-H
Number of imaginary frequencies: 0
Electronic Energy (SCF) = -1171.75657173 a.u
ZPV corrected Energy = -1171.421704 a.u.
Enthalpy = -1171.402921 a.u.
Free Energy = -1171.469648 a.u.

S	-1.856852	0.942203	-0.842105
C	-3.177896	0.084392	0.123635
H	-2.912467	-0.961659	0.242565
H	-4.072737	0.147787	-0.495139
C	-2.065029	2.500683	0.109102
H	-2.157099	3.295738	-0.624041
H	-1.130934	2.636266	0.644023
C	-3.272790	2.334336	1.025960

H	-4.185369	2.615476	0.497921
H	-3.171668	2.990043	1.890670
C	-3.342822	0.859826	1.421278
H	-2.534108	0.612070	2.111241
H	-4.285624	0.611069	1.908478
C	-0.326498	0.147995	-0.149208
C	0.711863	0.778730	-1.140857
O	0.231505	1.998023	-1.473810
H	0.782561	0.073683	-1.996348
C	2.080873	0.759681	-0.463930
C	2.841338	-0.408735	-0.398744
C	2.581793	1.918073	0.124338
C	4.069785	-0.422472	0.252696
H	2.470341	-1.313090	-0.864816
C	3.811558	1.910310	0.775485
H	1.994466	2.822814	0.045717
C	4.558996	0.738623	0.845334
H	4.648436	-1.336682	0.293570
H	4.190578	2.820156	1.224154
H	5.517236	0.731386	1.348453
H	-0.200437	0.596657	0.833917
C	-0.409786	-1.336351	-0.041272
C	-0.214625	-1.954594	1.194150
C	-0.634036	-2.135023	-1.166358
C	-0.233665	-3.340043	1.305868
H	-0.036145	-1.343269	2.069162
C	-0.664340	-3.518484	-1.054752
H	-0.786843	-1.673214	-2.132848
C	-0.460930	-4.125576	0.181413
H	-0.073197	-3.804639	2.269777
H	-0.842563	-4.124286	-1.933322
H	-0.479571	-5.203961	0.266197

A-anti-0-H
Number of imaginary frequencies: 0
Electronic Energy (SCF) = -1171.75268020 a.u
ZPV corrected Energy = -1171.421554 a.u.
Enthalpy = -1171.401256 a.u.
Free Energy = -1171.471495 a.u.

S	2.004579	0.130246	-0.619198
C	3.520208	1.197355	-0.911784
H	3.216048	2.221099	-0.709976
H	3.766046	1.089646	-1.968514
C	3.006305	-1.190320	0.190919
H	2.676809	-2.147255	-0.203498
H	2.759905	-1.131638	1.248698
C	4.471367	-0.876323	-0.074522
H	4.763195	-1.235556	-1.063123
H	5.098865	-1.377657	0.662161
C	4.611698	0.646643	-0.012936
H	4.463891	0.992569	1.012133
H	5.598049	0.977914	-0.338025
C	1.005326	0.728430	0.590814
H	1.432818	0.818131	1.580052
C	-0.437002	-1.768214	1.639996
H	0.529212	-2.234080	1.385095
O	-0.676887	-1.427975	2.783773
C	-0.233113	1.385190	0.290985
C	-0.999602	1.920137	1.352677
C	-0.783696	1.510723	-1.005164
C	-2.228079	2.522949	1.132828
H	-0.615523	1.842666	2.362110
C	-2.016423	2.106037	-1.213509
H	-0.238269	1.128006	-1.857568
C	-2.757320	2.622924	-0.152030
H	-2.781781	2.914724	1.977637
H	-2.405380	2.169033	-2.222573
H	-3.717136	3.091510	-0.322134
C	-1.383796	-1.672354	0.518267
C	-2.648721	-1.106844	0.691162
C	-0.999682	-2.141876	-0.737977
C	-3.519546	-1.017808	-0.383234
H	-2.929564	-0.736237	1.666291
C	-1.871843	-2.053069	-1.813802
H	-0.013286	-2.570133	-0.865171
C	-3.133017	-1.492089	-1.634779
H	-4.495872	-0.571445	-0.252638
H	-1.571787	-2.414598	-2.788218
H	-3.812790	-1.415770	-2.473221

A-anti-TS0-H
Number of imaginary frequencies: 1
Electronic Energy (SCF) = -1171.74144032 a.u
ZPV corrected Energy = -1171.409102 a.u.
Enthalpy = -1171.390211 a.u.
Free Energy = -1171.457617 a.u.

S	1.685440	-0.397940	-0.600216
C	2.945590	0.811136	-1.203602
H	2.482172	1.793871	-1.190598
H	3.161298	0.526049	-2.232317
C	2.904883	-1.358618	0.386524
H	2.737837	-2.414262	0.195231
H	2.693313	-1.140761	1.429694
C	4.282789	-0.857017	-0.038417
H	4.601948	-1.363785	-0.950371
H	5.007652	-1.082880	0.742763
C	4.151859	0.643078	-0.292532
H	3.988840	1.173519	0.646542
H	5.042354	1.057799	-0.763900
C	0.719280	0.399895	0.653743
H	1.384984	0.780770	1.422427
C	-0.283899	-0.847120	1.741359
H	0.547107	-1.562256	1.902730
O	-0.711421	-0.187038	2.740554
C	-0.209401	1.410202	0.127957
C	-0.568466	2.480340	0.959354
C	-0.849600	1.300132	-1.115270
C	-1.530878	3.400678	0.564102
H	-0.091731	2.576834	1.924609
C	-1.800126	2.229109	-1.513811
H	-0.617418	0.474104	-1.774390
C	-2.149990	3.284228	-0.676280
H	-1.794439	4.214840	1.227022
H	-2.278125	2.120601	-2.478765
H	-2.893696	4.005808	-0.987192
C	-1.266961	-1.425433	0.752064
C	-2.545612	-0.883515	0.634473
C	-0.914885	-2.499212	-0.065129
C	-3.447523	-1.392386	-0.292344
H	-2.815768	-0.060508	1.281296
C	-1.812519	-3.011979	-0.994799
H	0.067485	-2.946488	0.036692
C	-3.083143	-2.455210	-1.114150
H	-4.435450	-0.957744	-0.377892
H	-1.526696	-3.849101	-1.619032
H	-3.785726	-2.852913	-1.834969

A-anti-H
Number of imaginary frequencies: 0
Electronic Energy (SCF) = -1171.74494067 a.u
ZPV corrected Energy = -1171.410799 a.u.
Enthalpy = -1171.391821 a.u.
Free Energy = -1171.458575 a.u.

S	1.570209	-0.695637	-0.648056
C	2.884870	0.498415	-1.119824
H	2.476067	1.499263	-1.013600
H	3.092597	0.305335	-2.170722
C	2.723429	-1.804556	0.262368
H	2.502454	-2.827613	-0.026646
H	2.509268	-1.672156	1.319441
C	4.129941	-1.337564	-0.107450
H	4.432022	-1.777451	-1.058843
H	4.833630	-1.668630	0.655316
C	4.077220	0.183088	-0.227097
H	3.939602	0.636754	0.755019
H	4.987563	0.592208	-0.663769
C	0.665465	0.145601	0.726238
H	1.441780	0.415724	1.438353
C	-0.303454	-0.852320	1.555220
H	0.314674	-1.776126	1.673310
O	-0.615288	-0.237663	2.684577
C	-0.006172	1.369981	0.189806
C	0.072546	2.554344	0.923331
C	-0.757487	1.359410	-0.988496
C	-0.592087	3.698947	0.498126
H	0.648350	2.570750	1.838368
C	-1.408893	2.506537	-1.422465
H	-0.847541	0.451134	-1.568056
C	-1.332276	3.679753	-0.678632
H	-0.528407	4.605626	1.085326
H	-1.985727	2.479925	-2.337426
H	-1.845438	4.571234	-1.014142
C	-1.480560	-1.273080	0.667467
C	-2.698401	-0.606001	0.773220
C	-1.367964	-2.311318	-0.255894
C	-3.769965	-0.945805	-0.044352
H	-2.781204	0.179284	1.511882
C	-2.435181	-2.657508	-1.079343
H	-0.440475	-2.868792	-0.325769
C	-3.640470	-1.969077	-0.979989
H	-4.708379	-0.412905	0.045787
H	-2.330488	-3.468238	-1.789476
H	-4.474745	-2.236919	-1.615468

B-gauche-0-H
Number of imaginary frequencies: 0
Electronic Energy (SCF) = -1171.75111575 a.u
ZPV corrected Energy = -1171.420567 a.u.
Enthalpy = -1171.399760 a.u.
Free Energy = -1171.475136 a.u.

S	-2.215439	0.423780	0.283973
C	-3.832266	0.919098	-0.513976
H	-3.556685	1.281514	-1.502656
H	-4.249264	1.736881	0.068846
C	-2.613363	-1.380782	0.121281
H	-2.895299	-1.718762	1.116682
H	-1.693688	-1.872516	-0.176688
C	-3.763166	-1.497721	-0.864623
H	-4.266662	-2.458427	-0.756675
H	-3.388515	-1.427796	-1.887711
C	-4.702768	-0.325494	-0.571367
H	-5.473500	-0.219549	-1.335197
H	-5.202564	-0.482770	0.386408
C	-0.932647	0.782453	-0.731339
C	0.654218	-1.147657	1.750415
O	-0.198809	-1.881632	2.212397
H	0.746624	-0.106381	2.102004
C	1.636920	-1.536719	0.728496
C	1.610777	-2.807474	0.143560
C	2.609210	-0.612231	0.343830
C	2.547952	-3.143442	-0.818006
H	0.852415	-3.514096	0.451827
C	3.551135	-0.953125	-0.617964
H	2.616648	0.370806	0.794104
C	3.519117	-2.215591	-1.198912
H	2.529786	-4.123773	-1.275081
H	4.301091	-0.234522	-0.919068
H	4.249629	-2.481264	-1.951680
H	-0.972235	0.391777	-1.739493
C	0.111671	1.694945	-0.371469
C	1.136015	1.964339	-1.310805
C	0.228017	2.344971	0.880319
C	2.201594	2.797680	-1.012874
H	1.088418	1.486975	-2.281933
C	1.301166	3.171786	1.171313
H	-0.532382	2.190873	1.634706
C	2.307338	3.409863	0.234960
H	2.964979	2.965394	-1.763221
H	1.352136	3.640911	2.146678
H	3.141048	4.057903	0.468366

B-gauche-TS0-H
Number of imaginary frequencies: 1
Electronic Energy (SCF) = -1171.74365408 a.u
ZPV corrected Energy = -1171.411732 a.u.
Enthalpy = -1171.392761 a.u.
Free Energy = -1171.460448 a.u.

S	-2.217625	-0.040204	0.687332
C	-3.308752	0.820026	-0.540341
H	-2.638466	1.252736	-1.278750
H	-3.835256	1.615967	-0.020581
C	-2.796532	-1.709433	0.214585
H	-3.527139	-1.991640	0.970703
H	-1.931229	-2.360832	0.296740
C	-3.426925	-1.557201	-1.161535
H	-4.060857	-2.416815	-1.377791
H	-2.651507	-1.510585	-1.927761
C	-4.222701	-0.253509	-1.128845
H	-4.558582	0.049527	-2.120112
H	-5.104817	-0.375739	-0.498799
C	-0.578764	-0.001713	0.023310
C	0.414946	-1.150148	1.367117
O	-0.267905	-2.171203	1.686169
H	0.497900	-0.318303	2.091821
C	1.689676	-1.309219	0.582328
C	1.865630	-2.405299	-0.263523
C	2.715105	-0.369691	0.686862
C	3.034007	-2.551094	-0.999670
H	1.076133	-3.142267	-0.328706
C	3.889139	-0.514864	-0.044715
H	2.590973	0.479870	1.346095
C	4.051154	-1.604087	-0.894097
H	3.157364	-3.404869	-1.654087
H	4.676362	0.222347	0.048173
H	4.963771	-1.719394	-1.464249
H	-0.551588	-0.555877	-0.909891
C	0.012430	1.334341	-0.053316
C	0.835423	1.661602	-1.142622
C	-0.116622	2.282485	0.975577
C	1.505063	2.875613	-1.197670
H	0.955421	0.943900	-1.943778
C	0.547485	3.499778	0.913104
H	-0.734611	2.063624	1.836930
C	1.365191	3.805454	-0.171385
H	2.138104	3.097257	-2.047320
H	0.429781	4.212047	1.719705

H	1.883775	4.753682	-0.215983

B-gauche-H
Number of imaginary frequencies: 0
Electronic Energy (SCF) = -1171.75592095 a.u
ZPV corrected Energy = -1171.421183 a.u.
Enthalpy = -1171.402340 a.u.
Free Energy = -1171.469033 a.u.

S	1.894017	-0.846067	-0.771489
C	3.163092	0.103604	0.187584
H	2.658292	0.681853	0.957638
H	3.658917	0.783849	-0.499481
C	2.202371	-2.391427	0.169872
H	2.636576	-3.074447	-0.557933
H	1.229319	-2.768847	0.460489
C	3.159041	-2.056650	1.305366
H	3.709409	-2.947632	1.606866
H	2.598694	-1.699094	2.170932
C	4.080325	-0.951145	0.793967
H	4.687897	-0.516208	1.587209
H	4.757054	-1.348897	0.034863
C	0.313099	-0.147967	-0.107424
C	-0.680855	-0.826319	-1.116629
O	-0.156632	-2.030107	-1.430871
H	-0.753958	-0.127300	-1.977597
C	-2.064786	-0.845031	-0.469601
C	-2.536152	-2.010932	0.128261
C	-2.869561	0.294927	-0.442812
C	-3.779879	-2.037921	0.751715
H	-1.914964	-2.894879	0.078411
C	-4.112495	0.273936	0.180389
H	-2.521822	1.204309	-0.916950
C	-4.571784	-0.894068	0.783247
H	-4.135161	-2.953407	1.208268
H	-4.725495	1.166358	0.191202
H	-5.541169	-0.914127	1.264183
H	0.197897	-0.604698	0.874191
C	0.319013	1.339697	-0.000985
C	0.009084	1.950478	1.214387
C	0.572881	2.145054	-1.114543
C	-0.056150	3.335275	1.316510
H	-0.193162	1.333273	2.080130
C	0.520259	3.528595	-1.011511
H	0.811147	1.688757	-2.066466
C	0.201857	4.128097	0.203835
H	-0.305550	3.793807	2.264272
H	0.723083	4.139887	-1.880902
H	0.155630	5.206169	0.281709

B-anti-0-H
Number of imaginary frequencies: 0
Electronic Energy (SCF) = -1171.75122957 a.u
ZPV corrected Energy = -1171.420182 a.u.
Enthalpy = -1171.399739 a.u.
Free Energy = -1171.470856 a.u.

S	2.112940	0.338027	-0.652823
C	3.735857	1.244128	-0.490056
H	3.642398	1.825843	0.425239
H	3.820840	1.917011	-1.339687
C	2.894939	-1.277627	-0.169496
H	2.981774	-1.855395	-1.089155
H	2.201398	-1.769999	0.503236
C	4.254469	-0.958459	0.428465
H	4.901926	-1.835072	0.410457
H	4.140321	-0.646404	1.468399
C	4.828956	0.192975	-0.401011
H	5.727024	0.612684	0.052581
H	5.094489	-0.164054	-1.397988
C	1.052921	0.855667	0.534511
H	1.420037	0.901881	1.550519
C	-0.570587	-1.620285	1.792912
H	0.410906	-2.099007	1.634023
O	-0.865900	-1.168948	2.882285
C	-1.450918	-1.627156	0.615229
C	-0.984075	-2.181449	-0.577175
C	-2.730767	-1.071580	0.670228
C	-1.790709	-2.186837	-1.706393
H	0.014501	-2.598221	-0.613503
C	-3.536177	-1.078283	-0.456889
H	-3.073887	-0.632608	1.595801
C	-3.067691	-1.636952	-1.644299
H	-1.427773	-2.612983	-2.631981
H	-4.524504	-0.641149	-0.418363
H	-3.696564	-1.635177	-2.524863
C	-0.221713	1.432777	0.212458
C	-1.005173	1.985714	1.251750
C	-0.785004	1.473365	-1.083863
C	-2.251848	2.542049	1.009896
H	-0.617660	1.965244	2.262554
C	-2.030652	2.031087	-1.315491
H	-0.239632	1.050395	-1.916766
C	-2.784439	2.575986	-0.276588
H	-2.815917	2.950737	1.839795
H	-2.425269	2.031902	-2.324427
H	-3.758580	3.006800	-0.464123

B-anti-TS0-H
Number of imaginary frequencies: 1
Electronic Energy (SCF) = -1171.74073137 a.u
ZPV corrected Energy = -1171.408540 a.u.
Enthalpy = -1171.389665 a.u.
Free Energy = -1171.456939 a.u.

S	1.628383	-0.421663	-0.578271
C	2.906519	0.771824	-1.200687
H	2.698622	1.712819	-0.697313
H	2.756237	0.890253	-2.270191
C	2.841249	-1.400714	0.384952
H	3.038913	-2.292638	-0.206514
H	2.372876	-1.687305	1.321105
C	4.077291	-0.522090	0.525780
H	4.940221	-1.133044	0.788833
H	3.925940	0.208801	1.320976
C	4.260156	0.182326	-0.816594
H	5.007291	0.973911	-0.766151
H	4.581926	-0.535743	-1.572179
C	0.694526	0.414613	0.676401
H	1.376365	0.781718	1.438185
C	-0.386846	-0.764123	1.779551
H	0.439550	-1.450478	2.050913
O	-0.889503	-0.039728	2.694313
C	-1.289219	-1.423193	0.765341
C	-0.874648	-2.555117	0.062997
C	-2.555868	-0.901014	0.508764
C	-1.698369	-3.144505	-0.889142
H	0.097508	-2.986745	0.272713
C	-3.383508	-1.486047	-0.442388
H	-2.877008	-0.034157	1.068966
C	-2.956498	-2.607002	-1.148485
H	-1.365118	-4.025751	-1.422511
H	-4.362719	-1.066535	-0.635847
H	-3.601603	-3.064358	-1.887411
C	-0.189963	1.456178	0.135749

C	-0.504255	2.552646	0.950841
C	-0.833561	1.354890	-1.106643
C	-1.425547	3.507619	0.540384
H	-0.024370	2.643694	1.914932
C	-1.743926	2.317309	-1.520032
H	-0.634783	0.511405	-1.754408
C	-2.048751	3.398816	-0.698720
H	-1.654687	4.342128	1.190690
H	-2.225155	2.215178	-2.484098
H	-2.760787	4.146782	-1.021271

B-anti-H
Number of imaginary frequencies: 0
Electronic Energy (SCF) = -1171.74463907 a.u
ZPV corrected Energy = -1171.410674 a.u.
Enthalpy = -1171.391678 a.u.
Free Energy = -1171.458459 a.u.

S	1.526156	-0.796333	-0.628123
C	2.845665	0.389709	-1.132815
H	2.695258	1.285500	-0.535295
H	2.693848	0.626138	-2.181772
C	2.684019	-1.905499	0.262427
H	2.827264	-2.765164	-0.389416
H	2.198655	-2.227866	1.178543
C	3.966427	-1.105989	0.455078
H	4.797610	-1.781373	0.654353
H	3.863028	-0.435938	1.309264
C	4.170398	-0.303634	-0.827089
H	4.957714	0.442531	-0.725542
H	4.441148	-0.970434	-1.646776
C	0.655524	0.071679	0.755783
H	1.439911	0.283447	1.479021
C	-0.390279	-0.862101	1.557039
H	0.165548	-1.822402	1.692796
O	-0.688807	-0.224432	2.678415
C	-1.573652	-1.215028	0.648329
C	-1.508992	-2.264790	-0.266563
C	-2.751217	-0.476288	0.731232
C	-2.583057	-2.553747	-1.103078
H	-0.615133	-2.876556	-0.318547
C	-3.828942	-0.757894	-0.100234
H	-2.798092	0.316907	1.464610
C	-3.746883	-1.794534	-1.026428
H	-2.516659	-3.375125	-1.805547
H	-4.735468	-0.169932	-0.027493
H	-4.586523	-2.018076	-1.671898
C	0.076947	1.345694	0.223931
C	0.234946	2.518435	0.962855
C	-0.660020	1.393594	-0.962196
C	-0.338953	3.710165	0.534767
H	0.802687	2.489830	1.882629
C	-1.221560	2.586543	-1.398448
H	-0.809052	0.495923	-1.546444
C	-1.066636	3.748611	-0.649347
H	-0.213974	4.608410	1.125072
H	-1.789082	2.604987	-2.319398
H	-1.509148	4.676419	-0.986967

C-gauche-0-H
Number of imaginary frequencies: 0
Electronic Energy (SCF) = -1171.75216714 a.u
ZPV corrected Energy = -1171.420669 a.u.
Enthalpy = -1171.400203 a.u.
Free Energy = -1171.472580 a.u.

S	-0.058084	1.332361	-0.854651
C	-1.292069	2.674370	-1.291648
H	-2.156306	2.180521	-1.726323
H	-0.799467	3.271544	-2.056259
C	0.022863	1.878228	0.914517
H	1.038625	1.695068	1.249986
H	-0.662749	1.262582	1.489447
C	-0.369756	3.344239	0.897702
H	0.456528	3.948178	0.516119
H	-0.595214	3.693499	1.905310
C	-1.585819	3.474395	-0.029195
H	-2.467867	3.073514	0.470434
H	-1.789746	4.517255	-0.270667
C	-0.655264	-0.215107	-1.047013
C	1.097234	-1.606798	1.160482
H	0.532647	-2.360702	0.588209
O	0.699221	-1.228509	2.244805
C	2.351120	-1.159363	0.525569
C	2.714136	-1.694984	-0.710881
C	3.172895	-0.210463	1.139124
C	3.881463	-1.280448	-1.336517
H	2.071379	-2.429795	-1.179227
C	4.337036	0.205643	0.512984
H	2.885215	0.191440	2.100745
C	4.690736	-0.326839	-0.726018
H	4.159972	-1.694059	-2.296358
H	4.972383	0.943322	0.984722
H	5.599028	0.001489	-1.213818
H	-0.009381	-0.806599	-1.676085
C	-1.875966	-0.783237	-0.560427
C	-2.162555	-2.138377	-0.855151
C	-2.834628	-0.093889	0.213266
C	-3.318594	-2.753223	-0.407320
H	-1.453809	-2.702189	-1.449516
C	-3.991348	-0.718559	0.659371
H	-2.678523	0.942127	0.470856
C	-4.251076	-2.052101	0.358485
H	-3.496484	-3.792343	-0.656454
H	-4.699627	-0.151625	1.251366
H	-5.153479	-2.533851	0.709518

C-gauche-TS0-H
Number of imaginary frequencies: 1
Electronic Energy (SCF) = -1171.74027755 a.u
ZPV corrected Energy = -1171.407731 a.u.
Enthalpy = -1171.388924 a.u.
Free Energy = -1171.455404 a.u.

S	0.184680	1.112471	-0.865964
C	-1.242885	2.061598	-1.553565
H	-1.942064	1.341113	-1.969487
H	-0.827197	2.667233	-2.356800
C	0.238098	2.076477	0.703269
H	1.282820	2.306761	0.886553
H	-0.113720	1.421673	1.492970
C	-0.618615	3.315445	0.461057
H	-0.026817	4.079815	-0.045365
H	-0.945554	3.727515	1.415328
C	-1.801653	2.910038	-0.418017
H	-2.518708	2.328175	0.157707
H	-2.317278	3.781588	-0.820726
C	-0.306707	-0.546782	-0.527818
C	0.786504	-1.228942	0.978229
H	0.472773	-2.263087	0.735015
O	0.425255	-0.729775	2.084791
C	2.187296	-0.971589	0.459283
C	2.694263	-1.684131	-0.629155
C	3.002355	-0.028082	1.080614
C	3.975061	-1.438388	-1.105255

H	2.076973	-2.437262	-1.104821
C	4.285286	0.224293	0.605459
H	2.614952	0.495559	1.943390
C	4.774654	-0.475427	-0.492529
H	4.354096	-1.998193	-1.950788
H	4.907004	0.962292	1.096624
H	5.773405	-0.282545	-0.861813
H	0.072068	-1.104279	-1.378757
C	-1.714588	-0.858732	-0.236134
C	-2.364831	-1.831425	-1.010685
C	-2.419422	-0.300218	0.839052
C	-3.667614	-2.222212	-0.732633
H	-1.835791	-2.279994	-1.842326
C	-3.726290	-0.685283	1.110351
H	-1.940785	0.420868	1.480319
C	-4.359613	-1.645600	0.328188
H	-4.144821	-2.973313	-1.348903
H	-4.249103	-0.238115	1.946114
H	-5.376959	-1.943372	0.544600

C-gauche-H
Number of imaginary frequencies: 0
Electronic Energy (SCF) = -1171.75494129 a.u
ZPV corrected Energy = -1171.419784 a.u.
Enthalpy = -1171.401223 a.u.
Free Energy = -1171.466311 a.u.

S	0.127690	1.510999	0.103967
C	1.129319	1.886465	1.619135
H	1.009259	1.081333	2.338695
H	0.693508	2.793387	2.035520
C	1.472375	2.004533	-1.026781
H	0.997125	2.554909	-1.831965
H	1.900493	1.093315	-1.430798
C	2.474843	2.800453	-0.200015
H	2.132224	3.829758	-0.080009
H	3.436675	2.825324	-0.712113
C	2.565802	2.115143	1.162507
H	3.081465	1.160146	1.063480
H	3.109984	2.719346	1.888292
C	-0.002558	-0.349607	0.216981
C	-1.029511	-0.542784	-0.942400
H	-0.884686	-1.581355	-1.300139
O	-0.743954	0.403212	-1.872666
C	-2.455036	-0.494592	-0.378099
C	-2.922517	-1.486605	0.486316
C	-3.308985	0.546732	-0.723960
C	-4.212730	-1.434359	0.999477
H	-2.271185	-2.310910	0.755208
C	-4.603468	0.605251	-0.213244
H	-2.935164	1.299292	-1.404366
C	-5.059390	-0.383516	0.651532
H	-4.561916	-2.212686	1.666316
H	-5.257196	1.422475	-0.491820
H	-6.065763	-0.341550	1.047576
H	-0.399077	-0.508783	1.219652
C	1.300853	-1.066159	0.078298
C	1.983078	-1.489033	1.220930
C	1.853277	-1.341678	-1.176541
C	3.197430	-2.156800	1.118365
H	1.554886	-1.296586	2.196119
C	3.066054	-2.010616	-1.279168
H	1.320362	-1.024310	-2.062291
C	3.745035	-2.414985	-0.133440
H	3.712072	-2.478699	2.014088
H	3.482234	-2.217936	-2.256264
H	4.690047	-2.935224	-0.216676

C-gauche'-0-H
Number of imaginary frequencies: 0
Electronic Energy (SCF) = -1171.75302888 a.u
ZPV corrected Energy = -1171.421648 a.u.
Enthalpy = -1171.401149 a.u.
Free Energy = -1171.473346 a.u.

S	-2.011533	-1.013711	0.892600
C	-3.615839	-0.088457	1.170394
H	-3.352365	0.908956	1.510107
H	-4.112551	-0.621900	1.978039
C	-2.448309	-1.392278	-0.870608
H	-2.009342	-2.360253	-1.094420
H	-1.997507	-0.631578	-1.502329
C	-3.964931	-1.363846	-0.915095
H	-4.369418	-2.266750	-0.452566
H	-4.320233	-1.334112	-1.945118
C	-4.412214	-0.124653	-0.127925
H	-4.207590	0.771532	-0.714048
H	-5.482489	-0.148860	0.075124
C	-0.653109	-0.055430	1.064780
C	0.988485	-1.965700	-0.918714
H	0.391549	-1.357340	-1.618610
O	0.619963	-3.079135	-0.595784
C	2.244837	-1.332203	-0.488443
C	2.604520	-0.104979	-1.046057
C	3.078843	-1.938883	0.456475
C	3.793048	0.509703	-0.673968
H	1.947069	0.364495	-1.766155
C	4.257861	-1.319731	0.834664
H	2.786933	-2.887947	0.884851
C	4.617179	-0.095952	0.267011
H	4.068251	1.462300	-1.105543
H	4.902373	-1.783218	1.569614
H	5.540064	0.384317	0.564505
H	0.039254	-0.484325	1.772511
C	-0.389892	1.244407	0.526204
C	0.795577	1.914894	0.912829
C	-1.225107	1.924091	-0.387524
C	1.118987	3.166154	0.419549
H	1.465040	1.425063	1.608321
C	-0.892443	3.179365	-0.878726
H	-2.143220	1.467722	-0.723832
C	0.280300	3.817767	-0.485738
H	2.039041	3.639232	0.740533
H	-1.561652	3.663949	-1.579388
H	0.534849	4.795561	-0.871566

C-gauche'-TS0-H
Number of imaginary frequencies: 1
Electronic Energy (SCF) = -1171.74042217 a.u
ZPV corrected Energy = -1171.407658 a.u.
Enthalpy = -1171.388876 a.u.
Free Energy = -1171.455178 a.u.

S	-1.919987	-1.163010	0.758426
C	-3.202110	0.073292	1.244821
H	-2.693908	0.978214	1.565146
H	-3.719883	-0.369036	2.092800
C	-2.630418	-1.442170	-0.911467
H	-2.467725	-2.488331	-1.145895
H	-2.093937	-0.828043	-1.626627
C	-4.091114	-1.035749	-0.769205
H	-4.646752	-1.816435	-0.246287
H	-4.538954	-0.913466	-1.754974
C	-4.119412	0.263526	0.037370
H	-3.752927	1.087294	-0.573724

H	-5.127195	0.511474	0.368070
C	-0.336631	-0.381909	0.635012
C	0.567706	-1.567545	-0.681270
H	0.164292	-1.020457	-1.555946
O	0.213460	-2.771255	-0.490182
C	1.991616	-1.148281	-0.415957
C	2.542288	-0.036299	-1.051797
C	2.780731	-1.872879	0.477360
C	3.852934	0.351544	-0.795698
H	1.938413	0.529478	-1.749934
C	4.089296	-1.487280	0.738519
H	2.351811	-2.743432	0.955645
C	4.630230	-0.371133	0.103558
H	4.266964	1.217129	-1.296845
H	4.691908	-2.057078	1.434634
H	5.650748	-0.071896	0.304283
H	0.172452	-0.678886	1.546654
C	-0.211653	1.050244	0.352837
C	0.694246	1.805137	1.116096
C	-0.851392	1.695147	-0.716474
C	0.950105	3.136817	0.825263
H	1.209129	1.327266	1.939402
C	-0.603989	3.032294	-0.998999
H	-1.539519	1.153644	-1.346723
C	0.299051	3.763042	-0.233755
H	1.658811	3.688524	1.429395
H	-1.114205	3.502664	-1.829809
H	0.493685	4.802789	-0.459906

C-gauche'-H
Number of imaginary frequencies: 0
Electronic Energy (SCF) = -1171.75389369 a.u
ZPV corrected Energy = -1171.418472 a.u.
Enthalpy = -1171.399878 a.u.
Free Energy = -1171.465335 a.u.

S	-1.396419	-1.694826	0.617973
C	-2.822840	-0.739702	1.315385
H	-2.448165	0.098233	1.896982
H	-3.321903	-1.436048	1.988060
C	-2.190543	-1.874060	-1.020953
H	-2.017359	-2.899576	-1.329123
H	-1.671019	-1.212420	-1.706814
C	-3.659159	-1.499833	-0.848691
H	-4.215769	-2.355591	-0.462950
H	-4.088045	-1.231712	-1.814267
C	-3.721574	-0.344434	0.149641
H	-3.351034	0.569524	-0.311848
H	-4.739564	-0.159011	0.492540
C	-0.074778	-0.380768	0.455955
C	0.850962	-1.103003	-0.576709
H	0.571113	-0.711797	-1.576310
O	0.641273	-2.432577	-0.435388
C	2.284616	-0.627143	-0.334257
C	2.745919	0.585994	-0.845265
C	3.151562	-1.405827	0.428392
C	4.044229	1.016884	-0.592607
H	2.085690	1.195730	-1.449484
C	4.451047	-0.980220	0.683417
H	2.787476	-2.353319	0.801775
C	4.901982	0.234820	0.175617
H	4.388642	1.959498	-0.999029
H	5.115210	-1.598274	1.274751
H	5.914254	0.565393	0.369175
H	0.381173	-0.454284	1.443730
C	-0.521953	1.023556	0.221363
C	-0.534422	1.923275	1.290462
C	-0.898692	1.488640	-1.042589
C	-0.919601	3.245816	1.110055
H	-0.231717	1.579339	2.271251
C	-1.283753	2.809982	-1.225943
H	-0.885007	0.821845	-1.891269
C	-1.298794	3.692678	-0.150713
H	-0.919354	3.926238	1.951276
H	-1.570257	3.151761	-2.211753
H	-1.597108	4.722370	-0.296522

C-anti-0-H
Number of imaginary frequencies: 0
Electronic Energy (SCF) = -1171.75373809 a.u
ZPV corrected Energy = -1171.422264 a.u.
Enthalpy = -1171.401876 a.u.
Free Energy = -1171.472565 a.u.

S	-2.362112	-0.124330	1.108742
C	-3.532364	0.916256	0.079293
H	-3.078810	1.898649	-0.015332
H	-4.438530	0.996106	0.676218
C	-2.251231	-1.427986	-0.206742
H	-2.140099	-2.378516	0.308226
H	-1.364955	-1.233445	-0.802426
C	-3.535537	-1.300572	-1.005240
H	-4.370557	-1.722933	-0.442099
H	-3.460950	-1.848434	-1.944636
C	-3.767388	0.197807	-1.242650
H	-3.065272	0.560604	-1.993440
H	-4.774394	0.387712	-1.613281
C	-0.913239	0.639954	1.438387
C	1.427380	-1.493836	2.011952
O	1.990463	-0.872658	2.892151
H	-0.724859	0.672448	2.500248
C	-0.064270	1.379928	0.552600
C	1.010249	2.121784	1.097412
C	-0.206427	1.419382	-0.850584
C	1.875320	2.846050	0.295805
H	1.156061	2.112691	2.170045
C	0.667777	2.144256	-1.647697
H	-1.000117	0.867734	-1.329722
C	1.718020	2.866722	-1.089768
H	2.686409	3.397170	0.755996
H	0.526822	2.139493	-2.721695
H	2.397460	3.428996	-1.715879
H	0.574190	-2.153999	2.248800
C	1.811371	-1.476996	0.591836
C	1.158460	-2.331532	-0.296566
C	2.801912	-0.611288	0.122186
C	1.487400	-2.322243	-1.644967
H	0.389904	-2.995761	0.076596
C	3.124640	-0.595973	-1.224592
H	3.297358	0.048348	0.818829
C	2.467937	-1.450480	-2.108532
H	0.980547	-2.985210	-2.333272
H	3.880409	0.084381	-1.592214
H	2.719418	-1.432878	-3.160850

C-anti-TS0-H
Number of imaginary frequencies: 1
Electronic Energy (SCF) = -1171.73586507 a.u
ZPV corrected Energy = -1171.402935 a.u.
Enthalpy = -1171.384339 a.u.
Free Energy = -1171.449469 a.u.

S	0.083757	1.432365	-1.002332
C	-1.366208	2.571635	-0.852170
H	-2.186616	2.135469	-1.415048

H	-1.056012	3.501341	-1.327019
C	0.539724	1.594286	0.767460
H	1.616428	1.717206	0.816071
H	0.261811	0.667143	1.253774
C	-0.253829	2.780492	1.305750
H	0.254911	3.714526	1.061873
H	-0.322806	2.710024	2.390853
C	-1.626696	2.742370	0.637030
H	-2.205219	1.898310	1.011892
H	-2.195463	3.653583	0.820910
C	-0.499950	-0.231461	-1.263286
C	0.915147	-1.589691	-1.361858
O	0.401637	-2.712366	-1.071430
H	-0.782797	-0.205706	-2.314610
C	-1.638335	-0.661738	-0.415734
C	-2.944498	-0.610676	-0.917100
C	-1.449454	-1.161735	0.877426
C	-4.028778	-1.003885	-0.142561
H	-3.106609	-0.254012	-1.926462
C	-2.532408	-1.549974	1.656478
H	-0.449834	-1.270243	1.266309
C	-3.826725	-1.466273	1.153916
H	-5.029856	-0.949001	-0.550335
H	-2.363232	-1.932672	2.654685
H	-4.668548	-1.771586	1.761332
H	1.123045	-1.361047	-2.426264
C	2.046711	-1.052725	-0.509089
C	2.940252	-0.112108	-1.021584
C	2.233335	-1.511635	0.793622
C	3.972049	0.392940	-0.237925
H	2.826435	0.224447	-2.045436
C	3.257979	-1.006141	1.584068
H	1.564927	-2.273568	1.170635
C	4.127501	-0.044766	1.074051
H	4.657283	1.122480	-0.650546
H	3.385595	-1.365490	2.597479
H	4.928452	0.346387	1.687696

C-anti-H
Number of imaginary frequencies: 0
Electronic Energy (SCF) = -1171.74117093 a.u
ZPV corrected Energy = -1171.406662 a.u.
Enthalpy = -1171.387839 a.u.
Free Energy = -1171.453852 a.u.

S	0.583362	1.773162	0.839935
C	2.398710	2.028277	0.713002
H	2.885976	1.318068	1.374975
H	2.576590	3.037974	1.078676
C	0.295904	1.956149	-0.962257
H	-0.558240	2.613617	-1.086494
H	0.055468	0.975861	-1.357424
C	1.597380	2.510421	-1.541042
H	1.619964	3.595143	-1.428878
H	1.647032	2.280471	-2.604735
C	2.748001	1.879287	-0.762103
H	2.845326	0.824820	-1.016415
H	3.697949	2.370549	-0.970388
C	0.385988	-0.038613	1.329773
C	-1.097249	-0.389930	1.803669
O	-1.036076	-1.595148	2.354066
H	0.935320	0.005502	2.270340
C	1.097255	-0.974080	0.403545
C	2.348455	-1.468438	0.782753
C	0.575828	-1.370195	-0.831189
C	3.068112	-2.316300	-0.049118
H	2.756834	-1.188028	1.744844
C	1.293770	-2.217710	-1.665386
H	-0.401987	-1.036164	-1.137488
C	2.544527	-2.689030	-1.282019
H	4.033939	-2.687725	0.267051
H	0.870047	-2.514657	-2.615701
H	3.101477	-3.348938	-1.933887
H	-1.317174	0.448272	2.514242
C	-2.141058	-0.223557	0.686825
C	-2.648350	1.026459	0.333538
C	-2.641590	-1.348984	0.036109
C	-3.593165	1.158479	-0.679469
H	-2.319920	1.909519	0.870420
C	-3.585690	-1.227055	-0.977141
H	-2.271663	-2.315071	0.351180
C	-4.058630	0.029669	-1.346811
H	-3.975237	2.137956	-0.938583
H	-3.958735	-2.112182	-1.477363
H	-4.797345	0.126508	-2.131921

D-gauche-0-H
Number of imaginary frequencies: 0
Electronic Energy (SCF) = -1171.75263771 a.u
ZPV corrected Energy = -1171.421332 a.u.
Enthalpy = -1171.400840 a.u.
Free Energy = -1171.472649 a.u.

S	0.081232	1.291455	-0.839921
C	-0.998369	2.744672	-1.250293
H	-2.030345	2.402350	-1.235829
H	-0.732443	3.054342	-2.257780
C	0.179182	1.778386	0.964055
H	1.235025	1.962517	1.144186
H	-0.136337	0.920717	1.549616
C	-0.662408	3.032270	1.160693
H	-0.240885	3.656822	1.948023
H	-1.674424	2.763059	1.463283
C	-0.708203	3.779591	-0.178887
H	-1.470291	4.558912	-0.177254
H	0.254298	4.258514	-0.371893
C	-0.669025	-0.177315	-1.105791
C	0.929092	-1.832090	0.968676
H	0.413879	-2.538983	0.297884
O	0.456166	-1.553210	2.053528
C	2.215645	-1.320723	0.460163
C	2.659288	-1.726998	-0.799144
C	2.987993	-0.434719	1.215301
C	3.857038	-1.244758	-1.307334
H	2.054676	-2.413344	-1.378535
C	4.183285	0.048190	0.707052
H	2.636512	-0.131550	2.191625
C	4.617173	-0.353741	-0.555384
H	4.197267	-1.557282	-2.285362
H	4.780714	0.737226	1.289018
H	5.549580	0.026887	-0.950908
H	-0.078944	-0.805104	-1.754166
C	-1.920446	-0.659379	-0.605593
C	-2.360890	-1.948203	-0.992540
C	-2.756843	0.048392	0.283813
C	-3.547254	-2.483881	-0.522902
H	-1.747081	-2.524781	-1.673980
C	-3.946029	-0.495694	0.749420
H	-2.476248	1.034783	0.618371
C	-4.358801	-1.764899	0.355810
H	-3.844958	-3.474599	-0.844321
H	-4.556398	0.082319	1.432728
H	-5.285214	-2.185007	0.723107

D-gauche-TS0-H

Number of imaginary frequencies: 1
Electronic Energy (SCF) = -1171.74221192 a.u
ZPV corrected Energy = -1171.409488 a.u.
Enthalpy = -1171.390816 a.u.
Free Energy = -1171.456496 a.u.

S	0.202286	1.107043	-0.792322
C	-1.165251	2.107627	-1.511539
H	-2.082435	1.537791	-1.380201
H	-0.956624	2.234795	-2.570297
C	0.190949	2.019876	0.813073
H	1.151080	2.529662	0.838700
H	0.157547	1.273944	1.604526
C	-0.972752	3.004200	0.757263
H	-0.755176	3.869271	1.382781
H	-1.881153	2.538067	1.135848
C	-1.170753	3.400555	-0.707418
H	-2.110228	3.930438	-0.862408
H	-0.359732	4.052427	-1.036905
C	-0.319521	-0.556983	-0.540902
C	0.757946	-1.293814	0.944840
H	0.447525	-2.319719	0.668167
O	0.389031	-0.831942	2.067068
C	2.160864	-1.015636	0.444583
C	2.682945	-1.706531	-0.650593
C	2.959289	-0.068415	1.082266
C	3.962781	-1.437200	-1.116361
H	2.077771	-2.460796	-1.139936
C	4.240877	0.207697	0.616922
H	2.561969	0.440188	1.949470
C	4.745655	-0.471225	-0.487071
H	4.354052	-1.980744	-1.966904
H	4.849364	0.948455	1.120286
H	5.743513	-0.259741	-0.848460
H	0.062631	-1.086540	-1.408111
C	-1.731308	-0.859446	-0.265422
C	-2.398137	-1.795301	-1.069550
C	-2.424858	-0.317453	0.824554
C	-3.709447	-2.166274	-0.804209
H	-1.876761	-2.230112	-1.913260
C	-3.740070	-0.682253	1.083274
H	-1.926630	0.369353	1.488059
C	-4.391459	-1.605672	0.271787
H	-4.201396	-2.889093	-1.442323
H	-4.255077	-0.249597	1.931433
H	-5.415557	-1.887717	0.477154

D-gauche-H
Number of imaginary frequencies: 0
Electronic Energy (SCF) = -1171.75478228 a.u
ZPV corrected Energy = -1171.419788 a.u.
Enthalpy = -1171.401101 a.u.
Free Energy = -1171.467042 a.u.

S	-0.123416	1.489097	0.000331
C	-1.039494	1.985024	-1.526078
H	-1.435521	1.095420	-2.007955
H	-0.329901	2.469939	-2.192034
C	-1.531630	1.888283	1.099938
H	-1.176658	2.736264	1.683454
H	-1.674526	1.047786	1.765795
C	-2.722368	2.237956	0.217830
H	-3.405657	2.895769	0.754537
H	-3.264026	1.328892	-0.045207
C	-2.164126	2.894568	-1.044045
H	-2.920711	3.008816	-1.819921
H	-1.773759	3.886793	-0.809150
C	-0.000374	-0.356565	-0.227847
C	1.021627	-0.639494	0.922253
H	0.876618	-1.706361	1.186410
O	0.734473	0.220935	1.928851
C	2.447923	-0.541623	0.365553
C	2.924972	-1.469846	-0.562043
C	3.291598	0.483154	0.779693
C	4.214492	-1.371490	-1.070074
H	2.281563	-2.281144	-0.884390
C	4.585119	0.588184	0.273862
H	2.911000	1.185051	1.508819
C	5.050531	-0.337042	-0.653921
H	4.571591	-2.101304	-1.785806
H	5.230887	1.391824	0.605525
H	6.056326	-0.259169	-1.046033
H	0.397760	-0.459119	-1.237870
C	-1.315539	-1.061228	-0.133067
C	-2.000329	-1.414039	-1.298207
C	-1.875298	-1.396293	1.104008
C	-3.227169	-2.064715	-1.233629
H	-1.562203	-1.187171	-2.261525
C	-3.099902	-2.046924	1.168277
H	-1.336453	-1.140114	2.005755
C	-3.783974	-2.376399	0.001240
H	-3.743264	-2.331966	-2.146328
H	-3.521697	-2.299981	2.132142
H	-4.738842	-2.882311	0.055222

D-gauche'-0-H
Number of imaginary frequencies: 0
Electronic Energy (SCF) = -1171.75282708 a.u
ZPV corrected Energy = -1171.421298 a.u.
Enthalpy = -1171.400843 a.u.
Free Energy = -1171.472787 a.u.

S	-1.828034	-1.142860	0.810239
C	-3.464335	-0.455290	1.346113
H	-3.404361	0.629136	1.289717
H	-3.604512	-0.759085	2.380411
C	-2.419267	-1.375160	-0.951509
H	-2.307083	-2.438651	-1.145232
H	-1.742740	-0.824606	-1.597034
C	-3.869960	-0.914394	-1.025573
H	-4.425092	-1.508361	-1.751113
H	-3.916001	0.125681	-1.349230
C	-4.477459	-1.037926	0.378436
H	-5.429445	-0.511998	0.450263
H	-4.661032	-2.087976	0.615973
C	-0.574161	-0.050081	0.970114
C	1.113351	-1.723288	-1.159412
H	0.574276	-1.002065	-1.797213
O	0.702949	-2.860266	-1.025536
C	2.350936	-1.196380	-0.561884
C	2.771393	0.088694	-0.905902
C	3.105949	-1.959671	0.334442
C	3.939899	0.609086	-0.364948
H	2.176367	0.676755	-1.591951
C	4.266256	-1.436558	0.879254
H	2.767637	-2.953131	0.595350
C	4.684887	-0.151951	0.528457
H	4.262445	1.606566	-0.630820
H	4.849667	-2.022009	1.577272
H	5.592430	0.253264	0.956226
H	0.228568	-0.457682	1.564128
C	-0.476266	1.289150	0.475625
C	0.655384	2.065749	0.823823
C	-1.423399	1.904936	-0.372060
C	0.825453	3.356286	0.354624
H	1.404840	1.628547	1.470718

C	-1.245578	3.200886	-0.836998
H	-2.307961	1.366111	-0.674884
C	-0.122489	3.943911	-0.484288
H	1.708711	3.912366	0.644663
H	-1.996581	3.634178	-1.486515
H	0.011248	4.952863	-0.850152

D-gauche'-TS0-H
Number of imaginary frequencies: 1
Electronic Energy (SCF) = -1171.74115755 a.u
ZPV corrected Energy = -1171.408469 a.u.
Enthalpy = -1171.389708 a.u.
Free Energy = -1171.455695 a.u.

S	-1.818771	-1.268776	0.671643
C	-3.137122	-0.187438	1.346937
H	-2.809358	0.844604	1.243840
H	-3.257428	-0.432622	2.398844
C	-2.604534	-1.397048	-0.993195
H	-2.831752	-2.454161	-1.100896
H	-1.876868	-1.120967	-1.746936
C	-3.862308	-0.531839	-0.960928
H	-4.613150	-0.943718	-1.634102
H	-3.631261	0.478840	-1.295449
C	-4.357228	-0.489247	0.485865
H	-5.118364	0.276194	0.634112
H	-4.788924	-1.451057	0.768770
C	-0.301142	-0.369967	0.581208
C	0.657925	-1.460678	-0.794039
H	0.281809	-0.840285	-1.631873
O	0.292508	-2.672620	-0.711736
C	2.069897	-1.068901	-0.442969
C	2.633155	0.106074	-0.940209
C	2.834551	-1.881690	0.394156
C	3.931615	0.469417	-0.599789
H	2.049307	0.738941	-1.596388
C	4.131125	-1.521324	0.737832
H	2.395706	-2.799002	0.763626
C	4.684141	-0.341813	0.243550
H	4.356180	1.384301	-0.992894
H	4.714997	-2.159886	1.388773
H	5.695290	-0.061703	0.508891
H	0.228615	-0.657530	1.484369
C	-0.269772	1.070049	0.323884
C	0.553361	1.882228	1.120703
C	-0.923476	1.673898	-0.761121
C	0.714446	3.232847	0.848363
H	1.076710	1.436073	1.956443
C	-0.771431	3.028806	-1.024986
H	-1.543727	1.082508	-1.416924
C	0.048861	3.818172	-0.225026
H	1.359568	3.831589	1.478433
H	-1.289165	3.467796	-1.868223
H	0.169687	4.872179	-0.436244

D-gauche'-H
Number of imaginary frequencies: 0
Electronic Energy (SCF) = -1171.75328199 a.u
ZPV corrected Energy = -1171.417980 a.u.
Enthalpy = -1171.399321 a.u.
Free Energy = -1171.465210 a.u.

S	-1.489342	-1.596975	0.580534
C	-2.876295	-0.605468	1.291078
H	-2.644799	0.454374	1.221304
H	-2.976273	-0.886592	2.336840
C	-2.287558	-1.725044	-1.067081
H	-2.446374	-2.792583	-1.200580
H	-1.581237	-1.389389	-1.816917
C	-3.592752	-0.936121	-1.013718
H	-4.318493	-1.368119	-1.702263
H	-3.413727	0.095698	-1.315231
C	-4.084192	-0.967465	0.433119
H	-4.899574	-0.265462	0.605862
H	-4.445168	-1.966099	0.687706
C	-0.094554	-0.366298	0.457059
C	0.798284	-1.119852	-0.589501
H	0.530227	-0.691134	-1.578416
O	0.531526	-2.438754	-0.473540
C	2.251153	-0.710262	-0.342479
C	2.765982	0.486546	-0.840367
C	3.081438	-1.534350	0.413203
C	4.081529	0.857327	-0.581518
H	2.133790	1.130870	-1.438788
C	4.397794	-1.168860	0.674588
H	2.674614	-2.468359	0.776501
C	4.902500	0.030427	0.179899
H	4.467776	1.788031	-0.977636
H	5.033131	-1.821178	1.260759
H	5.927863	0.314357	0.378544
H	0.353121	-0.482952	1.444481
C	-0.449494	1.064452	0.234941
C	-0.285592	1.978866	1.277936
C	-0.908757	1.535175	-0.998892
C	-0.569868	3.327248	1.098675
H	0.074236	1.625742	2.235710
C	-1.200639	2.880641	-1.178204
H	-1.033344	0.853101	-1.826444
C	-1.031753	3.781780	-0.131158
H	-0.430859	4.020511	1.917614
H	-1.555421	3.227954	-2.139588
H	-1.255592	4.830421	-0.275171

D-anti-0-H
Number of imaginary frequencies: 0
Electronic Energy (SCF) = -1171.75350531 a.u
ZPV corrected Energy = -1171.421948 a.u.
Enthalpy = -1171.401552 a.u.
Free Energy = -1171.472677 a.u.

S	1.925088	-0.120866	-1.262676
C	3.425603	0.705203	-0.544539
H	3.088128	1.576539	0.011914
H	4.036270	1.024009	-1.385386
C	1.799455	-1.305647	0.181230
H	1.877999	-2.295925	-0.260044
H	0.811114	-1.185234	0.610082
C	2.945104	-1.007132	1.140948
H	3.288393	-1.921029	1.624879
H	2.608756	-0.328426	1.925091
C	4.073526	-0.342908	0.339813
H	4.817520	0.108534	0.996155
H	4.583834	-1.087357	-0.275187
C	0.613724	0.903325	-1.405319
C	-2.083433	-0.815647	-1.915349
O	-2.786705	0.158141	-2.094029
H	0.238614	0.930877	-2.416184
C	-0.005986	1.706334	-0.395224
C	-1.091272	2.541279	-0.751448
C	0.363586	1.703931	0.967052
C	-1.761515	3.303077	0.189358
H	-1.410586	2.564885	-1.784541
C	-0.311635	2.472902	1.904392
H	1.181721	1.086981	1.302676
C	-1.383378	3.278987	1.532607

H	-2.592559	3.922033	-0.126258
H	0.003173	2.436310	2.940304
H	-1.909125	3.874790	2.266449
H	-1.577708	-1.307318	-2.766417
C	-1.860270	-1.466865	-0.613567
C	-1.316306	-2.752204	-0.586448
C	-2.188644	-0.823449	0.582785
C	-1.115549	-3.399982	0.623979
H	-1.052952	-3.237007	-1.518521
C	-1.962388	-1.461674	1.791958
H	-2.595896	0.176788	0.549948
C	-1.431520	-2.750762	1.813749
H	-0.702760	-4.399598	0.643950
H	-2.195030	-0.957000	2.719976
H	-1.261295	-3.246734	2.760283

D-anti-TS0-H
Number of imaginary frequencies: 1
Electronic Energy (SCF) = -1171.73658186 a.u
ZPV corrected Energy = -1171.403600 a.u.
Enthalpy = -1171.384945 a.u.
Free Energy = -1171.450632 a.u.

S	-0.005295	1.666555	-1.060680
C	-1.438072	2.772607	-0.687207
H	-2.276952	2.121266	-0.452178
H	-1.652967	3.342650	-1.586898
C	0.761018	1.913029	0.598998
H	1.647643	2.509693	0.397687
H	1.074317	0.954302	0.992191
C	-0.251863	2.671853	1.446951
H	0.263846	3.214238	2.238715
H	-0.948606	1.975187	1.911277
C	-1.006629	3.611619	0.507779
H	-1.880334	4.052499	0.987214
H	-0.355767	4.424366	0.181366
C	-0.590505	0.002753	-1.277739
C	0.793245	-1.240243	-1.744531
O	0.241783	-2.375174	-1.907123
H	-1.018581	0.055929	-2.277046
C	-1.556267	-0.571558	-0.321087
C	-2.721588	-1.159408	-0.830971
C	-1.331097	-0.663778	1.058103
C	-3.631750	-1.799292	0.000232
H	-2.909682	-1.111978	-1.895528
C	-2.246018	-1.292769	1.892136
H	-0.429047	-0.264602	1.489745
C	-3.402029	-1.864048	1.370201
H	-4.521942	-2.245890	-0.423526
H	-2.046545	-1.347252	2.954591
H	-4.110493	-2.358005	2.021789
H	1.116547	-0.675885	-2.644935
C	1.830274	-1.071655	-0.658837
C	2.881685	-0.169530	-0.819032
C	1.758807	-1.819549	0.516090
C	3.826382	0.010393	0.185372
H	2.957879	0.395792	-1.740936
C	2.691871	-1.632888	1.528750
H	0.963427	-2.543986	0.619841
C	3.726327	-0.712640	1.369900
H	4.639314	0.711467	0.044922
H	2.619045	-2.209136	2.442540
H	4.456610	-0.573512	2.156374

D-anti-H
Number of imaginary frequencies: 0
Electronic Energy (SCF) = -1171.74163625 a.u
ZPV corrected Energy = -1171.407152 a.u.
Enthalpy = -1171.388323 a.u.
Free Energy = -1171.454141 a.u.

S	0.672990	1.767269	0.713328
C	2.498833	1.972896	0.730519
H	2.926572	1.001966	0.967476
H	2.740398	2.681846	1.517501
C	0.551052	1.801611	-1.115579
H	0.104806	2.766659	-1.347446
H	-0.126066	1.017689	-1.432877
C	1.974643	1.678161	-1.645384
H	2.029140	2.085263	-2.654454
H	2.264550	0.629028	-1.684797
C	2.874092	2.438234	-0.673165
H	3.930559	2.242629	-0.853649
H	2.708287	3.512305	-0.766871
C	0.379675	0.005787	1.315814
C	-1.117512	-0.217077	1.820147
O	-1.133482	-1.385844	2.446603
H	0.946920	0.061036	2.245159
C	1.007567	-1.021639	0.427308
C	2.223634	-1.591834	0.813900
C	0.436779	-1.434121	-0.779623
C	2.861223	-2.534053	0.016156
H	2.666487	-1.300080	1.757252
C	1.071600	-2.375793	-1.578451
H	-0.512615	-1.033864	-1.094372
C	2.288156	-2.925756	-1.187968
H	3.800577	-2.963976	0.337883
H	0.610588	-2.683871	-2.507632
H	2.779722	-3.659667	-1.812743
H	-1.276050	0.678080	2.475874
C	-2.155683	-0.053752	0.696297
C	-2.557956	1.200415	0.236147
C	-2.751999	-1.183984	0.142098
C	-3.494821	1.324584	-0.785244
H	-2.153842	2.095766	0.694660
C	-3.689990	-1.069628	-0.877803
H	-2.458178	-2.147513	0.535126
C	-4.058738	0.186250	-1.353369
H	-3.794242	2.307229	-1.127664
H	-4.138803	-1.959653	-1.301209
H	-4.792135	0.277775	-2.144082

E-gauche-0-H
Number of imaginary frequencies: 0
Electronic Energy (SCF) = -1171.75337168 a.u
ZPV corrected Energy = -1171.422278 a.u.
Enthalpy = -1171.401842 a.u.
Free Energy = -1171.473314 a.u.

S	-1.551596	-0.523410	-0.662404
C	-2.921342	-1.662897	-1.235536
H	-2.509598	-2.668710	-1.221332
H	-3.148438	-1.374162	-2.262276
C	-2.728829	0.536901	0.283138
H	-2.508204	1.571429	0.040327
H	-2.499531	0.361418	1.330649
C	-4.138700	0.096844	-0.087803
H	-4.458935	0.589746	-1.007556
H	-4.834691	0.378978	0.701975
C	-4.092235	-1.417666	-0.302355
H	-3.925290	-1.925440	0.649834
H	-5.022404	-1.797519	-0.725185
C	-0.572227	-1.240421	0.500905
H	-1.040031	-1.523832	1.433347
C	0.365882	1.235526	2.013235

H	0.979871	0.381941	2.342459
O	-0.581137	1.615134	2.679257
C	0.834390	1.884901	0.777065
C	2.015287	1.439599	0.181183
C	0.121249	2.941379	0.200717
C	2.478415	2.039451	-0.982780
H	2.560765	0.620874	0.629567
C	0.580924	3.534041	-0.963000
H	-0.788175	3.282009	0.675935
C	1.760937	3.082770	-1.556432
H	3.391054	1.688292	-1.444858
H	0.028004	4.348104	-1.412662
H	2.118105	3.547761	-2.465909
C	0.752775	-1.689616	0.199275
C	1.535811	-2.261125	1.231608
C	1.377347	-1.564874	-1.063478
C	2.849080	-2.647773	1.021683
H	1.093376	-2.384127	2.212762
C	2.694197	-1.948907	-1.262173
H	0.823385	-1.151247	-1.895745
C	3.452280	-2.491268	-0.226471
H	3.411520	-3.074683	1.843362
H	3.134603	-1.824722	-2.244165
H	4.477817	-2.793806	-0.388803

E-gauche-TS0-H
Number of imaginary frequencies: 1
Electronic Energy (SCF) = -1171.74393406 a.u
ZPV corrected Energy = -1171.411573 a.u.
Enthalpy = -1171.392775 a.u.
Free Energy = -1171.459542 a.u.

S	-1.412603	-0.081914	-0.777175
C	-2.493663	-1.472097	-1.301518
H	-1.889519	-2.375517	-1.303057
H	-2.807458	-1.243257	-2.319911
C	-2.787173	0.841195	0.020439
H	-2.838208	1.809606	-0.468318
H	-2.471650	0.980658	1.054093
C	-4.059740	0.005378	-0.161378
H	-4.591453	0.337487	-1.053650
H	-4.717883	0.151920	0.693986
C	-3.657690	-1.460391	-0.323700
H	-3.346894	-1.882830	0.632446
H	-4.479363	-2.066162	-0.705162
C	-0.482739	-0.693635	0.595584
H	-1.150900	-1.155057	1.315398
C	0.082015	0.800354	1.735573
H	0.799394	0.144170	2.263973
O	-0.915960	1.254558	2.384758
C	0.812788	1.700048	0.767561
C	2.136475	1.443078	0.408149
C	0.172477	2.811508	0.217967
C	2.797266	2.261356	-0.501454
H	2.651803	0.600089	0.848881
C	0.827717	3.628846	-0.694703
H	-0.839864	3.030811	0.528416
C	2.142405	3.353271	-1.062657
H	3.824352	2.049010	-0.769646
H	0.317789	4.486640	-1.114891
H	2.655543	3.991707	-1.769995
C	0.650489	-1.545160	0.208623
C	1.100287	-2.507587	1.127256
C	1.378477	-1.388958	-0.980876
C	2.229863	-3.271886	0.872137
H	0.553018	-2.648062	2.050824
C	2.504168	-2.160718	-1.236802
H	1.074982	-0.651336	-1.710756
C	2.940976	-3.104713	-0.313254
H	2.554312	-4.004137	1.600317
H	3.045808	-2.017662	-2.162946
H	3.819119	-3.703265	-0.514944

E-gauche-H
Number of imaginary frequencies: 0
Electronic Energy (SCF) = -1171.75423406 a.u
ZPV corrected Energy = -1171.419310 a.u.
Enthalpy = -1171.400625 a.u.
Free Energy = -1171.466567 a.u.

S	1.601432	-0.932791	-0.505661
C	2.551642	0.462067	-1.265982
H	1.920114	1.342200	-1.325589
H	2.780776	0.130261	-2.278553
C	3.097058	-1.578401	0.345561
H	3.134356	-2.641054	0.127270
H	2.897501	-1.455216	1.404954
C	4.302801	-0.782708	-0.141407
H	4.706379	-1.230998	-1.050775
H	5.086733	-0.800557	0.615789
C	3.816350	0.634486	-0.440377
H	3.588704	1.162843	0.487457
H	4.561359	1.217336	-0.982220
C	0.629111	-0.035514	0.800224
H	1.351191	0.147715	1.592648
C	-0.267739	-1.251043	1.271050
H	-0.471106	-1.038463	2.339143
O	0.495787	-2.349303	1.057675
C	-1.628676	-1.265789	0.579198
C	-2.673401	-0.456948	1.025396
C	-1.843618	-2.083617	-0.526586
C	-3.899001	-0.447189	0.370523
H	-2.522068	0.177333	1.890831
C	-3.066448	-2.075519	-1.190769
H	-1.038215	-2.730012	-0.846244
C	-4.097625	-1.253062	-0.747434
H	-4.698835	0.188085	0.729509
H	-3.217593	-2.714463	-2.051947
H	-5.050645	-1.247773	-1.260440
C	0.030910	1.259528	0.360164
C	0.179818	2.383352	1.173950
C	-0.717157	1.379117	-0.814952
C	-0.416678	3.593272	0.836008
H	0.763269	2.304626	2.082485
C	-1.298578	2.589449	-1.163247
H	-0.849476	0.521645	-1.459792
C	-1.156296	3.700026	-0.335813
H	-0.297279	4.451385	1.484227
H	-1.871914	2.664058	-2.077684
H	-1.617292	4.641086	-0.604932

E-gauche'-0-H
Number of imaginary frequencies: 0
Electronic Energy (SCF) = -1171.75276324 a.u
ZPV corrected Energy = -1171.421556 a.u.
Enthalpy = -1171.401296 a.u.
Free Energy = -1171.471228 a.u.

S	-2.275531	0.308711	0.425190
C	-3.766119	1.308169	-0.116103
H	-3.380066	2.177927	-0.641152
H	-4.263163	1.625645	0.800667
C	-3.087089	-1.283359	-0.025797
H	-2.881740	-1.991271	0.771446
H	-2.596096	-1.612058	-0.939260

C	-4.562330	-0.986814	-0.246768
H	-5.088345	-0.945284	0.708886
H	-5.016917	-1.777370	-0.843647
C	-4.634972	0.375658	-0.941064
H	-4.241423	0.296067	-1.956432
H	-5.658342	0.745066	-1.007726
C	-0.970263	0.468019	-0.623051
H	-1.138988	0.201928	-1.658346
C	0.174023	-2.137299	0.668987
H	-0.444784	-2.598509	-0.119337
O	-0.244051	-2.043356	1.809380
C	1.515897	-1.733765	0.227393
C	1.903483	-1.994511	-1.087392
C	2.398846	-1.089340	1.097364
C	3.167693	-1.629616	-1.528418
H	1.205895	-2.478548	-1.759756
C	3.658279	-0.719819	0.654603
H	2.082022	-0.880977	2.109082
C	4.044683	-0.992646	-0.656023
H	3.467496	-1.832254	-2.547809
H	4.339095	-0.210494	1.322851
H	5.027506	-0.697940	-0.999547
C	0.172598	1.264208	-0.278106
C	1.155349	1.517844	-1.263568
C	0.426148	1.793277	1.008579
C	2.302306	2.240128	-0.981472
H	1.005032	1.124243	-2.261220
C	1.576930	2.516441	1.281217
H	-0.288525	1.629716	1.804389
C	2.531629	2.752502	0.294516
H	3.031036	2.400735	-1.766735
H	1.730853	2.899382	2.282949
H	3.427830	3.317003	0.513247

E-gauche'-TS0-H
Number of imaginary frequencies: 1
Electronic Energy (SCF) = -1171.74345500 a.u
ZPV corrected Energy = -1171.411345 a.u.
Enthalpy = -1171.392439 a.u.
Free Energy = -1171.459256 a.u.

S	-2.236478	0.117399	0.638144
C	-3.315856	1.203214	-0.383927
H	-2.678687	1.931458	-0.878486
H	-3.972241	1.717537	0.317002
C	-3.172414	-1.407052	0.225971
H	-3.331884	-1.955283	1.148967
H	-2.534391	-1.988834	-0.433458
C	-4.451370	-0.948062	-0.469877
H	-5.205234	-0.678618	0.271307
H	-4.851283	-1.761580	-1.074150
C	-4.087891	0.274715	-1.310379
H	-3.460954	-0.018729	-2.153734
H	-4.969723	0.778409	-1.705064
C	-0.726553	-0.074622	-0.264141
H	-0.931766	-0.393363	-1.282087
C	0.042540	-1.632177	0.738725
H	-0.510083	-2.379660	0.137424
O	-0.233634	-1.517821	1.973320
C	1.464443	-1.539622	0.257880
C	1.815512	-1.999072	-1.011029
C	2.446968	-0.966160	1.062315
C	3.119718	-1.879496	-1.474829
H	1.056085	-2.449205	-1.640460
C	3.752173	-0.839864	0.601328
H	2.168665	-0.622327	2.048499
C	4.093039	-1.293354	-0.669460
H	3.379344	-2.241775	-2.461485
H	4.505277	-0.384842	1.232135
H	5.109225	-1.195323	-1.028651
C	0.162888	1.084524	-0.158690
C	0.953977	1.445502	-1.260793
C	0.357475	1.782660	1.043856
C	1.905186	2.449828	-1.162401
H	0.824995	0.916945	-2.196317
C	1.304055	2.794652	1.135493
H	-0.218625	1.515284	1.918629
C	2.086715	3.134180	0.036764
H	2.507338	2.700941	-2.026207
H	1.437129	3.314016	2.075928
H	2.825909	3.920372	0.112505

E-gauche'-H
Number of imaginary frequencies: 0
Electronic Energy (SCF) = -1171.75409530 a.u
ZPV corrected Energy = -1171.419325 a.u.
Enthalpy = -1171.400580 a.u.
Free Energy = -1171.466854 a.u.

S	2.126674	-0.126290	-0.775073
C	3.211605	0.987735	0.220747
H	2.597926	1.772317	0.655999
H	3.903480	1.438888	-0.489235
C	3.064623	-1.628186	-0.288294
H	3.259952	-2.179962	-1.202033
H	2.401686	-2.214351	0.340785
C	4.313818	-1.163772	0.456380
H	5.110225	-0.939147	-0.254810
H	4.667224	-1.957373	1.114428
C	3.936892	0.102077	1.223738
H	3.274110	-0.143866	2.055390
H	4.810730	0.610303	1.631219
C	0.629987	-0.129547	0.303787
H	0.966318	-0.183138	1.337766
C	-0.047692	-1.464726	-0.200404
H	0.341497	-2.242058	0.497822
O	0.323226	-1.656476	-1.482199
C	-1.549890	-1.424384	0.073732
C	-2.041267	-1.379216	1.379123
C	-2.454146	-1.431022	-0.981787
C	-3.407778	-1.327220	1.624162
H	-1.347207	-1.383560	2.211981
C	-3.825209	-1.384600	-0.743090
H	-2.058267	-1.476301	-1.986696
C	-4.307173	-1.328436	0.559971
H	-3.773658	-1.288186	2.642474
H	-4.518007	-1.391230	-1.575399
H	-5.372426	-1.290785	0.747673
C	-0.114316	1.148655	0.070135
C	-0.382969	1.997202	1.143888
C	-0.582675	1.496199	-1.200591
C	-1.112365	3.166635	0.959354
H	-0.025210	1.733043	2.130846
C	-1.309848	2.664129	-1.384994
H	-0.385935	0.833213	-2.030932
C	-1.577121	3.503356	-0.306787
H	-1.316141	3.812654	1.803150
H	-1.672311	2.919242	-2.372157
H	-2.143435	4.413481	-0.453931

E-anti-0-H
Number of imaginary frequencies: 0
Electronic Energy (SCF) = -1171.75256692 a.u
ZPV corrected Energy = -1171.421667 a.u.
Enthalpy = -1171.401016 a.u.

Free Energy = -1171.474552 a.u.

S	-0.327638	1.599841	0.519686
C	-0.566655	3.454784	0.486544
H	0.403770	3.884113	0.252265
H	-0.859573	3.740923	1.497171
C	-1.959032	1.304539	-0.297363
H	-2.459041	0.508525	0.245062
H	-1.718383	0.959977	-1.300313
C	-2.710223	2.627521	-0.305751
H	-3.217257	2.781323	0.648664
H	-3.468376	2.621692	-1.088826
C	-1.667568	3.727493	-0.521812
H	-1.269331	3.669226	-1.536867
H	-2.091515	4.722459	-0.385582
C	0.846113	1.093848	-0.564267
C	0.003037	-1.889156	-1.523014
O	-0.238643	-1.845095	-2.714285
H	1.039637	-1.820301	-1.158568
C	-1.013208	-2.055290	-0.469011
C	-0.612729	-1.991858	0.866083
C	-2.361783	-2.239306	-0.784399
C	-1.553654	-2.095705	1.881377
H	0.433557	-1.841724	1.098704
C	-3.299311	-2.350591	0.229490
H	-2.658961	-2.283985	-1.823175
C	-2.896473	-2.273747	1.562679
H	-1.244269	-2.035604	2.916045
H	-4.344398	-2.493050	-0.010966
H	-3.631798	-2.355342	2.352329
H	0.699967	1.334467	-1.608174
C	2.082207	0.513480	-0.131336
C	3.028611	0.110434	-1.104985
C	2.431132	0.264883	1.217584
C	4.219243	-0.505829	-0.755786
H	2.802293	0.280235	-2.150681
C	3.622232	-0.358673	1.555986
H	1.756197	0.565450	2.008227
C	4.534784	-0.754784	0.579450
H	4.909530	-0.799214	-1.537636
H	3.844252	-0.532490	2.602194
H	5.464709	-1.235632	0.850618

E-anti-TS0-H
Number of imaginary frequencies: 1
Electronic Energy (SCF) = -1171.73990705 a.u
ZPV corrected Energy = -1171.407777 a.u.
Enthalpy = -1171.388847 a.u.
Free Energy = -1171.455796 a.u.

S	-0.081828	1.181476	0.662331
C	0.836936	2.759275	0.373327
H	1.874341	2.501723	0.179174
H	0.771430	3.314285	1.308326
C	-1.568793	1.741448	-0.262168
H	-2.443779	1.493847	0.329162
H	-1.583666	1.168560	-1.183645
C	-1.378098	3.234781	-0.511437
H	-1.698264	3.805049	0.361972
H	-1.993270	3.541804	-1.356687
C	0.110781	3.460813	-0.763704
H	0.403526	3.025473	-1.720193
H	0.368120	4.519462	-0.782411
C	0.615215	-0.063796	-0.399459
C	-0.243554	-1.826315	-0.302179
O	0.075425	-2.430697	-1.369805
H	0.262410	-2.106601	0.643312
C	-1.696254	-1.509491	-0.031855
C	-2.145194	-1.290516	1.270868
C	-2.614465	-1.445054	-1.079047
C	-3.478261	-0.983737	1.521872
H	-1.443299	-1.366591	2.093111
C	-3.946211	-1.135572	-0.834014
H	-2.263603	-1.642676	-2.083103
C	-4.382775	-0.897941	0.467623
H	-3.812989	-0.818086	2.537980
H	-4.648849	-1.082941	-1.656152
H	-5.421181	-0.661171	0.659244
H	0.421614	0.202989	-1.433882
C	2.035235	-0.320449	-0.110005
C	2.926249	-0.529416	-1.170612
C	2.525231	-0.460935	1.197853
C	4.253527	-0.866187	-0.934817
H	2.567194	-0.429321	-2.186106
C	3.854655	-0.783901	1.431332
H	1.860929	-0.323306	2.041680
C	4.727981	-0.990543	0.366731
H	4.920016	-1.027590	-1.772343
H	4.208759	-0.882463	2.449418
H	5.763176	-1.245260	0.550430

E-anti-H
Number of imaginary frequencies: 0
Electronic Energy (SCF) = -1171.74581383 a.u
ZPV corrected Energy = -1171.411738 a.u.
Enthalpy = -1171.392732 a.u.
Free Energy = -1171.459203 a.u.

S	-0.023510	1.073371	0.830046
C	1.183935	2.415487	0.499461
H	2.158744	1.963282	0.345317
H	1.207193	3.013728	1.408987
C	-1.384199	1.908698	-0.088857
H	-2.262381	1.902824	0.548390
H	-1.577559	1.297959	-0.964628
C	-0.874267	3.304275	-0.444037
H	-1.067838	3.992022	0.380012
H	-1.406568	3.671330	-1.320908
C	0.628941	3.193440	-0.684015
H	0.830236	2.657174	-1.612312
H	1.107368	4.170234	-0.748048
C	0.476153	-0.336947	-0.312598
C	-0.380653	-1.660403	-0.088772
O	-0.056610	-2.484344	-1.079340
H	-0.095653	-2.003599	0.934594
C	-1.870375	-1.303025	0.006411
C	-2.487054	-1.045371	1.229761
C	-2.637500	-1.227490	-1.155300
C	-3.832508	-0.690562	1.292722
H	-1.911253	-1.131061	2.144141
C	-3.978432	-0.869145	-1.101324
H	-2.159417	-1.467747	-2.095581
C	-4.580929	-0.593080	0.125022
H	-4.296982	-0.498839	2.251833
H	-4.560170	-0.810196	-2.012824
H	-5.627489	-0.320969	0.169370
H	0.260337	0.028698	-1.314037
C	1.944488	-0.568913	-0.127918
C	2.822446	-0.353737	-1.189496
C	2.461233	-0.993675	1.098301
C	4.187423	-0.569102	-1.036465
H	2.429864	-0.024780	-2.142624
C	3.825397	-1.200156	1.255730
H	1.794787	-1.170128	1.932148
C	4.693363	-0.989640	0.188258
H	4.854277	-0.407851	-1.873163

H	4.211222	-1.530567	2.211080
H	5.755547	-1.154611	0.310945

F-gauche-0-H
Number of imaginary frequencies: 0
Electronic Energy (SCF) = -1171.75271211 a.u
ZPV corrected Energy = -1171.421702 a.u.
Enthalpy = -1171.401223 a.u.
Free Energy = -1171.473012 a.u.

S	-1.552435	-0.597206	-0.661197
C	-2.940907	-1.776618	-1.104095
H	-2.781887	-2.644057	-0.466341
H	-2.812166	-2.061746	-2.145447
C	-2.711479	0.552528	0.204447
H	-2.866542	1.386723	-0.477803
H	-2.206984	0.904959	1.097853
C	-3.999842	-0.215430	0.450703
H	-4.822478	0.472259	0.646858
H	-3.887813	-0.862706	1.322583
C	-4.246618	-1.059004	-0.801603
H	-5.053884	-1.777342	-0.656621
H	-4.523558	-0.410883	-1.635284
C	-0.538100	-1.273090	0.494957
H	-0.985949	-1.582211	1.429344
C	0.387718	1.189873	2.022410
H	1.052860	0.375224	2.350275
O	-0.578425	1.512032	2.692844
C	0.804118	-1.665022	0.185595
C	1.606314	-2.229044	1.207219
C	1.424875	-1.495866	-1.073887
C	2.930899	-2.571378	0.989949
H	1.169091	-2.383269	2.186328
C	2.751521	-1.838442	-1.280696
H	0.859051	-1.080101	-1.896895
C	3.527622	-2.376216	-0.255580
H	3.506848	-2.994385	1.804319
H	3.186783	-1.682509	-2.260493
H	4.561789	-2.644117	-0.423895
C	0.808695	1.862691	0.783259
C	2.008502	1.483000	0.179476
C	0.030978	2.873987	0.208621
C	2.427752	2.104379	-0.989742
H	2.603068	0.698078	0.626059
C	0.446732	3.486649	-0.960974
H	-0.893345	3.163933	0.688376
C	1.646628	3.101770	-1.561650
H	3.355456	1.804579	-1.457924
H	-0.155821	4.265181	-1.409740
H	1.969401	3.582834	-2.475619

F-gauche-TS0-H
Number of imaginary frequencies: 1
Electronic Energy (SCF) = -1171.74463680 a.u
ZPV corrected Energy = -1171.412234 a.u.
Enthalpy = -1171.393503 a.u.
Free Energy = -1171.459867 a.u.

S	1.445717	0.057409	-0.677414
C	2.514833	1.453210	-1.267969
H	2.218372	2.322206	-0.685329
H	2.292692	1.625298	-2.317499
C	2.816352	-0.808391	0.172936
H	3.089702	-1.633109	-0.482305
H	2.399976	-1.187359	1.108092
C	3.936921	0.213876	0.305166
H	4.884708	-0.294581	0.480204
H	3.745343	0.869053	1.156007
C	3.952024	1.020670	-0.992453
H	4.594996	1.897828	-0.925054
H	4.317315	0.399519	-1.811738
C	0.479957	0.679493	0.662859
H	1.131414	1.127772	1.406483
C	-0.137595	-0.839197	1.750785
H	-0.844993	-0.181048	2.289378
O	0.839194	-1.339171	2.398958
C	-0.631801	1.546100	0.250900
C	-1.122722	2.480877	1.177060
C	-1.303715	1.428121	-0.975171
C	-2.239334	3.254114	0.893879
H	-0.618234	2.592338	2.128508
C	-2.416749	2.208461	-1.258695
H	-0.966533	0.713103	-1.713091
C	-2.895680	3.123832	-0.327281
H	-2.596705	3.964459	1.628334
H	-2.915202	2.094958	-2.212727
H	-3.763748	3.729431	-0.550396
C	-0.873000	-1.688666	0.742755
C	-2.183842	-1.388225	0.370432
C	-0.248029	-2.794161	0.163658
C	-2.847635	-2.159190	-0.577517
H	-2.686741	-0.547846	0.830231
C	-0.906075	-3.563775	-0.787585
H	0.755034	-3.046905	0.479655
C	-2.208270	-3.245930	-1.165832
H	-3.864684	-1.913384	-0.855197
H	-0.407966	-4.416931	-1.230732
H	-2.723625	-3.847277	-1.903425

F-gauche-H
Number of imaginary frequencies: 0
Electronic Energy (SCF) = -1171.75382404 a.u
ZPV corrected Energy = -1171.419018 a.u.
Enthalpy = -1171.400299 a.u.
Free Energy = -1171.466065 a.u.

S	-1.631194	-0.837759	0.393924
C	-2.522204	0.591899	1.168085
H	-2.374274	1.479392	0.557617
H	-2.092238	0.760455	2.151530
C	-3.146906	-1.431661	-0.457341
H	-3.338116	-2.409202	-0.018714
H	-2.880047	-1.569063	-1.498358
C	-4.262670	-0.432638	-0.182174
H	-5.231786	-0.928888	-0.228617
H	-4.251898	0.353827	-0.938717
C	-3.986097	0.173620	1.192158
H	-4.625135	1.030453	1.404549
H	-4.156529	-0.571077	1.972727
C	-0.593850	0.014591	-0.880365
H	-1.288040	0.227095	-1.691216
C	0.275044	-1.228182	-1.330982
H	0.537347	-1.006536	-2.384567
O	-0.536428	-2.299919	-1.171982
C	0.035962	1.285463	-0.410872
C	-0.003750	2.406134	-1.241522
C	0.710931	1.379895	0.809111
C	0.629729	3.588355	-0.874378
H	-0.530841	2.346441	-2.185392
C	1.328485	2.563723	1.185832
H	0.761438	0.522869	1.466327
C	1.296042	3.670978	0.342642
H	0.595856	4.444522	-1.535211
H	1.844545	2.619678	2.135069
H	1.785225	4.590850	0.634290

C	1.599332	-1.301456	-0.573527
C	2.693618	-0.525722	-0.955314
C	1.729140	-2.140326	0.529935
C	3.884472	-0.569345	-0.240379
H	2.609202	0.124623	-1.817888
C	2.916708	-2.185540	1.253930
H	0.886103	-2.760653	0.800242
C	3.997886	-1.396289	0.874072
H	4.723643	0.040658	-0.550000
H	3.001175	-2.840388	2.112302
H	4.923656	-1.432558	1.433641

F-gauche'-0-H
Number of imaginary frequencies: 0
Electronic Energy (SCF) = -1171.75101736 a.u
ZPV corrected Energy = -1171.420158 a.u.
Enthalpy = -1171.399649 a.u.
Free Energy = -1171.471092 a.u.

S	-2.335541	0.336229	0.365562
C	-3.827270	1.243185	-0.299452
H	-3.555327	1.549111	-1.308365
H	-3.976831	2.123801	0.320480
C	-3.131218	-1.315728	0.094989
H	-3.357515	-1.703590	1.086188
H	-2.388817	-1.949009	-0.377311
C	-4.387640	-1.088443	-0.732139
H	-5.090905	-1.909372	-0.593477
H	-4.130979	-1.040972	-1.792099
C	-4.976584	0.250977	-0.283495
H	-5.781717	0.581994	-0.939952
H	-5.385225	0.160232	0.724990
C	-1.022749	0.482359	-0.671032
H	-1.178832	0.211334	-1.707150
C	0.275123	-2.112445	0.702882
H	-0.324280	-2.613636	-0.076888
O	-0.163386	-1.977350	1.829983
C	1.614134	-1.701606	0.260403
C	2.011980	-1.989322	-1.045700
C	2.483916	-1.025586	1.119648
C	3.272998	-1.616735	-1.489548
H	1.324896	-2.499914	-1.709096
C	3.740271	-0.649936	0.674577
H	2.158808	-0.797135	2.124419
C	4.136520	-0.947790	-0.628015
H	3.580912	-1.839393	-2.502256
H	4.411150	-0.116503	1.333971
H	5.117215	-0.648242	-0.973428
C	0.128748	1.261719	-0.313506
C	1.121465	1.505890	-1.291606
C	0.376036	1.796182	0.972558
C	2.266881	2.229303	-1.006266
H	0.979119	1.105264	-2.287812
C	1.524514	2.521824	1.247881
H	-0.343211	1.636426	1.764909
C	2.486613	2.752533	0.266868
H	3.001844	2.382200	-1.787326
H	1.671358	2.910560	2.248524
H	3.381659	3.317677	0.488540

F-gauche'-TS0-H
Number of imaginary frequencies: 1
Electronic Energy (SCF) = -1171.74248271 a.u
ZPV corrected Energy = -1171.410450 a.u.
Enthalpy = -1171.391501 a.u.
Free Energy = -1171.458962 a.u.

S	2.221990	0.058750	-0.649025
C	3.305698	1.217737	0.303057
H	2.723038	1.552424	1.157400
H	3.528313	2.066803	-0.337437
C	3.196414	-1.420451	-0.193256
H	3.792673	-1.658177	-1.071945
H	2.503985	-2.233232	-0.003544
C	4.059457	-1.014213	0.994020
H	4.891366	-1.708946	1.106325
H	3.470567	-1.042906	1.911894
C	4.535038	0.409423	0.712581
H	5.007902	0.866199	1.581364
H	5.262787	0.402192	-0.100344
C	0.734164	-0.100381	0.297116
H	0.963802	-0.385085	1.319792
C	-0.054543	-1.703127	-0.639106
H	0.454334	-2.419495	0.036084
O	0.277595	-1.675900	-1.864279
C	-1.489594	-1.542356	-0.225819
C	-1.906669	-1.920508	1.049914
C	-2.419063	-0.981920	-1.099816
C	-3.223821	-1.735369	1.451381
H	-1.188334	-2.358174	1.733954
C	-3.736320	-0.789025	-0.701026
H	-2.090297	-0.700513	-2.090305
C	-4.143417	-1.162999	0.576201
H	-3.534796	-2.035325	2.444068
H	-4.447459	-0.344431	-1.385654
H	-5.169350	-1.013470	0.886594
C	-0.147476	1.063299	0.180641
C	-0.926929	1.446004	1.284103
C	-0.347848	1.747603	-1.029372
C	-1.871589	2.455787	1.179913
H	-0.793844	0.929767	2.225890
C	-1.286558	2.766109	-1.126609
H	0.218848	1.466508	-1.906290
C	-2.058041	3.126006	-0.026449
H	-2.464749	2.722572	2.045264
H	-1.422746	3.274211	-2.072726
H	-2.792054	3.916563	-0.106592

F-gauche'-H
Number of imaginary frequencies: 0
Electronic Energy (SCF) = -1171.75282118 a.u
ZPV corrected Energy = -1171.418129 a.u.
Enthalpy = -1171.399301 a.u.
Free Energy = -1171.465988 a.u.

S	2.127722	-0.154072	-0.751493
C	3.206454	0.976734	0.249409
H	2.638990	1.303504	1.117045
H	3.436195	1.840768	-0.368241
C	3.058977	-1.651771	-0.254484
H	3.641316	-1.920337	-1.134364
H	2.341030	-2.438858	-0.057441
C	3.939617	-1.259671	0.923346
H	4.760529	-1.968711	1.029522
H	3.358058	-1.279866	1.846437
C	4.433892	0.156808	0.641224
H	4.928630	0.603437	1.503260
H	5.149833	0.140458	-0.182575
C	0.613916	-0.177851	0.296184
H	0.930775	-0.280326	1.332536
C	-0.086439	-1.481465	-0.268143
H	0.292025	-2.289689	0.401750
O	0.273599	-1.631373	-1.555915
C	-1.586189	-1.416552	0.015990
C	-2.074178	-1.472568	1.321836

C	-2.490717	-1.289098	-1.031855
C	-3.437818	-1.390958	1.576627
H	-1.379548	-1.582063	2.147115
C	-3.858387	-1.210851	-0.784142
H	-2.097366	-1.257532	-2.038322
C	-4.336970	-1.257733	0.520819
H	-3.801584	-1.434269	2.595510
H	-4.551574	-1.112368	-1.610320
H	-5.399903	-1.196903	0.715470
C	-0.102394	1.123560	0.105823
C	-0.409382	1.910135	1.215788
C	-0.514708	1.549344	-1.160529
C	-1.121025	3.095749	1.070667
H	-0.097175	1.583960	2.199654
C	-1.223753	2.734034	-1.305462
H	-0.292279	0.932786	-2.019553
C	-1.529250	3.511310	-0.191578
H	-1.355456	3.692609	1.942260
H	-1.542958	3.050312	-2.289849
H	-2.081768	4.434274	-0.307802

F-anti-0-H
Number of imaginary frequencies: 0
Electronic Energy (SCF) = -1171.75148760 a.u
ZPV corrected Energy = -1171.420706 a.u.
Enthalpy = -1171.399988 a.u.
Free Energy = -1171.472897 a.u.

S	-0.963400	1.691339	0.458936
C	-1.918324	3.211034	-0.024372
H	-1.438068	3.567478	-0.933676
H	-1.786161	3.947051	0.764647
C	-2.364040	0.571375	-0.047796
H	-2.840352	0.247253	0.876479
H	-1.910510	-0.281747	-0.540197
C	-3.302120	1.399414	-0.907127
H	-4.288056	0.938290	-0.966648
H	-2.908125	1.479416	-1.922325
C	-3.358694	2.785568	-0.258536
H	-3.870965	3.511903	-0.889624
H	-3.896865	2.728731	0.689668
C	0.340522	1.544621	-0.577192
C	1.289678	-2.009279	-1.052896
O	1.396547	-2.152111	-2.254599
H	2.127990	-1.611510	-0.458646
C	0.083024	-2.338345	-0.274753
C	0.063571	-2.050582	1.090507
C	-1.035618	-2.917594	-0.880998
C	-1.065692	-2.334007	1.846753
H	0.932014	-1.593729	1.546197
C	-2.158981	-3.206563	-0.123848
H	-1.008757	-3.133235	-1.940308
C	-2.174920	-2.913424	1.239687
H	-1.083050	-2.104087	2.903476
H	-3.025264	-3.657364	-0.589100
H	-3.055577	-3.137276	1.827163
H	0.154069	1.645267	-1.637868
C	1.663870	1.242267	-0.115078
C	2.707175	1.115532	-1.063681
C	2.025158	1.041664	1.238271
C	4.002636	0.799908	-0.688743
H	2.474872	1.252935	-2.112640
C	3.324556	0.728249	1.603914
H	1.270861	1.124147	2.009611
C	4.333673	0.597055	0.650358
H	4.763875	0.704631	-1.453772
H	3.552551	0.578640	2.652644
H	5.344690	0.347336	0.941926

F-anti-TS0-H
Number of imaginary frequencies: 1
Electronic Energy (SCF) = -1171.73938668 a.u
ZPV corrected Energy = -1171.407478 a.u.
Enthalpy = -1171.388458 a.u.
Free Energy = -1171.455822 a.u.

S	-0.060768	1.220984	0.660650
C	0.920208	2.747871	0.261048
H	1.687844	2.431182	-0.440320
H	1.387178	3.092517	1.179392
C	-1.538790	1.791430	-0.258994
H	-2.237739	2.141957	0.497024
H	-1.964350	0.935058	-0.769323
C	-1.064092	2.916606	-1.167385
H	-1.915819	3.512427	-1.494342
H	-0.581291	2.502581	-2.053745
C	-0.066534	3.735661	-0.352946
H	0.468705	4.461355	-0.964946
H	-0.588913	4.280756	0.434744
C	0.576924	-0.105102	-0.342355
C	-0.265802	-1.863074	-0.042576
O	0.106916	-2.602968	-1.000751
H	0.194578	-2.004290	0.956202
C	-1.727461	-1.514958	0.100473
C	-2.252782	-1.127156	1.333319
C	-2.576305	-1.571098	-1.004712
C	-3.591062	-0.768927	1.454672
H	-1.606872	-1.108334	2.203261
C	-3.913551	-1.212100	-0.888448
H	-2.167002	-1.898608	-1.950987
C	-4.424902	-0.802071	0.340936
H	-3.985064	-0.469329	2.417470
H	-4.561195	-1.252636	-1.755178
H	-5.466753	-0.524264	0.432361
H	0.325095	0.085198	-1.381767
C	2.010209	-0.344237	-0.114865
C	2.824881	-0.709145	-1.195179
C	2.588156	-0.321069	1.164238
C	4.161457	-1.036896	-1.005670
H	2.398330	-0.737602	-2.188559
C	3.926675	-0.637735	1.349771
H	1.987247	-0.059713	2.026441
C	4.723083	-0.998968	0.266358
H	4.767095	-1.319292	-1.857278
H	4.348166	-0.608148	2.346309
H	5.765831	-1.247005	0.412743

F-anti-H
Number of imaginary frequencies: 0
Electronic Energy (SCF) = -1171.74564023 a.u
ZPV corrected Energy = -1171.411698 a.u.
Enthalpy = -1171.392681 a.u.
Free Energy = -1171.459328 a.u.

S	-0.015162	1.099090	0.789820
C	1.237539	2.403331	0.423838
H	1.945567	1.970589	-0.277325
H	1.750670	2.632954	1.353145
C	-1.347390	1.943130	-0.148441
H	-1.986604	2.401055	0.603773
H	-1.911605	1.187485	-0.683591
C	-0.653003	2.975144	-1.024412
H	-1.370411	3.726656	-1.352509
H	-0.239357	2.492812	-1.911314
C	0.465213	3.574573	-0.177022

H	1.141690	4.195814	-0.763118
H	0.041800	4.191941	0.616363
C	0.453996	-0.365614	-0.295286
C	-0.396932	-1.674712	0.014090
O	-0.067984	-2.561547	-0.918952
H	-0.115766	-1.948124	1.059754
C	-1.885290	-1.309501	0.074556
C	-2.507524	-0.950117	1.269062
C	-2.640494	-1.310858	-1.097315
C	-3.846254	-0.566633	1.290983
H	-1.940799	-0.975085	2.192653
C	-3.975700	-0.926695	-1.083663
H	-2.156856	-1.627531	-2.011800
C	-4.582690	-0.545150	0.111641
H	-4.315248	-0.292893	2.227726
H	-4.548624	-0.927115	-2.002626
H	-5.623866	-0.250140	0.124475
H	0.218318	-0.046609	-1.308656
C	1.926080	-0.591986	-0.129861
C	2.778445	-0.464856	-1.225885
C	2.469917	-0.928688	1.111614
C	4.145234	-0.681269	-1.090507
H	2.364056	-0.204350	-2.190889
C	3.835955	-1.135793	1.250758
H	1.822954	-1.038056	1.972107
C	4.678418	-1.013750	0.149596
H	4.792171	-0.589290	-1.952949
H	4.243159	-1.397483	2.218472
H	5.742084	-1.179027	0.258331

G-gauche-0-H
Number of imaginary frequencies: 0
Electronic Energy (SCF) = -1171.75332837 a.u
ZPV corrected Energy = -1171.422124 a.u.
Enthalpy = -1171.401617 a.u.
Free Energy = -1171.473359 a.u.

S	2.445536	-0.599811	-1.168961
C	3.704033	0.752737	-0.857567
H	3.182950	1.699201	-0.969377
H	4.434809	0.649729	-1.656968
C	2.761916	-1.375605	0.484798
H	2.624354	-2.446128	0.356309
H	2.022722	-1.004021	1.186942
C	4.179060	-0.969626	0.845682
H	4.897181	-1.544623	0.256628
H	4.380220	-1.170346	1.897948
C	4.310425	0.524518	0.520914
H	3.767918	1.106652	1.266238
H	5.351667	0.845018	0.544435
C	0.892055	-0.017366	-1.375652
C	-0.765711	-2.082059	0.716955
O	-0.235161	-1.975182	1.805497
H	0.476773	-0.349688	-2.315301
C	0.205935	0.998197	-0.631077
C	-1.002131	1.524642	-1.146366
C	0.640408	1.518495	0.605848
C	-1.717933	2.497776	-0.473615
H	-1.373460	1.145435	-2.089870
C	-0.082942	2.497487	1.274610
H	1.545975	1.146542	1.059684
C	-1.267791	3.000941	0.747714
H	-2.641946	2.866029	-0.901904
H	0.285226	2.866278	2.224353
H	-1.829920	3.759949	1.274735
H	-0.301941	-2.692638	-0.076911
C	-2.043418	-1.458049	0.343962
C	-2.570567	-1.700256	-0.924816
C	-2.722726	-0.622623	1.233950
C	-3.773246	-1.119117	-1.302071
H	-2.028160	-2.338131	-1.611388
C	-3.921994	-0.042726	0.856498
H	-2.293444	-0.429764	2.207000
C	-4.448215	-0.291621	-0.410268
H	-4.181880	-1.304373	-2.286247
H	-4.446071	0.611618	1.539834
H	-5.383209	0.167226	-0.703721

G-gauche-TS0-H
Number of imaginary frequencies: 1
Electronic Energy (SCF) = -1171.74127193 a.u
ZPV corrected Energy = -1171.408630 a.u.
Enthalpy = -1171.389841 a.u.
Free Energy = -1171.456249 a.u.

S	1.865423	-1.279758	-0.864491
C	3.155532	-0.050436	-1.351099
H	2.642231	0.832543	-1.721420
H	3.704038	-0.518791	-2.165682
C	2.621095	-1.621772	0.779885
H	2.549074	-2.694458	0.937996
H	2.009722	-1.119198	1.523863
C	4.054924	-1.115953	0.673676
H	4.674958	-1.853266	0.160489
H	4.467812	-0.972205	1.671798
C	4.028716	0.188286	-0.124029
H	3.604563	0.988999	0.478910
H	5.029698	0.489425	-0.431676
C	0.296127	-0.490869	-0.655431
C	-0.591207	-1.438507	0.810181
O	-0.157544	-1.039347	1.935880
H	-0.251201	-0.772827	-1.548766
C	0.221862	0.948572	-0.376403
C	-0.619461	1.746992	-1.164938
C	0.862281	1.547103	0.716147
C	-0.804626	3.092723	-0.882929
H	-1.135451	1.296742	-2.003259
C	0.683323	2.897406	0.991672
H	1.482964	0.954668	1.367245
C	-0.148382	3.678148	0.196287
H	-1.460188	3.685799	-1.507451
H	1.189276	3.338696	1.840851
H	-0.286757	4.728503	0.415946
H	-0.315231	-2.456223	0.465083
C	-2.017643	-1.131071	0.414512
C	-2.659751	-1.860505	-0.585653
C	-2.707942	-0.098418	1.045037
C	-3.965383	-1.561921	-0.955448
H	-2.129482	-2.668882	-1.076686
C	-4.013324	0.206892	0.675656
H	-2.205356	0.454803	1.826139
C	-4.646357	-0.522060	-0.326437
H	-4.454332	-2.139375	-1.729772
H	-4.538056	1.015145	1.169210
H	-5.663393	-0.286841	-0.612106

G-gauche-H
Number of imaginary frequencies: 0
Electronic Energy (SCF) = -1171.75285937 a.u
ZPV corrected Energy = -1171.417648 a.u.
Enthalpy = -1171.399098 a.u.
Free Energy = -1171.463900 a.u.

S	1.847868	-1.537216	-0.419713
C	3.140918	-0.553268	-1.304545

H	2.675135	-0.010929	-2.122634
H	3.832740	-1.285265	-1.718403
C	2.478074	-1.076224	1.231144
H	2.440661	-1.981055	1.828318
H	1.786615	-0.352240	1.646225
C	3.871053	-0.493400	1.021510
H	4.604745	-1.295100	0.921077
H	4.149468	0.109631	1.885588
C	3.816079	0.331600	-0.262928
H	3.225368	1.233152	-0.101530
H	4.808108	0.632519	-0.599123
C	0.301035	-0.583762	-0.835602
C	-0.721042	-1.623831	-0.264265
O	-0.205958	-2.109335	0.887293
H	0.327681	-0.555841	-1.925703
C	0.275520	0.811791	-0.307068
C	0.551801	1.874265	-1.170301
C	-0.041343	1.094145	1.025675
C	0.530007	3.186992	-0.715056
H	0.778350	1.668313	-2.208541
C	-0.065776	2.405806	1.480157
H	-0.279282	0.275425	1.687800
C	0.225513	3.455995	0.614720
H	0.745488	3.997631	-1.398587
H	-0.317062	2.609103	2.512848
H	0.206490	4.476914	0.972547
H	-0.792078	-2.393183	-1.070238
C	-2.122553	-1.020544	-0.180838
C	-2.748899	-0.486592	-1.308737
C	-2.806720	-1.011581	1.029398
C	-4.024163	0.057874	-1.224490
H	-2.233250	-0.493928	-2.262025
C	-4.087879	-0.473271	1.119240
H	-2.311527	-1.433404	1.892858
C	-4.699979	0.067315	-0.005921
H	-4.493282	0.474215	-2.107048
H	-4.608054	-0.474684	2.069065
H	-5.694420	0.488992	0.062016

G-gauche'-0-H
Number of imaginary frequencies: 0
Electronic Energy (SCF) = -1171.75464004 a.u
ZPV corrected Energy = -1171.423096 a.u.
Enthalpy = -1171.402690 a.u.
Free Energy = -1171.473490 a.u.

S	2.346081	0.079727	-0.911140
C	3.339151	1.221938	0.193683
H	2.841409	2.187226	0.178919
H	4.309076	1.311732	-0.290910
C	2.096189	-1.146314	0.455208
H	2.063052	-2.126661	-0.009937
H	1.140401	-0.938770	0.926016
C	3.269070	-0.934802	1.394244
H	4.177183	-1.361148	0.962288
H	3.092913	-1.433535	2.347395
C	3.433088	0.580902	1.572464
H	2.637068	0.959823	2.213850
H	4.383142	0.822899	2.048137
C	0.933021	0.772655	-1.475042
C	-0.618544	-2.147116	-1.705298
O	0.253417	-2.995440	-1.732277
H	0.886219	0.739714	-2.553031
C	-0.054685	1.515106	-0.752218
C	-1.092639	2.155047	-1.471273
C	-0.088053	1.653587	0.651667
C	-2.086581	2.874479	-0.832020
H	-1.103335	2.071682	-2.551307
C	-1.089613	2.374442	1.285731
H	0.669451	1.183009	1.258739
C	-2.101177	2.993829	0.557977
H	-2.862316	3.346552	-1.422688
H	-1.080723	2.447541	2.366449
H	-2.880881	3.552332	1.057781
H	-0.866471	-1.570016	-2.612537
C	-1.440632	-1.819048	-0.530332
C	-2.444419	-0.858228	-0.656109
C	-1.227076	-2.442326	0.703256
C	-3.227267	-0.516376	0.438108
H	-2.600237	-0.376413	-1.611384
C	-2.006323	-2.099142	1.794975
H	-0.446811	-3.185401	0.790331
C	-3.006375	-1.134807	1.662892
H	-3.996778	0.236153	0.339815
H	-1.840274	-2.576901	2.751339
H	-3.609525	-0.864887	2.519718

G-gauche'-TS0-H
Number of imaginary frequencies: 1
Electronic Energy (SCF) = -1171.73810950 a.u
ZPV corrected Energy = -1171.405377 a.u.
Enthalpy = -1171.386611 a.u.
Free Energy = -1171.453279 a.u.

S	2.224569	-0.453593	-0.823212
C	3.158733	0.892797	0.023984
H	2.684136	1.840042	-0.215207
H	4.155973	0.869580	-0.410364
C	2.044686	-1.468641	0.692691
H	2.148345	-2.502522	0.385345
H	1.048400	-1.317086	1.088880
C	3.137326	-0.967831	1.630103
H	4.100883	-1.389129	1.337852
H	2.926891	-1.294872	2.648085
C	3.171356	0.555358	1.513306
H	2.294576	0.985630	1.994546
H	4.057560	0.978400	1.985042
C	0.647251	0.196950	-1.337254
C	-0.366935	-1.467807	-1.669128
O	0.436902	-2.458894	-1.686237
H	0.842879	0.478911	-2.368922
C	0.018767	1.301088	-0.597707
C	-0.456236	2.402910	-1.326568
C	-0.238736	1.279060	0.780889
C	-1.161496	3.428532	-0.711923
H	-0.271995	2.443400	-2.392856
C	-0.927372	2.312901	1.398542
H	0.074973	0.439186	1.379557
C	-1.398413	3.393017	0.658431
H	-1.519690	4.260915	-1.304006
H	-1.112928	2.264735	2.463756
H	-1.943614	4.192481	1.141909
H	-0.751079	-1.086497	-2.634342
C	-1.462943	-1.409454	-0.632981
C	-2.538477	-0.533806	-0.783536
C	-1.429592	-2.241147	0.484330
C	-3.543767	-0.468300	0.172340
H	-2.580846	0.107802	-1.654761
C	-2.431259	-2.178215	1.446761
H	-0.612118	-2.942240	0.579586
C	-3.489796	-1.286799	1.297297
H	-4.367408	0.222115	0.043351
H	-2.391349	-2.829145	2.311227
H	-4.270489	-1.236772	2.045152

G-gauche'-H
Number of imaginary frequencies: 0
Electronic Energy (SCF) = -1171.74916933 a.u
ZPV corrected Energy = -1171.413927 a.u.
Enthalpy = -1171.395404 a.u.
Free Energy = -1171.460365 a.u.

S	1.831481	-1.337548	-0.748810
C	3.164923	-0.190520	-0.140267
H	3.073355	0.774677	-0.630792
H	4.095879	-0.656892	-0.459576
C	1.430022	-1.911801	0.939578
H	1.266274	-2.980935	0.857211
H	0.499246	-1.440819	1.233467
C	2.596195	-1.512049	1.835637
H	3.407738	-2.234621	1.732253
H	2.279085	-1.514699	2.878623
C	3.064373	-0.130317	1.379696
H	2.337527	0.625026	1.674107
H	4.024195	0.138330	1.821304
C	0.549537	-0.119261	-1.338961
C	-0.662072	-1.124342	-1.548819
O	-0.139555	-2.371428	-1.444971
H	0.961732	0.100165	-2.324370
C	0.432469	1.174422	-0.599284
C	0.816222	2.349939	-1.250725
C	-0.067924	1.275549	0.703299
C	0.702484	3.588454	-0.630109
H	1.205003	2.289546	-2.259384
C	-0.173479	2.509004	1.329528
H	-0.393964	0.393707	1.230395
C	0.208679	3.670978	0.666091
H	1.000897	4.484421	-1.158199
H	-0.564768	2.563456	2.336706
H	0.117855	4.631805	1.155078
H	-1.025543	-0.882435	-2.568938
C	-1.853201	-0.839316	-0.633731
C	-2.670780	0.271103	-0.845474
C	-2.158875	-1.700034	0.415130
C	-3.747180	0.535965	-0.008298
H	-2.455500	0.940913	-1.669570
C	-3.233983	-1.440415	1.260446
H	-1.541939	-2.577392	0.548316
C	-4.028326	-0.316790	1.056500
H	-4.366973	1.406108	-0.183796
H	-3.456023	-2.118260	2.075330
H	-4.865835	-0.113328	1.711140

G-anti-0-H
Number of imaginary frequencies: 0
Electronic Energy (SCF) = -1171.75500420 a.u
ZPV corrected Energy = -1171.423100 a.u.
Enthalpy = -1171.402795 a.u.
Free Energy = -1171.473456 a.u.

S	1.269537	-1.511278	-1.228807
C	2.811380	-2.058306	-0.314552
H	3.451378	-1.185615	-0.222751
H	3.286884	-2.782426	-0.972940
C	0.159279	-2.113054	0.128984
H	-0.748351	-2.467229	-0.349301
H	-0.077063	-1.270420	0.771832
C	0.949050	-3.196510	0.840362
H	0.955016	-4.109501	0.240913
H	0.493230	-3.436296	1.800980
C	2.381880	-2.673104	1.011151
H	2.403439	-1.918798	1.797785
H	3.061648	-3.472718	1.304380
C	1.191198	0.138797	-1.480273
C	-1.997239	0.856331	-1.821054
H	-1.358124	1.754075	-1.850826
O	-2.390377	0.340080	-2.849593
C	-2.334189	0.390525	-0.465452
C	-3.124989	-0.745678	-0.268108
C	-1.837211	1.091824	0.634009
C	-3.402553	-1.179746	1.016622
H	-3.502665	-1.279926	-1.129134
C	-2.114935	0.654063	1.922296
H	-1.222509	1.966584	0.473550
C	-2.893032	-0.482553	2.113019
H	-4.009660	-2.061430	1.172753
H	-1.720336	1.192595	2.773092
H	-3.106978	-0.827380	3.116155
H	1.029621	0.369674	-2.521879
C	1.366407	1.201945	-0.539399
C	1.251424	2.539939	-0.988895
C	1.621268	1.023755	0.837379
C	1.358318	3.612020	-0.120726
H	1.064556	2.720424	-2.040351
C	1.728387	2.104392	1.701637
H	1.722356	0.030092	1.244130
C	1.592702	3.410394	1.240593
H	1.255815	4.618671	-0.507439
H	1.916420	1.920181	2.752447
H	1.672879	4.249043	1.918698

G-anti-TS0-H
Number of imaginary frequencies: 1
Electronic Energy (SCF) = -1171.73625713 a.u
ZPV corrected Energy = -1171.403828 a.u.
Enthalpy = -1171.384930 a.u.
Free Energy = -1171.452026 a.u.

S	-0.180787	1.214720	0.703619
C	1.235226	2.128170	1.466680
H	1.830836	1.401348	2.012632
H	0.786876	2.829734	2.168366
C	-0.040440	2.103356	-0.901848
H	-1.044721	2.393815	-1.192755
H	0.351726	1.402496	-1.630091
C	0.890762	3.287229	-0.656081
H	0.326250	4.119279	-0.232420
H	1.318055	3.621151	-1.601062
C	1.964352	2.831276	0.330881
H	2.651763	2.137993	-0.151839
H	2.542426	3.671559	0.714832
C	0.257480	-0.478852	0.444393
C	-0.894314	-1.427069	-0.858732
H	-0.542325	-0.865805	-1.746588
O	-0.610904	-2.655101	-0.760123
C	-2.260465	-0.942481	-0.431439
C	-2.912505	-1.531935	0.652402
C	-2.899643	0.086239	-1.120808
C	-4.162683	-1.082185	1.055043
H	-2.426762	-2.349450	1.168640
C	-4.155610	0.536478	-0.725178
H	-2.411949	0.531965	-1.979367
C	-4.788335	-0.042177	0.369497
H	-4.655237	-1.542825	1.902069
H	-4.642330	1.332516	-1.274303
H	-5.764808	0.305091	0.680946
H	-0.107540	-0.965885	1.345274
C	1.676506	-0.802944	0.175061
C	2.421310	-1.489608	1.141959
C	2.301141	-0.514610	-1.045608
C	3.742096	-1.855137	0.911105

H	1.952991	-1.736024	2.086324
C	3.623415	-0.869420	-1.274646
H	1.746525	-0.027790	-1.833745
C	4.353459	-1.538632	-0.296729
H	4.293430	-2.385653	1.676678
H	4.081848	-0.635303	-2.226771
H	5.382262	-1.819105	-0.479454

G-anti-H
Number of imaginary frequencies: 0
Electronic Energy (SCF) = -1171.74416996 a.u
ZPV corrected Energy = -1171.409815 a.u.
Enthalpy = -1171.390886 a.u.
Free Energy = -1171.456955 a.u.

S	-0.184142	1.235463	0.545912
C	1.240997	1.962042	1.450972
H	1.642572	1.209037	2.122457
H	0.815962	2.774698	2.037616
C	0.311437	1.944972	-1.071759
H	-0.584087	2.339187	-1.541878
H	0.710375	1.134332	-1.672746
C	1.368775	3.001112	-0.756932
H	0.886588	3.934097	-0.462204
H	1.963395	3.196566	-1.648593
C	2.216492	2.461245	0.392744
H	2.842239	1.639167	0.048379
H	2.863495	3.228635	0.816434
C	0.118996	-0.621568	0.387352
C	-0.896726	-1.277338	-0.636030
H	-0.650919	-0.832963	-1.629864
O	-0.734270	-2.595902	-0.551003
C	-2.311642	-0.774876	-0.316023
C	-2.985725	-1.256884	0.806973
C	-2.957922	0.151110	-1.131540
C	-4.261646	-0.807010	1.118843
H	-2.500964	-2.003377	1.422617
C	-4.241656	0.601427	-0.830329
H	-2.453298	0.518014	-2.018035
C	-4.895207	0.128002	0.300821
H	-4.769544	-1.187266	1.996427
H	-4.731196	1.315326	-1.480880
H	-5.892938	0.472968	0.539324
H	-0.180208	-0.949245	1.382673
C	1.562548	-0.931385	0.145056
C	2.415349	-1.140532	1.230169
C	2.095766	-0.995365	-1.144571
C	3.772179	-1.373484	1.038701
H	2.009050	-1.126617	2.233402
C	3.450348	-1.227247	-1.339072
H	1.446270	-0.884654	-2.000455
C	4.295882	-1.407453	-0.248225
H	4.416746	-1.533169	1.892953
H	3.846067	-1.276823	-2.344920
H	5.351365	-1.588713	-0.402185

H-gauche-0-H
Number of imaginary frequencies: 0
Electronic Energy (SCF) = -1171.75357212 a.u
ZPV corrected Energy = -1171.422162 a.u.
Enthalpy = -1171.401804 a.u.
Free Energy = -1171.472748 a.u.

S	2.114128	-1.091964	-0.886700
C	3.581507	-0.034941	-1.305046
H	3.269778	1.003739	-1.222002
H	3.840157	-0.256663	-2.337236
C	2.668698	-1.290238	0.889304
H	2.827379	-2.360136	1.009703
H	1.846410	-0.980538	1.526590
C	3.955498	-0.495551	1.070918
H	4.597076	-0.969233	1.813571
H	3.729211	0.509595	1.426883
C	4.650114	-0.407880	-0.294440
H	5.451963	0.330562	-0.288872
H	5.090728	-1.373183	-0.553365
C	0.657780	-0.290805	-1.072475
C	-0.890503	-1.607711	1.251148
O	-0.437874	-1.052090	2.234841
H	0.008889	-0.817579	-1.754506
C	0.271032	1.002083	-0.592291
C	-0.936608	1.570878	-1.060161
C	0.996374	1.755763	0.354325
C	-1.376243	2.807371	-0.621973
H	-1.529124	1.015722	-1.776242
C	0.549744	2.996627	0.787857
H	1.914799	1.366750	0.765307
C	-0.637517	3.540589	0.306860
H	-2.309397	3.202814	-1.004148
H	1.136597	3.541733	1.517181
H	-0.982356	4.506851	0.649417
H	-0.369669	-2.470301	0.801969
C	-2.161158	-1.252738	0.599937
C	-2.924725	-0.174159	1.051972
C	-2.599207	-2.004266	-0.491521
C	-4.119234	0.139492	0.422940
H	-2.565840	0.411117	1.886879
C	-3.792511	-1.687072	-1.124326
H	-1.994585	-2.831541	-0.841910
C	-4.553364	-0.615015	-0.664859
H	-4.709334	0.977241	0.769379
H	-4.130154	-2.268726	-1.971510
H	-5.483652	-0.364648	-1.157624

H-gauche-TS0-H
Number of imaginary frequencies: 1
Electronic Energy (SCF) = -1171.74275720 a.u
ZPV corrected Energy = -1171.409983 a.u.
Enthalpy = -1171.391318 a.u.
Free Energy = -1171.457092 a.u.

S	1.888774	-1.280583	-0.790428
C	3.161588	-0.081603	-1.363840
H	2.746601	0.914273	-1.225918
H	3.335351	-0.267146	-2.420199
C	2.660073	-1.500469	0.872971
H	2.991339	-2.536700	0.878885
H	1.868234	-1.361360	1.609978
C	3.832148	-0.527503	0.950748
H	4.596955	-0.922469	1.618552
H	3.502245	0.429795	1.351807
C	4.368229	-0.334238	-0.469756
H	5.058562	0.506562	-0.533372
H	4.898934	-1.229170	-0.799119
C	0.309506	-0.501846	-0.643436
C	-0.592700	-1.449449	0.817570
O	-0.168019	-1.051222	1.948093
H	-0.216615	-0.813802	-1.539662
C	0.203827	0.938504	-0.386152
C	-0.640372	1.714366	-1.193297
C	0.820007	1.556702	0.708758
C	-0.852205	3.058946	-0.924572
H	-1.137879	1.248278	-2.034140
C	0.613580	2.905421	0.971917
H	1.440811	0.976322	1.370642

C	-0.221016	3.664554	0.158615
H	-1.509641	3.635646	-1.562327
H	1.100263	3.362441	1.824018
H	-0.380739	4.714115	0.367243
H	-0.309751	-2.465184	0.473049
C	-2.015264	-1.143111	0.412638
C	-2.710098	-0.109471	1.036733
C	-2.649915	-1.874224	-0.591055
C	-4.012776	0.194933	0.657575
H	-2.213407	0.445708	1.820143
C	-3.952914	-1.576280	-0.970537
H	-2.116169	-2.683341	-1.076910
C	-4.638465	-0.535685	-0.347900
H	-4.541017	1.003936	1.146065
H	-4.436244	-2.155044	-1.747369
H	-5.653468	-0.301058	-0.641145

H-gauche-H
Number of imaginary frequencies: 0
Electronic Energy (SCF) = -1171.75288569 a.u
ZPV corrected Energy = -1171.417864 a.u.
Enthalpy = -1171.399259 a.u.
Free Energy = -1171.464482 a.u.

S	-1.845772	-1.553794	0.289342
C	-3.118266	-0.689161	1.308056
H	-2.661014	0.195240	1.743881
H	-3.423508	-1.366734	2.101265
C	-2.502839	-0.893735	-1.287136
H	-2.928491	-1.766752	-1.779605
H	-1.661317	-0.543972	-1.869259
C	-3.553086	0.152236	-0.941183
H	-4.257804	0.259603	-1.765468
H	-3.072360	1.116830	-0.777574
C	-4.237809	-0.316033	0.341660
H	-4.871222	0.455897	0.777679
H	-4.863837	-1.186269	0.136235
C	-0.293942	-0.641184	0.755243
C	0.734219	-1.637761	0.112909
O	0.254333	-2.020805	-1.088161
H	-0.311495	-0.681332	1.845537
C	-0.275815	0.784954	0.309197
C	-0.551588	1.797140	1.231691
C	0.035342	1.143561	-1.006462
C	-0.538907	3.133736	0.849913
H	-0.766615	1.534572	2.259580
C	0.051085	2.478027	-1.386954
H	0.276750	0.363741	-1.712294
C	-0.243266	3.477672	-0.463879
H	-0.753942	3.903717	1.579050
H	0.298323	2.739823	-2.407416
H	-0.231927	4.516850	-0.765010
H	0.782523	-2.472868	0.853210
C	2.137911	-1.030609	0.122892
C	2.847476	-0.900142	-1.065380
C	2.738722	-0.611908	1.311660
C	4.128844	-0.354910	-1.073789
H	2.370909	-1.235011	-1.976207
C	4.014141	-0.061130	1.309487
H	2.203509	-0.715887	2.248425
C	4.715501	0.070711	0.112677
H	4.668998	-0.260914	-2.007699
H	4.463740	0.264427	2.239137
H	5.710129	0.497406	0.108738

H-gauche'-0-H
Number of imaginary frequencies: 0
Electronic Energy (SCF) = -1171.75450342 a.u
ZPV corrected Energy = -1171.422910 a.u.
Enthalpy = -1171.402546 a.u.
Free Energy = -1171.473104 a.u.

S	2.338233	-0.082817	-0.898839
C	3.438856	1.098022	0.015690
H	2.818681	1.920471	0.365411
H	4.163789	1.469001	-0.704274
C	2.102829	-1.151736	0.617263
H	2.485205	-2.128996	0.334271
H	1.036808	-1.227519	0.797740
C	2.891059	-0.526797	1.761843
H	3.266688	-1.297501	2.434391
H	2.246789	0.131829	2.345030
C	4.037121	0.291985	1.153046
H	4.498292	0.946775	1.892468
H	4.813013	-0.375288	0.771551
C	0.938615	0.640181	-1.458035
C	-0.621564	-2.261929	-1.568218
O	0.222601	-3.129782	-1.445705
H	0.843172	0.529931	-2.527541
C	-0.004047	1.449075	-0.746021
C	-0.997740	2.146531	-1.473628
C	-0.044458	1.584438	0.658034
C	-1.954823	2.921182	-0.842315
H	-1.003465	2.064894	-2.553811
C	-1.008423	2.361377	1.283641
H	0.675594	1.065316	1.270869
C	-1.974579	3.040577	0.547347
H	-2.697331	3.436803	-1.439250
H	-1.007880	2.429837	2.364698
H	-2.725436	3.642485	1.040903
H	-0.782053	-1.770356	-2.542986
C	-1.519109	-1.802527	-0.497165
C	-2.465149	-0.817392	-0.782621
C	-1.429749	-2.325102	0.797201
C	-3.313287	-0.353243	0.213131
H	-2.523502	-0.412843	-1.783791
C	-2.274750	-1.859731	1.790501
H	-0.694283	-3.089495	1.006676
C	-3.216521	-0.872475	1.498684
H	-4.036884	0.418590	-0.007466
H	-2.204476	-2.259226	2.793423
H	-3.870938	-0.506001	2.278491

H-gauche'-TS0-H
Number of imaginary frequencies: 1
Electronic Energy (SCF) = -1171.73893898 a.u
ZPV corrected Energy = -1171.406147 a.u.
Enthalpy = -1171.387479 a.u.
Free Energy = -1171.453355 a.u.

S	2.199207	-0.569927	-0.793645
C	3.227032	0.800202	-0.142618
H	2.570028	1.632156	0.101275
H	3.920973	1.095047	-0.925237
C	2.034963	-1.391467	0.845208
H	2.491423	-2.367048	0.702981
H	0.983499	-1.532532	1.057370
C	2.792042	-0.531403	1.856197
H	3.200510	-1.161344	2.645437
H	2.114160	0.186375	2.316882
C	3.889974	0.217511	1.099545
H	4.328458	1.014877	1.698767
H	4.691034	-0.466823	0.814008
C	0.641746	0.110257	-1.319344
C	-0.381157	-1.575771	-1.561050

O	0.411155	-2.568468	-1.427964
H	0.832912	0.360426	-2.360427
C	0.037626	1.237239	-0.597112
C	-0.405809	2.347288	-1.332086
C	-0.222244	1.226598	0.781010
C	-1.083489	3.394298	-0.722104
H	-0.219327	2.377655	-2.398363
C	-0.884409	2.280359	1.393636
H	0.064483	0.376106	1.378277
C	-1.323658	3.370296	0.647988
H	-1.418180	4.234012	-1.317598
H	-1.074661	2.240991	2.458388
H	-1.847946	4.186177	1.127166
H	-0.697456	-1.290398	-2.581577
C	-1.531920	-1.401869	-0.602647
C	-2.567154	-0.510239	-0.885929
C	-1.585064	-2.133169	0.582060
C	-3.616144	-0.330193	0.005507
H	-2.541813	0.054662	-1.809461
C	-2.631800	-1.955361	1.480441
H	-0.800204	-2.850327	0.780255
C	-3.648191	-1.047767	1.198654
H	-4.407095	0.371655	-0.225747
H	-2.658466	-2.528792	2.398560
H	-4.462946	-0.907153	1.896924

H-gauche'-H
Number of imaginary frequencies: 0
Electronic Energy (SCF) = -1171.74879166 a.u
ZPV corrected Energy = -1171.413842 a.u.
Enthalpy = -1171.395215 a.u.
Free Energy = -1171.460712 a.u.

S	1.929436	-1.141764	-0.743230
C	3.179055	0.111361	-0.202283
H	2.701790	1.072290	-0.027528
H	3.909563	0.209273	-1.002370
C	1.631295	-1.720763	0.972255
H	1.885796	-2.777514	0.934287
H	0.573600	-1.638526	1.184019
C	2.539488	-0.923925	1.905081
H	2.830195	-1.536729	2.757949
H	2.005020	-0.052463	2.282932
C	3.748185	-0.465279	1.088370
H	4.340674	0.281281	1.616681
H	4.400555	-1.313575	0.870192
C	0.536201	-0.064772	-1.332884
C	-0.576249	-1.190715	-1.522893
O	0.052797	-2.381911	-1.377276
H	0.912927	0.197626	-2.322326
C	0.285729	1.203949	-0.585272
C	0.464220	2.419227	-1.249883
C	-0.140508	1.233608	0.746175
C	0.216694	3.630236	-0.613020
H	0.797703	2.412489	-2.279662
C	-0.374077	2.440126	1.389264
H	-0.306978	0.313224	1.282002
C	-0.200596	3.643985	0.712089
H	0.355221	4.559052	-1.150448
H	-0.704223	2.440365	2.419550
H	-0.391264	4.583164	1.214224
H	-0.950898	-1.012846	-2.551955
C	-1.794855	-0.981915	-0.622196
C	-2.688330	0.064631	-0.853290
C	-2.048779	-1.851973	0.432514
C	-3.789711	0.258830	-0.029775
H	-2.513711	0.740759	-1.681755
C	-3.149215	-1.663047	1.264300
H	-1.372099	-2.682013	0.580047
C	-4.020382	-0.602173	1.040722
H	-4.468546	1.080469	-0.220034
H	-3.330348	-2.347678	2.083598
H	-4.877317	-0.453478	1.684856

H-anti-0-H
Number of imaginary frequencies: 0
Electronic Energy (SCF) = -1171.75514664 a.u
ZPV corrected Energy = -1171.423355 a.u.
Enthalpy = -1171.403002 a.u.
Free Energy = -1171.473517 a.u.

S	-1.495867	1.237317	-1.181988
C	-3.170291	1.464768	-0.413800
H	-3.436078	0.536476	0.086519
H	-3.863832	1.649483	-1.230103
C	-0.633191	1.988406	0.299144
H	-0.089192	2.842066	-0.097125
H	0.075115	1.254470	0.665641
C	-1.698069	2.394367	1.310510
H	-1.391903	3.287838	1.853848
H	-1.839306	1.597943	2.041588
C	-3.010641	2.626392	0.549166
H	-3.861359	2.687157	1.227909
H	-2.961416	3.566797	-0.004146
C	-1.102499	-0.363540	-1.453826
C	2.147638	-0.405108	-1.852600
H	1.730182	-1.424899	-1.889714
O	2.404839	0.197489	-2.877010
C	2.380270	0.110273	-0.493028
C	2.070444	-0.701247	0.599477
C	2.887415	1.396617	-0.284440
C	2.253684	-0.228189	1.892263
H	1.671726	-1.691886	0.429782
C	3.071583	1.865718	1.004852
H	3.120865	2.015650	-1.139776
C	2.750418	1.054892	2.094354
H	2.002421	-0.854101	2.737621
H	3.459308	2.862125	1.169553
H	2.889404	1.426897	3.100917
H	-0.827823	-0.530565	-2.483732
C	-1.075558	-1.454582	-0.528966
C	-0.703460	-2.738523	-0.996488
C	-1.351751	-1.346538	0.851116
C	-0.596833	-3.822293	-0.142923
H	-0.488174	-2.865272	-2.050363
C	-1.244063	-2.438872	1.700492
H	-1.641056	-0.396482	1.271908
C	-0.861380	-3.688008	1.221160
H	-0.302196	-4.784704	-0.543378
H	-1.458002	-2.307924	2.754347
H	-0.775799	-4.535267	1.887801

H-anti-TS0-H
Number of imaginary frequencies: 1
Electronic Energy (SCF) = -1171.73725896 a.u
ZPV corrected Energy = -1171.404713 a.u.
Enthalpy = -1171.385859 a.u.
Free Energy = -1171.452450 a.u.

S	0.228313	1.165782	-0.706779
C	-1.104927	2.206165	-1.441989
H	-2.031864	1.644194	-1.351102
H	-0.861963	2.352023	-2.491026
C	0.186223	2.057032	0.909689
H	1.148973	2.558365	0.971628

H	0.121992	1.324275	1.706558
C	-0.970425	3.049600	0.847462
H	-0.767504	3.896128	1.502391
H	-1.890594	2.574846	1.185186
C	-1.120536	3.481773	-0.611730
H	-2.049553	4.025797	-0.780708
H	-0.293423	4.131516	-0.902756
C	-0.324456	-0.501290	-0.492645
C	0.758549	-1.526330	0.759625
H	0.389606	-1.015030	1.672202
O	0.462001	-2.746076	0.584577
C	2.152588	-1.049741	0.414695
C	2.797926	-0.098788	1.202963
C	2.819937	-1.564604	-0.697614
C	4.074236	0.350654	0.876790
H	2.298071	0.288355	2.082384
C	4.090005	-1.114713	-1.031275
H	2.329507	-2.323098	-1.293357
C	4.721442	-0.150616	-0.246832
H	4.564319	1.086153	1.502095
H	4.593518	-1.515690	-1.901875
H	5.713124	0.197058	-0.505231
H	0.010677	-0.990312	-1.403218
C	-1.747153	-0.760826	-0.203659
C	-2.474769	-1.602067	-1.054502
C	-2.390099	-0.277180	0.943411
C	-3.795625	-1.935186	-0.781324
H	-1.993561	-1.996417	-1.940101
C	-3.713854	-0.598715	1.210777
H	-1.851600	0.339213	1.646949
C	-4.426101	-1.428418	0.349711
H	-4.333266	-2.589477	-1.455398
H	-4.187206	-0.210289	2.103334
H	-5.456028	-1.682006	0.562569

H-anti-H
Number of imaginary frequencies: 0
Electronic Energy (SCF) = -1171.74352571 a.u
ZPV corrected Energy = -1171.409059 a.u.
Enthalpy = -1171.390138 a.u.
Free Energy = -1171.456442 a.u.

S	-0.205596	1.234809	0.478476
C	1.144511	2.036971	1.428191
H	1.899730	1.281552	1.627982
H	0.717594	2.388685	2.363351
C	0.337405	1.919208	-1.136947
H	-0.428081	2.642385	-1.409798
H	0.341441	1.115921	-1.866052
C	1.694909	2.573283	-0.897427
H	1.869183	3.346626	-1.644654
H	2.484213	1.828200	-0.986708
C	1.671635	3.143618	0.519073
H	2.662358	3.450950	0.852398
H	1.014545	4.013032	0.566677
C	0.141361	-0.616572	0.403931
C	-0.868956	-1.352090	-0.577095
H	-0.617284	-0.980795	-1.598734
O	-0.699167	-2.658551	-0.393307
C	-2.286679	-0.830165	-0.303746
C	-2.911332	0.069826	-1.164510
C	-2.980166	-1.259222	0.828306
C	-4.192857	0.546838	-0.897966
H	-2.390918	0.396792	-2.057585
C	-4.254575	-0.782281	1.105423
H	-2.509853	-1.985340	1.478621
C	-4.866168	0.126802	0.242935
H	-4.665417	1.240271	-1.582320
H	-4.778151	-1.120821	1.990858
H	-5.862234	0.493336	0.454760
H	-0.149353	-0.902992	1.414558
C	1.587151	-0.919695	0.167696
C	2.434388	-1.138260	1.255609
C	2.125067	-0.982571	-1.119666
C	3.787776	-1.396924	1.067909
H	2.024998	-1.116057	2.257476
C	3.475718	-1.237566	-1.310057
H	1.483257	-0.847733	-1.977981
C	4.314080	-1.440277	-0.217124
H	4.426650	-1.568228	1.924173
H	3.874491	-1.287446	-2.314702
H	5.366101	-1.642414	-0.368691

A-TS1-H
Number of imaginary frequencies: 1
Electronic Energy (SCF) = -1171.74228263 a.u
ZPV corrected Energy = -1171.408362 a.u.
Enthalpy = -1171.390209 a.u.
Free Energy = -1171.454431 a.u.

S	1.926579	-0.372258	-0.919565
C	3.082732	1.043522	-0.800343
H	2.494547	1.935971	-0.603049
H	3.550623	1.130950	-1.779703
C	3.088327	-1.560222	-0.122322
H	3.162428	-2.430658	-0.766802
H	2.621682	-1.842865	0.819412
C	4.409057	-0.810773	0.062621
H	5.024211	-0.920769	-0.831147
H	4.954514	-1.240222	0.901866
C	4.080353	0.664118	0.285284
H	3.636497	0.813577	1.269922
H	4.966073	1.294661	0.216325
C	0.745835	-0.086820	0.442845
C	0.007030	-1.530200	0.759256
O	0.194812	-1.829127	2.031046
H	0.492116	-2.234592	0.031356
C	-1.447233	-1.450653	0.289730
C	-1.816208	-1.854868	-0.989801
C	-2.430559	-0.954085	1.142887
C	-3.136063	-1.751670	-1.421393
H	-1.061520	-2.255338	-1.657660
C	-3.747940	-0.840802	0.718139
H	-2.143617	-0.663015	2.144146
C	-4.106209	-1.236280	-0.569251
H	-3.406577	-2.075012	-2.418814
H	-4.499497	-0.443566	1.388983
H	-5.133526	-1.152368	-0.899480
H	1.357641	0.064548	1.326656
C	-0.098019	1.116075	0.182800
C	-0.307507	2.017852	1.228314
C	-0.750905	1.338209	-1.033546
C	-1.167487	3.099186	1.073337
H	0.194777	1.856668	2.173141
C	-1.599574	2.423954	-1.192782
H	-0.613076	0.650911	-1.856505
C	-1.817104	3.306001	-0.137746
H	-1.326451	3.780488	1.898842
H	-2.100093	2.577026	-2.139728
H	-2.485678	4.147482	-0.261256

A-TS2-H
Number of imaginary frequencies: 1
Electronic Energy (SCF) = -1171.73559559 a.u
ZPV corrected Energy = -1171.401853 a.u.

Enthalpy = -1171.383653 a.u.
Free Energy = -1171.447673 a.u.

S	0.149385	1.060183	-0.644324
C	-1.061469	2.411562	-0.937141
H	-2.058026	1.996256	-0.819094
H	-0.918229	2.708304	-1.975290
C	1.354774	2.169752	0.201564
H	2.329846	1.995774	-0.240516
H	1.370758	1.860084	1.241956
C	0.830823	3.594085	0.025042
H	1.177507	4.001226	-0.925593
H	1.220446	4.225221	0.823018
C	-0.693866	3.523514	0.032499
H	-1.061924	3.286791	1.031661
H	-1.149464	4.462468	-0.280300
C	-0.563048	0.086971	0.738413
C	0.471055	-1.115949	1.332710
O	-0.126286	-2.282832	1.376368
H	0.704326	-0.660922	2.324668
C	1.799675	-1.114208	0.563585
C	2.950923	-0.553977	1.112734
C	1.898916	-1.739465	-0.680852
C	4.166417	-0.592114	0.433883
H	2.895074	-0.085822	2.088758
C	3.103036	-1.771103	-1.371238
H	1.021302	-2.217335	-1.094419
C	4.244313	-1.193355	-0.816809
H	5.050761	-0.157824	0.883133
H	3.157738	-2.251910	-2.339914
H	5.185527	-1.225362	-1.350107
H	-0.715540	0.814956	1.532155
C	-1.880773	-0.447829	0.283958
C	-3.045262	-0.062379	0.947639
C	-1.981718	-1.340152	-0.785900
C	-4.285364	-0.558518	0.559309
H	-2.977170	0.626539	1.779971
C	-3.219772	-1.826938	-1.183616
H	-1.090957	-1.656001	-1.307548
C	-4.375898	-1.440843	-0.510957
H	-5.177460	-0.252974	1.090187
H	-3.282171	-2.517672	-2.014322
H	-5.338791	-1.826401	-0.819092

B-TS1-H
Number of imaginary frequencies: 1
Electronic Energy (SCF) = -1171.74192557 a.u
ZPV corrected Energy = -1171.408352 a.u.
Enthalpy = -1171.390080 a.u.
Free Energy = -1171.454893 a.u.

S	1.929526	-0.421994	-0.865237
C	3.042391	1.045415	-0.803420
H	2.627054	1.714590	-0.053500
H	3.011505	1.528567	-1.775735
C	3.123588	-1.537563	-0.030168
H	3.527204	-2.180925	-0.808749
H	2.563477	-2.132999	0.687099
C	4.180651	-0.627455	0.583234
H	5.088565	-1.196493	0.779861
H	3.820860	-0.228883	1.532375
C	4.416298	0.505632	-0.412037
H	5.018560	1.308497	0.011549
H	4.932910	0.126847	-1.295118
C	0.727886	-0.155200	0.482031
C	-0.053331	-1.590534	0.715650
O	0.100184	-1.955744	1.974087
H	0.430793	-2.270529	-0.037120
C	-1.494941	-1.449218	0.220021
C	-2.487858	-0.993105	1.084336
C	-1.840382	-1.749738	-1.094328
C	-3.791188	-0.815999	0.637699
H	-2.218626	-0.783337	2.110563
C	-3.145715	-1.581428	-1.548421
H	-1.078651	-2.119014	-1.772009
C	-4.125221	-1.105804	-0.683738
H	-4.550500	-0.450943	1.318055
H	-3.397893	-1.823086	-2.573405
H	-5.141323	-0.971289	-1.031509
H	1.326575	-0.055909	1.382892
C	-0.081653	1.077688	0.256161
C	-0.333913	1.913964	1.345509
C	-0.664303	1.388940	-0.975937
C	-1.167834	3.018636	1.215507
H	0.113920	1.683188	2.303097
C	-1.485820	2.498851	-1.109647
H	-0.492797	0.753007	-1.833520
C	-1.746836	3.315300	-0.012605
H	-1.361317	3.648242	2.074022
H	-1.931104	2.722137	-2.070117
H	-2.394021	4.175895	-0.116712

B-TS2-H
Number of imaginary frequencies: 1
Electronic Energy (SCF) = -1171.73478089 a.u
ZPV corrected Energy = -1171.401236 a.u.
Enthalpy = -1171.383001 a.u.
Free Energy = -1171.447158 a.u.

S	0.166291	1.052832	-0.557430
C	-1.101579	2.349950	-0.897510
H	-1.927252	2.170671	-0.213954
H	-1.443656	2.211924	-1.919166
C	1.279242	2.206299	0.342475
H	2.125712	2.376038	-0.319106
H	1.623255	1.701764	1.237785
C	0.470762	3.473122	0.593885
H	1.143571	4.312015	0.768412
H	-0.150022	3.347625	1.481995
C	-0.408545	3.681938	-0.635230
H	-1.154327	4.461752	-0.483866
H	0.204416	3.964292	-1.492638
C	-0.540705	0.009764	0.777253
C	0.475118	-1.264040	1.251334
O	-0.117494	-2.427346	1.133796
H	0.675742	-0.931739	2.297865
C	1.822502	-1.162312	0.521150
C	1.968628	-1.710076	-0.754582
C	2.937755	-0.581993	1.121419
C	3.183092	-1.647918	-1.424293
H	1.115813	-2.199463	-1.205542
C	4.163282	-0.524615	0.461889
H	2.847629	-0.177173	2.123185
C	4.287615	-1.050215	-0.818722
H	3.274976	-2.069667	-2.417407
H	5.019619	-0.076281	0.949922
H	5.237191	-1.008568	-1.336310
H	-0.657210	0.690422	1.618393
C	-1.876113	-0.470141	0.317369
C	-3.023251	-0.123108	1.029923
C	-2.004315	-1.284154	-0.810807
C	-4.275243	-0.579636	0.630259
H	-2.932567	0.502136	1.909008
C	-3.254248	-1.729348	-1.219275
H	-1.123753	-1.573976	-1.364395
C	-4.393992	-1.381523	-0.498772

H	-5.154590	-0.305500	1.198255
H	-3.339029	-2.359311	-2.095092
H	-5.366244	-1.735467	-0.815430

C-TS1-H

Number of imaginary frequencies: 1
Electronic Energy (SCF) = -1171.73596022 a.u
ZPV corrected Energy = -1171.401994 a.u.
Enthalpy = -1171.384045 a.u.
Free Energy = -1171.446660 a.u.

S	0.252269	1.208451	-0.811119
C	-1.200307	2.321554	-0.998691
H	-1.912214	1.846047	-1.666587
H	-0.806347	3.218305	-1.474310
C	0.397625	1.488763	0.994846
H	1.449310	1.644945	1.210507
H	0.058158	0.585574	1.488813
C	-0.492263	2.690065	1.307691
H	0.049727	3.615307	1.106950
H	-0.757318	2.678709	2.364327
C	-1.721114	2.596769	0.405451
H	-2.368070	1.782531	0.727920
H	-2.302145	3.518425	0.414253
C	-0.363136	-0.514739	-1.061109
C	0.802308	-1.710942	-0.744819
H	0.979791	-2.039013	-1.796225
O	0.349053	-2.639751	0.057284
C	2.117122	-1.043262	-0.306656
C	2.946872	-0.384857	-1.215098
C	2.519770	-1.121535	1.023878
C	4.132252	0.212317	-0.799527
H	2.663070	-0.341935	-2.260615
C	3.701712	-0.525172	1.448672
H	1.888349	-1.670129	1.709573
C	4.510406	0.150766	0.538812
H	4.764072	0.718474	-1.518514
H	3.997663	-0.591187	2.488337
H	5.433738	0.611214	0.865730
H	-0.485791	-0.491175	-2.143792
C	-1.690746	-0.742758	-0.422078
C	-2.851941	-0.625521	-1.189750
C	-1.815520	-1.066009	0.933590
C	-4.108819	-0.791687	-0.618893
H	-2.768092	-0.402687	-2.246121
C	-3.070410	-1.228702	1.505447
H	-0.926319	-1.229421	1.518617
C	-4.220975	-1.085610	0.735147
H	-4.995751	-0.693447	-1.230933
H	-3.150921	-1.479760	2.555126
H	-5.196654	-1.214625	1.185095

C-TS3-H

Number of imaginary frequencies: 1
Electronic Energy (SCF) = -1171.75256770 a.u
ZPV corrected Energy = -1171.417380 a.u.
Enthalpy = -1171.399677 a.u.
Free Energy = -1171.462392 a.u.

S	-0.762432	-1.776780	0.415852
C	-2.117410	-1.362898	1.620525
H	-1.808673	-0.534259	2.251290
H	-2.217607	-2.251930	2.242419
C	-1.943941	-2.023024	-0.956717
H	-1.621982	-2.924946	-1.466547
H	-1.826582	-1.184570	-1.633715
C	-3.342675	-2.092582	-0.353523
H	-3.542300	-3.102974	0.007646
H	-4.085224	-1.858137	-1.116300
C	-3.382759	-1.105806	0.813064
H	-3.386462	-0.082456	0.439014
H	-4.272325	-1.241737	1.428511
C	0.054159	-0.111062	0.236825
C	1.081262	-0.479773	-0.901585
H	0.876518	0.214465	-1.737746
O	0.886146	-1.785801	-1.222002
C	2.487121	-0.126283	-0.411166
C	2.900096	1.203818	-0.319100
C	3.369613	-1.130542	-0.026430
C	4.165941	1.524143	0.158068
H	2.225620	1.994278	-0.627715
C	4.639200	-0.815595	0.450218
H	3.041421	-2.156854	-0.119580
C	5.041177	0.512721	0.547109
H	4.473370	2.560369	0.222159
H	5.317360	-1.607546	0.743035
H	6.028978	0.759047	0.914494
H	0.539504	-0.015034	1.208200
C	-0.857979	1.055433	0.039601
C	-1.177000	1.870864	1.128515
C	-1.407557	1.366720	-1.208436
C	-2.043843	2.947085	0.987388
H	-0.737943	1.656230	2.094222
C	-2.276796	2.440026	-1.351382
H	-1.148848	0.778281	-2.076424
C	-2.603756	3.229674	-0.253478
H	-2.278626	3.565175	1.843804
H	-2.695362	2.663710	-2.323676
H	-3.280186	4.066251	-0.367686

C-TS2-H

Number of imaginary frequencies: 1
Electronic Energy (SCF) = -1171.73615635 a.u
ZPV corrected Energy = -1171.402144 a.u.
Enthalpy = -1171.384044 a.u.
Free Energy = -1171.447950 a.u.

S	1.863234	-1.507299	-0.521320
C	3.323828	-0.399173	-0.457480
H	3.189977	0.379695	-1.203458
H	4.171145	-1.022040	-0.738523
C	1.700596	-1.626165	1.300883
H	1.528417	-2.670802	1.541708
H	0.833287	-1.042714	1.588104
C	2.997878	-1.058529	1.875457
H	3.768542	-1.830479	1.881066
H	2.831849	-0.741799	2.904500
C	3.424218	0.102194	0.978558
H	2.760975	0.953914	1.120082
H	4.443135	0.424404	1.190077
C	0.448978	-0.491726	-1.130044
C	-0.828100	-1.528404	-1.163062
H	-0.466131	-2.411343	-0.567879
O	-1.123274	-1.800286	-2.421764
C	-1.985170	-0.960917	-0.337741
C	-2.270818	-1.444730	0.935207
C	-2.794636	0.043191	-0.867391
C	-3.328214	-0.925304	1.679290
H	-1.658408	-2.237514	1.349909
C	-3.844562	0.572334	-0.129917
H	-2.588241	0.402056	-1.866837
C	-4.113560	0.092711	1.151471
H	-3.539825	-1.317292	2.666283
H	-4.456010	1.361273	-0.549784
H	-4.934982	0.501947	1.725221

H	0.697209	-0.441177	-2.188376
C	0.405179	0.897548	-0.575532
C	0.878402	1.938567	-1.382040
C	-0.087528	1.221076	0.694023
C	0.875617	3.254466	-0.936024
H	1.248392	1.710182	-2.373157
C	-0.085708	2.533842	1.143479
H	-0.502598	0.455450	1.328811
C	0.398003	3.556564	0.333538
H	1.244237	4.040896	-1.581297
H	-0.475265	2.759580	2.127300
H	0.392332	4.579560	0.685445

D-TS1-H
Number of imaginary frequencies: 1
Electronic Energy (SCF) = -1171.73516829 a.u
ZPV corrected Energy = -1171.401228 a.u.
Enthalpy = -1171.383223 a.u.
Free Energy = -1171.446096 a.u.

S	0.246251	1.235953	-0.763664
C	-1.158405	2.403645	-0.963143
H	-2.069148	1.813838	-1.028874
H	-1.000360	2.945883	-1.891491
C	0.364509	1.481477	1.053160
H	1.312656	1.993109	1.201899
H	0.411802	0.510914	1.530313
C	-0.830534	2.338149	1.458610
H	-0.598251	2.887302	2.370334
H	-1.691015	1.699882	1.654595
C	-1.131544	3.273524	0.289208
H	-2.088722	3.780657	0.406250
H	-0.355175	4.035298	0.202459
C	-0.393696	-0.458506	-1.079652
C	0.770538	-1.685615	-0.830486
H	0.949322	-1.947093	-1.899812
O	0.299655	-2.653042	-0.089710
C	2.088785	-1.060300	-0.343911
C	2.936772	-0.365452	-1.206948
C	2.479347	-1.220501	0.983102
C	4.129274	0.185604	-0.749714
H	2.662545	-0.258634	-2.250401
C	3.667340	-0.669276	1.449919
H	1.834275	-1.798628	1.631044
C	4.495460	0.041846	0.585461
H	4.775747	0.720044	-1.434412
H	3.953466	-0.799411	2.486279
H	5.424156	0.466165	0.944484
H	-0.529844	-0.392607	-2.159121
C	-1.709592	-0.720482	-0.431419
C	-2.879136	-0.643354	-1.190838
C	-1.812723	-1.054816	0.922527
C	-4.124889	-0.872537	-0.615591
H	-2.810810	-0.405481	-2.245130
C	-3.055660	-1.276996	1.499675
H	-0.917150	-1.170039	1.509111
C	-4.215999	-1.184141	0.735520
H	-5.018567	-0.807335	-1.222213
H	-3.119269	-1.536118	2.548549
H	-5.182004	-1.362265	1.189553

D-TS3-H
Number of imaginary frequencies: 1
Electronic Energy (SCF) = -1171.75152046 a.u
ZPV corrected Energy = -1171.416550 a.u.
Enthalpy = -1171.398723 a.u.
Free Energy = -1171.462107 a.u.

S	-0.819645	-1.734753	0.332429
C	-2.134524	-1.376916	1.589328
H	-2.237279	-0.304143	1.730132
H	-1.822420	-1.835881	2.524712
C	-2.032835	-1.882471	-1.033182
H	-1.935399	-2.916870	-1.356834
H	-1.706064	-1.244461	-1.843743
C	-3.414425	-1.566578	-0.472395
H	-4.179830	-2.094334	-1.041097
H	-3.608875	-0.496970	-0.554911
C	-3.406758	-1.975440	1.000233
H	-4.285780	-1.612869	1.532687
H	-3.390378	-3.063838	1.088085
C	0.042323	-0.094168	0.223808
C	1.064665	-0.442233	-0.933281
H	0.880461	0.303231	-1.729682
O	0.834785	-1.721472	-1.322792
C	2.477516	-0.154301	-0.421019
C	2.935582	1.156639	-0.281274
C	3.321107	-1.201100	-0.063226
C	4.207789	1.416520	0.215504
H	2.291709	1.979924	-0.568813
C	4.596546	-0.946819	0.433375
H	2.958574	-2.211730	-0.193661
C	5.043776	0.362745	0.577334
H	4.550816	2.438633	0.315664
H	5.244286	-1.771237	0.704705
H	6.036532	0.561872	0.959673
H	0.528744	-0.046171	1.198539
C	-0.832108	1.106406	0.063607
C	-1.077144	1.931358	1.164222
C	-1.408545	1.445479	-1.164454
C	-1.888678	3.054092	1.051046
H	-0.621605	1.689535	2.115906
C	-2.225959	2.561749	-1.278053
H	-1.211124	0.844236	-2.039561
C	-2.471829	3.368409	-0.170707
H	-2.063333	3.681252	1.915196
H	-2.666403	2.807112	-2.235367
H	-3.105581	4.240312	-0.263356

D-TS2-H
Number of imaginary frequencies: 1
Electronic Energy (SCF) = -1171.73630263 a.u
ZPV corrected Energy = -1171.402081 a.u.
Enthalpy = -1171.384108 a.u.
Free Energy = -1171.447228 a.u.

S	1.990878	-1.352793	-0.501180
C	3.376292	-0.150910	-0.513196
H	2.949703	0.827100	-0.725517
H	4.054496	-0.437502	-1.312254
C	1.899128	-1.434853	1.330620
H	2.257839	-2.431043	1.580970
H	0.858586	-1.354203	1.627181
C	2.818362	-0.341243	1.865531
H	3.155735	-0.600622	2.868322
H	2.280187	0.603454	1.920732
C	3.984130	-0.221313	0.884939
H	4.584137	0.668738	1.070803
H	4.637690	-1.091246	0.964786
C	0.499421	-0.471674	-1.107758
C	-0.691707	-1.617352	-1.104775
H	-0.261105	-2.440900	-0.471988
O	-0.956815	-1.964021	-2.349512
C	-1.887597	-1.100393	-0.301000
C	-2.126357	-1.535309	0.998853

C	-2.776966	-0.193116	-0.875334
C	-3.216660	-1.061274	1.725228
H	-1.451053	-2.254636	1.448644
C	-3.860903	0.290882	-0.156097
H	-2.606715	0.124805	-1.895308
C	-4.083239	-0.138330	1.151823
H	-3.390226	-1.414556	2.734036
H	-4.535911	1.004888	-0.611205
H	-4.930950	0.235384	1.711399
H	0.726500	-0.415887	-2.170672
C	0.308701	0.911216	-0.574371
C	0.512636	1.986780	-1.444426
C	-0.104077	1.189351	0.732863
C	0.307725	3.297505	-1.028696
H	0.825814	1.788923	-2.461321
C	-0.300076	2.496595	1.152703
H	-0.301187	0.385548	1.422834
C	-0.097592	3.557500	0.274340
H	0.464524	4.111736	-1.723820
H	-0.624648	2.687097	2.167118
H	-0.260274	4.575400	0.602744

E-TS1-H
Number of imaginary frequencies: 1
Electronic Energy (SCF) = -1171.74160650 a.u
ZPV corrected Energy = -1171.407556 a.u.
Enthalpy = -1171.389433 a.u.
Free Energy = -1171.453551 a.u.

S	0.104459	0.990040	-0.809818
C	-1.089931	2.359303	-1.064789
H	-2.090342	1.960283	-0.920883
H	-0.971396	2.661211	-2.104325
C	1.354751	2.069069	0.009058
H	2.310910	1.884408	-0.469070
H	1.396607	1.739862	1.043048
C	0.847361	3.503161	-0.136850
H	1.180000	3.918800	-1.088847
H	1.262760	4.116480	0.661848
C	-0.678222	3.458323	-0.096574
H	-1.028554	3.223108	0.908859
H	-1.123490	4.406927	-0.394768
C	-0.598069	0.082038	0.622715
H	-0.734312	0.841362	1.386663
C	0.453023	-0.965901	1.343022
H	-0.080168	-1.934765	1.203982
O	0.616991	-0.588089	2.597788
C	1.755762	-1.121375	0.527128
C	1.793583	-1.777788	-0.704127
C	2.950133	-0.639895	1.055968
C	2.986426	-1.917802	-1.405534
H	0.885412	-2.199305	-1.116691
C	4.147581	-0.775118	0.361690
H	2.908223	-0.167757	2.028137
C	4.169660	-1.409159	-0.877145
H	2.994943	-2.430964	-2.358924
H	5.066172	-0.391621	0.788404
H	5.100360	-1.520063	-1.418396
C	-1.919486	-0.498898	0.229478
C	-3.056756	-0.149738	0.960580
C	-2.052267	-1.415610	-0.817794
C	-4.294247	-0.709884	0.662626
H	-2.966130	0.560537	1.771843
C	-3.289770	-1.965017	-1.124764
H	-1.187172	-1.701650	-1.399347
C	-4.415463	-1.617157	-0.383265
H	-5.162746	-0.433113	1.245688
H	-3.374859	-2.670324	-1.940916
H	-5.377916	-2.050679	-0.620061

E-TS3-H
Number of imaginary frequencies: 1
Electronic Energy (SCF) = -1171.75247051 a.u
ZPV corrected Energy = -1171.417742 a.u.
Enthalpy = -1171.399978 a.u.
Free Energy = -1171.462799 a.u.

S	1.930678	-0.598990	-0.722025
C	2.976222	0.935083	-0.669674
H	2.334820	1.804181	-0.556203
H	3.457889	0.985643	-1.646005
C	3.188198	-1.567655	0.204232
H	3.319932	-2.496759	-0.340315
H	2.733939	-1.794080	1.163941
C	4.446650	-0.715996	0.328609
H	5.070937	-0.839788	-0.557929
H	5.027384	-1.038704	1.192830
C	3.997244	0.740269	0.440537
H	3.534825	0.920496	1.412998
H	4.831233	1.434471	0.335385
C	0.677277	-0.134823	0.549200
H	1.233287	0.047334	1.465353
C	-0.091127	-1.536225	0.638470
H	0.011417	-1.830591	1.703176
O	0.540949	-2.375730	-0.218616
C	-1.591326	-1.378615	0.405545
C	-2.404228	-0.753902	1.351399
C	-2.171878	-1.851822	-0.765718
C	-3.763930	-0.584959	1.122532
H	-1.966084	-0.388674	2.272937
C	-3.534412	-1.690175	-1.000292
H	-1.531607	-2.345791	-1.483475
C	-4.334854	-1.050937	-0.059704
H	-4.380015	-0.091998	1.863937
H	-3.972025	-2.062539	-1.918244
H	-5.394160	-0.922081	-0.240547
C	-0.062504	1.105843	0.166479
C	-0.181819	2.145348	1.088756
C	-0.679230	1.239444	-1.080360
C	-0.914526	3.287047	0.783594
H	0.297017	2.052754	2.055384
C	-1.402887	2.381946	-1.390656
H	-0.602435	0.442247	-1.806330
C	-1.527486	3.408053	-0.457967
H	-1.002820	4.081084	1.513378
H	-1.877050	2.469481	-2.359186
H	-2.097230	4.295447	-0.699653

E-TS2-H
Number of imaginary frequencies: 1
Electronic Energy (SCF) = -1171.73767282 a.u
ZPV corrected Energy = -1171.404078 a.u.
Enthalpy = -1171.385815 a.u.
Free Energy = -1171.450557 a.u.

S	-0.587509	1.611302	-0.890685
C	-1.486768	2.604490	0.361016
H	-2.222831	1.957797	0.830566
H	-2.000800	3.384512	-0.198602
C	1.030914	2.425111	-0.552976
H	1.460027	2.715921	-1.506925
H	1.660368	1.671524	-0.090465
C	0.735680	3.594311	0.385819
H	0.451479	4.474002	-0.192894
H	1.633266	3.839254	0.952380

C	-0.421038	3.170710	1.287600
H	-0.094292	2.406945	1.994405
H	-0.824292	4.007514	1.856822
C	-0.392369	-0.044607	-0.130815
H	0.064484	0.135379	0.838962
C	0.615043	-0.906148	-1.120861
H	0.804447	-0.198494	-1.975544
O	0.113313	-2.070802	-1.475473
C	1.959473	-1.018835	-0.389351
C	3.120144	-0.457218	-0.913776
C	2.053531	-1.753763	0.794913
C	4.348222	-0.614281	-0.272599
H	3.063329	0.106161	-1.837992
C	3.270877	-1.908070	1.443526
H	1.161668	-2.216520	1.198379
C	4.426890	-1.336267	0.911599
H	5.240797	-0.175279	-0.700623
H	3.324307	-2.476869	2.363478
H	5.377361	-1.460668	1.413968
C	-1.750537	-0.636189	0.049008
C	-2.232094	-0.876268	1.335466
C	-2.554754	-0.962276	-1.046076
C	-3.492349	-1.431938	1.531521
H	-1.611410	-0.631062	2.188116
C	-3.817546	-1.505822	-0.851583
H	-2.183054	-0.802112	-2.046610
C	-4.289619	-1.744359	0.436632
H	-3.849490	-1.616510	2.536123
H	-4.431838	-1.754149	-1.707147
H	-5.272074	-2.173281	0.583962

F-TS1-H
Number of imaginary frequencies: 1
Electronic Energy (SCF) = -1171.74083013 a.u
ZPV corrected Energy = -1171.406927 a.u.
Enthalpy = -1171.388739 a.u.
Free Energy = -1171.452880 a.u.

S	0.121299	1.032571	-0.696853
C	-1.124183	2.363199	-0.969480
H	-1.934373	2.185797	-0.266566
H	-1.496833	2.262006	-1.984751
C	1.289020	2.130133	0.202580
H	2.124215	2.291352	-0.475174
H	1.633180	1.592095	1.078771
C	0.520519	3.411094	0.503921
H	1.219898	4.228754	0.674361
H	-0.074079	3.281626	1.408971
C	-0.391264	3.671407	-0.691794
H	-1.113568	4.463408	-0.496922
H	0.201114	3.962133	-1.560781
C	-0.579681	0.023538	0.664283
H	-0.698819	0.722557	1.487323
C	0.459689	-1.101743	1.281875
H	-0.075649	-2.047825	1.036236
O	0.610226	-0.857042	2.570037
C	-1.908567	-0.516033	0.237727
C	-3.023970	-0.290036	1.046152
C	-2.064280	-1.287094	-0.918384
C	-4.262886	-0.828834	0.715843
H	-2.914933	0.305433	1.942965
C	-3.303790	-1.813321	-1.256128
H	-1.216221	-1.478686	-1.561666
C	-4.407867	-1.589385	-0.438218
H	-5.114286	-0.649016	1.358971
H	-3.407050	-2.403820	-2.156917
H	-5.371819	-2.004806	-0.700002
C	1.766533	-1.174144	0.463356
C	1.810191	-1.725139	-0.818276
C	2.954564	-0.719794	1.028814
C	3.002832	-1.789805	-1.530305
H	0.903721	-2.118522	-1.261771
C	4.151796	-0.777737	0.322923
H	2.910273	-0.332546	2.037922
C	4.179699	-1.307773	-0.963587
H	3.016611	-2.221259	-2.523302
H	5.065631	-0.415924	0.777814
H	5.110281	-1.358751	-1.513923

F-TS3-H
Number of imaginary frequencies: 1
Electronic Energy (SCF) = -1171.75151688 a.u
ZPV corrected Energy = -1171.416948 a.u.
Enthalpy = -1171.399035 a.u.
Free Energy = -1171.462605 a.u.

S	1.947298	-0.553485	-0.633942
C	2.979190	0.987545	-0.491101
H	2.640033	1.572766	0.360633
H	2.835530	1.566496	-1.399732
C	3.183914	-1.564197	0.273136
H	3.519665	-2.301496	-0.453065
H	2.654318	-2.080758	1.064748
C	4.302847	-0.638830	0.735828
H	5.234624	-1.195740	0.832753
H	4.058962	-0.222561	1.714621
C	4.403789	0.488184	-0.290317
H	5.053891	1.296331	0.044504
H	4.807915	0.104712	-1.229911
C	0.638543	-0.157574	0.594282
H	1.150008	-0.007476	1.542505
C	-0.129554	-1.562115	0.587432
H	-0.037039	-1.918637	1.634556
O	0.508776	-2.351796	-0.310669
C	-0.081992	1.097737	0.216637
C	-0.243062	2.110719	1.161492
C	-0.639935	1.267232	-1.053226
C	-0.958956	3.262530	0.854509
H	0.188324	1.989412	2.147084
C	-1.347006	2.419893	-1.364670
H	-0.531578	0.489424	-1.796153
C	-1.512943	3.419976	-0.410455
H	-1.080194	4.036025	1.601415
H	-1.775718	2.535518	-2.351284
H	-2.069533	4.315368	-0.653343
C	-1.627377	-1.386488	0.352989
C	-2.441747	-0.796948	1.320058
C	-2.204426	-1.807210	-0.839712
C	-3.799581	-0.612353	1.092437
H	-2.006472	-0.472585	2.258131
C	-3.565259	-1.629700	-1.073283
H	-1.562816	-2.273196	-1.574724
C	-4.367240	-1.026613	-0.110487
H	-4.416777	-0.147278	1.850754
H	-4.000199	-1.961392	-2.007954
H	-5.425139	-0.885523	-0.290415

F-TS2-H
Number of imaginary frequencies: 1
Electronic Energy (SCF) = -1171.73729978 a.u
ZPV corrected Energy = -1171.403768 a.u.
Enthalpy = -1171.385442 a.u.
Free Energy = -1171.450594 a.u.

S	-0.664750	1.617476	-0.905015

C	-1.653353	2.549477	0.343051
H	-1.940309	1.828447	1.103736
H	-2.547246	2.913891	-0.154992
C	0.883630	2.521077	-0.531887
H	0.973326	3.282560	-1.304833
H	1.708694	1.822084	-0.619515
C	0.686483	3.122983	0.851489
H	1.419921	3.910219	1.022295
H	0.829566	2.357661	1.615501
C	-0.744395	3.654298	0.882928
H	-1.059525	3.929737	1.888584
H	-0.822976	4.539601	0.251039
C	-0.375179	-0.024837	-0.145686
H	0.082806	0.186700	0.817124
C	0.662394	-0.835824	-1.143591
H	0.843147	-0.102783	-1.978205
O	0.200118	-2.005732	-1.535683
C	-1.696428	-0.689145	0.054533
C	-2.129975	-0.992124	1.344623
C	-2.507911	-1.032990	-1.029771
C	-3.350285	-1.626567	1.554631
H	-1.502743	-0.736424	2.189174
C	-3.731472	-1.655078	-0.820998
H	-2.171462	-0.825276	-2.034182
C	-4.155794	-1.955694	0.470857
H	-3.669918	-1.859411	2.561830
H	-4.351781	-1.915939	-1.668443
H	-5.107378	-2.445821	0.629510
C	2.002123	-0.932058	-0.400797
C	3.166010	-0.389693	-0.939223
C	2.094104	-1.640463	0.799736
C	4.394318	-0.540415	-0.297089
H	3.111844	0.151007	-1.877277
C	3.312159	-1.789768	1.448389
H	1.201237	-2.088400	1.217785
C	4.470881	-1.237886	0.901797
H	5.288615	-0.116115	-0.736165
H	3.363780	-2.339228	2.380104
H	5.421595	-1.358026	1.404687

G-TS1-H
Number of imaginary frequencies: 1
Electronic Energy (SCF) = -1171.73768915 a.u
ZPV corrected Energy = -1171.403733 a.u.
Enthalpy = -1171.385635 a.u.
Free Energy = -1171.449895 a.u.

S	0.484158	-1.745573	-0.297083
C	1.888095	-1.786397	-1.479136
H	1.744313	-0.990916	-2.204735
H	1.809316	-2.748513	-1.982602
C	1.532214	-2.064973	1.173783
H	1.041247	-2.835764	1.759801
H	1.567393	-1.144476	1.746794
C	2.903328	-2.473103	0.635215
H	2.909978	-3.541103	0.414327
H	3.661666	-2.282266	1.393493
C	3.155803	-1.673461	-0.641772
H	3.351220	-0.629358	-0.403781
H	4.006634	-2.061628	-1.200745
C	-0.070077	0.003257	-0.148138
C	-1.132127	0.028429	1.138732
O	-0.889069	0.996862	1.990869
H	-0.631889	0.108720	-1.074984
C	1.045428	0.993720	-0.144516
C	1.403530	1.610054	-1.346769
C	1.755236	1.325068	1.014523
C	2.464474	2.505847	-1.405020
H	0.843300	1.382307	-2.244914
C	2.815964	2.220044	0.956503
H	1.436369	0.913611	1.957371
C	3.180065	2.807055	-0.251577
H	2.727783	2.968832	-2.346890
H	3.355325	2.467853	1.861496
H	4.007341	3.503594	-0.290765
H	-1.043576	-1.005231	1.566665
C	-2.519365	0.058714	0.474451
C	-3.155021	-1.104876	0.045633
C	-3.159230	1.280898	0.281019
C	-4.400132	-1.050964	-0.575207
H	-2.679676	-2.065255	0.210215
C	-4.400380	1.342423	-0.342165
H	-2.668124	2.174239	0.643247
C	-5.025526	0.175505	-0.775935
H	-4.885342	-1.964910	-0.894493
H	-4.885808	2.299798	-0.486199
H	-5.995119	0.221306	-1.254672

G-TS3-H
Number of imaginary frequencies: 1
Electronic Energy (SCF) = -1171.74902741 a.u
ZPV corrected Energy = -1171.413648 a.u.
Enthalpy = -1171.396095 a.u.
Free Energy = -1171.457722 a.u.

S	1.858987	-1.394300	-0.696935
C	3.224736	-0.156553	-0.436421
H	3.067095	0.707076	-1.076241
H	4.128332	-0.670537	-0.762453
C	1.695470	-1.717487	1.094637
H	1.589793	-2.793004	1.191062
H	0.774425	-1.251499	1.423624
C	2.925166	-1.131372	1.779184
H	3.758168	-1.833297	1.709759
H	2.716191	-0.965880	2.836233
C	3.275696	0.166576	1.051189
H	2.545294	0.938352	1.290037
H	4.260756	0.536786	1.336198
C	0.487118	-0.273921	-1.275894
C	-0.680662	-1.354502	-1.317317
O	-0.133662	-2.531601	-0.916750
H	0.820610	-0.078420	-2.295289
C	0.365059	1.046824	-0.583961
C	0.772076	2.195732	-1.268136
C	-0.144783	1.196928	0.710580
C	0.684776	3.453289	-0.682822
H	1.159141	2.099071	-2.274572
C	-0.228691	2.450179	1.299734
H	-0.500875	0.339715	1.257851
C	0.186795	3.583655	0.607787
H	1.004002	4.327263	-1.235038
H	-0.628909	2.542283	2.300659
H	0.114888	4.559855	1.068658
H	-0.995316	-1.359894	-2.381544
C	-1.926899	-0.926074	-0.545807
C	-2.722726	0.128992	-0.992291
C	-2.297816	-1.597568	0.613911
C	-3.847197	0.524991	-0.279473
H	-2.452341	0.653095	-1.901556
C	-3.423658	-1.206947	1.333572
H	-1.684972	-2.428463	0.934928
C	-4.199131	-0.139525	0.893252
H	-4.449811	1.351033	-0.635376
H	-3.697955	-1.737504	2.236970
H	-5.074988	0.166275	1.450702

G-TS2-H
Number of imaginary frequencies: 1
Electronic Energy (SCF) = -1171.73863284 a.u
ZPV corrected Energy = -1171.404279 a.u.
Enthalpy = -1171.386311 a.u.
Free Energy = -1171.449328 a.u.

S	0.240859	1.079202	-0.907644
C	-1.216553	2.020235	-1.516015
H	-1.825896	1.352691	-2.118568
H	-0.806765	2.801683	-2.153620
C	0.160817	1.792583	0.780192
H	1.173271	2.061856	1.061576
H	-0.205474	1.019350	1.446655
C	-0.800606	2.976169	0.689827
H	-0.271399	3.856207	0.322098
H	-1.186601	3.206067	1.682345
C	-1.909877	2.586689	-0.283624
H	-2.555891	1.830537	0.160391
H	-2.526057	3.441033	-0.561859
C	-0.330852	-0.695409	-0.843043
C	0.845526	-1.748922	-0.427431
O	1.008409	-2.601180	-1.427645
H	-0.431775	-0.870744	-1.912782
C	-1.650741	-0.855843	-0.165060
C	-2.820089	-0.923190	-0.927548
C	-1.753903	-0.964436	1.225608
C	-4.060812	-1.065479	-0.319275
H	-2.751950	-0.868767	-2.006171
C	-2.994065	-1.100681	1.835761
H	-0.859559	-0.958461	1.832298
C	-4.152145	-1.145203	1.066203
H	-4.954590	-1.117779	-0.926796
H	-3.055405	-1.181180	2.912981
H	-5.117294	-1.255268	1.542438
H	0.439607	-2.195662	0.507411
C	2.118393	-1.004240	0.014666
C	2.316748	-0.632038	1.342703
C	3.119370	-0.718599	-0.912585
C	3.475669	0.030241	1.737415
H	1.555920	-0.869374	2.077747
C	4.274739	-0.048618	-0.529769
H	2.974205	-1.045180	-1.933801
C	4.456430	0.332159	0.798482
H	3.616048	0.304532	2.775513
H	5.040013	0.172536	-1.263376
H	5.360267	0.845627	1.099947

H-TS1-H
Number of imaginary frequencies: 1
Electronic Energy (SCF) = -1171.73736760 a.u
ZPV corrected Energy = -1171.403349 a.u.
Enthalpy = -1171.385289 a.u.
Free Energy = -1171.448957 a.u.

S	0.480142	-1.726576	-0.200233
C	1.818788	-1.877786	-1.448095
H	2.066482	-0.872096	-1.778783
H	1.425938	-2.453729	-2.281500
C	1.597644	-1.981244	1.233446
H	1.338203	-2.964265	1.620441
H	1.366785	-1.234076	1.985508
C	3.020914	-1.934066	0.686946
H	3.688329	-2.483560	1.349755
H	3.363789	-0.901946	0.636263
C	2.980462	-2.542354	-0.713778
H	3.906693	-2.370883	-1.261071
H	2.818126	-3.619545	-0.654411
C	-0.056119	0.028584	-0.138655
C	-1.120473	0.116077	1.148432
O	-0.886007	1.134960	1.940189
H	-0.618547	0.098191	-1.068690
C	1.057789	1.018406	-0.169498
C	1.382806	1.633089	-1.381447
C	1.788891	1.360877	0.972222
C	2.424949	2.549969	-1.463979
H	0.808700	1.390947	-2.266981
C	2.831801	2.274156	0.889975
H	1.507922	0.938956	1.922284
C	3.156857	2.868560	-0.326245
H	2.660750	3.013921	-2.412638
H	3.387825	2.531433	1.782119
H	3.968799	3.581699	-0.383369
H	-1.017772	-0.890139	1.635237
C	-2.507010	0.088525	0.483802
C	-3.146811	1.288599	0.179980
C	-3.143109	-1.108743	0.162051
C	-4.387733	1.293400	-0.446148
H	-2.656868	2.212248	0.458255
C	-4.388241	-1.111219	-0.461476
H	-2.667519	-2.049899	0.413283
C	-5.013457	0.091922	-0.772079
H	-4.873058	2.233762	-0.676606
H	-4.873435	-2.050372	-0.696588
H	-5.983040	0.094384	-1.253016

H-TS3-H
Number of imaginary frequencies: 1
Electronic Energy (SCF) = -1171.74846952 a.u
ZPV corrected Energy = -1171.413602 a.u.
Enthalpy = -1171.395903 a.u.
Free Energy = -1171.458217 a.u.

S	1.970439	-1.192836	-0.692650
C	3.232790	0.151170	-0.514247
H	2.744213	1.120018	-0.446475
H	3.864585	0.125237	-1.399385
C	1.911139	-1.502339	1.114049
H	2.221916	-2.541010	1.205128
H	0.881124	-1.432358	1.436418
C	2.873583	-0.533699	1.793121
H	3.282994	-0.983972	2.697136
H	2.341399	0.373629	2.078931
C	3.964400	-0.187767	0.778801
H	4.578719	0.650355	1.106964
H	4.624755	-1.044640	0.628462
C	0.490745	-0.229647	-1.256797
C	-0.566280	-1.427521	-1.268305
O	0.079103	-2.529494	-0.809995
H	0.782214	0.003166	-2.281689
C	0.225495	1.064371	-0.555947
C	0.372597	2.254527	-1.272184
C	-0.183989	1.141855	0.779014
C	0.118850	3.486890	-0.680572
H	0.686540	2.210832	-2.307312
C	-0.427458	2.370253	1.375582
H	-0.336713	0.242017	1.352044
C	-0.278967	3.548334	0.649005
H	0.234779	4.394859	-1.257505
H	-0.745878	2.407369	2.408903
H	-0.476478	4.504144	1.115851
H	-0.862862	-1.505905	-2.334841
C	-1.854162	-1.073461	-0.526906
C	-2.180589	-1.719982	0.659825
C	-2.728894	-0.107916	-1.025751

C	-3.341905	-1.391205	1.353819
H	-1.505617	-2.482686	1.023002
C	-3.888492	0.226741	-0.338443
H	-2.493205	0.395483	-1.956095
C	-4.197074	-0.411554	0.860915
H	-3.580769	-1.901153	2.278896
H	-4.552452	0.984477	-0.734920
H	-5.099845	-0.152849	1.398972

H-TS2-H
Number of imaginary frequencies: 1
Electronic Energy (SCF) = -1171.73845134 a.u
ZPV corrected Energy = -1171.403920 a.u.
Enthalpy = -1171.385986 a.u.
Free Energy = -1171.448869 a.u.

S	0.254897	1.160057	-0.730765
C	-1.102456	2.168396	-1.449870
H	-1.924487	1.494013	-1.674905
H	-0.726779	2.606160	-2.370623
C	-0.067672	1.713444	0.988089
H	0.768712	2.364726	1.232808
H	-0.042019	0.849121	1.640768
C	-1.398543	2.456730	0.963715
H	-1.451463	3.144953	1.806661
H	-2.219960	1.746672	1.051626
C	-1.473809	3.186065	-0.375535
H	-2.469769	3.581695	-0.571833
H	-0.770863	4.020080	-0.391083
C	-0.315782	-0.603429	-0.899781
C	0.882232	-1.687945	-0.642598
O	1.077350	-2.364034	-1.763561
H	-0.430944	-0.642038	-1.981927
C	-1.625032	-0.860451	-0.229005
C	-2.804837	-0.859062	-0.978846
C	-1.707067	-1.134139	1.140170
C	-4.034184	-1.100479	-0.377235
H	-2.754327	-0.679102	-2.044833
C	-2.934688	-1.370043	1.743850
H	-0.806463	-1.172104	1.735392
C	-4.103413	-1.349101	0.988664
H	-4.935040	-1.098912	-0.976498
H	-2.978517	-1.578401	2.804658
H	-5.058721	-1.537595	1.460147
H	0.476005	-2.287059	0.203032
C	2.132758	-0.992615	-0.069488
C	3.176014	-0.634293	-0.920653
C	2.266295	-0.728646	1.292689
C	4.310119	0.001507	-0.430312
H	3.075452	-0.877037	-1.970048
C	3.401482	-0.098067	1.793878
H	1.477449	-1.030192	1.972066
C	4.425728	0.277055	0.930560
H	5.109533	0.279271	-1.106078
H	3.490469	0.092328	2.856156
H	5.311704	0.765229	1.315493

A-TS4-H
Number of imaginary frequencies: 1
Electronic Energy (SCF) = -1171.73715908 a.u
ZPV corrected Energy = -1171.403965 a.u.
Enthalpy = -1171.385007 a.u.
Free Energy = -1171.452045 a.u.

S	1.445870	-1.056246	-0.819988
C	2.959386	-0.043792	-1.034769
H	2.695984	0.999633	-0.878440
H	3.290737	-0.172339	-2.063499
C	2.300613	-2.376004	0.130424
H	1.991331	-3.340099	-0.264502
H	1.973820	-2.296602	1.165467
C	3.796540	-2.104661	-0.017328
H	4.159293	-2.530812	-0.954093
H	4.344890	-2.573079	0.799989
C	3.973518	-0.588090	-0.034901
H	3.774643	-0.176604	0.955928
H	4.983771	-0.296866	-0.322543
C	0.459395	0.034366	0.934074
H	1.345780	-0.041989	1.548068
C	-0.652530	-0.809359	1.495837
H	-0.266966	-1.825531	1.705216
O	-0.775244	-0.006347	2.587788
C	0.244718	1.370484	0.343506
C	0.989807	2.448531	0.831011
C	-0.627017	1.588291	-0.728673
C	0.844638	3.718715	0.288873
H	1.676262	2.285378	1.651321
C	-0.767678	2.856477	-1.274778
H	-1.185432	0.764672	-1.144948
C	-0.037975	3.926593	-0.765477
H	1.420837	4.543391	0.686944
H	-1.446274	3.008832	-2.103543
H	-0.151486	4.913962	-1.193096
C	-1.893818	-0.984235	0.637059
C	-2.991491	-0.148089	0.816741
C	-1.956993	-1.977814	-0.339267
C	-4.121365	-0.280258	0.016287
H	-2.933863	0.608789	1.586063
C	-3.079997	-2.109502	-1.147913
H	-1.123168	-2.657805	-0.467815
C	-4.166270	-1.255475	-0.974984
H	-4.966830	0.379862	0.163498
H	-3.112619	-2.881176	-1.906484
H	-5.043571	-1.357966	-1.600372

B-TS4-H
Number of imaginary frequencies: 1
Electronic Energy (SCF) = -1171.73690633 a.u
ZPV corrected Energy = -1171.403748 a.u.
Enthalpy = -1171.384802 a.u.
Free Energy = -1171.451959 a.u.

S	1.425721	-1.124513	-0.787550
C	2.898133	-0.076531	-1.110422
H	2.788420	0.833660	-0.523671
H	2.904006	0.185980	-2.164895
C	2.355656	-2.364211	0.195070
H	2.492071	-3.243452	-0.431778
H	1.752880	-2.635560	1.058152
C	3.683714	-1.703906	0.549524
H	4.421558	-2.458481	0.821977
H	3.549891	-1.040231	1.405342
C	4.109646	-0.898763	-0.675669
H	4.951818	-0.240593	-0.461816
H	4.406161	-1.575035	-1.479047
C	0.441521	-0.019538	0.956337
H	1.315171	-0.121281	1.584799
C	-0.706246	-0.829308	1.498089
H	-0.357179	-1.857364	1.712550
O	-0.820614	-0.024276	2.589106
C	-1.938701	-0.961204	0.618964
C	-2.016102	-1.943602	-0.367647
C	-3.014086	-0.094932	0.790278
C	-3.130192	-2.034772	-1.193989
H	-1.199816	-2.645225	-0.491818

C	-4.135259	-0.186545	-0.027944
H	-2.945675	0.652804	1.567603
C	-4.193666	-1.150868	-1.029041
H	-3.173771	-2.797953	-1.960565
H	-4.963190	0.496740	0.113171
H	-5.063991	-1.221707	-1.668420
C	0.270120	1.325611	0.370839
C	1.018534	2.386591	0.888906
C	-0.568837	1.567576	-0.721954
C	0.908264	3.664226	0.355636
H	1.677851	2.205178	1.727408
C	-0.674453	2.842784	-1.259351
H	-1.130315	0.757050	-1.159652
C	0.058279	3.896164	-0.720041
H	1.486043	4.475733	0.777747
H	-1.328241	3.014178	-2.104169
H	-0.028274	4.889335	-1.140470

C-TS4-H
Number of imaginary frequencies: 1
Electronic Energy (SCF) = -1171.73760314 a.u
ZPV corrected Energy = -1171.403899 a.u.
Enthalpy = -1171.385193 a.u.
Free Energy = -1171.450830 a.u.

S	-0.869511	-1.834322	0.893002
C	-2.693539	-1.671417	0.764316
H	-3.010421	-0.841936	1.391862
H	-3.120468	-2.592899	1.155472
C	-0.621218	-2.039313	-0.910188
H	0.095547	-2.841382	-1.060958
H	-0.205574	-1.114035	-1.298981
C	-2.002370	-2.331282	-1.492265
H	-2.238870	-3.389529	-1.370970
H	-2.011323	-2.103777	-2.558254
C	-3.002879	-1.477656	-0.717398
H	-2.883827	-0.428394	-0.984391
H	-4.033092	-1.765665	-0.926234
C	-0.251527	0.252888	1.460991
C	1.222712	0.275555	1.810762
O	1.197278	1.509961	2.365654
H	-0.829664	0.184537	2.373880
C	-0.884258	1.123081	0.454802
C	-2.113488	1.714787	0.771879
C	-0.350349	1.343656	-0.820636
C	-2.790570	2.498799	-0.150711
H	-2.530686	1.562166	1.758399
C	-1.027484	2.128070	-1.743631
H	0.596744	0.907745	-1.091291
C	-2.250090	2.705292	-1.415637
H	-3.736718	2.950013	0.117031
H	-0.598434	2.288001	-2.723893
H	-2.775159	3.314860	-2.139003
H	1.397001	-0.554494	2.527505
C	2.222768	0.061540	0.679654
C	2.524589	-1.217962	0.214631
C	2.879020	1.150703	0.113396
C	3.426331	-1.404316	-0.826868
H	2.062156	-2.080399	0.679932
C	3.788774	0.971184	-0.923483
H	2.652307	2.134726	0.499273
C	4.057934	-0.306361	-1.405090
H	3.645138	-2.403996	-1.180150
H	4.288448	1.828234	-1.357238
H	4.764703	-0.447800	-2.212395

D-TS4-H
Number of imaginary frequencies: 1
Electronic Energy (SCF) = -1171.73770066 a.u
ZPV corrected Energy = -1171.404165 a.u.
Enthalpy = -1171.385418 a.u.
Free Energy = -1171.451473 a.u.

S	-0.960629	-1.853507	0.639338
C	-2.778543	-1.686535	0.796583
H	-2.992749	-0.679830	1.150039
H	-3.121812	-2.406366	1.535122
C	-0.965797	-1.718367	-1.189408
H	-0.727495	-2.708911	-1.572581
H	-0.182074	-1.029234	-1.488308
C	-2.369086	-1.267154	-1.582897
H	-2.577852	-1.549678	-2.614613
H	-2.439833	-0.182620	-1.508086
C	-3.339825	-1.922551	-0.603165
H	-4.342960	-1.502944	-0.679311
H	-3.406018	-2.993751	-0.799824
C	-0.221602	0.117741	1.446396
C	1.240733	-0.010280	1.816250
O	1.293475	1.152703	2.507698
H	-0.827710	0.000941	2.335932
C	-0.761505	1.123829	0.516036
C	-1.971773	1.748109	0.844429
C	-0.159440	1.443915	-0.706978
C	-2.565727	2.659068	-0.017582
H	-2.438858	1.522178	1.793820
C	-0.752283	2.355759	-1.567735
H	0.771816	0.980736	-0.986452
C	-1.958014	2.963729	-1.230528
H	-3.498363	3.132892	0.258373
H	-0.272011	2.590660	-2.508341
H	-2.417239	3.672499	-1.906728
H	1.341052	-0.925869	2.436319
C	2.244590	-0.167302	0.677859
C	2.416277	-1.386248	0.021449
C	3.030506	0.914937	0.293400
C	3.320548	-1.509797	-1.027203
H	1.848518	-2.253019	0.337927
C	3.944235	0.795776	-0.748826
H	2.896690	1.848358	0.821883
C	4.085466	-0.414551	-1.420320
H	3.436974	-2.461064	-1.530756
H	4.545692	1.648083	-1.038986
H	4.793927	-0.509051	-2.232981

E-TS4-H
Number of imaginary frequencies: 1
Electronic Energy (SCF) = -1171.74158091 a.u
ZPV corrected Energy = -1171.408375 a.u.
Enthalpy = -1171.389453 a.u.
Free Energy = -1171.456192 a.u.

S	-0.023201	1.153942	0.934859
C	1.322795	2.347850	0.591634
H	2.247946	1.793714	0.456766
H	1.417091	2.981965	1.471758
C	-1.255023	2.041351	-0.101461
H	-2.174478	2.134605	0.468752
H	-1.447572	1.424899	-0.975807
C	-0.618662	3.380447	-0.468404
H	-0.787862	4.102579	0.331983
H	-1.074730	3.773030	-1.377252
C	0.879535	3.136105	-0.634249
H	1.068100	2.552784	-1.536992
H	1.441172	4.067063	-0.711921
C	0.434737	-0.616189	-0.382674

C	-0.502647	-1.738197	-0.023616
O	-0.138507	-2.520445	-1.072727
H	-0.256965	-2.144242	0.974927
C	-1.961968	-1.302550	0.022358
C	-2.581964	-0.976895	1.227472
C	-2.693380	-1.196192	-1.158684
C	-3.899387	-0.529373	1.252726
H	-2.030144	-1.077649	2.154480
C	-4.009521	-0.749640	-1.140049
H	-2.212722	-1.477886	-2.085815
C	-4.615919	-0.409013	0.066513
H	-4.367755	-0.281114	2.196648
H	-4.566425	-0.670312	-2.065197
H	-5.641691	-0.064454	0.082690
H	0.150129	-0.129362	-1.306281
C	1.886336	-0.772936	-0.189189
C	2.766340	-0.258771	-1.145562
C	2.414127	-1.374594	0.957557
C	4.140960	-0.355642	-0.971261
H	2.363982	0.210875	-2.033539
C	3.786631	-1.466451	1.134858
H	1.747189	-1.768537	1.711620
C	4.654892	-0.958756	0.171192
H	4.809111	0.039167	-1.725080
H	4.182635	-1.935760	2.025653
H	5.724948	-1.034207	0.311712

F-TS4-H
Number of imaginary frequencies: 1
Electronic Energy (SCF) = -1171.74125704 a.u
ZPV corrected Energy = -1171.408219 a.u.
Enthalpy = -1171.389226 a.u.
Free Energy = -1171.456380 a.u.

S	-0.005488	1.177814	0.910142
C	1.335923	2.374342	0.537710
H	2.055456	1.874400	-0.107130
H	1.826652	2.633277	1.472252
C	-1.247623	2.044984	-0.124923
H	-1.910971	2.579241	0.552638
H	-1.822520	1.298481	-0.664013
C	-0.455905	2.991615	-1.017857
H	-1.106148	3.772476	-1.412384
H	-0.039951	2.441105	-1.863594
C	0.670307	3.560095	-0.158504
H	1.404873	4.106667	-0.749970
H	0.259192	4.245208	0.584590
C	0.427802	-0.626700	-0.372543
C	-0.503976	-1.743055	0.022473
O	-0.141714	-2.549309	-1.008550
H	-0.250315	-2.121763	1.029588
C	-1.963548	-1.311198	0.062776
C	-2.571005	-0.923763	1.255965
C	-2.703323	-1.256557	-1.116412
C	-3.882952	-0.460649	1.268899
H	-2.012463	-0.984168	2.182391
C	-4.015523	-0.797049	-1.109323
H	-2.230851	-1.584387	-2.032441
C	-4.607754	-0.390082	0.083445
H	-4.341054	-0.161039	2.202873
H	-4.579228	-0.757215	-2.032877
H	-5.629302	-0.032953	0.090536
H	0.134745	-0.161978	-1.305301
C	1.881212	-0.782406	-0.187655
C	2.755518	-0.324627	-1.177390
C	2.416483	-1.335633	0.979919
C	4.131343	-0.430878	-1.015211
H	2.347999	0.104443	-2.083522
C	3.789668	-1.436076	1.145059
H	1.754586	-1.686503	1.759382
C	4.652115	-0.985480	0.148057
H	4.794399	-0.081085	-1.795288
H	4.191033	-1.867926	2.052258
H	5.722828	-1.068134	0.279214

G-TS4-H
Number of imaginary frequencies: 1
Electronic Energy (SCF) = -1171.74076784 a.u
ZPV corrected Energy = -1171.407276 a.u.
Enthalpy = -1171.388482 a.u.
Free Energy = -1171.454540 a.u.

S	0.145585	1.363275	-0.603953
C	-1.363791	1.948368	-1.467614
H	-1.715183	1.158882	-2.126792
H	-1.060720	2.802207	-2.071406
C	-0.388999	1.980143	1.036554
H	0.464054	2.457061	1.510494
H	-0.696946	1.123841	1.631053
C	-1.552684	2.931942	0.767741
H	-1.169088	3.913800	0.486026
H	-2.151629	3.050677	1.670681
C	-2.362331	2.336284	-0.381745
H	-2.899678	1.451798	-0.042385
H	-3.090770	3.044359	-0.776633
C	-0.057127	-0.851688	-0.380379
C	0.975581	-1.326485	0.614617
H	0.708751	-0.996699	1.636223
O	0.795502	-2.649354	0.371058
C	2.365379	-0.769094	0.330783
C	3.113315	-1.265305	-0.735722
C	2.908248	0.246956	1.114047
C	4.367821	-0.742786	-1.025446
H	2.701924	-2.075237	-1.323373
C	4.165502	0.773099	0.831124
H	2.343570	0.625830	1.957771
C	4.897464	0.281989	-0.244215
H	4.937858	-1.136305	-1.857637
H	4.575105	1.559566	1.452228
H	5.876682	0.685681	-0.466495
H	0.254188	-1.046334	-1.399017
C	-1.489901	-1.069396	-0.126775
C	-2.364315	-1.212910	-1.209139
C	-2.018480	-1.097811	1.169238
C	-3.730447	-1.355388	-1.007751
H	-1.962897	-1.217910	-2.214093
C	-3.382592	-1.242659	1.370789
H	-1.358258	-1.023748	2.020700
C	-4.244958	-1.363339	0.284046
H	-4.391756	-1.464950	-1.856941
H	-3.775788	-1.266312	2.378453
H	-5.308914	-1.475286	0.445010

H-TS4-H
Number of imaginary frequencies: 1
Electronic Energy (SCF) = -1171.73993138 a.u
ZPV corrected Energy = -1171.406578 a.u.
Enthalpy = -1171.387708 a.u.
Free Energy = -1171.454447 a.u.

S	0.151129	1.379912	-0.489200
C	-1.267269	2.073473	-1.415184
H	-1.936356	1.255902	-1.676304
H	-0.881610	2.527921	-2.324397
C	-0.491657	1.896391	1.152113

H	0.175676	2.675543	1.514429
H	-0.441483	1.046720	1.826640
C	-1.910677	2.411772	0.919622
H	-2.187643	3.113648	1.706003
H	-2.611297	1.577993	0.947483
C	-1.935323	3.060492	-0.462118
H	-2.951434	3.273237	-0.794579
H	-1.380017	3.999656	-0.449391
C	-0.063622	-0.845049	-0.416118
C	0.972670	-1.382578	0.544956
H	0.702597	-1.124864	1.586429
O	0.800010	-2.686270	0.211896
C	2.359734	-0.799795	0.302683
C	2.894465	0.165422	1.152937
C	3.111888	-1.218224	-0.793542
C	4.148211	0.717949	0.907581
H	2.326118	0.484559	2.018687
C	4.362685	-0.668216	-1.046528
H	2.706242	-1.989828	-1.434332
C	4.884390	0.305444	-0.197585
H	4.551528	1.464220	1.580268
H	4.935883	-1.000414	-1.902941
H	5.860620	0.730556	-0.391291
H	0.246026	-0.973904	-1.445511
C	-1.495364	-1.079562	-0.173018
C	-2.373342	-1.154507	-1.259886
C	-2.019226	-1.194287	1.119765
C	-3.737780	-1.326458	-1.063999
H	-1.975400	-1.088892	-2.264248
C	-3.380469	-1.366610	1.315398
H	-1.357481	-1.159840	1.972505
C	-4.246200	-1.427782	0.225571
H	-4.400996	-1.385991	-1.916684
H	-3.769900	-1.457823	2.320686
H	-5.307888	-1.564085	0.382693

TS between A-anti-H and D-anti-H
Number of imaginary frequencies: 1
Electronic Energy (SCF) = -1171.73816425 a.u
ZPV corrected Energy = -1171.404311 a.u.
Enthalpy = -1171.386181 a.u.
Free Energy = -1171.450193 a.u.

S	-1.230265	-1.594418	0.003037
C	-2.901784	-1.820550	0.753345
H	-2.976170	-1.101344	1.564520
H	-2.944265	-2.827469	1.159060
C	-1.896183	-0.902544	-1.558780
H	-1.859776	-1.729691	-2.266537
H	-1.236835	-0.111782	-1.899516
C	-3.320348	-0.465875	-1.252443
H	-3.880999	-0.346481	-2.179083
H	-3.308306	0.493375	-0.734154
C	-3.913411	-1.550934	-0.358204
H	-4.863878	-1.249931	0.081104
H	-4.081834	-2.459984	-0.936653
C	-0.470614	-0.204032	1.054649
C	0.904874	-0.692246	1.699141
O	1.136692	0.106047	2.732540
H	-1.150901	-0.196512	1.901447
C	-0.494895	1.132413	0.388998
C	-1.257120	2.153081	0.962162
C	0.242804	1.419138	-0.763675
C	-1.278726	3.427096	0.408577
H	-1.828160	1.945023	1.857354
C	0.216358	2.690112	-1.323110
H	0.843853	0.651438	-1.226401
C	-0.542194	3.698952	-0.738906
H	-1.868558	4.205720	0.873951
H	0.794526	2.892894	-2.214848
H	-0.557231	4.689528	-1.173813
H	0.686052	-1.761990	1.960957
C	2.025960	-0.758533	0.650696
C	2.152842	-1.819910	-0.246128
C	2.956942	0.275657	0.589835
C	3.159973	-1.828551	-1.206940
H	1.469680	-2.658119	-0.186929
C	3.967322	0.273055	-0.364839
H	2.861777	1.076232	1.310097
C	4.067421	-0.775517	-1.275313
H	3.243484	-2.661141	-1.894336
H	4.678638	1.088907	-0.400919
H	4.854264	-0.781224	-2.018593

2-tBu
Number of imaginary frequencies: 0
Electronic Energy (SCF) = -660.431683791 a.u
ZPV corrected Energy = -660.098228 a.u.
Enthalpy = -660.079851 a.u.
Free Energy = -660.142731 a.u.

C	0.325486	3.404156	-0.000178
H	-0.632167	3.957676	-0.001295
C	0.183219	1.941827	0.000472
C	-1.101983	1.400638	-0.000198
C	1.306252	1.111288	0.001809
C	-1.289554	0.020835	0.000019
H	-1.944422	2.077112	-0.000819
C	1.155874	-0.268843	0.002312
H	2.281206	1.573146	0.002318
C	-0.147569	-0.781458	0.000866
H	-0.274732	-1.854428	0.000731
O	1.380278	4.008778	0.000356
C	-2.675240	-0.631052	0.000081
C	2.343901	-1.234972	0.000208
C	-2.823744	-1.510831	-1.254126
H	-3.807850	-1.983080	-1.266312
H	-2.721979	-0.913553	-2.161847
H	-2.073103	-2.300608	-1.284565
C	-2.825015	-1.506776	1.257024
H	-2.074399	-2.296456	1.290702
H	-2.724033	-0.906528	2.162877
H	-3.809171	-1.978906	1.269834
C	-3.802722	0.406419	-0.002113
H	-4.765690	-0.105723	-0.002005
H	-3.766809	1.045739	0.881215
H	-3.765658	1.043270	-0.887177
C	2.264460	-2.144116	1.239718
H	2.296817	-1.554124	2.157273
H	1.348127	-2.734194	1.249655
H	3.108391	-2.836383	1.250461
C	2.288235	-2.102256	-1.270365
H	2.340687	-1.482104	-2.166829
H	3.130216	-2.796868	-1.287241
H	1.370009	-2.687824	-1.318029
C	3.687350	-0.499307	0.026121
H	4.499169	-1.227745	0.027401
H	3.816349	0.139879	-0.848310
H	3.793126	0.118171	0.919216

ylide-tBu 1st conformer
Number of imaginary frequencies: 0
Electronic Energy (SCF) = -1140.67801050 a.u
ZPV corrected Energy = -1140.233999 a.u.
Enthalpy = -1140.210682 a.u.

Free Energy = -1140.284670 a.u.

S	-2.810025	-1.980238	-0.075229
C	-3.426595	-0.938603	1.334315
H	-2.593899	-0.335122	1.687233
H	-3.731269	-1.628722	2.116939
C	-3.448115	-0.755694	-1.346746
H	-4.167955	-1.316819	-1.939231
H	-2.604413	-0.484054	-1.974660
C	-4.090366	0.409923	-0.606641
H	-4.921936	0.821311	-1.178326
H	-3.363691	1.209903	-0.464649
C	-4.558327	-0.105400	0.762253
H	-4.812571	0.716464	1.431677
H	-5.451916	-0.721873	0.642327
C	-1.153919	-2.162678	-0.082521
H	-0.868581	-3.201710	-0.150286
C	-0.139555	-1.144638	-0.045147
C	1.220680	-1.539537	-0.028863
C	-0.405060	0.232409	-0.030795
C	2.253334	-0.618545	0.002575
H	1.434452	-2.598413	-0.041130
C	0.614802	1.185991	0.001287
H	-1.427389	0.571429	-0.047651
C	1.935670	0.751050	0.018739
H	2.737032	1.471261	0.043120
C	0.238167	2.673297	0.009500
C	3.728741	-1.038168	0.020055
C	-0.636289	2.975811	1.239834
H	-0.906974	4.033677	1.261177
H	-1.558207	2.394915	1.230438
H	-0.101682	2.742947	2.162486
C	1.465165	3.590041	0.060150
H	2.058928	3.421821	0.959680
H	2.112052	3.448673	-0.806667
H	1.140872	4.631931	0.065920
C	-0.556534	3.005576	-1.266560
H	0.037689	2.798039	-2.158254
H	-1.473953	2.420676	-1.330902
H	-0.830234	4.062884	-1.278488
C	4.401278	-0.490785	1.292245
H	4.353958	0.597096	1.335938
H	3.916504	-0.885257	2.187157
H	5.453387	-0.782740	1.319029
C	4.439688	-0.457054	-1.215846
H	5.492588	-0.747436	-1.217887
H	3.983266	-0.828025	-2.135373
H	4.391862	0.631554	-1.232161
C	3.909822	-2.559751	0.002451
H	3.480415	-3.008609	-0.894280
H	4.974420	-2.799098	0.016182
H	3.452106	-3.032365	0.872589

ylide-tBu 2nd conformer
Number of imaginary frequencies: 0
Electronic Energy (SCF) = -1140.67417817 a.u
ZPV corrected Energy = -1140.230883 a.u.
Enthalpy = -1140.207388 a.u.
Free Energy = -1140.282867 a.u.

S	3.033674	0.247093	-0.211142
C	4.117057	0.126832	1.316397
H	3.465728	-0.193354	2.125347
H	4.475813	1.137381	1.513785
C	4.355265	-0.484147	-1.280518
H	4.400853	0.103093	-2.193961
H	4.006410	-1.487776	-1.515966
C	5.643322	-0.496355	-0.472933
H	6.126472	0.481681	-0.513545
H	6.338374	-1.229055	-0.882992
C	5.250276	-0.821535	0.971479
H	4.906616	-1.855914	1.038176
H	6.089741	-0.704069	1.656966
C	1.770076	-0.847259	-0.204517
H	2.020681	-1.894997	-0.101023
C	0.391992	-0.436065	-0.114723
C	-0.607521	-1.432374	-0.068974
C	-0.038100	0.907572	-0.082730
C	-1.960783	-1.120624	-0.007894
H	-0.288655	-2.464360	-0.088006
C	-1.384385	1.247793	-0.028287
H	0.707570	1.687116	-0.099235
C	-2.338052	0.225382	0.009352
H	-3.385937	0.480692	0.059289
C	-3.047667	-2.203214	0.038634
C	-1.854236	2.708785	0.002085
C	-3.977755	-2.046382	-1.178224
H	-3.418265	-2.151501	-2.109583
H	-4.756326	-2.812349	-1.161108
H	-4.465591	-1.071950	-1.188063
C	-2.470379	-3.622785	0.015788
H	-1.821648	-3.810389	0.872497
H	-3.285915	-4.347004	0.052085
H	-1.898089	-3.813071	-0.893176
C	-3.873832	-2.044577	1.327987
H	-3.239052	-2.147490	2.210063
H	-4.360414	-1.070701	1.376317
H	-4.650433	-2.811149	1.377005
C	-0.691888	3.706164	-0.058209
H	-1.085107	4.723934	-0.038277
H	-0.017032	3.594926	0.791709
H	-0.108588	3.593312	-0.973179
C	-2.638600	2.966604	1.301506
H	-2.010839	2.783801	2.175624
H	-2.980153	4.003482	1.339064
H	-3.513772	2.321655	1.376085
C	-2.774214	2.975573	-1.203232
H	-2.244910	2.798406	-2.141320
H	-3.653991	2.332751	-1.187708
H	-3.115775	4.013093	-1.196527

3-cis-tButBu
Number of imaginary frequencies: 0
Electronic Energy (SCF) = -1245.58131953 a.u
ZPV corrected Energy = -1244.914141 a.u.
Enthalpy = -1244.879341 a.u.
Free Energy = -1244.978096 a.u.

C	-0.586215	0.304092	3.127493
H	-0.816062	0.948399	3.972588
C	0.760590	-0.305481	3.097938
H	1.428024	-0.061330	3.918676
O	-0.390702	-1.068498	3.491658
C	1.428628	-0.637972	1.813863
C	2.618938	0.013112	1.496288
C	0.846045	-1.506738	0.898013
C	3.221854	-0.168559	0.253073
H	3.055492	0.677833	2.227574
C	1.420745	-1.712513	-0.354207
H	-0.071577	-1.998398	1.172745
C	2.599601	-1.028901	-0.653779
H	3.047800	-1.166968	-1.626846
C	-1.331620	0.588065	1.867942
C	-2.405994	-0.208668	1.474970
C	-0.918040	1.639993	1.065677
C	-3.072490	0.040037	0.279584

H	-2.693030	-1.033285	2.108734
C	-1.548204	1.906942	-0.152721
H	-0.068380	2.231506	1.378278
C	-2.621813	1.100503	-0.517382
H	-3.124956	1.288022	-1.452499
C	-1.019495	3.036201	-1.042050
C	-4.267379	-0.796140	-0.191259
C	0.764145	-2.605095	-1.412092
C	4.517823	0.542957	-0.149707
C	0.223975	-1.704711	-2.539192
H	1.026289	-1.133607	-3.007495
H	-0.257334	-2.309357	-3.310948
H	-0.511049	-0.998717	-2.151105
C	1.791833	-3.589226	-1.995747
H	1.314510	-4.229351	-2.740238
H	2.618779	-3.074346	-2.483707
H	2.205988	-4.228715	-1.214152
C	-0.401605	-3.418412	-0.838683
H	-0.074844	-4.070024	-0.026597
H	-1.197293	-2.777539	-0.461552
H	-0.827275	-4.046845	-1.622328
C	4.256166	1.417138	-1.389819
H	5.175026	1.922707	-1.693833
H	3.904628	0.824194	-2.233984
H	3.503615	2.177725	-1.177519
C	5.592265	-0.506447	-0.487038
H	6.522883	-0.013635	-0.775809
H	5.797815	-1.142727	0.375616
H	5.282124	-1.147808	-1.311858
C	5.059138	1.443569	0.965475
H	4.352512	2.232211	1.228132
H	5.289458	0.876263	1.868424
H	5.980307	1.922406	0.630310
C	0.462358	2.769467	-1.366401
H	0.853482	3.556815	-2.013944
H	1.071748	2.744080	-0.464203
H	0.584062	1.813979	-1.878171
C	-1.140516	4.375208	-0.293101
H	-0.757357	5.189674	-0.911334
H	-2.182546	4.592758	-0.051812
H	-0.573631	4.365712	0.638002
C	-1.787732	3.148720	-2.362655
H	-1.716356	2.232491	-2.950550
H	-2.843739	3.369833	-2.200936
H	-1.367004	3.959677	-2.958888
C	-3.970523	-1.395701	-1.577766
H	-3.099223	-2.049792	-1.540612
H	-4.823477	-1.985136	-1.920145
H	-3.779619	-0.622411	-2.321402
C	-5.507026	0.111787	-0.289331
H	-5.744203	0.551404	0.681120
H	-5.351528	0.924826	-0.998700
H	-6.372348	-0.465012	-0.622379
C	-4.586150	-1.947291	0.768261
H	-3.746400	-2.636706	0.865963
H	-4.847996	-1.585642	1.763515
H	-5.437508	-2.512813	0.386735

3-trans-tButBu

Number of imaginary frequencies: 0

Electronic Energy (SCF) = -1245.57927815 a.u

ZPV corrected Energy = -1244.912517 a.u.

Enthalpy = -1244.877383 a.u.

Free Energy = -1244.979635 a.u.

C	-0.481936	0.561112	0.837646
C	0.481906	-0.560823	0.837645
O	0.000020	0.000175	2.067618
H	0.073754	-1.542123	0.615063
C	1.914908	-0.380899	0.499486
C	2.506528	-1.250905	-0.411191
C	2.664143	0.653642	1.052263
C	3.838559	-1.094602	-0.794613
H	1.912100	-2.056674	-0.821426
C	3.999343	0.837391	0.698856
H	2.193715	1.308313	1.771859
C	4.562982	-0.044569	-0.227327
H	5.593303	0.087113	-0.508663
H	-0.073804	1.542406	0.614995
C	-1.914949	0.381136	0.499560
C	-2.506634	1.251036	-0.411177
C	-2.664122	-0.653389	1.052446
C	-3.838675	1.094642	-0.794529
H	-1.912256	2.056793	-0.821510
C	-3.999327	-0.837234	0.699105
H	-2.193626	-1.308009	1.772039
C	-4.563041	0.044636	-0.227118
H	-5.593378	-0.087086	-0.508377
C	4.796087	1.984272	1.330722
C	4.455367	-2.068981	-1.804032
C	-4.455556	2.068884	-1.804034
C	-4.795894	-1.984305	1.330853
C	4.111262	3.322977	1.000549
H	3.093071	3.360173	1.387469
H	4.668862	4.150258	1.444236
H	4.068874	3.481821	-0.078505
C	4.829409	1.797087	2.858343
H	5.311347	0.854390	3.123523
H	5.390260	2.609185	3.325456
H	3.826007	1.793678	3.283679
C	6.240207	2.046227	0.821987
H	6.792074	1.134452	1.054764
H	6.283945	2.206175	-0.256306
H	6.757420	2.878263	1.301907
C	5.920524	-1.743424	-2.113151
H	6.032296	-0.748368	-2.546244
H	6.545604	-1.799579	-1.220900
H	6.306993	-2.464612	-2.834690
C	3.660737	-2.009229	-3.121297
H	4.089282	-2.699288	-3.850905
H	2.616216	-2.283602	-2.974235
H	3.689161	-1.004330	-3.546335
C	4.388443	-3.498634	-1.236990
H	4.945348	-3.571339	-0.301154
H	3.361009	-3.805919	-1.042613
H	4.822007	-4.206024	-1.946799
C	-4.828248	-1.798167	2.858616
H	-5.389035	-2.610462	3.325459
H	-3.824618	-1.795338	3.283414
H	-5.309806	-0.855543	3.124744
C	-4.111443	-3.322887	0.999351
H	-3.093010	-3.360399	1.385618
H	-4.668841	-4.150421	1.442828
H	-4.069735	-3.481009	-0.079834
C	-6.240354	-2.045673	0.823021
H	-6.791789	-1.133816	1.056505
H	-6.284856	-2.205204	-0.255301
H	-6.757490	-2.877755	1.302943
C	-3.660957	2.009019	-3.121315
H	-4.089552	2.698983	-3.850983
H	-2.616444	2.283450	-2.974313
H	-3.689356	1.004071	-3.546239
C	-4.388677	3.498600	-1.237156
H	-3.361247	3.805963	-1.042880
H	-4.822323	4.205890	-1.947016
H	-4.945527	3.571387	-0.301292
C	-5.920703	1.743212	-2.113080

H	-6.032426	0.748118	-2.546097
H	-6.545762	1.799391	-1.220814
H	-6.307242	2.464325	-2.834656

A-gauche-0-tBu
Number of imaginary frequencies: 0
Electronic Energy (SCF) = -1801.12194090 a.u
ZPV corrected Energy = -1800.343941 a.u.
Enthalpy = -1800.300883 a.u.
Free Energy = -1800.420943 a.u.

S	0.711070	2.100156	1.148561
C	0.974299	3.762492	1.979182
H	0.007889	4.067113	2.371948
H	1.277314	4.445558	1.185393
C	2.262163	1.416996	1.876827
H	2.750403	0.832161	1.105828
H	1.937628	0.757087	2.679352
C	3.073205	2.593098	2.391191
H	3.604064	3.075275	1.567829
H	3.815740	2.254234	3.113486
C	2.070054	3.570622	3.012924
H	1.652651	3.138098	3.924618
H	2.536848	4.519645	3.277170
C	-0.541804	1.210072	1.802182
C	1.876468	1.716288	-2.242912
O	2.659714	2.618316	-2.470459
H	0.807913	1.829824	-2.497633
C	2.219619	0.429052	-1.623431
C	1.187560	-0.473879	-1.357082
C	3.529856	0.134820	-1.258517
C	1.451032	-1.677605	-0.717208
H	0.181202	-0.202665	-1.638339
C	3.830035	-1.059511	-0.607189
H	4.299910	0.861651	-1.473425
C	2.777001	-1.938357	-0.347878
H	2.987684	-2.860847	0.168797
H	-0.463066	0.924460	2.842418
C	-1.715909	0.862479	1.049129
C	-2.636202	-0.045390	1.616967
C	-2.004635	1.347857	-0.243884
C	-3.763456	-0.480161	0.932586
H	-2.423915	-0.420186	2.607348
C	-3.122893	0.925480	-0.953605
H	-1.331851	2.066459	-0.685546
C	-3.992663	0.007344	-0.357755
H	-4.861967	-0.328074	-0.903100
C	5.270257	-1.345090	-0.171189
C	0.351360	-2.684669	-0.371132
C	-4.746721	-1.491135	1.537513
C	-3.427884	1.430610	-2.370521
C	5.702716	-0.274656	0.848146
H	6.733147	-0.450478	1.162986
H	5.647024	0.728628	0.425525
H	5.067447	-0.304118	1.734860
C	6.198394	-1.285215	-1.396958
H	7.228750	-1.484836	-1.096825
H	5.909756	-2.031546	-2.139095
H	6.173404	-0.306420	-1.875312
C	5.422202	-2.722532	0.482135
H	4.821729	-2.811138	1.388573
H	5.136450	-3.526687	-0.197561
H	6.465351	-2.877436	0.760527
C	0.747084	-4.089270	-0.858093
H	1.660394	-4.448290	-0.384297
H	-0.048180	-4.799049	-0.623263
H	0.900362	-4.098093	-1.938812
C	0.171972	-2.704566	1.158033
H	-0.099049	-1.714767	1.525782
H	-0.620615	-3.402025	1.436075
H	1.089271	-3.016037	1.659732
C	-0.987648	-2.314783	-1.017773
H	-1.364936	-1.358997	-0.661577
H	-0.906102	-2.271410	-2.105169
H	-1.731046	-3.072677	-0.770196
C	-4.789224	-2.751240	0.654231
H	-5.492597	-3.478649	1.065795
H	-5.102369	-2.518702	-0.363308
H	-3.805596	-3.220496	0.603697
C	-6.152911	-0.867941	1.596894
H	-6.512585	-0.592671	0.605724
H	-6.863733	-1.576608	2.027817
H	-6.152237	0.031261	2.215801
C	-4.355117	-1.920068	2.955812
H	-3.378861	-2.406314	2.976510
H	-4.328719	-1.072988	3.642668
H	-5.088847	-2.633077	3.335623
C	-3.391776	0.242541	-3.349336
H	-4.121934	-0.520265	-3.079656
H	-3.614273	0.579827	-4.364213
H	-2.406040	-0.225371	-3.356100
C	-4.828534	2.068279	-2.402821
H	-4.885762	2.911959	-1.712551
H	-5.053789	2.434888	-3.406722
H	-5.603610	1.354620	-2.125297
C	-2.420735	2.478904	-2.855478
H	-2.405634	3.356708	-2.207897
H	-1.408238	2.077428	-2.906575
H	-2.694606	2.809802	-3.858634

A-gauche-TS0-tBu
Number of imaginary frequencies: 1
Electronic Energy (SCF) = -1801.11120484 a.u
ZPV corrected Energy = -1800.331537 a.u.
Enthalpy = -1800.290403 a.u.
Free Energy = -1800.403830 a.u.

S	-0.982360	3.480137	-0.448548
C	-1.925564	4.023736	1.032897
H	-2.467357	3.160223	1.409999
H	-2.636993	4.771792	0.683393
C	0.417765	4.626094	-0.117970
H	0.580779	5.208782	-1.017846
H	1.280797	3.988723	0.039843
C	0.033452	5.459124	1.106375
H	-0.489198	6.362371	0.788607
H	0.934162	5.764249	1.638086
C	-0.892342	4.613319	1.979399
H	-0.334436	3.812611	2.466834
H	-1.377072	5.206036	2.754782
C	-0.295389	1.900363	-0.051302
C	0.828475	1.610674	-1.751226
O	1.349626	2.700989	-2.129549
H	-0.050914	1.221581	-2.297906
C	1.684499	0.487763	-1.235151
C	1.209762	-0.825819	-1.264008
C	2.953948	0.744710	-0.737376
C	1.987147	-1.879633	-0.798984
H	0.221790	-1.003017	-1.660969
C	3.764077	-0.286234	-0.253815
H	3.302713	1.767817	-0.744982
C	3.262033	-1.585037	-0.296312
H	3.874006	-2.398096	0.060483
H	0.403900	2.003912	0.772561
C	-1.292474	0.842901	0.133864
C	-1.181867	-0.025565	1.227857

C	-2.319307	0.622269	-0.796187
C	-2.072790	-1.080020	1.414201
H	-0.384719	0.149603	1.934420
C	-3.217700	-0.429223	-0.646483
H	-2.397220	1.279407	-1.648681
C	-3.081165	-1.262769	0.466564
H	-3.782426	-2.073106	0.598582
C	5.157528	0.041709	0.293910
C	1.509845	-3.335084	-0.838436
C	-2.014782	-1.994729	2.644060
C	-4.327687	-0.711000	-1.665107
C	5.023122	1.029848	1.466834
H	6.008251	1.277956	1.867599
H	4.543922	1.957948	1.155746
H	4.427985	0.596132	2.272709
C	6.001845	0.689568	-0.818242
H	6.997275	0.936455	-0.442703
H	6.116572	0.008700	-1.663575
H	5.543616	1.607327	-1.186273
C	5.899213	-1.200893	0.798026
H	5.359486	-1.694406	1.607710
H	6.058828	-1.928840	0.001351
H	6.878363	-0.909046	1.181074
C	2.399679	-4.132597	-1.809121
H	3.444429	-4.115626	-1.497392
H	2.076764	-5.175306	-1.850036
H	2.341754	-3.718879	-2.817473
C	1.618860	-3.954384	0.566161
H	1.030507	-3.386868	1.286874
H	1.246617	-4.980819	0.555929
H	2.649996	-3.977978	0.918216
C	0.054809	-3.453017	-1.306338
H	-0.624132	-2.893117	-0.663756
H	-0.071484	-3.091948	-2.327750
H	-0.251242	-4.500174	-1.285761
C	-2.051490	-3.472437	2.217345
H	-2.002946	-4.114822	3.098840
H	-2.966411	-3.718201	1.679470
H	-1.207183	-3.716154	1.574008
C	-3.240006	-1.701076	3.530029
H	-4.170584	-1.890578	2.994191
H	-3.226163	-2.335053	4.419351
H	-3.244114	-0.659073	3.855102
C	-0.749059	-1.768865	3.478662
H	0.155641	-1.931989	2.891959
H	-0.708897	-0.760302	3.891826
H	-0.735931	-2.468134	4.316019
C	-4.106869	-2.107864	-2.273908
H	-4.129848	-2.885900	-1.511063
H	-4.887130	-2.329052	-3.005216
H	-3.141222	-2.161520	-2.779488
C	-5.695967	-0.674589	-0.961354
H	-5.877591	0.305698	-0.516736
H	-6.494274	-0.874946	-1.678923
H	-5.762646	-1.420719	-0.169858
C	-4.348674	0.311558	-2.806153
H	-4.520167	1.324652	-2.439141
H	-3.416007	0.307259	-3.371686
H	-5.156659	0.067526	-3.497451

A-gauche-tBu
Number of imaginary frequencies: 0
Electronic Energy (SCF) = -1801.12570058 a.u
ZPV corrected Energy = -1800.343550 a.u.
Enthalpy = -1800.302387 a.u.
Free Energy = -1800.416772 a.u.

S	-0.850754	3.474046	-0.753572
C	-2.221253	3.711701	0.457574
H	-2.753032	2.772550	0.577163
H	-2.892421	4.437927	-0.000303
C	0.245717	4.653394	0.130929
H	0.612674	5.350836	-0.615497
H	1.081312	4.055563	0.479159
C	-0.562063	5.292601	1.257071
H	-1.079083	6.180231	0.889218
H	0.106617	5.604196	2.059355
C	-1.587149	4.260418	1.726405
H	-1.094342	3.456350	2.275787
H	-2.339921	4.699917	2.380818
C	-0.228957	1.788853	-0.276927
C	0.757695	1.570346	-1.473750
O	1.287196	2.779538	-1.763346
H	0.144042	1.141976	-2.295819
C	1.733039	0.469686	-1.060442
C	1.323769	-0.866844	-1.053029
C	3.011125	0.790404	-0.637697
C	2.165890	-1.875637	-0.604549
H	0.327366	-1.099813	-1.395748
C	3.898518	-0.198566	-0.192999
H	3.300362	1.831167	-0.671955
C	3.453862	-1.516848	-0.178832
H	4.117202	-2.294864	0.165017
H	0.352422	1.957553	0.627275
C	-1.299170	0.781465	-0.031895
C	-1.353993	0.133034	1.199463
C	-2.189355	0.419043	-1.042806
C	-2.275841	-0.885473	1.436619
H	-0.648340	0.423995	1.962528
C	-3.125838	-0.591291	-0.842656
H	-2.131441	0.926108	-1.993426
C	-3.146571	-1.226071	0.401571
H	-3.862368	-2.018363	0.565907
C	5.307773	0.201484	0.257587
C	1.731582	-3.344251	-0.547019
C	-2.354955	-1.633629	2.771186
C	-4.097225	-1.043644	-1.936942
C	5.206621	1.207706	1.418474
H	6.204052	1.504564	1.749858
H	4.669843	2.108730	1.122395
H	4.683573	0.766844	2.269322
C	6.047687	0.862546	-0.919402
H	7.052659	1.162815	-0.614641
H	6.139327	0.169435	-1.757619
H	5.523494	1.749989	-1.273386
C	6.135976	-0.996626	0.734070
H	5.673697	-1.495227	1.587440
H	6.274235	-1.734215	-0.057743
H	7.124545	-0.654967	1.045111
C	2.665087	-4.195217	-1.426032
H	3.700758	-4.130697	-1.092300
H	2.365814	-5.245014	-1.390420
H	2.625117	-3.865607	-2.465805
C	1.816307	-3.839743	0.908268
H	1.174773	-3.242510	1.557745
H	1.491858	-4.880462	0.974114
H	2.834093	-3.780356	1.293931
C	0.292910	-3.544281	-1.037722
H	-0.422937	-2.983089	-0.437069
H	0.175645	-3.239340	-2.078421
H	0.028543	-4.600736	-0.969270
C	-2.114014	-3.134689	2.529943
H	-2.174450	-3.680758	3.473470
H	-2.853376	-3.557608	1.850075
H	-1.125916	-3.305463	2.101448
C	-3.753461	-1.435604	3.382867
H	-4.536478	-1.816911	2.727418

H	-3.826131	-1.964236	4.335429
H	-3.951590	-0.377805	3.565289
C	-1.313062	-1.136006	3.778496
H	-0.295926	-1.270819	3.408517
H	-1.452374	-0.080597	4.017350
H	-1.406710	-1.701229	4.706749
C	-3.824968	-2.522628	-2.267221
H	-3.963875	-3.160859	-1.394593
H	-4.506512	-2.866131	-3.047951
H	-2.802514	-2.657890	-2.623604
C	-5.544577	-0.891914	-1.436612
H	-5.763928	0.149103	-1.192362
H	-6.244864	-1.213970	-2.209767
H	-5.729764	-1.493180	-0.546682
C	-3.945052	-0.227145	-3.224554
H	-4.141415	0.833032	-3.057542
H	-2.946293	-0.326061	-3.651698
H	-4.659855	-0.584595	-3.966967

A-anti-0-tBu
Number of imaginary frequencies: 0
Electronic Energy (SCF) = -1801.12164230 a.u
ZPV corrected Energy = -1800.342949 a.u.
Enthalpy = -1800.300371 a.u.
Free Energy = -1800.416239 a.u.

S	3.178809	-1.166070	0.229152
C	4.346784	-2.390816	1.051138
H	3.728145	-3.212479	1.402578
H	4.779802	-1.867757	1.904312
C	4.361400	-0.869489	-1.153896
H	4.380855	0.198840	-1.350496
H	3.939472	-1.385823	-2.013448
C	5.701402	-1.456853	-0.739552
H	6.233954	-0.764481	-0.084640
H	6.322181	-1.629691	-1.618601
C	5.392938	-2.753892	0.013688
H	4.992139	-3.496805	-0.679068
H	6.284390	-3.176941	0.477197
C	1.835004	-1.877851	-0.472117
H	2.010915	-2.601413	-1.256329
C	1.022787	-0.130556	-2.862721
H	2.124414	-0.118042	-2.891672
O	0.396768	-0.908629	-3.559452
C	0.426430	0.891489	-1.990521
C	1.269093	1.725039	-1.267310
C	-0.960864	1.040630	-1.905794
C	0.752007	2.717900	-0.434478
H	2.337989	1.585808	-1.358515
C	-1.510164	2.035792	-1.112597
H	-1.578575	0.369053	-2.480006
C	-0.632545	2.851499	-0.380995
H	-1.057831	3.617188	0.247960
C	0.532801	-1.786770	0.136162
C	-0.556698	-2.422098	-0.492398
C	0.262775	-1.087162	1.328025
C	-1.847438	-2.361381	0.019968
H	-0.365173	-2.969247	-1.405045
C	-1.015301	-1.011146	1.868572
H	1.081705	-0.610586	1.848154
C	-2.068241	-1.646710	1.202613
H	-3.060679	-1.596699	1.614099
C	-2.978806	-3.114741	-0.695109
C	-1.217337	-0.279101	3.203863
C	-3.017772	2.292731	-1.026666
C	1.703627	3.626523	0.349325
C	-4.360708	-2.776018	-0.122954
H	-5.128285	-3.309190	-0.686473
H	-4.454366	-3.073272	0.922028
H	-4.574849	-1.708659	-0.193884
C	-2.745109	-4.628139	-0.531058
H	-2.734888	-4.907167	0.524273
H	-3.538943	-5.193514	-1.025067
H	-1.792610	-4.929455	-0.967957
C	-2.992209	-2.772824	-2.194929
H	-3.141100	-1.704479	-2.350869
H	-2.062341	-3.054040	-2.686377
H	-3.806170	-3.305372	-2.691682
C	-0.414671	-1.004397	4.300077
H	-0.549066	-0.505464	5.262711
H	-0.746820	-2.039046	4.404277
H	0.651202	-1.014964	4.071910
C	-2.686439	-0.239711	3.639739
H	-3.091476	-1.240889	3.792513
H	-2.769680	0.298368	4.585630
H	-3.312362	0.273340	2.909476
C	-0.711485	1.169033	3.090283
H	0.349044	1.204157	2.847248
H	-1.249202	1.707283	2.311676
H	-0.857010	1.695893	4.036156
C	-3.820216	1.313721	-1.888146
H	-4.883940	1.539576	-1.799904
H	-3.551187	1.384315	-2.942965
H	-3.670720	0.284157	-1.566009
C	-3.487539	2.144109	0.429668
H	-3.290736	1.136541	0.793423
H	-2.983132	2.847714	1.091454
H	-4.561265	2.330766	0.498004
C	-3.312930	3.723312	-1.513410
H	-4.385672	3.921636	-1.467081
H	-2.807800	4.469873	-0.900182
H	-2.986708	3.857766	-2.546323
C	0.960047	4.552228	1.317815
H	0.374153	3.987266	2.044050
H	1.682398	5.156508	1.868362
H	0.290017	5.235566	0.794586
C	2.689189	2.770190	1.163066
H	2.159115	2.130023	1.868243
H	3.294582	2.130975	0.521745
H	3.365815	3.414785	1.727416
C	2.495045	4.492745	-0.647508
H	3.075700	3.876205	-1.334588
H	1.823299	5.116384	-1.239908
H	3.187083	5.147933	-0.114259

A-anti-TS0-tBu
Number of imaginary frequencies: 1
Electronic Energy (SCF) = -1801.11104309 a.u
ZPV corrected Energy = -1800.331638
Enthalpy = -1800.290367
Free Energy = -1800.404033

S	2.243482	-2.324935	0.050161
C	2.006153	-3.798948	1.139010
H	0.967559	-3.800080	1.457930
H	2.650246	-3.638598	2.002551
C	3.321361	-3.223589	-1.137785
H	4.180802	-2.596293	-1.354319
H	2.733615	-3.358913	-2.041638
C	3.671623	-4.551855	-0.471068
H	4.514353	-4.418835	0.208930
H	3.964640	-5.274378	-1.231848
C	2.437950	-4.999383	0.310432
H	1.639498	-5.287805	-0.374500
H	2.650155	-5.850054	0.957406
C	0.765400	-2.081707	-0.892476

H	0.561187	-2.978018	-1.469646
C	1.087021	-0.874321	-2.384055
H	2.141984	-1.170622	-2.558544
O	0.228466	-1.164919	-3.276877
C	1.048954	0.479524	-1.714027
C	2.153857	0.959563	-1.027070
C	-0.110686	1.252173	-1.772933
C	2.122833	2.190869	-0.365461
H	3.057114	0.362290	-1.010178
C	-0.178086	2.489500	-1.143676
H	-0.944648	0.849043	-2.324866
C	0.948230	2.934360	-0.437727
H	0.898090	3.889012	0.061680
C	-0.380355	-1.610842	-0.098714
C	-1.663967	-1.823448	-0.610010
C	-0.248397	-0.881927	1.087712
C	-2.793753	-1.305751	0.019656
H	-1.761215	-2.389242	-1.524697
C	-1.355645	-0.369640	1.757088
H	0.737522	-0.706625	1.494094
C	-2.620234	-0.582772	1.202162
H	-3.485179	-0.187034	1.705249
C	-4.179110	-1.573366	-0.582028
C	-1.160419	0.356909	3.094129
C	-1.427886	3.376023	-1.196028
C	3.367353	2.682888	0.382018
C	-5.297243	-0.854664	0.181007
H	-6.254691	-1.059469	-0.300511
H	-5.368594	-1.193791	1.215319
H	-5.151209	0.226529	0.185561
C	-4.457964	-3.087179	-0.538468
H	-4.435576	-3.457019	0.488402
H	-5.443506	-3.303307	-0.956609
H	-3.718549	-3.644188	-1.114207
C	-4.217601	-1.097606	-2.044903
H	-4.046714	-0.022770	-2.108944
H	-3.460978	-1.591532	-2.653086
H	-5.194771	-1.312626	-2.482402
C	-0.715137	-0.673940	4.148535
H	-0.569716	-0.189893	5.116902
H	-1.465695	-1.457333	4.268865
H	0.224551	-1.148437	3.863134
C	-2.446358	1.030073	3.586823
H	-3.238235	0.306184	3.782183
H	-2.245000	1.557155	4.520697
H	-2.819672	1.758395	2.865726
C	-0.074656	1.438340	2.965100
H	0.888674	1.014517	2.688179
H	-0.343315	2.173779	2.209047
H	0.051697	1.955071	3.918689
C	-2.552180	2.741800	-2.019905
H	-3.416409	3.408173	-2.032399
H	-2.250151	2.566461	-3.053193
H	-2.869394	1.791083	-1.593582
C	-1.957216	3.608087	0.230018
H	-2.234742	2.662148	0.695164
H	-1.213197	4.090995	0.863381
H	-2.841967	4.248208	0.204190
C	-1.067580	4.731622	-1.829972
H	-1.950807	5.372251	-1.879707
H	-0.305481	5.256263	-1.253475
H	-0.688425	4.596326	-2.844553
C	3.106325	3.974276	1.164814
H	2.312755	3.843438	1.902186
H	4.012590	4.266765	1.697566
H	2.829116	4.799568	0.508013
C	3.838942	1.609634	1.379382
H	3.063025	1.395460	2.115486
H	4.094948	0.676185	0.879092
H	4.726474	1.956739	1.912470
C	4.490187	2.948505	-0.637280
H	4.742769	2.045467	-1.193905
H	4.188358	3.712493	-1.355847
H	5.392384	3.295925	-0.128795

A-anti-tBu

Number of imaginary frequencies: 0
Electronic Energy (SCF) = -1801.11470779 a.u
ZPV corrected Energy = -1800.333509 a.u.
Enthalpy = -1800.292175 a.u.
Free Energy = -1800.405837 a.u.

S	-0.158090	3.219613	-0.004085
C	1.176944	4.016755	0.975484
H	1.907228	3.252156	1.223358
H	0.701189	4.368777	1.889023
C	-0.322427	4.629900	-1.174033
H	-1.380612	4.839579	-1.296825
H	0.093508	4.295255	-2.120401
C	0.476348	5.777881	-0.559853
H	-0.132091	6.303862	0.177190
H	0.749293	6.487492	-1.339912
C	1.697793	5.160940	0.116224
H	2.393949	4.779409	-0.631477
H	2.230117	5.880856	0.736939
C	0.704555	1.970262	-1.040235
H	1.460513	2.532698	-1.583589
C	-0.279094	1.402518	-2.205906
H	-0.952831	2.269090	-2.428629
O	0.478222	1.009186	-3.216561
C	-1.192185	0.336110	-1.586609
C	-2.335913	0.677385	-0.882401
C	-0.849140	-1.008413	-1.717212
C	-3.136271	-0.295743	-0.270677
H	-2.616628	1.721497	-0.812806
C	-1.620568	-2.007932	-1.138088
H	0.035659	-1.229778	-2.292625
C	-2.756785	-1.626945	-0.409249
H	-3.357791	-2.397033	0.048362
C	1.327161	0.913273	-0.184262
C	2.468703	0.275210	-0.661202
C	0.767417	0.490957	1.019547
C	3.042980	-0.790461	0.031575
H	2.886555	0.606808	-1.599612
C	1.332728	-0.544461	1.759143
H	-0.129332	0.969592	1.386800
C	2.463901	-1.178988	1.240881
H	2.906341	-1.989073	1.794427
C	4.278928	-1.484926	-0.550750
C	0.717122	-0.925810	3.110298
C	-1.282103	-3.496664	-1.279988
C	-4.390707	0.130742	0.500483
C	4.786422	-2.622785	0.341250
H	5.658858	-3.085343	-0.122655
H	5.086234	-2.264041	1.327136
H	4.031706	-3.398990	0.475153
C	5.412825	-0.456557	-0.714223
H	5.679031	-0.015051	0.248077
H	6.302391	-0.938789	-1.124613
H	5.128208	0.350270	-1.389425
C	3.927615	-2.073196	-1.929181
H	3.143177	-2.825730	-1.840552
H	3.576217	-1.305112	-2.617394
H	4.806504	-2.547899	-2.370543
C	0.843588	0.274821	4.066757
H	0.414793	0.030162	5.040835
H	1.890478	0.546567	4.214505

H	0.319699	1.148782	3.678445
C	1.413630	-2.130072	3.752292
H	2.465753	-1.929819	3.959567
H	0.928846	-2.365786	4.700826
H	1.353244	-3.016147	3.118905
C	-0.771181	-1.269481	2.930693
H	-1.331240	-0.435629	2.512069
H	-0.896445	-2.120340	2.262847
H	-1.216315	-1.521873	3.895391
C	-0.003268	-3.723314	-2.092380
H	0.201226	-4.793307	-2.160177
H	-0.092503	-3.335289	-3.107669
H	0.857296	-3.246763	-1.623310
C	-1.077600	-4.121774	0.110972
H	-0.239465	-3.650327	0.624909
H	-1.962897	-4.014017	0.737414
H	-0.861495	-5.188287	0.016285
C	-2.442701	-4.216330	-1.990874
H	-2.214098	-5.277744	-2.110385
H	-3.371789	-4.134887	-1.426261
H	-2.612612	-3.791557	-2.981830
C	-5.102511	-1.053515	1.162937
H	-4.452865	-1.568427	1.872326
H	-5.975353	-0.694141	1.710482
H	-5.449499	-1.781048	0.428109
C	-4.006468	1.133039	1.603819
H	-3.315384	0.680271	2.316130
H	-3.530908	2.023007	1.192459
H	-4.896530	1.451801	2.150475
C	-5.375445	0.806157	-0.471174
H	-4.934523	1.687382	-0.937695
H	-5.669509	0.117701	-1.265340
H	-6.277396	1.120446	0.058772

B-gauche-0-tBu
Number of imaginary frequencies: 0
Electronic Energy (SCF) = -1801.12151520 a.u
ZPV corrected Energy = -1800.343362 a.u.
Enthalpy = -1800.300342 a.u.
Free Energy = -1800.419543 a.u.

S	0.669397	2.352908	1.051989
C	0.932473	3.839582	2.155101
H	0.223547	3.703239	2.969240
H	0.661522	4.725370	1.585663
C	2.342622	1.673738	1.479641
H	2.998887	1.946169	0.655868
H	2.234174	0.594769	1.513163
C	2.770224	2.341165	2.772972
H	3.841129	2.221948	2.938002
H	2.248019	1.893296	3.621023
C	2.378423	3.814445	2.627938
H	2.483087	4.359250	3.566394
H	3.025055	4.293408	1.890449
C	-0.515810	1.378331	1.710241
C	1.829600	1.561427	-2.387264
O	2.609344	2.452203	-2.666615
H	0.761948	1.653881	-2.653449
C	2.176198	0.318716	-1.686104
C	3.489096	0.050847	-1.310472
C	1.147347	-0.567455	-1.357034
C	3.795011	-1.093886	-0.580039
H	4.256481	0.761881	-1.580447
C	1.417914	-1.726649	-0.641655
H	0.139079	-0.319785	-1.653169
C	2.745394	-1.956274	-0.258227
H	2.960933	-2.842351	0.316999
H	-0.403621	1.084617	2.745399
C	-1.672548	0.945347	0.973912
C	-2.574138	0.060188	1.604844
C	-1.975507	1.336889	-0.347912
C	-3.705754	-0.426565	0.963482
H	-2.349558	-0.246086	2.615859
C	-3.102105	0.866837	-1.012191
H	-1.308443	2.022278	-0.847185
C	-3.957917	-0.018920	-0.349333
H	-4.830109	-0.395207	-0.862232
C	-4.672711	-1.404911	1.644143
C	-3.428694	1.276458	-2.455011
C	0.326778	-2.720392	-0.235982
C	5.238516	-1.348754	-0.136120
C	-3.380467	0.029215	-3.356598
H	-4.094292	-0.727130	-3.030778
H	-3.618409	0.296728	-4.388657
H	-2.386494	-0.420833	-3.343804
C	-2.445468	2.309435	-3.015602
H	-2.436822	3.224629	-2.421877
H	-1.427360	1.921884	-3.056666
H	-2.737262	2.574955	-4.033044
C	-4.840430	1.887340	-2.514083
H	-5.079551	2.186760	-3.536873
H	-5.601401	1.180070	-2.186142
H	-4.906442	2.771445	-1.877240
C	-4.716995	-2.719499	0.844353
H	-5.415205	-3.420696	1.307049
H	-5.037864	-2.553869	-0.183814
H	-3.732612	-3.188631	0.817057
C	-4.260259	-1.739368	3.081888
H	-3.278235	-2.212561	3.121506
H	-4.235924	-0.850441	3.713763
H	-4.981422	-2.434905	3.514513
C	-6.083340	-0.790015	1.681915
H	-6.459123	-0.584243	0.679964
H	-6.781905	-1.474079	2.169020
H	-6.082194	0.148267	2.239760
C	0.728994	-4.144830	-0.655995
H	1.648378	-4.473025	-0.171622
H	-0.059438	-4.847712	-0.380741
H	0.874997	-4.205830	-1.736024
C	0.158127	-2.666808	1.293471
H	-0.124330	-1.664030	1.614060
H	-0.623103	-3.359664	1.611943
H	1.082398	-2.941339	1.803714
C	-1.018250	-2.390446	-0.890627
H	-1.396339	-1.419284	-0.580223
H	-0.944677	-2.402108	-1.979351
H	-1.756502	-3.137655	-0.599511
C	5.391286	-2.666359	0.630553
H	4.795499	-2.676246	1.544406
H	5.099895	-3.524514	0.023134
H	6.435691	-2.800021	0.915235
C	6.152417	-1.398428	-1.373131
H	5.852118	-2.203826	-2.045625
H	6.124935	-0.463608	-1.932672
H	7.185570	-1.576665	-1.068992
C	5.688475	-0.198181	0.783139
H	5.639230	0.764547	0.274568
H	5.060192	-0.144448	1.673593
H	6.719759	-0.356086	1.104602

B-gauche-TS0-tBu
Number of imaginary frequencies: 1
Electronic Energy (SCF) = -1801.11153027 a.u
ZPV corrected Energy = -1800.331974 a.u.
Enthalpy = -1800.290827 a.u.
Free Energy = -1800.404742 a.u.

S	-1.130512	3.463892	-0.469364
C	-2.003692	4.013168	1.073458
H	-1.953190	3.166834	1.754255
H	-3.042240	4.208413	0.819891
C	0.231716	4.665606	-0.265702
H	-0.012478	5.504371	-0.915435
H	1.123496	4.172266	-0.641898
C	0.220496	5.060038	1.203411
H	0.792997	5.975815	1.349375
H	0.681364	4.275863	1.805911
C	-1.247459	5.236506	1.589452
H	-1.381750	5.329441	2.666801
H	-1.643489	6.139870	1.123494
C	-0.344784	1.928822	-0.096055
C	0.765378	1.623052	-1.807981
O	1.286181	2.704026	-2.214698
H	-0.113812	1.221485	-2.344026
C	1.629525	0.515392	-1.271984
C	2.879996	0.798258	-0.740562
C	1.186949	-0.808329	-1.321243
C	3.703056	-0.216722	-0.246885
H	3.203972	1.829493	-0.728970
C	1.980031	-1.848134	-0.849308
H	0.212355	-1.005575	-1.741247
C	3.235249	-1.527520	-0.315646
H	3.860351	-2.328557	0.045055
H	0.372051	2.074707	0.706986
C	-1.287769	0.828462	0.133515
C	-1.106989	-0.021096	1.231597
C	-2.331514	0.553878	-0.762487
C	-1.946845	-1.110022	1.456946
H	-0.294699	0.193642	1.909389
C	-3.181543	-0.530377	-0.572987
H	-2.460136	1.195848	-1.620357
C	-2.977324	-1.343405	0.545441
H	-3.641237	-2.178981	0.708294
C	-1.809153	-2.004862	2.694867
C	-4.308265	-0.870644	-1.554596
C	1.543833	-3.315129	-0.926263
C	5.072631	0.140320	0.341344
C	-4.047493	-2.264936	-2.153566
H	-4.013988	-3.033490	-1.381567
H	-4.839818	-2.527930	-2.857552
H	-3.096500	-2.284626	-2.688426
C	-4.405033	0.135258	-2.706572
H	-4.607188	1.145263	-2.346647
H	-3.490024	0.161643	-3.299714
H	-5.222234	-0.150534	-3.370469
C	-5.655696	-0.881467	-0.810960
H	-6.465634	-1.123955	-1.501980
H	-5.667967	-1.619422	-0.009146
H	-5.864767	0.096183	-0.372689
C	-1.870107	-3.490336	2.299140
H	-1.748927	-4.115340	3.185978
H	-2.822889	-3.751455	1.840288
H	-1.077495	-3.742762	1.596108
C	-0.492372	-1.762923	3.442543
H	0.372092	-1.920470	2.796565
H	-0.433778	-0.752423	3.848318
H	-0.416793	-2.457505	4.280480
C	-2.977068	-1.696281	3.650467
H	-3.938435	-1.893229	3.174719
H	-2.908959	-2.316148	4.547269
H	-2.961283	-0.649250	3.958489
C	2.434435	-4.048537	-1.945912
H	3.485383	-4.006365	-1.657754
H	2.145130	-5.099546	-2.016790
H	2.338426	-3.601639	-2.937019
C	1.703733	-3.984189	0.450161
H	1.124641	-3.457680	1.208294
H	1.351338	-5.016771	0.409232
H	2.743912	-4.001845	0.774432
C	0.081810	-3.459275	-1.364896
H	-0.595385	-2.932580	-0.692998
H	-0.077172	-3.076972	-2.373714
H	-0.197023	-4.514207	-1.364480
C	5.837445	-1.090123	0.840764
H	5.294035	-1.612774	1.629405
H	6.034958	-1.798959	0.035365
H	6.799265	-0.778248	1.250941
C	5.925604	0.833741	-0.736065
H	6.081149	0.173439	-1.591114
H	5.451084	1.745533	-1.098112
H	6.903721	1.100926	-0.330220
C	4.879721	1.101364	1.528616
H	4.381667	2.021059	1.222079
H	4.276984	0.635451	2.310521
H	5.846634	1.369883	1.959499

B-gauche-tBu

Number of imaginary frequencies: 0

Electronic Energy (SCF) = -1801.12499382 a.u

ZPV corrected Energy = -1800.343105 a.u.

Enthalpy = -1800.301860 a.u.

Free Energy = -1800.416549 a.u.

S	-1.053683	3.419842	-0.728837
C	-2.465842	3.570485	0.459755
H	-2.428801	2.725378	1.142348
H	-3.387965	3.521260	-0.112939
C	-0.057577	4.690902	0.140948
H	-0.050782	5.548357	-0.530071
H	0.946903	4.289474	0.204779
C	-0.754991	4.988460	1.459545
H	-0.462266	5.972196	1.826156
H	-0.464754	4.250257	2.209420
C	-2.254820	4.892811	1.189211
H	-2.845084	4.918096	2.104942
H	-2.574403	5.727590	0.562315
C	-0.307414	1.791878	-0.247656
C	0.686446	1.622262	-1.449429
O	1.168477	2.848117	-1.740780
H	0.082802	1.171957	-2.268096
C	1.705894	0.556826	-1.047868
C	2.964457	0.926673	-0.606614
C	1.361986	-0.797531	-1.080267
C	3.896274	-0.030144	-0.183501
H	3.204253	1.980519	-0.612188
C	2.251178	-1.777136	-0.658525
H	0.379950	-1.068655	-1.436352
C	3.517635	-1.368686	-0.213682
H	4.217935	-2.123099	0.108773
H	0.263809	2.008618	0.653739
C	-1.311603	0.720056	0.007345
C	-1.293323	0.045546	1.225615
C	-2.208203	0.327402	-0.985985
C	-2.148211	-1.029127	1.466259
H	-0.584509	0.363379	1.975033
C	-3.080373	-0.738174	-0.781171
H	-2.205646	0.854879	-1.927399
C	-3.028581	-1.397747	0.449060
H	-3.695043	-2.231190	0.616718
C	-2.150442	-1.802970	2.788537
C	-4.057145	-1.222966	-1.856593
C	1.894081	-3.267519	-0.660540
C	5.280377	0.424781	0.292488

C	-3.711829	-2.677186	-2.226536
H	-3.788724	-3.339403	-1.364178
H	-4.395898	-3.043262	-2.994720
H	-2.694410	-2.747004	-2.614699
C	-3.990213	-0.373135	-3.129746
H	-4.240195	0.670694	-2.933649
H	-3.000967	-0.406636	-3.588065
H	-4.706213	-0.754989	-3.858726
C	-5.495151	-1.164744	-1.311356
H	-6.199268	-1.508480	-2.071589
H	-5.619029	-1.796029	-0.431600
H	-5.765015	-0.143739	-1.035400
C	-1.865885	-3.290317	2.513264
H	-1.868399	-3.851797	3.449617
H	-2.616686	-3.730381	1.857384
H	-0.890641	-3.418901	2.042906
C	-1.090534	-1.285186	3.766495
H	-0.084025	-1.372030	3.355241
H	-1.261180	-0.241371	4.034074
H	-1.126512	-1.872625	4.684929
C	-3.532276	-1.665357	3.452861
H	-4.324075	-2.062093	2.817329
H	-3.552623	-2.213252	4.397077
H	-3.760308	-0.618604	3.662133
C	2.853012	-4.024729	-1.596392
H	3.890619	-3.916282	-1.280309
H	2.613429	-5.090357	-1.603559
H	2.771853	-3.651225	-2.618781
C	2.034256	-3.827233	0.766640
H	1.380800	-3.293075	1.457635
H	1.760048	-4.884095	0.787614
H	3.056399	-3.739225	1.134616
C	0.457948	-3.519660	-1.134577
H	-0.272937	-3.019132	-0.499336
H	0.305675	-3.179838	-2.159756
H	0.247392	-4.589938	-1.105281
C	6.166827	-0.746282	0.729689
H	5.727261	-1.298722	1.561616
H	6.345589	-1.446291	-0.087729
H	7.135423	-0.366506	1.058794
C	5.992782	1.168208	-0.851960
H	6.125066	0.513982	-1.715600
H	5.426653	2.041226	-1.175954
H	6.979124	1.506934	-0.527327
C	5.119158	1.378921	1.490036
H	4.537411	2.261187	1.223718
H	4.614311	0.878906	2.318877
H	6.097375	1.714625	1.841071

B-anti-0-tBu

Number of imaginary frequencies: 0

Electronic Energy (SCF) = -1801.12033939 a.u

ZPV corrected Energy = -1800.341504 a.u.

Enthalpy = -1800.298852 a.u.

Free Energy = -1800.414707 a.u.

S	3.421443	-0.965199	0.324212
C	4.815294	-2.166890	0.651490
H	4.505706	-3.093248	0.170715
H	4.869418	-2.317916	1.726886
C	4.440120	-0.036665	-0.925165
H	4.746314	0.888382	-0.437988
H	3.779121	0.192430	-1.754520
C	5.632647	-0.909788	-1.275388
H	6.433622	-0.316172	-1.716048
H	5.338459	-1.669134	-2.002620
C	6.070849	-1.580998	0.029313
H	6.814138	-2.359664	-0.143477
H	6.514526	-0.839362	0.696591
C	2.149521	-1.740322	-0.428760
H	2.374198	-2.350156	-1.293491
C	0.726502	0.011503	-2.907893
H	1.829406	0.043182	-2.924233
O	0.126036	-0.773044	-3.617079
C	0.104839	0.987679	-2.003017
C	0.931894	1.810719	-1.249392
C	-1.286038	1.100752	-1.909133
C	0.395260	2.752946	-0.370834
H	2.002664	1.703869	-1.353485
C	-1.854073	2.041290	-1.065538
H	-1.891595	0.442474	-2.511632
C	-0.991406	2.842543	-0.299176
H	-1.431924	3.560603	0.374119
C	0.829821	-1.791638	0.149540
C	-0.153709	-2.570138	-0.503893
C	0.439759	-1.125424	1.323517
C	-1.447405	-2.682786	-0.020695
H	0.134505	-3.082362	-1.409556
C	-0.852238	-1.224939	1.837504
H	1.169378	-0.527295	1.850492
C	-1.790030	-1.998934	1.155661
H	-2.794324	-2.084659	1.536739
C	-2.510884	-3.534745	-0.726555
C	-1.185988	-0.518142	3.159760
C	-3.367816	2.239141	-0.941662
C	1.326528	3.661356	0.437148
C	-3.665620	-2.628419	-1.186691
H	-4.442448	-3.220321	-1.676334
H	-4.120842	-2.102170	-0.348075
H	-3.306590	-1.884167	-1.897982
C	-3.056923	-4.592001	0.250246
H	-3.521566	-4.133636	1.122819
H	-3.809457	-5.210287	-0.244372
H	-2.256558	-5.246660	0.600343
C	-1.961962	-4.265020	-1.957171
H	-1.593528	-3.568081	-2.710367
H	-1.150067	-4.945177	-1.695282
H	-2.757595	-4.856833	-2.413180
C	-0.335578	-1.135755	4.285280
H	-0.559265	-0.653666	5.239930
H	-0.541738	-2.202932	4.386728
H	0.730064	-1.016677	4.088598
C	-2.662766	-0.658386	3.546633
H	-2.946574	-1.700685	3.697269
H	-2.844592	-0.128063	4.483053
H	-3.320776	-0.231431	2.789326
C	-0.861497	0.981161	3.053786
H	0.195482	1.148905	2.857003
H	-1.427964	1.444318	2.247708
H	-1.113217	1.489352	3.987480
C	-4.147045	1.352466	-1.917540
H	-5.215803	1.538660	-1.802162
H	-3.881776	1.563302	-2.954724
H	-3.972169	0.294385	-1.728410
C	-3.809875	1.890095	0.489878
H	-3.577455	0.850821	0.721250
H	-3.313098	2.519408	1.228113
H	-4.887272	2.032767	0.596047
C	-3.720361	3.707502	-1.240970
H	-4.799656	3.854267	-1.167399
H	-3.243297	4.392463	-0.540308
H	-3.408369	3.984360	-2.249642
C	0.570179	4.491265	1.479696
H	0.031991	3.856511	2.184794
H	1.280273	5.094502	2.047002
H	-0.144832	5.173068	1.017819
C	2.382807	2.815736	1.168313

H	1.914647	2.107326	1.850874
H	3.000801	2.251226	0.472370
H	3.042506	3.464051	1.748003
C	2.038484	4.621608	-0.533539
H	2.624661	4.074522	-1.272951
H	1.316196	5.241586	-1.067302
H	2.715163	5.280905	0.014164

B-anti-TS0-tBu
Number of imaginary frequencies: 1
Electronic Energy (SCF) = -1801.11035731 a.u
ZPV corrected Energy = -1800.330494 a.u.
Enthalpy = -1800.289410 a.u.
Free Energy = -1800.401436 a.u.

S	1.939749	-2.583797	-0.000289
C	1.473276	-3.957705	1.159892
H	0.398454	-4.079426	1.051371
H	1.701872	-3.632490	2.171021
C	2.835159	-3.697948	-1.146191
H	3.893342	-3.560047	-0.932278
H	2.628079	-3.368008	-2.159283
C	2.364193	-5.108863	-0.818339
H	3.068740	-5.838524	-1.216344
H	1.392101	-5.292391	-1.277646
C	2.257092	-5.184566	0.702816
H	1.751378	-6.091413	1.033557
H	3.253998	-5.175787	1.145894
C	0.503572	-2.146843	-0.940263
H	0.190944	-3.004857	-1.527602
C	0.952791	-0.948919	-2.426451
H	1.956375	-1.378235	-2.624296
O	0.047032	-1.110963	-3.302995
C	1.098267	0.382156	-1.728818
C	2.268091	0.708110	-1.059130
C	0.042523	1.293464	-1.748548
C	2.404417	1.923029	-0.380144
H	3.088933	0.001734	-1.070465
C	0.141738	2.517833	-1.099194
H	-0.843978	1.008265	-2.291720
C	1.330159	2.807832	-0.414439
H	1.411028	3.753880	0.097174
C	-0.573291	-1.551239	-0.135911
C	-1.876303	-1.592076	-0.641709
C	-0.345798	-0.864912	1.061708
C	-2.928577	-0.946692	0.004272
H	-2.048786	-2.125209	-1.564735
C	-1.376325	-0.229419	1.747616
H	0.656351	-0.822003	1.464402
C	-2.660085	-0.271273	1.197053
H	-3.466052	0.220920	1.712726
C	-4.339622	-1.028839	-0.591724
C	-1.086761	0.440254	3.097228
C	-0.987402	3.554755	-1.116092
C	3.714066	2.241095	0.350335
C	-5.354445	-0.184190	0.186571
H	-6.331860	-0.259257	-0.292500
H	-5.464912	-0.525520	1.216713
H	-5.072279	0.869516	0.204432
C	-4.807365	-2.495720	-0.564353
H	-4.825919	-2.878550	0.457824
H	-5.814733	-2.580536	-0.977763
H	-4.147489	-3.134113	-1.152176
C	-4.324080	-0.533775	-2.048541
H	-4.013095	0.509890	-2.101693
H	-3.643356	-1.115978	-2.668230
H	-5.323745	-0.612931	-2.480904
C	-0.788619	-0.660844	4.132372
H	-0.581154	-0.218996	5.109578
H	-1.638976	-1.337018	4.237928
H	0.078553	-1.253190	3.837771
C	-2.270643	1.272904	3.602381
H	-3.153382	0.659823	3.786932
H	-2.000945	1.752405	4.544603
H	-2.541150	2.056746	2.893790
C	0.136513	1.366129	2.989569
H	1.036297	0.819430	2.713983
H	-0.022953	2.141612	2.242502
H	0.323731	1.848352	3.951190
C	-2.212829	3.060927	-1.890769
H	-2.990083	3.826819	-1.871681
H	-1.977485	2.850202	-2.934645
H	-2.623927	2.155211	-1.446662
C	-1.431962	3.865198	0.323685
H	-1.813583	2.967425	0.809894
H	-0.611365	4.253644	0.926569
H	-2.227259	4.613904	0.318369
C	-0.480944	4.847973	-1.779748
H	-1.276133	5.596287	-1.806404
H	0.362854	5.274275	-1.236823
H	-0.159568	4.656971	-2.805247
C	3.653087	3.571482	1.108331
H	2.861078	3.571157	1.858754
H	4.600226	3.741036	1.622968
H	3.486204	4.414405	0.436560
C	4.026279	1.127642	1.366335
H	3.233658	1.048994	2.111489
H	4.130398	0.156963	0.882333
H	4.961590	1.344333	1.886763
C	4.858481	2.318906	-0.676322
H	4.976779	1.377350	-1.213049
H	4.667741	3.103066	-1.411087
H	5.803280	2.543145	-0.176160

B-anti-tBu
Number of imaginary frequencies: 0
Electronic Energy (SCF) = -1801.11454791 a.u
ZPV corrected Energy = -1800.333212 a.u.
Enthalpy = -1800.291867 a.u.
Free Energy = -1800.405992 a.u.

S	-0.153121	3.231938	-0.019310
C	1.174736	3.904341	1.071224
H	2.066984	3.319733	0.863306
H	0.870327	3.744289	2.101129
C	-0.165520	4.695991	-1.123674
H	-1.053921	5.263128	-0.853051
H	-0.264377	4.340941	-2.144917
C	1.120314	5.456575	-0.826251
H	1.031371	6.485542	-1.172828
H	1.956000	4.993473	-1.352246
C	1.331634	5.372170	0.682640
H	2.317886	5.725563	0.981603
H	0.585504	5.977390	1.199383
C	0.691209	1.981062	-1.075140
H	1.440559	2.546668	-1.624980
C	-0.305811	1.413038	-2.224410
H	-0.972696	2.284312	-2.450869
O	0.442577	1.003108	-3.236345
C	-1.227038	0.359995	-1.594527
C	-2.376050	0.715695	-0.906500
C	-0.886025	-0.987680	-1.698429
C	-3.185395	-0.245425	-0.287153
H	-2.652809	1.761800	-0.855619
C	-1.663780	-1.975100	-1.108101
H	0.003636	-1.221363	-2.261241

C	-2.807167	-1.579312	-0.398341
H	-3.413497	-2.340176	0.067566
C	1.326389	0.921646	-0.231861
C	2.475711	0.298528	-0.723532
C	0.767926	0.478159	0.960662
C	3.050536	-0.773585	-0.050144
H	2.884989	0.652111	-1.655568
C	1.332635	-0.576638	1.680434
H	-0.132370	0.946036	1.332064
C	2.463477	-1.191271	1.150259
H	2.911579	-2.016227	1.680777
C	4.291779	-1.503423	-0.574285
C	0.733649	-0.985011	3.031168
C	-1.318181	-3.465486	-1.206067
C	-4.443476	0.197167	0.468618
C	3.940108	-2.979165	-0.835076
H	4.821143	-3.519061	-1.188346
H	3.582159	-3.473492	0.068069
H	3.162865	-3.062310	-1.595537
C	5.414064	-1.427337	0.476564
H	5.120204	-1.896417	1.415515
H	6.306808	-1.939233	0.111594
H	5.679872	-0.389570	0.686453
C	4.814342	-0.898021	-1.881262
H	4.069258	-0.948262	-2.675986
H	5.108663	0.145062	-1.755299
H	5.693095	-1.453386	-2.212520
C	1.095072	0.096733	4.066491
H	0.685346	-0.161167	5.045572
H	2.177457	0.196237	4.165391
H	0.691664	1.067745	3.776196
C	1.275318	-2.332395	3.522302
H	2.348902	-2.299423	3.709609
H	0.787226	-2.599488	4.460713
H	1.077903	-3.128617	2.803088
C	-0.797380	-1.091336	2.937541
H	-1.257485	-0.138005	2.684766
H	-1.096346	-1.816572	2.182440
H	-1.205552	-1.408605	3.899013
C	-0.046936	-3.710829	-2.024942
H	0.160930	-4.781583	-2.064371
H	-0.149453	-3.352108	-3.049831
H	0.817282	-3.219400	-1.579005
C	-1.093981	-4.038658	0.204527
H	-0.260230	-3.536870	0.696322
H	-1.977023	-3.921867	0.832784
H	-0.862427	-5.104576	0.146066
C	-2.480480	-4.218259	-1.878370
H	-2.244824	-5.281498	-1.962337
H	-3.404554	-4.124080	-1.307649
H	-2.663719	-3.830405	-2.882035
C	-5.177910	-0.977572	1.123302
H	-4.545054	-1.497370	1.844206
H	-6.055078	-0.607296	1.656488
H	-5.521070	-1.703453	0.384986
C	-4.056801	1.193920	1.576220
H	-3.380887	0.729928	2.295856
H	-3.562370	2.076046	1.169944
H	-4.947882	1.526647	2.112881
C	-5.409567	0.885080	-0.512798
H	-4.954248	1.761627	-0.974081
H	-5.703678	0.200728	-1.310495
H	-6.313131	1.209431	0.008206

C-gauche-0-tBu
Number of imaginary frequencies: 0
Electronic Energy (SCF) = -1801.12364756 a.u
ZPV corrected Energy = -1800.344218 a.u.
Enthalpy = -1800.301692 a.u.
Free Energy = -1800.418389 a.u.

S	0.455995	-1.241478	2.734613
C	-0.417116	-2.839270	3.191751
H	-1.476949	-2.612453	3.262974
H	-0.039803	-3.093372	4.180452
C	1.248586	-2.019570	1.251179
H	2.218820	-1.547404	1.137107
H	0.631530	-1.784506	0.388986
C	1.300995	-3.506203	1.546847
H	2.099376	-3.718750	2.261362
H	1.509933	-4.074449	0.640584
C	-0.058995	-3.889401	2.148367
H	-0.810534	-3.904549	1.359070
H	-0.029774	-4.884142	2.592539
C	-0.567400	0.010736	2.338018
C	-0.253606	0.286659	-2.017924
H	-1.055224	0.923877	-1.613284
O	-0.489473	-0.508210	-2.908904
C	1.072587	0.498257	-1.417886
C	1.241929	1.547442	-0.517822
C	2.147435	-0.324989	-1.752321
C	2.492931	1.813435	0.036587
H	0.386091	2.160783	-0.272146
C	3.400007	-0.113604	-1.191197
H	1.976164	-1.132527	-2.449556
C	3.546120	0.963428	-0.307253
H	4.515494	1.145864	0.123739
H	-0.314419	0.910639	2.874449
C	-1.599059	0.046350	1.341441
C	-2.188511	1.293621	1.022670
C	-2.065714	-1.077770	0.644118
C	-3.172005	1.416715	0.057055
H	-1.831191	2.164644	1.551739
C	-3.071147	-0.989535	-0.320288
H	-1.652380	-2.046357	0.870364
C	-3.609445	0.259779	-0.611393
H	-4.373970	0.351140	-1.365260
C	4.561585	-1.047239	-1.548917
C	2.680303	3.025490	0.955122
C	-3.775064	2.773339	-0.328102
C	-3.571222	-2.276214	-0.990653
C	-2.388438	-3.078984	-1.560263
H	-1.843635	-2.494003	-2.300566
H	-1.686372	-3.374511	-0.780924
H	-2.753104	-3.990300	-2.039016
C	-4.295020	-3.131640	0.066003
H	-5.144609	-2.591747	0.488158
H	-4.667177	-4.055939	-0.381904
H	-3.626323	-3.398328	0.885036
C	-4.548309	-1.996111	-2.137740
H	-5.448903	-1.488033	-1.791002
H	-4.088091	-1.384697	-2.915232
H	-4.856310	-2.939222	-2.592188
C	-5.301842	2.735502	-0.137429
H	-5.740113	3.698615	-0.407800
H	-5.767119	1.970400	-0.758286
H	-5.557190	2.526255	0.903013
C	-3.457177	3.064890	-1.806392
H	-3.862630	2.296356	-2.464023
H	-3.885935	4.024382	-2.103939
H	-2.378888	3.111320	-1.969403
C	-3.218347	3.926325	0.514363
H	-3.424135	3.787892	1.576684
H	-2.140543	4.038121	0.388316
H	-3.685960	4.862048	0.203816
C	1.720894	2.930442	2.152674
H	1.840082	3.805156	2.794977

H	0.681020	2.887196	1.833191
H	1.927251	2.039708	2.746661
C	2.361017	4.302725	0.155819
H	3.022975	4.397993	-0.706662
H	1.332778	4.300041	-0.206367
H	2.493635	5.183998	0.786473
C	4.110597	3.136432	1.492925
H	4.390480	2.260987	2.081056
H	4.839635	3.256857	0.690424
H	4.185585	4.009955	2.141867
C	4.208409	-2.481433	-1.114601
H	3.292848	-2.830692	-1.591498
H	5.013884	-3.165492	-1.388793
H	4.069572	-2.536912	-0.034184
C	5.870821	-0.645465	-0.861986
H	6.190015	0.356795	-1.151511
H	5.785306	-0.676122	0.225182
H	6.659345	-1.341296	-1.151611
C	4.786270	-1.019694	-3.071300
H	3.901561	-1.350350	-3.614899
H	5.032943	-0.011609	-3.409003
H	5.612418	-1.680587	-3.340583

C-gauche-TS0-tBu
Number of imaginary frequencies: 1
Electronic Energy (SCF) = -1801.10712445 a.u
ZPV corrected Energy = -1800.327441 a.u.
Enthalpy = -1800.286253 a.u.
Free Energy = -1800.400554 a.u.

S	0.394845	-0.528137	1.547436
C	-0.836118	-0.717716	2.908796
H	-1.528280	0.117267	2.837730
H	-0.266532	-0.637948	3.833488
C	0.386459	-2.319564	1.121411
H	1.425971	-2.622312	1.049650
H	-0.064126	-2.404932	0.138979
C	-0.372116	-3.027424	2.241624
H	0.311909	-3.257427	3.060094
H	-0.777296	-3.969732	1.873973
C	-1.472803	-2.089899	2.736885
H	-2.276552	-2.031088	2.006345
H	-1.895057	-2.431508	3.681909
C	-0.335947	0.373432	0.220888
C	0.606655	-0.155341	-1.469324
H	0.181818	0.732766	-1.976412
O	0.261090	-1.305978	-1.869800
C	2.040177	0.100204	-1.052974
C	2.495530	1.395881	-0.822869
C	2.913678	-0.966671	-0.883418
C	3.795571	1.640626	-0.388494
H	1.810345	2.220259	-0.977248
C	4.224200	-0.766616	-0.448297
H	2.540504	-1.957141	-1.098718
C	4.641876	0.542430	-0.200257
H	5.648873	0.713944	0.139009
H	0.016085	1.388233	0.381760
C	-1.797604	0.309169	0.023365
C	-2.556858	1.477440	0.201575
C	-2.448576	-0.851836	-0.389444
C	-3.929403	1.492449	-0.007676
H	-2.041160	2.372555	0.516631
C	-3.829012	-0.874898	-0.606379
H	-1.864253	-1.735538	-0.579231
C	-4.547789	0.299152	-0.405572
H	-5.614028	0.300386	-0.566476
C	5.150268	-1.973938	-0.262507
C	4.249489	3.081977	-0.129672
C	-4.782381	2.749839	0.194158
C	-4.489593	-2.175167	-1.078431
C	-3.919515	-2.554774	-2.457201
H	-4.128234	-1.773751	-3.190371
H	-2.839933	-2.697385	-2.419258
H	-4.372176	-3.483753	-2.810580
C	-4.183712	-3.304287	-0.078374
H	-4.569155	-3.061985	0.913638
H	-4.653250	-4.233871	-0.406373
H	-3.112968	-3.485482	0.008214
C	-6.011395	-2.047830	-1.204055
H	-6.476245	-1.790320	-0.250966
H	-6.296232	-1.292634	-1.937711
H	-6.429549	-3.000852	-1.531523
C	-5.815210	2.490632	1.306162
H	-6.434027	3.376884	1.461413
H	-6.475449	1.660306	1.055706
H	-5.318689	2.253757	2.249028
C	-5.521317	3.084660	-1.114106
H	-6.184065	2.275739	-1.420851
H	-6.127522	3.983630	-0.984755
H	-4.812867	3.265652	-1.924335
C	-3.942613	3.967758	0.593744
H	-3.421663	3.809383	1.539148
H	-3.201177	4.214723	-0.167451
H	-4.594582	4.833908	0.716120
C	3.337175	3.719870	0.933986
H	3.647471	4.748072	1.131489
H	2.296783	3.740330	0.609875
H	3.387164	3.163458	1.871823
C	4.145540	3.890896	-1.435207
H	4.784579	3.461671	-2.208904
H	3.124165	3.909459	-1.815258
H	4.461060	4.922852	-1.267249
C	5.695774	3.162387	0.370651
H	5.829641	2.626274	1.311395
H	6.399720	2.755663	-0.356660
H	5.962768	4.206242	0.542608
C	4.516372	-2.955207	0.740307
H	3.558407	-3.330667	0.381537
H	5.173836	-3.813057	0.896263
H	4.352325	-2.473009	1.705922
C	6.534109	-1.579150	0.264089
H	7.054735	-0.911771	-0.423972
H	6.472365	-1.086384	1.235643
H	7.146210	-2.474422	0.383744
C	5.331837	-2.685299	-1.615417
H	4.378205	-3.026950	-2.017591
H	5.785383	-2.015121	-2.347802
H	5.982025	-3.555475	-1.501815

C-gauche-tBu
Number of imaginary frequencies: 0
Electronic Energy (SCF) = -1801.12192139 a.u
ZPV corrected Energy = -1800.339499 a.u.
Enthalpy = -1800.298494 a.u.
Free Energy = -1800.412413 a.u.

S	-0.040988	-1.822638	-1.279914
C	0.790201	-1.337432	-2.866460
H	0.779574	-0.255470	-2.961391
H	0.174687	-1.762828	-3.657734
C	1.244535	-3.051929	-0.872555
H	0.717778	-3.915205	-0.479686
H	1.852666	-2.623337	-0.083235
C	2.042783	-3.302800	-2.145921
H	1.515666	-4.008979	-2.789939
H	3.010286	-3.737245	-1.894179

C	2.183911	-1.954427	-2.850694
H	2.866960	-1.314572	-2.292780
H	2.571970	-2.061160	-3.863700
C	0.190738	-0.261636	-0.274699
C	-0.704940	-0.678843	0.934138
H	-0.319794	-0.109268	1.802251
O	-0.577888	-2.026081	1.064197
C	-2.132304	-0.170798	0.698369
C	-2.412385	1.193292	0.729441
C	-3.156301	-1.065343	0.428523
C	-3.696066	1.678005	0.486734
H	-1.607072	1.883781	0.950611
C	-4.458102	-0.624326	0.178038
H	-2.904031	-2.115635	0.423938
C	-4.705446	0.748861	0.210982
H	-5.703714	1.105157	0.022598
H	-0.235138	0.508312	-0.917868
C	1.617756	0.064409	0.019999
C	2.287167	1.000502	-0.774793
C	2.293475	-0.550581	1.067219
C	3.622036	1.311669	-0.548140
H	1.739692	1.478438	-1.573087
C	3.637132	-0.265941	1.323582
H	1.754142	-1.261589	1.675955
C	4.277443	0.659294	0.504594
H	5.314718	0.892144	0.685677
C	-5.554668	-1.650959	-0.126942
C	-3.958686	3.188269	0.529070
C	4.390942	2.323694	-1.403357
C	4.344925	-0.983411	2.476755
C	3.632559	-0.643490	3.798207
H	3.664030	0.430319	3.991129
H	2.587762	-0.953036	3.780950
H	4.120829	-1.153391	4.631124
C	4.276916	-2.503728	2.242821
H	4.763483	-2.774811	1.303900
H	4.783701	-3.032019	3.052757
H	3.247081	-2.858247	2.205689
C	5.817353	-0.579773	2.602279
H	6.381927	-0.820780	1.700192
H	5.929821	0.487107	2.800636
H	6.272423	-1.121031	3.432904
C	5.586289	1.620065	-2.071607
H	6.145901	2.329533	-2.684446
H	6.271192	1.201486	-1.334316
H	5.247506	0.807612	-2.716997
C	4.909228	3.462680	-0.507009
H	5.580429	3.091434	0.267318
H	5.458255	4.192535	-1.105323
H	4.081277	3.978212	-0.017281
C	3.518823	2.936329	-2.504199
H	3.145279	2.177948	-3.193952
H	2.663840	3.473827	-2.091634
H	4.110306	3.647452	-3.082424
C	-3.075444	3.889134	-0.519430
H	-3.249359	4.967173	-0.501711
H	-2.015845	3.717500	-0.330171
H	-3.302370	3.525189	-1.523301
C	-3.607037	3.729219	1.926748
H	-4.217714	3.248747	2.693210
H	-2.559826	3.553853	2.172725
H	-3.787765	4.805348	1.972677
C	-5.420854	3.539730	0.233844
H	-5.725385	3.207624	-0.759770
H	-6.099505	3.096814	0.964064
H	-5.549792	4.622435	0.275573
C	-5.165042	-2.449514	-1.384495
H	-4.220240	-2.975166	-1.247360
H	-5.933216	-3.190712	-1.615708
H	-5.060712	-1.788000	-2.246714
C	-6.919724	-1.000555	-0.375849
H	-7.266204	-0.440433	0.493925
H	-6.896832	-0.322745	-1.230544
H	-7.658482	-1.775391	-0.586999
C	-5.691745	-2.619104	1.061988
H	-4.758941	-3.147293	1.257805
H	-5.970672	-2.080929	1.969739
H	-6.464477	-3.363239	0.856863

C-gauche'-0-tBu
Number of imaginary frequencies: 0
Electronic Energy (SCF) = -1801.12350692 a.u
ZPV corrected Energy = -1800.344160 a.u.
Enthalpy = -1800.301567 a.u.
Free Energy = -1800.418389 a.u.

S	2.510586	-2.831262	-1.047698
C	4.273673	-2.408826	-1.535408
H	4.231738	-1.487473	-2.109122
H	4.585289	-3.222056	-2.187690
C	2.947404	-2.868383	0.755967
H	2.312531	-3.622356	1.213882
H	2.714692	-1.893087	1.175948
C	4.429046	-3.191198	0.804276
H	4.586947	-4.249808	0.586675
H	4.837462	-2.994096	1.795539
C	5.111033	-2.326406	-0.265779
H	5.161348	-1.295403	0.081816
H	6.131385	-2.660998	-0.451532
C	1.390555	-1.656989	-1.423836
C	-0.907181	-3.444753	-0.059721
H	-0.022191	-3.626671	0.573859
O	-1.198698	-4.222982	-0.948534
C	-1.680462	-2.249463	0.310004
C	-1.217748	-1.454739	1.353275
C	-2.869850	-1.930541	-0.347046
C	-1.935577	-0.335362	1.769767
H	-0.287623	-1.724083	1.835308
C	-3.609317	-0.818492	0.034666
H	-3.200335	-2.578177	-1.145313
C	-3.119402	-0.040286	1.093155
H	-3.684870	0.822923	1.399460
H	0.575870	-2.062013	-2.002562
C	1.423022	-0.250109	-1.136135
C	0.320955	0.540567	-1.527392
C	2.489967	0.401672	-0.487574
C	0.278987	1.908508	-1.299903
H	-0.503786	0.043267	-2.015291
C	2.472388	1.771795	-0.241608
H	3.344700	-0.175898	-0.181779
C	1.363431	2.511954	-0.653392
H	1.338644	3.574849	-0.463975
C	-0.914475	2.773384	-1.724305
C	3.627705	2.488803	0.469398
C	-1.418931	0.505401	2.940590
C	-4.955204	-0.510538	-0.632765
C	-2.000403	1.960163	-2.434458
H	-1.627095	1.495049	-3.347960
H	-2.394915	1.176475	-1.790348
H	-2.828058	2.616034	-2.709164
C	-0.434900	3.879502	-2.680305
H	-1.277987	4.499176	-2.993864
H	0.301418	4.529672	-2.208172
H	0.020632	3.448781	-3.573933
C	-1.544758	3.417998	-0.477111
H	-0.829504	4.040600	0.060192
H	-2.391706	4.046175	-0.762243

H	-1.906244	2.651634	0.207867
C	4.183070	3.597484	-0.442354
H	3.421818	4.339056	-0.682868
H	5.010778	4.112976	0.049740
H	4.552761	3.179056	-1.380307
C	3.111093	3.119440	1.775080
H	3.917479	3.654781	2.281348
H	2.303142	3.826131	1.587350
H	2.735117	2.352048	2.452865
C	4.780102	1.542863	0.822788
H	4.459720	0.749199	1.499038
H	5.210935	1.081094	-0.066463
H	5.571673	2.103580	1.322571
C	-0.016804	1.041781	2.604780
H	0.355019	1.653478	3.428812
H	0.693972	0.232722	2.440800
H	-0.034500	1.654066	1.704660
C	-2.330444	1.696018	3.254530
H	-3.335415	1.376074	3.534001
H	-1.915990	2.256302	4.093722
H	-2.411084	2.378713	2.408482
C	-1.334463	-0.382025	4.195957
H	-2.316322	-0.778770	4.460451
H	-0.659249	-1.224609	4.045865
H	-0.963028	0.199201	5.042326
C	-4.883009	-0.738855	-2.151591
H	-4.661140	-1.775628	-2.400671
H	-5.844242	-0.492074	-2.605407
H	-4.120563	-0.109163	-2.610277
C	-6.009982	-1.461547	-0.035708
H	-6.988299	-1.272683	-0.482989
H	-5.746601	-2.503840	-0.220454
H	-6.095253	-1.320323	1.043120
C	-5.405242	0.936317	-0.389857
H	-5.602805	1.134402	0.663718
H	-4.660045	1.651898	-0.738927
H	-6.331695	1.123027	-0.934563

C-gauche'-TS0-tBu
Number of imaginary frequencies: 1
Electronic Energy (SCF) = -1801.11022118 a.u
ZPV corrected Energy = -1800.329805 a.u.
Enthalpy = -1800.288814 a.u.
Free Energy = -1800.400383 a.u.

S	-2.319615	-3.069752	0.528498
C	-3.693653	-2.364685	1.542054
H	-3.306609	-1.500112	2.073837
H	-3.953591	-3.142778	2.256245
C	-3.280794	-3.034478	-1.036015
H	-2.989278	-3.912348	-1.604082
H	-3.010809	-2.142565	-1.590673
C	-4.736852	-3.025254	-0.589396
H	-5.038151	-4.025816	-0.273999
H	-5.377224	-2.732484	-1.420957
C	-4.839026	-2.048384	0.583218
H	-4.740330	-1.025562	0.223497
H	-5.795205	-2.135165	1.097863
C	-0.972088	-1.932407	0.438425
C	-0.068532	-2.498964	-1.241090
H	-0.807755	-2.018345	-1.912132
O	0.039370	-3.763438	-1.251914
C	1.149305	-1.619477	-1.110462
C	1.191412	-0.389723	-1.756803
C	2.221765	-2.010167	-0.315713
C	2.282314	0.468370	-1.614511
H	0.351144	-0.106212	-2.376371
C	3.322298	-1.176730	-0.130911
H	2.170938	-2.976093	0.167401
C	3.330648	0.057784	-0.788882
H	4.174666	0.712276	-0.655483
H	-0.248852	-2.332764	1.141846
C	-1.191068	-0.490579	0.609313
C	-0.344897	0.207162	1.481747
C	-2.135742	0.236030	-0.123840
C	-0.433218	1.586044	1.639033
H	0.392267	-0.358876	2.030097
C	-2.265163	1.615047	0.022099
H	-2.770680	-0.282014	-0.820828
C	-1.408576	2.268749	0.908733
H	-1.499277	3.338293	1.030490
C	0.485806	2.365028	2.587371
C	-3.319613	2.421933	-0.745042
C	2.315341	1.795013	-2.381967
C	4.470098	-1.628177	0.779957
C	1.509905	1.460821	3.281143
H	1.030231	0.696437	3.894029
H	2.157632	0.962999	2.559577
H	2.141423	2.062998	3.936163
C	-0.366169	3.054227	3.668045
H	0.273266	3.615501	4.352612
H	-1.082278	3.750204	3.230958
H	-0.923753	2.318346	4.250482
C	1.258175	3.432572	1.792343
H	0.586504	4.133449	1.297237
H	1.905387	4.003560	2.461419
H	1.883461	2.967982	1.029713
C	-4.319692	3.018054	0.262538
H	-3.823295	3.679901	0.972471
H	-5.085637	3.595780	-0.259214
H	-4.816597	2.228517	0.829432
C	-2.643836	3.564152	-1.523643
H	-3.396652	4.154814	-2.049578
H	-2.093615	4.234851	-0.864743
H	-1.944467	3.170313	-2.261461
C	-4.099293	1.563458	-1.747421
H	-3.439612	1.110158	-2.488887
H	-4.658368	0.767197	-1.254599
H	-4.817668	2.188758	-2.279471
C	0.994612	2.557036	-2.181896
H	1.027831	3.509229	-2.715345
H	0.142690	1.995892	-2.560585
H	0.815228	2.761793	-1.127457
C	3.463243	2.704867	-1.929724
H	4.439597	2.258424	-2.121233
H	3.418822	3.645967	-2.480163
H	3.396477	2.936732	-0.865700
C	2.495232	1.490217	-3.880776
H	3.430317	0.956593	-4.060221
H	1.678548	0.873743	-4.257912
H	2.515447	2.417303	-4.458311
C	3.921152	-1.924838	2.187463
H	3.166301	-2.710498	2.166844
H	4.728611	-2.252302	2.845853
H	3.468142	-1.033053	2.623518
C	5.099472	-2.909317	0.204030
H	5.916061	-3.251328	0.843703
H	4.368868	-3.714787	0.130403
H	5.502112	-2.728429	-0.794255
C	5.568852	-0.567979	0.911652
H	6.031212	-0.340232	-0.049788
H	5.185279	0.361856	1.334440
H	6.351645	-0.937006	1.576238

C-gauche'-tBu
Number of imaginary frequencies: 0

Electronic Energy (SCF) = -1801.12331135 a.u
ZPV corrected Energy = -1800.340754 a.u.
Enthalpy = -1800.299730 a.u.
Free Energy = -1800.413187 a.u.

S	0.917298	3.498198	0.512272
C	2.505367	3.229022	1.425059
H	2.413205	2.345568	2.051437
H	2.614936	4.103717	2.064562
C	1.767848	3.874132	-1.064057
H	1.253842	4.730024	-1.488306
H	1.625341	3.023081	-1.721746
C	3.236545	4.117669	-0.730439
H	3.372613	5.146622	-0.393317
H	3.846486	3.975968	-1.622699
C	3.618996	3.148068	0.387088
H	3.686471	2.134267	-0.003430
H	4.580636	3.402015	0.833247
C	0.230108	1.763564	0.381349
C	-0.744952	1.973467	-0.821751
H	-0.176310	1.692512	-1.733114
O	-1.133725	3.268409	-0.802147
C	-1.853199	0.925332	-0.689486
C	-1.626346	-0.401010	-1.044878
C	-3.086717	1.284120	-0.163611
C	-2.602546	-1.379379	-0.863930
H	-0.664747	-0.670556	-1.459216
C	-4.098267	0.339538	0.020591
H	-3.233653	2.324736	0.088403
C	-3.833432	-0.986645	-0.330415
H	-4.601210	-1.728137	-0.189704
H	-0.362230	1.733612	1.296410
C	1.196683	0.627522	0.375223
C	1.325254	-0.155972	1.521677
C	1.924715	0.288647	-0.766069
C	2.162223	-1.269883	1.548859
H	0.743592	0.112429	2.390799
C	2.774938	-0.812853	-0.776765
H	1.809756	0.882684	-1.656093
C	2.876189	-1.573202	0.389794
H	3.526663	-2.435808	0.392256
C	2.311821	-2.162182	2.784719
C	3.577341	-1.216663	-2.017394
C	-2.286266	-2.834276	-1.229828
C	-5.447807	0.782446	0.597752
C	1.441595	-1.688228	3.953207
H	1.708525	-0.680430	4.274898
H	0.381444	-1.695257	3.696522
H	1.582093	-2.356001	4.804234
C	3.781994	-2.155466	3.240494
H	3.906422	-2.789489	4.120578
H	4.445733	-2.529673	2.461016
H	4.103224	-1.145327	3.501141
C	1.896956	-3.600787	2.426671
H	2.516007	-4.011659	1.629388
H	1.999246	-4.249191	3.299091
H	0.857717	-3.634226	2.097017
C	5.078176	-1.219261	-1.676413
H	5.308109	-1.923215	-0.876785
H	5.662723	-1.504957	-2.553093
H	5.406383	-0.228115	-1.358025
C	3.153300	-2.629483	-2.457567
H	3.717474	-2.934004	-3.341285
H	3.333527	-3.365336	-1.673882
H	2.091307	-2.655792	-2.706728
C	3.352290	-0.258386	-3.191795
H	2.307237	-0.237425	-3.503653
H	3.656113	0.760753	-2.947211
H	3.946500	-0.585325	-4.046067
C	-1.110959	-3.322825	-0.362164
H	-0.868972	-4.361342	-0.598573
H	-0.216436	-2.723474	-0.525751
H	-1.363747	-3.265096	0.697782
C	-3.476575	-3.771160	-0.999090
H	-4.339764	-3.485281	-1.602067
H	-3.198210	-4.788591	-1.278555
H	-3.782883	-3.787544	0.047781
C	-1.886978	-2.920103	-2.713628
H	-2.703027	-2.583143	-3.355475
H	-1.013347	-2.306016	-2.930743
H	-1.647123	-3.951324	-2.981554
C	-5.228621	1.395226	1.993057
H	-4.565979	2.259376	1.950676
H	-6.180737	1.721347	2.417401
H	-4.786749	0.663997	2.672681
C	-6.072105	1.844069	-0.325849
H	-7.033002	2.175802	0.073719
H	-5.428131	2.717720	-0.423470
H	-6.241321	1.437557	-1.324649
C	-6.440073	-0.377391	0.734788
H	-6.660894	-0.837947	-0.229246
H	-6.067576	-1.153135	1.405546
H	-7.379236	-0.005417	1.147472

C-anti-0-tBu

Number of imaginary frequencies: 0
Electronic Energy (SCF) = -1801.12389415 a.u
ZPV corrected Energy = -1800.344631 a.u.
Enthalpy = -1800.302080 a.u.
Free Energy = -1800.418229 a.u.

S	-0.784139	1.772833	2.261689
C	-0.093933	3.519774	2.297066
H	0.967470	3.437424	2.512084
H	-0.590762	4.000889	3.137334
C	-1.481608	2.014740	0.562237
H	-2.393416	1.430296	0.515245
H	-0.758737	1.610996	-0.140533
C	-1.681600	3.509197	0.404649
H	-2.564168	3.830455	0.962423
H	-1.839716	3.771768	-0.641653
C	-0.426433	4.189895	0.970096
H	0.397475	4.065385	0.267290
H	-0.582255	5.260105	1.104475
C	0.371397	0.575736	2.308749
C	-1.463352	-2.432194	2.215432
O	-0.416902	-3.036954	2.341938
H	0.136583	-0.172403	3.049041
C	1.474082	0.365937	1.410627
C	2.153357	-0.869269	1.441201
C	1.933736	1.323795	0.488761
C	3.230309	-1.143638	0.609175
H	1.794884	-1.625159	2.125616
C	3.019164	1.085022	-0.349790
H	1.445901	2.283193	0.441938
C	3.659474	-0.154130	-0.284226
H	4.494843	-0.352725	-0.931925
H	-2.140865	-2.307194	3.081074
C	-1.959760	-1.837040	0.967370
C	-3.262700	-1.355306	0.939968
C	-1.146587	-1.757973	-0.168763
C	-3.798417	-0.808342	-0.228644
H	-3.856720	-1.413669	1.842628
C	-1.638330	-1.201271	-1.337113
H	-0.133465	-2.119331	-0.096563
C	-2.969055	-0.748294	-1.343426
H	-3.355701	-0.325254	-2.256968

C	-5.238044	-0.286373	-0.234573
C	-0.795177	-1.065322	-2.607347
C	3.489078	2.199300	-1.295331
C	3.898682	-2.523667	0.680714
C	-5.370988	0.844158	0.802182
H	-6.399612	1.208703	0.829535
H	-5.108457	0.505369	1.804444
H	-4.721566	1.683187	0.547951
C	-6.196085	-1.431823	0.138240
H	-7.227503	-1.074252	0.136438
H	-6.118595	-2.251291	-0.578337
H	-5.981742	-1.829681	1.130140
C	-5.652921	0.264617	-1.602371
H	-5.027890	1.105275	-1.907262
H	-5.601387	-0.499340	-2.379483
H	-6.683245	0.619070	-1.552642
C	-1.400184	-1.954036	-3.708780
H	-2.426560	-1.665458	-3.938236
H	-0.813556	-1.869080	-4.625754
H	-1.403942	-3.001693	-3.403107
C	0.658241	-1.488602	-2.375203
H	1.129826	-0.901537	-1.588214
H	0.732239	-2.541623	-2.102952
H	1.231586	-1.342328	-3.291690
C	-0.803082	0.399793	-3.078162
H	-0.198667	0.504061	-3.980783
H	-1.809961	0.746283	-3.310456
H	-0.386114	1.055510	-2.314459
C	2.867589	-3.605850	0.311483
H	3.321120	-4.597666	0.376013
H	2.005886	-3.583208	0.977403
H	2.508377	-3.463688	-0.708895
C	5.088790	-2.651677	-0.276680
H	5.525429	-3.647328	-0.182008
H	4.788270	-2.519875	-1.317020
H	5.870031	-1.923702	-0.052749
C	4.406210	-2.778031	2.111337
H	5.134324	-2.019614	2.405364
H	3.591907	-2.761346	2.835133
H	4.889893	-3.755431	2.172912
C	2.330149	2.635030	-2.208429
H	1.483661	3.010334	-1.633910
H	2.657727	3.432609	-2.878872
H	1.981924	1.800849	-2.817922
C	4.655108	1.760173	-2.187884
H	5.532706	1.482776	-1.602723
H	4.383631	0.911842	-2.817838
H	4.941282	2.584068	-2.843742
C	3.950861	3.407719	-0.460105
H	4.772760	3.130092	0.202231
H	4.296902	4.211819	-1.113521
H	3.141583	3.799515	0.156209

C-anti-TS0-tBu
Number of imaginary frequencies: 1
Electronic Energy (SCF) = -1801.10597729 a.u
ZPV corrected Energy = -1800.325860 a.u.
Enthalpy = -1800.284837 a.u.
Free Energy = -1800.398213 a.u.

S	-0.226407	-2.291260	0.344317
C	1.039290	-2.928989	1.533540
H	1.926406	-3.187877	0.963038
H	0.607820	-3.832608	1.961859
C	-0.742282	-0.925633	1.454877
H	-1.825835	-0.886749	1.442698
H	-0.345546	-0.011721	1.030493
C	-0.135777	-1.227060	2.820664
H	-0.765407	-1.932868	3.364979
H	-0.080037	-0.308555	3.404386
C	1.242645	-1.837498	2.573634
H	1.925431	-1.081283	2.187371
H	1.674478	-2.254375	3.483266
C	0.601286	-1.553779	-1.055869
C	-0.617948	-0.726795	-2.362233
O	0.034288	0.140040	-3.020346
H	0.904736	-2.430428	-1.626770
C	1.765638	-0.700168	-0.710491
C	3.056760	-1.232191	-0.723039
C	1.599744	0.637848	-0.355074
C	4.164207	-0.476147	-0.343216
H	3.184811	-2.263234	-1.024365
C	2.678653	1.424936	0.041077
H	0.613890	1.067823	-0.409832
C	3.949780	0.847603	0.048514
H	4.792105	1.444043	0.354264
H	-0.833907	-1.702592	-2.842770
C	-1.824392	-0.294649	-1.552626
C	-2.797919	-1.217506	-1.198545
C	-1.969061	1.036114	-1.158446
C	-3.892812	-0.856011	-0.406265
H	-2.690139	-2.239709	-1.539221
C	-3.042319	1.438629	-0.373851
H	-1.217618	1.735497	-1.489197
C	-3.986179	0.470787	0.002335
H	-4.817667	0.774517	0.618219
C	-4.918419	-1.919614	-0.000804
C	-3.220872	2.883010	0.106165
C	2.430743	2.883385	0.439746
C	5.556483	-1.117514	-0.350970
C	-4.212954	-3.004787	0.833495
H	-4.926274	-3.775799	1.132097
H	-3.413266	-3.486245	0.271348
H	-3.778546	-2.577568	1.739430
C	-5.519887	-2.562654	-1.262802
H	-6.248427	-3.327393	-0.985711
H	-6.027586	-1.814569	-1.874098
H	-4.754808	-3.035979	-1.877675
C	-6.064913	-1.341273	0.835270
H	-5.707214	-0.906118	1.769625
H	-6.614988	-0.573026	0.290042
H	-6.767102	-2.137004	1.088440
C	-4.594885	3.413217	-0.340606
H	-5.411698	2.820508	0.070678
H	-4.727547	4.443908	-0.005286
H	-4.680697	3.396438	-1.428489
C	-2.143124	3.817854	-0.451538
H	-1.145884	3.526875	-0.124829
H	-2.150061	3.837980	-1.542010
H	-2.323418	4.833925	-0.096967
C	-3.139937	2.916348	1.643741
H	-3.261906	3.938753	2.007625
H	-3.916935	2.302696	2.100065
H	-2.172428	2.547827	1.989731
C	5.891833	-1.589699	-1.776971
H	6.878870	-2.056417	-1.798217
H	5.167207	-2.319012	-2.138901
H	5.897674	-0.748495	-2.472100
C	6.651579	-0.146749	0.103689
H	7.617998	-0.652268	0.076849
H	6.715097	0.725706	-0.547960
H	6.487649	0.201016	1.124761
C	5.564328	-2.329834	0.598332
H	5.315082	-2.027256	1.617155
H	4.846363	-3.088217	0.286228
H	6.553568	-2.791904	0.613470
C	1.894979	3.652095	-0.781584

H	0.978696	3.206523	-1.165820
H	1.681146	4.689072	-0.514047
H	2.629494	3.652097	-1.588614
C	3.701596	3.583034	0.932044
H	4.124775	3.085993	1.806558
H	4.468712	3.622903	0.157689
H	3.464363	4.609685	1.215140
C	1.387436	2.930812	1.571203
H	1.744638	2.391222	2.450583
H	1.191115	3.964434	1.862661
H	0.441221	2.486938	1.265436

C-anti-tBu
Number of imaginary frequencies: 0
Electronic Energy (SCF) = -1801.11053503 a.u
ZPV corrected Energy = -1800.328350 a.u.
Enthalpy = -1800.287369 a.u.
Free Energy = -1800.399155 a.u.

S	0.076521	-2.480321	-0.119915
C	-1.368595	-3.163659	-1.027143
H	-2.185294	-3.290437	-0.323079
H	-1.046865	-4.141473	-1.382029
C	0.522218	-1.303081	-1.452628
H	1.595798	-1.364712	-1.590101
H	0.261051	-0.310662	-1.102077
C	-0.293387	-1.717812	-2.674423
H	0.217563	-2.521205	-3.206982
H	-0.385279	-0.870407	-3.353053
C	-1.652271	-2.200716	-2.173160
H	-2.242503	-1.360358	-1.812268
H	-2.219399	-2.706750	-2.953829
C	-0.620541	-1.462826	1.299986
C	0.552089	-0.871185	2.248895
O	0.009101	0.004180	3.077767
H	-1.070242	-2.266896	1.882575
C	-1.709586	-0.550283	0.830916
C	-3.025036	-1.008563	0.869378
C	-1.453003	0.714062	0.307272
C	-4.078224	-0.255519	0.353778
H	-3.220989	-1.978624	1.305332
C	-2.476872	1.498924	-0.218285
H	-0.443929	1.088836	0.326739
C	-3.778086	0.990863	-0.196131
H	-4.576589	1.585769	-0.604977
H	0.918646	-1.814163	2.729101
C	1.771985	-0.381564	1.442344
C	2.733701	-1.282129	1.006156
C	1.948266	0.975777	1.172422
C	3.831203	-0.875301	0.238714
H	2.627622	-2.325566	1.275103
C	3.033052	1.427850	0.428788
H	1.221414	1.656708	1.586622
C	3.952048	0.479815	-0.046575
H	4.791463	0.823390	-0.630112
C	4.841774	-1.918570	-0.249456
C	3.269071	2.910035	0.116910
C	-2.152747	2.893708	-0.762650
C	-5.502384	-0.819000	0.396848
C	4.114794	-2.966160	-1.112533
H	4.821248	-3.719037	-1.468179
H	3.334740	-3.478951	-0.550257
H	3.652260	-2.499102	-1.984056
C	5.478744	-2.619121	0.963960
H	6.198276	-3.370620	0.632092
H	6.004029	-1.899647	1.594359
H	4.729597	-3.119245	1.577544
C	5.962688	-1.300357	-1.091405
H	5.573872	-0.807132	-1.983648
H	6.540349	-0.570236	-0.523070
H	6.647759	-2.084525	-1.417345
C	4.642355	3.337958	0.665735
H	5.454085	2.767917	0.214135
H	4.818430	4.395310	0.456903
H	4.688960	3.192927	1.746427
C	2.201936	3.812362	0.742706
H	1.208809	3.587456	0.358362
H	2.175394	3.715211	1.828645
H	2.420845	4.854771	0.505498
C	3.246050	3.121324	-1.408183
H	3.411836	4.174109	-1.646638
H	4.021390	2.539025	-1.906321
H	2.281696	2.827934	-1.826455
C	-5.903258	-1.078622	1.859975
H	-6.916125	-1.483809	1.907060
H	-5.233413	-1.792074	2.339825
H	-5.878893	-0.153649	2.438385
C	-6.528307	0.136404	-0.221360
H	-7.521343	-0.311591	-0.163917
H	-6.563451	1.089755	0.307492
H	-6.315034	0.336260	-1.272526
C	-5.547852	-2.145358	-0.383773
H	-5.268779	-1.991967	-1.427782
H	-4.870468	-2.885236	0.042600
H	-6.556618	-2.562417	-0.360194
C	-1.781374	3.801950	0.423739
H	-0.949942	3.390630	0.994108
H	-1.495605	4.795027	0.070462
H	-2.628765	3.911266	1.102704
C	-3.335614	3.524616	-1.505507
H	-3.659610	2.908369	-2.346073
H	-4.191405	3.685036	-0.849225
H	-3.038042	4.497470	-1.899622
C	-0.966566	2.812797	-1.739723
H	-1.204789	2.165167	-2.585893
H	-0.731725	3.805444	-2.128002
H	-0.068806	2.426702	-1.261403

D-gauche-0-tBu
Number of imaginary frequencies: 0
Electronic Energy (SCF) = -1801.12021759 a.u
ZPV corrected Energy = -1800.341356 a.u.
Enthalpy = -1800.298587 a.u.
Free Energy = -1800.418321 a.u.

S	0.484172	-1.078608	1.345511
C	-0.187369	-2.191344	2.671770
H	-1.258606	-2.017709	2.739255
H	0.288269	-1.888458	3.601196
C	0.374317	-2.433101	0.056962
H	1.393532	-2.567250	-0.291653
H	-0.231253	-2.052939	-0.759390
C	-0.175480	-3.684924	0.727771
H	0.252925	-4.581208	0.279512
H	-1.256026	-3.738064	0.595115
C	0.154076	-3.600284	2.224716
H	-0.405331	-4.337902	2.800211
H	1.217139	-3.794603	2.383730
C	-0.496695	0.231165	1.027192
C	0.664745	0.633993	-1.989101
H	0.053550	1.526943	-1.771405
O	0.229772	-0.246819	-2.705890
C	1.996055	0.633248	-1.360657
C	2.341688	1.701392	-0.537655
C	2.877363	-0.431457	-1.538677
C	3.555763	1.715080	0.142012

H	1.632316	2.508728	-0.414975
C	4.102565	-0.450189	-0.881826
H	2.578553	-1.244211	-2.184512
C	4.414127	0.628924	-0.046257
H	5.354836	0.619492	0.476830
H	0.058284	1.153813	1.071817
C	-1.870568	0.252930	0.613929
C	-2.462648	1.505063	0.315859
C	-2.672156	-0.886728	0.454979
C	-3.770908	1.615370	-0.120495
H	-1.851699	2.387727	0.436189
C	-3.994462	-0.808175	0.012445
H	-2.259483	-1.856846	0.676671
C	-4.532557	0.442995	-0.270170
H	-5.550841	0.523485	-0.613707
C	5.051981	-1.636976	-1.076692
C	3.888922	2.880784	1.077543
C	-4.796561	-2.105208	-0.155707
C	-4.414333	2.967949	-0.450804
C	4.344376	-2.929494	-0.631372
H	3.448582	-3.119390	-1.221954
H	5.012600	-3.784233	-0.752670
H	4.053415	-2.871687	0.418990
C	5.421964	-1.750315	-2.566311
H	4.539728	-1.902156	-3.188075
H	5.926667	-0.846084	-2.910944
H	6.094156	-2.596088	-2.723648
C	6.344916	-1.493121	-0.267293
H	6.149076	-1.441019	0.804768
H	6.981329	-2.360758	-0.446121
H	6.907642	-0.604177	-0.555621
C	2.829575	2.943367	2.193561
H	3.054713	3.761617	2.880370
H	1.830822	3.109984	1.790445
H	2.811046	2.013581	2.764629
C	3.866206	4.198080	0.282136
H	4.607747	4.181573	-0.518476
H	2.889536	4.380906	-0.166030
H	4.095018	5.038244	0.940590
C	5.267961	2.733273	1.728356
H	5.333178	1.833001	2.341030
H	6.065531	2.699322	0.984633
H	5.455282	3.589582	2.377471
C	-4.867241	-2.846052	1.191764
H	-3.874940	-3.104220	1.561116
H	-5.357480	-2.230245	1.947976
H	-5.436682	-3.772007	1.085679
C	-6.229562	-1.852164	-0.636368
H	-6.248753	-1.359528	-1.609373
H	-6.753415	-2.804210	-0.736103
H	-6.790229	-1.236733	0.068603
C	-4.096372	-3.006050	-1.189858
H	-4.022179	-2.502373	-2.155198
H	-3.088115	-3.272549	-0.873401
H	-4.660128	-3.931003	-1.330246
C	-5.642537	3.183897	0.451941
H	-6.114315	4.142886	0.226633
H	-6.387780	2.401577	0.309563
H	-5.353966	3.186307	1.504680
C	-4.863130	2.974837	-1.923538
H	-5.595752	2.193620	-2.124675
H	-5.318346	3.935166	-2.175586
H	-4.012107	2.816914	-2.588581
C	-3.454892	4.144373	-0.240668
H	-3.118678	4.213436	0.794812
H	-2.574758	4.067894	-0.880722
H	-3.964320	5.077307	-0.487412

D-gauche-TS0-tBu
Number of imaginary frequencies: 1
Electronic Energy (SCF) = -1801.10863024 a.u
ZPV corrected Energy = -1800.328514 a.u.
Enthalpy = -1800.287531 a.u.
Free Energy = -1800.401041 a.u.

S	0.417793	-0.602379	1.501422
C	-0.745257	-0.808784	2.916134
H	-1.723550	-0.481621	2.569971
H	-0.403070	-0.163505	3.720689
C	0.356103	-2.384482	1.034035
H	1.349145	-2.761884	1.266160
H	0.198589	-2.424645	-0.041583
C	-0.715674	-3.031668	1.901976
H	-0.507592	-4.094531	2.022212
H	-1.690227	-2.930234	1.429275
C	-0.719962	-2.295956	3.242817
H	-1.586568	-2.559186	3.848960
H	0.176743	-2.541843	3.814502
C	-0.340080	0.351860	0.229965
C	0.577724	-0.136006	-1.477870
H	0.142805	0.756195	-1.967387
O	0.234952	-1.283532	-1.895742
C	2.014007	0.118905	-1.072920
C	2.469736	1.414797	-0.845732
C	2.886364	-0.948344	-0.899041
C	3.770028	1.660042	-0.412179
H	1.784174	2.238727	-1.000920
C	4.196107	-0.747815	-0.461994
H	2.513807	-1.939932	-1.110232
C	4.615079	0.561722	-0.219190
H	5.621871	0.733369	0.120623
H	0.021186	1.359534	0.412845
C	-1.803063	0.298166	0.047349
C	-2.552622	1.468056	0.251163
C	-2.461702	-0.852529	-0.381874
C	-3.925478	1.496406	0.047121
H	-2.029360	2.353234	0.581335
C	-3.842571	-0.859125	-0.602914
H	-1.880319	-1.734632	-0.590341
C	-4.551881	0.316328	-0.376166
H	-5.617870	0.329494	-0.537476
C	5.117602	-1.956266	-0.262566
C	4.225141	3.101855	-0.158368
C	-4.515671	-2.132917	-1.127608
C	-4.769658	2.754846	0.276716
C	4.481265	-2.917686	0.757978
H	3.516771	-3.287362	0.411093
H	5.131407	-3.779659	0.921824
H	4.328348	-2.420039	1.717572
C	5.291163	-2.689467	-1.604721
H	4.335007	-3.033484	-1.998680
H	5.745212	-2.032858	-2.348996
H	5.937981	-3.560538	-1.479568
C	6.505185	-1.560279	0.253022
H	6.449757	-1.054644	1.218282
H	7.114183	-2.456391	0.381926
H	7.025361	-0.904001	-0.446030
C	3.313651	3.744020	0.903439
H	3.624978	4.772571	1.097419
H	2.273193	3.764426	0.579564
H	3.363465	3.190678	1.843101
C	4.121244	3.906378	-1.466623
H	4.759426	3.473921	-2.239224
H	3.099656	3.924653	-1.846099
H	4.437882	4.938568	-1.302292
C	5.671662	3.182816	0.341160
H	5.805389	2.650146	1.283876

H	6.374974	2.772736	-0.384876
H	5.939613	4.227069	0.509127
C	-4.183999	-3.320996	-0.208661
H	-3.114536	-3.524414	-0.184867
H	-4.517283	-3.130800	0.813345
H	-4.685068	-4.222598	-0.566509
C	-6.040605	-2.001049	-1.204954
H	-6.346777	-1.211344	-1.891970
H	-6.467255	-2.937242	-1.568010
H	-6.478562	-1.792036	-0.227443
C	-3.981465	-2.428126	-2.541368
H	-4.214978	-1.608006	-3.222531
H	-2.900153	-2.563150	-2.538681
H	-4.437237	-3.339049	-2.935662
C	-5.809046	2.476474	1.377839
H	-6.421432	3.363558	1.552497
H	-6.474810	1.657938	1.104393
H	-5.318233	2.212501	2.316484
C	-5.500223	3.127081	-1.026169
H	-6.166803	2.330465	-1.355952
H	-6.100971	4.026698	-0.877287
H	-4.786721	3.323261	-1.828391
C	-3.922320	3.956068	0.709539
H	-3.404549	3.769297	1.651507
H	-3.177469	4.217649	-0.043380
H	-4.568519	4.823307	0.852986

D-gauche-tBu

Number of imaginary frequencies: 0
Electronic Energy (SCF) = -1801.12163776 a.u
ZPV corrected Energy = -1800.339589 a.u.
Enthalpy = -1800.298447 a.u.
Free Energy = -1800.413592 a.u.

S	-0.049313	-1.715116	-1.317903
C	0.673720	-1.139215	-2.920055
H	1.175703	-0.189882	-2.757480
H	-0.147563	-0.997037	-3.618213
C	1.312350	-2.898561	-1.011356
H	0.841852	-3.875521	-1.111396
H	1.627510	-2.768713	0.014993
C	2.384785	-2.652970	-2.062961
H	2.977060	-3.555571	-2.212414
H	3.053597	-1.860714	-1.726228
C	1.663905	-2.220887	-3.339010
H	2.351829	-1.836087	-4.091579
H	1.133715	-3.069884	-3.774740
C	0.193553	-0.203012	-0.249977
C	-0.694750	-0.655653	0.955180
H	-0.318793	-0.081647	1.825731
O	-0.548097	-1.998720	1.078323
C	-2.130446	-0.168291	0.720623
C	-2.434934	1.190292	0.761201
C	-3.137888	-1.078723	0.441667
C	-3.726604	1.653899	0.519425
H	-1.642521	1.893426	0.989171
C	-4.446737	-0.659205	0.191245
H	-2.865661	-2.124007	0.429994
C	-4.718646	0.709054	0.234183
H	-5.722664	1.048996	0.046113
H	-0.227830	0.600093	-0.854967
C	1.630305	0.086295	0.045773
C	2.311805	1.045258	-0.711434
C	2.302501	-0.585649	1.059739
C	3.656095	1.316933	-0.486608
H	1.765961	1.578428	-1.475187
C	3.653735	-0.341086	1.313550
H	1.752311	-1.309881	1.642404
C	4.307916	0.603088	0.526963
H	5.352678	0.802696	0.705146
C	-5.523986	-1.702903	-0.124264
C	-4.016310	3.158880	0.572571
C	4.356167	-1.122523	2.427854
C	4.438100	2.348612	-1.305753
C	-5.116019	-2.486428	-1.385427
H	-4.162412	-2.995926	-1.248340
H	-5.869979	-3.239849	-1.623822
H	-5.020882	-1.817750	-2.243192
C	-5.647918	-2.680892	1.058044
H	-4.706469	-3.193536	1.253803
H	-5.939447	-2.153486	1.968152
H	-6.406541	-3.437383	0.845561
C	-6.899384	-1.075052	-0.373985
H	-6.885190	-0.390950	-1.223857
H	-7.623510	-1.861317	-0.593437
H	-7.259102	-0.527254	0.498250
C	-3.142222	3.883600	-0.467358
H	-3.335184	4.958233	-0.441722
H	-2.080343	3.729206	-0.275961
H	-3.359467	3.523729	-1.474848
C	-3.679217	3.695050	1.975674
H	-4.284472	3.198298	2.736037
H	-2.630117	3.535706	2.224524
H	-3.878543	4.767547	2.029096
C	-5.483521	3.486551	0.274838
H	-5.778821	3.156213	-0.722156
H	-6.156597	3.026530	0.999603
H	-5.631727	4.566483	0.323798
C	4.257759	-2.629095	2.126178
H	3.221015	-2.962104	2.082897
H	4.730618	-2.866287	1.171275
H	4.761300	-3.202876	2.906698
C	5.836754	-0.752290	2.559781
H	5.970636	0.302835	2.802733
H	6.286843	-1.337245	3.363039
H	6.390929	-0.964752	1.644171
C	3.659721	-0.829277	3.768783
H	3.712844	0.234077	4.009174
H	2.609148	-1.118155	3.745893
H	4.144158	-1.384884	4.574249
C	5.596264	1.642937	-2.034684
H	6.165512	2.364071	-2.624620
H	6.282198	1.167338	-1.333849
H	5.217894	0.874073	-2.710917
C	5.010632	3.424471	-0.365559
H	5.686328	2.995020	0.373844
H	5.569256	4.166829	-0.939072
H	4.209721	3.939277	0.167965
C	3.563413	3.043597	-2.354497
H	3.153780	2.334466	-3.075391
H	2.733243	3.582939	-1.896184
H	4.164593	3.766993	-2.906922

D-gauche'-0-tBu

Number of imaginary frequencies: 0
Electronic Energy (SCF) = -1801.12314943 a.u
ZPV corrected Energy = -1800.343897 a.u.
Enthalpy = -1800.301341 a.u.
Free Energy = -1800.417650 a.u.

S	2.207436	-2.874562	-1.074857
C	3.939490	-2.611206	-1.690109
H	4.095461	-1.540920	-1.801470
H	3.995297	-3.089678	-2.664564
C	2.781975	-2.961018	0.709075
H	2.471935	-3.943435	1.058604

H	2.236023	-2.197993	1.255618
C	4.295380	-2.788200	0.729344
H	4.741244	-3.367470	1.537591
H	4.547328	-1.742093	0.897058
C	4.838474	-3.230785	-0.636976
H	5.873254	-2.916995	-0.774855
H	4.810411	-4.319802	-0.715764
C	1.180055	-1.602917	-1.390947
C	-1.050223	-3.511989	0.150280
H	-0.172539	-3.687230	0.797187
O	-1.348185	-4.317202	-0.711255
C	-1.796478	-2.284078	0.460893
C	-1.331925	-1.462801	1.484332
C	-2.948549	-1.944535	-0.247276
C	-2.008140	-0.293598	1.822672
H	-0.427386	-1.747556	2.004920
C	-3.650713	-0.786455	0.063193
H	-3.278810	-2.603477	-1.037338
C	-3.162044	0.016698	1.100660
H	-3.694149	0.919519	1.344768
H	0.287077	-1.935672	-1.896071
C	1.348913	-0.206347	-1.096499
C	0.358932	0.702631	-1.512821
C	2.458753	0.323333	-0.401612
C	0.466657	2.072880	-1.287928
H	-0.506835	0.308402	-2.028564
C	2.588971	1.682613	-0.154659
H	3.223681	-0.348963	-0.054775
C	1.587742	2.550307	-0.610025
H	1.689836	3.606704	-0.423285
C	-0.646285	3.007555	-1.781460
C	3.789117	2.265322	0.602706
C	-4.918881	-0.432466	-0.721055
C	-1.471737	0.598587	2.945559
C	-0.841283	2.825429	-3.297301
H	-1.626038	3.493081	-3.659653
H	0.078316	3.057481	-3.837790
H	-1.129106	1.804726	-3.547077
C	-0.335496	4.484761	-1.516407
H	-1.153991	5.100713	-1.892681
H	-0.229249	4.691967	-0.450738
H	0.578946	4.803629	-2.018868
C	-1.957177	2.655753	-1.058926
H	-2.233404	1.616580	-1.221053
H	-1.859477	2.809735	0.016224
H	-2.771970	3.286570	-1.421155
C	4.520977	3.277933	-0.296625
H	5.380586	3.699102	0.229338
H	4.882250	2.796633	-1.207351
H	3.869723	4.101474	-0.588254
C	3.294087	2.981239	1.872476
H	4.136996	3.416342	2.413967
H	2.596119	3.783741	1.635536
H	2.787439	2.281097	2.538051
C	4.796388	1.191635	1.027083
H	4.346063	0.457596	1.696253
H	5.210928	0.662730	0.167898
H	5.625614	1.660341	1.559460
C	-0.047777	1.061575	2.589833
H	0.340876	1.708783	3.378188
H	0.634500	0.219531	2.481084
H	-0.038202	1.619329	1.654115
C	-2.339316	1.841458	3.168643
H	-1.913296	2.439331	3.975459
H	-2.382332	2.469125	2.277856
H	-3.359707	1.579819	3.452871
C	-1.431581	-0.206383	4.256781
H	-2.430678	-0.544918	4.537261
H	-0.790582	-1.083816	4.169415
H	-1.042761	0.413869	5.066855
C	-4.584944	-0.337007	-2.220769
H	-4.194476	-1.278058	-2.607329
H	-5.483734	-0.088989	-2.788683
H	-3.841495	0.438954	-2.406498
C	-5.966084	-1.540268	-0.506565
H	-6.878552	-1.308183	-1.059483
H	-5.600700	-2.507756	-0.851129
H	-6.222740	-1.632786	0.550281
C	-5.528575	0.901752	-0.277619
H	-5.830826	0.881610	0.770442
H	-4.834482	1.731045	-0.417608
H	-6.418105	1.108387	-0.874198

D-gauche'-TS0-tBu
Number of imaginary frequencies: 1
Electronic Energy (SCF) = -1801.10901442 a.u
ZPV corrected Energy = -1800.329148 a.u.
Enthalpy = -1800.287951 a.u.
Free Energy = -1800.402436 a.u.

S	1.337863	3.381973	0.604404
C	2.869679	3.122218	1.589087
H	3.006495	2.047862	1.692349
H	2.712808	3.568271	2.567638
C	2.258067	3.682075	-0.964394
H	2.081564	4.731923	-1.186379
H	1.811320	3.082198	-1.748512
C	3.728096	3.392678	-0.682950
H	4.359046	3.960738	-1.365906
H	3.934447	2.333855	-0.834030
C	3.988256	3.763731	0.777333
H	4.956089	3.402292	1.124189
H	3.970528	4.847816	0.902089
C	0.449176	1.861389	0.500492
C	-0.705580	2.247044	-1.142129
H	0.067824	1.860814	-1.833480
O	-1.001450	3.476572	-1.172223
C	-1.763312	1.203592	-0.905203
C	-1.564465	-0.104719	-1.334099
C	-2.952147	1.539265	-0.265819
C	-2.531865	-1.088021	-1.132311
H	-0.634802	-0.349884	-1.829002
C	-3.942950	0.585768	-0.039746
H	-3.085354	2.566177	0.044588
C	-3.712195	-0.721171	-0.481266
H	-4.470040	-1.468024	-0.317639
H	-0.287850	1.935178	1.295672
C	1.181737	0.586534	0.498444
C	0.985423	-0.326720	1.541323
C	2.034658	0.226548	-0.552183
C	1.638003	-1.557400	1.568430
H	0.318456	-0.043611	2.341768
C	2.694206	-0.998445	-0.564666
H	2.160207	0.907974	-1.375848
C	2.490735	-1.868536	0.508624
H	3.009349	-2.815564	0.516213
C	1.497135	-2.534525	2.742123
C	3.609971	-1.423031	-1.717878
C	-5.236220	0.994828	0.675062
C	-2.260144	-2.521199	-1.602571
C	2.824830	-2.551068	3.522818
H	2.759262	-3.237348	4.369917
H	3.651793	-2.873403	2.889176
H	3.062006	-1.557438	3.907360
C	1.201008	-3.955009	2.231445
H	1.114157	-4.641610	3.075736
H	0.265186	-3.982809	1.673864

H	1.991304	-4.329008	1.581592
C	0.372386	-2.131990	3.702574
H	0.564667	-1.168814	4.176354
H	-0.590066	-2.074412	3.192007
H	0.287511	-2.876763	4.495325
C	5.024674	-1.694891	-1.176212
H	5.688916	-1.994752	-1.989337
H	5.440733	-0.799316	-0.710871
H	5.025127	-2.491764	-0.432727
C	3.054964	-2.708118	-2.358673
H	3.699681	-3.028973	-3.179534
H	2.995952	-3.524103	-1.638740
H	2.053926	-2.540430	-2.759190
C	3.711476	-0.350968	-2.808297
H	2.740029	-0.133862	-3.254505
H	4.125981	0.581770	-2.423264
H	4.370199	-0.703024	-3.603424
C	-1.066189	-3.079710	-0.808795
H	-0.839457	-4.100309	-1.125179
H	-0.171442	-2.475210	-0.948534
H	-1.292673	-3.096068	0.257444
C	-3.460241	-3.449071	-1.385525
H	-3.216292	-4.449068	-1.747659
H	-3.721168	-3.535121	-0.329874
H	-4.342073	-3.104271	-1.927856
C	-1.917825	-2.522788	-3.102892
H	-2.745443	-2.122629	-3.691472
H	-1.032963	-1.923799	-3.316084
H	-1.720715	-3.541832	-3.442286
C	-4.892144	1.541043	2.072770
H	-4.238358	2.410898	2.012399
H	-5.802357	1.839577	2.597295
H	-4.387788	0.781007	2.672498
C	-5.941260	2.095921	-0.137431
H	-6.861895	2.404347	0.362733
H	-5.309160	2.976243	-0.251757
H	-6.200388	1.736012	-1.134796
C	-6.211459	-0.175008	0.844866
H	-6.520826	-0.587944	-0.116380
H	-5.778297	-0.981289	1.438695
H	-7.108273	0.171889	1.360588

D-gauche'-tBu
Number of imaginary frequencies: 0
Electronic Energy (SCF) = -1801.12264882 a.u
ZPV corrected Energy = -1800.340233 a.u.
Enthalpy = -1800.299127 a.u.
Free Energy = -1800.413336 a.u.

S	1.040255	3.454195	0.533005
C	2.606410	3.162866	1.464697
H	2.814419	2.096437	1.500527
H	2.461597	3.538316	2.474938
C	1.916363	3.782169	-1.046616
H	1.660827	4.812156	-1.283872
H	1.495527	3.139866	-1.811349
C	3.409156	3.586803	-0.797234
H	3.986354	4.223068	-1.467659
H	3.682725	2.551146	-0.995860
C	3.672171	3.912926	0.672349
H	4.668975	3.607198	0.989494
H	3.583580	4.987851	0.842318
C	0.275976	1.755951	0.419461
C	-0.682024	1.999225	-0.797371
H	-0.101166	1.702981	-1.697502
O	-1.037920	3.300811	-0.782758
C	-1.815127	0.976595	-0.689981
C	-1.617330	-0.346239	-1.073702
C	-3.043177	1.353461	-0.163848
C	-2.618160	-1.304451	-0.922583
H	-0.658917	-0.628637	-1.487287
C	-4.078078	0.429493	-0.007428
H	-3.167877	2.391482	0.110039
C	-3.842950	-0.894199	-0.388199
H	-4.629612	-1.619764	-0.270430
H	-0.324290	1.753002	1.329866
C	1.194212	0.582389	0.411833
C	1.223257	-0.264407	1.518591
C	1.981637	0.281965	-0.699690
C	2.020721	-1.407790	1.534335
H	0.599601	-0.018598	2.364796
C	2.794969	-0.846657	-0.719953
H	1.943241	0.931837	-1.557029
C	2.796426	-1.672912	0.406094
H	3.418972	-2.555803	0.401191
C	2.077785	-2.359692	2.733316
C	3.658716	-1.212874	-1.930387
C	-5.420622	0.891355	0.571234
C	-2.334511	-2.757514	-1.320673
C	3.505332	-2.354684	3.309621
H	3.569236	-3.029291	4.165790
H	4.235862	-2.680728	2.568978
H	3.786291	-1.354080	3.643299
C	1.720719	-3.786243	2.279008
H	1.759592	-4.469856	3.129316
H	0.714413	-3.820621	1.860297
H	2.413519	-4.154939	1.522830
C	1.104211	-1.953541	3.844561
H	1.333707	-0.965089	4.245008
H	0.071338	-1.948558	3.493489
H	1.173530	-2.667530	4.666345
C	5.135143	-1.286160	-1.502119
H	5.762411	-1.546725	-2.356829
H	5.474574	-0.324655	-1.112424
H	5.293572	-2.037963	-0.729124
C	3.215415	-2.584773	-2.470137
H	3.819608	-2.862385	-3.336091
H	3.325620	-3.366715	-1.718832
H	2.169092	-2.559865	-2.779193
C	3.536868	-0.187422	-3.062563
H	2.513736	-0.111820	-3.433106
H	3.859759	0.805690	-2.746020
H	4.169995	-0.491319	-3.897152
C	-1.182241	-3.295978	-0.451995
H	-0.965107	-4.335210	-0.708608
H	-0.270400	-2.717214	-0.592695
H	-1.444940	-3.254384	0.606210
C	-3.550931	-3.668658	-1.126089
H	-3.295743	-4.685800	-1.427787
H	-3.869437	-3.702809	-0.083283
H	-4.399624	-3.345469	-1.730835
C	-1.919368	-2.818212	-2.801381
H	-2.716640	-2.441043	-3.444435
H	-1.024693	-2.226010	-2.992340
H	-1.706922	-3.849278	-3.092112
C	-5.196226	1.469122	1.980576
H	-4.514078	2.318788	1.960565
H	-6.143351	1.807659	2.406359
H	-4.775153	0.713763	2.646885
C	-6.014360	1.986534	-0.333138
H	-6.969675	2.332266	0.067955
H	-5.349558	2.846669	-0.408584
H	-6.186880	1.605267	-1.341286
C	-6.440294	-0.247718	0.677706
H	-6.666364	-0.681782	-0.297342
H	-6.089622	-1.046436	1.333088
H	-7.372872	0.136952	1.093682

D-anti-0-tBu
Number of imaginary frequencies: 0
Electronic Energy (SCF) = -1801.12278428 a.u
ZPV corrected Energy = -1800.343489 a.u.
Enthalpy = -1800.300932 a.u.
Free Energy = -1800.417876 a.u.

S	-0.670978	0.038500	2.724007
C	-0.095183	1.493669	3.726500
H	0.921426	1.726468	3.418024
H	-0.096265	1.175250	4.765818
C	-1.476672	1.148893	1.452524
H	-2.520478	0.855149	1.445351
H	-1.037777	0.904338	0.492817
C	-1.283184	2.594999	1.889080
H	-2.143257	3.200273	1.603276
H	-0.406956	3.019930	1.401634
C	-1.075135	2.607129	3.410272
H	-0.696169	3.569748	3.753887
H	-2.024180	2.425131	3.919620
C	0.578426	-0.885046	2.118767
C	-0.628123	-2.883996	-0.100199
O	0.402362	-3.128467	-0.695827
H	0.481135	-1.919028	2.410522
C	1.697067	-0.476402	1.317125
C	2.653360	-1.449230	0.953641
C	1.896801	0.835805	0.844693
C	3.751608	-1.143911	0.163647
H	2.497121	-2.458786	1.300511
C	2.991191	1.171450	0.051578
H	1.183429	1.598199	1.103349
C	3.908938	0.174067	-0.280960
H	4.758867	0.424974	-0.897950
H	-0.904542	-3.461147	0.800757
C	-1.623575	-1.878891	-0.509027
C	-2.867917	-1.873734	0.127255
C	-1.342070	-0.962893	-1.515111
C	-3.848755	-0.966263	-0.247000
H	-3.044109	-2.594695	0.912369
C	-2.293447	-0.018176	-1.897698
H	-0.363315	-0.987047	-1.971774
C	-3.532076	-0.047865	-1.257592
H	-4.283061	0.671248	-1.543758
C	3.220238	2.598730	-0.463926
C	4.782522	-2.202016	-0.249739
C	-1.938841	1.023905	-2.962585
C	-5.235051	-0.924036	0.403168
C	6.185468	-1.764827	0.208306
H	6.221311	-1.651843	1.293496
H	6.927109	-2.513203	-0.079698
H	6.478580	-0.814893	-0.238036
C	4.491308	-3.578168	0.358903
H	4.501719	-3.547946	1.449505
H	3.524748	-3.966816	0.037409
H	5.257451	-4.286280	0.038505
C	4.771829	-2.343848	-1.782714
H	5.025785	-1.404195	-2.273344
H	5.497304	-3.096581	-2.099593
H	3.785627	-2.651848	-2.134107
C	4.556857	3.129853	0.084662
H	5.394495	2.508393	-0.231283
H	4.737157	4.146125	-0.273068
H	4.546914	3.150609	1.176075
C	2.111438	3.565312	-0.034954
H	1.138066	3.257967	-0.417212
H	2.042926	3.646546	1.050619
H	2.323581	4.560708	-0.428638
C	3.275185	2.588594	-2.002401
H	3.428060	3.601400	-2.381866
H	4.089940	1.966043	-2.371170
H	2.343659	2.205538	-2.421412
C	-1.502281	0.312334	-4.255076
H	-2.306386	-0.314741	-4.644254
H	-1.244784	1.048392	-5.019029
H	-0.629532	-0.319519	-4.092724
C	-0.774974	1.885259	-2.438626
H	-0.488199	2.627474	-3.186006
H	-1.063610	2.414172	-1.529170
H	0.100130	1.278110	-2.212624
C	-3.114716	1.948264	-3.293450
H	-3.440844	2.518107	-2.422125
H	-2.808990	2.662284	-4.059270
H	-3.971089	1.393048	-3.679458
C	-5.402151	-2.002688	1.478165
H	-5.287181	-3.006087	1.065883
H	-4.681344	-1.881453	2.288166
H	-6.401335	-1.933046	1.910137
C	-6.308868	-1.144471	-0.677551
H	-7.303689	-1.124288	-0.228438
H	-6.272002	-0.371387	-1.445088
H	-6.175084	-2.111326	-1.165679
C	-5.454288	0.449849	1.062056
H	-4.730855	0.618288	1.861387
H	-5.360047	1.263266	0.342739
H	-6.454267	0.500827	1.497032

D-anti-TS0-tBu
Number of imaginary frequencies: 1
Electronic Energy (SCF) = -1801.10537010 a.u
ZPV corrected Energy = -1800.324728 a.u.
Enthalpy = -1800.283944 a.u.
Free Energy = -1800.395488 a.u.

S	-0.286679	-2.337505	0.402034
C	0.932448	-3.019901	1.613027
H	1.914368	-2.684274	1.286803
H	0.876114	-4.103718	1.557516
C	-0.848899	-1.013145	1.543556
H	-1.846470	-1.316991	1.852176
H	-0.925754	-0.090529	0.982043
C	0.129194	-0.988079	2.710140
H	-0.344022	-0.538685	3.582817
H	1.001057	-0.389797	2.450003
C	0.547413	-2.435098	2.966687
H	1.390098	-2.501303	3.654712
H	-0.282734	-2.997394	3.397279
C	0.594731	-1.586200	-0.958110
C	-0.539525	-0.746408	-2.295802
O	0.158869	0.106098	-2.928519
H	0.908623	-2.461458	-1.525369
C	1.759721	-0.745432	-0.590781
C	3.055049	-1.254783	-0.755959
C	1.606774	0.552318	-0.116118
C	4.178724	-0.504362	-0.432916
H	3.159304	-2.257589	-1.142785
C	2.711880	1.337074	0.225624
H	0.613225	0.962286	-0.031503
C	3.980788	0.789842	0.064684
H	4.845899	1.381050	0.319669
H	-0.763433	-1.713647	-2.791639
C	-1.774061	-0.287384	-1.545214
C	-2.765472	-1.211939	-1.207716
C	-1.936006	1.042431	-1.189801
C	-3.887019	-0.834810	-0.480054
H	-2.637445	-2.236526	-1.526612

C	-3.058351	1.467759	-0.468940
H	-1.167453	1.739329	-1.491788
C	-4.008157	0.513174	-0.115103
H	-4.876894	0.816616	0.447189
C	2.488980	2.765000	0.734204
C	5.605708	-1.038465	-0.594288
C	-3.200412	2.944301	-0.083153
C	-4.970828	-1.830120	-0.053285
C	6.309382	-1.038583	0.774965
H	5.777051	-1.674098	1.485262
H	7.327841	-1.419746	0.675680
H	6.367439	-0.035672	1.197691
C	5.634988	-2.466661	-1.148510
H	5.123472	-3.168401	-0.487808
H	5.172861	-2.527527	-2.134678
H	6.670075	-2.797477	-1.245627
C	6.383646	-0.129584	-1.562996
H	6.441199	0.894323	-1.193956
H	7.403373	-0.498957	-1.690161
H	5.904148	-0.107911	-2.543023
C	3.796551	3.464082	1.120038
H	4.470874	3.562603	0.268454
H	3.576370	4.468412	1.485237
H	4.322755	2.929989	1.912900
C	1.581848	2.729590	1.977292
H	0.625553	2.254793	1.767542
H	2.062019	2.179910	2.789001
H	1.380803	3.744043	2.327325
C	1.812595	3.588237	-0.376918
H	1.608761	4.602746	-0.028202
H	2.457894	3.651001	-1.254692
H	0.870445	3.142484	-0.690969
C	-3.127868	3.815586	-1.349574
H	-3.932487	3.561684	-2.041943
H	-3.226098	4.870712	-1.086078
H	-2.181021	3.688017	-1.872992
C	-2.046644	3.328445	0.858899
H	-2.120790	4.380090	1.144011
H	-2.071968	2.728113	1.770255
H	-1.080808	3.179427	0.381626
C	-4.524002	3.243087	0.628892
H	-4.614808	2.693634	1.567087
H	-4.576646	4.307283	0.863738
H	-5.384240	2.997013	0.004622
C	-4.696704	-3.246748	-0.569474
H	-4.660851	-3.279902	-1.659212
H	-3.755689	-3.643672	-0.186075
H	-5.495623	-3.913855	-0.242283
C	-6.337491	-1.376513	-0.595748
H	-7.115119	-2.082304	-0.296391
H	-6.615913	-0.392787	-0.218574
H	-6.324240	-1.327817	-1.685946
C	-5.027138	-1.880919	1.484710
H	-4.070019	-2.206949	1.896567
H	-5.261666	-0.905103	1.910017
H	-5.794565	-2.584913	1.813144

D-anti-tBu
Number of imaginary frequencies: 0
Electronic Energy (SCF) = -1801.11176206 a.u
ZPV corrected Energy = -1800.329663 a.u.
Enthalpy = -1800.288585 a.u.
Free Energy = -1800.400871 a.u.

S	0.552708	-2.870485	-0.623329
C	2.236830	-3.534437	-0.309861
H	2.943721	-2.824409	-0.729851
H	2.317066	-4.482739	-0.833773
C	0.283153	-2.367498	1.118272
H	-0.419709	-3.096554	1.515985
H	-0.181242	-1.388597	1.125940
C	1.641451	-2.440291	1.803792
H	1.506478	-2.539192	2.880426
H	2.200909	-1.525935	1.612663
C	2.369402	-3.642805	1.207079
H	3.423531	-3.661765	1.481955
H	1.913112	-4.570107	1.555909
C	0.836427	-1.295564	-1.663549
C	-0.464097	-0.857659	-2.456555
O	-0.061678	0.059816	-3.330735
H	1.470857	-1.734172	-2.433523
C	1.621385	-0.284381	-0.890948
C	3.011152	-0.246735	-1.054028
C	1.027580	0.565894	0.033000
C	3.808275	0.588937	-0.282527
H	3.454868	-0.886849	-1.801434
C	1.795365	1.416346	0.831974
H	-0.044486	0.566160	0.131089
C	3.176762	1.401372	0.666748
H	3.787693	2.044184	1.280552
H	-0.800887	-1.826036	-2.911501
C	-1.640317	-0.444720	-1.548968
C	-2.349392	-1.381643	-0.799775
C	-2.042322	0.885555	-1.521031
C	-3.411171	-1.005921	0.021789
H	-2.081374	-2.425309	-0.879518
C	-3.122937	1.300459	-0.741414
H	-1.483449	1.568541	-2.141341
C	-3.775485	0.342106	0.036466
H	-4.609019	0.649058	0.652035
C	1.094832	2.342888	1.830056
C	5.332754	0.637461	-0.421530
C	-3.636531	2.745480	-0.738479
C	-4.182590	-2.008408	0.887625
C	5.974741	0.202675	0.908570
H	5.678996	-0.815821	1.167632
H	7.063325	0.229980	0.828929
H	5.682180	0.857435	1.729354
C	5.846661	-0.288020	-1.529163
H	5.603545	-1.332975	-1.330711
H	5.434829	-0.020383	-2.503074
H	6.932699	-0.208356	-1.593570
C	5.772208	2.075072	-0.752097
H	5.477568	2.775961	0.028743
H	6.858257	2.122486	-0.853386
H	5.327443	2.409668	-1.690690
C	2.081586	3.048962	2.765527
H	2.766658	3.698301	2.219574
H	1.529725	3.672248	3.470575
H	2.673022	2.335458	3.342102
C	0.106194	1.538932	2.693614
H	-0.673213	1.070512	2.094076
H	0.622796	0.756492	3.252761
H	-0.383662	2.199588	3.411222
C	0.320562	3.408477	1.035496
H	-0.209009	4.080915	1.713247
H	0.999396	4.004573	0.423345
H	-0.409631	2.947093	0.373782
C	-2.781618	3.668571	-1.613308
H	-2.782087	3.349600	-2.656053
H	-3.184638	4.682035	-1.574474
H	-1.747009	3.707905	-1.273347
C	-3.640908	3.300298	0.697021
H	-4.004874	4.329937	0.701191
H	-4.287104	2.716289	1.352322
H	-2.636904	3.294763	1.120975
C	-5.076002	2.763847	-1.286074

H	-5.744824	2.159695	-0.672688
H	-5.462412	3.785389	-1.301275
H	-5.107840	2.373155	-2.304649
C	-3.664030	-3.441539	0.725613
H	-3.759104	-3.793022	-0.302628
H	-2.617363	-3.529796	1.019429
H	-4.244361	-4.112238	1.361068
C	-5.671844	-1.992076	0.499179
H	-6.230227	-2.704089	1.110703
H	-6.114959	-1.007113	0.643720
H	-5.801476	-2.268512	-0.548617
C	-4.041452	-1.609355	2.367955
H	-2.993368	-1.612407	2.673468
H	-4.441367	-0.612534	2.553555
H	-4.582731	-2.312954	3.004115

E-gauche-0-tBu
Number of imaginary frequencies: 0
Electronic Energy (SCF) = -1801.12111977 a.u
ZPV corrected Energy = -1800.342794 a.u.
Enthalpy = -1800.299837 a.u.
Free Energy = -1800.420118 a.u.

S	-3.631900	-1.954615	0.054730
C	-5.436039	-2.216893	0.476929
H	-5.835642	-1.238870	0.730659
H	-5.449193	-2.855625	1.360111
C	-3.731460	-3.118919	-1.382464
H	-2.835367	-3.733103	-1.355130
H	-3.713920	-2.484517	-2.266827
C	-5.035926	-3.887522	-1.251285
H	-4.920296	-4.713438	-0.546557
H	-5.327358	-4.309383	-2.213253
C	-6.075727	-2.894682	-0.721793
H	-6.306488	-2.155009	-1.491245
H	-7.006262	-3.390515	-0.444890
C	-3.345431	-0.415835	-0.532699
H	-4.006071	-0.044063	-1.305425
C	-0.172717	-2.389947	-0.920067
H	-0.969894	-1.837850	-1.447411
O	-0.406611	-3.472356	-0.415028
C	1.127357	-1.707950	-0.901426
C	1.232716	-0.471103	-1.542731
C	2.222934	-2.261913	-0.247775
C	2.435270	0.221084	-1.544780
H	0.353459	-0.066291	-2.021142
C	3.443489	-1.591273	-0.222358
H	2.100907	-3.215321	0.245464
C	3.520665	-0.362046	-0.877944
H	4.454619	0.176807	-0.862374
C	-2.434797	0.492197	0.119558
C	-2.447397	1.853470	-0.253461
C	-1.498795	0.103240	1.101200
C	-1.596387	2.788249	0.326943
H	-3.160469	2.161729	-1.004251
C	-0.633470	1.013806	1.693690
H	-1.457122	-0.935185	1.389450
C	-0.692248	2.355460	1.300972
H	-0.031333	3.071651	1.765516
C	-1.660040	4.280353	-0.030655
C	0.378632	0.591002	2.767374
C	2.601926	1.591155	-2.206034
C	4.629531	-2.203427	0.528621
C	-2.147564	5.063943	1.202367
H	-2.210835	6.130677	0.975126
H	-3.137026	4.722743	1.512261
H	-1.469593	4.938100	2.046704
C	-0.266520	4.793934	-0.431420
H	-0.314341	5.859279	-0.667600
H	0.460919	4.663020	0.368525
H	0.102969	4.268141	-1.311452
C	-2.618765	4.568688	-1.191445
H	-2.327098	4.034364	-2.096944
H	-3.646054	4.293948	-0.949599
H	-2.606895	5.636640	-1.415806
C	0.354794	-0.917318	3.039391
H	-0.616997	-1.248794	3.407769
H	0.595727	-1.491791	2.145219
H	1.098324	-1.160576	3.800215
C	1.799884	0.968365	2.314476
H	2.527533	0.671476	3.072884
H	2.055376	0.465997	1.382985
H	1.900734	2.041597	2.157093
C	0.056728	1.322465	4.083124
H	0.765861	1.032085	4.861782
H	0.112872	2.404479	3.963797
H	-0.948341	1.074073	4.429363
C	2.920337	2.631614	-1.116831
H	2.112136	2.685803	-0.388634
H	3.839697	2.384062	-0.585452
H	3.044687	3.619596	-1.564369
C	3.758436	1.541883	-3.219625
H	3.561004	0.804804	-4.000096
H	3.879564	2.516692	-3.695874
H	4.704504	1.284216	-2.743700
C	1.332751	2.030165	-2.943512
H	1.070583	1.332847	-3.740997
H	0.481172	2.114458	-2.269135
H	1.496019	3.008442	-3.397339
C	4.248530	-2.378107	2.010052
H	4.008069	-1.416523	2.464957
H	3.384173	-3.031251	2.128377
H	5.081297	-2.818637	2.561707
C	4.959612	-3.579309	-0.076901
H	5.231516	-3.485472	-1.129797
H	5.800664	-4.030903	0.452784
H	4.113367	-4.262632	-0.006414
C	5.884370	-1.327898	0.453452
H	6.694529	-1.809201	1.002807
H	6.219349	-1.186374	-0.575175
H	5.720175	-0.345314	0.898230

E-gauche-TS0-tBu
Number of imaginary frequencies: 1
Electronic Energy (SCF) = -1801.11183578 a.u
ZPV corrected Energy = -1800.332087 a.u.
Enthalpy = -1800.291050 a.u.
Free Energy = -1800.403849 a.u.

S	-2.246589	-2.573731	-0.117828
C	-3.624909	-2.162146	1.024909
H	-3.448901	-1.165805	1.419346
H	-3.562976	-2.884494	1.838726
C	-3.326977	-3.540541	-1.248746
H	-2.855615	-4.506425	-1.391188
H	-3.314930	-3.007976	-2.193477
C	-4.711299	-3.602354	-0.602226
H	-4.772640	-4.469501	0.056988
H	-5.471310	-3.715981	-1.374773
C	-4.900462	-2.325248	0.213513
H	-5.035072	-1.465459	-0.444343
H	-5.766791	-2.384214	0.872046
C	-1.924519	-1.081633	-1.036875
H	-2.734758	-0.913633	-1.738457
C	-0.467941	-1.851962	-2.248061
H	-0.783244	-1.213719	-3.093440

O	-0.653182	-3.109624	-2.325505
C	0.776994	-1.310432	-1.608427
C	1.197338	-0.009098	-1.891130
C	1.533958	-2.101449	-0.756963
C	2.356803	0.510404	-1.332048
H	0.597143	0.583711	-2.565493
C	2.709149	-1.617136	-0.172940
H	1.200711	-3.114123	-0.580311
C	3.092560	-0.311139	-0.465525
H	3.994503	0.084731	-0.026500
C	-1.600162	0.088811	-0.222050
C	-1.907706	1.364106	-0.720141
C	-0.948508	-0.001576	1.015276
C	-1.578194	2.519518	-0.018196
H	-2.415033	1.428018	-1.671427
C	-0.630381	1.132312	1.756690
H	-0.691806	-0.976660	1.399245
C	-0.943192	2.381683	1.218833
H	-0.697516	3.269643	1.781380
C	-1.943154	3.920404	-0.525812
C	0.058893	1.041955	3.123530
C	2.852864	1.929302	-1.630850
C	3.550567	-2.543528	0.713225
C	-3.017217	4.516510	0.403197
H	-3.301721	5.514878	0.063690
H	-3.912643	3.892294	0.412439
H	-2.653713	4.598744	1.427793
C	-0.704987	4.833783	-0.510227
H	-0.976133	5.832572	-0.858305
H	-0.284588	4.934072	0.489732
H	0.074294	4.446772	-1.166042
C	-2.497444	3.898475	-1.954565
H	-1.781276	3.470877	-2.657842
H	-3.424936	3.328879	-2.023492
H	-2.712892	4.918507	-2.276293
C	0.102501	-0.394639	3.655114
H	-0.899885	-0.810887	3.770878
H	0.670344	-1.052513	2.999727
H	0.585290	-0.406811	4.633446
C	1.502940	1.556253	2.989096
H	2.017701	1.496543	3.950684
H	2.057951	0.960623	2.264453
H	1.523695	2.594118	2.656905
C	-0.691948	1.903259	4.154121
H	-0.213025	1.815902	5.131406
H	-0.696382	2.957899	3.881496
H	-1.728553	1.576027	4.254525
C	2.815147	2.759411	-0.335785
H	1.802620	2.805713	0.063103
H	3.459254	2.328193	0.430408
H	3.155922	3.779144	-0.528845
C	4.298133	1.877211	-2.157714
H	4.357195	1.284093	-3.072168
H	4.652280	2.885383	-2.383227
H	4.980136	1.440169	-1.428665
C	1.989909	2.632758	-2.683724
H	2.003292	2.099228	-3.635479
H	0.954017	2.726234	-2.360743
H	2.375395	3.638379	-2.858882
C	2.700838	-3.072522	1.880818
H	2.388638	-2.256286	2.532409
H	1.806374	-3.586415	1.529814
H	3.281304	-3.778231	2.478942
C	4.034555	-3.734534	-0.134972
H	4.638643	-3.391132	-0.976654
H	4.645437	-4.407431	0.470877
H	3.196745	-4.306243	-0.534079
C	4.779167	-1.840386	1.300167
H	5.331287	-2.539285	1.930593
H	5.458042	-1.490568	0.521327
H	4.499117	-0.985860	1.917986

E-gauche-tBu

Number of imaginary frequencies: 0
Electronic Energy (SCF) = -1801.12424112 a.u
ZPV corrected Energy = -1800.341881 a.u.
Enthalpy = -1800.300902 a.u.
Free Energy = -1800.413774 a.u.

S	1.115419	3.212368	-0.159045
C	2.569642	3.059923	0.982650
H	2.676446	2.027943	1.299183
H	2.312137	3.664678	1.852085
C	1.963102	4.492278	-1.169967
H	1.250879	5.301771	-1.293477
H	2.125342	4.030662	-2.138496
C	3.257005	4.880716	-0.464396
H	3.065226	5.667058	0.267682
H	3.971559	5.268598	-1.190569
C	3.771512	3.630973	0.247857
H	4.145098	2.906097	-0.478205
H	4.583189	3.858518	0.939215
C	1.271494	1.634871	-1.123932
H	2.123376	1.798529	-1.780036
C	-0.043554	1.783565	-1.996886
H	0.233480	1.352065	-2.979064
O	-0.298022	3.115731	-2.034686
C	-1.175446	0.910855	-1.463883
C	-1.215784	-0.450162	-1.771844
C	-2.161160	1.453448	-0.659315
C	-2.212879	-1.275449	-1.269483
H	-0.445296	-0.851473	-2.414250
C	-3.182341	0.658112	-0.124470
H	-2.121118	2.516305	-0.470928
C	-3.181116	-0.699179	-0.433002
H	-3.958967	-1.332468	-0.035886
C	1.500789	0.416692	-0.290134
C	2.388046	-0.552137	-0.753005
C	0.809111	0.199405	0.901132
C	2.582260	-1.744156	-0.054893
H	2.915105	-0.365197	-1.676850
C	1.011537	-0.955504	1.649871
H	0.111159	0.947771	1.241598
C	1.892666	-1.914613	1.145234
H	2.051102	-2.821582	1.710503
C	3.520878	-2.848505	-0.551850
C	0.336440	-1.182959	3.006631
C	-2.301038	-2.766649	-1.614969
C	-4.281446	1.308910	0.723987
C	4.629851	-3.085358	0.488932
H	5.306817	-3.870452	0.146194
H	5.214624	-2.177277	0.646586
H	4.219612	-3.393114	1.450597
C	2.717453	-4.147794	-0.742317
H	3.372956	-4.947368	-1.093308
H	2.256473	-4.475822	0.189139
H	1.924603	-4.009584	-1.478945
C	4.182190	-2.494040	-1.887782
H	3.443707	-2.336982	-2.675231
H	4.796557	-1.595746	-1.811116
H	4.831115	-3.313478	-2.199910
C	-0.660843	-0.071925	3.347162
H	-0.178497	0.903709	3.420594
H	-1.449218	-0.006847	2.598376
H	-1.128834	-0.283416	4.309659
C	-0.427081	-2.517588	3.004529
H	-0.874098	-2.692077	3.985261

H	-1.226737	-2.501928	2.264884
H	0.225067	-3.361490	2.781019
C	1.422652	-1.220472	4.097169
H	0.967472	-1.369312	5.078529
H	2.131037	-2.031788	3.927518
H	1.982446	-0.283607	4.120453
C	-2.197548	-3.605655	-0.330196
H	-1.231954	-3.452971	0.151471
H	-2.977942	-3.346361	0.385145
H	-2.296303	-4.667904	-0.564614
C	-3.650347	-3.052533	-2.299189
H	-3.754421	-2.464333	-3.212722
H	-3.722861	-4.109451	-2.564726
H	-4.490499	-2.812554	-1.647496
C	-1.180062	-3.210739	-2.560999
H	-1.218263	-2.678921	-3.512736
H	-0.195524	-3.051817	-2.120295
H	-1.282045	-4.276720	-2.770963
C	-3.660374	2.157469	1.847154
H	-3.073884	1.538427	2.526426
H	-3.008581	2.936598	1.453709
H	-4.448072	2.642875	2.427062
C	-5.121797	2.224998	-0.185444
H	-5.582153	1.653096	-0.993104
H	-5.917225	2.705756	0.388584
H	-4.508140	3.006340	-0.634517
C	-5.213231	0.277014	1.368816
H	-5.952940	0.789442	1.986088
H	-5.754616	-0.306077	0.622959
H	-4.664396	-0.414725	2.010126

E-gauche'-0-tBu
Number of imaginary frequencies: 0
Electronic Energy (SCF) = -1801.11983515 a.u
ZPV corrected Energy = -1800.341183 a.u.
Enthalpy = -1800.298523 a.u.
Free Energy = -1800.416023 a.u.

S	3.945903	-0.590501	-0.380031
C	5.544080	-0.459937	0.597310
H	5.287663	0.008380	1.543938
H	6.193629	0.203442	0.025742
C	4.370259	-2.281363	-0.978474
H	4.119274	-2.326598	-2.034095
H	3.724172	-2.956868	-0.421719
C	5.840401	-2.516168	-0.667356
H	6.468506	-2.052650	-1.430494
H	6.056261	-3.584546	-0.661764
C	6.108789	-1.865579	0.692570
H	5.595548	-2.423713	1.478390
H	7.171783	-1.851160	0.934057
C	2.593143	-0.775017	0.595151
H	2.599830	-1.606954	1.287954
C	1.092972	-1.671258	-2.018662
H	1.573761	-2.568822	-1.596592
O	1.675128	-0.985508	-2.840281
C	-0.287683	-1.458282	-1.562098
C	-0.850836	-2.393485	-0.695998
C	-1.040027	-0.380005	-2.029425
C	-2.181382	-2.284508	-0.299302
H	-0.231750	-3.209827	-0.352883
C	-2.368702	-0.237452	-1.652353
H	-0.562829	0.322848	-2.693545
C	-2.909896	-1.201527	-0.793352
H	-3.946816	-1.105596	-0.506545
C	1.673255	0.312450	0.813375
C	0.696444	0.176500	1.825110
C	1.661067	1.504270	0.066636
C	-0.235960	1.168678	2.087258
H	0.697923	-0.735865	2.403141
C	0.727879	2.512554	0.296602
H	2.402918	1.636916	-0.705996
C	-0.215262	2.335065	1.309784
H	-0.939006	3.108273	1.509763
C	-2.869820	-3.335885	0.577782
C	-3.255992	0.906017	-2.153177
C	-1.274581	1.035837	3.208994
C	0.803603	3.821977	-0.502850
C	-1.863087	-4.299455	1.216391
H	-1.326493	-4.884345	0.468757
H	-1.130119	-3.767303	1.824844
H	-2.391333	-5.000452	1.864067
C	-3.672183	-2.667833	1.706228
H	-4.162870	-3.432356	2.311373
H	-3.022207	-2.085711	2.357950
H	-4.446679	-2.004691	1.322848
C	-3.833666	-4.146773	-0.308892
H	-4.339090	-4.913185	0.282529
H	-4.595089	-3.504444	-0.752982
H	-3.295033	-4.641112	-1.119182
C	-1.139876	-0.281731	3.979849
H	-1.260324	-1.146439	3.328088
H	-0.170562	-0.362180	4.474150
H	-1.911568	-0.334767	4.749618
C	-1.098439	2.191340	4.211087
H	-1.238379	3.161361	3.734611
H	-1.828648	2.105506	5.019097
H	-0.100112	2.173853	4.652338
C	-2.688955	1.103755	2.607189
H	-3.440838	0.976280	3.389457
H	-2.868585	2.061864	2.121270
H	-2.830832	0.319976	1.864452
C	1.869376	4.724530	0.147343
H	1.954169	5.669985	-0.394115
H	1.610151	4.947814	1.183708
H	2.846987	4.240289	0.141211
C	-0.535045	4.573922	-0.506840
H	-0.825560	4.901509	0.491052
H	-0.451134	5.465795	-1.130276
H	-1.338185	3.955903	-0.909095
C	1.202445	3.564918	-1.965536
H	2.208929	3.158128	-2.053173
H	0.516603	2.866778	-2.445582
H	1.179646	4.502460	-2.524373
C	-4.464916	0.321425	-2.905566
H	-5.070197	-0.318034	-2.263090
H	-5.103630	1.127611	-3.271900
H	-4.140229	-0.271103	-3.762795
C	-3.752525	1.728298	-0.951711
H	-4.380350	2.552840	-1.295653
H	-4.342193	1.120944	-0.265351
H	-2.912307	2.144135	-0.396916
C	-2.505753	1.844227	-3.103012
H	-1.648792	2.307233	-2.615918
H	-2.151726	1.321606	-3.992851
H	-3.174538	2.642066	-3.429098

E-gauche'-TS0-tBu
Number of imaginary frequencies: 1
Electronic Energy (SCF) = -1801.11300467 a.u
ZPV corrected Energy = -1800.333334 a.u.
Enthalpy = -1800.292263 a.u.
Free Energy = -1800.404416 a.u.

S	3.618313	-1.083242	-0.711275
C	4.744145	-0.930059	0.736440

H	4.228632	-0.348511	1.495997
H	5.612172	-0.372930	0.385748
C	4.090462	-2.824056	-1.048186
H	4.272955	-2.914442	-2.114009
H	3.230860	-3.430996	-0.777869
C	5.304009	-3.118354	-0.169291
H	6.214983	-2.778746	-0.664603
H	5.390293	-4.192756	-0.010232
C	5.107536	-2.352468	1.138048
H	4.297300	-2.796126	1.718585
H	6.005507	-2.357417	1.755254
C	1.981170	-1.224381	-0.057351
H	1.932191	-2.033317	0.666182
C	1.063376	-1.873782	-1.744509
H	1.264088	-2.936244	-1.505686
O	1.636927	-1.360458	-2.755562
C	-0.360133	-1.531181	-1.408602
C	-1.098592	-2.351912	-0.559303
C	-0.947448	-0.392609	-1.948988
C	-2.422765	-2.055395	-0.241914
H	-0.620021	-3.233949	-0.156349
C	-2.266568	-0.057288	-1.651067
H	-0.345406	0.211106	-2.608841
C	-2.980186	-0.900906	-0.797323
H	-4.006236	-0.655859	-0.564433
C	1.444069	0.050742	0.428026
C	0.605940	0.047198	1.551086
C	1.669568	1.264868	-0.235341
C	-0.001513	1.211179	2.009414
H	0.436500	-0.893025	2.053301
C	1.089834	2.451427	0.207700
H	2.295357	1.267375	-1.113689
C	0.256372	2.402133	1.326040
H	-0.199288	3.314711	1.679851
C	-3.288079	-2.968333	0.635260
C	-2.940194	1.201175	-2.208678
C	-0.946057	1.221079	3.216596
C	1.376292	3.799891	-0.464904
C	-2.472729	-4.091494	1.285876
H	-2.029499	-4.755922	0.543357
H	-1.670757	-3.695880	1.911299
H	-3.124262	-4.694469	1.920297
C	-3.960155	-2.157687	1.756623
H	-4.578736	-2.814116	2.372155
H	-3.215839	-1.691453	2.401720
H	-4.602822	-1.371349	1.362650
C	-4.379706	-3.606279	-0.244079
H	-5.011844	-4.267697	0.352750
H	-5.017356	-2.846005	-0.696083
H	-3.935291	-4.194202	-1.049082
C	-0.979891	-0.129134	3.940674
H	-1.329023	-0.930504	3.290970
H	0.004592	-0.404680	4.322795
H	-1.663351	-0.070366	4.789072
C	-0.500375	2.289888	4.229744
H	-0.517658	3.291160	3.800716
H	-1.167998	2.287367	5.093804
H	0.513118	2.092078	4.583908
C	-2.367865	1.548757	2.725379
H	-3.068821	1.547340	3.563162
H	-2.406199	2.530192	2.252879
H	-2.703455	0.812035	1.995984
C	2.212643	4.660154	0.501000
H	2.439985	5.627973	0.048515
H	1.678454	4.840169	1.434456
H	3.156319	4.167384	0.742286
C	0.062467	4.535758	-0.778886
H	-0.520653	4.732511	0.119936
H	0.278448	5.496484	-1.250625
H	-0.556861	3.955066	-1.461446
C	2.156768	3.643583	-1.774859
H	3.141143	3.202728	-1.613230
H	1.619619	3.021046	-2.491668
H	2.305812	4.624706	-2.228366
C	-4.258442	0.828443	-2.909424
H	-4.964767	0.359955	-2.224507
H	-4.733396	1.723813	-3.316168
H	-4.078086	0.135974	-3.733705
C	-3.239329	2.161453	-1.043091
H	-3.701271	3.078877	-1.415086
H	-3.920307	1.708646	-0.322287
H	-2.322933	2.427333	-0.517281
C	-2.052837	1.927702	-3.225086
H	-1.109166	2.247086	-2.785233
H	-1.827651	1.294981	-4.085019
H	-2.568517	2.817729	-3.589380

E-gauche'-tBu

Number of imaginary frequencies: 0
Electronic Energy (SCF) = -1801.12393760 a.u
ZPV corrected Energy = -1800.341397 a.u.
Enthalpy = -1800.300544 a.u.
Free Energy = -1800.411666 a.u.

S	3.570731	-1.083956	-0.841615
C	4.556188	-1.124491	0.720813
H	3.994313	-0.611130	1.496941
H	5.463963	-0.559597	0.513411
C	4.073396	-2.784021	-1.315667
H	4.373701	-2.740062	-2.357454
H	3.186095	-3.404139	-1.231638
C	5.176706	-3.214865	-0.352420
H	6.143539	-2.854716	-0.707647
H	5.222144	-4.302783	-0.306159
C	4.860816	-2.588757	1.004705
H	3.991157	-3.074034	1.451275
H	5.691831	-2.684497	1.703495
C	1.882835	-1.276547	-0.136659
H	1.933183	-2.015847	0.661627
C	1.119977	-1.809133	-1.420967
H	1.200705	-2.917622	-1.342556
O	1.741020	-1.295267	-2.500710
C	-0.368293	-1.511363	-1.261979
C	-1.152045	-2.293993	-0.417890
C	-0.934905	-0.421886	-1.908634
C	-2.494712	-1.993978	-0.193549
H	-0.692669	-3.143894	0.068077
C	-2.273646	-0.084825	-1.709214
H	-0.295135	0.151066	-2.560268
C	-3.029283	-0.881822	-0.847804
H	-4.067982	-0.632794	-0.683698
C	1.422612	0.040762	0.409073
C	0.701242	0.047105	1.601281
C	1.615143	1.234463	-0.285150
C	0.147516	1.223476	2.100808
H	0.555750	-0.889567	2.117304
C	1.107194	2.436838	0.203918
H	2.148403	1.207325	-1.220946
C	0.374878	2.403504	1.390912
H	-0.034324	3.326240	1.775675
C	-3.393933	-2.838072	0.717282
C	-2.921695	1.135465	-2.372043
C	-0.715491	1.253827	3.365742
C	1.349431	3.776264	-0.500576
C	-2.631431	-3.985785	1.388066
H	-2.216463	-4.679658	0.655831
H	-1.814206	-3.618442	2.010695

H	-3.311016	-4.548625	2.029974
C	-3.993708	-1.951944	1.823397
H	-4.645905	-2.544681	2.468472
H	-3.207975	-1.518029	2.442437
H	-4.584817	-1.134878	1.410720
C	-4.536939	-3.441313	-0.119192
H	-5.189098	-4.049680	0.511388
H	-5.145228	-2.663887	-0.581586
H	-4.142538	-4.076822	-0.914093
C	-0.781883	-0.112240	4.056550
H	-1.216685	-0.872915	3.408498
H	0.206054	-0.454025	4.369948
H	-1.407389	-0.039551	4.947324
C	-0.146423	2.272207	4.368470
H	-0.128951	3.280487	3.955689
H	-0.759904	2.292648	5.271440
H	0.872626	2.007314	4.656553
C	-2.145040	1.670722	2.972139
H	-2.792513	1.680790	3.851538
H	-2.160366	2.666931	2.529739
H	-2.564667	0.974500	2.245469
C	2.219216	4.661752	0.411054
H	2.411858	5.624193	-0.067705
H	1.728544	4.851987	1.366070
H	3.179649	4.184586	0.614494
C	0.011571	4.488360	-0.764705
H	-0.535987	4.682966	0.156978
H	0.190881	5.447763	-1.254075
H	-0.625856	3.890414	-1.415144
C	2.070590	3.604010	-1.841756
H	3.059391	3.160530	-1.718033
H	1.500800	2.976704	-2.528444
H	2.202045	4.579935	-2.311384
C	-4.168552	0.704622	-3.164142
H	-4.917048	0.240796	-2.521964
H	-4.629921	1.571832	-3.641640
H	-3.903898	-0.011212	-3.944509
C	-3.337157	2.133506	-1.275349
H	-3.783425	3.025427	-1.720955
H	-4.067474	1.695151	-0.594724
H	-2.473617	2.441667	-0.685596
C	-1.965902	1.843771	-3.337847
H	-1.069700	2.200662	-2.831983
H	-1.655393	1.185280	-4.150349
H	-2.465997	2.707622	-3.778997

E-anti-0-tBu
Number of imaginary frequencies: 0
Electronic Energy (SCF) = -1801.12162287 a.u
ZPV corrected Energy = -1800.343260 a.u.
Enthalpy = -1800.300368 a.u.
Free Energy = -1800.420449 a.u.

S	0.441520	0.612845	2.148893
C	0.565189	1.119429	3.951370
H	-0.434392	1.020818	4.366270
H	0.858922	2.169728	3.950167
C	2.057087	-0.277970	2.221747
H	2.615082	-0.012761	1.331312
H	1.802384	-1.334791	2.183861
C	2.734225	0.106113	3.527699
H	3.253510	1.060304	3.418895
H	3.473991	-0.645568	3.802715
C	1.626556	0.234051	4.577581
H	1.209607	-0.750904	4.798176
H	1.997638	0.656031	5.511800
C	-0.717581	-0.558294	1.869222
C	-0.101239	-1.612311	-1.376464
O	-0.289119	-2.813823	-1.378503
H	-0.950687	-0.914467	-1.451915
C	1.220165	-0.969305	-1.290198
C	1.286126	0.422643	-1.306393
C	2.380303	-1.735975	-1.171923
C	2.513071	1.073327	-1.202370
H	0.363605	0.978634	-1.384437
C	3.619927	-1.120368	-1.064792
H	2.280438	-2.809989	-1.154666
C	3.655026	0.279078	-1.085777
H	4.613702	0.768494	-0.991650
H	-0.588411	-1.515707	2.355186
C	-1.911975	-0.294238	1.113767
C	-2.778612	-1.378539	0.834234
C	-2.272448	0.960819	0.593180
C	-3.910945	-1.235126	0.051892
H	-2.507752	-2.347421	1.226625
C	-3.407192	1.135392	-0.200867
H	-1.648918	1.816229	0.813851
C	-4.215591	0.033361	-0.469527
H	-5.095005	0.149466	-1.081173
C	-3.732923	2.534993	-0.741063
C	-4.825407	-2.418984	-0.287562
C	2.646657	2.598276	-1.172635
C	4.923348	-1.902775	-0.884755
C	-3.954113	3.499743	0.438088
H	-3.068676	3.569201	1.069838
H	-4.185236	4.502120	0.070486
H	-4.786174	3.166065	1.060831
C	-2.555962	3.044091	-1.591509
H	-2.354718	2.366436	-2.423302
H	-2.782876	4.029944	-2.003443
H	-1.646300	3.130589	-1.000718
C	-4.993151	2.551407	-1.613181
H	-4.890000	1.904306	-2.485357
H	-5.876300	2.234915	-1.056743
H	-5.173191	3.566229	-1.971852
C	-6.260693	-2.115801	0.178852
H	-6.919893	-2.954051	-0.057574
H	-6.291478	-1.954065	1.258027
H	-6.663536	-1.226679	-0.305597
C	-4.823643	-2.635504	-1.812070
H	-5.467217	-3.477633	-2.076504
H	-5.186777	-1.755008	-2.341994
H	-3.815281	-2.852438	-2.168943
C	-4.374258	-3.723458	0.378368
H	-3.376146	-4.019204	0.053500
H	-4.369200	-3.641543	1.466329
H	-5.062798	-4.526066	0.108596
C	3.239339	3.023671	0.183532
H	3.354856	4.108595	0.219397
H	2.582469	2.727025	1.002596
H	4.218272	2.575753	0.354289
C	3.581415	3.057249	-2.305136
H	3.678984	4.144417	-2.292902
H	4.579629	2.631256	-2.204542
H	3.185089	2.762437	-3.278275
C	1.295116	3.296764	-1.345200
H	0.822401	3.037500	-2.293143
H	0.606894	3.041505	-0.540117
H	1.438708	4.377903	-1.328831
C	5.531878	-1.544930	0.483828
H	6.468593	-2.085623	0.632262
H	5.743510	-0.478424	0.562448
H	4.851702	-1.815776	1.292991
C	5.914152	-1.522560	-1.998788
H	6.152941	-0.459310	-1.982960
H	6.846761	-2.076278	-1.875690
H	5.503701	-1.761929	-2.981239

C	4.699672	-3.417357	-0.933111
H	4.036273	-3.754926	-0.135791
H	4.274020	-3.732422	-1.886862
H	5.654792	-3.929319	-0.808728

E-anti-TS0-tBu

Number of imaginary frequencies: 1
Electronic Energy (SCF) = -1801.10702953 a.u
ZPV corrected Energy = -1800.327501 a.u.
Enthalpy = -1800.286283 a.u.
Free Energy = -1800.400617 a.u.

S	0.251916	0.434137	1.501339
C	-0.592826	0.234968	3.133761
H	-1.640989	0.028482	2.936884
H	-0.502258	1.199522	3.631666
C	1.750147	-0.485300	2.031922
H	2.619544	0.068343	1.694856
H	1.713987	-1.441443	1.520365
C	1.641779	-0.636633	3.545521
H	1.997080	0.268387	4.041077
H	2.268668	-1.465624	3.872784
C	0.165533	-0.862036	3.865592
H	-0.153422	-1.841166	3.505227
H	-0.035657	-0.814713	4.935502
C	-0.524279	-0.667083	0.338054
C	0.260787	-0.664663	-1.459626
O	-0.061678	-1.771226	-1.990821
H	-0.274619	0.253364	-1.775369
C	1.714880	-0.348485	-1.199392
C	2.119368	0.973295	-1.027865
C	2.649679	-1.373621	-1.096219
C	3.439218	1.287410	-0.707880
H	1.379294	1.753176	-1.137776
C	3.978620	-1.101486	-0.780449
H	2.299797	-2.381654	-1.256756
C	4.345921	0.232763	-0.583418
H	5.370903	0.457241	-0.325637
H	-0.335168	-1.692155	0.641976
C	-1.950788	-0.355233	0.143003
C	-2.866118	-1.409071	0.009596
C	-2.411704	0.953429	-0.006412
C	-4.206235	-1.171404	-0.269887
H	-2.498249	-2.416981	0.121846
C	-3.754832	1.230328	-0.268560
H	-1.710109	1.772535	0.076551
C	-4.632462	0.157299	-0.397921
H	-5.673158	0.347534	-0.605238
C	-4.205301	2.688466	-0.412522
C	-5.223360	-2.304771	-0.442771
C	3.905679	2.723970	-0.449724
C	5.029515	-2.203515	-0.608957
C	-3.891371	3.450591	0.887794
H	-2.823970	3.445596	1.108739
H	-4.210823	4.491230	0.801933
H	-4.414089	3.003782	1.735575
C	-3.444068	3.344121	-1.579009
H	-3.645202	2.821337	-2.515541
H	-3.755553	4.384275	-1.695510
H	-2.366942	3.332947	-1.413121
C	-5.707146	2.812634	-0.689947
H	-5.989011	2.318412	-1.620590
H	-6.304674	2.386755	0.117386
H	-5.973598	3.866764	-0.780138
C	-6.349438	-2.148859	0.595189
H	-7.081221	-2.951247	0.480554
H	-5.951034	-2.195642	1.610486
H	-6.873300	-1.199773	0.482372
C	-5.825154	-2.232066	-1.858045
H	-6.551032	-3.035387	-2.000879
H	-6.335112	-1.284166	-2.029459
H	-5.046423	-2.338031	-2.615089
C	-4.592314	-3.689364	-0.261907
H	-3.802384	-3.871740	-0.991441
H	-4.170693	-3.815226	0.736523
H	-5.355749	-4.456747	-0.398733
C	4.344477	2.846029	1.021511
H	4.683493	3.862212	1.233721
H	3.514150	2.619706	1.693468
H	5.161991	2.162959	1.252898
C	5.096099	3.062243	-1.363715
H	5.429250	4.086614	-1.184943
H	5.943057	2.399959	-1.185798
H	4.814837	2.974790	-2.414540
C	2.796836	3.750284	-0.706277
H	2.444117	3.713910	-1.737903
H	1.940585	3.595867	-0.048325
H	3.180487	4.754487	-0.520015
C	5.560961	-2.170477	0.835999
H	6.309603	-2.951922	0.982422
H	6.025023	-1.212569	1.071301
H	4.752366	-2.337765	1.550319
C	6.195425	-1.963792	-1.584287
H	6.676530	-1.001600	-1.409287
H	6.951419	-2.743187	-1.467967
H	5.844969	-1.981814	-2.617675
C	4.457425	-3.599182	-0.878154
H	3.649498	-3.846260	-0.188102
H	4.073615	-3.689181	-1.895065
H	5.243253	-4.345303	-0.750371

E-anti-tBu

Number of imaginary frequencies: 0
Electronic Energy (SCF) = -1801.11310712 a.u
ZPV corrected Energy = -1800.331841 a.u.
Enthalpy = -1800.290370 a.u.
Free Energy = -1800.405750 a.u.

S	0.141924	0.398318	1.434676
C	-1.019830	-0.176600	2.733427
H	-2.014543	-0.224177	2.301240
H	-1.000767	0.594704	3.502068
C	1.508538	-0.696330	2.004717
H	2.405840	-0.091286	2.079892
H	1.648531	-1.437041	1.223901
C	1.049130	-1.312467	3.324162
H	1.291276	-0.644892	4.152168
H	1.573406	-2.253573	3.487250
C	-0.463382	-1.500211	3.235750
H	-0.708926	-2.298112	2.533707
H	-0.903261	-1.752028	4.200279
C	-0.446995	-0.430885	-0.160269
C	0.353793	0.047389	-1.446344
O	0.007914	-0.783972	-2.426042
H	0.043327	1.109467	-1.591255
C	1.850234	0.107101	-1.110861
C	2.424483	1.269378	-0.603704
C	2.636202	-1.029525	-1.269097
C	3.763419	1.305306	-0.210331
H	1.806364	2.151865	-0.517866
C	3.977342	-1.036675	-0.891599
H	2.160773	-1.898703	-1.697086
C	4.514145	0.138705	-0.357350
H	5.552103	0.147326	-0.056441
H	-0.221647	-1.484284	-0.011764
C	-1.923383	-0.200506	-0.255160

C	-2.798519	-1.281209	-0.129094
C	-2.435635	1.077694	-0.433372
C	-4.175375	-1.099803	-0.190195
H	-2.377821	-2.265319	0.010058
C	-3.813704	1.301899	-0.494137
H	-1.751783	1.908996	-0.530043
C	-4.658164	0.202482	-0.370902
H	-5.725006	0.351306	-0.417544
C	-4.335746	2.727374	-0.698786
C	-5.167446	-2.260596	-0.070804
C	4.417588	2.557907	0.383058
C	4.866929	-2.277697	-1.023624
C	-3.826279	3.626523	0.442386
H	-2.737333	3.664375	0.470070
H	-4.193042	4.646263	0.309912
H	-4.177203	3.262698	1.409971
C	-3.813663	3.268074	-2.042412
H	-4.160025	2.647640	-2.870497
H	-4.174405	4.285910	-2.204077
H	-2.724424	3.288224	-2.071741
C	-5.866405	2.793412	-0.715303
H	-6.288545	2.202334	-1.529104
H	-6.297893	2.440917	0.222814
H	-6.182786	3.827557	-0.858487
C	-6.123040	-2.000839	1.107789
H	-6.832175	-2.825274	1.204897
H	-5.570467	-1.916543	2.045499
H	-6.695031	-1.083505	0.969414
C	-5.984043	-2.363199	-1.371852
H	-6.696045	-3.188415	-1.305782
H	-6.545583	-1.449142	-1.565122
H	-5.329897	-2.544391	-2.226202
C	-4.466740	-3.602461	0.166166
H	-3.797518	-3.860343	-0.655473
H	-3.887203	-3.598640	1.090824
H	-5.214145	-4.392997	0.246332
C	4.906784	2.244870	1.808931
H	5.383538	3.123928	2.247671
H	4.071797	1.957697	2.451205
H	5.631571	1.430934	1.815285
C	5.618173	2.974687	-0.484681
H	6.093090	3.866483	-0.070195
H	6.370014	2.187414	-0.536239
H	5.298814	3.201708	-1.503233
C	3.447126	3.741507	0.460235
H	3.080265	4.028965	-0.525942
H	2.585822	3.518818	1.091989
H	3.959088	4.604224	0.889325
C	5.368624	-2.693553	0.371377
H	6.003319	-3.579153	0.297345
H	5.951768	-1.901941	0.841960
H	4.530849	-2.931620	1.029835
C	6.075478	-1.952580	-1.919563
H	6.679099	-1.145602	-1.504198
H	6.715820	-2.831044	-2.024018
H	5.748239	-1.650023	-2.915729
C	4.119322	-3.465424	-1.638537
H	3.268578	-3.769260	-1.027028
H	3.751970	-3.237039	-2.639614
H	4.794358	-4.319191	-1.716862

F-gauche-0-tBu
Number of imaginary frequencies: 0
Electronic Energy (SCF) = -1801.11985921 a.u
ZPV corrected Energy = -1800.341095 a.u.
Enthalpy = -1800.298541 a.u.
Free Energy = -1800.415263 a.u.

S	-1.046964	2.504716	0.809672
C	-0.943118	4.181737	1.659380
H	-0.115304	4.690457	1.168606
H	-0.697030	4.008362	2.704450
C	-2.592664	2.977963	-0.079676
H	-3.401319	2.466781	0.438391
H	-2.511982	2.594659	-1.090880
C	-2.728928	4.488956	0.021252
H	-3.758030	4.791460	-0.173281
H	-2.090969	4.970523	-0.722197
C	-2.276057	4.875065	1.430373
H	-2.175434	5.954956	1.544260
H	-3.010766	4.531318	2.161315
C	0.142979	2.305692	-0.358577
H	0.125869	2.981098	-1.202257
C	-1.185070	0.739283	-2.582952
H	-0.108502	0.707323	-2.814748
O	-1.907423	1.568348	-3.111545
C	1.309366	1.503624	-0.105976
C	2.215674	1.269295	-1.164126
C	1.616422	0.924229	1.143301
C	3.355886	0.493812	-1.000540
H	1.989350	1.706733	-2.125122
C	2.760366	0.157789	1.340099
H	0.954918	1.113159	1.974658
C	3.619852	-0.054110	0.259463
H	4.512555	-0.644042	0.401230
C	-1.643600	-0.315992	-1.667706
C	-0.718909	-1.269080	-1.231712
C	-2.967610	-0.373983	-1.245805
C	-1.108969	-2.288872	-0.375212
H	0.301977	-1.184963	-1.571988
C	-3.389729	-1.372498	-0.370386
H	-3.656776	0.374967	-1.607294
C	-2.446249	-2.312739	0.043553
H	-2.754576	-3.097501	0.715771
C	-4.847045	-1.402299	0.100330
C	-0.146177	-3.373085	0.114610
C	3.139904	-0.394387	2.721881
C	4.316812	0.198134	-2.159305
C	-5.173876	-0.076978	0.812334
H	-4.518873	0.073916	1.672222
H	-5.056993	0.775479	0.143605
H	-6.206222	-0.085218	1.167447
C	-5.771409	-1.562222	-1.119797
H	-5.563019	-2.495928	-1.645041
H	-6.815743	-1.576193	-0.801682
H	-5.646422	-0.742457	-1.827212
C	-5.125186	-2.553382	1.072302
H	-6.171898	-2.524259	1.377967
H	-4.941296	-3.525597	0.612783
H	-4.514023	-2.480109	1.973103
C	-0.642974	-4.747130	-0.370559
H	0.031588	-5.533559	-0.025914
H	-1.640625	-4.971354	0.007629
H	-0.678922	-4.784105	-1.460816
C	1.275866	-3.157009	-0.413271
H	1.308996	-3.201135	-1.502660
H	1.685951	-2.198500	-0.098614
H	1.930247	-3.941501	-0.031045
C	-0.108108	-3.363220	1.652825
H	0.590783	-4.118147	2.017332
H	0.214126	-2.391350	2.026227
H	-1.086874	-3.581354	2.080333
C	3.908523	0.898790	-3.459799
H	3.891850	1.983775	-3.347159
H	4.627560	0.657037	-4.244288
H	2.924418	0.576851	-3.802901
C	4.335526	-1.319673	-2.418957

H	5.022871	-1.556840	-3.234152
H	4.654208	-1.873340	-1.536119
H	3.342591	-1.676327	-2.697645
C	5.735797	0.666160	-1.790344
H	6.426968	0.460377	-2.610641
H	5.750909	1.740034	-1.595087
H	6.110093	0.158067	-0.902068
C	3.467730	-1.894806	2.633016
H	2.603864	-2.463582	2.292649
H	4.291836	-2.091872	1.948496
H	3.755529	-2.271872	3.616742
C	2.021604	-0.215675	3.755388
H	1.801750	0.836389	3.940363
H	1.099693	-0.706011	3.440155
H	2.328882	-0.658990	4.704054
C	4.386046	0.358935	3.224189
H	4.677324	-0.002818	4.213096
H	5.231362	0.219173	2.549970
H	4.188295	1.429866	3.297983

F-gauche-0-tBu
Number of imaginary frequencies: 0
Electronic Energy (SCF) = -1801.11769404 a.u
ZPV corrected Energy = -1800.339369 a.u.
Enthalpy = -1800.296615 a.u.
Free Energy = -1800.416554 a.u.

S	-3.203429	1.052115	-0.025493
C	-4.012776	2.319725	1.121384
H	-3.311324	3.150974	1.143748
H	-4.084273	1.877846	2.112594
C	-4.539610	1.226717	-1.268147
H	-5.226429	0.400903	-1.092474
H	-4.087264	1.103659	-2.244728
C	-5.204557	2.570298	-1.011058
H	-6.166683	2.618058	-1.521677
H	-4.578996	3.377169	-1.396833
C	-5.356301	2.691058	0.504814
H	-5.654082	3.696292	0.805189
H	-6.127684	2.000251	0.849980
C	-1.842048	1.692205	-0.807133
H	-2.050574	2.464305	-1.537211
C	-1.894854	-0.780760	-2.637019
H	-1.477368	0.016131	-3.274353
O	-3.075386	-1.081806	-2.714320
C	-0.575247	1.721375	-0.125179
C	0.507885	2.409911	-0.714682
C	-0.347781	1.117928	1.129608
C	1.744397	2.514651	-0.089390
H	0.341310	2.880091	-1.672962
C	0.880336	1.200087	1.775574
H	-1.165081	0.602746	1.610814
C	1.919378	1.898851	1.154744
H	2.870382	1.985020	1.657196
C	-0.904133	-1.481098	-1.806289
C	0.451491	-1.231841	-2.032337
C	-1.298384	-2.416820	-0.856659
C	1.424908	-1.940005	-1.340940
H	0.719280	-0.491863	-2.771790
C	-0.349119	-3.116718	-0.113846
H	-2.355314	-2.590884	-0.715531
C	0.997169	-2.864753	-0.379501
H	1.747979	-3.408264	0.171740
C	-0.806205	-4.153765	0.917433
C	2.922273	-1.765624	-1.608426
C	1.114785	0.564677	3.153553
C	2.888264	3.349064	-0.684781
C	-1.783724	-3.503740	1.911237
H	-1.299431	-2.690402	2.451417
H	-2.663263	-3.099156	1.411395
H	-2.122862	-4.242286	2.640201
C	-1.522219	-5.301564	0.182299
H	-0.851556	-5.784520	-0.530568
H	-1.860390	-6.055099	0.896655
H	-2.393223	-4.941103	-0.365507
C	0.363848	-4.741141	1.713743
H	-0.018204	-5.451562	2.448187
H	1.066000	-5.275478	1.072449
H	0.913046	-3.967353	2.252286
C	3.519455	-3.116154	-2.044152
H	4.586947	-3.006187	-2.245767
H	3.401356	-3.876282	-1.271786
H	3.037434	-3.480433	-2.953217
C	3.190802	-0.743732	-2.717660
H	2.751876	-1.055989	-3.666607
H	2.794680	0.239040	-2.465308
H	4.266529	-0.641983	-2.866782
C	3.621508	-1.293426	-0.323071
H	4.688402	-1.149973	-0.506101
H	3.201384	-0.350134	0.021224
H	3.514187	-2.022728	0.479286
C	2.612907	3.775861	-2.131386
H	1.739963	4.424839	-2.207234
H	3.469700	4.331482	-2.516224
H	2.454141	2.913846	-2.781196
C	4.208803	2.560625	-0.670420
H	5.013195	3.178404	-1.075273
H	4.497242	2.261735	0.336421
H	4.134314	1.661461	-1.280142
C	3.058786	4.618647	0.171147
H	3.858623	5.245112	-0.230686
H	2.138921	5.206126	0.181823
H	3.309876	4.368443	1.202281
C	2.105499	-0.603482	3.007360
H	1.713472	-1.357201	2.324226
H	3.064450	-0.263298	2.617533
H	2.280984	-1.076388	3.976490
C	-0.176439	0.024199	3.777688
H	-0.920026	0.811513	3.911895
H	-0.621149	-0.763741	3.171608
H	0.043226	-0.399023	4.759278
C	1.702529	1.606409	4.122209
H	1.850385	1.159497	5.107666
H	2.665824	1.983334	3.780964
H	1.028257	2.457657	4.233560

F-gauche-TS0-tBu
Number of imaginary frequencies: 1
Electronic Energy (SCF) = -1801.11300767 a.u
ZPV corrected Energy = -1800.333327 a.u.
Enthalpy = -1800.292262 a.u.
Free Energy = -1800.406226 a.u.

S	-1.057915	2.391836	0.666470
C	-0.316475	3.700220	1.754718
H	0.692476	3.858867	1.381946
H	-0.272643	3.310338	2.767999
C	-2.225747	3.542319	-0.144107
H	-3.189177	3.358933	0.327697
H	-2.256113	3.250587	-1.195741
C	-1.704129	4.941917	0.149742
H	-2.496559	5.674521	-0.001680
H	-0.887095	5.185726	-0.530694
C	-1.206048	4.930035	1.594642
H	-0.642999	5.829232	1.843462
H	-2.054275	4.866752	2.277917

C	0.106968	2.054129	-0.613304
H	0.366427	2.974094	-1.128398
C	-0.864320	1.211088	-2.117185
H	0.095726	0.930820	-2.588352
O	-1.597953	2.072246	-2.704709
C	1.269242	1.261518	-0.180784
C	2.358138	1.173648	-1.060627
C	1.323986	0.544456	1.019853
C	3.466660	0.386652	-0.769021
H	2.311162	1.728909	-1.985158
C	2.436710	-0.222562	1.361400
H	0.489004	0.592872	1.701420
C	3.490514	-0.294097	0.451502
H	4.357417	-0.887482	0.701039
C	-1.497657	0.011641	-1.458384
C	-0.751015	-1.148822	-1.241863
C	-2.824377	0.053819	-1.055759
C	-1.314115	-2.259561	-0.625395
H	0.276258	-1.164308	-1.572498
C	-3.422098	-1.034381	-0.413587
H	-3.387320	0.953345	-1.260022
C	-2.651560	-2.176567	-0.212598
H	-3.095685	-3.034310	0.267067
C	-4.882625	-0.932126	0.038669
C	-0.535642	-3.560377	-0.403347
C	2.554774	-0.933078	2.715653
C	4.644815	0.235719	-1.737544
C	-5.029674	0.254683	1.008549
H	-4.390584	0.122372	1.883564
H	-4.757366	1.196124	0.531785
H	-6.063274	0.338451	1.350934
C	-5.781631	-0.695038	-1.188047
H	-5.701275	-1.524168	-1.893240
H	-6.826282	-0.609497	-0.880995
H	-5.509154	0.219975	-1.713739
C	-5.366948	-2.198093	0.753106
H	-6.405797	-2.067746	1.060255
H	-5.321148	-3.072675	0.102673
H	-4.779330	-2.407867	1.648188
C	-1.193604	-4.689256	-1.217391
H	-0.652487	-5.626785	-1.071364
H	-2.229198	-4.847849	-0.915484
H	-1.185380	-4.454388	-2.283230
C	0.928230	-3.438965	-0.841928
H	1.013483	-3.242644	-1.911543
H	1.446856	-2.641808	-0.310369
H	1.450108	-4.374495	-0.634515
C	-0.567780	-3.933918	1.089233
H	0.010089	-4.843858	1.263457
H	-0.139235	-3.138076	1.698033
H	-1.584730	-4.113365	1.437922
C	4.458727	1.055985	-3.018438
H	4.380115	2.123674	-2.808281
H	5.320005	0.909000	-3.671766
H	3.568263	0.750430	-3.569298
C	4.780762	-1.246876	-2.131103
H	5.609383	-1.377516	-2.830220
H	4.971120	-1.876972	-1.262400
H	3.869069	-1.604739	-2.612662
C	5.942111	0.700244	-1.052092
H	6.787532	0.594222	-1.735017
H	5.871957	1.749440	-0.759260
H	6.158535	0.114878	-0.158666
C	2.910437	-2.416348	2.515308
H	2.139856	-2.930326	1.942813
H	3.857637	-2.539503	1.991269
H	3.000371	-2.911557	3.484217
C	1.258222	-0.856032	3.529720
H	0.997458	0.172849	3.781711
H	0.417303	-1.298696	2.994819
H	1.383535	-1.402191	4.465767
C	3.676049	-0.255745	3.525925
H	3.781606	-0.735061	4.501621
H	4.634208	-0.324135	3.010275
H	3.455032	0.800869	3.687784

F-gauche-tBu

Number of imaginary frequencies: 0
Electronic Energy (SCF) = -1801.12393627 a.u
ZPV corrected Energy = -1800.341631 a.u.
Enthalpy = -1800.300626 a.u.
Free Energy = -1800.413382 a.u.

S	1.083668	3.182834	-0.284997
C	2.512757	3.051169	0.895174
H	3.144194	2.216191	0.601392
H	2.110486	2.859531	1.885923
C	1.926907	4.471895	-1.286163
H	1.302699	5.356211	-1.172987
H	1.871053	4.143815	-2.317109
C	3.329626	4.664611	-0.725994
H	3.684057	5.673483	-0.936735
H	4.018185	3.962062	-1.198676
C	3.251371	4.376602	0.771600
H	4.236231	4.312112	1.233974
H	2.697279	5.170275	1.277733
C	1.252602	1.596502	-1.219736
H	2.104128	1.758579	-1.878361
C	-0.065258	1.706529	-2.093504
H	0.210383	1.239669	-3.059933
O	-0.329479	3.034073	-2.179995
C	1.499517	0.404437	-0.351154
C	2.368622	-0.585233	-0.804424
C	0.839233	0.232652	0.864280
C	2.576703	-1.752737	-0.070063
H	2.871123	-0.433639	-1.748186
C	1.055464	-0.897155	1.647150
H	0.150705	0.993838	1.195457
C	1.918735	-1.877638	1.153401
H	2.088850	-2.764930	1.745834
C	-1.185334	0.845282	-1.518243
C	-1.216479	-0.526497	-1.774673
C	-2.163613	1.408006	-0.718355
C	-2.194720	-1.342860	-1.222901
H	-0.452252	-0.943714	-2.414329
C	-3.165411	0.622584	-0.134602
H	-2.131868	2.477431	-0.570607
C	-3.153635	-0.745625	-0.390477
H	-3.914733	-1.372026	0.047804
C	-4.254983	1.293076	0.710729
C	-2.262759	-2.849538	-1.498565
C	0.414004	-1.074615	3.027620
C	3.499841	-2.877280	-0.550586
C	-3.621739	2.197568	1.782339
H	-3.006488	1.617081	2.470233
H	-2.995011	2.971773	1.341342
H	-4.403502	2.691986	2.362643
C	-5.129758	2.157228	-0.216835
H	-5.600034	1.544504	-0.988003
H	-5.918867	2.651239	0.354689
H	-4.538334	2.926856	-0.713501
C	-5.155779	0.276146	1.420249
H	-5.888678	0.803552	2.033004
H	-5.705529	-0.345611	0.712747
H	-4.581696	-0.380145	2.076534
C	-3.637357	-3.203571	-2.094101
H	-3.690823	-4.273041	-2.308851

H	-4.449036	-2.959756	-1.408820
H	-3.808546	-2.660821	-3.025467
C	-1.182317	-3.308461	-2.483898
H	-1.281640	-2.815143	-3.451923
H	-0.180062	-3.113113	-2.102264
H	-1.271308	-4.383770	-2.646874
C	-2.066707	-3.622387	-0.183118
H	-2.143623	-4.697391	-0.361137
H	-1.083434	-3.417521	0.239987
H	-2.817951	-3.349864	0.558173
C	4.122809	-2.576295	-1.917740
H	4.740694	-1.677465	-1.893578
H	4.761053	-3.408967	-2.216625
H	3.362123	-2.447427	-2.689004
C	2.691735	-4.182354	-0.666748
H	3.337798	-4.996034	-1.002452
H	2.255765	-4.472148	0.289093
H	1.879548	-4.073438	-1.386921
C	4.637955	-3.074174	0.466996
H	5.304598	-3.873445	0.137008
H	5.227362	-2.161432	0.571630
H	4.254601	-3.342395	1.451384
C	-0.346688	-2.409235	3.094371
H	-1.161885	-2.424084	2.371948
H	0.302391	-3.260443	2.890780
H	-0.772089	-2.545260	4.090680
C	-0.577400	0.046715	3.351452
H	-0.096419	1.025360	3.377213
H	-1.384755	0.082595	2.621074
H	-1.020505	-0.129786	4.332585
C	1.526653	-1.070386	4.091747
H	1.096220	-1.185225	5.088727
H	2.233109	-1.885684	3.933491
H	2.084005	-0.132096	4.067799

F-gauche'-0-tBu
Number of imaginary frequencies: 0
Electronic Energy (SCF) = -1801.11949419 a.u
ZPV corrected Energy = -1800.340974 a.u.
Enthalpy = -1800.298247 a.u.
Free Energy = -1800.415757 a.u.

S	4.459077	-0.512800	-0.309691
C	5.964514	-1.278189	0.451769
H	5.598365	-1.820033	1.321816
H	6.612473	-0.469497	0.780120
C	4.501474	-1.716001	-1.746091
H	4.899046	-1.154557	-2.591784
H	3.472429	-1.989895	-1.952176
C	5.419167	-2.852710	-1.337581
H	5.761777	-3.417318	-2.205019
H	4.890051	-3.543482	-0.677363
C	6.583620	-2.202754	-0.583974
H	7.221464	-2.941635	-0.098736
H	7.202119	-1.633297	-1.280377
C	3.196046	-0.777811	0.751076
H	3.110864	-1.760218	1.196496
C	0.757748	-1.101126	-1.793849
H	1.291349	-1.932820	-1.300154
O	1.343753	-0.366382	-2.563755
C	-0.667995	-1.014232	-1.450438
C	-1.237075	-2.066995	-0.735269
C	-1.441185	0.077481	-1.847073
C	-2.595059	-2.063771	-0.429517
H	-0.599897	-2.886704	-0.436411
C	-2.792745	0.127608	-1.533132
H	-0.957242	0.874317	-2.388909
C	-3.339954	-0.955236	-0.834611
H	-4.392802	-0.928939	-0.592668
C	2.157710	0.198098	0.968705
C	1.129405	-0.103577	1.892648
C	2.088972	1.445164	0.325811
C	0.105642	0.787569	2.175817
H	1.164281	-1.064870	2.384526
C	1.071825	2.360104	0.587302
H	2.842242	1.694022	-0.407550
C	0.087047	2.024717	1.515116
H	-0.708584	2.719239	1.727519
C	-0.991855	0.474622	3.201104
C	1.072519	3.705107	-0.152693
C	-3.690017	1.308553	-1.913275
C	-3.287179	-3.220887	0.297691
C	-2.297101	-4.310880	0.720622
H	-1.796403	-4.760430	-0.137976
H	-1.534231	-3.922182	1.396759
H	-2.832533	-5.104089	1.244050
C	-3.998385	-2.701993	1.559113
H	-4.733737	-1.933169	1.323374
H	-4.520187	-3.522587	2.055149
H	-3.282831	-2.279546	2.263560
C	-4.328179	-3.847113	-0.648212
H	-4.831919	-4.680965	-0.155224
H	-5.087254	-3.121974	-0.942543
H	-3.852525	-4.224262	-1.555232
C	-4.850755	0.807895	-2.791114
H	-5.461473	0.069109	-2.272122
H	-5.498026	1.642248	-3.068076
H	-4.474580	0.350577	-3.707952
C	-4.255648	1.945520	-0.631207
H	-4.908976	2.782462	-0.885507
H	-4.836001	1.230968	-0.047528
H	-3.450234	2.320764	0.000440
C	-2.926924	2.386919	-2.688462
H	-2.108786	2.802180	-2.100133
H	-2.514226	2.000390	-3.621451
H	-3.605703	3.203767	-2.937567
C	-0.846688	1.431391	4.398823
H	-0.935437	2.472429	4.087560
H	-1.623301	1.232933	5.141352
H	0.124652	1.305955	4.880849
C	-2.376321	0.679355	2.561530
H	-3.163247	0.419452	3.272869
H	-2.530389	1.714335	2.260245
H	-2.493368	0.053530	1.678455
C	-0.914264	-0.963748	3.724871
H	-1.001339	-1.691868	2.918141
H	0.020486	-1.152995	4.254112
H	-1.731741	-1.141484	4.425555
C	2.326989	4.502007	0.249002
H	2.348374	5.464780	-0.266989
H	2.337871	4.692009	1.323957
H	3.239118	3.962227	-0.005964
C	1.094806	3.465270	-1.672664
H	1.984370	2.919304	-1.983709
H	0.223569	2.890633	-1.987009
H	1.083324	4.419353	-2.204503
C	-0.160593	4.555310	0.173672
H	-0.210745	4.809319	1.233239
H	-0.117972	5.489509	-0.388883
H	-1.085183	4.044859	-0.098583

F-gauche'-TS0-tBu
Number of imaginary frequencies: 1
Electronic Energy (SCF) = -1801.11207401 a.u
ZPV corrected Energy = -1800.332610 a.u.
Enthalpy = -1800.291478 a.u.

Free Energy = -1800.403993 a.u.

S	3.604350	-1.115382	-0.754194
C	4.730661	-0.943373	0.705571
H	4.087053	-0.863203	1.577744
H	5.291963	-0.020543	0.586649
C	4.092221	-2.846733	-1.076880
H	4.795884	-2.804457	-1.906029
H	3.207690	-3.391133	-1.389231
C	4.745313	-3.349953	0.203557
H	5.339347	-4.239775	-0.003378
H	3.979775	-3.618351	0.932967
C	5.600960	-2.198894	0.728556
H	5.963419	-2.382583	1.739530
H	6.468720	-2.060423	0.081908
C	1.969136	-1.254457	-0.097029
H	1.918417	-2.069317	0.619586
C	1.016827	-1.871999	-1.794202
H	1.205833	-2.938468	-1.559991
O	1.589473	-1.359258	-2.803134
C	-0.395693	-1.510672	-1.435680
C	-1.133432	-2.331339	-0.585597
C	-0.974695	-0.357258	-1.953947
C	-2.449547	-2.022467	-0.247857
H	-0.660916	-3.224250	-0.199856
C	-2.285395	-0.009050	-1.634368
H	-0.374226	0.246935	-2.614854
C	-2.998893	-0.854125	-0.781895
H	-4.018813	-0.599150	-0.533191
C	1.449590	0.019673	0.408557
C	0.623072	0.013507	1.540196
C	1.679921	1.238386	-0.245325
C	0.031640	1.178945	2.015850
H	0.449248	-0.929861	2.034921
C	1.117517	2.426300	0.215808
H	2.296449	1.243690	-1.130267
C	0.294722	2.374135	1.342115
H	-0.148341	3.287423	1.709626
C	-0.899995	1.186152	3.233073
C	1.413247	3.778688	-0.444932
C	-2.951127	1.263353	-2.169191
C	-3.315312	-2.936067	0.628150
C	-2.505614	-4.076536	1.255320
H	-2.079067	-4.736970	0.499592
H	-1.691669	-3.698019	1.875848
H	-3.156662	-4.679627	1.890125
C	-3.965230	-2.132055	1.767132
H	-4.602624	-1.332992	1.390569
H	-4.584959	-2.789035	2.380912
H	-3.208368	-1.683316	2.409966
C	-4.424168	-3.550404	-0.246439
H	-5.057078	-4.211918	0.349458
H	-5.058037	-2.777244	-0.681591
H	-3.995842	-4.133325	-1.063703
C	-4.278390	0.911658	-2.863840
H	-4.982772	0.439744	-2.179287
H	-4.749056	1.816440	-3.254418
H	-4.110791	0.228898	-3.698845
C	-3.231902	2.210934	-0.988757
H	-3.687833	3.137450	-1.345266
H	-3.911793	1.755433	-0.268638
H	-2.309026	2.461205	-0.466563
C	-2.065305	1.994816	-3.183323
H	-1.114486	2.298134	-2.747536
H	-1.854396	1.371928	-4.053971
H	-2.574867	2.895096	-3.530622
C	-0.432158	2.239260	4.252681
H	-0.443207	3.245201	3.834502
H	-1.090603	2.234587	5.123753
H	0.582843	2.026920	4.593874
C	-2.323212	1.534281	2.760444
H	-3.015539	1.530698	3.605372
H	-2.356031	2.521343	2.299418
H	-2.673996	0.809490	2.026324
C	-0.940726	-0.171275	3.943165
H	-1.304314	-0.962018	3.288407
H	0.044560	-0.460915	4.312602
H	-1.615204	-0.114669	4.798864
C	2.279026	4.613242	0.517671
H	2.514005	5.583263	0.073854
H	1.763120	4.789216	1.462157
H	3.218664	4.102787	0.736871
C	2.169208	3.626861	-1.769757
H	3.150058	3.170930	-1.630130
H	1.611387	3.019873	-2.484128
H	2.324062	4.610988	-2.214709
C	0.106646	4.539605	-0.727588
H	-0.457361	4.735968	0.183421
H	0.330664	5.501947	-1.192187
H	-0.534167	3.977325	-1.405690

F-gauche'-tBu
Number of imaginary frequencies: 0
Electronic Energy (SCF) = -1801.12276827 a.u
ZPV corrected Energy = -1800.340601 a.u.
Enthalpy = -1800.299617 a.u.
Free Energy = -1800.411249 a.u.

S	3.560775	-1.119488	-0.877175
C	4.525767	-1.176501	0.705604
H	3.812863	-1.206911	1.525433
H	5.100369	-0.256717	0.771291
C	4.030119	-2.824492	-1.348387
H	4.795766	-2.701792	-2.112815
H	3.163170	-3.298853	-1.792738
C	4.571635	-3.494926	-0.094115
H	5.171514	-4.364426	-0.362432
H	3.745213	-3.837668	0.530401
C	5.389208	-2.435790	0.640878
H	5.670891	-2.752108	1.644829
H	6.306612	-2.229182	0.087058
C	1.850793	-1.284383	-0.225727
H	1.862200	-2.052557	0.545731
C	1.080490	-1.746826	-1.535198
H	1.169023	-2.859074	-1.509199
O	1.670663	-1.178140	-2.599121
C	-0.406598	-1.467102	-1.322569
C	-1.160083	-2.278686	-0.478053
C	-0.996941	-0.361038	-1.917241
C	-2.495287	-1.990545	-0.200655
H	-0.682256	-3.140389	-0.032184
C	-2.330123	-0.036349	-1.665731
H	-0.377389	0.234539	-2.568290
C	-3.054698	-0.862041	-0.804717
H	-4.087928	-0.621541	-0.598965
C	1.426146	0.028079	0.359289
C	0.719950	0.023197	1.560316
C	1.632935	1.231764	-0.313729
C	0.194639	1.199368	2.090604
H	0.562729	-0.920947	2.058875
C	1.147791	2.431927	0.202450
H	2.152480	1.210905	-1.257255
C	0.430091	2.388125	1.398352
H	0.038685	3.309812	1.803688
C	-0.649809	1.219252	3.368263
C	1.394330	3.779496	-0.484630
C	-3.000399	1.204802	-2.264673

C	-3.360624	-2.863441	0.715835
C	-2.567909	-4.016396	1.341191
H	-2.167205	-4.691331	0.583795
H	-1.736867	-3.652589	1.947508
H	-3.223119	-4.599079	1.990690
C	-3.939391	-2.008314	1.857049
H	-4.554438	-1.192043	1.479403
H	-4.563533	-2.623445	2.508866
H	-3.141709	-1.575322	2.461334
C	-4.519513	-3.460971	-0.102552
H	-5.149904	-4.087779	0.532135
H	-5.146048	-2.679872	-0.533387
H	-4.140201	-4.076739	-0.920004
C	-4.294669	0.808121	-2.996027
H	-5.014536	0.341124	-2.324205
H	-4.768078	1.692079	-3.428825
H	-4.083810	0.106169	-3.804881
C	-3.341628	2.180412	-1.122870
H	-3.800554	3.088263	-1.521064
H	-4.038650	1.732732	-0.413929
H	-2.442762	2.463735	-0.574952
C	-2.091156	1.925368	-3.265870
H	-1.166067	2.265381	-2.802205
H	-1.830100	1.280684	-4.106448
H	-2.606605	2.801740	-3.662355
C	-0.064802	2.226449	4.373060
H	-0.051780	3.239121	3.971181
H	-0.665321	2.238113	5.284825
H	0.957872	1.957246	4.643745
C	-2.084004	1.642498	2.999002
H	-2.718703	1.646550	3.887747
H	-2.103045	2.642557	2.565504
H	-2.515641	0.953345	2.272653
C	-0.708433	-0.153243	4.047109
H	-1.157609	-0.906608	3.400408
H	0.284045	-0.500839	4.339086
H	-1.317402	-0.086985	4.949777
C	2.271612	4.649874	0.434134
H	2.465232	5.617742	-0.033133
H	1.786704	4.829628	1.394073
H	3.231417	4.166802	0.626345
C	2.108918	3.621590	-1.831015
H	3.094879	3.169100	-1.717341
H	1.532237	3.008204	-2.524426
H	2.245591	4.603244	-2.287037
C	0.057649	4.499530	-0.733418
H	-0.484095	4.684977	0.193613
H	0.237513	5.463923	-1.212715
H	-0.585406	3.911150	-1.387165

F-anti-0-tBu
Number of imaginary frequencies: 0
Electronic Energy (SCF) = -1801.12070052 a.u
ZPV corrected Energy = -1800.342174 a.u.
Enthalpy = -1800.299382 a.u.
Free Energy = -1800.417486 a.u.

S	0.490219	0.553636	2.431700
C	0.779726	0.661882	4.270091
H	0.159337	-0.122943	4.699108
H	0.416726	1.631129	4.603314
C	2.173992	-0.190024	2.150393
H	2.788432	0.600658	1.726150
H	2.037785	-0.972434	1.412407
C	2.693057	-0.646808	3.501700
H	3.775750	-0.771775	3.482415
H	2.250086	-1.608195	3.769349
C	2.261444	0.424356	4.507723
H	2.439836	0.113736	5.537389
H	2.825767	1.342932	4.334270
C	-0.697255	-0.576476	2.118896
H	-0.555826	-1.572457	2.517371
C	-0.246720	-1.543644	-1.470846
H	-1.111626	-0.861286	-1.498712
O	-0.407526	-2.746426	-1.548242
C	-1.829712	-0.307731	1.272806
C	-2.669607	-1.385571	0.917852
C	-2.166258	0.964949	0.763407
C	-3.761968	-1.226825	0.075593
H	-2.417071	-2.363583	1.299824
C	-3.258720	1.153947	-0.075511
H	-1.553608	1.808848	1.040237
C	-4.042451	0.048877	-0.423307
H	-4.886531	0.185885	-1.082357
C	1.063919	-0.882812	-1.352393
C	1.113647	0.513022	-1.303836
C	2.233309	-1.632994	-1.290435
C	2.333281	1.171355	-1.211690
H	0.182993	1.059488	-1.338193
C	3.473215	-1.006719	-1.184225
H	2.153618	-2.710073	-1.319590
C	3.492927	0.387269	-1.155192
H	4.443467	0.890838	-1.079452
C	4.744884	-1.853013	-1.075536
C	2.456148	2.697560	-1.176412
C	-3.633543	2.534473	-0.632809
C	-4.654814	-2.404580	-0.336862
C	4.668063	-2.699016	0.208811
H	4.608384	-2.060326	1.091520
H	3.795990	-3.352948	0.205576
H	5.558306	-3.324132	0.301376
C	4.847368	-2.787112	-2.293993
H	4.892524	-2.212787	-3.220935
H	5.751972	-3.394273	-2.225552
H	3.995244	-3.463427	-2.357137
C	6.014311	-0.997286	-1.015413
H	6.886534	-1.648543	-0.946138
H	6.129769	-0.380885	-1.908217
H	6.021650	-0.341342	-0.143597
C	3.269815	3.165871	-2.396312
H	3.358262	4.253994	-2.393151
H	4.276613	2.747620	-2.393264
H	2.782306	2.866639	-3.325677
C	1.087900	3.382958	-1.209810
H	0.534556	3.142867	-2.118265
H	0.478774	3.096435	-0.353161
H	1.219810	4.465180	-1.177342
C	3.178010	3.133953	0.111132
H	3.290132	4.219677	0.124990
H	2.608193	2.844307	0.994880
H	4.172755	2.695638	0.189108
C	-4.222290	-3.727602	0.304326
H	-4.262196	-3.681345	1.393610
H	-4.894180	-4.524477	-0.019183
H	-3.210021	-4.007553	0.011242
C	-4.598622	-2.573311	-1.866140
H	-5.230994	-3.407434	-2.178708
H	-4.945406	-1.677433	-2.380898
H	-3.578702	-2.778379	-2.195304
C	-6.107411	-2.120955	0.086734
H	-6.754323	-2.953742	-0.198220
H	-6.177301	-1.991673	1.168431
H	-6.495410	-1.218373	-0.384817
C	-3.504687	2.513247	-2.166882
H	-2.481347	2.281376	-2.467282
H	-4.161902	1.766584	-2.612402
H	-3.768217	3.487628	-2.584565

C	-2.739081	3.654986	-0.092553
H	-2.795172	3.727786	0.994599
H	-1.695990	3.511483	-0.370718
H	-3.062954	4.610944	-0.507598
C	-5.087966	2.868178	-0.253828
H	-5.362121	3.852176	-0.640640
H	-5.789943	2.141329	-0.660862
H	-5.211505	2.883449	0.830634

F-anti-TS0-tBu
Number of imaginary frequencies: 1
Electronic Energy (SCF) = -1801.10654398 a.u
ZPV corrected Energy = -1800.327220 a.u.
Enthalpy = -1800.285956 a.u.
Free Energy = -1800.400550 a.u.

S	0.235332	0.664636	1.466037
C	-0.666223	0.516258	3.083760
H	-1.449640	-0.219147	2.918925
H	-1.116017	1.480404	3.303969
C	1.745406	-0.148796	2.102939
H	2.451434	0.654756	2.299755
H	2.137521	-0.782635	1.315990
C	1.331905	-0.874251	3.374942
H	2.212621	-1.107573	3.972739
H	0.835971	-1.813192	3.124793
C	0.368057	0.056275	4.106635
H	-0.129347	-0.437909	4.940933
H	0.910886	0.916410	4.501572
C	-0.474021	-0.536993	0.357464
H	-0.241816	-1.533126	0.724057
C	0.303907	-0.616716	-1.460320
H	-0.209145	0.303786	-1.804079
O	-0.050113	-1.732364	-1.945957
C	-1.912943	-0.298557	0.149540
C	-2.787394	-1.387856	0.092179
C	-2.420939	0.986431	-0.084486
C	-4.140666	-1.217520	-0.194479
H	-2.384139	-2.372685	0.268970
C	-3.769553	1.194273	-0.355784
H	-1.743714	1.827128	-0.061459
C	-4.609878	0.079163	-0.407301
H	-5.658171	0.225923	-0.622254
C	1.762677	-0.337138	-1.198048
C	2.205460	0.978607	-1.041915
C	2.664040	-1.381426	-1.064431
C	3.525837	1.257550	-0.711377
H	1.489471	1.777317	-1.174121
C	4.002902	-1.143470	-0.736308
H	2.292167	-2.386159	-1.206705
C	4.406668	0.177111	-0.556795
H	5.431794	0.383995	-0.293715
C	4.957514	-2.328647	-0.559191
C	4.035297	2.683995	-0.481241
C	-4.351325	2.590833	-0.602228
C	-5.115927	-2.396791	-0.284582
C	4.444733	-3.218750	0.588272
H	4.409997	-2.661042	1.526264
H	3.444060	-3.598667	0.383180
H	5.107844	-4.075042	0.728092
C	4.998129	-3.151880	-1.858754
H	5.359858	-2.544795	-2.690503
H	5.669234	-4.005547	-1.743407
H	4.012324	-3.532446	-2.124435
C	6.386386	-1.888777	-0.223966
H	7.021540	-2.769197	-0.115182
H	6.813280	-1.266439	-1.011795
H	6.430629	-1.331049	0.712717
C	5.235258	2.966350	-1.402219
H	5.598635	3.984083	-1.245867
H	6.062396	2.283446	-1.209692
H	4.950938	2.864424	-2.450939
C	2.958486	3.738065	-0.760264
H	2.606922	3.692609	-1.791897
H	2.096562	3.622495	-0.101815
H	3.371982	4.733641	-0.592503
C	4.478489	2.824537	0.986851
H	4.848245	3.834403	1.176372
H	3.641970	2.637759	1.663099
H	5.274989	2.122505	1.233959
C	-4.435607	-3.741288	-0.005698
H	-4.010749	-3.779701	0.998568
H	-5.170932	-4.543381	-0.085692
H	-3.639057	-3.947491	-0.721498
C	-5.717205	-2.446911	-1.700950
H	-6.414328	-3.283256	-1.785770
H	-6.259174	-1.531767	-1.939182
H	-4.933681	-2.578973	-2.448879
C	-6.248271	-2.208010	0.740799
H	-6.950807	-3.042120	0.684455
H	-5.849919	-2.168059	1.756407
H	-6.805456	-1.288556	0.561696
C	-4.993712	2.638890	-2.000242
H	-4.254329	2.425211	-2.773944
H	-5.801264	1.913524	-2.097265
H	-5.408939	3.630834	-2.190250
C	-3.285491	3.689566	-0.527532
H	-2.811255	3.729073	0.454336
H	-2.506126	3.549124	-1.277730
H	-3.750624	4.659466	-0.709723
C	-5.425533	2.889435	0.459349
H	-5.849704	3.882773	0.298520
H	-6.240493	2.166719	0.421033
H	-4.997639	2.861507	1.463247

F-anti-tBu
Number of imaginary frequencies: 0
Electronic Energy (SCF) = -1801.11316289 a.u
ZPV corrected Energy = -1800.332247 a.u.
Enthalpy = -1800.290670 a.u.
Free Energy = -1800.407134 a.u.

S	0.165261	0.586282	1.407644
C	-1.002801	0.120985	2.757027
H	-1.739236	-0.547209	2.318862
H	-1.498849	1.028692	3.088203
C	1.544973	-0.409642	2.092561
H	2.216415	0.305484	2.562968
H	2.055998	-0.886011	1.263181
C	0.916703	-1.361274	3.099498
H	1.679648	-1.741551	3.778165
H	0.472051	-2.212118	2.581165
C	-0.160133	-0.563938	3.829591
H	-0.797034	-1.196281	4.447415
H	0.303455	0.182024	4.476704
C	-0.407554	-0.395915	-0.101912
H	-0.160627	-1.427814	0.139317
C	0.367228	-0.016477	-1.438327
H	0.057552	1.034257	-1.653702
O	-0.002111	-0.916957	-2.344991
C	-1.888918	-0.202472	-0.209148
C	-2.740477	-1.292913	-0.057412
C	-2.422947	1.063664	-0.443194
C	-4.123914	-1.143081	-0.151143
H	-2.302685	-2.262552	0.124080
C	-3.799231	1.254391	-0.533779

H	-1.749901	1.898515	-0.563475
C	-4.624465	0.137255	-0.386900
H	-5.693901	0.270301	-0.461479
C	1.868225	0.053977	-1.133713
C	2.458182	1.242425	-0.699295
C	2.640158	-1.093139	-1.219460
C	3.793415	1.286411	-0.308976
H	1.849158	2.134804	-0.668582
C	3.986220	-1.094208	-0.839791
H	2.158063	-1.989183	-1.584593
C	4.536388	0.100758	-0.380715
H	5.571634	0.123066	-0.078796
C	4.790232	-2.396434	-0.913509
C	4.464447	2.566148	0.201160
C	-4.424556	2.627912	-0.796426
C	-5.092986	-2.320630	-0.005607
C	4.141939	-3.439494	0.015772
H	4.144780	-3.091891	1.050820
H	3.110157	-3.642827	-0.270290
H	4.694920	-4.380280	-0.026544
C	4.773902	-2.927258	-2.357895
H	5.227729	-2.206670	-3.040525
H	5.338049	-3.860019	-2.423219
H	3.758978	-3.122948	-2.702515
C	6.249789	-2.213077	-0.484825
H	6.773581	-3.167227	-0.560435
H	6.770986	-1.496581	-1.121362
H	6.330132	-1.872374	0.548579
C	5.656312	2.920230	-0.706087
H	6.143178	3.831662	-0.352738
H	6.401399	2.124864	-0.718655
H	5.324926	3.087414	-1.732345
C	3.503322	3.759608	0.220128
H	3.127285	3.992474	-0.776868
H	2.647636	3.580160	0.872993
H	4.026080	4.641828	0.592757
C	4.971549	2.336540	1.636755
H	5.455699	3.239012	2.015654
H	4.144434	2.090277	2.305460
H	5.695430	1.523167	1.682947
C	-4.366527	-3.641470	0.269055
H	-3.794655	-3.603846	1.197677
H	-5.098157	-4.445199	0.362769
H	-3.685508	-3.904588	-0.541161
C	-5.901579	-2.474572	-1.306417
H	-6.596932	-3.312096	-1.220978
H	-6.480773	-1.577885	-1.526779
H	-5.240083	-2.665200	-2.153018
C	-6.058027	-2.048860	1.162555
H	-6.752337	-2.883470	1.278526
H	-5.510747	-1.930175	2.099642
H	-6.645892	-1.145708	0.999084
C	-5.199568	2.583721	-2.125715
H	-4.533715	2.336549	-2.954076
H	-5.995733	1.839738	-2.100162
H	-5.652926	3.555791	-2.330247
C	-3.372419	3.738230	-0.887641
H	-2.799559	3.829754	0.036622
H	-2.673259	3.567827	-1.707181
H	-3.867723	4.693436	-1.067258
C	-5.395923	2.976439	0.345611
H	-5.845361	3.955987	0.170975
H	-6.202854	2.248178	0.425254
H	-4.873948	3.008538	1.303838

G-gauche-0-tBu
Number of imaginary frequencies: 0
Electronic Energy (SCF) = -1801.12453539 a.u
ZPV corrected Energy = -1800.345150 a.u.
Enthalpy = -1800.302659 a.u.
Free Energy = -1800.418328 a.u.

S	-1.192102	-3.064149	-1.740507
C	-0.921649	-4.477792	-0.538067
H	0.112423	-4.418671	-0.210632
H	-1.060025	-5.381593	-1.127737
C	-2.566387	-2.360486	-0.715136
H	-3.260146	-1.900153	-1.410719
H	-2.149606	-1.599579	-0.063159
C	-3.134492	-3.543000	0.046036
H	-3.734169	-4.164226	-0.622958
H	-3.785829	-3.206061	0.852651
C	-1.942999	-4.347489	0.584606
H	-1.499165	-3.816643	1.425716
H	-2.257655	-5.328149	0.940803
C	0.137020	-2.065922	-1.894274
C	-1.919301	0.203276	-3.184535
O	-2.920019	-0.423782	-3.481971
H	0.462112	-2.012496	-2.922568
C	0.994548	-1.550100	-0.862328
C	2.242127	-0.999395	-1.230333
C	0.680249	-1.575047	0.510297
C	3.150154	-0.532765	-0.289400
H	2.489838	-0.979896	-2.281565
C	1.560138	-1.092188	1.474464
H	-0.268202	-1.976762	0.820244
C	2.792450	-0.581301	1.062737
H	3.486547	-0.218297	1.805421
H	-1.044025	0.207989	-3.857186
C	-1.763937	1.026836	-1.977825
C	-0.596690	1.772445	-1.827219
C	-2.762146	1.066755	-1.003102
C	-0.413673	2.584201	-0.710932
H	0.157962	1.704936	-2.596798
C	-2.595397	1.833203	0.141658
H	-3.651361	0.478316	-1.160526
C	-1.419939	2.585076	0.255600
H	-1.284681	3.196080	1.136457
C	-3.633752	1.877302	1.266121
C	0.822643	3.466119	-0.519253
C	4.549496	-0.036331	-0.680643
C	1.208019	-1.089283	2.968304
C	4.718469	0.109690	-2.197187
H	4.612425	-0.844932	-2.713378
H	3.992239	0.807011	-2.617435
H	5.716025	0.493766	-2.416710
C	4.843105	1.330425	-0.039083
H	4.773399	1.295688	1.047389
H	5.854576	1.651417	-0.296995
H	4.147172	2.087153	-0.397203
C	5.585830	-1.057219	-0.173909
H	6.595673	-0.741885	-0.446446
H	5.544086	-1.158370	0.910925
H	5.406813	-2.041335	-0.610711
C	-0.136281	-1.765743	3.257850
H	-0.960333	-1.266488	2.747845
H	-0.131047	-2.814018	2.957415
H	-0.339591	-1.727951	4.329260
C	1.127191	0.366265	3.462612
H	0.886175	0.391508	4.527805
H	2.071137	0.890713	3.316652
H	0.351960	0.912635	2.925023
C	2.294722	-1.834782	3.762936
H	3.270774	-1.362812	3.655036
H	2.044193	-1.845082	4.825926
H	2.381968	-2.868976	3.424585
C	-4.143330	3.319305	1.436783

H	-4.882762	3.364772	2.238714
H	-3.333736	4.004479	1.688688
H	-4.614239	3.675774	0.519055
C	-4.833568	0.968922	0.978239
H	-5.361316	1.268479	0.071852
H	-4.533045	-0.074189	0.868627
H	-5.539447	1.026127	1.807891
C	-2.979036	1.409732	2.578361
H	-2.602086	0.390593	2.483042
H	-2.145383	2.050221	2.865064
H	-3.709626	1.427872	3.389385
C	0.383725	4.940168	-0.456351
H	-0.299050	5.120235	0.374304
H	1.254163	5.585451	-0.321687
H	-0.120332	5.236750	-1.377960
C	1.822006	3.307911	-1.669213
H	2.174148	2.280629	-1.757006
H	1.386898	3.604672	-2.624802
H	2.688925	3.944399	-1.488446
C	1.522397	3.087683	0.796852
H	0.869729	3.243698	1.655291
H	1.826092	2.042264	0.788205
H	2.412914	3.702748	0.939233

G-gauche-TS0-tBu

Number of imaginary frequencies: 1
Electronic Energy (SCF) = -1801.10970926 a.u
ZPV corrected Energy = -1800.329263 a.u.
Enthalpy = -1800.288433 a.u.
Free Energy = -1800.399302 a.u.

S	0.057826	3.547237	-1.612436
C	-1.022698	4.369768	-0.365420
H	-1.936795	3.789885	-0.274428
H	-1.256319	5.346739	-0.783858
C	1.552280	3.588912	-0.546944
H	2.384430	3.838777	-1.193781
H	1.710363	2.594027	-0.150927
C	1.251120	4.627193	0.529183
H	1.428629	5.629399	0.135082
H	1.920334	4.481549	1.377006
C	-0.216632	4.474752	0.926372
H	-0.354723	3.571452	1.516822
H	-0.564257	5.321412	1.517644
C	-0.511159	1.871156	-1.817514
C	1.064978	1.069751	-2.722462
O	1.937125	1.973484	-2.956386
H	-1.185442	1.958304	-2.666385
C	-1.159974	1.175246	-0.693283
C	-2.402236	0.565561	-0.913891
C	-0.561081	1.022357	0.561671
C	-3.040831	-0.176911	0.076667
H	-2.859883	0.686195	-1.884582
C	-1.178563	0.307677	1.581898
H	0.407346	1.452591	0.735995
C	-2.413068	-0.288355	1.318151
H	-2.897998	-0.853310	2.100660
H	0.446426	0.713576	-3.568110
C	1.403618	-0.091385	-1.822437
C	0.632141	-1.249807	-1.866417
C	2.493518	-0.026600	-0.962197
C	0.927514	-2.345013	-1.060252
H	-0.207973	-1.277812	-2.545108
C	2.824893	-1.101566	-0.136104
H	3.083590	0.876427	-0.975196
C	2.026371	-2.244629	-0.201875
H	2.273167	-3.090451	0.422566
C	4.065565	-1.083992	0.766362
C	0.099164	-3.634324	-1.082530
C	-4.407345	-0.838300	-0.139890
C	-0.536859	0.145491	2.964296
C	-4.926288	-0.654378	-1.569939
H	-5.071236	0.398108	-1.817524
H	-4.247015	-1.087286	-2.305580
H	-5.890579	-1.154103	-1.673382
C	-4.305678	-2.347825	0.142082
H	-3.980265	-2.547774	1.162573
H	-5.280166	-2.820544	0.003073
H	-3.597703	-2.823769	-0.536373
C	-5.427476	-0.212465	0.828677
H	-6.410464	-0.667152	0.688710
H	-5.133776	-0.359737	1.868021
H	-5.521325	0.860888	0.653562
C	0.766219	0.941413	3.095180
H	1.510589	0.620748	2.367993
H	0.599083	2.011465	2.964154
H	1.187859	0.790174	4.089858
C	-0.224118	-1.341904	3.204166
H	0.237014	-1.478076	4.184814
H	-1.128551	-1.949225	3.169708
H	0.462895	-1.718047	2.447232
C	-1.510741	0.642135	4.047382
H	-2.440475	0.073609	4.047220
H	-1.057389	0.540168	5.035535
H	-1.758679	1.694269	3.894718
C	5.152521	-1.956058	0.110175
H	6.058518	-1.961974	0.720661
H	4.812749	-2.986254	-0.003630
H	5.410460	-1.575362	-0.879491
C	4.622762	0.332796	0.952628
H	4.969598	0.763382	0.013439
H	3.875120	1.003491	1.379532
H	5.473909	0.303119	1.634812
C	3.746467	-1.651945	2.159538
H	2.977326	-1.062696	2.657797
H	3.403507	-2.684771	2.114857
H	4.644021	-1.630799	2.780775
C	1.009577	-4.830657	-1.411913
H	1.799467	-4.953367	-0.671110
H	0.425747	-5.753381	-1.434069
H	1.480514	-4.703169	-2.388333
C	-1.019965	-3.585819	-2.128222
H	-1.717718	-2.771482	-1.936133
H	-0.623264	-3.464167	-3.137335
H	-1.583806	-4.519599	-2.101956
C	-0.539225	-3.848212	0.301467
H	0.218674	-3.951335	1.077786
H	-1.178671	-3.006812	0.565685
H	-1.147684	-4.755330	0.301428

G-gauche-tBu

Number of imaginary frequencies: 0
Electronic Energy (SCF) = -1801.12347038 a.u
ZPV corrected Energy = -1800.340793 a.u.
Enthalpy = -1800.299976 a.u.
Free Energy = -1800.411388 a.u.

S	2.333390	-2.676874	-1.775903
C	4.011809	-1.887908	-1.749647
H	3.940004	-0.885120	-2.161340
H	4.630043	-2.494338	-2.410068
C	2.530388	-3.373379	-0.098992
H	2.136480	-4.382974	-0.147562
H	1.906278	-2.784585	0.562868
C	4.008762	-3.270448	0.257129
H	4.562965	-4.099712	-0.186031

H	4.130520	-3.326412	1.338862
C	4.509212	-1.943097	-0.310405
H	4.090626	-1.113399	0.258927
H	5.595780	-1.865598	-0.272392
C	1.245118	-1.169225	-1.741555
C	-0.121219	-1.913150	-1.962345
O	-0.014604	-3.127104	-1.364649
H	1.580825	-0.619326	-2.621615
C	1.405076	-0.302969	-0.535930
C	2.116014	0.890594	-0.648555
C	0.798197	-0.628020	0.677064
C	2.233512	1.766558	0.428955
H	2.559315	1.135153	-1.601983
C	0.885916	0.225535	1.771746
H	0.220797	-1.534710	0.729386
C	1.611287	1.409903	1.625437
H	1.677665	2.085149	2.466504
H	-0.213909	-1.983255	-3.071743
C	-1.297709	-1.048874	-1.509147
C	-1.389477	0.290615	-1.883006
C	-2.266567	-1.592638	-0.677495
C	-2.405628	1.107739	-1.396410
H	-0.627121	0.703954	-2.526752
C	-3.324747	-0.817255	-0.198220
H	-2.146815	-2.627897	-0.400933
C	-3.365241	0.528699	-0.560642
H	-4.160229	1.150010	-0.173668
C	-4.420950	-1.389381	0.709024
C	-2.465568	2.610637	-1.690351
C	2.994866	3.092742	0.335476
C	0.180475	-0.074939	3.097062
C	3.617346	3.310872	-1.047602
H	4.332908	2.526101	-1.297963
H	2.859801	3.346238	-1.831780
H	4.150971	4.262240	-1.059496
C	2.027427	4.256490	0.617637
H	1.581124	4.176615	1.608698
H	2.558210	5.209156	0.563303
H	1.219004	4.275495	-0.114676
C	4.124623	3.106353	1.380666
H	4.676886	4.046658	1.326708
H	3.736047	3.003433	2.393734
H	4.826571	2.289250	1.204145
C	-0.529288	-1.432869	3.077578
H	-1.289176	-1.480567	2.298864
H	0.172502	-2.252959	2.917374
H	-1.022727	-1.599641	4.036130
C	-0.870478	1.020082	3.357265
H	-1.403911	0.819313	4.288645
H	-0.408394	2.004116	3.439013
H	-1.599127	1.056365	2.546962
C	1.206353	-0.081184	4.243531
H	1.713100	0.879105	4.339115
H	0.708693	-0.292217	5.192207
H	1.965465	-0.848784	4.081244
C	-5.781108	-1.263930	-0.001049
H	-6.576176	-1.672699	0.626570
H	-6.024384	-0.223391	-0.217065
H	-5.777525	-1.811748	-0.945120
C	-4.185938	-2.866606	1.042265
H	-4.177951	-3.487698	0.145839
H	-3.241454	-3.016839	1.566563
H	-4.987759	-3.225672	1.689704
C	-4.469079	-0.602867	2.031246
H	-3.523351	-0.685920	2.567159
H	-4.669529	0.455385	1.864981
H	-5.259304	-0.996322	2.674419
C	-3.833909	2.980184	-2.288248
H	-4.651107	2.736936	-1.609587
H	-3.878888	4.051826	-2.493764
H	-4.004767	2.447369	-3.225470
C	-1.377559	3.056995	-2.673423
H	-0.377075	2.873575	-2.280463
H	-1.466445	2.546096	-3.633493
H	-1.469898	4.128749	-2.856341
C	-2.262682	3.377075	-0.369511
H	-3.043836	3.139170	0.352754
H	-1.301792	3.122459	0.080230
H	-2.282733	4.454674	-0.547253

G-gauche'-0-tBu

Number of imaginary frequencies: 0

Electronic Energy (SCF) = -1801.12453346 a.u

ZPV corrected Energy = -1800.345081 a.u.

Enthalpy = -1800.302611 a.u.

Free Energy = -1800.418407 a.u.

S	-1.206869	-3.053991	-1.741027
C	-0.947739	-4.461980	-0.529097
H	0.086692	-4.408559	-0.201765
H	-1.092980	-5.368598	-1.112811
C	-2.580656	-2.337548	-0.725338
H	-3.271305	-1.880224	-1.426021
H	-2.161982	-1.572588	-0.080113
C	-3.155369	-3.510217	0.045832
H	-3.758319	-4.134100	-0.617743
H	-3.804838	-3.162496	0.849329
C	-1.968592	-4.316293	0.591978
H	-1.522516	-3.780226	1.428515
H	-2.288583	-5.292078	0.956765
C	0.126777	-2.060847	-1.893690
C	-1.930072	0.224417	-3.165113
O	-2.941442	-0.387925	-3.456298
H	0.460308	-2.020036	-2.919937
C	0.985531	-1.549293	-0.860202
C	2.242794	-1.019340	-1.225668
C	0.663219	-1.558549	0.510834
C	3.152734	-0.558991	-0.283593
H	2.496178	-1.011353	-2.275706
C	1.545096	-1.080655	1.475805
H	-0.292689	-1.943853	0.818974
C	2.787272	-0.592047	1.066860
H	3.483051	-0.234303	1.810556
H	-1.059023	0.216507	-3.843069
C	-1.755427	1.046241	-1.958858
C	-0.575684	1.781004	-1.818076
C	-2.740182	1.101165	-0.978436
C	-0.375518	2.588783	-0.706989
H	0.170624	1.704553	-2.594892
C	-2.556565	1.869358	0.168741
H	-3.642216	0.525065	-1.121293
C	-1.375308	2.603832	0.274074
H	-1.218371	3.214319	1.149129
C	1.187278	-1.062385	2.968214
C	4.561043	-0.085500	-0.670919
C	-3.635346	1.876464	1.256142
C	0.869637	3.460161	-0.526615
C	4.868976	1.283170	-0.040269
H	4.791616	1.259644	1.045997
H	5.886292	1.588228	-0.294488
H	4.185427	2.045577	-0.410088
C	4.740998	0.044117	-2.187665
H	4.624511	-0.913660	-2.695725
H	4.027147	0.747681	-2.618579
H	5.745134	0.412132	-2.404698
C	5.580383	-1.115530	-0.148478
H	6.595971	-0.816706	-0.418218

H	5.531273	-1.205600	0.937025
H	5.390275	-2.101265	-0.576858
C	1.146442	0.395733	3.459434
H	0.905355	0.429548	4.524333
H	2.104053	0.894658	3.313427
H	0.387043	0.962207	2.919646
C	-0.177048	-1.698104	3.255208
H	-0.984520	-1.176735	2.740350
H	-0.202127	-2.747048	2.958327
H	-0.382682	-1.651062	4.325819
C	2.249816	-1.836948	3.767932
H	3.239353	-1.393732	3.660567
H	1.996735	-1.837009	4.830381
H	2.307659	-2.874149	3.432529
C	1.861707	3.286173	-1.680575
H	2.202299	2.254722	-1.765382
H	1.425165	3.583150	-2.635428
H	2.736178	3.914282	-1.506970
C	0.443865	4.938538	-0.470615
H	-0.232830	5.129498	0.362604
H	1.320644	5.576927	-0.344406
H	-0.062351	5.233871	-1.391448
C	1.573493	3.084858	0.788184
H	0.927783	3.253074	1.649531
H	1.867941	2.036836	0.785312
H	2.470246	3.693053	0.920609
C	-3.258420	2.760508	2.449180
H	-4.056759	2.725828	3.191661
H	-2.341416	2.419983	2.932009
H	-3.123866	3.802582	2.155752
C	-4.954619	2.399532	0.660797
H	-4.835850	3.418913	0.289454
H	-5.297129	1.777299	-0.166008
H	-5.735663	2.404177	1.423634
C	-3.843811	0.438958	1.766594
H	-4.167777	-0.227864	0.968003
H	-2.920304	0.039275	2.188114
H	-4.607663	0.424191	2.546425

G-gauche'-TS0-tBu
Number of imaginary frequencies: 1
Electronic Energy (SCF) = -1801.10969506 a.u
ZPV corrected Energy = -1800.329265 a.u.
Enthalpy = -1800.288403 a.u.
Free Energy = -1800.399277 a.u.

S	0.169216	3.542860	-1.600516
C	-0.881877	4.390994	-0.345782
H	-1.815868	3.842757	-0.260265
H	-1.080546	5.379572	-0.755121
C	1.666975	3.531535	-0.538605
H	2.504074	3.764242	-1.185562
H	1.798398	2.529052	-0.152342
C	1.398984	4.567206	0.548971
H	1.607325	5.567640	0.165666
H	2.064122	4.391575	1.394313
C	-0.072382	4.456162	0.946426
H	-0.238051	3.551163	1.527298
H	-0.392881	5.306812	1.547250
C	-0.454068	1.886880	-1.814105
C	1.091075	1.042909	-2.732086
O	1.989906	1.920273	-2.966083
H	-1.129583	2.002145	-2.658694
C	-1.120226	1.205451	-0.690595
C	-2.385647	0.643455	-0.905840
C	-0.518380	1.021079	0.558606
C	-3.043757	-0.082271	0.084547
H	-2.845990	0.787878	-1.872006
C	-1.153567	0.321923	1.578598
H	0.465960	1.415148	0.728448
C	-2.411566	-0.225582	1.320534
H	-2.912319	-0.776894	2.102919
H	0.458270	0.709491	-3.576468
C	1.395937	-0.130319	-1.836238
C	0.586656	-1.263133	-1.878520
C	2.485723	-0.099303	-0.975366
C	0.843962	-2.363975	-1.068208
H	-0.252890	-1.265191	-2.558437
C	2.776704	-1.179199	-0.139534
H	3.105700	0.783464	-0.991009
C	1.940801	-2.294914	-0.202752
H	2.152675	-3.144346	0.428807
C	-0.509274	0.131204	2.956277
C	-4.434379	-0.693264	-0.126825
C	4.017503	-1.187853	0.763070
C	-0.023077	-3.627578	-1.092939
C	-4.383245	-2.207869	0.141656
H	-4.058050	-2.427762	1.158117
H	-5.374475	-2.645350	0.005619
H	-3.696805	-2.702261	-0.545744
C	-4.957083	-0.479135	-1.551309
H	-5.065513	0.579870	-1.789563
H	-4.299556	-0.930402	-2.295627
H	-5.939596	-0.942787	-1.651081
C	-5.425086	-0.040762	0.854634
H	-6.424319	-0.459653	0.718065
H	-5.129532	-0.207319	1.890538
H	-5.482645	1.036692	0.689355
C	-0.274161	-1.368819	3.205248
H	0.182496	-1.521361	4.185565
H	-1.207972	-1.930132	3.178223
H	0.390739	-1.784894	2.449494
C	0.837037	0.854179	3.070861
H	1.554591	0.488045	2.338223
H	0.729422	1.931374	2.935566
H	1.259266	0.684579	4.062342
C	-1.446607	0.686567	4.043010
H	-2.404674	0.167289	4.053711
H	-0.991476	0.568285	5.028549
H	-1.640655	1.749026	3.884114
C	-1.138552	-3.544088	-2.140474
H	-1.811858	-2.709364	-1.948280
H	-0.736921	-3.433150	-3.148849
H	-1.730171	-4.460590	-2.116347
C	0.852243	-4.849701	-1.423030
H	1.636611	-4.996925	-0.680805
H	0.241575	-5.754816	-1.448274
H	1.328806	-4.734145	-2.398202
C	-0.670610	-3.825587	0.288947
H	0.081603	-3.952169	1.067264
H	-1.286729	-2.966972	0.553051
H	-1.304735	-4.714944	0.285351
C	3.728887	-1.868105	2.111380
H	4.620514	-1.829434	2.739910
H	2.919677	-1.366740	2.642109
H	3.458707	-2.916932	1.995573
C	5.130949	-1.969721	0.040866
H	4.822932	-2.998179	-0.152939
H	5.373767	-1.504729	-0.915978
H	6.038415	-1.994251	0.648865
C	4.524483	0.231755	1.052952
H	4.863008	0.739505	0.150405
H	3.750321	0.843656	1.518829
H	5.371893	0.183753	1.738744

G-gauche'-tBu

Number of imaginary frequencies: 0
Electronic Energy (SCF) = -1801.12041041 a.u
ZPV corrected Energy = -1800.337388 a.u.
Enthalpy = -1800.296667 a.u.
Free Energy = -1800.407455 a.u.

S	-0.585245	3.374242	-1.461749
C	-2.087489	3.678393	-0.404296
H	-2.832912	2.911394	-0.596522
H	-2.476190	4.637487	-0.743603
C	0.564740	3.648773	-0.067311
H	1.384971	4.229386	-0.475967
H	0.948793	2.683681	0.239339
C	-0.223297	4.349483	1.033195
H	-0.280010	5.419128	0.823348
H	0.285557	4.225452	1.989455
C	-1.627130	3.744333	1.047661
H	-1.596333	2.742026	1.469970
H	-2.316593	4.338807	1.647582
C	-0.772908	1.554216	-1.834246
C	0.665846	1.291649	-2.446831
O	1.239818	2.513234	-2.601924
H	-1.477040	1.621011	-2.664453
C	-1.392336	0.694466	-0.779188
C	-2.620025	0.090672	-1.055915
C	-0.793693	0.468866	0.460380
C	-3.256637	-0.725231	-0.122530
H	-3.070092	0.268869	-2.021145
C	-1.417270	-0.304503	1.435232
H	0.169666	0.899628	0.661936
C	-2.642990	-0.892172	1.119079
H	-3.138989	-1.496582	1.863944
H	0.452005	0.784403	-3.410234
C	1.478360	0.278971	-1.643153
C	1.143440	-1.076184	-1.683653
C	2.560624	0.685565	-0.885686
C	1.858642	-2.019122	-0.958750
H	0.304368	-1.377493	-2.294175
C	3.306032	-0.229364	-0.131233
H	2.816726	1.734531	-0.906717
C	2.934229	-1.568793	-0.177033
H	3.494086	-2.293127	0.393536
C	-0.832668	-0.454742	2.844562
C	-4.591421	-1.421974	-0.405583
C	4.501291	0.270366	0.687699
C	1.521513	-3.513896	-0.989636
C	-4.407517	-2.943971	-0.264342
H	-4.080027	-3.218025	0.738434
H	-5.350686	-3.457227	-0.462083
H	-3.664613	-3.312509	-0.973709
C	-5.114314	-1.130433	-1.816002
H	-5.298017	-0.065895	-1.968799
H	-4.418148	-1.470829	-2.583809
H	-6.058842	-1.654530	-1.968138
C	-5.644382	-0.941617	0.609181
H	-6.600560	-1.434334	0.421945
H	-5.347054	-1.165523	1.633580
H	-5.796431	0.136364	0.529910
C	-0.883717	-1.917766	3.312793
H	-0.472960	-1.996680	4.321030
H	-1.901135	-2.306106	3.341709
H	-0.294104	-2.558136	2.657481
C	0.624823	0.014229	2.914147
H	1.254017	-0.533242	2.212778
H	0.722478	1.078175	2.698420
H	1.014112	-0.152303	3.919519
C	-1.674860	0.407707	3.803764
H	-2.716010	0.082906	3.815138
H	-1.284932	0.335049	4.821297
H	-1.652593	1.457043	3.504947
C	0.335592	-3.823930	-1.909663
H	-0.570313	-3.309814	-1.585814
H	0.538925	-3.539358	-2.942993
H	0.133494	-4.896149	-1.893521
C	2.742941	-4.304547	-1.491938
H	3.604965	-4.169733	-0.838631
H	2.513547	-5.371688	-1.528590
H	3.027521	-3.983049	-2.495440
C	1.158120	-3.987461	0.427818
H	1.965863	-3.802288	1.135807
H	0.268882	-3.468486	0.785352
H	0.948745	-5.059398	0.427618
C	5.152511	-0.841941	1.516243
H	5.983498	-0.429419	2.090699
H	4.447630	-1.284928	2.221635
H	5.549824	-1.638386	0.885721
C	5.558940	0.844249	-0.272768
H	5.905558	0.078837	-0.969399
H	5.158243	1.672841	-0.856707
H	6.421913	1.210824	0.287585
C	4.044861	1.379929	1.652153
H	3.598611	2.218897	1.118748
H	3.309111	0.999937	2.361662
H	4.897722	1.759502	2.218801

G-anti-0-tBu
Number of imaginary frequencies: 0
Electronic Energy (SCF) = -1801.11829632 a.u
ZPV corrected Energy = -1800.339638 a.u.
Enthalpy = -1800.296804 a.u.
Free Energy = -1800.416495 a.u.

S	0.512984	-1.278000	0.789033
C	-0.075443	-2.499978	2.085312
H	-0.882990	-2.020019	2.630492
H	0.774869	-2.631837	2.751648
C	0.247970	-2.502830	-0.579606
H	1.023739	-2.313599	-1.314867
H	-0.725451	-2.295543	-1.017344
C	0.323949	-3.872281	0.068051
H	1.364574	-4.130248	0.273184
H	-0.080785	-4.638476	-0.593535
C	-0.465763	-3.794264	1.382913
H	-1.533513	-3.794635	1.164634
H	-0.261935	-4.656784	2.017063
C	-0.448290	0.072178	0.637831
C	1.134529	1.053692	-2.437116
H	0.949438	0.187511	-3.099531
O	0.424865	2.037380	-2.506663
C	2.275817	0.891966	-1.523807
C	2.548382	1.847932	-0.546648
C	3.062272	-0.252063	-1.628038
C	3.595046	1.664078	0.346944
H	1.911761	2.718849	-0.489976
C	4.132963	-0.461944	-0.761503
H	2.820155	-0.980263	-2.390653
C	4.372489	0.506030	0.215174
H	5.186463	0.352677	0.902519
H	0.140271	0.974856	0.656455
C	-1.861280	0.150384	0.391873
C	-2.436277	1.409223	0.118103
C	-2.728517	-0.956844	0.403661
C	-3.791924	1.566553	-0.136252
H	-1.782215	2.270949	0.101406
C	-4.092198	-0.833464	0.151989
H	-2.329215	-1.933855	0.620052
C	-4.615248	0.432846	-0.118539

H	-5.667213	0.540115	-0.315512
C	4.976657	-1.734355	-0.884344
C	3.847783	2.704652	1.442216
C	-4.341926	2.968033	-0.437463
C	-4.969844	-2.092112	0.188498
C	6.124112	-1.775440	0.129915
H	5.758361	-1.762670	1.157572
H	6.694655	-2.694955	-0.006953
H	6.809400	-0.936505	0.000532
C	5.577239	-1.816218	-2.298881
H	4.802516	-1.842492	-3.065095
H	6.220292	-0.957326	-2.498337
H	6.178336	-2.721952	-2.399347
C	4.074940	-2.958790	-0.643958
H	4.658239	-3.877932	-0.726519
H	3.634420	-2.924375	0.353298
H	3.265178	-3.009634	-1.370971
C	5.026446	2.330621	2.346847
H	4.857021	1.384848	2.863783
H	5.959521	2.252411	1.786886
H	5.159046	3.103416	3.105110
C	4.148872	4.065991	0.790439
H	5.042266	4.008758	0.166038
H	3.321800	4.403557	0.166014
H	4.319320	4.821503	1.559884
C	2.585621	2.827079	2.315884
H	2.750020	3.554559	3.113138
H	1.724817	3.156232	1.734388
H	2.334921	1.868942	2.774264
C	-4.453912	-3.109064	-0.845741
H	-3.425686	-3.405736	-0.639897
H	-5.071683	-4.009686	-0.832886
H	-4.486884	-2.688705	-1.852498
C	-4.901584	-2.721923	1.591874
H	-3.882370	-3.002362	1.857294
H	-5.264556	-2.023162	2.347739
H	-5.520161	-3.621145	1.635012
C	-6.439554	-1.795079	-0.129084
H	-6.878174	-1.104003	0.591901
H	-6.560107	-1.369160	-1.126104
H	-7.014246	-2.722024	-0.091981
C	-5.854061	2.964924	-0.689051
H	-6.120988	2.348380	-1.548480
H	-6.408747	2.600937	0.176956
H	-6.189750	3.982747	-0.894416
C	-4.058189	3.898915	0.755218
H	-2.989661	3.986305	0.950739
H	-4.446345	4.900115	0.555032
H	-4.536823	3.524303	1.662011
C	-3.649651	3.530372	-1.692282
H	-4.036450	4.525235	-1.924423
H	-2.571995	3.611723	-1.554418
H	-3.828681	2.886190	-2.555102

G-anti-TS0-tBu
Number of imaginary frequencies: 1
Electronic Energy (SCF) = -1801.10324813 a.u
ZPV corrected Energy = -1800.323672 a.u.
Enthalpy = -1800.282407 a.u.
Free Energy = -1800.397701 a.u.

S	0.334141	-0.395937	1.509207
C	-0.865199	-0.247340	2.910881
H	-1.404976	0.686189	2.778237
H	-0.256769	-0.189801	3.812525
C	0.018255	-2.193349	1.290210
H	0.982693	-2.682553	1.203906
H	-0.525157	-2.304027	0.358777
C	-0.798749	-2.648604	2.496052
H	-0.134098	-2.879492	3.330000
H	-1.349109	-3.555100	2.245680
C	-1.729938	-1.497791	2.875694
H	-2.513683	-1.384105	2.127788
H	-2.203398	-1.661991	3.843565
C	-0.305292	0.399239	0.065628
C	0.682242	-0.102286	-1.615388
H	0.378051	-1.167205	-1.615061
O	0.247504	0.656203	-2.526181
C	2.099567	0.041812	-1.115090
C	2.695778	1.296583	-1.043805
C	2.817282	-1.077876	-0.709422
C	3.986134	1.454595	-0.544455
H	2.124226	2.148759	-1.384412
C	4.116816	-0.965759	-0.211924
H	2.346607	-2.049833	-0.789068
C	4.677292	0.310357	-0.131775
H	5.676381	0.417026	0.253918
H	0.057188	1.420895	0.155071
C	-1.776572	0.326568	-0.127476
C	-2.590807	1.380828	0.291893
C	-2.379693	-0.777879	-0.730766
C	-3.977334	1.337982	0.152636
H	-2.119647	2.243572	0.743247
C	-3.761462	-0.864552	-0.881214
H	-1.754127	-1.571527	-1.108909
C	-4.542640	0.200310	-0.427018
H	-5.611759	0.147226	-0.539068
C	4.881835	-2.225185	0.210219
C	4.596985	2.857983	-0.457417
C	-4.823181	2.517879	0.644780
C	-4.367460	-2.106715	-1.543936
C	6.251937	-1.904686	0.817407
H	6.163610	-1.278735	1.706778
H	6.744957	-2.832685	1.110836
H	6.902781	-1.395568	0.105607
C	5.096158	-3.121036	-1.023312
H	4.147027	-3.415123	-1.471696
H	5.680146	-2.599950	-1.783886
H	5.634119	-4.029614	-0.743974
C	4.063676	-3.001067	1.257659
H	4.614542	-3.883931	1.588133
H	3.859055	-2.380133	2.131779
H	3.111105	-3.338632	0.850930
C	6.015435	2.849216	0.122745
H	6.036839	2.447619	1.136992
H	6.700903	2.262903	-0.490867
H	6.397267	3.870556	0.162520
C	4.656151	3.476087	-1.865919
H	5.278747	2.872016	-2.528351
H	3.665177	3.551713	-2.312857
H	5.082721	4.480388	-1.819970
C	3.713829	3.739749	0.444360
H	4.132564	4.745730	0.517091
H	2.700803	3.825060	0.051705
H	3.651522	3.323980	1.451891
C	-3.833095	-2.224319	-2.982768
H	-2.746699	-2.308377	-3.002406
H	-4.249315	-3.109025	-3.469231
H	-4.111231	-1.348735	-3.571583
C	-3.959925	-3.359898	-0.747639
H	-2.877509	-3.484261	-0.719772
H	-4.320170	-3.300999	0.281179
H	-4.387164	-4.254046	-1.206036
C	-5.897829	-2.053353	-1.601207
H	-6.339005	-1.990734	-0.605207
H	-6.251807	-1.202950	-2.185338
H	-6.274962	-2.960792	-2.075130

C	-6.322092	2.303085	0.409508
H	-6.552544	2.183923	-0.650025
H	-6.694943	1.426047	0.940678
H	-6.874121	3.170679	0.773858
C	-4.596238	2.711053	2.155308
H	-3.551765	2.927710	2.379208
H	-5.197707	3.544435	2.524107
H	-4.882163	1.814679	2.708891
C	-4.396757	3.796625	-0.098302
H	-4.990451	4.646936	0.243618
H	-3.346127	4.029999	0.073452
H	-4.545569	3.687699	-1.173862

G-anti-tBu
Number of imaginary frequencies: 0
Electronic Energy (SCF) = -1801.11184698 a.u
ZPV corrected Energy = -1800.330190 a.u.
Enthalpy = -1800.288984 a.u.
Free Energy = -1800.402439 a.u.

S	0.337074	-0.028963	1.458449
C	-0.927298	0.476326	2.691536
H	-1.410274	1.381406	2.336363
H	-0.365044	0.702201	3.596158
C	-0.095174	-1.808562	1.551724
H	0.834218	-2.366484	1.602676
H	-0.619808	-2.057821	0.635612
C	-0.985725	-1.964141	2.782863
H	-0.368911	-2.054815	3.677878
H	-1.578878	-2.873202	2.689476
C	-1.859512	-0.714807	2.872000
H	-2.609508	-0.719822	2.082884
H	-2.372746	-0.644621	3.830499
C	-0.277177	0.539361	-0.245175
C	0.595110	-0.090222	-1.404645
H	0.354533	-1.179362	-1.406194
O	0.268608	0.559541	-2.521684
C	2.070659	-0.011971	-0.991120
C	2.728795	1.213759	-1.001567
C	2.757585	-1.141983	-0.565187
C	4.045959	1.334079	-0.565656
H	2.185799	2.075227	-1.365779
C	4.082544	-1.070931	-0.126553
H	2.241499	-2.094199	-0.576586
C	4.703733	0.177942	-0.129175
H	5.723108	0.254331	0.207999
H	-0.009971	1.594289	-0.193404
C	-1.757408	0.370662	-0.373829
C	-2.602061	1.408614	0.012305
C	-2.316585	-0.822647	-0.825576
C	-3.988429	1.264139	-0.004331
H	-2.159195	2.341637	0.333275
C	-3.695946	-1.006898	-0.859314
H	-1.664738	-1.611868	-1.167142
C	-4.512219	0.044020	-0.434138
H	-5.580328	-0.087453	-0.449464
C	4.796194	-2.345380	0.337004
C	4.722888	2.709688	-0.569068
C	-4.874925	2.427642	0.450928
C	-4.261187	-2.342794	-1.352841
C	6.226851	-2.077798	0.816375
H	6.248430	-1.390532	1.663518
H	6.684063	-3.015105	1.137239
H	6.848294	-1.660295	0.023105
C	4.857132	-3.346746	-0.830415
H	3.860347	-3.607840	-1.185745
H	5.414031	-2.928341	-1.670539
H	5.355167	-4.266341	-0.515604
C	4.009798	-2.975669	1.501133
H	4.511606	-3.880156	1.851228
H	3.935021	-2.281680	2.340483
H	2.999362	-3.250577	1.199053
C	6.161724	2.660978	-0.043386
H	6.205706	2.304143	0.986793
H	6.795100	2.016626	-0.654708
H	6.590250	3.664211	-0.065379
C	4.752835	3.259090	-2.006516
H	5.321444	2.597339	-2.662252
H	3.749022	3.358131	-2.418909
H	5.224391	4.244057	-2.025180
C	3.918532	3.673184	0.322954
H	4.385861	4.660224	0.331922
H	2.895576	3.789396	-0.034450
H	3.877949	3.307303	1.350789
C	-3.824949	-2.567836	-2.811707
H	-2.739593	-2.577455	-2.910201
H	-4.206055	-3.523931	-3.176371
H	-4.211932	-1.777811	-3.457328
C	-3.709891	-3.481753	-0.475469
H	-2.622478	-3.537216	-0.524540
H	-3.994834	-3.340091	0.568854
H	-4.108519	-4.441797	-0.809036
C	-5.791458	-2.390258	-1.293605
H	-6.162601	-2.260572	-0.275754
H	-6.245137	-1.623530	-1.922918
H	-6.137927	-3.360628	-1.651956
C	-6.366742	2.082286	0.398344
H	-6.690239	1.837071	-0.614042
H	-6.611714	1.240866	1.048369
H	-6.949353	2.941428	0.733611
C	-4.517405	2.800689	1.901325
H	-3.479791	3.122592	1.988953
H	-5.150973	3.619914	2.246830
H	-4.666610	1.950650	2.569763
C	-4.626337	3.642280	-0.460913
H	-5.246847	4.483593	-0.145576
H	-3.584843	3.961939	-0.428648
H	-4.873459	3.405987	-1.497146

H-gauche-0-tBu
Number of imaginary frequencies: 0
Electronic Energy (SCF) = -1801.12464941 a.u
ZPV corrected Energy = -1800.345574 a.u.
Enthalpy = -1800.302978 a.u.
Free Energy = -1800.419294 a.u.

S	1.151400	3.037652	-1.755155
C	0.831706	4.498526	-0.654903
H	0.004089	4.240432	0.002062
H	0.539193	5.319179	-1.304904
C	2.489502	2.388927	-0.620071
H	3.385238	2.351649	-1.234372
H	2.208684	1.379731	-0.343385
C	2.623566	3.347819	0.555553
H	3.656105	3.392083	0.901256
H	2.013545	3.001460	1.388853
C	2.127593	4.727246	0.099884
H	1.971789	5.395297	0.947180
H	2.865836	5.192692	-0.556862
C	-0.174037	2.035254	-1.903502
C	1.948494	-0.201867	-3.199518
O	2.929527	0.457792	-3.489687
H	-0.453312	1.907954	-2.938465
C	-1.029911	1.519142	-0.871201
C	-2.267767	0.944871	-1.235915
C	-0.716368	1.558276	0.501174

C	-3.169055	0.472093	-0.291421
H	-2.513445	0.910948	-2.287256
C	-1.592357	1.077029	1.469460
H	0.228064	1.972219	0.806867
C	-2.816059	0.543193	1.061103
H	-3.506576	0.178473	1.806357
H	1.076210	-0.231795	-3.875883
C	1.811750	-1.029112	-1.992773
C	2.812179	-1.043297	-1.019456
C	0.655938	-1.789805	-1.831891
C	2.656359	-1.794678	0.136726
H	3.691344	-0.442096	-1.185889
C	0.485492	-2.589002	-0.704413
H	-0.101510	-1.741283	-2.600196
C	1.491860	-2.561671	0.261700
H	1.364530	-3.159735	1.152579
C	-0.739961	-3.482579	-0.497914
C	3.690288	-1.798500	1.265885
C	-4.556639	-0.058538	-0.679600
C	-1.244244	1.102184	2.963891
C	-5.618167	0.934764	-0.170568
H	-5.466322	1.923136	-0.608065
H	-6.619889	0.592686	-0.440763
H	-5.576815	1.037363	0.914088
C	-4.725656	-0.208937	-2.195643
H	-4.641880	0.747690	-2.712282
H	-3.985282	-0.890164	-2.617589
H	-5.714930	-0.615209	-2.412676
C	-4.812704	-1.432528	-0.037159
H	-4.740785	-1.395266	1.049111
H	-5.815979	-1.780394	-0.292313
H	-4.097770	-2.170590	-0.396892
C	-2.349650	1.833580	3.745759
H	-3.314538	1.338248	3.642414
H	-2.101909	1.863921	4.809002
H	-2.459568	2.860836	3.393179
C	-1.131261	-0.344068	3.478791
H	-0.893156	-0.349282	4.544946
H	-2.062527	-0.892057	3.337324
H	-0.341838	-0.879869	2.951144
C	0.083737	1.813509	3.245659
H	0.920476	1.317756	2.753492
H	0.061143	2.853724	2.917916
H	0.280406	1.806440	4.318957
C	-1.444392	-3.089260	0.811421
H	-1.760229	-2.047714	0.784369
H	-2.327815	-3.711853	0.964603
H	-0.790139	-3.222671	1.672462
C	-1.740981	-3.356191	-1.650366
H	-1.301385	-3.661501	-2.601207
H	-2.598934	-4.001891	-1.459846
H	-2.107632	-2.335449	-1.753953
C	-0.283544	-4.949972	-0.409845
H	-1.146171	-5.603061	-0.263223
H	0.223210	-5.256453	-1.326732
H	0.402033	-5.107819	0.423008
C	4.222281	-3.227704	1.470695
H	4.956648	-3.243877	2.278372
H	3.422305	-3.920795	1.731612
H	4.705517	-3.595598	0.563934
C	4.875960	-0.876104	0.964220
H	5.412350	-1.184699	0.065968
H	4.558256	0.159446	0.832766
H	5.579223	-0.904883	1.797555
C	3.020379	-1.312492	2.563969
H	2.624750	-0.303112	2.442827
H	2.197649	-1.962003	2.861688
H	3.747148	-1.297657	3.378456

H-gauche-TS0-tBu
Number of imaginary frequencies: 1
Electronic Energy (SCF) = -1801.11045242 a.u
ZPV corrected Energy = -1800.329958 a.u.
Enthalpy = -1800.289157 a.u.
Free Energy = -1800.399990 a.u.

S	-0.175807	3.541990	-1.567922
C	-1.357284	4.317644	-0.404615
H	-1.833293	3.527927	0.172642
H	-2.103048	4.847688	-0.991266
C	1.252694	3.697602	-0.415839
H	1.970447	4.306311	-0.957583
H	1.682495	2.715939	-0.266688
C	0.734635	4.380020	0.849482
H	1.521425	4.991117	1.290180
H	0.444987	3.628756	1.581404
C	-0.489151	5.214775	0.468699
H	-1.048574	5.540922	1.345073
H	-0.188045	6.104875	-0.086914
C	-0.636368	1.837591	-1.781816
C	1.016716	1.184859	-2.675067
O	1.815087	2.165724	-2.862853
H	-1.304184	1.879485	-2.639271
C	-1.238929	1.085693	-0.669794
C	-2.421699	0.374087	-0.911831
C	-0.648611	0.983810	0.594927
C	-3.010319	-0.422358	0.066704
H	-2.873343	0.459673	-1.889129
C	-1.218881	0.212607	1.603661
H	0.278101	1.496895	0.779832
C	-2.393152	-0.485668	1.316898
H	-2.839682	-1.094820	2.088588
H	0.446411	0.806462	-3.543930
C	1.432870	0.030132	-1.801181
C	2.535982	0.139415	-0.961532
C	0.726173	-1.168641	-1.852889
C	2.942100	-0.930915	-0.163819
H	3.078656	1.071854	-0.971245
C	1.095682	-2.259505	-1.071750
H	-0.123607	-1.231560	-2.516843
C	2.204051	-2.113764	-0.232906
H	2.508510	-2.953891	0.374353
C	0.335720	-3.590009	-1.100001
C	4.186934	-0.865782	0.729919
C	-4.319639	-1.185260	-0.168494
C	-0.583334	0.079630	2.992837
C	-5.404460	-0.608141	0.759422
H	-5.567855	0.451340	0.553609
H	-6.350082	-1.133621	0.609969
H	-5.125301	-0.707946	1.808499
C	-4.810930	-1.070292	-1.615347
H	-5.026882	-0.037223	-1.890744
H	-4.080684	-1.466517	-2.322390
H	-5.732283	-1.642730	-1.732500
C	-4.125149	-2.677662	0.152390
H	-3.815600	-2.834190	1.185207
H	-5.062504	-3.216571	0.000029
H	-3.369982	-3.120904	-0.496355
C	-1.626204	0.390258	4.080998
H	-2.466200	-0.302804	4.051278
H	-1.168357	0.314345	5.069325
H	-2.019295	1.402251	3.966505
C	-0.077621	-1.362834	3.171563
H	0.389421	-1.481798	4.151688
H	-0.894261	-2.080841	3.096577
H	0.658745	-1.610296	2.408215
C	0.595563	1.038298	3.188393

H	1.386940	0.869743	2.459779
H	0.277184	2.078740	3.114352
H	1.023731	0.891712	4.181002
C	-0.265994	-3.856662	0.291251
H	-0.946062	-3.055361	0.577171
H	-0.823504	-4.795947	0.288811
H	0.509858	-3.927373	1.053343
C	-0.802679	-3.583704	-2.125733
H	-0.429921	-3.430153	-3.139638
H	-1.319768	-4.544211	-2.102502
H	-1.535331	-2.806460	-1.911017
C	1.300872	-4.732517	-1.462394
H	0.765017	-5.683797	-1.489032
H	1.748034	-4.566955	-2.444254
H	2.108470	-4.824763	-0.736369
C	5.255789	-1.814092	0.155448
H	6.160373	-1.783844	0.767028
H	4.898537	-2.844032	0.130807
H	5.523005	-1.526058	-0.862750
C	4.778797	0.546452	0.798214
H	5.112267	0.896367	-0.179086
H	4.058014	1.266416	1.189278
H	5.644349	0.546845	1.462702
C	3.844277	-1.308064	2.163038
H	3.097560	-0.650231	2.607284
H	3.456995	-2.325870	2.194265
H	4.739643	-1.274552	2.787254

H-gauche-tBu

Number of imaginary frequencies: 0
Electronic Energy (SCF) = -1801.12318628 a.u
ZPV corrected Energy = -1800.340580 a.u.
Enthalpy = -1800.299739 a.u.
Free Energy = -1800.411261 a.u.

S	2.391125	-2.637599	-1.739670
C	4.046025	-1.814636	-1.811235
H	3.912448	-0.751456	-1.628930
H	4.445836	-1.962983	-2.811290
C	2.652272	-3.239569	-0.029821
H	2.727539	-4.320068	-0.140907
H	1.758091	-3.012087	0.533745
C	3.938421	-2.612819	0.489823
H	4.369613	-3.238036	1.271496
H	3.723020	-1.634025	0.918744
C	4.875251	-2.462525	-0.707821
H	5.746343	-1.850126	-0.476108
H	5.232320	-3.442696	-1.029668
C	1.258996	-1.168576	-1.717534
C	-0.094360	-1.945610	-1.925086
O	0.018184	-3.140169	-1.295341
H	1.574650	-0.611465	-2.601036
C	1.400978	-0.298328	-0.510708
C	2.085663	0.910983	-0.620108
C	0.798279	-0.637784	0.700236
C	2.184473	1.786779	0.460203
H	2.519715	1.170633	-1.573939
C	0.864165	0.216180	1.795334
H	0.242879	-1.557991	0.749122
C	1.567479	1.414994	1.654030
H	1.619130	2.088856	2.497203
H	-0.177524	-2.044583	-3.033623
C	-1.283935	-1.081222	-1.502203
C	-2.258501	-1.621589	-0.674888
C	-1.382322	0.252432	-1.894804
C	-3.328359	-0.848988	-0.218074
H	-2.132773	-2.651816	-0.382822
C	-2.410088	1.067787	-1.429553
H	-0.616285	0.664426	-2.534958
C	-3.374832	0.492051	-0.597568
H	-4.178075	1.112286	-0.226157
C	-2.476759	2.567005	-1.740330
C	-4.428500	-1.417337	0.686734
C	2.918742	3.128552	0.370657
C	0.159449	-0.098910	3.117525
C	4.044836	3.163986	1.419318
H	4.764140	2.361800	1.244101
H	4.577847	4.115497	1.368372
H	3.655226	3.051652	2.430964
C	3.540690	3.361674	-1.010220
H	4.271845	2.591352	-1.260500
H	2.784950	3.384431	-1.796561
H	4.056018	4.323101	-1.018522
C	1.927067	4.271991	0.651719
H	1.480341	4.181857	1.641701
H	2.438442	5.235332	0.599560
H	1.120059	4.275277	-0.082332
C	1.183037	-0.096841	4.266016
H	1.677072	0.869631	4.366188
H	0.686521	-0.318170	5.212965
H	1.952306	-0.853806	4.101736
C	-0.905987	0.981586	3.379785
H	-1.442397	0.767374	4.306439
H	-0.456509	1.970531	3.471841
H	-1.630222	1.015351	2.565364
C	-0.533047	-1.465568	3.090579
H	-1.289618	-1.519927	2.308953
H	0.179692	-2.275996	2.929763
H	-1.027534	-1.642163	4.046843
C	-2.297673	3.348074	-0.424545
H	-1.343613	3.101289	0.043738
H	-2.318341	4.423695	-0.613839
H	-3.089824	3.115165	0.287303
C	-1.378389	3.011049	-2.712805
H	-1.450242	2.489729	-3.668690
H	-1.475673	4.080194	-2.908016
H	-0.382286	2.838716	-2.304105
C	-3.839230	2.920758	-2.360770
H	-3.889124	3.990060	-2.577129
H	-3.993101	2.377878	-3.295155
H	-4.664069	2.677948	-1.691250
C	-5.786604	-1.293746	-0.027414
H	-6.583309	-1.700372	0.599490
H	-6.029618	-0.254034	-0.247453
H	-5.780731	-1.844691	-0.969658
C	-4.195764	-2.893530	1.026046
H	-4.181549	-3.517098	0.131372
H	-3.255019	-3.042411	1.557318
H	-5.002010	-3.250663	1.669056
C	-4.479197	-0.625595	2.005756
H	-3.535327	-0.709098	2.544897
H	-4.675832	0.432561	1.834109
H	-5.272575	-1.014173	2.648059

H-gauche'-0-tBu

Number of imaginary frequencies: 0
Electronic Energy (SCF) = -1801.12538901 a.u
ZPV corrected Energy = -1800.346015 a.u.
Enthalpy = -1800.303497 a.u.
Free Energy = -1800.419782 a.u.

S	0.886265	3.190340	-1.627314
C	0.494446	4.535316	-0.407836
H	-0.320248	4.177193	0.217640
H	0.159795	5.392216	-0.986853
C	2.279375	2.537887	-0.562281

H	3.170917	2.631674	-1.176823
H	2.080297	1.487631	-0.387765
C	2.352146	3.384317	0.701360
H	3.379550	3.457928	1.056798
H	1.765125	2.924135	1.496230
C	1.773360	4.767835	0.374582
H	1.575906	5.342024	1.280035
H	2.483829	5.336460	-0.229248
C	-0.382409	2.124138	-1.830645
C	1.862138	0.038888	-3.205453
O	2.798911	0.757458	-3.501324
H	-0.689942	2.071056	-2.864047
C	-1.181690	1.496995	-0.813762
C	-2.424174	0.919669	-1.171190
C	-0.808371	1.423436	0.535702
C	-3.266348	0.347719	-0.231868
H	-2.716057	0.963784	-2.210365
C	-1.625207	0.836847	1.502475
H	0.144071	1.824132	0.838209
C	-2.854507	0.316401	1.111241
H	-3.506218	-0.126621	1.846041
H	0.967568	-0.005773	-3.850703
C	1.811082	-0.843075	-2.029963
C	0.688347	-1.653669	-1.848465
C	2.851925	-0.864829	-1.107411
C	0.599403	-2.501653	-0.752511
H	-0.104489	-1.601497	-2.579876
C	2.778324	-1.671224	0.025579
H	3.708165	-0.230539	-1.283628
C	1.650447	-2.478998	0.173124
H	1.579340	-3.118038	1.038791
C	-1.132699	0.785362	2.954448
C	-4.651929	-0.201154	-0.599085
C	3.916214	-1.644635	1.050671
C	-0.581619	-3.449712	-0.530308
C	5.207065	-2.150057	0.382405
H	5.085163	-3.174818	0.027205
H	5.485317	-1.528770	-0.469118
H	6.033065	-2.132991	1.096230
C	4.130150	-0.199702	1.537344
H	4.392829	0.467938	0.717399
H	3.228824	0.185905	2.014915
H	4.941386	-0.167827	2.267117
C	3.622537	-2.522047	2.271753
H	3.504703	-3.571759	1.999816
H	4.453923	-2.454161	2.974665
H	2.719518	-2.199415	2.792047
C	-0.064468	-4.899196	-0.492631
H	0.648720	-5.051137	0.317745
H	-0.896387	-5.589788	-0.340774
H	0.428774	-5.161745	-1.430286
C	-1.263152	-3.118255	0.808417
H	-1.632753	-2.094212	0.811896
H	-2.108947	-3.788219	0.974541
H	-0.576438	-3.235209	1.646765
C	-1.622007	-3.338272	-1.648921
H	-2.035636	-2.332414	-1.714137
H	-1.198243	-3.599385	-2.619947
H	-2.444779	-4.025991	-1.450639
C	0.219285	0.051087	3.009289
H	0.123667	-0.965524	2.627881
H	0.976235	0.559270	2.414058
H	0.581074	-0.001000	4.038474
C	-2.110238	0.053118	3.879731
H	-3.080806	0.549380	3.919272
H	-2.267805	-0.978942	3.563436
H	-1.706972	0.033164	4.893526
C	-0.948427	2.219331	3.483617
H	-1.891073	2.768768	3.452262
H	-0.599855	2.200499	4.518641
H	-0.217376	2.773261	2.894369
C	-5.722965	0.700028	0.043744
H	-6.723419	0.338710	-0.205086
H	-5.629397	0.714139	1.129909
H	-5.632618	1.726750	-0.315507
C	-4.822272	-1.634867	-0.066950
H	-4.091722	-2.306426	-0.515674
H	-4.705733	-1.685077	1.014885
H	-5.819640	-2.006226	-0.311854
C	-4.890878	-0.229009	-2.112715
H	-4.864032	0.770407	-2.548205
H	-4.150242	-0.844375	-2.625700
H	-5.875776	-0.651761	-2.317852

H-gauche'-TS0-tBu

Number of imaginary frequencies: 1

Electronic Energy (SCF) = -1801.11056531 a.u

ZPV corrected Energy = -1800.329683 a.u.

Enthalpy = -1800.288938 a.u.

Free Energy = -1800.399445 a.u.

S	0.541950	3.443238	-1.384983
C	-0.396666	4.373961	-0.119985
H	-1.035227	3.675621	0.416380
H	-1.006224	5.111989	-0.634802
C	1.983580	3.147987	-0.279417
H	2.824013	3.588782	-0.807125
H	2.145983	2.081146	-0.203121
C	1.684332	3.845511	1.047787
H	2.607318	4.224555	1.484460
H	1.252073	3.134387	1.749611
C	0.680538	4.968570	0.778851
H	0.235285	5.345346	1.699198
H	1.169831	5.805150	0.276604
C	-0.319147	1.924360	-1.729455
C	1.109828	0.959762	-2.712803
O	2.035175	1.790654	-3.006799
H	-0.939388	2.196448	-2.580329
C	-1.105184	1.256198	-0.681822
C	-2.392124	0.799174	-1.014570
C	-0.619614	0.984964	0.597450
C	-3.176668	0.100889	-0.107081
H	-2.761806	1.009719	-2.007238
C	-1.384990	0.296125	1.541292
H	0.376498	1.298821	0.860541
C	-2.654712	-0.135351	1.171153
H	-3.261445	-0.667299	1.885673
H	0.410088	0.652134	-3.512643
C	1.408815	-0.217130	-1.819663
C	0.509517	-1.282122	-1.739318
C	2.585576	-0.266535	-1.088971
C	0.759765	-2.376787	-0.922861
H	-0.393725	-1.231616	-2.328942
C	2.865361	-1.337361	-0.234220
H	3.288711	0.544079	-1.212332
C	1.939154	-2.373665	-0.163984
H	2.131289	-3.208648	0.490805
C	-0.824158	0.062713	2.949559
C	-4.596600	-0.374565	-0.436825
C	4.176283	-1.346790	0.560303
C	-0.209686	-3.558493	-0.810169
C	5.351626	-1.493723	-0.423206
H	5.274182	-2.427528	-0.982845
H	5.372824	-0.672820	-1.140379
H	6.301359	-1.497263	0.116412
C	4.333775	-0.025185	1.333626
H	4.347124	0.835938	0.666316

H	3.518228	0.109384	2.045126
H	5.272292	-0.027831	1.891688
C	4.239585	-2.497165	1.570930
H	4.205545	-3.471409	1.082051
H	5.175872	-2.441068	2.128475
H	3.419577	-2.446404	2.289108
C	0.535350	-4.877468	-1.081178
H	1.332008	-5.051426	-0.358260
H	-0.158122	-5.718868	-1.020494
H	0.979592	-4.875138	-2.078216
C	-0.801300	-3.592258	0.610552
H	-1.346684	-2.673103	0.822009
H	-1.492278	-4.432079	0.713210
H	-0.020937	-3.704036	1.363613
C	-1.364969	-3.452335	-1.811219
H	-1.957985	-2.553588	-1.648851
H	-1.004233	-3.442136	-2.841045
H	-2.026890	-4.312136	-1.697066
C	0.588484	-0.538677	2.866485
H	0.576611	-1.490861	2.338463
H	1.279489	0.120053	2.345095
H	0.982783	-0.706387	3.870660
C	-1.696728	-0.888904	3.775961
H	-2.695345	-0.485063	3.946002
H	-1.798790	-1.861573	3.292766
H	-1.235693	-1.046951	4.752072
C	-0.753135	1.415707	3.680358
H	-1.742661	1.870124	3.756167
H	-0.360727	1.282938	4.690938
H	-0.101067	2.113420	3.155263
C	-5.595177	0.398503	0.444650
H	-6.617323	0.080977	0.227653
H	-5.406375	0.226061	1.504500
H	-5.526036	1.472064	0.259851
C	-4.731014	-1.879568	-0.145182
H	-4.045423	-2.459716	-0.762612
H	-4.521555	-2.111460	0.898523
H	-5.748041	-2.211718	-0.363097
C	-4.965340	-0.139243	-1.905413
H	-4.956031	0.920577	-2.162936
H	-4.284845	-0.660174	-2.580579
H	-5.972456	-0.515032	-2.091876

H-gauche'-tBu

Number of imaginary frequencies: 0
Electronic Energy (SCF) = -1801.12034115 a.u
ZPV corrected Energy = -1800.337253 a.u.
Enthalpy = -1800.296534 a.u.
Free Energy = -1800.407433 a.u.

S	-0.560249	3.363950	-1.489551
C	-2.065508	3.722111	-0.474561
H	-2.462921	2.803735	-0.049830
H	-2.806701	4.167636	-1.134551
C	0.571540	3.631560	-0.069603
H	1.231948	4.424257	-0.413360
H	1.164120	2.737111	0.067844
C	-0.269118	4.036855	1.138292
H	0.290735	4.724901	1.771225
H	-0.507589	3.154284	1.730848
C	-1.562641	4.662533	0.613890
H	-2.308344	4.779104	1.400045
H	-1.362066	5.652226	0.197676
C	-0.768464	1.553916	-1.855519
C	0.673936	1.271049	-2.468370
O	1.273865	2.478939	-2.617511
H	-1.477534	1.611192	-2.682437
C	-1.372694	0.708083	-0.781923
C	-2.565791	0.038288	-1.051435
C	-0.784027	0.564800	0.473560
C	-3.175648	-0.770741	-0.093451
H	-3.010373	0.158286	-2.028206
C	-1.382384	-0.199311	1.470311
H	0.152609	1.054280	0.668342
C	-2.573144	-0.859569	1.161445
H	-3.049768	-1.458957	1.922696
H	0.453527	0.769714	-3.433300
C	1.456621	0.239075	-1.658028
C	1.067949	-1.102402	-1.672979
C	2.560822	0.614031	-0.916120
C	1.746433	-2.059918	-0.932838
H	0.213012	-1.380347	-2.272223
C	3.273765	-0.317245	-0.149697
H	2.861007	1.650814	-0.958808
C	2.844588	-1.640029	-0.165347
H	3.376183	-2.376029	0.417141
C	-0.803984	-0.271906	2.887937
C	-4.469807	-1.543886	-0.366301
C	4.500568	0.145160	0.644364
C	1.345341	-3.539237	-0.931906
C	5.575492	0.634434	-0.343365
H	5.874605	-0.168390	-1.019645
H	5.210725	1.464570	-0.948373
H	6.462357	0.972908	0.197068
C	4.114193	1.306019	1.578389
H	3.708777	2.151802	1.023742
H	3.366686	0.988523	2.305831
H	4.992438	1.655456	2.125287
C	5.102394	-0.974102	1.500414
H	5.454978	-1.807062	0.890804
H	5.957248	-0.587265	2.057434
H	4.381774	-1.361344	2.222566
C	2.529630	-4.391451	-1.422525
H	3.399960	-4.280560	-0.775752
H	2.254630	-5.448299	-1.436628
H	2.822788	-4.102698	-2.433494
C	0.967798	-3.969076	0.495987
H	0.100473	-3.409361	0.845957
H	0.716983	-5.031911	0.517445
H	1.784973	-3.802182	1.197677
C	0.143483	-3.816603	-1.841639
H	-0.737759	-3.256873	-1.526007
H	0.354947	-3.562966	-2.881393
H	-0.105089	-4.878404	-1.802148
C	0.632005	0.260767	2.947098
H	1.285977	-0.288942	2.270494
H	0.687672	1.318395	2.689588
H	1.021594	0.149170	3.959947
C	-0.791411	-1.718289	3.407768
H	-1.789063	-2.154429	3.440519
H	-0.163776	-2.351364	2.781179
H	-0.388996	-1.741360	4.422178
C	-1.690262	0.586360	3.810009
H	-2.715943	0.215617	3.827842
H	-1.304857	0.569275	4.831701
H	-1.712892	1.624021	3.472199
C	-5.556418	-1.091152	0.625392
H	-6.484645	-1.635792	0.441541
H	-5.259891	-1.274113	1.658113
H	-5.761178	-0.024519	0.516640
C	-4.207462	-3.049309	-0.178730
H	-3.440450	-3.399169	-0.871599
H	-3.874168	-3.275572	0.834045
H	-5.120857	-3.617001	-0.367405
C	-4.993368	-1.320913	-1.788787
H	-5.226022	-0.271482	-1.975535
H	-4.275010	-1.650821	-2.540502

H -5.910345 -1.894182 -1.931912

H-anti-0-tBu
Number of imaginary frequencies: 0
Electronic Energy (SCF) = -1801.11965155 a.u
ZPV corrected Energy = -1800.340346 a.u.
Enthalpy = -1800.297758 a.u.
Free Energy = -1800.415926 a.u.

S	0.528634	-0.881700	1.168606
C	-0.016444	-1.985608	2.556539
H	-1.095909	-1.898807	2.653488
H	0.460897	-1.607454	3.457130
C	0.445311	-2.285029	-0.069515
H	1.444421	-2.346203	-0.488271
H	-0.247816	-1.981814	-0.848634
C	0.045783	-3.553856	0.673590
H	0.536086	-4.425823	0.240988
H	-1.029515	-3.708913	0.595884
C	0.427879	-3.378004	2.150894
H	-0.042673	-4.136053	2.777067
H	1.509000	-3.474322	2.273022
C	-0.535212	0.357619	0.842651
C	0.826220	1.710818	-1.859513
H	0.332913	0.993186	-2.540404
O	0.332855	2.803528	-1.659292
C	2.086887	1.229099	-1.274106
C	2.616878	0.024267	-1.731210
C	2.727687	1.951635	-0.267026
C	3.801687	-0.476032	-1.197649
H	2.085156	-0.510416	-2.504727
C	3.896480	1.469289	0.305963
H	2.277852	2.875566	0.060913
C	4.410803	0.261788	-0.181890
H	5.322600	-0.120586	0.253870
H	-0.031834	1.310056	0.823413
C	-1.931163	0.290589	0.513590
C	-2.608805	1.493788	0.214692
C	-2.675543	-0.904056	0.451959
C	-3.952739	1.516071	-0.128697
H	-2.040004	2.410532	0.251731
C	-4.025876	-0.910826	0.111231
H	-2.185079	-1.835259	0.675648
C	-4.651397	0.303469	-0.175750
H	-5.698145	0.308897	-0.440664
C	-4.843143	-2.208069	0.044098
C	-4.691609	2.818450	-0.462243
C	4.441783	-1.784141	-1.671733
C	4.616302	2.186831	1.450787
C	5.851031	-1.488264	-2.215593
H	6.491774	-1.047277	-1.451995
H	6.323107	-2.410792	-2.558959
H	5.804278	-0.795846	-3.057803
C	3.628362	-2.457385	-2.781973
H	2.622341	-2.716516	-2.449522
H	3.542371	-1.819914	-3.662969
H	4.124700	-3.379648	-3.086543
C	4.551181	-2.761586	-0.487558
H	3.566354	-2.994768	-0.080605
H	5.012547	-3.695597	-0.813653
H	5.158474	-2.351521	0.319120
C	6.061536	2.508339	1.030930
H	6.583187	3.017655	1.843431
H	6.622856	1.606170	0.787555
H	6.075485	3.160901	0.156261
C	3.922645	3.496521	1.838281
H	3.890667	4.199182	1.004433
H	2.901179	3.327096	2.181323
H	4.472041	3.970925	2.652494
C	4.635320	1.265521	2.684473
H	5.140637	1.760935	3.515781
H	3.619481	1.020966	2.999671
H	5.159280	0.331281	2.481898
C	-4.009351	-3.448318	0.383122
H	-3.180920	-3.580423	-0.313665
H	-3.601825	-3.398897	1.393710
H	-4.638559	-4.337796	0.322745
C	-6.011317	-2.129444	1.043585
H	-6.601644	-3.047859	1.009628
H	-5.641422	-2.000748	2.062485
H	-6.675444	-1.294975	0.819842
C	-5.406201	-2.386563	-1.377484
H	-5.988494	-3.308503	-1.441276
H	-6.056458	-1.558741	-1.659058
H	-4.598567	-2.443527	-2.109656
C	-5.873851	2.999643	0.506934
H	-6.409898	3.924128	0.281120
H	-6.582752	2.174925	0.435676
H	-5.523549	3.053808	1.539368
C	-3.789838	4.052478	-0.351215
H	-3.397249	4.177207	0.658940
H	-2.945852	4.000932	-1.039910
H	-4.365669	4.946435	-0.596644
C	-5.227346	2.744791	-1.903722
H	-5.920804	1.913789	-2.032495
H	-5.755241	3.666309	-2.159121
H	-4.408968	2.614157	-2.614069

H-anti-TS0-tBu
Number of imaginary frequencies: 1
Electronic Energy (SCF) = -1801.10427804 a.u
ZPV corrected Energy = -1800.324405 a.u.
Enthalpy = -1800.283247 a.u.
Free Energy = -1800.397509 a.u.

S	0.422524	-0.398418	1.494813
C	-0.708769	-0.567075	2.945271
H	-1.687851	-0.223743	2.617866
H	-0.332691	0.084838	3.729137
C	0.340147	-2.193819	1.086010
H	1.329609	-2.578895	1.319513
H	0.171164	-2.292313	0.019997
C	-0.733788	-2.809183	1.974606
H	-0.540791	-3.872825	2.110920
H	-1.711243	-2.698624	1.507980
C	-0.706552	-2.047947	3.299854
H	-1.568920	-2.285180	3.922655
H	0.193606	-2.299321	3.863367
C	-0.364705	0.523921	0.205406
C	0.534787	0.356506	-1.535129
H	0.173711	-0.671302	-1.744223
O	0.108231	1.307721	-2.254919
C	1.983808	0.330269	-1.105413
C	2.657962	-0.881246	-0.987709
C	2.645930	1.518011	-0.804505
C	3.980331	-0.932477	-0.545204
H	2.130905	-1.787817	-1.246841
C	3.964441	1.509875	-0.358045
H	2.101365	2.441539	-0.927974
C	4.606979	0.273898	-0.232502
H	5.630901	0.254866	0.112263
H	-0.065945	1.547545	0.416447
C	-1.824268	0.379848	0.026273
C	-2.653020	1.487737	0.224901
C	-2.399370	-0.819523	-0.405073
C	-4.029738	1.414796	0.019379

H -2.196120 2.411487 0.546211
C -3.771472 -0.935337 -0.605642
H -1.759012 -1.661122 -0.605298
C -4.565957 0.191563 -0.383771
H -5.632095 0.115977 -0.540736
C -4.425657 -2.244096 -1.062012
C -4.959238 2.616039 0.225208
C 4.751299 -2.248822 -0.395832
C 4.724499 2.791846 -0.000483
C 5.977000 -2.231785 -1.326678
H 6.649308 -1.407852 -1.087611
H 6.538587 -3.163559 -1.231032
H 5.670421 -2.124488 -2.368563
C 3.894187 -3.468663 -0.750766
H 3.019859 -3.549730 -0.103084
H 3.549937 -3.435578 -1.785141
H 4.485138 -4.377273 -0.626332
C 5.224474 -2.403754 1.060891
H 4.375572 -2.412718 1.747200
H 5.769497 -3.342129 1.182791
H 5.886834 -1.591127 1.358252
C 5.963407 2.920410 -0.905011
H 6.516121 3.830319 -0.661733
H 6.639414 2.074087 -0.782759
H 5.672123 2.969987 -1.955610
C 3.864934 4.047302 -0.181845
H 3.534851 4.166181 -1.214502
H 2.980850 4.028072 0.457046
H 4.449025 4.929504 0.084907
C 5.176333 2.725749 1.469659
H 5.715864 3.635874 1.740084
H 4.316702 2.629977 2.135609
H 5.837998 1.878532 1.650139
C -3.400241 -3.358502 -1.297997
H -2.681619 -3.088667 -2.073094
H -2.848255 -3.601442 -0.389010
H -3.915892 -4.263080 -1.623456
C -5.413981 -2.718196 0.018933
H -5.892502 -3.650290 -0.288693
H -4.899459 -2.896859 0.965024
H -6.197167 -1.981029 0.196033
C -5.188393 -2.007507 -2.377621
H -5.652635 -2.936157 -2.715875
H -5.976107 -1.263867 -2.258886
H -4.512007 -1.659932 -3.160246
C -5.994308 2.280657 1.314192
H -6.666449 3.127106 1.469534
H -6.600786 1.417179 1.040954
H -5.501732 2.059931 2.263069
C -4.199693 3.875271 0.655953
H -3.682545 3.732210 1.605948
H -3.464649 4.178891 -0.090489
H -4.904563 4.698279 0.782963
C -5.691914 2.923783 -1.093284
H -6.297643 2.079846 -1.423143
H -6.353914 3.782683 -0.964998
H -4.979721 3.157655 -1.886317

H-anti-tBu

Number of imaginary frequencies: 0

Electronic Energy (SCF) = -1801.11081789 a.u

ZPV corrected Energy = -1800.329267 a.u.

Enthalpy = -1800.287858 a.u.

Free Energy = -1800.403425 a.u.

S 0.355179 -0.123833 1.478273
C -0.861330 0.292051 2.788885
H -1.727259 0.736021 2.304071
H -0.393347 1.019756 3.446077
C -0.051037 -1.914642 1.482497
H 0.819422 -2.401931 1.915921
H -0.165711 -2.242922 0.455204
C -1.295506 -2.076988 2.349247
H -1.342975 -3.091625 2.743211
H -2.186674 -1.900395 1.749418
C -1.202523 -1.034904 3.461475
H -2.138508 -0.938388 4.010786
H -0.420795 -1.307577 4.171855
C -0.298401 0.557218 -0.163128
C 0.562641 0.034805 -1.392350
H 0.311705 -1.047547 -1.490644
O 0.226984 0.785991 -2.439042
C 2.044078 0.067131 -0.994100
C 2.712721 -1.095590 -0.625287
C 2.723976 1.281697 -0.971451
C 4.046385 -1.067252 -0.211368
H 2.172597 -2.030976 -0.666502
C 4.052430 1.356389 -0.560373
H 2.186796 2.159630 -1.297060
C 4.689177 0.169631 -0.181726
H 5.721504 0.211114 0.135132
H -0.040139 1.608505 -0.037801
C -1.777967 0.388337 -0.294395
C -2.619986 1.429373 0.093090
C -2.333119 -0.797096 -0.772891
C -4.006961 1.302409 0.027431
H -2.169919 2.346558 0.442271
C -3.712281 -0.962251 -0.854400
H -1.674124 -1.585522 -1.095756
C -4.525484 0.096756 -0.443282
H -5.598198 -0.018861 -0.501605
C -4.354952 -2.249837 -1.380314
C -4.960020 2.423606 0.453448
C 4.812125 -2.329019 0.202576
C 4.830304 2.676237 -0.510657
C 6.029688 -2.515375 -0.720541
H 6.709502 -1.665402 -0.663144
H 6.586224 -3.411445 -0.437507
H 5.714510 -2.624424 -1.759685
C 3.947209 -3.591144 0.115393
H 3.080232 -3.534817 0.775555
H 3.590778 -3.768738 -0.900018
H 4.537080 -4.458060 0.417015
C 5.297810 -2.179017 1.655624
H 4.454249 -2.041165 2.334810
H 5.842262 -3.073098 1.966846
H 5.964017 -1.324109 1.770226
C 6.050089 2.586837 -1.445496
H 6.613586 3.522111 -1.422496
H 6.723847 1.782080 -1.151316
H 5.736205 2.404165 -2.474614
C 3.976665 3.870924 -0.949040
H 3.625371 3.762170 -1.975728
H 3.106102 4.003857 -0.305120
H 4.572863 4.783305 -0.895526
C 5.313799 2.932113 0.928340
H 5.869834 3.870706 0.979076
H 4.467998 3.000905 1.615085
H 5.968978 2.135477 1.280680
C -3.312757 -3.305571 -1.764328
H -2.650824 -2.952050 -2.555689
H -2.699244 -3.598434 -0.910773
H -3.819958 -4.199556 -2.129709
C -5.268877 -2.847272 -0.295331
H -5.734913 -3.764743 -0.660115
H -4.699135 -3.091400 0.603219
H -6.064507 -2.158109 -0.012821

C	-5.194575	-1.923300	-2.628424
H	-5.658095	-2.831434	-3.019147
H	-5.988476	-1.211214	-2.403036
H	-4.569657	-1.494355	-3.413323
C	-5.857243	1.919196	1.598196
H	-6.545049	2.706589	1.912849
H	-6.450865	1.057147	1.294056
H	-5.257301	1.627725	2.462494
C	-4.213169	3.669310	0.941634
H	-3.590892	3.453918	1.811741
H	-3.576943	4.091032	0.162508
H	-4.934684	4.434269	1.231789
C	-5.840650	2.826197	-0.742927
H	-6.437490	1.988266	-1.102799
H	-6.524644	3.626833	-0.454365
H	-5.227640	3.183799	-1.571782

A-TS1-tBu
Number of imaginary frequencies: 1
Electronic Energy (SCF) = -1801.11286964 a.u
ZPV corrected Energy = -1800.331624 a.u.
Enthalpy = -1800.291086 a.u.
Free Energy = -1800.402413 a.u.

S	2.214088	-2.755145	-0.288724
C	3.863976	-2.248218	-0.897158
H	3.815603	-1.189800	-1.139869
H	4.036602	-2.818038	-1.808879
C	2.886931	-3.953886	0.936782
H	2.394041	-4.906915	0.770817
H	2.605347	-3.564551	1.913252
C	4.401120	-3.994592	0.717494
H	4.644553	-4.755355	-0.024945
H	4.896231	-4.263731	1.649592
C	4.839382	-2.621698	0.210553
H	4.795084	-1.883779	1.011943
H	5.857817	-2.636723	-0.176134
C	1.718795	-1.380456	0.815246
C	0.517800	-1.952806	1.774408
O	0.880811	-1.797081	3.038316
H	0.409820	-3.023475	1.448615
C	-0.812133	-1.309024	1.370439
C	-1.673795	-1.947197	0.479787
C	-1.171286	-0.078105	1.895033
C	-2.888876	-1.375269	0.109017
H	-1.379668	-2.911810	0.089004
C	-2.369971	0.542189	1.536100
H	-0.494413	0.387371	2.597046
C	-3.212157	-0.124629	0.648098
H	-4.151817	0.327526	0.374306
H	2.544623	-1.262485	1.510435
C	1.522414	-0.128607	0.024410
C	2.131481	1.042142	0.473414
C	0.726500	-0.092343	-1.120339
C	1.938693	2.254126	-0.188906
H	2.750224	0.990092	1.356144
C	0.518761	1.096241	-1.812861
H	0.250960	-1.000277	-1.454261
C	1.127541	2.254167	-1.323234
H	0.967116	3.186318	-1.844302
C	-2.713259	1.916539	2.120534
C	-3.891854	-2.086821	-0.807859
C	2.607991	3.556619	0.263619
C	-0.382195	1.170736	-3.049374
C	-1.594946	2.910418	1.761049
H	-1.819653	3.898163	2.169552
H	-0.634401	2.590488	2.161309
H	-1.490518	3.003028	0.679628
C	-2.817583	1.806242	3.652213
H	-3.050233	2.780682	4.087571
H	-3.606877	1.108572	3.938316
H	-1.884012	1.455102	4.091484
C	-4.037443	2.471188	1.585049
H	-4.016194	2.591435	0.500834
H	-4.880904	1.828044	1.840362
H	-4.224729	3.452434	2.024315
C	-5.102156	-2.526467	0.037231
H	-5.593975	-1.668686	0.497194
H	-5.835304	-3.044154	-0.585716
H	-4.792738	-3.204242	0.834708
C	-4.378873	-1.142565	-1.920959
H	-3.549198	-0.809756	-2.544005
H	-5.095327	-1.661976	-2.560709
H	-4.874778	-0.258907	-1.521189
C	-3.288426	-3.328935	-1.473816
H	-2.409150	-3.077767	-2.069422
H	-2.999822	-4.084291	-0.742386
H	-4.025543	-3.780182	-2.139943
C	1.542737	4.643492	0.490842
H	2.022349	5.578250	0.787916
H	0.962614	4.838448	-0.410784
H	0.851113	4.350692	1.280453
C	3.578911	4.024492	-0.836017
H	3.057464	4.209733	-1.775285
H	4.072732	4.950882	-0.535456
H	4.348933	3.272985	-1.020171
C	3.397232	3.383828	1.565629
H	2.756871	3.051028	2.383667
H	4.211580	2.666271	1.456098
H	3.836424	4.340101	1.853434
C	-1.665017	1.935210	-2.673360
H	-1.436210	2.949084	-2.343468
H	-2.333686	2.001405	-3.534369
H	-2.194789	1.428695	-1.866653
C	0.335235	1.912553	-4.190241
H	1.264882	1.407839	-4.459832
H	-0.303709	1.940772	-5.074967
H	0.574501	2.941464	-3.923769
C	-0.771353	-0.220468	-3.561542
H	0.107722	-0.803242	-3.842964
H	-1.330926	-0.785831	-2.818340
H	-1.403594	-0.120551	-4.444817

A-TS2-tBu
Number of imaginary frequencies: 1
Electronic Energy (SCF) = -1801.10346837 a.u
ZPV corrected Energy = -1800.322644 a.u.
Enthalpy = -1800.281955 a.u.
Free Energy = -1800.394578 a.u.

S	0.282385	-1.135732	1.217409
C	-0.859504	-2.016446	2.356430
H	-1.875417	-1.808732	2.033492
H	-0.696608	-1.566285	3.334794
C	1.526905	-2.495526	1.233122
H	2.499061	-2.049695	1.412442
H	1.511811	-2.932548	0.239480
C	1.079856	-3.480756	2.311301
H	1.457791	-3.162341	3.283787
H	1.492161	-4.466287	2.097290
C	-0.446544	-3.479648	2.323948
H	-0.836560	-3.959518	1.425277
H	-0.850230	-4.005730	3.188480
C	-0.503163	-1.287876	-0.438265
C	0.473661	-0.742727	-1.706999
O	-0.175824	0.090179	-2.487132

H	0.721288	-1.729521	-2.167555
C	1.802845	-0.208759	-1.155095
C	2.969762	-0.968210	-1.242200
C	1.868086	1.053997	-0.583867
C	4.183030	-0.499103	-0.745907
H	2.908055	-1.942189	-1.706958
C	3.058395	1.560220	-0.058088
H	0.966619	1.648092	-0.557537
C	4.200701	0.766832	-0.147353
H	5.133347	1.137960	0.247755
H	-0.652167	-2.357565	-0.569360
C	-1.824930	-0.596613	-0.356606
C	-2.994561	-1.334925	-0.519789
C	-1.905799	0.773733	-0.117841
C	-4.247943	-0.725901	-0.452811
H	-2.910135	-2.395176	-0.706292
C	-3.137558	1.415963	-0.035463
H	-0.994615	1.334730	-0.006805
C	-4.290719	0.646310	-0.206062
H	-5.253292	1.134063	-0.148406
C	3.058840	2.942230	0.603986
C	5.479009	-1.314374	-0.819738
C	-5.555148	-1.505219	-0.631608
C	-3.260450	2.921714	0.219225
C	2.078810	2.929249	1.791510
H	2.057183	3.907302	2.276860
H	1.065423	2.692028	1.469667
H	2.378869	2.187331	2.534164
C	2.600412	3.999475	-0.416354
H	2.585608	4.988935	0.045648
H	3.278415	4.032682	-1.270930
H	1.599145	3.788846	-0.790878
C	4.441976	3.344443	1.125878
H	4.809951	2.646795	1.879831
H	5.177794	3.397050	0.322166
H	4.383736	4.331301	1.587734
C	6.535889	-0.529318	-1.617078
H	6.759445	0.430167	-1.150927
H	7.465726	-1.099089	-1.676555
H	6.189079	-0.336759	-2.633769
C	6.005240	-1.566820	0.605024
H	5.277264	-2.127790	1.194464
H	6.930270	-2.146203	0.569799
H	6.213911	-0.633708	1.128030
C	5.275563	-2.672079	-1.500728
H	4.557927	-3.292472	-0.961522
H	4.925133	-2.561417	-2.527650
H	6.223955	-3.210838	-1.528891
C	-6.334922	-0.924962	-1.825221
H	-7.269102	-1.472165	-1.967615
H	-6.581435	0.125387	-1.671289
H	-5.750816	-1.002241	-2.743707
C	-6.407040	-1.372590	0.643673
H	-6.649250	-0.331975	0.859004
H	-7.345527	-1.919046	0.529585
H	-5.879182	-1.781138	1.507534
C	-5.314129	-2.995365	-0.895215
H	-4.735717	-3.156393	-1.805902
H	-4.788633	-3.474054	-0.067317
H	-6.272483	-3.502331	-1.016515
C	-3.996128	3.578823	-0.962375
H	-4.999529	3.171791	-1.087857
H	-4.087779	4.654466	-0.798054
H	-3.449293	3.421147	-1.893316
C	-4.061586	3.158013	1.512016
H	-3.560123	2.702556	2.368124
H	-4.158925	4.228271	1.705540
H	-5.064849	2.736953	1.446128
C	-1.893339	3.598007	0.368788
H	-1.330903	3.193275	1.211160
H	-1.288208	3.486173	-0.531368
H	-2.032846	4.665441	0.545823

B-TS1-tBu

Number of imaginary frequencies: 1

Electronic Energy (SCF) = -1801.11176874 a.u

ZPV corrected Energy = -1800.330494 a.u.

Enthalpy = -1800.290003 a.u.

Free Energy = -1800.401378 a.u.

S	-1.928825	-2.904130	0.288282
C	-3.601940	-2.488309	0.933282
H	-3.833345	-1.490373	0.567954
H	-3.545951	-2.469625	2.017900
C	-2.547825	-4.127352	-0.930779
H	-2.311811	-5.107468	-0.522284
H	-1.995585	-3.964997	-1.854552
C	-4.048295	-3.883303	-1.036142
H	-4.539045	-4.765434	-1.445839
H	-4.240305	-3.047932	-1.710392
C	-4.541307	-3.553328	0.370731
H	-5.564381	-3.178983	0.372342
H	-4.509194	-4.446301	0.996717
C	-1.496480	-1.513333	-0.823254
C	-0.221598	-2.032482	-1.702564
O	-0.559155	-2.019646	-2.985353
H	-0.019753	-3.058524	-1.287778
C	1.032191	-1.239663	-1.330179
C	1.215544	0.050605	-1.813506
C	2.004180	-1.796963	-0.509896
C	2.339858	0.798061	-1.473567
H	0.454891	0.463008	-2.461073
C	3.156562	-1.091047	-0.155391
H	1.855412	-2.808014	-0.150693
C	3.299347	0.207485	-0.644177
H	4.173883	0.773870	-0.373804
H	-2.301742	-1.476680	-1.551778
C	-1.455595	-0.236097	-0.047076
C	-2.241087	0.826999	-0.484061
C	-0.660628	-0.073883	1.087313
C	-2.239683	2.053595	0.180905
H	-2.855971	0.686184	-1.362107
C	-0.647524	1.128040	1.789958
H	-0.033674	-0.891663	1.411052
C	-1.440270	2.178134	1.316089
H	-1.432925	3.114124	1.846997
C	-3.117710	3.196107	-0.340384
C	0.249967	1.277510	3.023408
C	2.479307	2.231791	-1.998094
C	4.222227	-1.762375	0.719721
C	-2.987294	4.466849	0.505694
H	-3.623491	5.247724	0.086649
H	-3.300700	4.302034	1.537597
H	-1.963110	4.842237	0.515155
C	-2.711683	3.537018	-1.785262
H	-1.679627	3.886491	-1.825108
H	-2.799979	2.673179	-2.443810
H	-3.354248	4.327376	-2.178550
C	-4.591830	2.751654	-0.320349
H	-5.233357	3.558450	-0.680409
H	-4.758238	1.882708	-0.957101
H	-4.905991	2.494237	0.692896
C	0.004763	0.109321	3.994816
H	0.234703	-0.853700	3.540391
H	0.637614	0.218803	4.877457
H	-1.035882	0.088346	4.324058
C	-0.004156	2.585936	3.780215

H	0.225342	3.460565	3.170693
H	-1.039335	2.665151	4.116621
H	0.637360	2.623363	4.661888
C	1.718692	1.256182	2.565795
H	1.947695	0.346669	2.016048
H	1.931042	2.101262	1.910021
H	2.386876	1.315734	3.427730
C	5.343512	-0.797347	1.120326
H	6.058151	-1.315753	1.761679
H	5.889182	-0.424510	0.252739
H	4.957739	0.059690	1.674673
C	3.584985	-2.310000	2.009446
H	2.797838	-3.033064	1.798030
H	4.341756	-2.810229	2.617361
H	3.153341	-1.504577	2.604351
C	4.844251	-2.931905	-0.065537
H	5.307617	-2.576379	-0.987495
H	5.611833	-3.427550	0.533193
H	4.091812	-3.674335	-0.332842
C	3.760890	2.917001	-1.512361
H	3.796506	2.986154	-0.424198
H	4.655402	2.390961	-1.849276
H	3.803651	3.932124	-1.910618
C	2.495932	2.212266	-3.537079
H	1.584045	1.773073	-3.941028
H	2.583311	3.228416	-3.928194
H	3.341744	1.630835	-3.908618
C	1.275134	3.061868	-1.518512
H	1.346023	4.085351	-1.893719
H	0.336096	2.638045	-1.868762
H	1.236595	3.098961	-0.429039

B-TS2-tBu
Number of imaginary frequencies: 1
Electronic Energy (SCF) = -1801.10257500 a.u
ZPV corrected Energy = -1800.322033 a.u.
Enthalpy = -1800.281266 a.u.
Free Energy = -1800.394200 a.u.

S	0.259652	-0.996736	1.247332
C	-0.931490	-1.761986	2.430404
H	-1.742805	-2.176291	1.837397
H	-1.318914	-0.968298	3.062652
C	1.473637	-2.368086	1.399979
H	2.305920	-1.959933	1.969075
H	1.817371	-2.620198	0.403957
C	0.760900	-3.495420	2.136106
H	1.492621	-4.162139	2.591155
H	0.164450	-4.078903	1.433477
C	-0.145628	-2.837003	3.171735
H	-0.833283	-3.546414	3.631235
H	0.453997	-2.387953	3.965114
C	-0.496017	-1.236023	-0.412089
C	0.460993	-0.675668	-1.692085
O	-0.173999	0.204381	-2.430755
H	0.661151	-1.656444	-2.187768
C	1.817948	-0.200451	-1.156003
C	1.933136	1.054116	-0.574721
C	2.957064	-0.998062	-1.267953
C	3.148110	1.515701	-0.064122
H	1.051466	1.677073	-0.531678
C	4.193208	-0.573642	-0.787334
H	2.856162	-1.963823	-1.743684
C	4.261722	0.685477	-0.178054
H	5.212597	1.022407	0.203825
H	-0.599255	-2.315465	-0.507001
C	-1.841811	-0.590754	-0.358680
C	-2.989448	-1.361108	-0.526381
C	-1.959055	0.780741	-0.140951
C	-4.258317	-0.781952	-0.483050
H	-2.876201	-2.421162	-0.697898
C	-3.206816	1.393241	-0.082557
H	-1.061601	1.364351	-0.032789
C	-4.338174	0.592112	-0.256158
H	-5.313509	1.055861	-0.217926
C	-5.543975	-1.595886	-0.664694
C	-3.368536	2.899663	0.145270
C	5.462302	-1.426625	-0.895697
C	3.208096	2.895546	0.599671
C	-4.089510	3.520728	-1.064670
H	-5.080106	3.088497	-1.206878
H	-4.209257	4.596620	-0.921151
H	-3.516760	3.359850	-1.979317
C	-2.020272	3.608418	0.316396
H	-1.469089	3.227388	1.177390
H	-1.391649	3.498414	-0.567772
H	-2.187806	4.674671	0.475371
C	-4.205451	3.140298	1.414342
H	-4.328459	4.211378	1.587621
H	-5.198563	2.698782	1.331778
H	-3.716233	2.709041	2.289849
C	-6.327648	-1.049341	-1.871579
H	-7.245869	-1.622410	-2.016035
H	-6.603082	-0.004235	-1.731537
H	-5.733457	-1.121291	-2.784014
C	-5.263352	-3.082435	-0.908780
H	-4.671305	-3.239151	-1.811418
H	-4.735168	-3.538671	-0.069995
H	-6.207559	-3.614308	-1.034224
C	-6.410643	-1.470337	0.601410
H	-6.681722	-0.433832	0.801657
H	-7.333553	-2.042423	0.485839
H	-5.880144	-1.854300	1.474881
C	6.526047	-0.664158	-1.706122
H	6.789932	0.281630	-1.233106
H	7.435926	-1.261955	-1.792775
H	6.163962	-0.447669	-2.712616
C	6.010175	-1.711056	0.514791
H	5.279228	-2.259378	1.112209
H	6.918031	-2.314882	0.453307
H	6.255371	-0.790691	1.044554
C	5.205729	-2.769641	-1.587847
H	4.481515	-3.375962	-1.041583
H	4.838498	-2.636556	-2.606191
H	6.137432	-3.334958	-1.641272
C	4.605233	3.232724	1.130408
H	4.937232	2.515226	1.882513
H	5.347193	3.256351	0.331052
H	4.589192	4.219005	1.596827
C	2.806439	3.971016	-0.425659
H	3.490955	3.970646	-1.275684
H	1.799002	3.805060	-0.806438
H	2.834420	4.961337	0.034022
C	2.221256	2.932244	1.780907
H	1.197594	2.754748	1.452752
H	2.474178	2.171744	2.522325
H	2.252394	3.907989	2.270254

C-TS1-tBu
Number of imaginary frequencies: 1
Electronic Energy (SCF) = -1801.10416885 a.u
ZPV corrected Energy = -1800.322929 a.u.
Enthalpy = -1800.282598 a.u.
Free Energy = -1800.393023 a.u.

S	-0.394676	-1.370711	0.907581
C	0.925644	-1.968318	2.038287
H	1.644125	-2.535994	1.454636
H	0.418276	-2.636940	2.732047
C	-0.489680	0.286000	1.687186
H	-1.540868	0.508626	1.831840
H	-0.065143	0.993110	0.984677
C	0.315018	0.198189	2.983085
H	-0.313624	-0.196888	3.782196
H	0.640566	1.196195	3.275013
C	1.495009	-0.739860	2.734710
H	2.233808	-0.261494	2.094807
H	1.984583	-1.029381	3.664142
C	0.400184	-1.155075	-0.756831
C	-0.612321	-0.414827	-1.894654
H	-0.718457	-1.278558	-2.594128
O	-0.082607	0.660076	-2.425052
C	-2.003724	-0.226033	-1.266025
C	-2.860364	-1.300478	-1.044125
C	-2.418453	1.049344	-0.908841
C	-4.103986	-1.128185	-0.436438
H	-2.539219	-2.290590	-1.344862
C	-3.651794	1.270293	-0.297339
H	-1.742167	1.862989	-1.128521
C	-4.476861	0.166819	-0.064189
H	-5.433309	0.319133	0.405950
H	0.467574	-2.205810	-1.039705
C	1.771261	-0.581265	-0.635952
C	2.871778	-1.438572	-0.607843
C	1.971722	0.793494	-0.497223
C	4.162984	-0.954378	-0.403950
H	2.700705	-2.496397	-0.740847
C	3.244595	1.311849	-0.282200
H	1.119674	1.438052	-0.607191
C	4.318889	0.421116	-0.230611
H	5.311380	0.813499	-0.060645
C	-4.051146	2.696519	0.098367
C	-5.002144	-2.345696	-0.188039
C	3.493815	2.811933	-0.095653
C	5.390562	-1.869734	-0.346427
C	-4.093799	3.579689	-1.161514
H	-4.367866	4.603475	-0.897566
H	-3.126590	3.607895	-1.662700
H	-4.830336	3.203008	-1.873487
C	-3.005499	3.265061	1.075477
H	-3.269846	4.285789	1.359927
H	-2.955349	2.663639	1.985208
H	-2.011629	3.288400	0.629338
C	-5.423731	2.758362	0.776699
H	-6.216977	2.399407	0.119471
H	-5.449546	2.169019	1.694704
H	-5.653573	3.792117	1.039587
C	-6.329741	-1.971035	0.479700
H	-6.177215	-1.506963	1.455267
H	-6.913332	-1.285439	-0.136201
H	-6.927028	-2.871524	0.631175
C	-4.266086	-3.338628	0.730341
H	-4.894014	-4.210246	0.926974
H	-3.337471	-3.689097	0.279891
H	-4.020986	-2.873431	1.687098
C	-5.316424	-3.034428	-1.528061
H	-4.409177	-3.367846	-2.031471
H	-5.950665	-3.908384	-1.364485
H	-5.842320	-2.353421	-2.199446
C	4.125981	3.052196	1.287306
H	4.312879	4.117460	1.437846
H	3.461177	2.709076	2.082540
H	5.075477	2.527220	1.393054
C	2.200145	3.629227	-0.180729
H	2.429312	4.687370	-0.045728
H	1.711460	3.513857	-1.148567
H	1.488622	3.342635	0.595301
C	4.455260	3.312021	-1.188218
H	4.644857	4.380374	-1.065191
H	5.413549	2.793993	-1.147243
H	4.028412	3.153769	-2.179931
C	6.050753	-1.745883	1.038833
H	6.928724	-2.392396	1.098531
H	6.372357	-0.724112	1.240351
H	5.356435	-2.042238	1.827454
C	6.398672	-1.444083	-1.428675
H	6.729810	-0.415442	-1.287448
H	7.279975	-2.088045	-1.397708
H	5.955342	-1.521452	-2.422868
C	5.032282	-3.341520	-0.577724
H	4.346601	-3.717057	0.183418
H	4.575311	-3.497681	-1.555906
H	5.938590	-3.947174	-0.533871

C-TS3-tBu
Number of imaginary frequencies: 1
Electronic Energy (SCF) = -1801.11983994 a.u
ZPV corrected Energy = -1800.337332 a.u.
Enthalpy = -1800.297243 a.u.
Free Energy = -1800.408152 a.u.

S	0.264255	-2.722489	-0.948671
C	1.476057	-2.475164	-2.337936
H	1.372293	-1.470319	-2.737331
H	1.175019	-3.187231	-3.105538
C	1.486525	-3.621194	0.068936
H	0.960256	-4.478759	0.475102
H	1.758745	-2.965241	0.887475
C	2.673447	-3.962875	-0.825576
H	2.469455	-4.879305	-1.382022
H	3.558840	-4.134856	-0.213286
C	2.862373	-2.796404	-1.795280
H	3.267061	-1.932688	-1.268907
H	3.548752	-3.049105	-2.603825
C	0.109104	-0.967710	-0.338553
C	-0.842134	-1.236208	0.887499
H	-0.356531	-0.761436	1.760459
O	-0.978259	-2.584231	1.013805
C	-2.136391	-0.451153	0.660681
C	-2.127111	0.940648	0.710560
C	-3.318062	-1.111894	0.363551
C	-3.276295	1.684270	0.450531
H	-1.199679	1.445517	0.952529
C	-4.496691	-0.406345	0.106645
H	-3.289096	-2.191899	0.343245
C	-4.452721	0.988274	0.151574
H	-5.351761	1.546222	-0.046621
H	-0.411298	-0.499297	-1.173796
C	1.396595	-0.248152	-0.110149
C	1.862882	0.636026	-1.082562
C	2.150401	-0.452575	1.046396
C	3.082953	1.292658	-0.938129
H	1.253776	0.799414	-1.958512
C	3.378076	0.179190	1.223440
H	1.765725	-1.105471	1.812179
C	3.823891	1.037360	0.216171
H	4.775985	1.532364	0.340899
C	3.631370	2.257884	-1.993562
C	4.244778	-0.045536	2.466301
C	-3.214378	3.215610	0.498459
C	-5.781639	-1.177826	-0.214668

C	2.672532	2.433221	-3.175895
H	2.495380	1.491633	-3.697742
H	1.709017	2.835120	-2.859284
H	3.104705	3.132546	-3.892825
C	4.967133	1.711925	-2.529727
H	5.375336	2.387832	-3.283752
H	5.706407	1.609313	-1.735380
H	4.828803	0.732027	-2.990289
C	3.866641	3.637961	-1.354139
H	4.584855	3.586171	-0.536063
H	4.256056	4.335500	-2.098244
H	2.934745	4.046854	-0.959834
C	5.592495	-0.652847	2.036431
H	6.132022	0.006502	1.356582
H	6.224298	-0.824503	2.910111
H	5.443570	-1.609269	1.531779
C	4.494409	1.300578	3.169457
H	5.110992	1.151598	4.058007
H	5.010371	2.006197	2.518468
H	3.552790	1.755442	3.481664
C	3.585851	-0.997807	3.469698
H	2.629894	-0.612532	3.826887
H	3.415728	-1.985903	3.039817
H	4.238703	-1.121171	4.334787
C	-2.165838	3.715185	-0.512805
H	-2.108226	4.805559	-0.490513
H	-1.173787	3.322460	-0.290496
H	-2.429501	3.409949	-1.527219
C	-4.557403	3.869869	0.156600
H	-5.339015	3.584235	0.861930
H	-4.453957	4.955294	0.198326
H	-4.892158	3.606702	-0.847897
C	-2.805046	3.665956	1.912455
H	-3.530641	3.324852	2.653013
H	-1.828555	3.270513	2.191787
H	-2.753855	4.755772	1.962993
C	-5.556329	-2.040546	-1.469785
H	-4.746339	-2.754756	-1.323651
H	-6.461573	-2.602029	-1.710947
H	-5.304917	-1.416281	-2.329414
C	-6.133057	-2.095347	0.970496
H	-7.043890	-2.658649	0.755951
H	-5.335283	-2.809380	1.173619
H	-6.300419	-1.510715	1.876948
C	-6.975504	-0.253405	-0.476523
H	-7.207305	0.365281	0.391634
H	-6.799006	0.406243	-1.327511
H	-7.858289	-0.854417	-0.700550

C-TS2-tBu

Number of imaginary frequencies: 1

Electronic Energy (SCF) = -1801.10766824 a.u

ZPV corrected Energy = -1800.326026 a.u.

Enthalpy = -1800.285774 a.u.

Free Energy = -1800.395451 a.u.

S	1.476587	-3.076355	-1.522232
C	3.169159	-3.022327	-0.819531
H	3.667570	-2.139034	-1.210113
H	3.668691	-3.912354	-1.198525
C	0.689746	-3.582487	0.053620
H	0.006016	-4.394931	-0.173274
H	0.128887	-2.730323	0.420566
C	1.830675	-3.972243	0.993605
H	2.115680	-5.009931	0.816295
H	1.495246	-3.887970	2.026565
C	3.010052	-3.047610	0.695966
H	2.807613	-2.043045	1.062134
H	3.928720	-3.402377	1.161905
C	0.975248	-1.319873	-1.829650
C	-0.518455	-1.426830	-2.489117
H	-0.808790	-2.500861	-2.317428
O	-0.441199	-1.081571	-3.765434
C	-1.522476	-0.623376	-1.660566
C	-2.408716	-1.258117	-0.792506
C	-1.565191	0.755696	-1.782310
C	-3.331485	-0.533089	-0.041610
H	-2.368164	-2.336245	-0.720861
C	-2.458084	1.524601	-1.034576
H	-0.882467	1.222681	-2.477608
C	-3.332449	0.860178	-0.175784
H	-4.035786	1.432779	0.407998
H	1.575982	-1.107602	-2.711867
C	1.383575	-0.392526	-0.727368
C	2.562933	0.341075	-0.909213
C	0.691339	-0.260492	0.473003
C	3.066808	1.167310	0.086849
H	3.080030	0.244472	-1.851728
C	1.183245	0.533963	1.511111
H	-0.249518	-0.768507	0.599430
C	2.366909	1.233416	1.297058
H	2.759186	1.857413	2.084179
C	-2.439073	3.050173	-1.177742
C	-4.334394	-1.199755	0.907511
C	4.351772	1.982128	-0.086907
C	0.404526	0.625321	2.828308
C	-0.888858	1.420889	2.584185
H	-0.659001	2.442973	2.280772
H	-1.491923	0.967415	1.801235
H	-1.486926	1.462916	3.496906
C	0.046671	-0.787304	3.323854
H	0.947923	-1.377255	3.500842
H	-0.505612	-0.724327	4.263041
H	-0.576322	-1.325167	2.610725
C	1.206182	1.324024	3.932042
H	2.149900	0.813335	4.131594
H	1.425701	2.362308	3.681615
H	0.624972	1.325844	4.855295
C	5.380202	1.540246	0.969998
H	6.302443	2.114547	0.862191
H	5.006928	1.692218	1.982702
H	5.623631	0.482252	0.855422
C	4.035407	3.476503	0.104256
H	3.632820	3.678392	1.096739
H	4.942399	4.071817	-0.018153
H	3.303888	3.814629	-0.631552
C	4.976625	1.796681	-1.473465
H	5.257100	0.758298	-1.656809
H	4.299275	2.112503	-2.267984
H	5.881266	2.401708	-1.547404
C	-4.136820	-0.662716	2.336431
H	-4.865090	-1.116386	3.012105
H	-3.138725	-0.898942	2.707145
H	-4.264759	0.418232	2.383260
C	-4.173967	-2.723378	0.947278
H	-4.340466	-3.174499	-0.031683
H	-3.180979	-3.016211	1.292545
H	-4.903802	-3.148619	1.638004
C	-5.765788	-0.877236	0.441728
H	-5.954414	0.196333	0.444773
H	-5.935575	-1.245705	-0.571439
H	-6.495642	-1.349378	1.103082
C	-1.018827	3.574412	-0.895613
H	-0.289269	3.156385	-1.588223
H	-0.992906	4.661625	-0.996021
H	-0.703152	3.317792	0.115906
C	-3.404473	3.742365	-0.209796

H	-4.440259	3.452611	-0.392077
H	-3.164782	3.515398	0.830217
H	-3.336621	4.823796	-0.338966
C	-2.837935	3.425788	-2.616143
H	-2.155666	2.988315	-3.345000
H	-3.845067	3.072033	-2.844237
H	-2.820479	4.510434	-2.744476

D-TS1-tBu
Number of imaginary frequencies: 1
Electronic Energy (SCF) = -1801.10328434 a.u
ZPV corrected Energy = -1800.322534 a.u.
Enthalpy = -1800.281929 a.u.
Free Energy = -1800.394849 a.u.

S	-0.413969	-1.432944	0.937905
C	0.836580	-2.072680	2.121917
H	1.810705	-1.990514	1.646193
H	0.602659	-3.117260	2.308969
C	-0.542360	0.205772	1.758996
H	-1.540578	0.219366	2.189676
H	-0.478030	0.976349	1.002045
C	0.550140	0.251564	2.821486
H	0.269545	0.947440	3.611317
H	1.481279	0.597204	2.375576
C	0.724065	-1.171760	3.347445
H	1.616301	-1.274576	3.964291
H	-0.137766	-1.461315	3.950686
C	0.432059	-1.208548	-0.684877
C	-0.560268	-0.475118	-1.871031
H	-0.664361	-1.357671	-2.545771
O	0.003880	0.573152	-2.410564
C	-1.957957	-0.245277	-1.273933
C	-2.845041	-1.296022	-1.060497
C	-2.349709	1.043626	-0.939249
C	-4.098626	-1.086912	-0.485137
H	-2.541049	-2.296646	-1.343579
C	-3.589835	1.300611	-0.356562
H	-1.649538	1.838850	-1.152614
C	-4.447906	0.220178	-0.133821
H	-5.410796	0.400452	0.312740
H	0.521595	-2.256029	-0.973864
C	1.789601	-0.610276	-0.547225
C	2.911218	-1.438592	-0.592334
C	1.960470	0.761573	-0.360274
C	4.199191	-0.925600	-0.437537
H	2.759926	-2.495883	-0.752020
C	3.228666	1.308918	-0.195746
H	1.091187	1.390911	-0.387730
C	4.328250	0.448025	-0.234768
H	5.318230	0.863950	-0.112923
C	-3.960586	2.739408	0.020757
C	-5.032208	-2.279550	-0.247495
C	3.449296	2.811604	0.007769
C	5.451613	-1.808044	-0.473831
C	-2.919607	3.288799	1.013835
H	-1.917621	3.286946	0.585422
H	-3.164929	4.317046	1.288039
H	-2.899672	2.691407	1.927332
C	-3.956627	3.613725	-1.245929
H	-4.688909	3.250508	-1.969181
H	-4.209248	4.646174	-0.994676
H	-2.979583	3.613368	-1.728556
C	-5.343801	2.840750	0.672161
H	-6.133243	2.496664	0.002533
H	-5.401715	2.259229	1.593697
H	-5.552592	3.881920	0.923069
C	-4.342268	-3.281952	0.695984
H	-4.995946	-4.136128	0.885155
H	-3.412517	-3.659035	0.270187
H	-4.107947	-2.814458	1.654328
C	-5.332119	-2.972585	-1.588619
H	-5.991654	-3.829022	-1.432632
H	-5.824859	-2.284968	-2.278171
H	-4.421733	-3.332874	-2.067287
C	-6.365039	-1.866360	0.386055
H	-6.223411	-1.397814	1.361138
H	-6.916841	-1.171709	-0.248712
H	-6.988075	-2.750359	0.530714
C	2.131299	3.593669	0.036050
H	1.578475	3.488715	-0.897919
H	1.484752	3.269110	0.852873
H	2.341610	4.654294	0.181787
C	4.179262	3.047574	1.342065
H	4.347249	4.115367	1.496641
H	3.588874	2.673099	2.180488
H	5.148716	2.549868	1.364868
C	4.310272	3.357765	-1.145297
H	4.480028	4.428799	-1.016572
H	5.282103	2.865617	-1.186874
H	3.813049	3.203823	-2.104298
C	6.198691	-1.688609	0.866839
H	7.095608	-2.311360	0.856566
H	6.506113	-0.661968	1.065714
H	5.566811	-2.017500	1.694164
C	6.374643	-1.337160	-1.612000
H	7.272604	-1.957315	-1.650907
H	5.868463	-1.409357	-2.576082
H	6.687339	-0.302336	-1.473287
C	5.117889	-3.285482	-0.706605
H	4.488003	-3.688797	0.087932
H	4.608028	-3.440280	-1.658469
H	6.040342	-3.867611	-0.725541

D-TS3-tBu
Number of imaginary frequencies: 1
Electronic Energy (SCF) = -1801.11882444 a.u
ZPV corrected Energy = -1800.336664 a.u.
Enthalpy = -1800.296434 a.u.
Free Energy = -1800.408189 a.u.

S	0.253131	-2.753363	-0.953835
C	1.379649	-2.538656	-2.411167
H	1.844958	-1.556799	-2.375905
H	0.769978	-2.617553	-3.308381
C	1.520573	-3.656948	0.012225
H	1.096577	-4.652187	0.132341
H	1.584605	-3.197443	0.989382
C	2.812949	-3.661625	-0.794553
H	3.411980	-4.535579	-0.538969
H	3.395723	-2.771018	-0.559231
C	2.419986	-3.644111	-2.271308
H	3.273545	-3.454722	-2.921972
H	1.990917	-4.606924	-2.556775
C	0.127134	-1.005009	-0.348321
C	-0.806785	-1.255526	0.903653
H	-0.296020	-0.768004	1.755615
O	-0.952279	-2.596908	1.057441
C	-2.095549	-0.458629	0.686270
C	-2.064023	0.933707	0.699724
C	-3.292484	-1.108010	0.428605
C	-3.205487	1.688652	0.440102
H	-1.124071	1.429423	0.910189
C	-4.465021	-0.390412	0.176077
H	-3.280296	-2.188544	0.434702

C	-4.398222	1.004028	0.181567
H	-5.291754	1.571093	-0.015729
H	-0.403857	-0.530464	-1.174035
C	1.417643	-0.285417	-0.131123
C	1.824591	0.674620	-1.056910
C	2.211617	-0.535305	0.988742
C	3.014756	1.381569	-0.891983
H	1.186382	0.867596	-1.905959
C	3.415332	0.136833	1.178657
H	1.873365	-1.251159	1.719009
C	3.793709	1.086247	0.226191
H	4.721392	1.621444	0.368525
C	-3.116673	3.219396	0.442809
C	-5.768411	-1.149343	-0.097625
C	3.480976	2.454473	-1.880880
C	4.320796	-0.125287	2.386133
C	5.698291	-0.604337	1.893192
H	6.357288	-0.792180	2.743101
H	5.606581	-1.531264	1.324251
H	6.178438	0.136870	1.254402
C	3.747199	-1.194670	3.321544
H	2.778593	-0.901144	3.728228
H	3.627935	-2.153627	2.815218
H	4.427773	-1.345232	4.160598
C	4.492576	1.177798	3.187094
H	5.136133	1.005673	4.052068
H	4.945321	1.964456	2.583533
H	3.528656	1.541169	3.547408
C	4.844445	2.047919	-2.468046
H	5.191146	2.804701	-3.174454
H	5.602162	1.942491	-1.691681
H	4.770309	1.096774	-2.998443
C	3.624394	3.797577	-1.142583
H	4.351181	3.736115	-0.332704
H	3.957265	4.573653	-1.834624
H	2.669411	4.109165	-0.715979
C	2.495785	2.646197	-3.038781
H	2.371714	1.732134	-3.621410
H	1.513134	2.962731	-2.686149
H	2.872016	3.418833	-3.710602
C	-6.112446	-2.023266	1.122248
H	-7.037080	-2.576333	0.942979
H	-5.323431	-2.745001	1.332351
H	-6.251566	-1.407877	2.012992
C	-6.951495	-0.213049	-0.366233
H	-6.780168	0.416568	-1.240718
H	-7.848274	-0.805425	-0.554851
H	-7.156499	0.436288	0.486115
C	-5.581902	-2.054614	-1.328877
H	-5.336632	-1.461966	-2.212332
H	-4.781517	-2.778103	-1.175687
H	-6.501041	-2.607350	-1.535135
C	-2.676745	3.702868	1.836540
H	-3.396377	3.396696	2.597890
H	-1.703174	3.297752	2.112185
H	-2.605262	4.792568	1.854451
C	-2.075876	3.670979	-0.598791
H	-1.999126	4.760347	-0.609310
H	-1.087200	3.267588	-0.380438
H	-2.360730	3.341071	-1.599666
C	-4.453581	3.887044	0.103037
H	-4.808792	3.601051	-0.888108
H	-5.228568	3.635299	0.828311
H	-4.331094	4.971250	0.111868

D-TS2-tBu

Number of imaginary frequencies: 1

Electronic Energy (SCF) = -1801.10819284 a.u

ZPV corrected Energy = -1800.326708 a.u.

Enthalpy = -1800.286274 a.u.

Free Energy = -1800.397318 a.u.

S	1.534852	-3.207898	-1.532805
C	3.171916	-3.245678	-0.707496
H	3.407764	-2.222247	-0.423022
H	3.898960	-3.603415	-1.431364
C	0.628997	-3.833254	-0.062403
H	0.318275	-4.838939	-0.338198
H	-0.256216	-3.223393	0.084408
C	1.620775	-3.836156	1.096301
H	1.317690	-4.571882	1.840286
H	1.637442	-2.857026	1.571177
C	2.994265	-4.153335	0.506479
H	3.796672	-3.973519	1.221092
H	3.043419	-5.199551	0.201361
C	1.096865	-1.440886	-1.781945
C	-0.384310	-1.469524	-2.512536
H	-0.741119	-2.522850	-2.346510
O	-0.243604	-1.134821	-3.782835
C	-1.352485	-0.598544	-1.707268
C	-2.316602	-1.170611	-0.883432
C	-1.272534	0.783372	-1.795884
C	-3.198507	-0.386279	-0.138797
H	-2.369110	-2.249864	-0.834848
C	-2.115667	1.607895	-1.052855
H	-0.525836	1.206850	-2.452218
C	-3.071835	1.002849	-0.234627
H	-3.740259	1.625709	0.336089
H	1.739921	-1.202411	-2.627136
C	1.424163	-0.534170	-0.640462
C	2.449380	0.396566	-0.815622
C	0.724323	-0.550360	0.566001
C	2.774682	1.316522	0.180711
H	2.980380	0.398473	-1.755327
C	1.033746	0.335456	1.591213
H	-0.093720	-1.236380	0.686355
C	2.055513	1.262482	1.373364
H	2.289377	1.970819	2.154799
C	3.876329	2.367381	0.006818
C	0.268692	0.344491	2.918247
C	-1.957940	3.129524	-1.148321
C	-4.320359	-1.009953	0.702049
C	4.945392	2.170178	1.096527
H	5.738087	2.912779	0.985592
H	4.524631	2.275972	2.096428
H	5.396361	1.178737	1.023231
C	3.264768	3.773462	0.144169
H	4.042354	4.532627	0.038110
H	2.512931	3.946303	-0.626515
H	2.789602	3.913180	1.114957
C	4.560050	2.274061	-1.361141
H	5.040415	1.305679	-1.509331
H	3.854714	2.433569	-2.177724
H	5.331794	3.041577	-1.434247
C	1.257407	0.230504	4.091322
H	0.715448	0.232703	5.039015
H	1.828733	-0.697787	4.029970
H	1.964179	1.059618	4.110356
C	-0.513147	1.665494	3.034982
H	-1.067901	1.694455	3.975221
H	0.154719	2.526932	3.010836
H	-1.224982	1.765332	2.215372
C	-0.726764	-0.816048	3.016493
H	-1.484271	-0.760833	2.237308
H	-0.226844	-1.783292	2.942405
H	-1.237608	-0.778182	3.979494

C	-4.576042	-0.207970	1.988916
H	-5.346048	-0.703762	2.582793
H	-3.674193	-0.135998	2.597225
H	-4.926507	0.802436	1.782789
C	-5.608984	-1.013461	-0.142195
H	-6.434774	-1.456446	0.419709
H	-5.893667	0.001663	-0.422624
H	-5.471538	-1.589742	-1.058519
C	-3.996495	-2.455324	1.107062
H	-3.925194	-3.117763	0.244916
H	-3.058201	-2.513170	1.661324
H	-4.789898	-2.841370	1.749068
C	-0.515092	3.515421	-0.774515
H	0.208597	3.050372	-1.441706
H	-0.385261	4.597998	-0.838273
H	-0.279212	3.202595	0.242769
C	-2.241999	3.577739	-2.593095
H	-2.121614	4.659487	-2.685741
H	-1.562045	3.100840	-3.298938
H	-3.262348	3.322503	-2.885142
C	-2.911039	3.880505	-0.212632
H	-3.956750	3.682177	-0.452009
H	-2.743992	3.614579	0.832259
H	-2.746926	4.954718	-0.312311

E-TS1-tBu

Number of imaginary frequencies: 1
Electronic Energy (SCF) = -1801.10981848 a.u
ZPV corrected Energy = -1800.328128 a.u.
Enthalpy = -1800.287706 a.u.
Free Energy = -1800.399252 a.u.

S	0.334862	-0.992086	1.608221
C	-0.750519	-1.800272	2.845112
H	-1.780867	-1.605957	2.559218
H	-0.541258	-1.303354	3.791240
C	1.592354	-2.340251	1.642292
H	2.562578	-1.876489	1.782883
H	1.545464	-2.800804	0.657594
C	1.181947	-3.282209	2.773910
H	1.609754	-2.937915	3.716473
H	1.570653	-4.279865	2.572984
C	-0.342489	-3.266712	2.862587
H	-0.780368	-3.787896	2.010906
H	-0.704335	-3.745771	3.771846
C	-0.514897	-1.351241	0.034677
H	-0.712324	-2.417763	0.072430
C	0.491793	-1.257189	-1.265610
H	-0.114681	-0.626239	-1.956259
O	0.738072	-2.485627	-1.696476
C	1.749624	-0.412913	-0.953523
C	1.684448	0.945032	-0.623695
C	2.988560	-1.024182	-1.035015
C	2.836182	1.676830	-0.351864
H	0.716636	1.422691	-0.588073
C	4.171966	-0.326829	-0.770702
H	2.993452	-2.067725	-1.314229
C	4.071539	1.017226	-0.429598
H	4.968516	1.580608	-0.226200
C	-1.817245	-0.613321	-0.050219
C	-2.908205	-1.325891	-0.550736
C	-1.979995	0.726817	0.302754
C	-4.151975	-0.722627	-0.720027
H	-2.762001	-2.362494	-0.813185
C	-3.218887	1.355911	0.172834
H	-1.136487	1.278396	0.688202
C	-4.281350	0.614368	-0.345332
H	-5.243413	1.092814	-0.455200
C	-5.363721	-1.473360	-1.281026
C	-3.457459	2.813609	0.582096
C	2.811168	3.164739	0.015532
C	5.513692	-1.061561	-0.857436
C	-6.490975	-1.477735	-0.232785
H	-7.363624	-2.007006	-0.620658
H	-6.168744	-1.978915	0.681831
H	-6.801189	-0.466040	0.028561
C	-5.856699	-0.760092	-2.553026
H	-6.719986	-1.284555	-2.967442
H	-6.155435	0.267821	-2.347540
H	-5.074166	-0.738787	-3.313294
C	-5.034587	-2.925887	-1.640957
H	-4.253914	-2.988049	-2.400165
H	-4.708988	-3.495738	-0.769429
H	-5.926340	-3.411301	-2.039948
C	-2.193976	3.478256	1.133305
H	-1.813186	2.960465	2.014958
H	-1.402176	3.511863	0.387164
H	-2.419513	4.505301	1.423669
C	-3.927049	3.618099	-0.643612
H	-4.096319	4.660540	-0.366435
H	-3.174937	3.594122	-1.433961
H	-4.857725	3.224717	-1.052081
C	-4.545404	2.865003	1.670172
H	-4.723868	3.898538	1.973540
H	-5.490139	2.451926	1.317255
H	-4.239111	2.300415	2.552799
C	3.507473	3.375869	1.372418
H	3.002806	2.811275	2.158845
H	4.549935	3.059624	1.347365
H	3.485452	4.432748	1.646448
C	3.554615	3.969846	-1.065305
H	3.077341	3.843320	-2.038639
H	3.547087	5.033748	-0.818236
H	4.593684	3.653161	-1.156721
C	1.384609	3.706637	0.128995
H	0.838854	3.616218	-0.810651
H	0.822108	3.183671	0.901850
H	1.412139	4.764161	0.396303
C	5.515746	-2.228831	0.146714
H	5.396143	-1.861924	1.168055
H	4.709372	-2.932843	-0.057395
H	6.460547	-2.773951	0.091861
C	5.698014	-1.619959	-2.279739
H	5.704752	-0.812846	-3.014496
H	6.645908	-2.157188	-2.355619
H	4.897444	-2.309640	-2.545786
C	6.705035	-0.152190	-0.538885
H	7.630853	-0.724851	-0.613851
H	6.774327	0.683345	-1.236985
H	6.646916	0.252861	0.472628

E-TS3-tBu

Number of imaginary frequencies: 1
Electronic Energy (SCF) = -1801.12404343 a.u
ZPV corrected Energy = -1800.341487 a.u.
Enthalpy = -1800.301582 a.u.
Free Energy = -1800.410206 a.u.

S	-2.644591	-2.589241	0.222782
C	-4.215202	-1.671713	0.591510
H	-3.981224	-0.626450	0.773114
H	-4.594781	-2.107828	1.515211
C	-3.483815	-3.669956	-1.003822
H	-3.191349	-4.687736	-0.767566
H	-3.059665	-3.404940	-1.967518
C	-4.983439	-3.402941	-0.924431

H	-5.432196	-4.022563	-0.146168
H	-5.456764	-3.663301	-1.871267
C	-5.162741	-1.927065	-0.570645
H	-4.893071	-1.298000	-1.421207
H	-6.192680	-1.694670	-0.298928
C	-1.806229	-1.305352	-0.801825
H	-2.500688	-1.051519	-1.599215
C	-0.601619	-2.199433	-1.351108
H	-0.793635	-2.262727	-2.443885
O	-0.686623	-3.400228	-0.723533
C	0.743348	-1.485709	-1.219299
C	0.974100	-0.275919	-1.870608
C	1.741154	-2.036025	-0.428003
C	2.173212	0.412524	-1.709849
H	0.187139	0.139399	-2.483518
C	2.971363	-1.394766	-0.266226
H	1.517027	-2.972739	0.056564
C	3.158231	-0.170392	-0.906523
H	4.098205	0.347581	-0.779579
C	-1.518255	-0.085411	0.012280
C	-2.002946	1.148919	-0.412346
C	-0.728290	-0.159523	1.158168
C	-1.693836	2.320689	0.278146
H	-2.616031	1.180111	-1.300783
C	-0.389926	0.988390	1.867237
H	-0.353687	-1.121852	1.466840
C	-0.881777	2.212337	1.406566
H	-0.618771	3.111562	1.944496
C	0.527681	0.953602	3.092155
C	-2.203264	3.697781	-0.160121
C	2.430185	1.784420	-2.342634
C	4.111212	-1.997711	0.564319
C	-1.003989	4.604133	-0.491042
H	-1.353143	5.593638	-0.793333
H	-0.345304	4.728542	0.368241
H	-0.415502	4.186751	-1.308497
C	-3.014741	4.329035	0.985428
H	-3.386308	5.311346	0.686854
H	-3.872512	3.705264	1.243607
H	-2.410424	4.458045	1.883141
C	-3.102490	3.619027	-1.398340
H	-2.572519	3.211271	-2.260080
H	-3.985736	3.004263	-1.218282
H	-3.443497	4.620736	-1.663493
C	1.813399	1.735708	2.765243
H	1.596672	2.779770	2.538437
H	2.497992	1.710191	3.615623
H	2.321045	1.301937	1.904002
C	-0.176878	1.609463	4.292322
H	0.474314	1.585614	5.168416
H	-0.430146	2.650824	4.093957
H	-1.098106	1.078822	4.539967
C	0.913576	-0.476748	3.483569
H	0.037250	-1.073092	3.742917
H	1.445953	-0.986351	2.681453
H	1.569524	-0.451465	4.354868
C	4.576071	-0.996493	1.636274
H	5.400786	-1.421145	2.212706
H	4.924196	-0.062659	1.195812
H	3.766114	-0.761692	2.326630
C	3.691636	-3.290383	1.272580
H	2.855976	-3.123402	1.953549
H	3.400382	-4.065838	0.563454
H	4.529094	-3.672780	1.858642
C	5.292658	-2.317879	-0.369894
H	5.663224	-1.419536	-0.864438
H	6.117404	-2.756991	0.196034
H	4.993977	-3.028392	-1.142734
C	2.620875	2.820619	-1.219142
H	3.479950	2.576646	-0.594161
H	2.782941	3.814486	-1.642391
H	1.740007	2.861653	-0.578425
C	3.700786	1.735335	-3.208684
H	3.594848	1.004392	-4.012435
H	3.887978	2.712038	-3.660211
H	4.578847	1.465326	-2.622384
C	1.266082	2.243818	-3.227066
H	1.093676	1.555679	-4.056178
H	0.339681	2.333075	-2.660178
H	1.493211	3.224091	-3.649063

E-TS2-tBu

Number of imaginary frequencies: 1
Electronic Energy (SCF) = -1801.10403237 a.u
ZPV corrected Energy = -1800.323360 a.u.
Enthalpy = -1800.282645 a.u.
Free Energy = -1800.396220 a.u.

S	0.312830	-2.266609	1.291254
C	1.051079	-1.772779	2.895195
H	1.859612	-1.077656	2.686962
H	1.464478	-2.685054	3.322547
C	-1.403481	-2.323748	1.960632
H	-1.840614	-3.271540	1.661942
H	-1.945276	-1.513991	1.483252
C	-1.284226	-2.139320	3.473250
H	-1.113251	-3.103592	3.953338
H	-2.215369	-1.729603	3.862731
C	-0.094759	-1.216676	3.727272
H	-0.326624	-0.198572	3.412252
H	0.183836	-1.188965	4.780171
C	0.335871	-0.739109	0.275189
H	-0.152202	0.017976	0.883266
C	-0.533958	-1.038559	-1.097158
H	-0.741048	-2.144037	-1.040537
O	0.091807	-0.652213	-2.191052
C	-1.894075	-0.358394	-0.889330
C	-3.070022	-1.092481	-0.823772
C	-1.960610	1.029288	-0.787959
C	-4.310909	-0.473152	-0.640582
H	-3.009787	-2.170346	-0.910231
C	-3.170906	1.688424	-0.596681
H	-1.040238	1.592374	-0.866577
C	-4.337240	0.916149	-0.523786
H	-5.282330	1.410176	-0.377080
C	1.762633	-0.363322	0.047313
C	2.240112	0.833684	0.574220
C	2.614089	-1.183624	-0.690127
C	3.560815	1.233952	0.371545
H	1.558871	1.453339	1.138223
C	3.941791	-0.823675	-0.903126
H	2.218398	-2.092977	-1.110394
C	4.388073	0.386184	-0.364503
H	5.414508	0.679938	-0.531189
C	-5.579297	-1.330068	-0.563133
C	-3.187698	3.216675	-0.471950
C	4.117115	2.551484	0.921095
C	4.906636	-1.688549	-1.720501
C	-2.277378	3.644983	0.693864
H	-1.246909	3.327029	0.536461
H	-2.280356	4.732187	0.796434
H	-2.624350	3.214824	1.635553
C	-2.659066	3.836920	-1.777920
H	-3.289383	3.555159	-2.623136
H	-2.654062	4.926722	-1.705197
H	-1.642715	3.508139	-1.993773

C	-4.592084	3.771069	-0.209502
H	-5.279308	3.536148	-1.023347
H	-5.015520	3.380815	0.717409
H	-4.543409	4.857528	-0.120857
C	-6.843301	-0.492038	-0.343061
H	-6.800870	0.069039	0.591750
H	-7.007233	0.214742	-1.157578
H	-7.712039	-1.150551	-0.295452
C	-5.745676	-2.116040	-1.876150
H	-4.893772	-2.769247	-2.064148
H	-6.643445	-2.736634	-1.834767
H	-5.840812	-1.436149	-2.724525
C	-5.451670	-2.323102	0.606932
H	-6.350392	-2.939136	0.680467
H	-4.599584	-2.989721	0.475906
H	-5.324800	-1.793970	1.553457
C	5.297063	2.256036	1.864293
H	5.704585	3.187692	2.262112
H	4.976866	1.638952	2.705971
H	6.101854	1.731471	1.349405
C	4.608764	3.422274	-0.249258
H	5.005234	4.368479	0.124757
H	5.398644	2.926792	-0.813571
H	3.791291	3.643231	-0.937538
C	3.065153	3.346690	1.701923
H	2.211950	3.612516	1.076343
H	2.695926	2.791694	2.565820
H	3.509092	4.273213	2.068793
C	6.124470	-2.060023	-0.855942
H	5.817479	-2.627156	0.024909
H	6.820194	-2.675295	-1.430118
H	6.662810	-1.175277	-0.516127
C	5.380390	-0.891798	-2.949614
H	4.533544	-0.612944	-3.578585
H	5.899932	0.021593	-2.659571
H	6.066849	-1.494197	-3.548277
C	4.251359	-2.983938	-2.211156
H	3.912205	-3.605303	-1.380728
H	3.396921	-2.784066	-2.858631
H	4.976552	-3.562843	-2.785014

F-TS1-tBu
Number of imaginary frequencies: 1
Electronic Energy (SCF) = -1801.10953988 a.u
ZPV corrected Energy = -1800.328077 a.u.
Enthalpy = -1800.287584 a.u.
Free Energy = -1800.399031 a.u.

S	0.279972	-0.916748	1.383629
C	-0.911640	-1.628310	2.595357
H	-1.752771	-2.011153	2.022679
H	-1.251608	-0.819611	3.235917
C	1.458432	-2.319142	1.525779
H	2.310820	-1.934496	2.080776
H	1.772295	-2.582992	0.522629
C	0.718775	-3.420852	2.273951
H	1.434887	-4.110053	2.720112
H	0.095712	-3.985330	1.579214
C	-0.151964	-2.733518	3.321816
H	-0.857638	-3.420040	3.788465
H	0.472488	-2.306690	4.108217
C	-0.503332	-1.226665	-0.254242
H	-0.615652	-2.306353	-0.299473
C	0.461618	-0.889174	-1.542359
H	-0.098747	-0.055156	-2.024636
O	0.570468	-1.986944	-2.272573
C	-1.840117	-0.553630	-0.258804
C	-2.985290	-1.309964	-0.462170
C	-1.953992	0.830891	-0.086859
C	-4.251587	-0.713495	-0.508515
H	-2.884326	-2.378391	-0.591875
C	-3.192013	1.456523	-0.113559
H	-1.059443	1.411926	0.068459
C	-4.328615	0.662290	-0.328278
H	-5.293352	1.143151	-0.353871
C	1.791794	-0.253908	-1.085863
C	1.848038	1.046728	-0.593317
C	2.966221	-0.987757	-1.172370
C	3.043102	1.610773	-0.149429
H	0.937478	1.629552	-0.556948
C	4.186230	-0.466144	-0.740498
H	2.889796	-1.981431	-1.590838
C	4.201753	0.832742	-0.226056
H	5.134313	1.250805	0.112609
C	5.449911	-1.328303	-0.836428
C	3.041407	3.038139	0.409938
C	-5.485478	-1.589762	-0.743924
C	-3.351420	2.967586	0.079113
C	5.250080	-2.613342	-0.011858
H	4.401302	-3.192847	-0.374136
H	6.138967	-3.244880	-0.073426
H	5.075509	-2.376991	1.039598
C	5.696686	-1.707504	-2.307591
H	5.845207	-0.814792	-2.917767
H	6.589478	-2.330811	-2.393332
H	4.856546	-2.262678	-2.723952
C	6.696402	-0.609847	-0.309131
H	6.910890	0.297637	-0.875315
H	6.594060	-0.338625	0.742834
H	7.561459	-1.268846	-0.398786
C	4.433317	3.496159	0.858169
H	4.834318	2.860670	1.649204
H	5.144256	3.499669	0.030764
H	4.373244	4.513401	1.248340
C	2.098242	3.103117	1.625371
H	2.081999	4.113458	2.039587
H	1.077198	2.836385	1.354103
H	2.427814	2.418883	2.409587
C	2.537968	4.012547	-0.670009
H	1.526850	3.766234	-0.993331
H	2.526974	5.033705	-0.282934
H	3.186787	3.988064	-1.547232
C	-4.228305	3.233737	1.316123
H	-3.770786	2.814103	2.213996
H	-4.349442	4.308239	1.467285
H	-5.220964	2.797012	1.207629
C	-4.029186	3.572236	-1.163796
H	-4.145947	4.650759	-1.040622
H	-3.428606	3.394597	-2.057436
H	-5.017917	3.145384	-1.331598
C	-2.005607	3.671849	0.282800
H	-1.486882	3.306632	1.170153
H	-1.347288	3.541065	-0.576744
H	-2.171463	4.741977	0.413486
C	-5.586139	-2.637366	0.379944
H	-6.460191	-3.272878	0.224621
H	-4.706418	-3.280255	0.410071
H	-5.686361	-2.154020	1.353578
C	-6.784639	-0.777963	-0.762759
H	-6.955559	-0.263510	0.184053
H	-6.785188	-0.034286	-1.560857
H	-7.628176	-1.448335	-0.933186
C	-5.344827	-2.310111	-2.097067
H	-5.270648	-1.590085	-2.913708
H	-4.457745	-2.942592	-2.125762
H	-6.215778	-2.943019	-2.278552

F-TS3-tBu
Number of imaginary frequencies: 1
Electronic Energy (SCF) = -1801.12266779 a.u
ZPV corrected Energy = -1800.340361 a.u.
Enthalpy = -1800.300330 a.u.
Free Energy = -1800.409399 a.u.

S	-2.568384	-2.592335	0.058323
C	-4.144553	-1.689307	0.458312
H	-4.186407	-0.764471	-0.112686
H	-4.126399	-1.449933	1.518416
C	-3.402820	-3.673855	-1.169606
H	-3.318921	-4.676249	-0.754808
H	-2.819003	-3.631607	-2.081468
C	-4.848536	-3.210225	-1.301988
H	-5.482265	-4.039948	-1.614812
H	-4.919987	-2.430441	-2.062107
C	-5.260736	-2.644237	0.055607
H	-6.218661	-2.125876	0.014878
H	-5.351399	-3.451985	0.785363
C	-1.717762	-1.295075	-0.927475
H	-2.389131	-1.050068	-1.747835
C	-0.478590	-2.163496	-1.442882
H	-0.623696	-2.198053	-2.544533
O	-0.576592	-3.381748	-0.853183
C	-1.492194	-0.075147	-0.090042
C	-1.950319	1.159584	-0.542350
C	-0.793288	-0.152931	1.113254
C	-1.701912	2.327769	0.177336
H	-2.492589	1.194470	-1.475515
C	-0.530931	0.990017	1.862493
H	-0.435842	-1.115251	1.443387
C	-0.987684	2.214790	1.369834
H	-0.778480	3.111362	1.935014
C	0.849982	-1.439182	-1.238180
C	1.071045	-0.187324	-1.818485
C	1.844244	-2.013544	-0.468683
C	2.248954	0.511191	-1.596677
H	0.288111	0.246535	-2.422943
C	3.066527	-1.361358	-0.253353
H	1.638975	-2.983279	-0.039369
C	3.238616	-0.099842	-0.810505
H	4.160265	0.433582	-0.639363
C	4.159937	-2.056205	0.566121
C	2.486052	1.924286	-2.138924
C	0.269277	0.946439	3.167341
C	-2.197121	3.703316	-0.281830
C	3.630644	-2.383047	1.973195
H	3.371301	-1.470786	2.511371
H	2.744103	-3.015145	1.933108
H	4.394068	-2.910703	2.549227
C	4.555552	-3.366614	-0.138942
H	4.936118	-3.167562	-1.142514
H	5.336806	-3.880264	0.425775
H	3.705205	-4.042271	-0.229918
C	5.417092	-1.193532	0.719677
H	6.159166	-1.733460	1.310137
H	5.868441	-0.958250	-0.245224
H	5.202837	-0.255239	1.233740
C	3.776227	1.960113	-2.976252
H	3.947138	2.967111	-3.363050
H	4.648446	1.676948	-2.387429
H	3.707092	1.277620	-3.825365
C	1.329723	2.408317	-3.020488
H	1.195814	1.769747	-3.895172
H	0.388921	2.436508	-2.471492
H	1.538646	3.419029	-3.374649
C	2.625497	2.893234	-0.949606
H	2.774749	3.914629	-1.306913
H	1.728914	2.875379	-0.329892
H	3.474918	2.629561	-0.319332
C	-2.918422	3.640966	-1.632241
H	-3.803742	3.004889	-1.590937
H	-3.243474	4.642695	-1.916757
H	-2.265450	3.266932	-2.422089
C	-1.005003	4.666818	-0.419171
H	-1.355970	5.653555	-0.727952
H	-0.467590	4.782869	0.521581
H	-0.299202	4.307680	-1.168072
C	-3.178157	4.257420	0.767355
H	-3.546274	5.238404	0.459892
H	-4.036414	3.593333	0.885138
H	-2.701222	4.366638	1.741417
C	1.620530	1.648091	2.936668
H	2.181704	1.154731	2.142611
H	1.481469	2.691481	2.652216
H	2.220900	1.622794	3.848596
C	0.536307	-0.489067	3.631276
H	-0.393429	-1.031438	3.811675
H	1.120987	-1.048885	2.903752
H	1.099833	-0.471067	4.565289
C	-0.498751	1.674014	4.284317
H	0.069478	1.632217	5.215567
H	-0.669580	2.723700	4.047519
H	-1.468973	1.204915	4.458185

F-TS2-tBu
Number of imaginary frequencies: 1
Electronic Energy (SCF) = -1801.10361090 a.u
ZPV corrected Energy = -1800.322488 a.u.
Enthalpy = -1800.281954 a.u.
Free Energy = -1800.393830 a.u.

S	0.392435	-2.413438	1.247406
C	1.126908	-1.933775	2.870641
H	1.489907	-0.917113	2.745279
H	1.971338	-2.591225	3.056633
C	-1.303270	-2.553175	1.922010
H	-1.455895	-3.614478	2.111319
H	-1.997714	-2.220162	1.158204
C	-1.307322	-1.732751	3.202783
H	-2.171084	-1.997195	3.811857
H	-1.377402	-0.670746	2.964191
C	0.009896	-2.044236	3.909672
H	0.206962	-1.360117	4.734116
H	-0.019696	-3.056999	4.312935
C	0.347216	-0.848605	0.295854
H	-0.156201	-0.139101	0.946604
C	-0.534246	-1.119149	-1.074778
H	-0.770755	-2.218597	-1.019567
O	0.090764	-0.750726	-2.175492
C	1.755005	-0.407830	0.065147
C	2.178825	0.811736	0.586232
C	2.638018	-1.184631	-0.682376
C	3.474768	1.278186	0.365975
H	1.475006	1.397917	1.157932
C	3.944122	-0.760182	-0.909505
H	2.283787	-2.112472	-1.099116
C	4.335159	0.471848	-0.378501
H	5.343264	0.816851	-0.558336
C	-1.876729	-0.404666	-0.855921
C	-3.077665	-1.112054	-0.857738
C	-1.908673	0.974108	-0.692828
C	-4.301241	-0.467784	-0.680554
H	-3.038132	-2.182675	-1.002467
C	-3.109755	1.660812	-0.507282

H	-0.973043	1.516635	-0.718471
C	-4.291552	0.919828	-0.500739
H	-5.232122	1.427838	-0.356928
C	-3.087630	3.181956	-0.319265
C	-5.637505	-1.218962	-0.671458
C	4.941976	-1.578433	-1.735170
C	3.969409	2.624776	0.903982
C	-2.202973	3.535445	0.890364
H	-2.589969	3.075916	1.802060
H	-1.177024	3.196007	0.750048
H	-2.180170	4.617045	1.039022
C	-2.501133	3.839019	-1.581888
H	-3.111835	3.609371	-2.456735
H	-2.469151	4.924355	-1.463982
H	-1.487657	3.490987	-1.780386
C	-4.483848	3.765101	-0.077175
H	-4.408741	4.845455	0.056134
H	-5.151420	3.581178	-0.920024
H	-4.946047	3.350900	0.820354
C	-6.534284	-0.684128	-1.802600
H	-7.489832	-1.213009	-1.809829
H	-6.740093	0.379439	-1.682782
H	-6.058505	-0.827610	-2.774202
C	-5.460142	-2.727598	-0.874170
H	-4.989265	-2.954913	-1.831341
H	-4.856200	-3.174759	-0.083000
H	-6.436932	-3.213584	-0.860266
C	-6.341529	-0.996423	0.679531
H	-7.294947	-1.528582	0.701401
H	-5.727461	-1.366844	1.502761
H	-6.542999	0.059543	0.859087
C	2.886579	3.370237	1.691714
H	2.554497	2.802282	2.562226
H	3.286640	4.320113	2.049243
H	2.014963	3.589157	1.073444
C	4.404078	3.512899	-0.275872
H	4.756417	4.479827	0.089241
H	5.211875	3.054729	-0.846282
H	3.569363	3.688678	-0.956429
C	5.172705	2.394554	1.835684
H	5.538227	3.347957	2.222632
H	4.892607	1.768347	2.684878
H	5.996564	1.906477	1.315089
C	5.357210	-0.765152	-2.974542
H	4.488833	-0.534724	-3.593664
H	5.831579	0.175896	-2.695683
H	6.066778	-1.334032	-3.579042
C	4.349309	-2.909450	-2.209886
H	4.052775	-3.541964	-1.371555
H	3.478138	-2.758808	-2.848301
H	5.096871	-3.453731	-2.788913
C	6.188780	-1.880309	-0.884864
H	6.908340	-2.461780	-1.464861
H	6.684380	-0.966632	-0.556865
H	5.923299	-2.457491	0.002931

G-TS1-tBu
Number of imaginary frequencies: 1
Electronic Energy (SCF) = -1801.10425240 a.u
ZPV corrected Energy = -1800.323003 a.u.
Enthalpy = -1800.282556 a.u.
Free Energy = -1800.394468 a.u.

S	-0.247325	-2.619623	0.307369
C	-1.488094	-2.836456	1.641937
H	-1.458168	-1.955324	2.276767
H	-1.150032	-3.701739	2.209965
C	-1.328109	-3.313736	-1.001082
H	-0.737134	-4.028920	-1.565128
H	-1.609250	-2.489299	-1.647673
C	-2.527556	-3.932565	-0.282052
H	-2.289691	-4.953680	0.018082
H	-3.376802	-3.971997	-0.963226
C	-2.824455	-3.081597	0.951906
H	-3.269936	-2.132282	0.661584
H	-3.508618	-3.585424	1.633989
C	-0.136319	-0.813820	-0.055374
C	0.762439	-0.695807	-1.453167
O	0.200188	0.054021	-2.373550
H	0.472974	-0.486228	0.785320
C	-1.457122	-0.122691	0.003134
C	-1.833190	0.490378	1.199658
C	-2.339011	-0.111844	-1.078745
C	-3.086704	1.079448	1.350142
H	-1.126975	0.494512	2.016472
C	-3.601877	0.462520	-0.962622
H	-1.993124	-0.515810	-2.012413
C	-3.954648	1.040680	0.258289
H	-4.934097	1.486436	0.358245
H	0.912475	-1.767693	-1.753510
C	2.144232	-0.234517	-0.955682
C	3.054760	-1.142318	-0.411467
C	2.477177	1.107453	-1.014926
C	4.283770	-0.721278	0.085796
H	2.788834	-2.190079	-0.393839
C	3.701618	1.575104	-0.524060
H	1.757603	1.777646	-1.463725
C	4.583706	0.647022	0.022804
H	5.536084	0.982218	0.402494
C	4.024288	3.071049	-0.606186
C	5.310700	-1.688107	0.685970
C	-4.601687	0.477335	-2.123602
C	-3.535181	1.747820	2.653930
C	4.011772	3.512414	-2.080929
H	3.041200	3.335210	-2.543573
H	4.232991	4.579184	-2.158353
H	4.762840	2.966664	-2.655023
C	2.955614	3.865255	0.166873
H	2.940294	3.572635	1.218560
H	3.168536	4.935228	0.115251
H	1.959505	3.700746	-0.243113
C	5.396022	3.411445	-0.014077
H	5.458694	3.145764	1.042356
H	6.203344	2.902005	-0.542232
H	5.571666	4.485226	-0.096819
C	6.616162	-1.618981	-0.126921
H	7.358518	-2.303599	0.289114
H	6.439994	-1.899087	-1.166982
H	7.041613	-0.615622	-0.116550
C	5.601733	-1.286537	2.143443
H	6.003602	-0.275640	2.209474
H	4.692214	-1.327085	2.745859
H	6.332324	-1.967798	2.584896
C	4.818534	-3.139429	0.679887
H	3.905560	-3.259496	1.265254
H	4.623457	-3.496433	-0.332254
H	5.581529	-3.784544	1.118378
C	-4.927218	1.934244	-2.498045
H	-5.638967	1.961943	-3.325772
H	-5.365717	2.476136	-1.659929
H	-4.024773	2.464082	-2.807215
C	-4.054720	-0.229156	-3.368420
H	-3.146686	0.247208	-3.738829
H	-3.831229	-1.278911	-3.171684
H	-4.800230	-0.191825	-4.164162
C	-5.894548	-0.238542	-1.692927
H	-6.616127	-0.239021	-2.512426

H	-5.691036	-1.275892	-1.419909
H	-6.360988	0.249429	-0.837140
C	-4.791205	1.034702	3.186352
H	-5.123334	1.499416	4.116904
H	-5.614869	1.085225	2.474356
H	-4.583853	-0.018082	3.387537
C	-3.867292	3.225949	2.381748
H	-4.666956	3.329908	1.648510
H	-4.189784	3.716247	3.302573
H	-2.991934	3.755716	2.002286
C	-2.454974	1.690759	3.739139
H	-2.197253	0.663387	4.001386
H	-1.543060	2.203131	3.429707
H	-2.821241	2.180830	4.642299

G-TS3-tBu
Number of imaginary frequencies: 1
Electronic Energy (SCF) = -1801.12027844 a.u
ZPV corrected Energy = -1800.337678 a.u.
Enthalpy = -1800.297740 a.u.
Free Energy = -1800.406384 a.u.

S	-1.052432	3.290217	-1.476252
C	-2.720698	3.319690	-0.651179
H	-3.310502	2.470869	-0.986257
H	-3.190408	4.234819	-1.010146
C	-0.182577	3.717124	0.073917
H	0.556472	4.462466	-0.200947
H	0.337414	2.827491	0.407847
C	-1.229875	4.198287	1.072522
H	-1.447541	5.253749	0.899614
H	-0.845000	4.100459	2.087913
C	-2.493431	3.364666	0.854668
H	-2.347802	2.356445	1.237633
H	-3.353788	3.797069	1.366155
C	-0.876709	1.465855	-1.830206
C	0.627676	1.483535	-2.341121
O	1.030061	2.782354	-2.313562
H	-1.536380	1.379378	-2.694008
C	-1.388357	0.538537	-0.773905
C	-2.565987	-0.166084	-1.027768
C	-0.752176	0.368039	0.456507
C	-3.124027	-1.019451	-0.078022
H	-3.042768	-0.033534	-1.987528
C	-1.295177	-0.448831	1.443621
H	0.179088	0.872665	0.637331
C	-2.478302	-1.129960	1.153294
H	-2.911302	-1.767806	1.909668
H	0.559670	1.073818	-3.370517
C	1.527764	0.503294	-1.593257
C	1.324667	-0.872987	-1.717433
C	2.562664	0.966861	-0.803327
C	2.120033	-1.783343	-1.036428
H	0.518449	-1.217596	-2.349033
C	3.398110	0.083731	-0.106445
H	2.705158	2.036407	-0.751878
C	3.152423	-1.279450	-0.228905
H	3.777353	-1.979822	0.302769
C	4.552778	0.644082	0.731668
C	1.906321	-3.297975	-1.130488
C	-4.405620	-1.817913	-0.335756
C	-0.672077	-0.556263	2.839790
C	-4.974965	-1.569858	-1.736327
H	-5.237747	-0.521806	-1.887711
H	-4.271129	-1.861838	-2.516934
H	-5.881905	-2.161246	-1.869136
C	-4.101383	-3.320660	-0.197103
H	-3.741041	-3.566945	0.801661
H	-5.003733	-3.906556	-0.383085
H	-3.340385	-3.630073	-0.915593
C	-5.473724	-1.418044	0.697837
H	-6.393179	-1.981458	0.526979
H	-5.142445	-1.618520	1.716631
H	-5.707815	-0.354576	0.622232
C	0.735543	0.046526	2.887382
H	1.401239	-0.438313	2.173934
H	0.729607	1.115281	2.672918
H	1.155377	-0.084961	3.885663
C	-0.576391	-2.023715	3.287508
H	-0.135663	-2.077981	4.284661
H	-1.553042	-2.504223	3.333206
H	0.053093	-2.598162	2.608160
C	-1.571347	0.213053	3.825761
H	-2.579344	-0.202740	3.849376
H	-1.158766	0.161077	4.835577
H	-1.647279	1.264663	3.543628
C	5.538773	1.367534	-0.203935
H	6.373528	1.778303	0.368382
H	5.942366	0.679320	-0.948819
H	5.054708	2.188616	-0.732748
C	4.013244	1.649815	1.764321
H	3.475071	2.468383	1.287121
H	3.335385	1.162152	2.465132
H	4.838646	2.080144	2.335400
C	5.315870	-0.449368	1.486597
H	4.665459	-0.995795	2.171535
H	5.774870	-1.167944	0.806301
H	6.114113	0.004297	2.076314
C	1.568499	-3.851993	0.264647
H	0.639413	-3.416718	0.633098
H	1.442245	-4.936048	0.221985
H	2.354451	-3.632487	0.987244
C	3.190947	-3.970021	-1.646746
H	3.457753	-3.589860	-2.634506
H	4.034263	-3.792574	-0.979388
H	3.047361	-5.049812	-1.725074
C	0.757101	-3.665682	-2.075582
H	-0.190889	-3.241807	-1.741336
H	0.944466	-3.322607	-3.094109
H	0.643729	-4.750567	-2.105432

G-TS2-tBu
Number of imaginary frequencies: 1
Electronic Energy (SCF) = -1801.10741194 a.u
ZPV corrected Energy = -1800.325838 a.u.
Enthalpy = -1800.285389 a.u.
Free Energy = -1800.396931 a.u.

S	-0.388493	-1.331777	1.109115
C	0.945061	-1.932562	2.221126
H	1.621227	-2.551511	1.638632
H	0.441829	-2.552184	2.961493
C	-0.399140	0.356810	1.823351
H	-1.436062	0.624852	1.991828
H	0.028180	1.023192	1.083243
C	0.444182	0.289421	3.095961
H	-0.170367	-0.055469	3.928360
H	0.811766	1.285604	3.340261
C	1.582209	-0.696412	2.842164
H	2.310070	-0.268923	2.154865
H	2.099719	-0.965126	3.762604
C	0.397861	-1.243813	-0.594919
C	-0.604978	-0.707789	-1.758300
O	-0.626931	-1.616973	-2.725292
H	0.460599	-2.311511	-0.798208
C	1.760351	-0.636122	-0.544688

C	2.882193	-1.462178	-0.470680
C	1.932214	0.748989	-0.544946
C	4.165333	-0.930293	-0.363744
H	2.732408	-2.530463	-0.497645
C	3.196587	1.317868	-0.423439
H	1.065804	1.380257	-0.647605
C	4.292758	0.459040	-0.327458
H	5.280499	0.886236	-0.231932
H	-0.144626	0.262948	-2.045751
C	-1.973446	-0.332434	-1.168075
C	-2.263982	0.981503	-0.821004
C	-2.944760	-1.309533	-0.979649
C	-3.497783	1.338940	-0.273328
H	-1.507263	1.738375	-0.988307
C	-4.182972	-1.003897	-0.419579
H	-2.705006	-2.318169	-1.286621
C	-4.438865	0.327585	-0.072931
H	-5.395104	0.581488	0.351392
C	-3.775677	2.804810	0.079830
C	-5.207658	-2.121598	-0.194868
C	3.411767	2.833647	-0.376859
C	5.417737	-1.807500	-0.272155
C	-2.733862	3.293829	1.102364
H	-1.720250	3.219417	0.709454
H	-2.917332	4.339738	1.357015
H	-2.785295	2.708058	2.021940
C	-3.670253	3.662868	-1.193876
H	-4.397006	3.338424	-1.940533
H	-3.865953	4.711826	-0.961139
H	-2.678235	3.596940	-1.640443
C	-5.169325	3.010305	0.683038
H	-5.958037	2.715094	-0.010163
H	-5.298896	2.444986	1.607224
H	-5.312055	4.066389	0.917234
C	2.097262	3.611753	-0.500984
H	1.587753	3.400056	-1.441858
H	1.412841	3.383755	0.317738
H	2.304676	4.682113	-0.469000
C	4.068083	3.207133	0.964808
H	4.226276	4.285993	1.019654
H	3.432502	2.913959	1.802696
H	5.034986	2.719762	1.089141
C	4.336302	3.256765	-1.531825
H	4.500706	4.335802	-1.506977
H	5.308172	2.767709	-1.467759
H	3.893170	3.001769	-2.495863
C	6.135051	-1.527009	1.060787
H	7.031213	-2.145090	1.143497
H	6.438988	-0.483439	1.142066
H	5.485042	-1.756230	1.907357
C	6.363140	-1.470903	-1.439141
H	6.673013	-0.426211	-1.416980
H	7.261425	-2.089182	-1.386042
H	5.876946	-1.658344	-2.397913
C	5.088021	-3.302407	-0.336588
H	4.444910	-3.612068	0.488614
H	4.594333	-3.567823	-1.272336
H	6.010820	-3.880419	-0.271727
C	-4.602719	-3.176732	0.749410
H	-5.316591	-3.985062	0.922082
H	-3.695045	-3.612548	0.332326
H	-4.350786	-2.733802	1.715062
C	-5.545222	-2.784291	-1.542278
H	-4.658799	-3.207198	-2.014322
H	-6.267533	-3.590546	-1.396978
H	-5.979480	-2.058894	-2.232537
C	-6.510874	-1.611109	0.429172
H	-6.342157	-1.155110	1.405997
H	-7.005106	-0.876922	-0.208703
H	-7.199131	-2.446367	0.567892

H-TS1-tBu

Number of imaginary frequencies: 1
Electronic Energy (SCF) = -1801.10373266 a.u
ZPV corrected Energy = -1800.322823 a.u.
Enthalpy = -1800.282267 a.u.
Free Energy = -1800.395025 a.u.

S	-0.163997	-2.614201	0.441868
C	-1.293903	-2.824749	1.873304
H	-1.770802	-1.863025	2.048071
H	-0.687989	-3.098429	2.732655
C	-1.296421	-3.431636	-0.748748
H	-0.819439	-4.380149	-0.986020
H	-1.343914	-2.825332	-1.647164
C	-2.624948	-3.619512	-0.023520
H	-3.180385	-4.440000	-0.476235
H	-3.222034	-2.713521	-0.108824
C	-2.297407	-3.891669	1.443509
H	-3.182645	-3.840321	2.076484
H	-1.859989	-4.884748	1.556320
C	-0.135302	-0.831116	-0.004362
C	0.744062	-0.749564	-1.427218
O	0.153000	-0.047975	-2.364856
H	0.476547	-0.437991	0.806084
C	-1.469123	-0.167882	0.026464
C	-1.816729	0.581814	1.151609
C	-2.374284	-0.278432	-1.029186
C	-3.058116	1.208152	1.251265
H	-1.094925	0.670705	1.949977
C	-3.624869	0.329510	-0.962766
H	-2.061578	-0.805515	-1.911130
C	-3.943297	1.061067	0.183603
H	-4.909281	1.542015	0.241103
H	0.909826	-1.830000	-1.685188
C	2.119084	-0.240740	-0.963027
C	2.396913	1.118940	-1.015506
C	3.076417	-1.109293	-0.451581
C	3.608144	1.629400	-0.549345
H	1.640869	1.763686	-1.441239
C	4.302422	-0.642756	0.026243
H	2.859733	-2.170328	-0.434217
C	4.545358	0.731697	-0.028335
H	5.486150	1.109912	0.333250
C	5.326302	-1.638739	0.583301
C	3.870891	3.137983	-0.624163
C	-4.640250	0.236923	-2.106784
C	-3.472356	2.032516	2.474695
C	5.237341	3.528087	-0.050379
H	6.056035	3.056025	-0.595422
H	5.368517	4.608689	-0.126364
H	5.327727	3.256380	1.002519
C	3.818565	3.589268	-2.095083
H	2.848433	3.377601	-2.543995
H	3.997353	4.664330	-2.167855
H	4.581046	3.077035	-2.684794
C	2.784377	3.884865	0.170868
H	2.795161	3.583271	1.220085
H	2.955971	4.962539	0.125707
H	1.789438	3.686077	-0.226519
C	6.602401	-0.953573	1.083807
H	7.115873	-0.418948	0.283525
H	6.393639	-0.245711	1.887339
H	7.289876	-1.705683	1.474188
C	5.717585	-2.639085	-0.519470
H	6.445920	-3.357844	-0.137577
H	4.852977	-3.196086	-0.880329

H	6.163927	-2.122325	-1.370873
C	4.700652	-2.407734	1.761525
H	3.810652	-2.956948	1.454080
H	5.415736	-3.126511	2.167460
H	4.414871	-1.723769	2.562951
C	-3.793901	3.472463	2.036038
H	-4.611058	3.500189	1.315383
H	-4.087265	4.073052	2.899470
H	-2.922526	3.940852	1.575396
C	-4.725348	1.404776	3.111600
H	-4.525037	0.381638	3.435500
H	-5.035307	1.982566	3.984741
H	-5.561544	1.378807	2.412869
C	-2.370576	2.089600	3.538173
H	-2.119098	1.097006	3.915213
H	-1.459631	2.549378	3.152720
H	-2.712577	2.687993	4.383768
C	-4.141130	-0.646405	-3.255228
H	-3.222121	-0.258307	-3.695060
H	-3.954935	-1.669919	-2.926150
H	-4.897540	-0.682505	-4.040667
C	-4.905075	1.648291	-2.661184
H	-5.307509	2.310392	-1.894173
H	-3.983894	2.092756	-3.041315
H	-5.625950	1.604220	-3.480303
C	-5.959366	-0.356506	-1.580933
H	-6.689438	-0.425601	-2.389885
H	-5.803305	-1.359750	-1.179841
H	-6.393286	0.257403	-0.791874

H-TS3-tBu
Number of imaginary frequencies: 1
Electronic Energy (SCF) = -1801.11972058 a.u
ZPV corrected Energy = -1800.336874 a.u.
Enthalpy = -1800.297021 a.u.
Free Energy = -1800.405044 a.u.

S	-0.765109	3.349702	-1.504026
C	-2.311461	3.622060	-0.523603
H	-2.659156	2.684239	-0.097545
H	-3.065195	4.013487	-1.203437
C	0.317402	3.709682	-0.066109
H	0.924208	4.545841	-0.405967
H	0.972753	2.862853	0.089190
C	-0.573179	4.064796	1.121439
H	-0.072924	4.790801	1.761906
H	-0.768389	3.171821	1.714204
C	-1.892479	4.602539	0.564882
H	-2.660180	4.678549	1.334673
H	-1.746314	5.599217	0.142733
C	-0.853691	1.527012	-1.851843
C	0.611448	1.332511	-2.444329
O	1.144759	2.574455	-2.571343
H	-1.557541	1.529856	-2.685265
C	-1.411589	0.654256	-0.775220
C	-2.560670	-0.088192	-1.044265
C	-0.819433	0.554392	0.482868
C	-3.123730	-0.927766	-0.083378
H	-3.007865	-0.001879	-2.023414
C	-1.374247	-0.238011	1.481938
H	0.086443	1.098461	0.676894
C	-2.521729	-0.971681	1.173793
H	-2.961485	-1.595129	1.938262
H	0.434448	0.832266	-3.418618
C	1.442207	0.334340	-1.638060
C	2.529755	0.763228	-0.890738
C	1.125880	-1.021464	-1.669490
C	3.298665	-0.138842	-0.151487
H	2.762582	1.815740	-0.922151
C	1.859839	-1.954206	-0.943038
H	0.284858	-1.338051	-2.269287
C	2.937954	-1.486305	-0.185062
H	3.524232	-2.197691	0.379243
C	1.533316	-3.452036	-0.957817
C	4.533223	0.295602	0.648886
C	-4.366541	-1.780707	-0.357333
C	-0.795044	-0.277463	2.900031
C	-1.769275	0.456912	3.839716
H	-2.748930	-0.022370	3.847093
H	-1.383912	0.457041	4.861510
H	-1.904257	1.493945	3.527073
C	-0.627698	-1.727980	3.381193
H	-0.240152	-1.736705	4.401657
H	-1.570938	-2.273243	3.381577
H	0.076156	-2.267204	2.747794
C	0.575616	0.403093	2.978260
H	1.285785	-0.061266	2.295109
H	0.521270	1.466456	2.742792
H	0.971618	0.310529	3.990488
C	-5.487103	-1.382046	0.619600
H	-5.754391	-0.331046	0.495487
H	-6.379375	-1.983660	0.435284
H	-5.189098	-1.533948	1.656933
C	-4.889252	-1.606579	-1.786932
H	-5.184360	-0.575742	-1.988366
H	-4.144602	-1.900058	-2.528136
H	-5.767636	-2.237078	-1.931317
C	-4.016159	-3.265369	-0.149297
H	-3.221028	-3.575530	-0.829242
H	-3.682264	-3.460373	0.869746
H	-4.891250	-3.889265	-0.341915
C	1.186747	-3.915747	0.467272
H	2.000759	-3.717922	1.164630
H	0.296804	-3.401757	0.830599
H	0.986866	-4.989501	0.478026
C	0.341163	-3.778636	-1.864063
H	0.533383	-3.503224	-2.902011
H	0.145852	-4.851841	-1.835351
H	-0.564799	-3.267008	-1.537177
C	2.755338	-4.238660	-1.464728
H	2.532983	-5.307622	-1.490061
H	3.028260	-3.923946	-2.473594
H	3.622702	-4.092405	-0.821053
C	4.400045	-0.145492	2.116880
H	3.546309	0.337793	2.592767
H	4.269681	-1.223618	2.207423
H	5.298198	0.130163	2.673681
C	5.781873	-0.365947	0.036689
H	5.902308	-0.073185	-1.007769
H	6.678733	-0.062591	0.581419
H	5.715487	-1.453467	0.075321
C	4.733640	1.814959	0.627336
H	4.890087	2.190359	-0.384376
H	3.878317	2.339881	1.055620
H	5.613641	2.074645	1.218143

H-TS2-tBu
Number of imaginary frequencies: 1
Electronic Energy (SCF) = -1801.10714286 a.u
ZPV corrected Energy = -1800.325834 a.u.
Enthalpy = -1800.285337 a.u.
Free Energy = -1800.397409 a.u.

S	-0.430850	-1.265686	1.151044
C	0.733722	-2.215536	2.208811
H	1.606029	-2.446287	1.603479

H	0.233384	-3.136785	2.494502
C	-0.057851	0.336597	1.957347
H	-0.940028	0.561588	2.551850
H	0.049523	1.092412	1.189059
C	1.181804	0.115493	2.815615
H	1.224477	0.866950	3.603493
H	2.077300	0.212360	2.203468
C	1.078280	-1.299519	3.379814
H	2.009264	-1.626553	3.841819
H	0.292535	-1.347149	4.134988
C	0.369379	-1.271391	-0.546132
C	-0.636620	-0.796697	-1.732123
O	-0.695173	-1.770970	-2.631340
H	0.432527	-2.348476	-0.695991
C	1.729765	-0.656795	-0.523361
C	2.858715	-1.469604	-0.415457
C	1.890728	0.727503	-0.601551
C	4.139630	-0.923390	-0.356009
H	2.717669	-2.539147	-0.385983
C	3.152741	1.310150	-0.541055
H	1.016548	1.345767	-0.716901
C	4.257119	0.465645	-0.410639
H	5.243148	0.904398	-0.361394
H	-0.160367	0.140555	-2.095740
C	-1.984283	-0.346591	-1.140082
C	-3.046046	-1.239123	-1.080332
C	-2.159293	0.944962	-0.653947
C	-4.267371	-0.877311	-0.514201
H	-2.885559	-2.224465	-1.495044
C	-3.370129	1.358468	-0.095385
H	-1.332944	1.641577	-0.727684
C	-4.407573	0.425770	-0.025922
H	-5.348589	0.723939	0.403509
C	-3.527989	2.802619	0.395648
C	-5.402656	-1.905051	-0.446259
C	3.359491	2.826501	-0.608028
C	5.399585	-1.785460	-0.229587
C	-4.914164	3.073269	0.990578
H	-4.968884	4.109613	1.327579
H	-5.706787	2.921001	0.256938
H	-5.118277	2.433338	1.850412
C	-3.314975	3.764936	-0.787168
H	-3.427427	4.800125	-0.457726
H	-2.320030	3.655701	-1.218539
H	-4.045355	3.577281	-1.576000
C	-2.476243	3.100006	1.479055
H	-2.573483	4.130614	1.826604
H	-2.605787	2.439378	2.338059
H	-1.462633	2.968843	1.102040
C	-6.664051	-1.348729	0.222903
H	-6.473823	-1.036084	1.250909
H	-7.070171	-0.495947	-0.322908
H	-7.433214	-2.122246	0.248593
C	-5.769610	-2.351980	-1.872676
H	-4.917284	-2.799260	-2.383465
H	-6.572238	-3.092162	-1.842828
H	-6.111260	-1.503667	-2.468477
C	-4.930361	-3.128952	0.359813
H	-5.726362	-3.874449	0.418230
H	-4.061906	-3.599404	-0.100605
H	-4.658576	-2.841856	1.377590
C	2.037311	3.587645	-0.753013
H	1.504920	3.306980	-1.662575
H	1.376092	3.416176	0.097638
H	2.238414	4.658514	-0.802966
C	4.051283	3.301800	0.682223
H	4.203512	4.382463	0.651713
H	3.442512	3.069410	1.558055
H	5.025075	2.830240	0.813902
C	4.248401	3.167549	-1.817202
H	4.404223	4.246477	-1.877410
H	5.225923	2.691099	-1.744031
H	3.781074	2.838283	-2.746677
C	6.143792	-1.409109	1.064408
H	5.514778	-1.584508	1.939240
H	7.046743	-2.013852	1.168811
H	6.440765	-0.360439	1.067593
C	6.317419	-1.526377	-1.437682
H	6.620401	-0.480958	-1.494872
H	7.220847	-2.134715	-1.361689
H	5.811770	-1.782486	-2.370142
C	5.078241	-3.282769	-0.180907
H	4.450493	-3.534787	0.675253
H	4.571275	-3.617964	-1.086642
H	6.005590	-3.849895	-0.089986

A-TS4-tBu

Number of imaginary frequencies: 1
Electronic Energy (SCF) = -1801.10751943 a.u
ZPV corrected Energy = -1800.326869 a.u.
Enthalpy = -1800.285619 a.u.
Free Energy = -1800.399719 a.u.

S	0.796179	2.885180	0.397039
C	2.535808	2.975299	0.966355
H	2.956063	1.972957	0.930853
H	2.520998	3.315494	2.000147
C	0.899367	4.426852	-0.596843
H	0.025455	5.035803	-0.380694
H	0.878323	4.138277	-1.646087
C	2.219873	5.093422	-0.213533
H	2.084889	5.685390	0.693199
H	2.546051	5.764417	-1.008192
C	3.226379	3.974342	0.045101
H	3.495692	3.487313	-0.893597
H	4.142182	4.345360	0.505323
C	0.995991	1.376947	-1.298947
H	1.741928	1.967755	-1.812202
C	-0.283235	1.332163	-2.093080
H	-0.572918	2.367176	-2.363717
O	0.288024	0.617685	-3.100409
C	-1.496925	0.706490	-1.419748
C	-2.268894	1.432330	-0.524846
C	-1.838274	-0.613282	-1.704818
C	-3.363890	0.855477	0.125984
H	-2.011342	2.464416	-0.324614
C	-2.934630	-1.218137	-1.098724
H	-1.212945	-1.137615	-2.409289
C	-3.677650	-0.466238	-0.179394
H	-4.525113	-0.929546	0.300461
C	1.539532	0.218759	-0.565666
C	2.858063	-0.158760	-0.793841
C	0.794087	-0.450344	0.412860
C	3.444423	-1.216846	-0.093656
H	3.425598	0.379159	-1.540474
C	1.346293	-1.496091	1.137039
H	-0.213189	-0.127374	0.606958
C	2.669527	-1.869139	0.859143
H	3.099542	-2.686879	1.415292
C	4.898363	-1.599904	-0.385236
C	0.560460	-2.247001	2.215906
C	-3.359577	-2.657611	-1.411076
C	-4.160759	1.681519	1.141163
C	5.379449	-2.769049	0.480147
H	6.416974	-2.999144	0.233918
H	5.335957	-2.531344	1.544072
H	4.789735	-3.670245	0.306779

C	5.805435	-0.387361	-0.106638
H	5.730463	-0.078610	0.937734
H	6.847323	-0.640740	-0.312147
H	5.539241	0.465743	-0.730717
C	5.031763	-2.004935	-1.864138
H	4.396074	-2.862688	-2.089942
H	4.747264	-1.191811	-2.531745
H	6.065012	-2.277907	-2.087853
C	1.326071	-2.189488	3.549782
H	0.768699	-2.719301	4.324813
H	2.310552	-2.650638	3.473231
H	1.460992	-1.156387	3.875584
C	0.400617	-3.716371	1.785682
H	1.368071	-4.205775	1.670141
H	-0.170019	-4.269856	2.534208
H	-0.127843	-3.786148	0.834338
C	-0.832283	-1.647424	2.437148
H	-0.773967	-0.609882	2.769286
H	-1.438589	-1.678974	1.533446
H	-1.354657	-2.215501	3.208291
C	-2.397125	-3.346642	-2.384260
H	-2.733496	-4.368660	-2.566436
H	-2.356513	-2.833262	-3.345537
H	-1.383828	-3.394365	-1.983504
C	-3.404229	-3.486588	-0.115230
H	-2.420185	-3.531081	0.351572
H	-4.102579	-3.069592	0.610190
H	-3.721418	-4.508242	-0.334892
C	-4.761974	-2.640767	-2.046626
H	-5.081661	-3.657206	-2.286710
H	-5.500974	-2.206095	-1.373053
H	-4.762339	-2.057306	-2.969050
C	-5.324157	0.894844	1.753827
H	-4.976689	0.008557	2.286657
H	-5.853149	1.526246	2.469407
H	-6.042290	0.577843	0.996262
C	-3.223000	2.118554	2.281411
H	-2.803022	1.249684	2.791133
H	-2.394866	2.722103	1.910565
H	-3.770865	2.712472	3.016145
C	-4.734725	2.931456	0.450460
H	-3.946635	3.560252	0.036154
H	-5.404304	2.650874	-0.364516
H	-5.301765	3.531669	1.165209

B-TS4-tBu
Number of imaginary frequencies: 1
Electronic Energy (SCF) = -1801.10624568 a.u
ZPV corrected Energy = -1800.325837 a.u.
Enthalpy = -1800.284482 a.u.
Free Energy = -1800.399379 a.u.

S	0.937796	2.853456	0.252764
C	2.674402	2.872082	0.844806
H	3.192424	2.041838	0.367905
H	2.669415	2.709324	1.919085
C	1.138439	4.340480	-0.802734
H	0.645184	5.168746	-0.297487
H	0.636409	4.154294	-1.749019
C	2.642025	4.562722	-0.930713
H	2.849896	5.590165	-1.229226
H	3.049241	3.900973	-1.696826
C	3.249765	4.223691	0.428405
H	4.337931	4.171706	0.389989
H	2.974557	4.986215	1.158804
C	1.028115	1.266426	-1.366744
H	1.780932	1.800220	-1.930293
C	-0.268917	1.214754	-2.132429
H	-0.537090	2.241618	-2.451689
O	0.262676	0.436901	-3.113687
C	-1.491989	0.657432	-1.414140
C	-2.189427	1.420101	-0.487983
C	-1.934312	-0.628773	-1.711668
C	-3.308117	0.911725	0.178036
H	-1.854543	2.426566	-0.274654
C	-3.066065	-1.160056	-1.099470
H	-1.363711	-1.183373	-2.438925
C	-3.730761	-0.374700	-0.149777
H	-4.605731	-0.779336	0.333421
C	1.552072	0.127726	-0.586498
C	2.847623	-0.321340	-0.838304
C	0.823629	-0.445419	0.455368
C	3.418186	-1.349088	-0.089510
H	3.402913	0.140315	-1.642999
C	1.367429	-1.457897	1.239818
H	-0.167013	-0.076427	0.665193
C	2.660250	-1.899495	0.945342
H	3.089962	-2.685205	1.542506
C	4.840134	-1.820597	-0.410900
C	0.542354	-2.030378	2.396621
C	-3.626365	-2.540452	-1.462908
C	-4.011064	1.767793	1.237069
C	5.814698	-0.639044	-0.253533
H	6.834405	-0.959163	-0.476623
H	5.566634	0.179933	-0.928670
H	5.795363	-0.253441	0.767529
C	4.890910	-2.330567	-1.862308
H	4.613864	-1.551640	-2.572387
H	5.900196	-2.666980	-2.108078
H	4.208159	-3.170314	-2.002106
C	5.304060	-2.951743	0.512890
H	5.322820	-2.640625	1.558412
H	4.663760	-3.830923	0.429155
H	6.316451	-3.251077	0.238085
C	0.208038	-0.898122	3.385138
H	-0.386371	-1.285649	4.215013
H	1.118995	-0.458315	3.795632
H	-0.364034	-0.104384	2.905411
C	1.283590	-3.133934	3.157424
H	2.210624	-2.769159	3.602446
H	0.651159	-3.504351	3.965492
H	1.524698	-3.979178	2.511138
C	-0.766279	-2.615425	1.839789
H	-1.357032	-1.858552	1.327251
H	-0.559716	-3.417068	1.129192
H	-1.372764	-3.025382	2.649951
C	-3.857882	-3.383188	-0.196801
H	-4.281248	-4.352503	-0.467932
H	-2.921271	-3.559543	0.331173
H	-4.549533	-2.901775	0.493970
C	-4.972117	-2.347178	-2.186972
H	-5.695162	-1.836800	-1.549672
H	-4.843114	-1.753037	-3.093335
H	-5.393884	-3.314130	-2.470421
C	-2.685155	-3.321112	-2.386745
H	-3.111095	-4.304163	-2.594354
H	-2.539717	-2.814939	-3.341410
H	-1.705928	-3.468945	-1.929163
C	-5.243745	1.075780	1.828327
H	-4.984344	0.139477	2.324289
H	-5.704748	1.727742	2.571948
H	-5.992435	0.861238	1.064327
C	-3.022822	2.053320	2.383038
H	-2.696135	1.123606	2.852058
H	-2.136841	2.578097	2.025811
H	-3.496759	2.671810	3.148355
C	-4.461209	3.100632	0.613068

H	-3.617315	3.668664	0.221921
H	-5.160259	2.928549	-0.207286
H	-4.961312	3.717840	1.362356

C-TS4-tBu

Number of imaginary frequencies: 1
Electronic Energy (SCF) = -1801.10760906 a.u
ZPV corrected Energy = -1800.326668 a.u.
Enthalpy = -1800.285583 a.u.
Free Energy = -1800.399483 a.u.

S	0.604312	-2.972041	-0.425011
C	2.351949	-3.374278	-0.028045
H	2.983302	-2.993838	-0.826852
H	2.426451	-4.459797	-0.006302
C	0.218468	-2.365859	1.257155
H	-0.755145	-2.751241	1.537005
H	0.176039	-1.280871	1.217666
C	1.354442	-2.857169	2.150212
H	1.176760	-3.894515	2.438397
H	1.397279	-2.258175	3.059887
C	2.639621	-2.749607	1.334452
H	2.912977	-1.703441	1.207921
H	3.473253	-3.262405	1.813894
C	0.810481	-1.185997	-1.746749
C	-0.528908	-0.870922	-2.386561
O	-0.055448	0.041064	-3.267457
H	1.411697	-1.745003	-2.454077
C	1.613578	-0.237478	-0.957284
C	2.999759	-0.250894	-1.127872
C	1.060786	0.604434	0.009678
C	3.839380	0.534598	-0.344029
H	3.416893	-0.896670	-1.887831
C	1.868164	1.400064	0.817675
H	-0.009317	0.634203	0.123396
C	3.251488	1.344806	0.628963
H	3.885325	1.952001	1.251892
H	-0.879485	-1.819090	-2.848962
C	-1.683300	-0.419489	-1.489735
C	-2.451457	-1.344012	-0.794569
C	-2.030362	0.929819	-1.439225
C	-3.546081	-0.948186	-0.017338
H	-2.217629	-2.397065	-0.890507
C	-3.116322	1.365567	-0.687208
H	-1.429423	1.609775	-2.021828
C	-3.849017	0.409788	0.029019
H	-4.694559	0.740803	0.610787
C	-4.410246	-2.003758	0.683693
C	-3.558241	2.832735	-0.640678
C	1.221345	2.323613	1.855339
C	5.354832	0.471498	-0.560453
C	-3.537294	-2.904899	1.572731
H	-4.158593	-3.645316	2.080488
H	-2.791563	-3.444359	0.989823
H	-3.019123	-2.318879	2.333708
C	-5.094604	-2.872393	-0.388223
H	-5.717518	-3.636014	0.082789
H	-5.730800	-2.263327	-1.032595
H	-4.361629	-3.376033	-1.018779
C	-5.498091	-1.379416	1.564780
H	-5.072003	-0.743395	2.342438
H	-6.200847	-0.782344	0.982709
H	-6.066025	-2.171181	2.055622
C	-5.001803	2.944387	-1.165706
H	-5.697508	2.364802	-0.559080
H	-5.329417	3.986080	-1.149383
H	-5.068779	2.583255	-2.193460
C	-2.669672	3.735373	-1.502990
H	-1.630801	3.718801	-1.175055
H	-2.696889	3.441367	-2.552909
H	-3.023804	4.765365	-1.435468
C	-3.511070	3.341265	0.811382
H	-3.827283	4.385637	0.856191
H	-4.170659	2.764007	1.459506
H	-2.500439	3.276120	1.214737
C	5.679130	0.883585	-2.007346
H	6.756865	0.843334	-2.177595
H	5.200082	0.223717	-2.730500
H	5.340617	1.901850	-2.205683
C	6.120279	1.399466	0.388409
H	7.189982	1.316188	0.191559
H	5.837511	2.443747	0.248921
H	5.954353	1.137434	1.434410
C	5.841478	-0.969661	-0.322588
H	5.628540	-1.288781	0.698941
H	5.362706	-1.672456	-1.004236
H	6.919984	-1.033124	-0.479885
C	0.499095	3.456933	1.106473
H	-0.260789	3.058723	0.437202
H	0.012284	4.132721	1.812465
H	1.203745	4.037060	0.508241
C	2.248244	2.942919	2.809602
H	2.811142	2.178430	3.348112
H	2.957699	3.584323	2.285938
H	1.731524	3.558960	3.546854
C	0.197565	1.541742	2.697522
H	0.678839	0.721153	3.232811
H	-0.259088	2.203752	3.435426
H	-0.603836	1.126419	2.088165

D-TS4-tBu

Number of imaginary frequencies: 1
Electronic Energy (SCF) = -1801.10945005 a.u
ZPV corrected Energy = -1800.328009 a.u.
Enthalpy = -1800.287261 a.u.
Free Energy = -1800.397710 a.u.

S	-0.751597	2.938772	-0.668070
C	-2.511703	3.403750	-0.461399
H	-3.117733	2.633885	-0.933207
H	-2.672882	4.349386	-0.972559
C	-0.544415	2.402143	1.071934
H	0.076236	3.154819	1.553943
H	-0.018105	1.453338	1.077841
C	-1.944701	2.334958	1.672464
H	-1.890811	2.425866	2.757237
H	-2.399094	1.374119	1.435951
C	-2.753787	3.467925	1.044854
H	-3.819186	3.375777	1.255990
H	-2.415211	4.429854	1.432696
C	-0.855938	1.073054	-1.881576
C	0.496884	0.807384	-2.520276
O	0.079441	-0.164598	-3.362773
H	-1.504309	1.543339	-2.611436
C	-1.571343	0.120035	-1.016346
C	-2.967513	0.043672	-1.129907
C	-0.931821	-0.637514	-0.039786
C	-3.721257	-0.745767	-0.273807
H	-3.445141	0.614938	-1.910749
C	-1.656484	-1.443249	0.838882
H	0.140706	-0.596060	0.036213
C	-3.042163	-1.473283	0.711841
H	-3.619001	-2.082548	1.389432
H	0.797238	1.754561	-3.020081
C	1.662356	0.454613	-1.594110
C	2.266361	1.426483	-0.799121

C	2.153604	-0.844525	-1.572858
C	3.319109	1.110176	0.056909
H	1.914269	2.445535	-0.862955
C	3.227583	-1.197225	-0.753369
H	1.663815	-1.560250	-2.213774
C	3.780166	-0.208654	0.061734
H	4.606355	-0.467224	0.708410
C	-0.904513	-2.268712	1.886705
C	-5.247852	-0.833429	-0.357871
C	3.832670	-2.606032	-0.744665
C	3.974080	2.143437	0.980054
C	-5.855263	-0.317190	0.959245
H	-5.577565	0.724088	1.133113
H	-6.944814	-0.375502	0.920491
H	-5.518266	-0.902868	1.814456
C	-5.816238	0.001665	-1.509866
H	-5.585530	1.061838	-1.395276
H	-5.433397	-0.328809	-2.476348
H	-6.902145	-0.099780	-1.529923
C	-5.667271	-2.298836	-0.571249
H	-5.335977	-2.936609	0.248136
H	-6.754658	-2.372915	-0.635059
H	-5.244237	-2.691874	-1.497194
C	-1.848950	-2.976032	2.863342
H	-2.501171	-3.687144	2.355030
H	-1.262785	-3.532249	3.596203
H	-2.474949	-2.265871	3.406483
C	0.033486	-1.356347	2.697714
H	0.777399	-0.873043	2.065818
H	-0.530312	-0.578719	3.216269
H	0.568245	-1.942969	3.446957
C	-0.066642	-3.331234	1.154459
H	0.496294	-3.932744	1.870787
H	-0.707304	-3.999163	0.576184
H	0.640557	-2.867918	0.469006
C	3.026590	-3.589639	-1.599928
H	3.000943	-3.288397	-2.647490
H	3.487620	-4.577447	-1.549592
H	1.998399	-3.682345	-1.249239
C	3.889484	-3.150103	0.693816
H	4.311298	-4.157284	0.696806
H	4.511121	-2.528469	1.337763
H	2.892907	-3.199061	1.132378
C	5.263691	-2.533285	-1.309157
H	5.894701	-1.876764	-0.709398
H	5.719416	-3.525941	-1.317317
H	5.257938	-2.153078	-2.332154
C	3.377776	3.544162	0.804376
H	3.508552	3.914972	-0.213171
H	2.312971	3.563963	1.038692
H	3.878603	4.239969	1.479198
C	5.482111	2.224352	0.683774
H	5.956219	2.959286	1.337665
H	5.976840	1.266534	0.842991
H	5.657769	2.525911	-0.350318
C	3.767511	1.711290	2.443528
H	2.704518	1.649564	2.683956
H	4.212684	0.735797	2.639320
H	4.227355	2.433872	3.121100

E-TS4-tBu

Number of imaginary frequencies: 1

Electronic Energy (SCF) = -1801.10966274 a.u

ZPV corrected Energy = -1800.329304 a.u.

Enthalpy = -1800.287962 a.u.

Free Energy = -1800.403505 a.u.

S	0.127847	0.365851	1.575095
C	-1.137009	-0.404754	2.650868
H	-2.091224	-0.375636	2.131651
H	-1.205583	0.203550	3.551408
C	1.431868	-0.853126	2.011159
H	2.337220	-0.306842	2.257158
H	1.619417	-1.454878	1.125935
C	0.876813	-1.681332	3.168363
H	1.069074	-1.171899	4.114064
H	1.371681	-2.651963	3.202995
C	-0.629257	-1.809381	2.949482
H	-0.831463	-2.462780	2.099244
H	-1.138273	-2.223132	3.820031
C	-0.428851	-0.321837	-0.495278
C	0.443188	0.400423	-1.489304
O	0.050623	-0.343949	-2.555730
H	0.168261	1.470565	-1.537055
C	1.918869	0.338617	-1.113395
C	2.525816	1.388669	-0.433704
C	2.655169	-0.805749	-1.392753
C	3.851252	1.306233	-0.003419
H	1.946664	2.281482	-0.234992
C	3.980884	-0.931748	-0.980238
H	2.161249	-1.597066	-1.938729
C	4.557243	0.134518	-0.283546
H	5.580051	0.053150	0.041927
H	-0.110597	-1.346161	-0.349956
C	-1.886925	-0.122039	-0.492087
C	-2.722119	-1.209871	-0.242260
C	-2.447200	1.143380	-0.662609
C	-4.105770	-1.060694	-0.172197
H	-2.271170	-2.182930	-0.105450
C	-3.823925	1.333690	-0.598184
H	-1.793542	1.983449	-0.845080
C	-4.632603	0.219193	-0.352644
H	-5.699181	0.353872	-0.300224
C	-4.984555	-2.283831	0.108325
C	-4.399718	2.740224	-0.794227
C	4.745613	-2.225612	-1.280546
C	4.473838	2.478266	0.763291
C	3.675629	2.714755	2.058577
H	4.100566	3.551091	2.617608
H	2.631557	2.946224	1.849429
H	3.703565	1.831083	2.698949
C	5.936724	2.223008	1.141628
H	6.561988	2.070464	0.260819
H	6.327959	3.086837	1.681230
H	6.042554	1.351854	1.789828
C	4.416074	3.748145	-0.104461
H	4.971278	3.608077	-1.033575
H	3.390911	4.013126	-0.362232
H	4.856362	4.591783	0.431224
C	4.024202	-3.404249	-0.600808
H	2.999819	-3.505068	-0.959044
H	4.547592	-4.339877	-0.808925
H	3.993201	-3.267226	0.481895
C	4.776355	-2.461096	-2.801154
H	5.312689	-3.384525	-3.029736
H	3.771854	-2.544331	-3.215107
H	5.282649	-1.638978	-3.310136
C	6.190164	-2.189425	-0.770587
H	6.762805	-1.384561	-1.233670
H	6.235920	-2.061176	0.311938
H	6.684895	-3.131122	-1.013287
C	-4.581945	-2.896241	1.462380
H	-5.202730	-3.767286	1.681065
H	-3.541097	-3.219235	1.463758
H	-4.713515	-2.174365	2.270514
C	-4.773849	-3.327905	-1.002800
H	-5.391742	-4.208027	-0.813722

H	-5.050944	-2.919619	-1.976062
H	-3.734976	-3.652725	-1.058601
C	-6.474971	-1.933221	0.163721
H	-6.693767	-1.215671	0.955889
H	-6.828951	-1.518043	-0.780850
H	-7.051962	-2.836616	0.365988
C	-3.821638	3.679936	0.279633
H	-2.735211	3.741328	0.217519
H	-4.222208	4.687652	0.152994
H	-4.084020	3.333583	1.281049
C	-4.003506	3.259234	-2.188320
H	-4.402301	4.263817	-2.342967
H	-2.921201	3.304453	-2.307423
H	-4.400653	2.610594	-2.970873
C	-5.927869	2.769093	-0.686269
H	-6.399629	2.142849	-1.444753
H	-6.272700	2.436741	0.294071
H	-6.281644	3.790473	-0.833219

F-TS4-tBu
Number of imaginary frequencies: 1
Electronic Energy (SCF) = -1801.10953415 a.u
ZPV corrected Energy = -1800.328834 a.u.
Enthalpy = -1800.287591 a.u.
Free Energy = -1800.402263 a.u.

S	0.153181	0.447096	1.544929
C	-1.114379	-0.235916	2.683367
H	-1.882178	-0.707054	2.073279
H	-1.561795	0.591983	3.226996
C	1.439779	-0.760361	2.041312
H	2.119563	-0.245725	2.717594
H	1.985815	-1.057350	1.151486
C	0.695374	-1.894307	2.732858
H	1.383219	-2.488758	3.334360
H	0.247264	-2.552127	1.985962
C	-0.394005	-1.242067	3.580715
H	-1.104369	-1.970580	3.971537
H	0.059045	-0.725927	4.428617
C	-0.404639	-0.341992	-0.479341
H	-0.079381	-1.357397	-0.290076
C	0.452428	0.330641	-1.526418
H	0.166462	1.394524	-1.627799
O	0.059913	-0.471409	-2.548038
C	-1.864396	-0.152507	-0.483040
C	-2.693684	-1.241897	-0.213680
C	-2.427217	1.108597	-0.686268
C	-4.078293	-1.097107	-0.157078
H	-2.234276	-2.206491	-0.059581
C	-3.804441	1.292275	-0.634071
H	-1.771893	1.941616	-0.884052
C	-4.604902	0.176748	-0.369116
H	-5.676500	0.307843	-0.326085
C	1.930242	0.301171	-1.157305
C	2.519242	1.383279	-0.501428
C	2.677996	-0.839588	-1.396575
C	3.834043	1.323190	-0.051783
H	1.924488	2.270926	-0.340821
C	4.004863	-0.940912	-0.964441
H	2.196447	-1.653083	-1.920326
C	4.555982	0.146009	-0.291265
H	5.575544	0.089827	0.055994
C	4.783894	-2.234921	-1.220790
C	4.505550	2.477394	0.699724
C	-4.462036	2.658918	-0.848891
C	-5.019472	-2.268389	0.140511
C	4.062864	-3.402156	-0.521244
H	4.015066	-3.237919	0.557053
H	3.044633	-3.521947	-0.890799
H	4.597556	-4.337816	-0.697897
C	4.837302	-2.507655	-2.734487
H	5.344986	-1.694761	-3.256728
H	5.383064	-3.432587	-2.932468
H	3.839173	-2.608437	-3.159817
C	6.220511	-2.172300	-0.691756
H	6.727276	-3.115117	-0.903228
H	6.792178	-1.373984	-1.167239
H	6.249304	-2.016089	0.387726
C	5.756767	2.936373	-0.070080
H	6.241509	3.762118	0.455003
H	6.484586	2.131539	-0.172321
H	5.491075	3.279470	-1.071494
C	3.574611	3.683071	0.866389
H	3.260822	4.088192	-0.096573
H	2.680049	3.427972	1.436411
H	4.097527	4.474502	1.405474
C	4.922291	1.994952	2.101154
H	5.408268	2.803943	2.650532
H	4.051412	1.671547	2.674426
H	5.619980	1.159336	2.047122
C	-4.262772	-3.586351	0.336166
H	-3.570414	-3.535155	1.178000
H	-4.975103	-4.386520	0.541567
H	-3.697750	-3.864524	-0.554452
C	-6.003192	-2.444292	-1.029773
H	-6.681853	-3.275620	-0.828827
H	-6.605910	-1.549829	-1.186515
H	-5.468427	-2.657342	-1.956805
C	-5.808249	-1.968825	1.428206
H	-6.483198	-2.795783	1.657479
H	-5.132473	-1.837078	2.275386
H	-6.407617	-1.063654	1.331637
C	-5.437422	2.574092	-2.036585
H	-4.911751	2.291166	-2.950034
H	-6.224798	1.841403	-1.859986
H	-5.911955	3.543234	-2.202590
C	-3.436060	3.757264	-1.146849
H	-2.733148	3.890044	-0.323054
H	-2.865471	3.542620	-2.051382
H	-3.953622	4.705711	-1.296918
C	-5.238708	3.053911	0.420252
H	-5.715689	4.026245	0.282162
H	-6.017560	2.329354	0.658017
H	-4.568612	3.123227	1.279153

G-TS4-tBu
Number of imaginary frequencies: 1
Electronic Energy (SCF) = -1801.10900132 a.u
ZPV corrected Energy = -1800.328188 a.u.
Enthalpy = -1800.287075 a.u.
Free Energy = -1800.400697 a.u.

S	0.277930	-0.199586	1.607006
C	-1.090607	0.248557	2.743640
H	-1.514813	1.194689	2.419327
H	-0.642934	0.381100	3.727261
C	-0.237887	-1.951501	1.461418
H	0.650912	-2.569862	1.541531
H	-0.681617	-2.086490	0.479064
C	-1.252151	-2.188810	2.577929
H	-0.729656	-2.396839	3.513008
H	-1.871093	-3.053534	2.338811
C	-2.079181	-0.912230	2.712733
H	-2.744781	-0.804517	1.857942
H	-2.686854	-0.913618	3.617560
C	-0.265383	0.638059	-0.382528

C	0.647439	0.023713	-1.421682
H	0.386580	-1.040345	-1.578544
O	0.299999	0.853416	-2.436960
C	2.108464	0.048637	-0.986165
C	2.814860	1.245542	-1.001842
C	2.737443	-1.104698	-0.533120
C	4.131960	1.314087	-0.551124
H	2.311726	2.121768	-1.386555
C	4.055569	-1.082873	-0.072863
H	2.182592	-2.034504	-0.542673
C	4.730979	0.138423	-0.087259
H	5.748385	0.175291	0.262335
H	0.054259	1.639521	-0.121376
C	-1.723961	0.467188	-0.471260
C	-2.559450	1.459858	0.041897
C	-2.293461	-0.690830	-1.002459
C	-3.943367	1.305715	0.061747
H	-2.107805	2.363058	0.428507
C	-3.670737	-0.881869	-1.001118
H	-1.648185	-1.442665	-1.429574
C	-4.475117	0.123574	-0.457054
H	-5.542378	-0.014917	-0.446602
C	4.706079	-2.377215	0.427398
C	4.872731	2.655809	-0.577961
C	-4.819856	2.416061	0.650420
C	-4.249939	-2.178953	-1.575795
C	6.148100	-2.166180	0.900926
H	6.203086	-1.460079	1.730767
H	6.559764	-3.116052	1.245580
H	6.788511	-1.799031	0.097796
C	4.718507	-3.415105	-0.709140
H	3.710381	-3.640051	-1.057224
H	5.294687	-3.050177	-1.561127
H	5.172029	-4.347289	-0.365539
C	3.888903	-2.932707	1.608038
H	4.346562	-3.848411	1.988280
H	3.843521	-2.209611	2.424361
H	2.867593	-3.168450	1.310206
C	6.299031	2.553206	-0.026772
H	6.309467	2.220446	1.012288
H	6.911350	1.865120	-0.611296
H	6.774565	3.534464	-0.065784
C	4.951350	3.162841	-2.029309
H	5.496609	2.456158	-2.657486
H	3.959625	3.299068	-2.460059
H	5.470900	4.122777	-2.067032
C	4.101320	3.680504	0.273646
H	4.616399	4.643454	0.264940
H	3.091544	3.836642	-0.105166
H	4.025596	3.345011	1.309744
C	-3.828790	-2.316229	-3.049615
H	-2.744772	-2.337641	-3.159308
H	-4.228161	-3.241188	-3.470405
H	-4.208696	-1.481036	-3.640280
C	-3.699082	-3.373763	-0.775827
H	-2.611395	-3.423535	-0.825900
H	-3.987399	-3.303243	0.274555
H	-4.095962	-4.309656	-1.173982
C	-5.780048	-2.219252	-1.506456
H	-6.142442	-2.160294	-0.479042
H	-6.232284	-1.405915	-2.075577
H	-6.136959	-3.159162	-1.929781
C	-6.312548	2.074891	0.596247
H	-6.657846	1.928416	-0.428075
H	-6.544159	1.174326	1.166978
H	-6.887208	2.896499	1.025917
C	-4.430618	2.642334	2.122670
H	-3.388615	2.946272	2.220886
H	-5.050076	3.428305	2.559023
H	-4.575488	1.732425	2.707721
C	-4.591455	3.716532	-0.140267
H	-5.207240	4.519477	0.269826
H	-3.550329	4.036024	-0.096928
H	-4.858950	3.583954	-1.189874

H-TS4-tBu

Number of imaginary frequencies: 1
Electronic Energy (SCF) = -1801.10801364 a.u
ZPV corrected Energy = -1800.327538 a.u.
Enthalpy = -1800.286250 a.u.
Free Energy = -1800.401839 a.u.

S	0.313251	-0.125958	1.552029
C	-0.936501	0.420843	2.772189
H	-1.707372	0.975351	2.240994
H	-0.442804	1.079005	3.482729
C	-0.312297	-1.850657	1.482114
H	0.447413	-2.474098	1.948514
H	-0.415374	-2.138067	0.440172
C	-1.628639	-1.866818	2.256013
H	-1.834906	-2.871825	2.623791
H	-2.443502	-1.573485	1.595683
C	-1.495686	-0.856426	3.392651
H	-2.453655	-0.652439	3.871279
H	-0.810288	-1.231781	4.154183
C	-0.270811	0.675125	-0.429875
C	0.634630	0.055468	-1.479065
H	0.356787	-1.005360	-1.632156
O	0.299120	0.883714	-2.496973
C	2.095937	0.051232	-1.043784
C	2.683014	-1.112742	-0.558911
C	2.835836	1.227907	-1.082843
C	3.994971	-1.119282	-0.084197
H	2.094496	-2.019153	-0.553299
C	4.150716	1.264786	-0.621758
H	2.357271	2.104956	-1.491170
C	4.703820	0.081774	-0.123209
H	5.721964	0.096862	0.238901
H	0.041991	1.684517	-0.191789
C	-1.727801	0.487262	-0.502481
C	-2.568411	1.471321	0.023626
C	-2.285587	-0.679530	-1.030386
C	-3.950131	1.300030	0.054768
H	-2.118397	2.375528	0.404517
C	-3.659869	-0.883798	-1.019575
H	-1.629661	-1.422179	-1.452369
C	-4.467310	0.113910	-0.465614
H	-5.536698	-0.037702	-0.444648
C	-4.306763	-2.155541	-1.577208
C	-4.898490	2.344425	0.651262
C	4.663780	-2.377690	0.479488
C	4.997156	2.542432	-0.638492
C	5.928586	-2.700369	-0.335927
H	6.648830	-1.883203	-0.300863
H	6.415443	-3.594517	0.059219
H	5.678614	-2.883608	-1.382390
C	3.739473	-3.599342	0.433305
H	2.836389	-3.446230	1.026053
H	3.440136	-3.842418	-0.587111
H	4.261246	-4.465942	0.842186
C	5.056223	-2.127935	1.947148
H	4.176141	-1.892450	2.548598
H	5.529644	-3.017011	2.369218
H	5.757113	-1.298342	2.039339
C	6.258392	2.310637	-1.490146
H	6.870506	3.214809	-1.512760
H	6.870151	1.501582	-1.090985

H	5.990775	2.055522	-2.516987
C	4.236068	3.736662	-1.223667
H	3.931016	3.555708	-2.255029
H	3.343570	3.972148	-0.642317
H	4.880092	4.617513	-1.215933
C	5.416151	2.895482	0.800250
H	6.021581	3.804410	0.805678
H	4.539641	3.067428	1.427767
H	6.003943	2.098311	1.255365
C	-3.271388	-3.137136	-2.135953
H	-2.697981	-2.698770	-2.953607
H	-2.571720	-3.469295	-1.367444
H	-3.781240	-4.020399	-2.522970
C	-5.086104	-2.863595	-0.454184
H	-5.556102	-3.771386	-0.837496
H	-4.419989	-3.145933	0.363104
H	-5.871037	-2.227821	-0.044662
C	-5.277077	-1.779152	-2.711056
H	-5.742577	-2.677515	-3.121317
H	-6.071796	-1.121142	-2.359460
H	-4.749425	-1.269486	-3.518820
C	-5.641259	1.720689	1.846911
H	-6.323358	2.449309	2.289456
H	-6.226875	0.851938	1.545950
H	-4.937433	1.402279	2.618343
C	-4.155617	3.591188	1.142413
H	-3.440893	3.353484	1.931731
H	-3.618348	4.086943	0.332786
H	-4.873562	4.303779	1.550612
C	-5.921882	2.779233	-0.412483
H	-6.521848	1.939107	-0.761776
H	-6.601302	3.526017	0.003004
H	-5.420374	3.218319	-1.276454

TS between A-anti-tBu and D-anti-tBu

Number of imaginary frequencies: 1
Electronic Energy (SCF) = -1801.10822553 a.u
ZPV corrected Energy = -1800.326595 a.u.
Enthalpy = -1800.286219 a.u.
Free Energy = -1800.397276 a.u.

S	0.563043	3.096132	-0.355961
C	1.286138	2.900605	1.317519
H	1.187309	1.862072	1.614491
H	0.668030	3.520569	1.965088
C	1.872195	4.269271	-0.915865
H	1.378083	5.108915	-1.396327
H	2.473659	3.737194	-1.648380
C	2.673029	4.643909	0.329051
H	2.184092	5.465539	0.853665
H	3.668009	4.976699	0.034931
C	2.717383	3.406992	1.221871
H	3.351106	2.638057	0.778516
H	3.101009	3.632706	2.216490
C	0.923493	1.530356	-1.392860
H	1.669821	1.911770	-2.082791
C	-0.317004	1.203950	-2.331240
H	-0.671790	2.224651	-2.639914
O	0.158025	0.452899	-3.319477
C	1.511367	0.424321	-0.581680
C	2.812026	0.011439	-0.867235
C	0.801158	-0.208610	0.439327
C	3.411649	-1.030674	-0.162865
H	3.342957	0.512846	-1.661760
C	1.373954	-1.240613	1.177321
H	-0.202469	0.115727	0.654462
C	2.673159	-1.635507	0.854226
H	3.124829	-2.441089	1.414796
C	-1.492630	0.596778	-1.545857
C	-1.734842	-0.771068	-1.655524
C	-2.321568	1.364227	-0.739230
C	-2.781704	-1.378843	-0.970514
H	-1.074820	-1.322659	-2.305922
C	-3.368190	0.790249	-0.008984
H	-2.157958	2.431521	-0.679964
C	-3.582513	-0.578190	-0.144969
H	-4.396446	-1.039584	0.390980
C	0.628252	-1.945463	2.314324
C	4.832715	-1.519020	-0.461434
C	-3.108801	-2.869174	-1.124449
C	-4.221358	1.674468	0.906896
C	1.433746	-1.808880	3.618647
H	0.910395	-2.306484	4.437597
H	2.422720	-2.258578	3.531682
H	1.563062	-0.758624	3.887375
C	0.476854	-3.436908	1.965812
H	-0.065389	-3.562664	1.028499
H	1.447585	-3.921437	1.859648
H	-0.074629	-3.955997	2.752291
C	-0.765124	-1.352210	2.546822
H	-0.711059	-0.302195	2.838757
H	-1.392528	-1.424375	1.660200
H	-1.262096	-1.894583	3.352437
C	5.705222	-1.346648	0.795012
H	5.315872	-1.918580	1.637338
H	6.722459	-1.690791	0.597602
H	5.753605	-0.297644	1.093353
C	4.788272	-3.008057	-0.848700
H	5.794776	-3.370161	-1.067923
H	4.379049	-3.620440	-0.045242
H	4.170782	-3.158913	-1.735616
C	5.485426	-0.743446	-1.610414
H	4.922557	-0.846759	-2.538903
H	5.575589	0.319780	-1.382262
H	6.490040	-1.131121	-1.785039
C	-3.281447	-3.533607	0.252536
H	-2.367673	-3.457488	0.840986
H	-4.090530	-3.083112	0.826723
H	-3.515252	-4.592846	0.127240
C	-4.425693	-3.003530	-1.911630
H	-4.684747	-4.056448	-2.045104
H	-5.249666	-2.515718	-1.389284
H	-4.335288	-2.547080	-2.898791
C	-2.009894	-3.624435	-1.880547
H	-1.894368	-3.260050	-2.901388
H	-1.045084	-3.535256	-1.379297
H	-2.265440	-4.684130	-1.933465
C	-5.310845	0.883556	1.638594
H	-4.887360	0.101554	2.270552
H	-5.881233	1.556905	2.280463
H	-6.009674	0.417927	0.942335
C	-4.900767	2.776362	0.074677
H	-4.169420	3.405022	-0.433283
H	-5.554120	2.341384	-0.683626
H	-5.506621	3.418161	0.718039
C	-3.311729	2.327918	1.964178
H	-2.816413	1.567648	2.570902
H	-2.541098	2.944832	1.501762
H	-3.898680	2.964906	2.629391

References

[1] S. Grimme, *J. Chem. Theory Comput.* **2019**, *15*, 2847–2862.
[2] P. Pracht, F. Bohle, S. Grimme, *Phys. Chem. Chem. Phys.* **2020**, *22*, 7169–7192.
[3] A. D. Becke, *The Journal of Chemical Physics* **1993**, *98*, 5648–5652.
[4] C. Lee, W. Yang, R. G. Parr, *Phys. Rev. B* **1988**, *37*, 785–789.
[5] F. Weigend, R. Ahlrichs, *Phys. Chem. Chem. Phys.* **2005**, *7*, 3297–3305.
[6] S. Grimme, J. Antony, S. Ehrlich, H. Krieg, *J. Chem. Phys.* **2010**, *132*, 154104.
[7] S. Grimme, S. Ehrlich, L. Goerigk, *J. Comput. Chem.* **2011**, *32*, 1456–1465.
[8] A. V. Marenich, C. J. Cramer, D. G. Truhlar, *J. Phys. Chem. B* **2009**, *113*, 6378–6396.
[9] Frisch, M. J.; Trucks, G. W.; Schlegel, H. B.; Scuseria, G. E.; Robb, M. A.; Cheeseman, J. R.; Scalmani, G.; Barone, V.; Petersson, G. A.; Nakatsuji, H.; Li, X.; Caricato, M.; Marenich, A. V.; Bloino, J.; Janesko, B. G.; Gomperts, R.; Mennucci, B.; Hratchian, H. P.; Ortiz, J. V.; Izmaylov, A. F.; Sonnenberg, J. L.; Williams-Young, D.; Ding, F.; Lipparini, F.; Egidi, F.; Goings, J.; Peng, B.; Petrone, A.; Henderson, T.; Ranasinghe, D.; Zakrzewski, V. G.; Gao, J.; Rega, N.; Zheng, G.; Liang, W.; Hada, M.; Ehara, M.; Toyota, K.; Fukuda, R.; Hasegawa, J.; Ishida, M.; Nakajima, T.; Honda, Y.; Kitao, O.; Nakai, H.; Vreven, T.; Throssell, K.; Montgomery, J. A., Jr.; Peralta, J. E.; Ogliaro, F.; Bearpark, M. J.; Heyd, J. J.; Brothers, E. N.; Kudin, K. N.; Staroverov, V. N.; Keith, T. A.; Kobayashi, R.; Normand, J.; Raghavachari, K.; Rendell, A. P.; Burant, J. C.; Iyengar, S. S.; Tomasi, J.; Cossi, M.; Millam, J. M.; Klene, M.; Adamo, C.; Cammi, R.; Ochterski, J. W.; Martin, R. L.; Morokuma, K.; Farkas, O.; Foresman, J. B.; Fox, D. J. Gaussian, Inc., Wallingford CT, 2016.
[10] J. Contreras-García, E. R. Johnson, S. Keinan, R. Chaudret, J.-P. Piquemal, D. N. Beratan, W. Yang, *J. Chem. Theory Comput.* **2011**, *7*, 625–632.
[11] R. A. Boto, F. Peccati, R. Laplaza, C. Quan, A. Carbone, J.-P. Piquemal, Y. Maday, J. Contreras-García, *J. Chem. Theory Comput.* **2020**, *16*, 4150–4158.
[12] T. Lu, F. Chen, *J. Comput. Chem.* **2012**, *33*, 580–592.
[13] W. Humphrey, A. Dalke, K. Schulten, *J. Mol. Graph.* **1996**, *14*, 33–38.
[14] E. D. Glendening, C. R. Landis, F. Weinhold, *WIREs Comput. Mol. Sci.* **2012**, *2*, 1–42.
[15] R. M. Parrish, J. F. Gonthier, C. Corminbœuf, C. D. Sherrill, *J. Chem. Phys.* **2015**, *143*, 051103.
[16] E. Pastorczak, A. Prlj, J. F. Gonthier, C. Corminboeuf, *J. Chem. Phys.* **2015**, *143*, 224107.
[17] D. Luu, K. Patkowski, *J. Phys. Chem. A* **2023**, *127*, 356–377.
[18] R. M. Parrish, L. A. Burns, D. G. A. Smith, A. C. Simmonett, A. E. DePrince, E. G. Hohenstein, U. Bozkaya, A. Yu. Sokolov, R. Di Remigio, R. M. Richard, J. F. Gonthier, A. M. James, H. R. McAlexander, A. Kumar, M. Saitow, X. Wang, B. P. Pritchard, P. Verma, H. F. Schaefer, K. Patkowski, R. A. King, E. F. Valeev, F. A. Evangelista, J. M. Turney, T. D. Crawford, C. D. Sherrill, *J. Chem. Theory Comput.* **2017**, *13*, 3185–3197.
